# Fe-Catalyzed Fluoroalkyl(hetero)arylation of Vinyl Azaarenes: Rapid and Modular Synthesis of Unsymmetrical 1,1-Bis(hetero)arylalkanes

**DOI:** 10.1021/acs.orglett.4c02515

**Published:** 2024-08-14

**Authors:** Macayla Guerrero, Ángel Rentería-Gómez, Deborshee Das, Osvaldo Gutierrez

**Affiliations:** Department of Chemistry, Texas A&M University, College Station, Texas 77843, United States

## Abstract

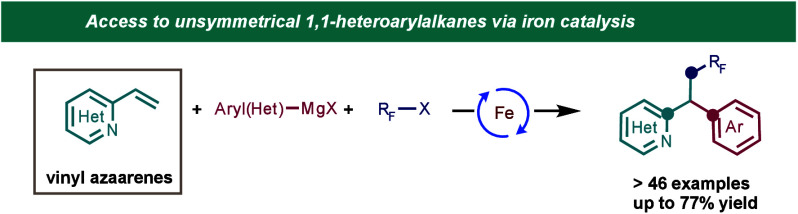

In contrast to transition-metal-catalyzed difunctionalization of activated alkenes, selective alkylarylation of vinyl azaarenes is underdeveloped. Consequently, the lack of modular and rapid syntheses of 1,1-bis(hetero)arylalkanes limits their exploration in medicinal chemistry. Herein we report a protocol using commercially available iron salts, bisphosphine ligands, fluoroalkyl halides, and Grignard reagents that enables the selective 1,2-fluoroalkyl(hetero)arylation of vinyl azaarenes. We demonstrate the versatility and robustness of the method through the selective synthesis of a range of unsymmetrical 1,1-bis(hetero)arylalkenes, including pyridine *N*-oxides, triazoles, pyrazines, carbazoles, indazoles, and 1,2-azaborines. Mechanistic insights from experimental and computational investigations support a radical pathway and provide insights into the role of non-covalent interactions in iron catalysis.

Aromatic nitrogen-containing heterocycles are vital scaffolds in the synthesis of new pharmaceuticals, agrochemicals, and materials.^1^ Due to their ability to participate in a wide range of intermolecular interactions in biological systems^2^ these scaffolds are abundant in FDA-approved small-molecule drugs, with pyridine being the second most frequently found ring system.^3^ In this vein, 1,1-diarylalkanes are a common and important motif in pharmaceutical and agricultural chemistry (Scheme 1a).^4,5^ Traditionally, 1,1-diarylalkanes are synthesized through two general reaction types: two- or three-component reactions. In contrast to two-component variants, three-component reactions conveniently permit the rapid and modular synthesis of complex molecules through the union of three (often readily accessible) chemical building blocks and thus allow for greater diversity and efficiency. However, the synthesis of 1,1-diarylalkanes through transition-metal-catalyzed three-component dicarbofunctionalization of alkenes has been limited to the use of styrenyl or alkenyl olefins.^6−9^ Recently, Guo^10^ and Liu^11^ reported the use of nickel and copper as catalysts with bipyridine ligands to enable the three-component difunctionalization of vinyl azaarenes to form 1,1-diarylalkenes (Scheme 1b). However, a major drawback of these methods is the use of designed cycloalkylsilyl peroxides as radical precursors in the sole example by Guo. In addition, the requisite for excess aryl and alkyl coupling partners (>3 equiv) and extended reaction times (>72 h) by the Liu group limit applicability in medicinal chemistry. Builing upon prior work in our lab on multicomponent iron-catalyzed cross-coupling reactions,^12^ we questioned whether we could use vinyl azaarenes as effective cross-coupling partners in this transformation to access unsymmetrical 1,1-diarylalkenes.

**Scheme 1:**
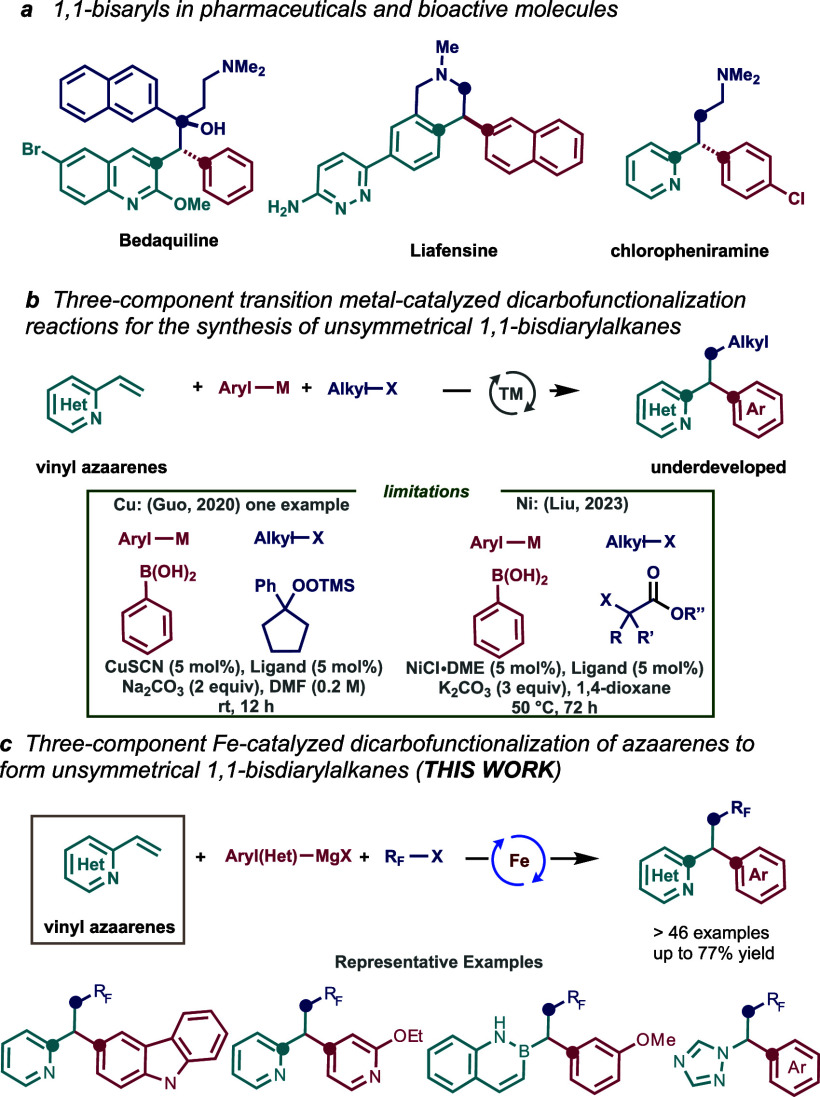
Importance of 1,1-Diarylalkanes and Current Three-Component Transition-Metal-Catalyzed Dicarbofunctionalizion Strategies to Access These Scaffolds

Herein, we report the use of a bisphosphine–iron catalytic system that allowed three-component dicarbofunctionalization of vinyl azaarenes with fluoroalkyl bromides and (hetero)aryl Grignard reagents, leading to the synthesis of previously inaccessible unsymmetrical 1,1-bis(hetero)arylalkanes at low temperatures with short reaction times.

To evaluate the feasibility of our designed multicomponent strategy, we selected 2-vinylpyridine (**1a**) (1.0 equiv), 2-(2-bromo-1,1,2,2-tetrafluoroethoxy)anisole (**2a**) (2.0 equiv), and 3-methoxyphenylmagnesium bromide (**3a**) (2.0 equiv) as the model substrates (Table 1). Gratifyingly, after extensive reaction screening (see the Supporting Information for full details), we identified 1,2-bis(dicyclohexylphosphino)ethane (dcpe) ($140/g, Sigma-Aldrich) and FeCl_3_ (∼$9/g, Sigma-Aldrich) as a suitable catalytic system that enables the formation of the desired product in good yield (entry 12). Notably, as shown in Table 1, we found a pronounced effect on the overall efficiency impacted by the position of the nitrogen atom in the aromatic ring. The most striking result is the lack of reactivity of obvious styrenyl substrate **1a-[N]**, which has been widely utilized in transition-metal-catalyzed dicarbofunctionalization reactions.^6−9,13^ As shown by the reactivity of **1a-N**, **1a-3N**, and **1a-4N**, it is likely that having a nitrogen atom in closer proximity to the reactive center promotes selective radical cross-coupling through weak interactions with the iron catalyst (*vide infra*). Overall, the optimized conditions consisted of FeCl_3_ (20 mol %) and dcpe (40 mol %) in THF at 0 °C to give the desired product **4a** in 77% yield, although lowering the loadings of catalyst (5 mol %) and ligand (10 mol %) led to only a slight decrease in yield (55%; entry 11).

**Table 1:**
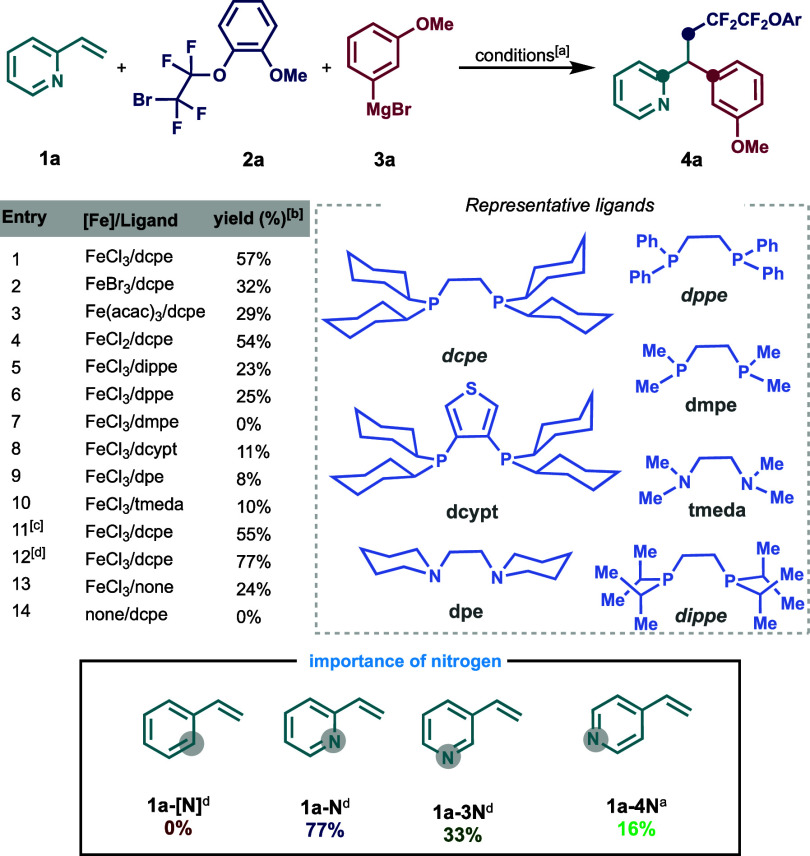
Optimization of the Reaction Conditions

a Reaction conditions: **1a** (0.2 mmol, 1.0 equiv), **2a** (0.4 mmol, 2.0 equiv), Fe catalyst (10 mol %), ligand (20 mol %), THF (*c* 1.0 M), 0 °C, slow addition of **3a** (0.4 mmol, 2.0 equiv) in 1 h, nitrogen atmosphere.

b Isolated yields.

c FeCl_3_ catalyst (5 mol %), dcpe (10 mol %).

d **1a** (0.2 mmol, 1.0 equiv), **2a** (0.3 mmol), FeCl_3_ catalyst (20 mol %), dcpe (40 mol %), **3a** (0.3 mmol, 1.5 equiv).

As shown in Scheme 2, a wide range of aryl and heteroaryl Grignard reagents, even those bearing traditional cross-coupling C–Cl synthetic handles, were compatible in this one-pot protocol to form the corresponding unsymmetrical 1,1-diarylalkanes. Notably, extended π systems work well in this transformation (**4p**) in addition to sterically encumbered *ortho*-substituted systems (**4q** and **4s**). To our delight, employing heteroaromatic turbo Grignard reagents, using MgBr and LiCl, worked well in this transformation, yielding the unsymmetrical 1,1-bisheteroarylalkanes, including those bearing substituted benzofurans (**4t**), carbazoles (**4u**), *N*-methyl-substituted indazoles (**4v**), and 2-ethoxypyridines (**4w**).

**Scheme 2:**
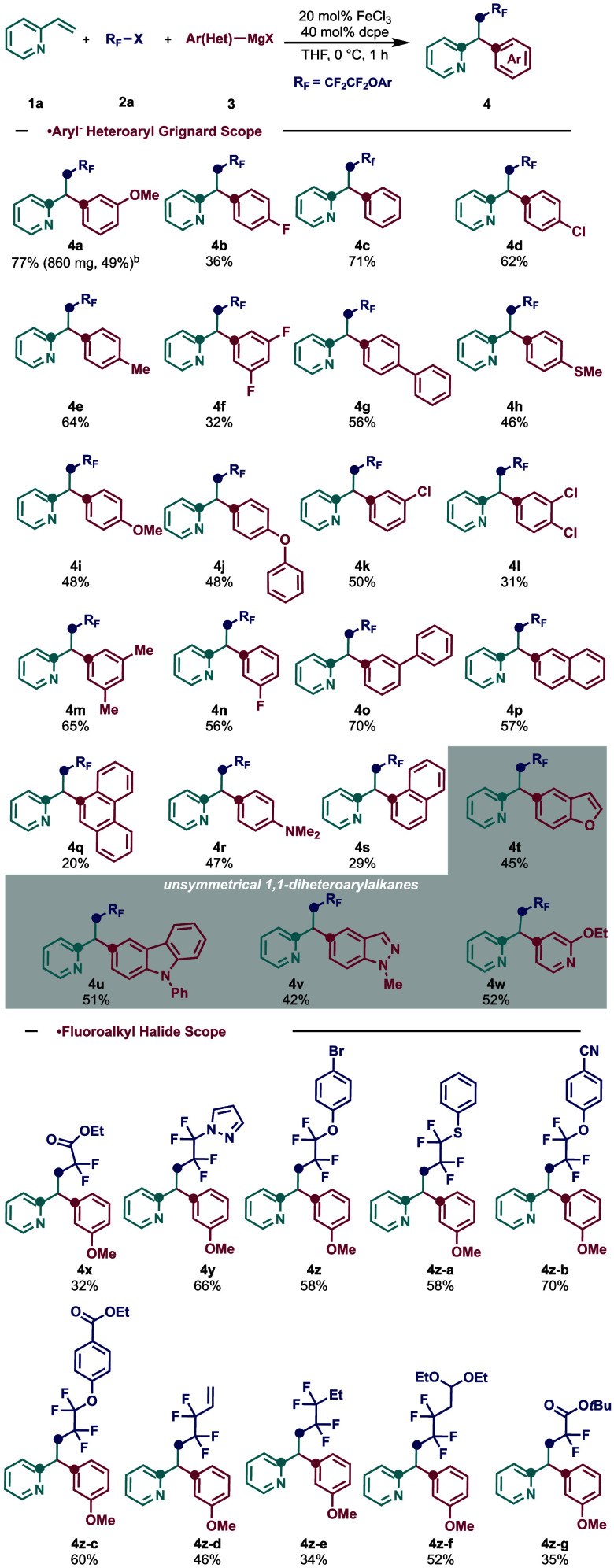
Reaction Scope in the Three-Component Dicarbofunctionalizion Transformation with 2-Vinylpyridine (**1a**) Reaction conditions: **1a** (0.2 mmol, 1.0 equiv), **2a** (0.3 mmol, 1.5 equiv), Fe catalyst (20 mol %), ligand dcpe (40 mol %), THF (*c* 1.0 M), 0 °C, slow addition of **3a** (0.3 mmol, 1.5 equiv) in 1 h, nitrogen atmosphere. Using a 4.0 mmol scale.

Next, we examined the scope of fluoroalkyl halides as radical precursors in this three-component coupling reaction. Overall, a wide range of di- and tetrafluorobromides as radical precursors bearing various functionalities, including those bearing diverse and versatile functional groups such as esters (**4x**, **4z-c**, **4z-g**) halogens (**4z**), nitrile (**4z-b**), (thio)ethers (**4z-a**, **4z**–**f**) and pendant alkenes (**4z-d**).

Due to the importance of azaarene compounds, the scope of vinyl azaarenes as radical linchpins in this multicomponent radical cross-coupling reaction was investigated (Scheme 3). Overall, a range of different functionalities of vinyl azaarene compounds were compatible with this transformation, leading to the desired 1,1-diarylalkanes containing quinoline (**5a**), 2-, 3-, and 4-picolines (**5b**, **5c**, **5d**), 2-, 3-, and 4-bromopyridines (**5e**, **5f**, **5g**), 4-methoxypyridine (**5h**), *N*-alkyltriazole (**5i**), pyrazine (**5j**), and pyridine *N*-oxide (**5k**) after oxidation of **4a**. Remarkably, this work also provides direct access to azaborines (**5l**). Given the importance of azaborines as isosteres for naphthalenes, we anticipate that this work can expand utility of these systems in medicinal chemistry, and current efforts to develop asymmetric variants are ongoing in our laboratory. Finally, considering recent developments in skeletal editing strategies with pyridines and pyridine oxides, we anticipate that these products can be further derivatized for applications in medicinal chemistry.

**Scheme 3:**
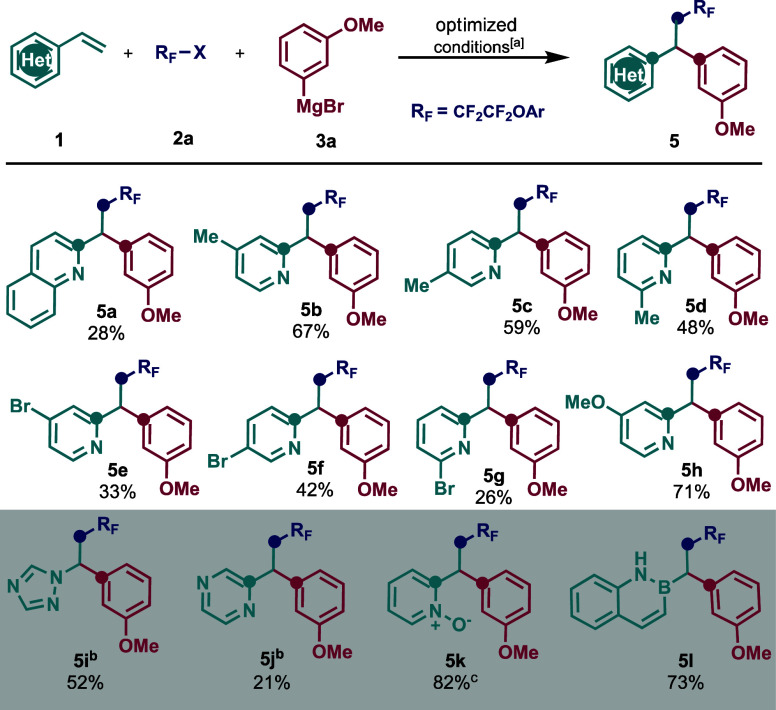
Scope of Vinyl Azaarenes Reaction conditions: **1a** (0.2 mmol, 1.0 equiv), **2a** (0.3 mmol, 1.5 equiv), Fe catalyst (20 mol %), ligand dcpe (40 mol %), THF (*c* 1.0 M), 0 °C, slow addition of **3a** (0.3 mmol, 1.5 equiv) in 1 h, nitrogen atmosphere. Reaction conditions: **1a** (0.2 mmol, 1.0 equiv), **2a** (0.4 mmol, 2.0 equiv). Yield for postfunctionalization step only.

To gain some insight into the mechanism, we used 2-(1-cyclopropylethenyl)pyridine (**1-A**) as a radical probe (Scheme 4). Using the model radical precursor **2a** and Grignard reagent **3a**, we were able to isolate the desired ring-opened product **6a** in moderate yield (Scheme 4a). Further, upon the use of disubstituted **1-B** we observed products **7a** and **7b** through HRMS. A reaction was carried out with TEMPO as a radical scavenger, and the results showed evidence for heterobenzyl radical formation (Figure S5). Taken together, these results support the intermediacy of heterobenzyl radicals. Next we turned to dispersion-corrected density functional theory (DFT) calculations to gain insight into the mechanism of the C–C bond formation (see the Supporting Information). We considered the possibility of a fluoroalkyl bromide undergoing halogen abstraction by an iron species to generate a MeCF_2_CF_2_^•^ radical based on previous mechanistic studies from our group and others.^14^ In Scheme 4c, radical addition to 2-vinylpyridine **1a** (via **TS1**) occurs with a low energy barrier (8.9 kcal/mol), leading to radical **Int**^•^. Subsequently, **Int**^•^ rapidly and reversibly adds to the monoaryl Fe(II) species ^**5**^**C** through the selective quartet spin state ^**4**^**TS2** (barrier ≈ 3.7 kcal/mol) to form the distorted square-pyramidal Fe(III) intermediate ^**4**^**D** in the same spin state. Finally, the subsequent and irreversible reductive elimination via selective quartet spin state ^**4**^**TS3** (barrier ≈ 12.4 kcal/mol) results in the formation of the desired product and Fe(I) species ^**4**^**A**, which can then initiate the catalytic process again. A proposed catalytic cycle can be found in Figure S13. We also explored an alternative pathway in which the C–C bond is formed through an outer-sphere mechanism via ^**4**^**TS4**. Here we found that the pyridine nitrogen can interact with the metal center to facilitate this process.^15^ However, this pathway is ruled out based on a much higher energy barrier (∼20.1 kcal/mol) compared with the inner-sphere stepwise C–C bond formation.

**Scheme 4:**
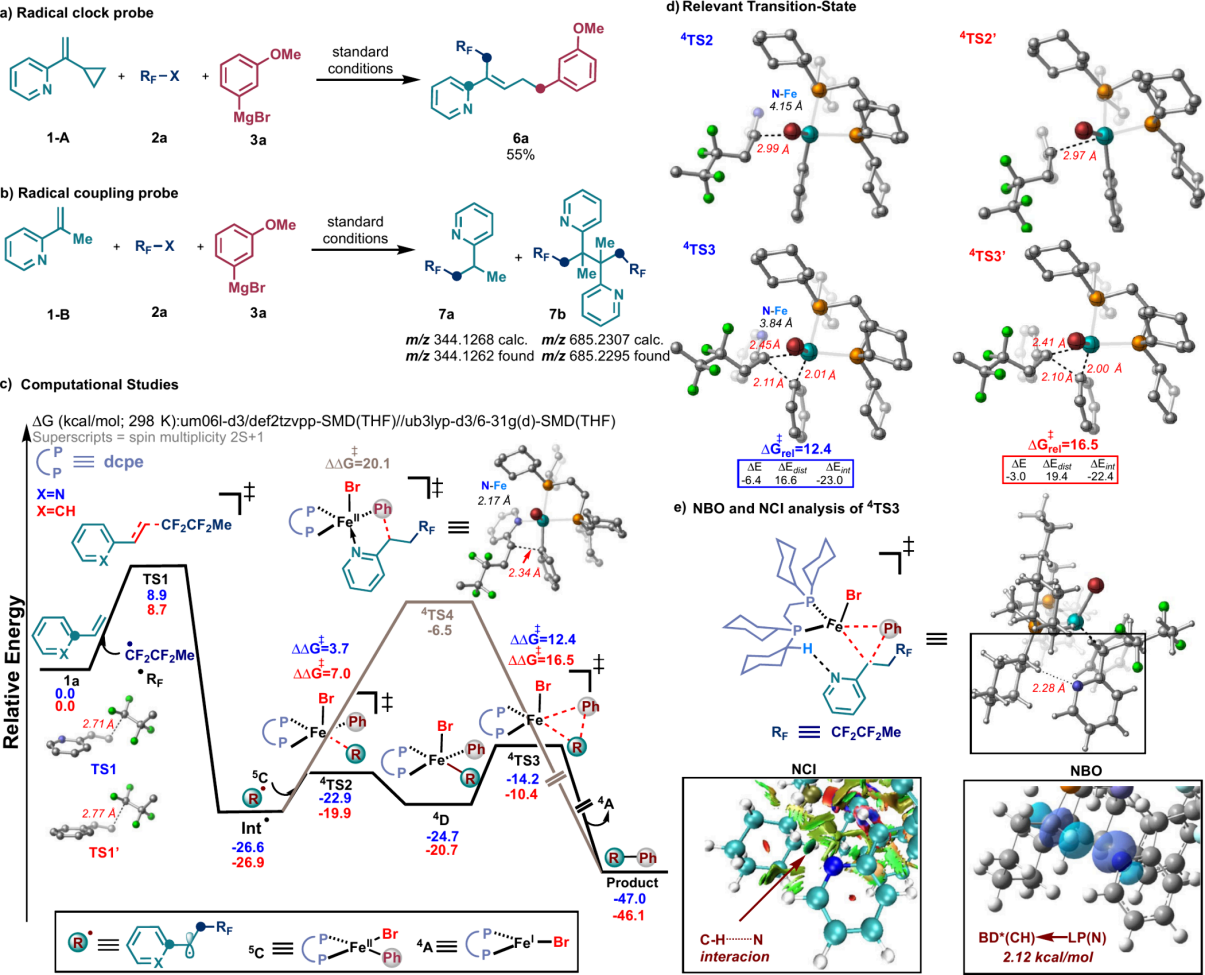
Mechanistic Studies

Next, we redirected our efforts toward using calculations to gain a deeper understanding of nitrogen’s crucial role in ensuring the success of this transformation. To accomplish this, we conducted a mechanism calculation using styrene. In Scheme 4c, the thermodynamic drive for the first addition is essentially the same. However, a significant difference was observed in the rate-determining reductive elimination step. Specifically, ^**4**^**TS3** for the pyridine system shows a lower energy barrier by approximately ∼4.0 kcal/mol compared to ^**4**^**TS3′** for the phenyl system (12.4 vs 16.5 kcal/mol; Scheme 4d). Distortion/interaction analysis revealed that the lower barrier associated with ^**4**^**TS3** is due to the lower distortion energy (Δ*E*_dis_) between the two fragments compared to that of ^**4**^**TS3′** (Scheme 4d). In addition, the natural bond orbital (NBO) analysis showed that ^**4**^**TS3** benefits from favorable donor–acceptor interaction energy from the lone pair (LP) in the pyridine moiety with the antibonding (BD*) orbital of the C–H bond on the ligand, supported by the non-covalent interaction (NCI) (Scheme 4e). The efficiency of achieving this weak interaction can be seen in the poor yield obtained with ligands in the absence of alkyl C–H (e.g., dppe).

In conclusion, we have developed a bisphosphine–iron catalytic system that enables the rapid and modular synthesis of unsymmetrical 1,1-bis(hetero)arylalkanes using vinyl azaarenes as a linchpin. The reaction tolerates a variety of vinyl azaarene linchpins, radical precursors, and (hetero)aryl Grignard and turbo Grignard reagents. This method provides access to the selective dicarbofunctionalization of heterobenzyl radicals, forming two new C–C bonds under mild conditions with short reaction times. The azaarene handle provides access to medicinally and synthetically relevant compounds, such as those containing pyridine *N*-oxides, pyrazines, triazoles, carbazoles, indazoles, and 1,2-azaborines. Experimental and computational studies were used to gain insights into the heterobenzyl radical intermediate and the C–C bond-forming step. We are currently using a mechanistic-driven approach to develop asymmetric variants of these transformations, and this work will be reported in due course.

## Data Availability

The data underlying this study are available in the published article and its Supporting Information.

## Supporting Information Available

Experimental procedures, product characterizations, computational studies, and copies of the ^1^H and ^13^C NMR spectra (PDF)
