# Supplementary material for: Fe-Catalyzed Fluoroalkyl(hetero)arylation of Vinyl Azaarenes: Rapid and Modular Synthesis of Unsymmetrical 1,1-Bis(hetero)arylalkanes
Source: Org Lett. 2024 Aug 14;26(33):7015–20. doi: 10.1021/acs.orglett.4c02515 (PMC11348425; doi:10.1021/acs.orglett.4c02515)
Supplement: Supplementary file 1 — ol4c02515_si_001.pdf [file ol4c02515_si_001.pdf]

Supporting Information

Fe-catalyzed Fluoroalkyl(hetero)arylation of Vinyl Azaarenes: Rapid and Modular Synthesis of Unsymmetrical 1,1-Bis(hetero)arylalkanes

Macayla Guerrero[‡], Ángel Rentería-Gómez[‡], Deborshee Das, Osvaldo Gutierrez*
Department of Chemistry, Texas A&M University, College Station, Texas 77843, United States
og.labs@tamu.edu

Table of Contents

1. **General Considerations** **S2**
2. **Procedure for Synthesis of 2-Vinylquinoline.** **S3**
3. **Procedure for Synthesis of 2-(1-cyclopropylethenyl)pyridine.** **S3**
4. **Procedure for Synthesis of Radical Precursors.** **S3 – S4**
5. **General Procedure for Synthesis of Grignard Reagents.** **S4 – S5**
6. **General Procedure for Synthesis of 2-Vinyl-1,2-dihydrobenzo[e][1,2]azaborinine S5 – S6**
7. **General Procedure for Iron-Catalyzed Multicomponent Cross-Coupling Reaction. S7**
8. **Screening of Reaction Conditions** **S8 – S11**
9. **Current Limitations** **S12 – S13**
10. **Product Characterization Data.** **S14 – S46**
11. **General Procedure for Synthesis of Pyridine N-Oxide.** **S46 – S47**
12. **Radical Trapping Experimental and Radical Proof** **S48 – S49**
13. **Crystal Data and Experimental for Product 9b** **S49 – S57**
14. **Spectral Data.** **S58 – S210**
15. **Computational Methods, Results, and Coordinates** **S211 – S235**
16. **References** **S236 – S237**

1. General Considerations.

Unless otherwise indicated, all reactions were carried out under a nitrogen atmosphere in oven- (110 °C) or flame-dried glassware. When necessary, solvents and reagents were dried prior to use. Tetrahydrofuran (THF) was dried by passage through activated alumina in Inert's PureSolv MB-SPS solvent purification system. All solvents were obtained from VWR, Sigma-Aldrich, or Fisher. Organometallic reagents were purchased from Sigma-Aldrich and Synthonix. Organic reagents and starting materials were purchased from Sigma-Aldrich, Ambeed, A2B Chem, Apollo Scientific, Enamine, AA Blocks, Combi-Blocks, Matrix Scientific, TCI Chemicals, Accela ChemBio, 1Click Chemistry, AOBChem, Fischer Scientific, and Oakwood Chemical. Silicycle 250 µm silica-gel F-254 plates were used to perform analytical thin layer chromatography (TLC). Column chromatography was performed with Silica gel (230-400 mesh) and Biotage® Selekt Flash Systems silica gel chromatography was performed with prepacked silica-gel cartridges (Sfar; Biotage). NMR (^{1}H, ^{13}C and ^{19}F) spectra were recorded on Acsend™ 400 (Bruker) NMR spectrometer. Chemical shifts (δ) are reported in parts per million (ppm) relative to the internal residual solvent resonance peak δ 7.26 ($CDCl_3$) and δ 0.00 (TMS) for all ^{1}H and δ 77.16 ($CDCl_3$) and δ 0.00 (TMS) for all ^{13}C. Other data are designated as follows: multiplicity (s = singlet, d = doublet, t = triplet, q = quartet, qu = quintet, sept = septet, oct = octet, m = multiplet, dd= doublet of doublets, dt = doublet of triplets, td = triplet of doublets, dq = doublet of quartets, qd = quartet of doublets, tt = triplet of triplets, tdd = triplet of doublet of doublets, bs = broad singlet), coupling constants (*J*) are reported in Hertz (Hz), and number of protons. High Resolution Mass (HRMS) spectra using Electrospray ionization (ESI) mode and Atmospheric chemical ionization (APCI) mode were acquired on Thermo Scientific Q Exactive Focus mass spectrometer with a Fourier Transform Ion Cyclotron Resonance Mass Analyzer. Thermo Scientific Nicolet Summit FTIR spectrometer was employed for recording IR spectra and are reported in wavenumbers (cm^{-1}).

2. Procedure for Synthesis of 2-Vinylquinoline.

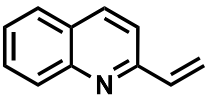

This compound was made following a literature procedure.[1] A solution of 2-chloroquinoline (3.0 mmol, 1.0 equiv.), potassium vinyltrifluoroborate (3.6 mmol, 1.2 equiv.), $PdCl_2(dppf)\cdot CH_2Cl_2$ (2 mol%), and Et_3N (1.0 equiv.) in *i*-PrOH (10 mL/mmol) was heated to reflux for 16 h using an oil bath. The reaction was cooled to room temperature, concentrated *in vacuo*, the resulting solution was washed with brine (25 mL), and CH_2Cl_2 (3x 25 mL). The organic extracts were washed with brine (25 mL) and then dried with $MgSO_4$. The dried product was filtered and then concentrated *in vacuo*. The resulting solution was purified on silica gel column chromatography with hexane/EtOAc (9:1) giving the product (216.9 mg, 46% yield) as a yellow liquid.

3. Procedure for Synthesis of 2-(1-cyclopropylethenyl)pyridine.

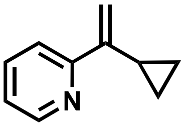

This compound was made using a literature procedure.[2] To a flask charged with methyltriphenylphosphonium bromide (2.72 mmol, 2.0 equiv) and 8.2 mL of anhydrous THF at 0 °C was added *n*-butyllithium (1.7 mL, 1.6 M in hexanes, 2.0 equiv.). The reaction was allowed to warm to room temperature spontaneously and then stirred for 1 h. Cyclopropyl-2-pyridinylmethanone (1.36 mmol, 1.0 equiv.) in anhydrous THF (2.3 mL) was added dropwise over 20 mins, and then the reaction was stirred for 12 h at 40 °C using an aluminum heating block. After being quenched with brine, the mixture was extracted with petroleum ether (3x). The combined organic layers were washed with water (3x) and then dried ($MgSO_4$) and then concentrated *in vacuo*. The residue was purified with silica gel column chromatography with hexane:EtOAc (8:2) to afford the product as a colorless oil (99.9 mg, 50% yield).

4. General Procedure for Synthesis of Radical Precursors.

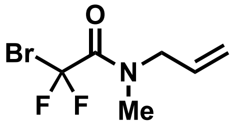

***N*-allyl-2-bromo-2,2-difluoro-*N*-methylacetamide**

This compound was made following a literature procedure.[3] Ethyl bromodifluoroacetate (3.04 mmol, 1.0 equiv.) was added to a flask and bubbled under Argon with a stir bar. Then *N*-methylallylamine (3.65 mmol, 1.2 equiv.) was added to the flask dropwise. The reaction mixture was stirred overnight at rt under Argon atmosphere. The reaction mixture was diluted with diethyl ether (10 mL) and washed with 2 M HCl (2x 10 mL), $NaHCO_3$ (2x 10 mL), and brine (10 mL). The resulting organic extract was concentrated *in vacuo* and then purified using silica

gel column chromatrography hexane:EtOAc (6:4) to afford the product (398.5 mg, 57% yield) as a colorless oil. The characterized data of the compound agreed with previous reports.

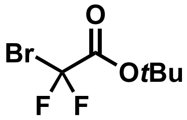

***Tert*-Butyl 2-bromo,2,2-difluoroacete**

This compound was made using a modified literature procedure.[3] Bromodifluoroacetic acid (2.65 g, 15.00 mmol, 1.0 equiv.) was dissolved in dry DCM [0.45 M] in a dry flask under inert atmosphere. Oxalyl chloride (2.094 g, 16.5 mmol, 1.1 equiv) was then added to the flask and then followed by 2 drops of DMF. The solution was then stirred at rt for 2 h or until no more gas evolved. *tert*-Butyl alcohol (2.224 g, 30.0 mmol, 2.0 equiv.) and triethylamine (1.670 g, 16.5 mmol, 1.1 equiv.) were added dropwise as a solution in DCM (75.0 mL) at 0 °C over 20 min. The reaction mixture was warmed to rt and stirred for 2 h. The reaction mixture was diluted with water and extracted 3x with DCM. The combined organic phases was washed with sat. bicarbonate and dried with $MgSO_4$. The resulting extracted was filter and concentrated *in vacuo*. The resulting mixture was isolated using low-pressure distillation. The product comes out starting at 9 mbar at 55 °C, and ends at 5 mbar at 48 °C. The product (2.359 g, 68% yield) was isolated as a colorless oil.

5. General Procedure for Synthesis of Grignard Reagents.[4]

General procedure a: In an Argon filled glovebox, a flame-dried two-neck 25 mL flask with a stir bar was transferred and the vial was charged with Mg powder (243 mg, 10 mmol, 2.0 equiv.) and LiCl (254.3 mg, 6.0 mmol, 1.2 equiv.). The flask was sealed with a Teflon cap and brought out of the glovebox. The flask was heated under vacuum for 2 min with stirring. Anhydrous THF (5 mL) and DIBALH (0.25 mL, 1.0 M solution in hexane, 0.25 mmol) were added to the cooled flask and the mixture was stirred vigorously. After 5 min at room temperature, the heteroaryl halide (5.0 mmol, 1.0 equiv.) was added to the reaction mixture at the specified temperature using either an oil bath or an aluminum heating block. The resulting solution was stirred for the specified time. The concentration of the resulting heteroaryl Grignard reagent was calculated via iodometric titration, with solution colors ranging from green to black.

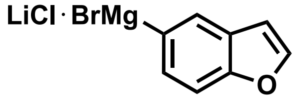

5-Benzofuranmagnesium bromide lithium chloride complex (3a): Compound **3a** was synthesized following the general procedure 1, using 5-benzofuran (985.2 mg, 0.5 mmol), Mg powder (10.0 mmol, 2.0 equiv), LiCl (6.0 mmol, 1.2 equiv.), THF (5.0 mL), and DIBALH (0.25 mL, 0.05 equiv.) stirred for 2 h at 60 °C. The product **3a** was obtained as a green liquid with the concentration [0.76 M] determined via iodometric titration.

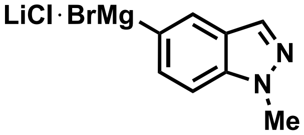

5-(1-methyl-1H-indazole)magnesium bromide lithium chloride complex (3b): Compound **3b** was synthesized following the general procedure 1, using 5-Bromo-1-methyl-1H-indazole (1.055 g, 5.0 mmol), Mg powder (7.5 mmol, 1.5 equiv), LiCl (5.5 mmol, 1.1 equiv.), THF (5.0 mL), and DIBALH (0.25 mL, 0.05 equiv.) stirred for 2 h at rt. The product **3b** was obtained as a green liquid with the concentration [0.70 M] determined via iodometric titration.

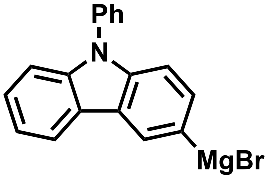

3-(9-phenyl-9H-carbazole)magnesium bromide (3c): Compound **3c** was synthesized following a modified general procedure 1, using 3-Bromo-9-phenyl-9H-carbazole (1.611 g, 5.0 mmol), Mg powder (6.0 mmol, 1.2 equiv), I_2 (single crystal), and THF (4.2 mL). The product **3c** was obtained as a black liquid with the concentration [1.10 M] determined via iodometric titration.

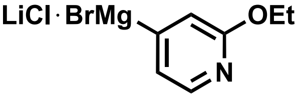

4-(2-ethoxypyridine)magnesium bromide lithium chloride complex (3d): Compound **3d** was synthesized following the general procedure 1, using 4-Bromo-2-ethoxypyridine (1.010 g, 5.0 mmol), Mg powder (10.0 mmol, 2.0 equiv), LiCl (6.0 mmol, 1.2 equiv.), THF (5.0 mL), and DIBALH (0.25 mL, 0.05 equiv.) stirred for 2 h at 60 °C. The product **3d** was obtained as a dark purple liquid with the concentration [0.86 M] determined via iodometric titration.

6. General Procedure for Synthesis of 2-Vinyl-1,2-dihydrobenzo[e][1,2]azaborinine (BN2VN)[5]

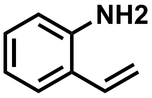

An oven-dried 100 mL round bottom flask was transferred to an Argon filled glovebox. To the flask, 2-aminophenethyl alcohol (15 g, 109 mmol, 1.0 equiv.) and potassium hydroxide pellets (6.1 g, 109 mmol, 1.0 equiv.) were added. Using a short path distillation head and a tared receiving flask, the 100 mL RB was purged Argon and placed under vacuum for 3 times, then left on vacuum. The flask was heated to 180°C with continuous heating and applied vacuum using an oil bath. (4.7 mbar) The clear product (4 g, 30% yield) distilled over at 80 – 90°C.

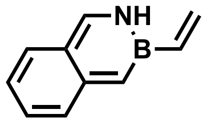

In an oven-dried 3-neck 250 mL round bottom flask, 2-aminostyrene (1 g, 8.4 mmol, 1.0 equiv.) was charged inside an Argon filled glovebox. Then the RB was removed from the glovebox and kept under vacuum and purged with Argon 3 times. Then toluene (37.5 mL) and cyclopentyl methyl ether (37.5 mL) was added into the RB. Next, potassium vinyltrifluoroborate (1.23 g, 9.24 mmol, 1.1 equiv.), silica (1.57 g, 9.24 mmol, 1.1 equiv.), and EtN_3 (1.27 g, 12.6 mmol, 1.5 equiv.) was added into the mixture under Argon. The solution was then heated at 60°C for 18 h using an oil bath under Argon with a condenser with continuous water flow and vigorous stirring. The reaction was then cooled to room temperature and then 50 mL of hexane was added to the mixture. Then the mixture was filtered through celite using a Buchner funnel and washed with hexane. The resulting solution was then evaporated and the crude mixture was isolated using column chromatography (9:1 Hexane:EtOAc), to give the product as a white solid (1.7 g).

7. General Procedure for Iron-Catalyzed Multicomponent Cross-Coupling Reaction.

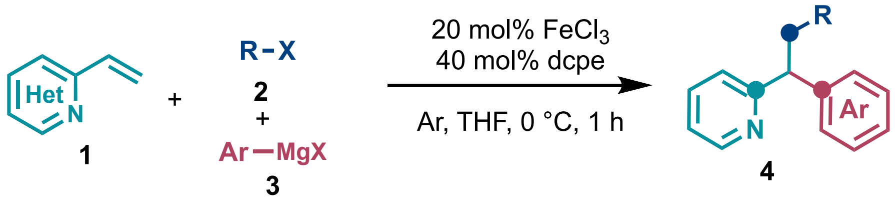

General Procedure B - Standard-scale: A flame-dried 5 mL microwave vial with a stir bar was transferred into an argon-filled glovebox and the vial was charged with $FeCl_3$ (6.5 mg, 0.04 mmol), 1,2-bis(dicyclohexylphosphanyl)ethane **dcpe** (33.8 mg, 0.08 mmol), alkyl halide **2** (0.3 mmol, 1.5 equiv) and azaarene **1** (0.2 mmol, 1.0 equiv) (using oven-dried glass pipette to transfer alkyl halide to the vial). The vial was sealed with a Teflon cap and brought out of the glovebox without solvent. THF (0.2 mL) was then added into the reaction mixture. The resulting green solution was stirred at room temperature for 5 min. The reaction mixture was then cooled to 0 °C using an ice bath and a ArMgBr **3** (0.5–1.0 M solution in THF, 1.5 equiv) was added slowly for 1 h using a syringe pump. Over which time the heterogeneous solution turned from red to yellow, brown, grass green or orange color (depending on ArMgBr and substrate). The resulting reaction mixture was then stirred at 0 °C for an additional 10 min. After completion, the reaction mixture was quenched with a 1.0 M aqueous solution of hydrochloric acid (0.1 mL) and water (0.5 mL) and then extracted with ethyl acetate (3 x 2 mL). The combined organic solution was filtered through a short pad of silica and evaporation of solvent gave a residue that was purified on silica gel column chromatography with hexane/ethyl acetate to obtain product.

8. Screening of Reaction Conditions

Table S1. Screening of Fe salt.

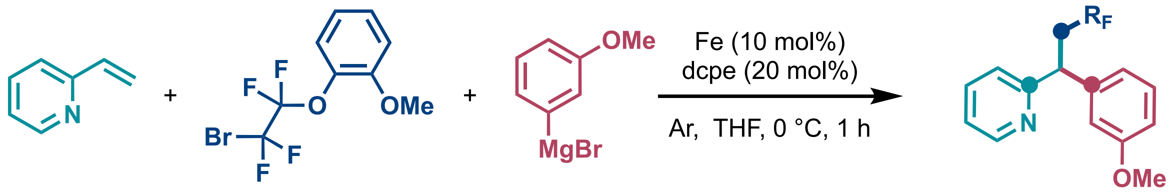

1	2	3	4
1.0 equiv.	2.0 equiv.	2.0 equiv.	

Entry	Fe	Isolated Yield
1	$FeCl_3$	57%
2	$Fe(OTf)_3$	7%
3	$Fe(acac)_3$	29%
4	$FeBr_3$	32%
5	$FeCl_2$	54%
6	$Fe(dibm)_3$	9%

Table S2. Screening of ligands.

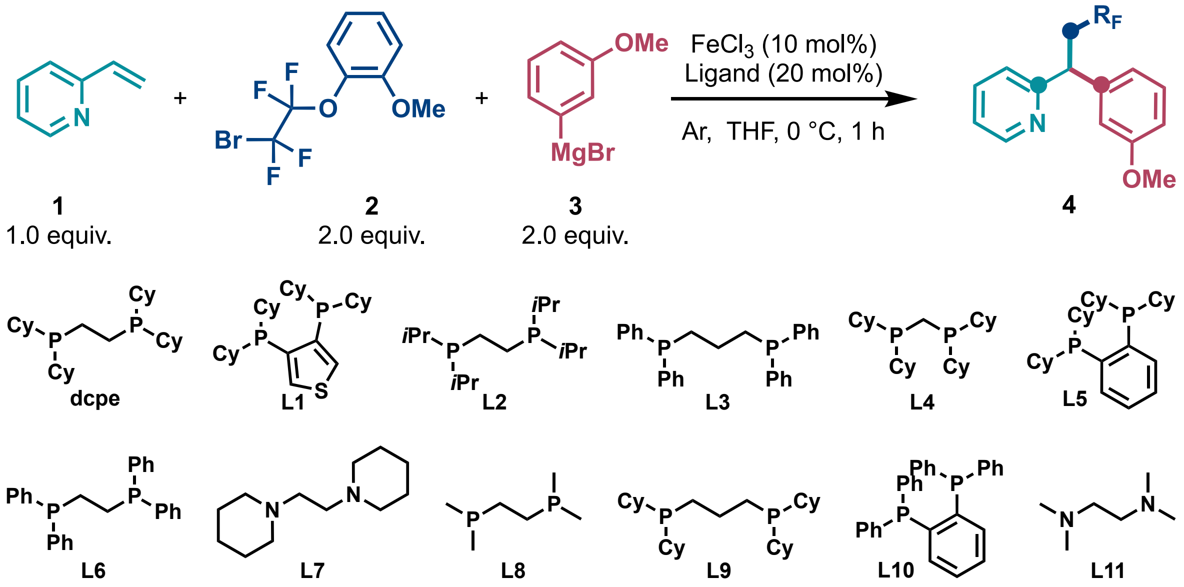

Entry	Ligand	Isolated Yield
**1**	dcpe	57%
**2**	L1	11%
**3**	L2	23%
**4**	L3	26%
**5**	L4	27%
**6**	L5	17%
**7**	L6	24%
**8**	L7	8%
**9**	L8	0%
**10**	L9	25%
**11**	L10	13%
**12**	L11	10%

Table S3. Screening of Fe salt and ligand loading.

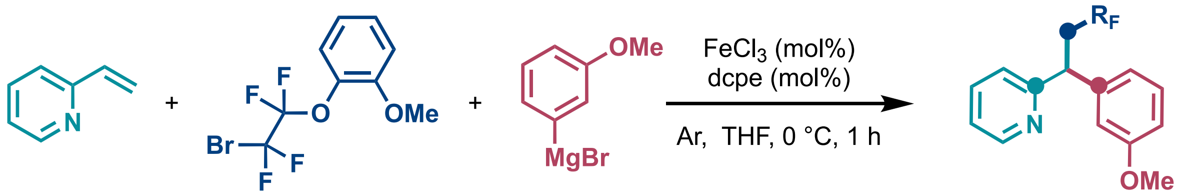

Entry	$FeCl_3$ (mol %)	Ligand (mol %)	Isolated Yield
1[a]	20 mol%	40 mol%	72%
2[a]	10 mol%	20 mol%	57%
3[b]	5 mol%	10 mol%	60%
4[b]	40 mol%	20 mol%	29%

Table S3. a) Equivalence of 1/2/3: 1.0/2.0/2.0 b) Equivalence of 1/2/3: 1.0/2.0/1.5

Table S5. Screening of solvent and reaction time.

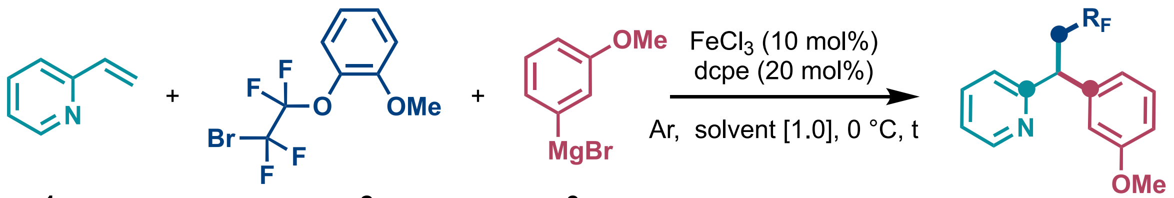

Entry	Time	Solvent	Isolated Yield
1	1 h	THF	57%
2[a]	1 h	THF	38%
3	30 min	THF	42%
4	2 h	THF	43%
5[b,c]	70 min	THF	74%
6[b]	1 h	2-MeTHF	73%

Table S5: a) $FeCl_3$, dcpe, and 0.1 mL solvent stirred for 30 min, then 1/2/3 added and slow addition of Grignard for 1 h. b) $FeCl_3$ (20 mol%), dcpe (40 mol%) c) 1.0 equiv. of Grignard reagent added in 10 min and then 1.0 equiv. Grignard reagent added for 1 h.

Table S6. Screening of ratios of alkene, radical precursor, and Grignard reagent.

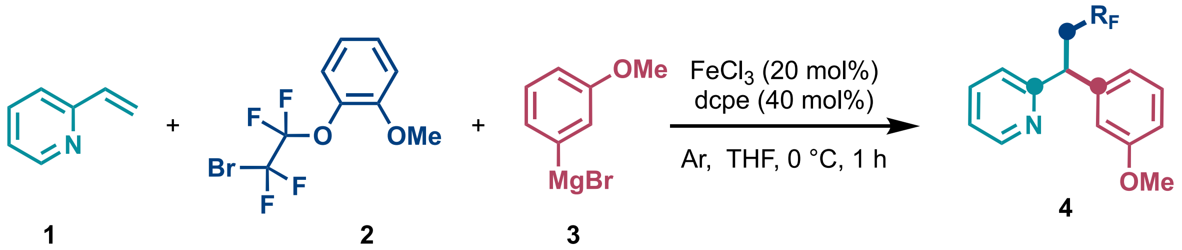

Entry	1 (equiv.)	2 (equiv.)	3 (equiv.)	Isolated Yield
**1**	1.0	2.0	2.0	72%
**2**[a]	1.0	2.0	2.0	57%
**3**	1.0	2.0	1.5	72%
**4**	1.0	2.0	1.0	62%
**5**	1.0	1.5	1.5	77%

Table S6: a) $FeCl_3$ (10 mol%) and dcpe (20 mol%)

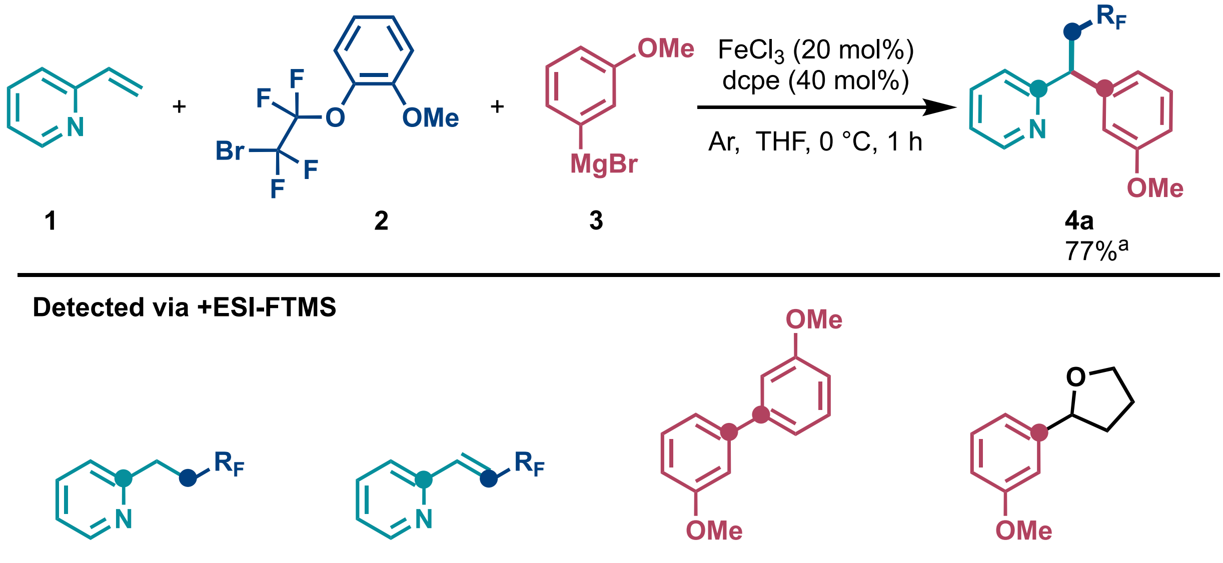

**B**	**C**	**D**	**E**
m/z 330.1112 calc.	***m/z*** 328.0955 calc.	***m/z*** 215.1067 calc.	***m/z*** 179.1067 calc.
m/z 330.1104 found	***m/z*** 328.0948 found	***m/z*** 215.1064 found	***m/z*** 179.1063 found

Figure S1. Side Products from General Procedure B

Fig. S1: a) ^{1}H NMR yield using dibromomethane

Following general procedure B, using the model substrates, the desired product **4a** was detected along with the side products, **B**, **C**, **D**, and **E** using +ESI-FTMS.

9. Current Limitations.

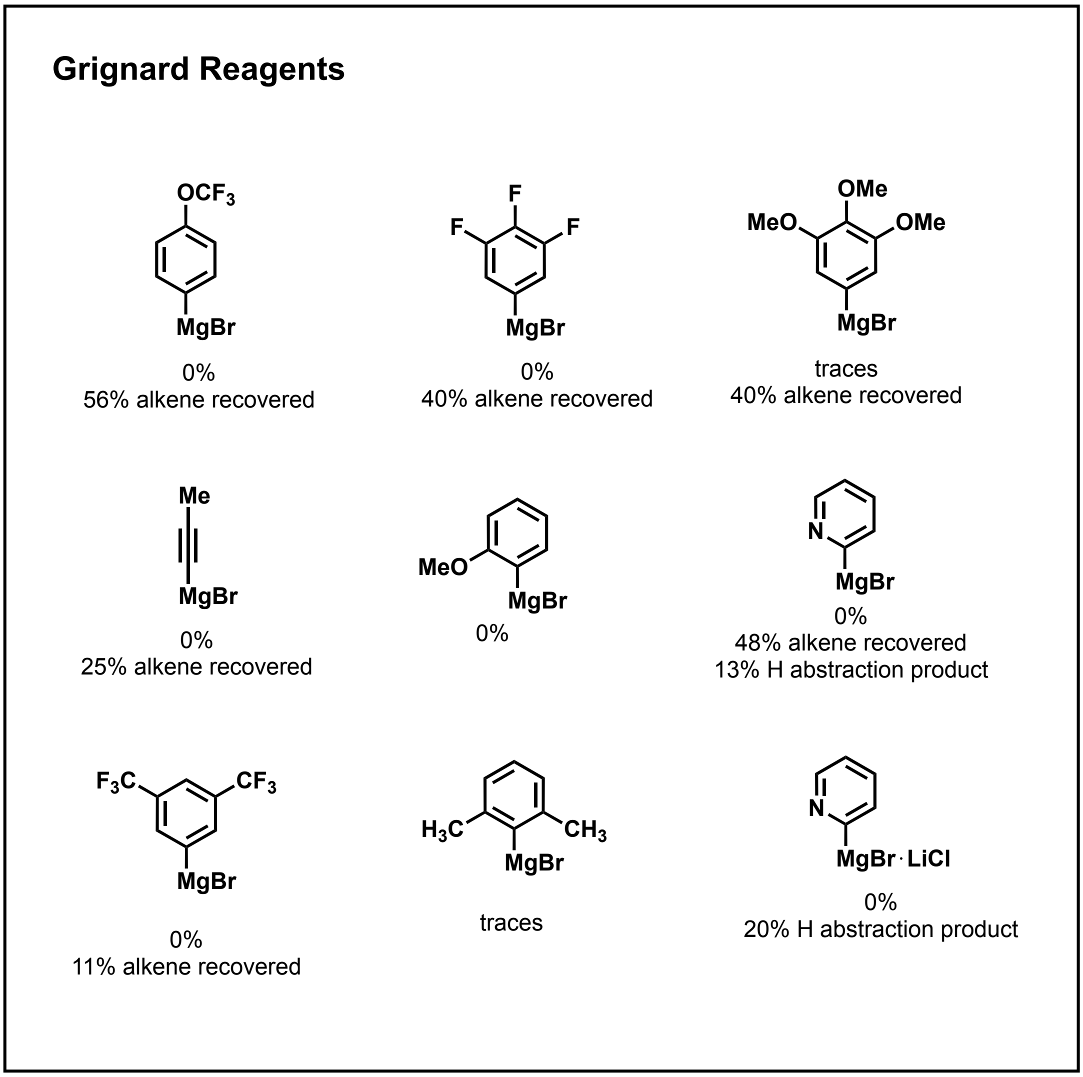

Figure S2. Limitations of Grignard Reagents

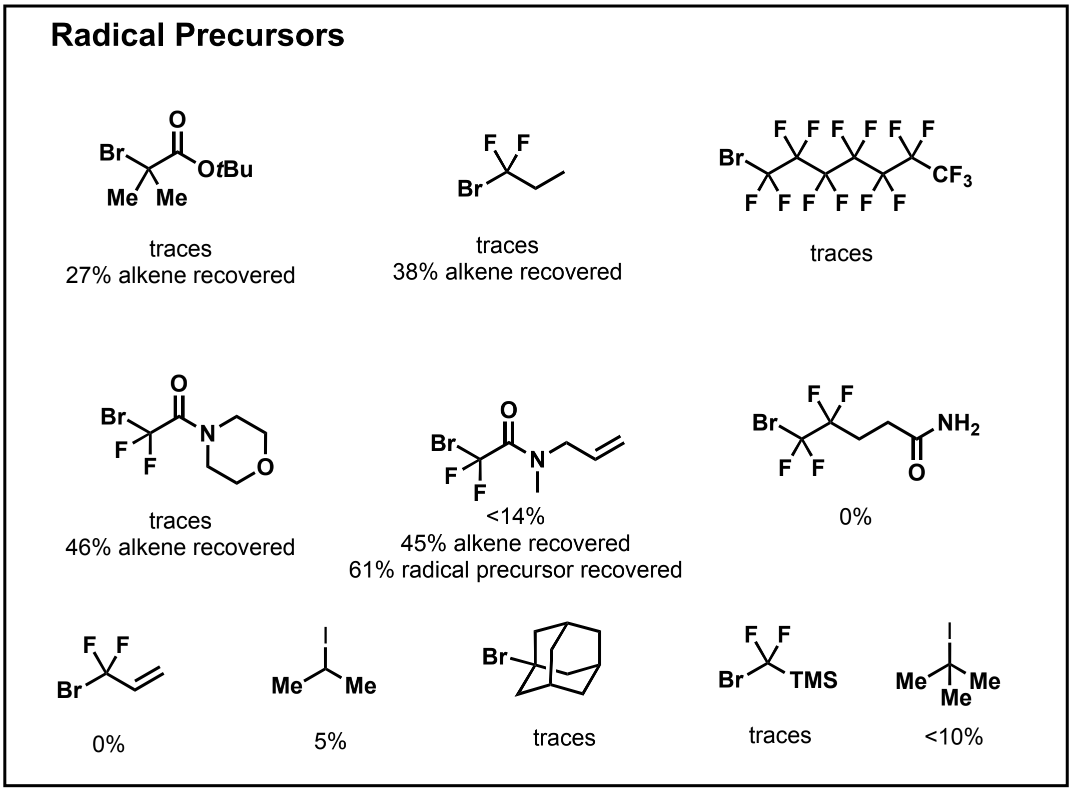

Figure S3. Limitations of Radical Precursors

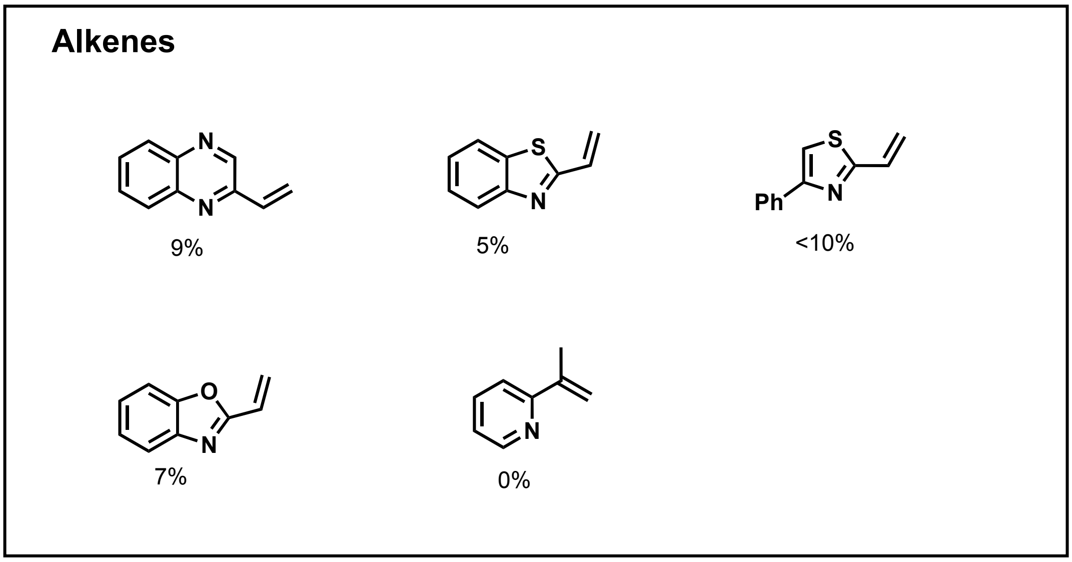

Figure S4. Limitations of Alkenes

10. Product Characterization Data.

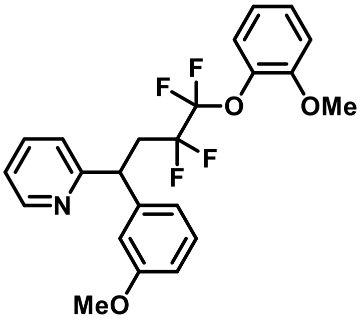

2-(3,3,4,4-tetrafluoro-4-(2-methoxyphenoxy)-1-(3-methoxyphenyl)butyl)pyridine (4a): Compound **4a** was synthesized following the general procedure 1 (standard-scale), using 2-(2-bromo-1,1,2,2-tetrafluoroethoxy) anisole (90.91 mg, 0.3 mmol), 2-Vinylpyridine (21.7 mg, 0.2 mmol) and 3-methoxyphenylmagnesium bromide (0.3 mL, 1.0 M solution in THF, 0.3 mmol). The product **4a** was obtained as a colorless liquid (67.1 mg, 77% yield) after purification by column chromatography on silica gel with hexane/EtOAc (8:2).

2-(3,3,4,4-tetrafluoro-4-(2-methoxyphenoxy)-1-(3-methoxyphenyl)butyl)pyridine (4a) [4.0 mmol scale: Compound **4a** was synthesized following the general procedure 1 (standard-scale), using 2-(2-bromo-1,1,2,2-tetrafluoroethoxy) anisole (1.84 g, 6.0 mmol), 2-Vinylpyridine (421 mg mg, 4.0 mmol) and 3-methoxyphenylmagnesium bromide (6.7 mL, 0.9 M solution in THF, 6.0 mmol). The product **4a** was obtained as a colorless liquid (860.8 mg, 77% yield) after purification by column chromatography on silica gel with hexane/EtOAc (8:2).

^{1}H NMR (400 MHz, CDCl$_3$) δ = 8.65 (d, J = 4.9 Hz, 1H), 7.61 (td, J = 7.7, 1.9 Hz, 1H), 7.30 – 7.23 (m, 4H), 7.15 (ddd, J = 7.5, 4.9, 1.2 Hz, 1H), 7.08 – 6.99 (m, 3H), 6.98 – 6.93 (m, 1H), 6.80 (ddd, J = 8.2, 2.6, 1.0 Hz, 1H), 4.66 (dd, J = 7.9, 5.7 Hz, 1H), 3.85 (s, 3H), 3.82 (s, 3H), 3.68 – 3.53 (m, 1H), 3.08 – 2.91 (m, 1H).

^{13}C NMR (100 MHz, CDCl$_3$) δ = 161.9, 159.8, 152.6, 149.4, 144.9, 138.0, 136.6, 129.7, 127.5, 124.0, 123.3, 121.7, 120.7, 120.3, 120.6 - 114.8 (m), 114.0, 112.9, 112.1, 56.1, 55.3, 46.1, 35.9 (t, J = 20.6 Hz).

^{19}F NMR (376 MHz, CDCl$_3$) δ = -88.35 to -88.51 (m, 2F), -115.76 (tt, J = 15.3, 4.5 Hz, 2F).

IR (film): 623, 653, 698, 723, 745, 772, 808, 854, 940, 991, 1042, 1077, 1104, 1169, 1187, 1220, 1260, 1282, 1303, 1434, 1465, 1502, 1571, 1589, 2839, 2944 cm^{-1}

HRMS (+ESI-FTMS) calcd for $C_{23}H_{21}F_4NO_3$ $[M+H]^+$ m/z = 436.1530; found: 436.1520.

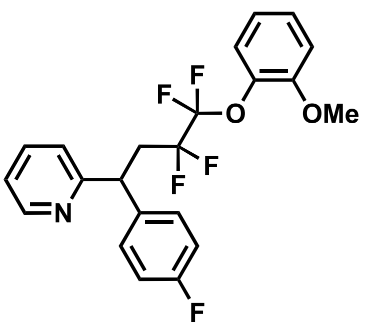

2-(3,3,4,4-tetrafluoro-1-(4-fluorophenyl)-4-(2-methoxyphenoxy)butyl)pyridine (4b): Compound **4b** was synthesized following the general procedure 1 (standard-scale), using 2-(2-bromo-1,1,2,2-tetrafluoroethoxy) anisole (90.3 mg, 0.3 mmol), 2-Vinylpyridine (21.7 mg, 0.2 mmol) and 4-fluorophenylmagnesium bromide (0.3 mL, 1.0 M solution in THF, 0.3 mmol). The product **4b** was obtained as a pale yellow liquid (31.2 mg, 36% yield) after purification by column chromatography on silica gel with hexane/EtOAc (8:2).

^{1}H NMR (400 MHz, $CDCl_3$) δ = 8.60 (d, J = 2.6 Hz, 1H), 7.58 (td, J = 7.7, 1.9 Hz, 1H), 7.42 – 7.36 (m, 2H), 7.24 – 7.19 (m, 3H), 7.12 (dd, J = 7.0, 4.2 Hz, 1H), 7.01 – 6.89 (m, 4H), 4.62 (t, J = 6.9 Hz, 1H), 3.81 (s, 3H), 3.57 – 3.42 (m, 1H), 3.02 – 2.86 (m, 1H).

^{13}C NMR (100 MHz, $CDCl_3$) δ = 161.9, 161.8 (d, J = 243.6 Hz), 152.6, 149.5, 138.9 (d, J = 3.2 Hz), 138.0, 136.8, 129.5 (d, J = 7.8 Hz), 127.6, 124.0, 123.2, 121.9, 120.7, 121.6 – 114.7 (m),115.5 (d, J = 21.3 Hz) , 112.9, 56.1, 45.4, 36.2 (t, J = 20.5Hz).

^{19}F NMR (376 MHz, $CDCl_3$) δ = -88.48 (s, 2F), -115.52 to -115.86 (m, F), -116.22 to -116.35 (m, 1F).

IR (film): 457, 523, 552, 569, 610, 628, 718, 746, 792, 810, 830, 927, 992, 1025, 1044, 1078, 1105, 1159, 1170, 1187, 1221, 1261, 1282, 1305, 1436, 1465, 1502, 1571, 1590, 1604, 2853, 2923, 2957 cm^{-1}

HRMS (+ESI-FTMS) calcd for $C_{22}H_{18}F_5NO_2$ $[M+H]^+$ m/z = 424.1330; found: 424.1329.

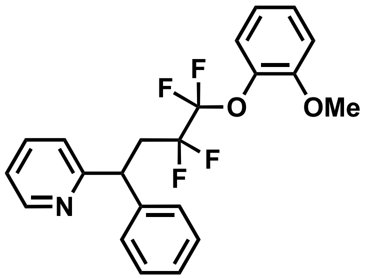

2-(3,3,4,4-tetrafluoro-4-(2-methoxyphenoxy)-1-phenylbutyl)pyridine (4c): Compound **4c** was synthesized following the general procedure 1 (standard-scale), using 2-(2-bromo-1,1,2,2-tetrafluoroethoxy) anisole (90.3 mg, 0.3 mmol), 2-Vinylpyridine (21.7 mg, 0.2 mmol) and phenylmagnesium bromide (0.3 mL, 1.0 M solution in THF, 0.3 mmol). The product **4c** was obtained as a pale yellow liquid (57.9 mg, 71% yield) after purification by column chromatography on silica gel with hexane/EtOAc (8:2).

^{1}H NMR (400 MHz, CDCl$_3$) δ = 8.65 (d, *J* = 4.4 Hz, 1H), 7.61 (td, *J* = 1.9 1H), 7.49-7.44 (m, 2H), 7.35 (t, *J* = 7.4 Hz, 2H), 7.26 (t, *J* = 7.2 Hz, 4H), 7.15 (dd, *J* = 7.8, 7.6 Hz, 1H), 7.01 – 6.95 (m, 2H), 4.68 (t, *J* = 5.8 Hz, 1H), 3.85 (s, 3H), 3.69 – 3.51 (m, 1H), 3.08 – 2.93 (m, 1H)
^{13}C NMR (100 MHz, CDCl$_3$) *δ* = 162.1, 152.6, 149.4, 143.3, 138.0, 136.7, 128.8, 128.0, 127.5, 126.9, 124.0, 123.3, 121.7, 120.7, 119.9 – 114.2 (m),112.9, 56.1, 46.2, 36.0 (t, *J* = 21.5 Hz).
^{19}F NMR (376 MHz, CDCl$_3$) δ = -88.46 (d, *J* = 4.2 Hz, 2F), -115.76 (tdt, *J* = 15.3, 7.6, 4.2 Hz, 2F).
IR (film): 568, 612, 698, 712, 745, 990, 1027, 1044, 1070, 1103, 1169, 1186, 1220, 1261, 1282, 1303, 1434, 1456, 1501, 1590, 2842, 2926, 3009, 3028, 3065 cm^{-1}
HRMS (+APCI-FTMS) calcd for $C_{22}H_{19}F_4NO_2$ $[M+H]^+$ m/z = 406.1425; found: 406.1421.

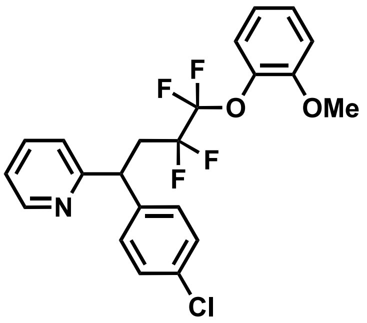

2-(1-(4-chlorophenyl)-3,3,4,4-tetrafluoro-4-(2-methoxyphenoxy)butyl)pyridineb (4d): Compound **4d** was synthesized following the general procedure 1 (standard-scale), using 2-(2-bromo-1,1,2,2-tetrafluoroethoxy) anisole (90.3 mg, 0.3 mmol), 2-Vinylpyridine (21.7 mg, 0.2 mmol) and 4-chlorophenylmagnesium bromide (0.3 mL, 1.0 M solution in diethyl ether, 0.3 mmol). The product **4d** was obtained as a colorless liquid (55.0 mg, 62% yield) after purification by column chromatography on silica gel with hexane/EtOAc (8:2).
^{1}H NMR (400 MHz, CDCl$_3$) δ = 8.59 (d, *J* = 3.0 Hz, 1H), 7.57 (td, *J* = 7.6, 1.8 Hz, 1H), 7.36 (d, *J* = 8.5 Hz, 2H), 7.26 (d, *J* = 8.6 Hz, 2H), 7.24 – 7.16 (m, 3H), 7.15 – 7.09 (m, 1H), 6.98 – 6.88 (m, 2H), 4.60 (t, *J* = 6.9 Hz, 1H), 3.80 (s, 3H), 3.57 – 3.40 (m, 1H), 3.03 – 2.85 (m, 1H).
^{13}C NMR (100 MHz, CDCl$_3$) *δ* = 161.6, 152.6, 149.5, 141.7, 137.9, 136.8, 132.8, 129.4, 128.9, 127.6, 124.0, 123.2, 121.9, 120.7, 119.7 – 114.3 (m), 112.9, 56.1, 45.6, 36.1 (t, *J* = 20.2 Hz)
^{19}F NMR (376 MHz, CDCl$_3$) δ = -88.46 (s, 2F), -115.62 (dddt, *J* = 70.1, 24.3, 12.5, 4.5 Hz, 2F).
IR (film): 568, 608, 714, 746, 765, 992, 1015, 1026, 1045, 1079, 1105, 1170, 1187, 1261, 1282, 1311, 1435, 1465, 1502, 2852, 2923 cm^{-1}
HRMS (+ESI-FTMS) calcd for $C_{22}H_{18}ClF_4NO_2$ $[M+H]^+$ m/z = 440.1035; found: 440.1028.

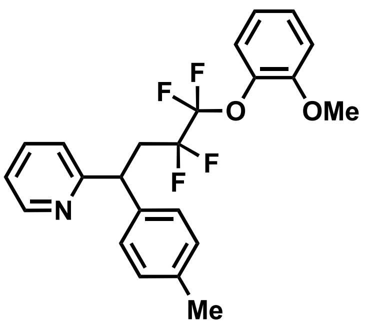

2-(3,3,4,4-tetrafluoro-4-(2-methoxyphenoxy)-1-(*p*-tolyl)butyl)pyridine (4e): Compound **4e** was synthesized following the general procedure 1 (standard-scale), using 2-(2-bromo-1,1,2,2-tetrafluoroethoxy) anisole (90.3 mg, 0.3 mmol), 2-Vinylpyridine (21.7 mg, 0.2 mmol) and p-tolylmagnesium bromide (0.3 mL, 1.0 M solution in THF, 0.3 mmol). The product **4e** was obtained as a colorless liquid (54.2 mg, 64% yield) after purification by column chromatography on silica gel with hexane/EtOAc (8:2).

^{1}H NMR (400 MHz, CDCl$_3$) δ = 8.60 (d, *J* = 5.9 Hz, 1H), 7.56 (td, *J* = 7.6, 1.9 Hz, 1H), 7.31 (d, *J* = 8.1 Hz, 2H), 7.26 – 7.16 (m, 3H), 7.15 – 7.01 (m, 3H), 6.97 (dd, *J* =6.7, 1.4 Hz, 1H), 6.92 (td, *J* = 7.6, 1.5 Hz), 4.64 – 4.57 (t, *J* = 6.0 Hz, 1H), 3.81 (s, 3H), 3.55 (m, 1H), 2.95 (m, 1H), 2.30 (s, 3H).

^{13}C NMR (100 MHz, CDCl$_3$) *δ* = 162.3, 152.6, 149.4, 140.3, 138.0, 136.6, 136.5, 129.4, 127.8, 127.5, 124.0, 123.2, 121.6, 120.7, 119.9-114.8 (m), 112.9, 56.1, 45.8, 36.0 (t, *J* = 20.5 Hz), 21.1.

^{19}F NMR (376 MHz, CDCl$_3$) δ = -88.47 (s, 2F), -115.77 (dtt, *J* = 22.2, 15.3, 4.2 Hz, 2F).

IR (film): 553, 569, 610, 705, 718, 145, 991, 1023, 1044, 1078, 1102, 1169, 1186, 1261, 1282, 1435, 1502, 2854, 2924, 3009 cm^{-1}

HRMS (+ESI-FTMS) calcd for $C_{23}H_{21}F_4NO_2$ [M+H]$^+$ m/z = 420.1581; found: 420.1574.

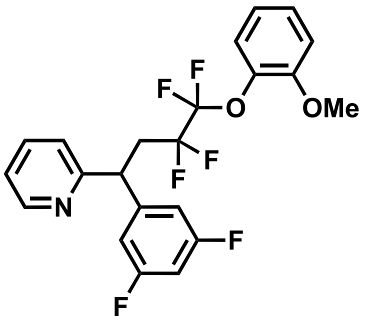

2-(1-(3,5-difluorophenyl)-3,3,4,4-tetrafluoro-4-(2-methoxyphenoxy)butyl)pyridine (4f): Compound **4f** was synthesized following the general procedure 1 (standard-scale), using 2-(2-bromo-1,1,2,2-tetrafluoroethoxy) anisole (90.3 mg, 0.3 mmol), 2-Vinylpyridine (21.7 mg, 0.2 mmol) and 3,5-difluorophenylmagnesium bromide (0.6 mL, 0.5 M solution in THF, 0.3 mmol). The product **4f** was obtained as a pale yellow liquid (28.8 mg, 32% yield) after purification by column chromatography on silica gel with hexane/EtOAc (8:2).

^{1}H NMR (400 MHz, CDCl$_3$) δ = 8.62 (d, *J* = 5.9 Hz, 1H), 7.61 (td, *J* = 7.6, 1.8 Hz, 1H), 7.26 – 7.08 (m, 4H), 7.07 – 6.74 (m, 4H), 6.65 (tt, *J* = 9.0, 2.3 Hz, 1H), 4.58 (t, *J* = 6.9 Hz, 1H), 3.83 (s, 3H), 3.53 – 3.36 (m, 1H), 3.02 – 2.86 (m, 1H).

^{13}C NMR (100 MHz, CDCl$_3$) *δ* = 163.2 (dd, J = 233.8, 12.9 Hz), 160.7, 152.5, 149.7, 146.9 (t, J = 9.4 Hz), 137.9, 137.0, 127.6, 124.0, 123.3, 122.3, 120.7, 120.9-114.5 (m), 112.9, 111.1(dd, J = 11.7, 7.0 Hz), 102.4 (t, J = 26.2 Hz), 56.1, 45.8, 36.1 (t, *J* = 19.4 Hz).

^{19}F NMR (376 MHz, CDCl$_3$) δ = -88.46 (d, *J* = 3.5 Hz, 2F), -109.63 (t, *J* = 8.3 Hz, 2F), -115.54 (dddt, *J* = 25.7, 22.2, 13.9, 4.2 Hz, 2F).

IR (film): 510, 690, 746, 849, 969, 988, 1025, 1044, 1062, 1108, 1170, 1188, 1261, 1282, 1303, 1435, 1459, 1503, 1597, 1624, 2852, 2923 cm^{-1}

HRMS (+ESI-FTMS) calcd for $C_{22}H_{17}F_6NO_2$ $[M+H]^+$ m/z = 442.1236; found: 442.1236.

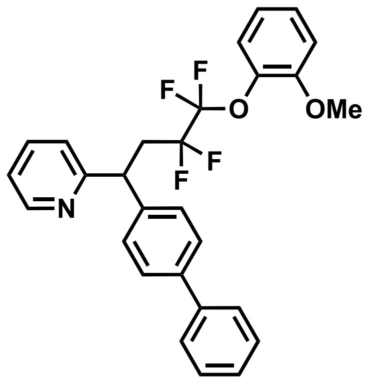

2-(1-([1,1'-biphenyl]-4-yl)-3,3,4,4-tetrafluoro-4-(2-methoxyphenoxy)butyl)pyridine (4g): Compound **4g** was synthesized following the general procedure 1 (standard-scale), using 2-(2-bromo-1,1,2,2-tetrafluoroethoxy) anisole (90.3 mg, 0.3 mmol), 2-Vinylpyridine (21.7 mg, 0.2 mmol) and 4-biphenylmagnesium bromide (0.6 mL, 0.5 M solution in THF, 0.3 mmol). The product **4g** was obtained as a pale opaque liquid (53.9 mg, 56% yield) after purification by column chromatography on silica gel with hexane/EtOAc (8:2).

^{1}H NMR (400 MHz, CDCl$_3$) δ = 8.54 (d, *J* = 4.0 Hz, 1H), 7.62 – 7.37 (m, 7H), 7.36 – 7.30 (m, 2H), 7.25 – 7.10 (m, 4H), 7.04 (ddd, *J* = 7.5, 4.8, 1.1 Hz, 1H), 6.88, *J* = 8.7, 1.4 Hz, 1H), 6.83 (td, *J* = 7.6, 1.5 Hz, 1H), 4.60 (dd, *J* = 7.9, 5.9 Hz, 1H), 3.73 (s, 3H), 3.60 – 3.43 (m, 1H), 3.03 – 2.81 (m, 1H).

^{13}C NMR (100 MHz, CDCl$_3$) *δ* = 162.0, 152.6, 149.5, 142.3, 140.9, 139.8, 138.0, 136.7, 128.8, 128.4, 127.54, 127.50, 127.3, 127.2, 124.0, 123.3, 121.8, 120.7, 117.9-114.8 (m), 112.9, 56.1, 45.9, 36.1 (t, *J* = 20.1 Hz).

^{19}F NMR (376 MHz, CDCl$_3$) δ = -88.41 (d, *J* = 4.2 Hz, 2F), -115.65 (ddq, *J* = 20.1, 16.0, 3.8 Hz, 2F).

IR (film): 571, 612, 697, 745, 991, 1025, 1044, 1078, 1103, 1170, 1187, 1217, 1261, 1281, 1435, 1488, 1502, 2852, 2923, 2956, 3010, 3029 cm^{-1};

HRMS (+ESI-FTMS) calcd for $C_{28}H_{23}F_4NO_2$ $[M+H]^+$ m/z =482.1738; found:482.1739.

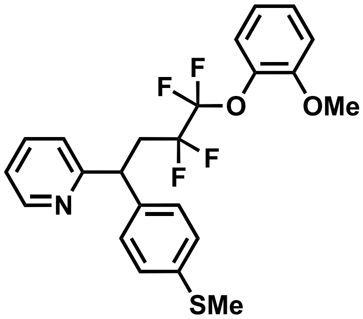

2-(3,3,4,4-tetrafluoro-4-(2-methoxyphenoxy)-1-(4-(methylthio)phenyl)butyl)pyridine (4h): Compound **4h** was synthesized following the general procedure 1 (standard-scale), using 2-(2-bromo-1,1,2,2-tetrafluoroethoxy) anisole (90.3 mg, 0.3 mmol), 2-Vinylpyridine (21.7 mg, 0.2 mmol) and 4-thioanisolemagnesium bromide (0.6 mL, 0.5 M solution in THF, 0.3 mmol). The product **4h** was obtained as a pale yellow liquid (42.2 mg, 46% yield) after purification by column chromatography on silica gel with hexane/EtOAc (8:2).

^{1}H NMR (400 MHz, CDCl$_3$) δ = 8.52 (d, *J* = 3.8 Hz, 1H), 7.49 (td, *J* = 7.7, 2.0 Hz, 1H), 7.27 (d, *J* = 8.3 Hz, 2H), 7.17 – 7.06 (m, 5H), 7.03 (dd, *J* = 7.5, 4.9 Hz, 1H), 6.89 (d, J = 8.0 Hz, 1H), 6.84 (t, *J* = 7.0 Hz, 1H), 4.51 (t, *J* = 6.9 Hz, 1H), 3.74 (s, 3H), 3.52-3.36 (m, 1H), 2.95 – 2.77 (m, 1H), 2.37 (s, 3H).

^{13}C NMR (100 MHz, CDCl$_3$) *δ* = 162.0, 152.6, 149.4, 140.2, 138.0, 136.8, 136.7, 128.5, 127.5, 127.1, 124.0, 123.2, 121.8, 120.7, 119.8-114.4 (m), 112.9, 56.1, 45.7, 36.0 (t, *J* = 20.9 Hz), 16.1.

^{19}F NMR (376 MHz, CDCl$_3$) δ = -88.46 (d, *J* = 4.2 Hz, 2F), -115.55 to -115.84 (m, 2F).

IR (film): 745, 1045, 1079, 1104, 1170, 1186, 1261, 1502, 2852, 2923, 2958, 3010 cm^{-1}

HRMS (+ESI-FTMS) calcd for $C_{23}H_{21}F_4NO_2S$ $[M+H]^+$ m/z = 452.1302; found: 452.1304.

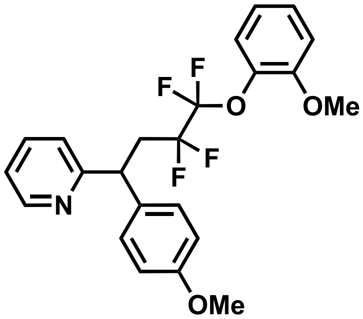

2-(3,3,4,4-tetrafluoro-4-(2-methoxyphenoxy)-1-(4-methoxyphenyl)butyl)pyridine (4i): Compound **4i** was synthesized following the general procedure 1 (standard-scale), using 2-(2-bromo-1,1,2,2-tetrafluoroethoxy) anisole (90.3 mg, 0.3 mmol), 2-Vinylpyridine (21.7 mg, 0.2 mmol) and 4-methoxyphenylmagnesium bromide (0.6 mL, 0.5 M solution in THF, 0.3 mmol). The product **4i** was obtained as a pale yellow liquid (42.3 mg, 48% yield) after purification by column chromatography on silica gel with hexane/EtOAc (8:2).

^{1}H NMR (400 MHz, CDCl$_3$) δ = 8.59 (d, *J* = 4.9 Hz, 1H), 7.56 (td, *J* = 7.6, 1.8 Hz, 1H), 7.34 (d, *J* = 8.8 Hz, 2H), 7.24 – 7.17 (m, 3H), 7.10 (ddd, *J* = 7.5, 4.8, 1.1 Hz, 1H), 6.96 (dd, *J* = 8.6,

1.5 Hz, 1H), 6.91 (td, J = 7.6, 1.4 Hz, 1H), 6.84 (d, J = 8.8 Hz, 2H), 4.59 (t, J = 6.9 Hz, 1H), 3.81 (s, 3H), 3.77 (s, 3H), 3.51 (m, J = 1H), 3.03 – 2.83 (m, 1H).

^{13}C NMR (100 MHz, CDCl$_3$) δ = 162.4, 158.5, 152.6, 149.4, 138.0, 136.6, 135.5, 129.0, 127.5, 124.0, 123.2, 121.6, 120.7, 121.0 – 114.8 (m), 114.1, 112.9, 56.1, 55.3, 45.4, 36.1 (t, J = 20.4 Hz).

^{19}F NMR (376 MHz, CDCl$_3$) δ = -88.47 (d, J = 3.5 Hz, 2F), -115.79 (tdt, J = 22.2, 13.9, 4.2 Hz, 2F).

IR (film): 561, 610, 745, 991, 1026, 1078, 1101, 1169, 1252, 1282, 1302, 1435, 1464, 1501, 2839, 2956, 3008 cm^{-1}

HRMS (+ESI-FTMS) calcd for $C_{23}H_{21}F_4NO_3$ [M+H]$^+$ m/z = 436.1529; found: 436.1529.

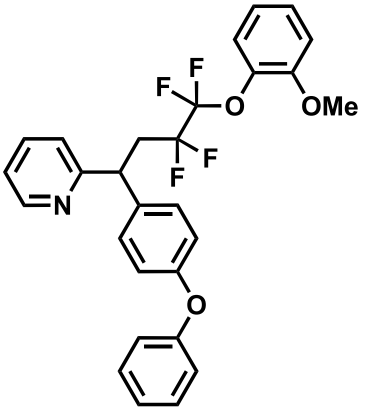

2-(3,3,4,4-tetrafluoro-4-(2-methoxyphenoxy)-1-(4-phenoxyphenyl)butyl)pyridine (4j): Compound **4j** was synthesized following the general procedure 1 (standard-scale), using 2-(2-bromo-1,1,2,2-tetrafluoroethoxy) anisole (90.3 mg, 0.3 mmol), 2-Vinylpyridine (21.7 mg, 0.2 mmol) and 4-phenoxyphenylmagnesium bromide (0.6 mL, 0.5 M solution in THF, 0.3 mmol). The product **4j** was obtained as a yellow liquid (48.4 mg, 48% yield) after purification by column chromatography on silica gel with hexane/EtOAc (8:2).

^{1}H NMR (400 MHz, CDCl$_3$) δ = 8.60 (d, J = 4.8 Hz, 1H), 7.59 (td, J = 7.6, 1.8 Hz, 1H), 7.40 – 7.35 (m, 2H), 7.37 (d, J =8.6 Hz, 2H), 7.22 (t, J = 7.6 Hz, 3H), 7.14 – 7.06 (m, 2H), 6.99 – 6.89 (m, 6H), 4.61 (t, J = 8.0 Hz, 1H), 3.82 (s, 3H), 3.59 – 3.45 (m, 1H), 3.02 – 2.87 (m, 1H).

^{13}C NMR (100 MHz, CDCl$_3$) δ = 162.1, 157.3, 156.1, 152.6, 149.4, 138.0, 136.80, 129.8, 129.7, 129.3, 127.6, 124.0, 123.35, 123.30, 121.8, 121.1, 120.7, 119.0, 119.8-113.7 (m), 117.9, 117.7, 117.3, 116.5, 112.9, 56.1, 45.5, 36.2 (t, J = 19.8 Hz).

^{19}F NMR (376 MHz, CDCl$_3$) δ = -88.43 (d, J = 4.2 Hz, 2F), -115.56 to -115.80 (m, 2F).

IR (film): 564, 691, 707, 746, 870, 992, 1024, 1044, 1078, 1103, 1168, 1187, 1235, 1261, 1488, 1503, 1589, 2853, 2923, 2957 cm^{-1}

HRMS (+APCI-FTMS) calcd for $C_{28}H_{23}F_4NO_3$ [M+H]$^+$ m/z = 498.1687; found: 498.1675.

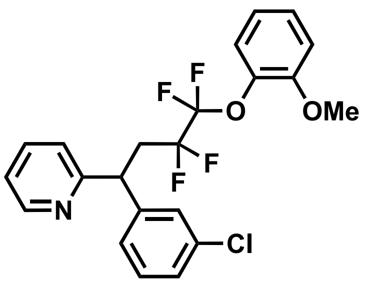

2-(1-(3-chlorophenyl)-3,3,4,4-tetrafluoro-4-(2-methoxyphenoxy)butyl)pyridine(4k): Compound **4k** was synthesized following the general procedure 1 (standard-scale), using 2-(2-bromo-1,1,2,2-tetrafluoroethoxy) anisole (90.3 mg, 0.3 mmol), 2-Vinylpyridine (21.7 mg, 0.2 mmol) and 3-chlorophenylmagnesium bromide (0.6 mL, 0.5 M solution in THF, 0.3 mmol). The product **4k** was obtained as a colorless liquid (44.9 mg, 50% yield) after purification by column chromatography on silica gel with hexane/EtOAc (8:2).

^{1}H NMR (400 MHz, CDCl$_3$) δ = 8.53 (d, *J* = 3.0 Hz, 1H), 7.50 (t, *J* = 7.7 Hz, 1H), 7.35 (s, 1H), 7.24 (d, *J* = 7.4 Hz, 1H), 7.19 – 7.03 (m, 6H), 6.95 – 6.79 (m, 2H), 4.52 (t, *J* = 6.9 Hz, 1H), 3.74 (s, 3H), 3.52-3.34 (m, 1H), 2.95 – 2.76 (m, 1H).

^{13}C NMR (100 MHz, CDCl$_3$) *δ* = 161.3, 152.6, 149.6, 145.2, 137.9, 136.8, 134.5, 130.0, 128.2, 127.6, 127.1, 126.3, 124.0, 123.3, 122.0, 120.7, 120.1-117.2 (m), 112.9, 56.1, 45.9, 36.1 (t, *J* = 21.3 Hz).

^{19}F NMR (376 MHz, CDCl$_3$) δ = -88.45 (d, *J* = 4.2 Hz, 2F), -115.50 to -115.72 (m, 2F).

IR (film): 682, 695, 744, 992, 1026, 1044, 1077, 1105, 1169, 1187, 1261, 1282, 1432, 1502, 2841, 2961, 3011, 3070 cm^{-1}

HRMS (+APCI-FTMS) calcd for $C_{22}H_{18}ClF_4NO_2$ $[M+H]^+$ m/z = 440.1035; found: 440.1024.

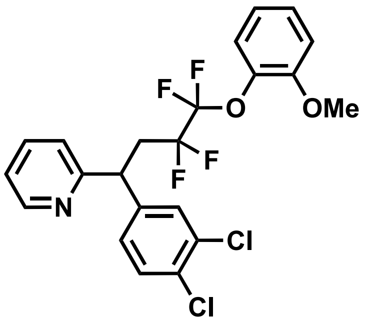

2-(1-(3,4-dichlorophenyl)-3,3,4,4-tetrafluoro-4-(2-methoxyphenoxy)butyl)pyridine (4l): Compound **4l** was synthesized following the general procedure 1 (standard-scale), using 2-(2-bromo-1,1,2,2-tetrafluoroethoxy) anisole (90.3 mg, 0.3 mmol), 2-Vinylpyridine (21.7 mg, 0.2 mmol) and 3,4-dichlorophenylmagnesium bromide (0.6 mL, 0.5 M solution in THF, 0.3 mmol). The product **4l** was obtained as a colorless liquid (30.2 mg, 31% yield) after purification by column chromatography on silica gel with hexane/EtOAc (8:2).

^{1}H NMR (400 MHz, CDCl$_3$) δ = 8.51 (d, *J* =4.9 Hz, 1H), 7.51 (td, *J* = 7.7, 1.9 Hz, 1H), 7.44 (d, *J* = 2.1 Hz, 1H), 7.27 (d, *J* = 8.4 Hz, 1H), 7.23 – 7.08 (m, 4H), 7.08 – 7.05 (ddd, 7.5, 2.6, 1.1 Hz, 1H), 6.88 (dd, *J* =8.3, 1.5 Hz, 1H), 6.86 (td, *J* = 1.5, 7.5 Hz, 1 H) 4.48 (t, *J* = 6.9 Hz, 1H), 3.73

(s, 3H), 3.36 (dddd, J = 22.8, 15.4, 12.6, 7.4 Hz, 1H), 2.85 (dddd, J = 22.0, 15.4, 13.1, 6.4 Hz, 1H).

^{13}C NMR (100 MHz, CDCl$_3$) δ = 160.9, 152.5, 149.7, 143.3, 137.9, 137.0, 132.6, 131.0, 130.7, 130.1, 127.6, 127.5, 124.0, 123.2, 122.2, 120.7, 119.6-115.1 (m), 112.9, 56.1, 45.4, 36.1 (t, J = 20.9 Hz).

^{19}F NMR (376 MHz, CDCl$_3$) δ = -88.46 (d, J = 4.2 Hz, 2F), -115.49 (dddt, J = 52.7, 22.9, 11.8, 4.2 Hz, 2F).

IR (film): 683, 706, 745, 993, 1028, 1044, 1105, 1170, 1187, 1217, 1261, 1282, 1436, 1468, 1502, 2841, 2960, 3012, 3071 cm^{-1}

HRMS (+APCI-FTMS) calcd for $C_{22}H_{17}Cl_2F_4NO_2$ $[M+H]^+$ m/z = 474.0645; found: 474.0628

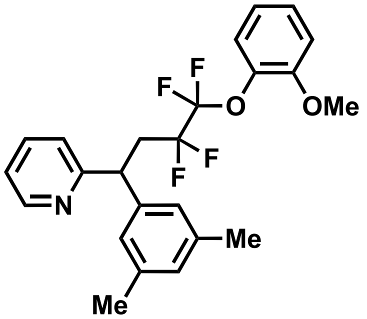

2-(1-(3,5-dimethylphenyl)-3,3,4,4-tetrafluoro-4-(2-methoxyphenoxy)butyl)pyridine (4m): Compound **4m** was synthesized following the general procedure 1 (standard-scale), using 2-(2-bromo-1,1,2,2-tetrafluoroethoxy) anisole (90.3 mg, 0.3 mmol), 2-Vinylpyridine (21.7 mg, 0.2 mmol) and 3,4-dimethylphenylmagnesium bromide (0.6 mL, 0.5 M solution in THF, 0.3 mmol). The product **4m** was obtained as a pale yellow liquid (57.1 mg, 65% yield) after purification by column chromatography on silica gel with hexane/EtOAc (8:2).

^{1}H NMR (400 MHz, CDCl$_3$) δ = 8.64 (d, J = 3.1 Hz, 1H), 7.61 (td, J = 7.6, 1.8 Hz, 1H), 7.31 – 7.24 (m, 3H), 7.17 – 7.12 (m, 1H), 7.07 (s, 2H), 7.05 – 6.86 (m, 3H), 4.60 (dd, J = 8.2, 5.5 Hz, 1H), 3.86 (s, 3H), 3.69 – 3.53 (m, 1H), 3.04 – 2.86 (m, 1H), 2.33 (s, 6H).

^{13}C NMR (100 MHz, CDCl$_3$) δ = 162.2, 152.6, 149.3, 143.2, 138.2, 138.0, 136.6, 128.6, 127.5, 125.7, 124.0, 123.3, 121.6, 120.7, 119.9-113.2 (m), 112.9, 56.1, 46.1, 35.9 (t, J = 20.9 Hz), 21.5.

^{19}F NMR (376 MHz, CDCl$_3$) δ = -88.37 to -88.52 (m, 2F), -115.88 (tt, J = 18.0, 4.2 Hz, 2F).

IR (film): 703, 745, 852, 990, 1025, 1044, 1063, 1105, 1170, 1186, 1219, 1261, 1282, 1302, 1434, 1464, 1502, 1589, 2841, 2920, 2948, 3010 cm^{-1}

HRMS (+APCI-FTMS) calcd for $C_{24}H_{23}F_4NO_2$ $[M+H]^+$ m/z =434.1738; found: 434.1720.

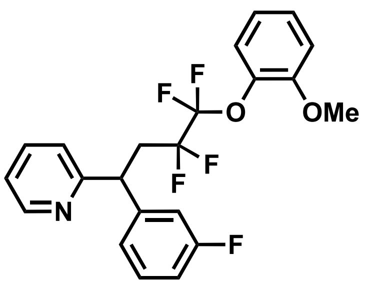

2-(3,3,4,4-tetrafluoro-1-(3-fluorophenyl)-4-(2-methoxyphenoxy)butyl)pyridine (4n): Compound **4n** was synthesized following the general procedure 1 (standard-scale), using 2-(2-bromo-1,1,2,2-tetrafluoroethoxy) anisole (90.3 mg, 0.3 mmol), 2-Vinylpyridine (21.7 mg, 0.2 mmol) and 3-fluorophenylmagnesium bromide (0.3 mL, 1.0 M solution in THF, 0.3 mmol). The product **4n** was obtained as a colorless liquid (47.9 mg, 56% yield) after purification by column chromatography on silica gel with hexane/EtOAc (8:2).

^{1}H NMR (400 MHz, CDCl$_3$) δ = 8.66 (d, *J* = 4.8 Hz, 1H), 7.64 (td, *J* = 7.6, 1.8 Hz, 1H), 7.34 – 7.17 (m, 7H), 7.11 – 6.82 (m, 3H), 4.68 (t, *J* = 6.9 Hz, 1H), 3.87 (s, 3H), 3.64 – 3.47 (m, 1H), 3.10 – 2.93 (m, 1H).

^{13}C NMR (100 MHz, CDCl$_3$) *δ* = 163.0 (d, *J* = 244 Hz), 161.4, 152.6, 149.5, 145.7 (d, *J* = 6.8 Hz), 137.9, 136.8, 130.2 (d, *J* = 8.3 Hz), 127.6, 124.0, 123.7 (d, J = 3.0 Hz), 123.3, 122.0, 120.7, 119.7-114.3 (m), 115.0 (d, *J* = 21.1 Hz), 113.8 (d, *J* = 21.1 Hz), 112.9, 56.1, 45.9, 36.1 (t, *J* = 21.1 Hz).

^{19}F NMR (376 MHz, CDCl$_3$) δ = -88.46 (d, *J* = 4.2 Hz, 2F), -112.80 (td, *J* = 9.4, 5.5 Hz, 1F), -115.64 (dtd, *J* = 25.7, 12.8, 7.3 Hz, 2F).

IR (film): 521, 695, 710, 745, 955, 994, 1026, 1044, 1105, 1170, 1187, 1261, 1435, 1502, 1590, 2841, 2948, 3012, 3070 cm^{-1}

HRMS (+APCI-FTMS) calcd for $C_{22}H_{18}F_5NO_2$ $[M+H]^+$ m/z = 424.1330; found: 424.1330.

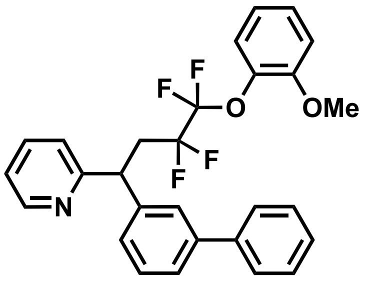

2-(1-([1,1'-biphenyl]-3-yl)-3,3,4,4-tetrafluoro-4-(2-methoxyphenoxy)butyl)pyridine (4o): Compound **4o** was synthesized following the general procedure 1 (standard-scale), using 2-(2-bromo-1,1,2,2-tetrafluoroethoxy) anisole (90.3 mg, 0.3 mmol), 2-Vinylpyridine (21.7 mg, 0.2 mmol) and 3-biphenylphenylmagnesium bromide (0.6 mL, 0.5 M solution in THF, 0.3 mmol). The product **4o** was obtained as an orange liquid (68.2 mg, 70% yield) after purification by column chromatography on silica gel with hexane/EtOAc (8:2).

^{1}H NMR (400 MHz, CDCl$_3$) δ = 8.62 (d, *J* = 6.0 Hz, 1H), 7.66 (s, 1H), 7.62 – 7.51 (m, 3H), 7.50 – 7.31 (m, 6H), 7.28 – 7.20 (m, 3H), 7.16 – 7.07 (m, 1H), 7.04 – 6.86 (m, 2H), 4.71 (t, *J* = 6.9 Hz, 1H), 3.80 (s, 3H), 3.70 – 3.54 (m, 1H), 3.03 – 2.83 (m, 1H).

^{13}C NMR (100 MHz, CDCl$_3$) *δ* = 162.0, 152.6, 149.5, 143.8, 141.7, 141.3, 138.0, 136.7, 129.2, 128.8, 127.5, 127.43, 127.46, 127.0, 126.9, 125.8, 124.0, 123.3, 121.8, 120.7, 119.9-114.8 (m), 112.9, 56.1, 46.3, 36.1 (t, *J* = 21.1 Hz).

^{19}F NMR (376 MHz, CDCl$_3$) δ = -88.31 to -88.54 (m, 2F), -115.63 (dddt, *J* = 19.4, 15.3, 6.9, 4.2 Hz, 2F).

IR (film): 698, 746, 990, 1025, 1044, 1077, 1105, 1169, 1187, 1261, 1282, 1303, 1435, 1474, 1501, 1590, 2840, 2930, 3011, 3033, 3061 cm^{-1}

HRMS (+APCI-FTMS) calcd for $C_{28}H_{23}F_4NO_2$ $[M+H]^+$ m/z = 482.1738; found: 482.1738.

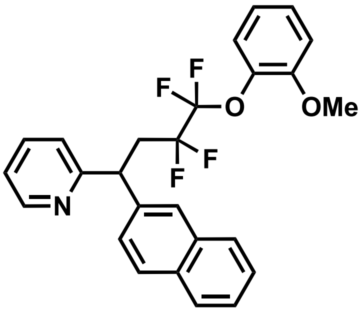

2-(3,3,4,4-tetrafluoro-4-(2-methoxyphenoxy)-1-(naphthalen-2-yl)butyl)pyridine (4p): Compound **4p** was synthesized following the general procedure 1 (standard-scale), using 2-(2-bromo-1,1,2,2-tetrafluoroethoxy) anisole (90.3 mg, 0.3 mmol), 2-Vinylpyridine (21.7 mg, 0.2 mmol) and 2-naphthylmagnesium bromide (0.6 mL, 0.5 M solution in THF, 0.3 mmol). The product **4p** was obtained as a orange liquid (52.8 mg, 57% yield) after purification by column chromatography on silica gel with hexane/EtOAc (8:2).

^{1}H NMR (400 MHz, CDCl$_3$) δ = 8.63 (d, *J* = 2.1 Hz, 1H), 7.91 – 7.68 (m, 4H), 7.60 – 7.52 (m, 2H), 7.48 – 7.37 (m, 2H), 7.29 – 7.18 (m, 3H), 6.99 – 6.88 (m, 2H), 4.81 (t, *J* = 6.8 Hz, 1H), 3.80 (s, 3H), 3.73 – 3.55 (m, 1H), 3.14 – 2.99 (m, 1H).

^{13}C NMR (100 MHz, CDCl$_3$) *δ* = 162.0, 152.6, 149.4, 140.7, 138.0, 136.7, 133.7, 132.5, 128.5, 128.0, 127.7, 127.5, 126.5, 126.3, 126.2, 125.8, 124.0, 123.5, 121.8, 120.7, 119.8 – 114.8 (m),112.9, 56.1, 46.4, 35.9 (t, *J* = 20.4 Hz).

^{19}F NMR (376 MHz, CDCl$_3$) δ = -88.39 (d, *J* = 4.9 Hz, 2F), -115.47 to -115.71 (m, 2F).

IR (film): 477, 701, 744, 817, 993, 1025, 1044, 1079, 1103, 1169, 1186, 1261, 1282, 1434, 1501, 1589, 2840, 2928, 3010, 3056 cm^{-1}

HRMS (+APCI-FTMS) calcd for $C_{26}H_{21}F_4NO_2$ $[M+H]^+$ m/z = 456.1581; found: 456.1579.

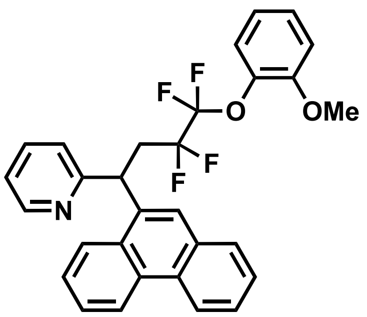

2-(3,3,4,4-tetrafluoro-4-(2-methoxyphenoxy)-1-(phenanthren-9-yl)butyl)pyridine (4q): Compound **4q** was synthesized following the general procedure 1 (standard-scale), using 2-(2-bromo-1,1,2,2-tetrafluoroethoxy) anisole (90.3 mg, 0.3 mmol), 2-Vinylpyridine (21.7 mg, 0.2 mmol) and 9-phenanthrylmagnesium bromide (0.6 mL, 0.5 M solution in THF, 0.3 mmol). The product **4q** was obtained as a brown liquid (20.7 mg, 20% yield) after purification by column chromatography on silica gel with hexane/EtOAc (8:2).

^{1}H NMR (400 MHz, CDCl$_3$) δ = 8.78 – 8.72 (m, 1H), 8.69 (d, *J* = 1.0, 3.88 Hz, 1H), 8.66 (d, *J* = 7.4 Hz, 1H), 8.39 (d, *J* = 8.5 Hz, 1H), 7.85 (dd, *J* = 7.8, 1.8 Hz, 1H), 7.79 (s, 1H), 7.67 – 7.51 (m, 5H), 7.29 (d, *J* = 8.0 Hz, 1H), 7.23 – 7.17 (m, 2H), 7.14 (ddd, *J* = 7.4, 4.9, 1.2 Hz, 1H), 6.95 – 6.88 (m, 2H), 5.57 – 5.45 (m, 1H), 3.91 – 3.81 (m, 1H), 3.73 (s, 3H), 3.20 – 3.05 (m, 1H).

^{13}C NMR (100 MHz, CDCl$_3$) *δ* = 161.7, 152.6, 149.3, 138.0, 137.3, 136.7, 131.7, 131.2, 130.4, 129.9, 128.9, 127.5, 127.0, 126.9, 126.8, 126.7, 126.4, 124.4, 124.0, 123.6, 123.5, 122.5, 121.8, 120.7, 120.0-114.4 (m), 112.9, 56.1, 41.6, 35.3.

^{19}F NMR (376 MHz, CDCl$_3$) δ = -88.41 (d, *J* = 4.2 Hz, 2F), -115.78 (bs, 2F).

IR (film): 665, 723, 743, 981, 1025, 1043, 1105, 1169, 1187, 1261, 1501, 2840, 2959, 3011, 3069 cm^{-1}

HRMS (+APCI-FTMS) calcd for $C_{30}H_{23}F_4NO_2$ $[M+H]^+$ m/z = 506.1738; found: 506.1738.

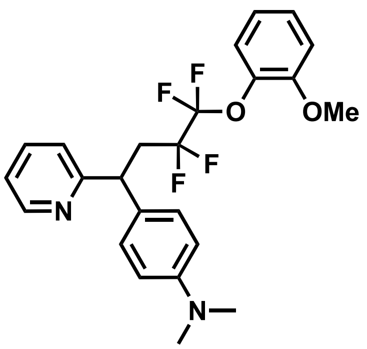

***N*,*N*-dimethyl-4-(3,3,4,4-tetrafluoro-4-(2-methoxyphenoxy)-1-(pyridin-2-yl)butyl)aniline (4r):** Compound **4r** was synthesized following the general procedure 1 (standard-scale), using 2-(2-bromo-1,1,2,2-tetrafluoroethoxy) anisole (90.3 mg, 0.3 mmol), 2-Vinylpyridine (21.7 mg, 0.2 mmol) and 4-(N,N-Dimethyl)aniline magnesium bromide (0.6 mL, 0.5 M solution in THF, 0.3 mmol). The product **4r** was obtained as a brown liquid (42.8 mg, 47% yield) after purification by column chromatography on silica gel with hexane/EtOAc (8:2).

^{1}H NMR (400 MHz, CDCl$_3$) δ = 8.59 (dd, *J* = 4.9, 0.9 Hz, 1H), 7.55 (td, *J* = 7.6, 1.8 Hz, 1H), 7.27 (d, *J* = 8.7 Hz, 2H), 7.24 – 7.19 (m, 3H), 7.08 (ddd, *J* = 7.5, 4.9, 1.1 Hz, 1H), 6.98 - 6.89 (m, 2H) 6.69 (d, *J* = 8.8, 2H), 4.55 (dd, *J* = 8.1, 5.7 Hz, 1H), 3.82 (s, 3H), 3.63 – 3.46 (m, 1H), 3.00 – 2.92 (m, 1H), 2.91 (s, 6H).

^{13}C NMR (100 MHz, CDCl$_3$) *δ* = 162.9, 152.6, 149.5, 149.3, 138.1, 136.5, 131.3, 128.5, 127.5, 124.1, 123.1, 121.4, 120.6, 119.9-113.2 (m), 113.0, 112.9, 56.1, 45.2, 40.7, 36.0 (t, *J* = 20.8).

^{19}F NMR (376 MHz, CDCl$_3$) δ = -88.48 (d, *J* = 3.5 Hz, 2F), -115.89 (tt, *J* = 19.4, 4.2 Hz, 2F).

IR (film): 553, 569, 609, 746, 768, 818, 947, 990, 1025, 1044, 1078, 1102, 1168, 1186, 1219, 1261, 1282, 1303, 1346, 1435, 1464, 1501, 1520, 1590, 1612, 2803, 2853, 2923, 3008 cm^{-1}

HRMS (+APCI-FTMS) calcd for $C_{24}H_{24}F_4N_2O_2$ $[M+H]^+$ m/z = 449.1847; found: 449.1845.

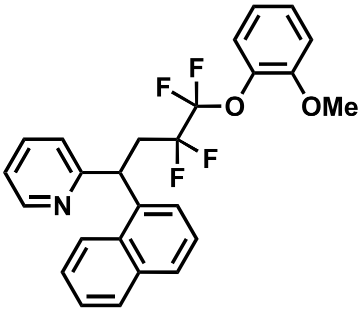

2-(3,3,4,4-tetrafluoro-4-(2-methoxyphenoxy)-1-(naphthalen-1-yl)butyl)pyridine (4s): Compound **4s** was synthesized following the general procedure 1 (standard-scale), using 2-(2-bromo-1,1,2,2-tetrafluoroethoxy) anisole (90.3 mg, 0.3 mmol), 2-Vinylpyridine (21.7 mg, 0.2 mmol) and 1-naphthylmagnesium bromide (1.2 mL, 0.25 M slurry in THF, 0.3 mmol). The product **4s** was obtained as a brown liquid (27.1 mg, 29% yield) after purification by column chromatography on silica gel with hexane/EtOAc (8:2).

^{1}H NMR (400 MHz, CDCl$_3$) δ = 8.65 (d, *J* = 3.8 Hz, 1H), 8.36 (d, *J* = 8.5 Hz, 1H), 7.87 (d, *J* = 9.4 Hz, 1H), 7.76 (d, *J* = 8.1 Hz, 1H), 7.59 – 7.41 (m, 5H), 7.27 – 7.18 (m, 3H), 7.12 (dd, *J* = 8.5, 4.8 Hz, 1H), 6.98 – 6.87 (m, 2H), 5.51 (dd, *J* = 8.3, 5.0 Hz, 1H), 3.92 – 3.77 (m, 1H), 3.74 (s, 3H), 3.12 – 2.96 (m, 1H).

^{13}C NMR (100 MHz, CDCl$_3$) *δ* = 161.7, 152.5, 149.1, 139.1, 137.9, 136.5, 134.2, 131.1, 129.0, 127.5, 127.4, 126.4, 125.7, 125.5, 123.9, 123.4, 123.4, 121.6, 120.5, 120.9-114.6 (m), 112.8, 55.9, 41.1, 35.50 (t, *J* = 22.1 Hz).

^{19}F NMR (376 MHz, CDCl$_3$) δ = -88.41 (d, *J* = 4.9 Hz, 2F), -115.80 (q, *J* = 23.9 Hz, 2F).

IR (film): 746, 779, 984, 1025, 1044, 1078, 1104, 1169, 1186, 1261, 1281, 1433, 1501, 1589, 2840, 2959, 3009, 3050 cm^{-1}

HRMS (+APCI-FTMS) calcd for $C_{26}H_{21}F_4NO_2$ $[M+H]^+$ m/z = 456.1581; found: 456.1566.

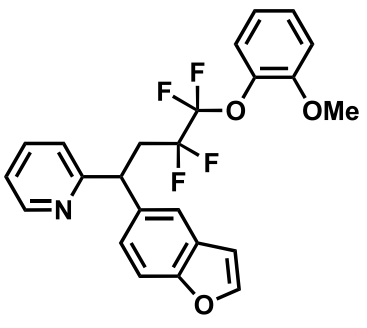

2-(1-(benzofuran-5-yl)-3,3,4,4-tetrafluoro-4-(2-methoxyphenoxy)butyl)pyridinepyridine (4t): Compound **4t** was synthesized following the general procedure 1 (standard-scale), using 2-(2-bromo-1,1,2,2-tetrafluoroethoxy) anisole (90.3 mg, 0.3 mmol), 2-Vinylpyridine (21.7 mg, 0.2 mmol) and benzofuran-5-ylmagnesium bromide lithium chloride complex (0.4 mL, 0.76 M solution in THF, 0.3 mmol). The product **4t** was obtained as a yellow liquid (40.5 mg, 45% yield) after purification by column chromatography on silica gel with hexane/EtOAc (8:2).

^{1}H NMR (400 MHz, CDCl$_3$) δ = 8.62 (d, J = 4.9 Hz, 1H), 7.66 (d, J = 1.8 Hz, 1H), 7.61 – 7.51 (m, 2H), 7.45 – 7.34 (m, 2H), 7.26 – 7.20 (m, 3H), 7.11 (ddd, J = 7.5, 4.8, 1.1 Hz, 1H), 6.96 (dd, J = 8.6, 1.5 Hz, 1H), 6.91 (td, J = 7.6, 1.5 Hz, 1H), 6.72 (dd, J = 2.1, 1.0 Hz, 1H), 4.75 (dd, J = 7.7, 6.0 Hz, 1H), 3.80 (s, 3H), 3.69 – 3.53 (m, 1H), 3.14 – 2.89 (m, 1H).

^{13}C NMR (100 MHz, CDCl$_3$) δ = 162.4, 154.1, 152.6, 149.4, 145.5, 138.0, 136.7, 127.8, 127.5, 124.4, 124.0, 123.3, 121.7, 120.7, 120.4, 119.9-106.3 (m), 112.9, 111.5, 106.8, 56.1, 46.0, 36.4 (t, J = 20.2 Hz).

^{19}F NMR (376 MHz, CDCl$_3$) δ = -88.43 (dt, J = 8.3, 3.8 Hz, 2F), -115.72 (dddt, J = 36.1, 22.2, 13.2, 4.2 Hz, 2F).

IR (film): 550, 660, 712, 745, 992, 1027, 1044, 1102, 1169, 1186, 1260, 1281, 1303, 1435, 1466, 1501, 1590, 2841, 2947, 3010, 3070 cm^{-1}

HRMS (+APCI-FTMS) calcd for $C_{24}H_{19}F_4NO_3$ $[M+H]^+$ m/z = 446.1374; found: 446.1361.

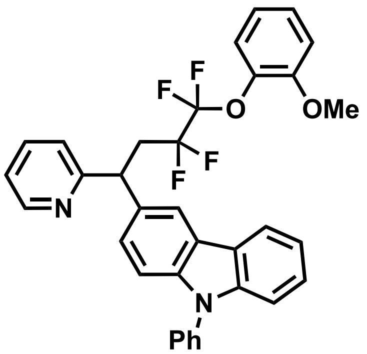

9-phenyl-3-(3,3,4,4-tetrafluoro-4-(2-methoxyphenoxy)-1-(pyridin-2-yl)butyl)-9*H*-carbazole (4u): Compound **4u** was synthesized following the general procedure 1 (standard-scale), using 2-(2-bromo-1,1,2,2-tetrafluoroethoxy) anisole (90.3 mg, 0.3 mmol), 2-Vinylpyridine (21.7 mg, 0.2 mmol) and (9-Phenyl-9*H*-carbazol-3-yl)magnesium bromide (0.27 mL, 1.1 M solution in THF, 0.3 mmol). The product **4u** was obtained as an opaque liquid (59.7

mg, 51% yield) after purification by column chromatography on silica gel with hexane/EtOAc (8:2).

^{1}H NMR (400 MHz, $CDCl_3$) δ = 8.64 (d, *J* = 4.9 Hz, 1H), 8.19 (d, *J* = 1.8 Hz, 1H), 8.13 (dt, *J* = 7.9, 1.1 Hz, 1H), 7.61 – 7.54 (m, 3H), 7.54 – 7.50 (m, 2H), 7.49 – 7.41 (m, 2H), 7.39 – 7.18 (m, 7H) 7.11 (ddd, *J* = 7.5, 4.9, 1.3 Hz, 1H), 6.96 (dd, *J* = 8.8, 1.5 Hz, 1H), 6.91 (td, *J* = 7.6, 1.5 Hz, 1H), 4.85 (dd, *J* = 7.9, 6.0 Hz, 1H), 3.81 (s, 3H), 3.78 – 3.61 (m, 1H), 3.16 – 3.01 (m, 1H).

^{13}C NMR (100 MHz, $CDCl_3$) *δ* = 162.6, 152.5, 149.2, 141.2, 139.9, 137.9, 137.7, 136.6, 135.0, 129.8, 127.39, 127.37, 127.0, 126.0, 124.0, 123.6, 123.3, 123.2, 121.5, 120.6, 120.4, 119.9, 119.4, 118.9-114.2 (m), 112.8, 109.9, 109.8, 56.0, 46.1, 36.3 (t, *J* = 20.2 Hz).

^{19}F NMR (376 MHz, $CDCl_3$) δ = -88.42 (d, *J* = 4.2 Hz, 2F), -115.68 (tdt, *J* = 21.5, 13.9, 3.8 Hz, 2F).

IR (film): 642, 699, 745, 992, 1027, 1045, 1104, 1170, 1186, 1232, 1261, 1282, 1303, 1434, 1456, 1483, 1500, 1596, 2853, 2923, 3010, 3066 cm^{-1}

HRMS (+APCI-FTMS) calcd for $C_{34}H_{26}F_4N_2O_2$ $[M+H]^+$ m/z = 571.2003; found: 571.1990.

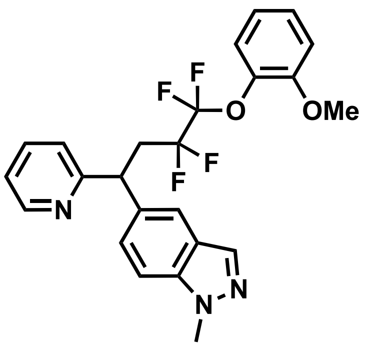

1-methyl-5-(3,3,4,4-tetrafluoro-4-(2-methoxyphenoxy)-1-(pyridin-2-yl)butyl)-1*H*-indazole (4v): Compound **4v** was synthesized following the general procedure 1 (standard-scale), using 2-(2-bromo-1,1,2,2-tetrafluoroethoxy) anisole (90.3 mg, 0.3 mmol), 2-Vinylpyridine (21.7 mg, 0.2 mmol) and (3-methyl-3*H*-indazol-6-yl)magnesium bromide lithium chloride complex (0.43 mL, 0.70 M solution in THF, 0.3 mmol). The product **4v** was obtained as a orange liquid (39.1 mg, 42% yield) after purification by column chromatography on silica gel with hexane/EtOAc (8:2).

^{1}H NMR (400 MHz, $CDCl_3$) δ = 8.61 (d, *J* = 4.9 Hz, 1H), 7.92 (d, *J* = 1.0 Hz, 1H), 7.75 (s, 1H), 7.57 (td, *J* = 7.7, 1.9 Hz, 1H), 7.50 (dd, *J* = 8.8, 1.8 Hz, 1H), 7.32 (d, *J* = 8.8 Hz, 1H), 7.26 – 7.19 (m, 3H), 7.12 (ddd, *J* = 7.5, 4.9, 1.1 Hz, 1H), 6.96 (dd, *J* = 8.6, 1.5 Hz, 1H), 6.91 (td, *J* = 7.8, 1.5 Hz, 1H), 4.77 (t, *J* = 6.9 Hz, 1H), 4.02 (s, 3H), 3.80 (s, 3H), 3.67 – 3.52 (m, 1H), 3.10 – 2.92 (m, 1H).

^{13}C NMR (100 MHz, $CDCl_3$) *δ* =162.3, 152.6, 150.8, 149.3, 139.2, 138.0, 136.8, 136.0, 135.6, 132.8, 131.6, 127.5, 126.9, 124.7, 124.3, 124.0, 123.3, 121.8, 120.7, 119.8, 118.1, 117.9-113.3 (m), 112.9, 109.9, 109.3, 103.8, 56.1, 45.9, 36.20 (t, *J* = 20.3 Hz), 35.6.

^{19}F NMR (376 MHz, CDCl$_3$) δ = -88.44 (d, *J* = 4.2 Hz, 2F), -115.47 to -115.88 (m, 2F).

IR (film): 549, 615, 686, 715, 745, 803, 845, 991, 1025, 1044, 1104, 1169, 1186, 1218, 1261, 1282, 1304, 1434, 1457, 1502, 1590, 2840, 2943, 3009, 3069 cm^{-1}

HRMS (+APCI-FTMS) calcd for $C_{24}H_{21}F_4N_3O_2$ $[M+H]^+$ m/z = 460.1643; found: 460.1630.

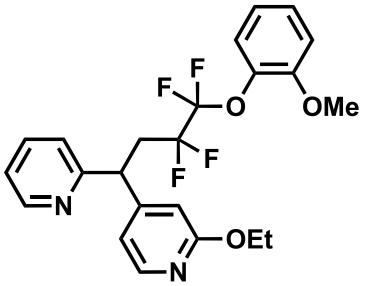

2-ethoxy-4-(3,3,4,4-tetrafluoro-4-(2-methoxyphenoxy)-1-(pyridin-2-yl)butyl)pyridine (4w): Compound **4w** was synthesized following the general procedure 1 (standard-scale), using 2-(2-bromo-1,1,2,2-tetrafluoroethoxy) anisole (90.3 mg, 0.3 mmol), 2-Vinylpyridine (21.7 mg, 0.2 mmol) and (6-ethoxypyridin-3-yl)magnesium bromide lithium chloride complex (0.35 mL, 0.86 M solution in THF, 0.3 mmol). The product **4w** was obtained as a colorless liquid (47.5 mg, 52% yield) after purification by column chromatography on silica gel with hexane/EtOAc (8:2).

^{1}H NMR (400 MHz, CDCl$_3$) δ = 8.60 (d, *J* = 4.8 Hz, 1H), 8.05 (d, *J* = 5.4 Hz, 1H), 7.59 (td, *J* = 7.7, 1.9 Hz, 1H), 7.25 – 7.17 (m, 3H), 7.14 (ddd, *J* = 7.5, 4.8, 1.1 Hz, 1H), 6.98 -6.88 (m, 1H), 6.77 (s, 1H), 4.55 (t, *J* = 6.2 Hz, 1H), 4.32 (q, *J* = 7.1 Hz, 2H), 3.82 (s, 3H), 3.55 – 3.40 (m, 1H), 3.03 – 2.87 (m, 1H), 1.37 (t, *J* = 7.1 Hz, 3H).

^{13}C NMR (100 MHz, CDCl$_3$) *δ* = 164.4, 160.5, 154.5, 152.6, 149.7, 147.2, 137.9, 136.8, 127.6, 124.0, 123.3, 122.2, 121.6-114.6 (m), 120.7, 116.5, 112.9, 110.2, 61.8, 56.1, 45.5, 35.5 (t, *J* = 20.6 Hz), 14.8.

^{19}F NMR (376 MHz, CDCl$_3$) δ = -88.44 (d, *J* = 4.2 Hz, 2F), -115.53 (dddt, *J* = 19.4, 15.9, 11.8, 3.8 Hz, 2F).

IR (film): 674, 713, 746, 992, 1042, 1104, 1169, 1187, 1219, 1261, 1282, 1315, 1382, 1421, 1502, 1559, 1590, 1608, 2841, 2903, 2946, 2979, 3010, 3070 cm^{-1}

HRMS (+APCI-FTMS) calcd for $C_{23}H_{22}F_4N_2O_3$ $[M+H]^+$ m/z = 451.1639; found: 451.1633.

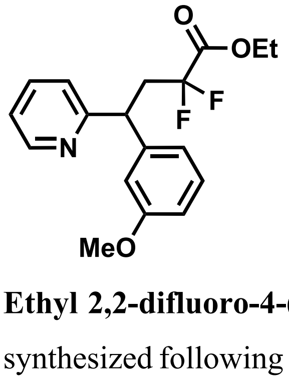

Ethyl 2,2-difluoro-4-(3-methoxyphenyl)-4-(pyridin-2-yl)butanoate (4x): Compound **4x** was synthesized following the general procedure 1 (standard-scale), using ethyl bromodifluoroacetate

(60.9 mg, 0.3 mmol), 2-Vinylpyridine (21.7 mg, 0.2 mmol) and 3-methoxyphenylmagnesium bromide (0.3 mL, 1.0 M solution in THF, 0.3 mmol). The product **4x** was obtained as a clear solid (21.2 mg, 32% yield) after purification by column chromatography on silica gel with hexane/EtOAc (8:2).

^{1}H NMR (400 MHz, CDCl$_3$) δ = 8.56 (dd, J = 4.9, 2.0 Hz, 1H), 7.55 (td, J = 7.6, 1.8 Hz, 1H), 7.19 (t, J = 7.9 Hz, 1H), 7.15 (d, J = 7.9 Hz, 1H), 7.13 – 7.07 (m, 1H), 6.95 – 6.87 (m, 2H), 6.73 (dd, J = 8.2, 2.6 Hz, 1H), 4.36 (t, J = 7.0 Hz, 1H), 4.05 – 3.91 (m, 2H), 3.76 (s, 3H), 2.44 – 3.28 (m, 1H), 2.92 – 2.77 (m, 1H), 1.21 (t, J = 7.2 Hz, 3H).

^{13}C NMR (100 MHz, CDCl$_3$) δ = 164.1 (t, J = 32.5 Hz), 161.4, 159.8, 149.2, 144.1, 136.7, 129.7, 123.5, 121.9, 120.5, 115.93 (t, J = 248.8 Hz), 113.97, 112.34, 62.77, 55.31, 46.8 (t, J = 4.33 Hz), 39.4 (t, J = 23.1 Hz), 13.87.

^{19}F NMR (376 MHz, CDCl$_3$) δ = -104.02 (dt, J = 122.1, 16.3 Hz, 2F).

IR (film): 625, 665, 699, 747, 854, 944, 995, 1048, 1074, 1150, 1189, 1216, 1261, 1298, 1375, 1434, 1455, 1472, 1489, 1570, 1589, 1762, 2837, 2941 cm^{-1}

HRMS (+APCI-FTMS) calcd for $C_{18}H_{19}F_2NO_3$ [M+H]$^+$ m/z = 336.1406; found: 336.1400.

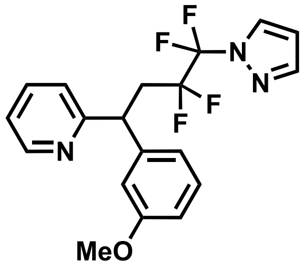

2-(3,3,4,4-tetrafluoro-1-(3-methoxyphenyl)-4-(1H-pyrazol-1-yl)butyl)pyridine (4y): Compound **4y** was synthesized following the general procedure 1 (standard-scale), using pyrazolyltetrafluorobromoethane (74.1 mg, 0.3 mmol), 2-Vinylpyridine (21.7 mg, 0.2 mmol) and 3-methoxyphenylmagnesium bromide (0.3 mL, 1.0 M solution in THF, 0..3 mmol). The product **4y** was obtained as a pale yellow oil (49.9 mg, 66% yield) after purification by column chromatography on silica gel with hexane/EtOAc (8:2).

^{1}H NMR (400 MHz, CDCl$_3$) δ = 8.56 (d, J = 5.8 Hz, 1H), 7.76 – 7.70 (m, 2H), 7.54 (td, J = 7.6, 1.8 Hz, 1H), 7.22 – 7.13 (m, 2H), 7.09 (ddd, J = 7.5, 4.9, 1.1 Hz, 1H), 6.96 – 6.89 (m, 2H), 6.73 (ddd, J = 8.3, 2.6, 1.0 Hz, 1H), 6.41 (t, J = 2.6 Hz, 1H), 4.47 (dd, J = 7.8, 5.8 Hz, 1H), 3.76 (s, 3H), 3.58 – 3.42 (m, 1H), 2.93 – 2.77 (m, 1H).

^{13}C NMR (100 MHz, CDCl$_3$) δ = 161.4, 159.8, 149.3, 144.4, 143.0, 136.7, 129.8, 129.2, 123.2, 121.8, 120.4 – 113.5 (m), 120.2, 113.8, 112.2, 108.0, 55.3, 46.1, 35.5 (t, J = 20.2 Hz).

^{19}F NMR (376 MHz, CDCl$_3$) δ = -98.33 (d, J = 8.3 Hz, 2F), -113.67 (ddd, J = 104.0, 27.1, 10.4 Hz, 2F).

IR (film): 589, 639, 699, 720, 749, 899, 917, 974, 996, 1045, 1066, 1107, 1161, 1209, 1259, 1341, 1394, 1433, 1455, 1472, 1488, 1525, 1571, 1589, 2837, 2941, 3009 cm^{-1}

HRMS (+APCI-FTMS) calcd for $C_{19}H_{17}F_4N_3O$ $[M+H]^+$ m/z = 380.1381; found: 380.1375.

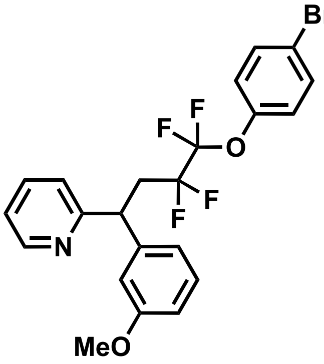

2-(4-(4-bromophenoxy)-3,3,4,4-tetrafluoro-1-(3-methoxyphenyl)butyl)pyridine (4z): Compound **4z** was synthesized following the general procedure 1 (standard-scale), using 1-bromo-4-(2-bromo-1,1,2,2-tetrafluoroethoxy)benzene (105.6 mg, 0.3 mmol), 2-Vinylpyridine (21.7 mg, 0.2 mmol) and 3-methoxyphenylmagnesium bromide (0.3 mL, 1.0 M solution in THF, 0..3 mmol). The product **4z** was obtained as a yellow oil (56.2 mg, 58% yield) after purification by column chromatography on silica gel with hexane/EtOAc (8:2).

^{1}H NMR (400 MHz, $CDCl_3$) δ = 8.63 (ddd, J = 4.9, 1.8, 0.9 Hz, 1H), 7.60 (td, J = 7.6, 1.8 Hz, 1H), 7.51 (d, *J* = 9.1 Hz), 2H), 7.30 – 7.23 (m, 2H), 7.16 (ddd, J = 7.6, 4.9, 1.2 Hz, 1H), 7.08 (d, J = 8.9 Hz, 2H), 7.04 – 6.98 (m, 2H), 6.79 (ddd, J = 8.2, 2.6, 0.8 Hz, 1H), 4.57 (t, *J* = 5.8 Hz, 1H), 3.82 (s, 3H), 3.64 – 3.48 (m, 1H), 2.97 – 2.77 (m, 1H).

^{13}C NMR (100 MHz, $CDCl_3$) *δ* = 161.6, 159.9, 149.3, 148.2, 144.7, 136.7, 132.8, 129.8, 123.7, 123.4, 121.9, 120.3, 119.7, 119.5 – 115.0 (m), 113.9, 112.2, 55.3, 46.0, 35.7 (t, *J* = 20.6 Hz).

^{19}F NMR (376 MHz, $CDCl_3$) δ = -88.42 (s, 2F), -116.36 (dddd, *J* = 154.7, 27.1, 11.4, 3.5 Hz, 2F).

IR (film): 624, 654, 698, 719, 747, 766, 783, 825, 853, 942, 992, 1012, 1044, 1068, 1093, 1107, 1182, 1223, 1260, 1310, 1333, 1434, 1455, 1484, 1509, 1571, 1589, 2836, 2954 cm^{-1}

HRMS (+APCI-FTMS) calcd for $C_{22}H_{18}BrF_4NO_2$ $[M+H]^+$ m/z = 484.0530; found: 484.0527.

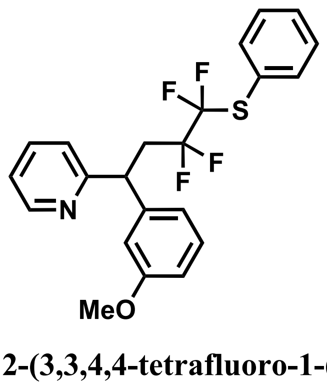

2-(3,3,4,4-tetrafluoro-1-(3-methoxyphenyl)-4-(phenylthio)butyl)pyridine (4z-a): Compound **4z-a** was synthesized following the general procedure 1 (standard-scale), using (2-Bromo-1,1,2,2-tetrafluoro-ethyl)sulfanylbenzene (86.7 mg, 0.3 mmol), 2-Vinylpyridine (21.7 mg, 0.2

mmol) and 3-methoxyphenylmagnesium bromide (0.3 mL, 1.0 M solution in THF, 0..3 mmol). The product **4z-a** was obtained as a pale yellow oil (49.2 mg, 58% yield) after purification by column chromatography on silica gel with hexane/EtOAc (8:2).

^{1}H NMR (400 MHz, CDCl$_3$) δ = 8.59 (ddd, *J* = 4.9, 1.9, 0.9 Hz, 1H), 7.62 (dd, *J* = 8.3, 1.4 Hz, 2H), 7.56 (td, *J* = 7.7, 1.9 Hz, 1H), 7.49 – 7.41 (m, 1H), 7.41 – 7.34 (m, 2H), 7.24 – 7.17 (m, 2H), 7.11 (ddd, *J* = 7.5, 4.9, 1.1 Hz, 1H), 6.99 – 6.91 (m, 2H), 6.75 (ddd, *J* = 8.3, 2.6, 1.1 Hz, 1H), 4.49 (dd, *J* = 7.8, 5.7 Hz, 1H), 3.78 (s, 3H), 3.57 – 3.40 (m, 1H), 2.87 – 2.71 (m, 1H).

^{13}C NMR (100 MHz, CDCl$_3$) *δ* = 161.6, 159.9, 149.3, 144.7, 137.3, 136.7, 130.5, 129.8, 129.3, 124.5 – 116.6 (m), 124.2, 123.3, 121.8, 120.3, 113.9, 112.2, 55.3, 46.2, 35.8 (t, *J* = 20.9 Hz).

^{19}F NMR (376 MHz, CDCl$_3$) δ = -88.57 (s, 2F), -110.57 to -110.96 (m, 2F).

IR (film): 639, 690, 698, 719, 746, 771, 878, 923, 996, 1048, 1065, 1149, 1221, 1258, 1311, 1371, 1433, 1454, 1473, 1487, 1570, 1589, 2836, 2942, 3008 cm^{-1}

HRMS (+APCI-FTMS) calcd for $C_{22}H_{19}F_4NOS$ [M+H]$^+$ m/z = 422.1196; found: 422.1191.

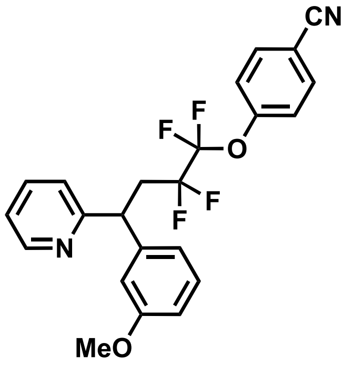

4-(1,1,2,2-tetrafluoro-4-(3-methoxyphenyl)-4-(pyridin-2-yl)butoxy)benzonitrile (4z-b): Compound **4z-b** was synthesized following the general procedure 1 (standard-scale), using 4-(2-bromo-1,1,2,2-tetrafluoroethoxy)benzonitrile (89.4 mg, 0.3 mmol), 2-Vinylpyridine (21.7 mg, 0.2 mmol) and 3-methoxyphenylmagnesium bromide (0.3 mL, 1.0 M solution in THF, 0..3 mmol). The product **4z-b** was obtained as a pale yellow oil (60.1 mg, 70% yield) after purification by column chromatography on silica gel with hexane/EtOAc (8:2).

^{1}H NMR (400 MHz, CDCl$_3$) δ = 8.61 (d, *J* = 4.9 Hz, 1H), 7.69 (d, *J* =8.9 Hz, 2H), 7.59 (td, *J* = 7.7, 1.9 Hz, 1H), 7.33 – 7.27 (m, 2H), 7.27 – 7.19 (m, 2H), 7.17 – 7.10 (m, 1H), 7.02 – 6.94 (m, 2H), 6.97 (t, *J* = 2.1 Hz, 1H), 6.77 (dd, *J* = 8.2, 2.6 Hz, 1H), 4.54 (t, *J* = 6.7 Hz, 1H), 3.79 (s, 3H), 3.65 – 3.45 (m, 1H), 2.95 – 2.76 (m, 1H).

^{13}C NMR (100 MHz, CDCl$_3$) *δ* = 161.4, 159.9, 152.6, 149.3, 144.5, 136.8, 134.0, 129.8, 123.4, 122.2, 121.9, 120.2, 118.0, 114.0, 112.1, 110.4, 55.3, 46.0, 35.6 (t, *J* = 20.6 Hz).

^{19}F NMR (376 MHz, CDCl$_3$) δ = -88.42 (s, 2F), -116.36 (ddd, *J* = 198.4, 28.1, 9.4 Hz, 2F).

IR (film): 623, 654, 699, 749, 770, 842, 865, 945, 993, 1019, 1044, 1078, 1109, 1167, 1205, 1224, 1260, 1308, 1335, 1434, 1455, 1472, 1489, 1499, 1571, 1588, 2232, 2838, 2956 cm^{-1}

HRMS (+APCI-FTMS) calcd for $C_{23}H_{18}F_4N_2O_2$ [M+H]$^+$ m/z = 431.1377; found: 431.1374.

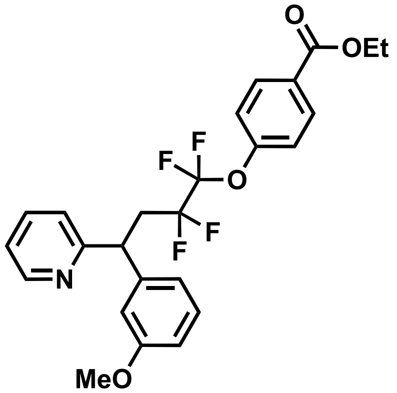

Ethyl 4-(1,1,2,2-tetrafluoro-4-(3-methoxyphenyl)-4-(pyridin-2-yl)butoxy)benzoate (4z-c): Compound **4z-d** was synthesized following the general procedure 1 (standard-scale), using Ethyl 4-(2-bromo-1,1,2,2-tetrafluoroethoxy)benzoate (103.5 mg, 0.3 mmol), 2-Vinylpyridine (21.7 mg, 0.2 mmol) and 3-methoxyphenylmagnesium bromide (0.3 mL, 1.0 M solution in THF, 0.3 mmol). The product **4z-d** was obtained as a clear oil (57.1 mg, 60% yield) after purification by column chromatography on silica gel with hexane/EtOAc (8:2).

^{1}H NMR (400 MHz, CDCl$_3$) δ = 8.60 (d, 1H), 8.05 (d, *J* = 8.9 Hz, 2H), 7.57 (td, *J* = 7.6, 1.8 Hz, 1H), 7.26 – 7.19 (m, 4H), 7.12 (ddd, *J* = 7.5, 4.9, 1.3 Hz, 1H), 7.03 – 6.93 (m, 2H), 6.76 (ddd, *J* = 8.3, 2.6, 1.0 Hz, 1H), 4.54 (dd, *J* = 7.6, 5.8 Hz, 1H), 4.38 (q, *J* = 7.1 Hz, 2H), 3.78 (s, 3H), 3.62 – 3.46 (m, 1H), 2.94 – 2.77 (m 1H), 1.39 (t, *J* = 7.1 Hz, 3H).

^{13}C NMR (100 MHz, CDCl$_3$) *δ* = 165.8, 161.6, 159.9, 152.8, 149.3, 144.6, 136.7, 131.4, 129.8, 128.6, 123.4, 121.9, 121.3, 121.1 – 116.6 (m), 120.3, 113.9, 112.2, 61.3, 55.3, 46.1, 35.69 (t, *J* = 20.9 Hz), 14.4.

^{19}F NMR (376 MHz, CDCl$_3$) δ = -88.36 (s, 2F), -116.40 (ddd, *J* = 163.0, 27.4, 9.7 Hz, 2F).

IR (film): 655, 699, 719, 749, 768, 857, 945, 993, 1018, 1045, 1079, 1105, 1163, 1274, 1368, 1434, 1473, 1488, 1505, 1571, 1589, 1717, 2983 cm^{-1}

HRMS (+APCI-FTMS) calcd for $C_{25}H_{23}F_4NO_4$ $[M+H]^+$ m/z = 478.1636; found: 478.1629.

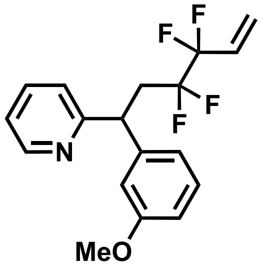

2-(3,3,4,4-tetrafluoro-1-(3-methoxyphenyl)hex-5-en-1-yl)pyridine (4z-d): Compound **4z-d** was synthesized following the general procedure 1 (standard-scale), using 4-bromo-3,3,4,4-tetrafluorobut-1-ene (62.8 mg, 0.30 mmol), 2-Vinylpyridine (21.7 mg, 0.20 mmol) and 3-methoxyphenylmagnesium bromide (0.42 mL, 1.0 M solution in THF, 0.42 mmol). The product **4z-d** was obtained as a pale yellow oil (31.6 mg, 46% yield) after purification by column chromatography on silica gel with hexane/EtOAc (8:2).

^{1}H NMR (400 MHz, CDCl$_3$) δ = 8.58 (d, *J* = 4.8 Hz, 1H), 7.55 (td, *J* = 7.7, 1.9 Hz, 1H), 7.23 – 7.17 (m, 2H), 7.10 (ddd, *J* = 7.5, 4.9, 1.2 Hz, 1H), 6.99 – 6.92 (m, 2H), 6.74 (ddd, *J* = 8.2, 2.6, 1.0 Hz), 6.01 – 5.81 (m, 2H), 5.65 (d, *J* = 10.7 Hz, 1H), 4.49 (dd, *J* = 7.7, 5.7 Hz, 1H), 3.78 (s, 3H), 3.45 – 3.30 (m, 1H), 2.80 – 2.65 (m, 1H).

^{13}C NMR (100 MHz, CDCl$_3$) *δ* = 161.8, 159.8, 149.3, 144.8, 136.7, 131.5, 129.7, 126.6 (t, *J* = 24.2 Hz), 126.4 – 112.4 (m), 124.0 (t, *J* = 9.4 Hz), 121.8, 120.3, 113.9, 112.1, 55.3, 46.1, 35.0 (t, *J* = 20.9 Hz).

^{19}F NMR (376 MHz, CDCl$_3$) δ = -114.25 (ddd, *J* = 112.4, 27.7, 10.1 Hz, 2F), -115.38 (d, *J* = 11.8 Hz, 2F).

IR (film): 699, 714, 748, 773, 781, 959, 995, 1009, 1038, 1073, 1106, 1149, 1188, 1228, 1259, 1420, 1434, 1455, 1472, 1488, 1571, 1588, 2837, 2941, 3008, 3054 cm^{-1}

HRMS (+APCI-FTMS) calcd for $C_{18}H_{17}F_4NO$ $[M+H]^+$ m/z = 340.1319; found: 340.1309.

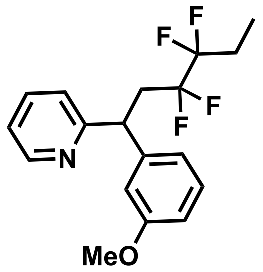

2-(3,3,4,4-tetrafluoro-1-(3-methoxyphenyl)hexyl)pyridine (4z-e): Compound **4z-e** was synthesized following the general procedure 1 (standard-scale), using 1-bromo-1,1,2,2-tetrafluorobutane (81.5 mg, 0.39 mmol), 2-Vinylpyridine (27.4 mg, 0.26 mmol) and 3-methoxyphenylmagnesium bromide (0.39 mL, 1.0 M solution in THF, 0.39 mmol). The product **4z-e** was obtained as a pale yellow oil (30.1 mg, 34% yield) after purification by column chromatography on silica gel with hexane/EtOAc (8:2).

^{1}H NMR (400 MHz, CDCl$_3$) δ = 1H NMR (400 MHz, CDCl3) δ 8.58 (d, *J* = 4.9 Hz, 1H), 7.55 (td, *J* = 7.7, 1.9 Hz, 1H), 7.24 – 7.16 (m, 2H), 7.10 (ddd, *J* = 7.5, 4.8, 1.2 Hz, 1H), 7.00 – 6.92 (m, 2H), 6.74 (ddd, *J* = 8.3, 2.5, 1.0 Hz, 1H), 4.49 (dd, *J* = 7.9, 5.8 Hz, 1H), 3.77 (s, 3H), 3.44 – 3.28 (m, 1H), 2.81 – 2.65 (m, 1H), 2.06 – 1.91 (m, 2H), 1.06 (t, *J* = 7.6 Hz, 3H).

^{13}C NMR (100 MHz, CDCl$_3$) *δ* = 162.0, 159.8, 149.3, 144.9, 136.6, 129.7, 123.2, 122.1 – 116.8 (m), 121.7, 120.3, 119.3 (td, *J* = 35.4 Hz, 5.8 Hz) , 113.9, 112.1, 55.3, 46.1, 34.8 (t, *J* = 21.3 Hz), 23.5 (t, *J* = 23.8 Hz), 5.0 (t, *J* = 5.0 Hz).

^{19}F NMR (376 MHz, CDCl$_3$) δ = -113.84 to -114.32 (m, 2F), -117.64 (tt, *J* = 17.3, 7.6 Hz, 2F).

IR (film): 515, 698, 747, 923, 950, 1002, 1048, 1071, 1107, 1152, 1172, 1260, 1434, 1468, 1488, 1589, 2838, 2852, 2924, 2952, 2994, 3054 cm^{-1}

HRMS (+APCI-FTMS) calcd for $C_{18}H_{19}F_4NO$ $[M+H]^+$ m/z = 342.1476; found: 342.1467.

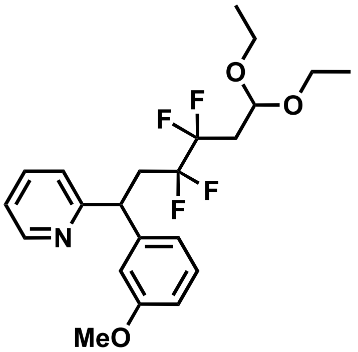

2-(6,6-diethoxy-3,3,4,4-tetrafluoro-1-(3-methoxyphenyl)hexyl)pyridine (4z-f): Compound **4z-f** was synthesized following the general procedure 1 (standard-scale), using 1-bromo-4,4-diethoxy-1,1,2,2-tetrafluorobutane (89.1 mg, 0.3 mmol), 2-Vinylpyridine (21.7 mg, 0.2 mmol) and 3-methoxyphenylmagnesium bromide (0.3 mL, 1.0 M solution in THF, 0.3 mmol). The product **4z-f** was obtained as a pale yellow oil (50.3 mg, 52% yield) after purification by column chromatography on silica gel with hexane/EtOAc (8:2).

^{1}H NMR (400 MHz, CDCl$_3$) δ = 8.57 (d, J = 4.9 Hz, 1H), 7.55 (td, *J* = 7.7, 1.9 Hz, 1H), 7.22 – 7.16 (m, 2H), 7.10 (ddd, *J* = 7.5, 4.9, 1.2 Hz, 1H), 6.98 – 6.91 (m, 2H), 6.74 (ddd, *J* = 8.3, 2.6, 1.1 Hz, 1H), 4.88 (t, *J* = 5.2 Hz, 1H), 4.48 (dd, *J* = 7.8, 5.8 Hz, 1H), 3.77 (s, 3H), 3.68 -3.59 (m, 2H), 3.56 – 3.47 (m, 2H), 3.44 – 3.28 (m, 1H), 2.80 – 2.65 (m, 1H), 2.34 (td, *J* = 19.1, 5.2, 2H), 1.20 (t, *J* = 7.0 Hz, 6H).

^{13}C NMR (100 MHz, CDCl$_3$) *δ* = 161.9, 159.8, 149.3, 144.8, 136.6, 129.7, 123.2, 121.7, 120.2, 113.8, 112.1, 97.5, 61.7, 55.3, 46.1, 34.8 (t, *J* = 21.3 Hz), 34.5 (t, *J* = 10.5 Hz), 15.25.

^{19}F NMR (376 MHz, CDCl$_3$) δ = -114.20 (dddd, *J* = 92.9, 26.4, 10.1, 5.2 Hz, 2F), -114.52 (t, *J* = 18.4 Hz, 2F).

IR (film): 530, 699, 748, 949, 995, 1049, 1068, 1094, 1147, 1175, 1260, 1434, 1472, 1488, 1590, 2929, 2977 cm^{-1}

HRMS (+APCI-FTMS) calcd for $C_{22}H_{27}F_4NO_3$ $[M+H]^+$ m/z = 430.2000; found: 430.1990.

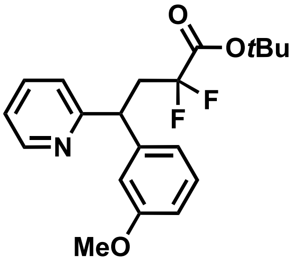

tert-butyl 2,2-difluoro-4-(3-methoxyphenyl)-4-(pyridin-2-yl)butanoate (4z-g): Compound **4z-g** was synthesized following the general procedure 1 (standard-scale), using Tert-Butyl 2-bromo,2,2-difluoroacete (68.41 mg, 0.3 mmol), 2-Vinylpyridine (21.7 mg, 0.2 mmol) and 3-methoxyphenylmagnesium bromide (0.3 mL, 1.0 M solution in THF, 0.3 mmol). The product **4z-g** was obtained as a pale yellow oil (25.2 mg, 35% yield) after purification by column chromatography on silica gel with hexane/EtOAc (8:2).

^{1}H NMR (400 MHz, CDCl$_3$) δ = 8.57 (d, *J* = 4.9 Hz, 1H), 7.54 (td, *J* = 7.7, 1.9 Hz, 1H), 7.23 – 7.14 (m, 2H), 7.10 (ddd, *J* = 7.5, 4.9, 1.1 Hz, 1H), 6.94 – 6.88 (m, 2H), 6.73 (ddd, *J* = 8.2, 2.6, 0.9 Hz, 1H), 4.38 (t, *J* = 6.7 Hz, 1H), 3.76 (s, 3H), 3.41 – 3.26 (m, 1H), 2.86 – 2.70 (m, 1H), 1.41 (s, 9H).

^{13}C NMR (100 MHz, CDCl$_3$) *δ* = 163.0 (t, *J* = 31.8 Hz), 161.6, 159.7, 149.2, 144.5, 136.6, 129.6, 123.3, 121.7, 120.3, 115. 7 (t, *J* = 249.4 Hz), 113.8, 112.1, 84.3, 55.2, 46.7, 39.0 (t, *J* = 22.7 Hz), 27.6.

^{19}F NMR (376 MHz, CDCl$_3$) δ = -104.13 (ddd, *J* = 142.2, 18.4, 14.6 Hz, 2F).

IR (film): 670, 700, 749, 838, 944, 995, 1050, 1077, 1108, 1158, 1196, 1218, 1260, 1310, 1370, 1396, 1434, 1473, 1488, 1571, 1589, 1757, 2935, 2982 cm^{-1}

HRMS (+ESI-FTMS) calcd for $C_{20}H_{23}F_2NO_3$ $[M+H]^+$ m/z = 364.1719; found: 364.1712.

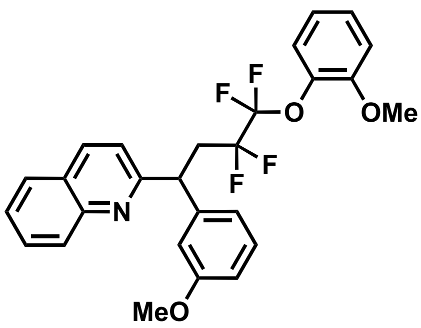

2-(3,3,4,4-tetrafluoro-4-(2-methoxyphenoxy)-1-(3-methoxyphenyl)butyl)quinoline (5a): Compound **5a** was synthesized following the general procedure 1 (standard-scale), using 2-(2-bromo-1,1,2,2-tetrafluoroethoxy) anisole (109.1 mg, 0.36 mmol), 2-Vinylquinoline (37.1 mg, 0.24 mmol) and 3-methoxyphenylmagnesium bromide (0.36 mL, 1.0 M solution in THF, 0.36 mmol). The product **5a** was obtained as a pale yellow oil (32.9 mg, 28% yield) after purification by column chromatography on silica gel with hexane/EtOAc (8:2).

^{1}H NMR (400 MHz, CDCl$_3$) δ = 8.14 (d, *J* = 8.5 Hz, 1H), 8.01 (d, *J* = 8.5 Hz, 1H), 7.75 (dd, *J* = 8.0, 1.8 Hz, 1H), 7.70 (ddd, *J* = 8.4, 6.9, 1.5 Hz, 1H), 7.50 (ddd, *J* = 8.1, 6.9, 1.3 Hz, 1H), 7.30 (d, *J* = 8.5 Hz, 1H), 7.24 – 7.19 (m, 3H), 7.06 – 7.04 (m, 2H), 6.97 (dd, *J* = 8.6, 1.5 Hz, 1H), 6.92 (td, *J* = 8.0, 1.5 Hz, 1H), 6.78 – 6.72 (m, 1H), 4.79 (t, *J* = 6.7 Hz, 1H), 3.90 – 3.79 (m, 4H), 3.76 (s, 3H), 3.12 – 2.97 (m, 1H).

^{13}C NMR (100 MHz, CDCl$_3$) *δ* = 161.7, 159.9, 152.6, 147.8, 144.7, 138.1, 136.5, 129.7, 129.6, 129.4, 127.6, 127.5, 127.1, 126.2, 124.0, 121.8, 120.7, 121.5 – 114.9 (m), 114.3, 113.0, 112.1, 56.2, 55.3, 46.7, 35.7 (t, *J* = 20.6 Hz).

^{19}F NMR (376 MHz, CDCl$_3$) δ = -88.38 (t, *J* = 6.9 Hz, 2F), -114.94 to -115.93 (m, 2F).

IR (film): 623, 710, 724, 751, 768, 834, 971, 994, 1027, 1044, 1108, 1171, 1188, 1219, 1260, 1282, 1304, 1438, 1457, 1502, 1567, 1598, 2839, 2932 cm^{-1}

HRMS (+ESI-FTMS) calcd for $C_{27}H_{23}F_4NO_3$ $[M+H]^+$ m/z = 486.1687; found: 486.1681

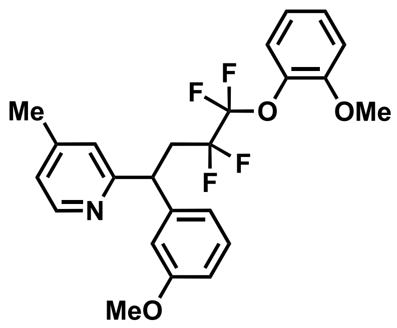

4-methyl-2-(3,3,4,4-tetrafluoro-4-(2-methoxyphenoxy)-1-(3-methoxyphenyl)butyl)pyridine (5b): Compound **5b** was synthesized following the general procedure 1 (standard-scale), using 2-(2-bromo-1,1,2,2-tetrafluoroethoxy) anisole (90.91 mg, 0.3 mmol), 4-Methyl-2-vinylpyridine (23.8 mg, 0.2 mmol) and 3-methoxyphenylmagnesium bromide (0.3 mL, 1.0 M solution in THF, 0.3 mmol). The product **5b** was obtained as a clear oil (60.3 mg, 67% yield) after purification by column chromatography on silica gel with hexane/EtOAc (8:2).

^{1}H NMR (400 MHz, CDCl$_3$) δ = 8.45 (d, J = 5.1 Hz, 1H), 7.24 – 7.18 (m, 3H), 7.06 – 6.89 (m, 6H), 6.75 (ddd, J = 8.2, 2.6, 1.0 Hz, 1H), 4.55 (dd, J = 7.8, 5.8 Hz, 1H), 3.81 (s, 3H), 3.78 (s, 3H), 3.61 – 3.46 (m, 1H), 3.02 – 2.86 (m, 1H), 2.28 (s, 3H).

^{13}C NMR (100 MHz, CDCl$_3$) δ = 161.7, 159.8, 152.6, 149.1, 147.7, 145.0, 138.0, 129.7, 127.5, 126.6 – 112.9 (m), 124.2, 124.0, 122.8, 120.7, 120.3, 113.9, 112.9, 112.0, 56.1, 55.3, 46.0, 35.9 (t, J = 20.2 Hz), 21.1.

^{19}F NMR (376 MHz, CDCl$_3$) δ = -88.44 (d, J = 4.9 Hz, 2F), -115.85 (dddd, J = 20.8, 15.9, 8.3, 4.2 Hz, 2F).

IR (film): 483, 519, 561, 606, 663, 699, 748, 767, 831, 855, 950, 993, 1026, 1042, 1105, 1169, 1187, 1218, 1260, 1282, 1304, 1438, 1456, 1488, 1502, 1562, 1585, 1602, 2839, 2946 cm^{-1}

HRMS (+APCI-FTMS) calcd for $C_{24}H_{23}F_4NO_3$ $[M+H]^+$ m/z = 450.1687; found: 450.1678.

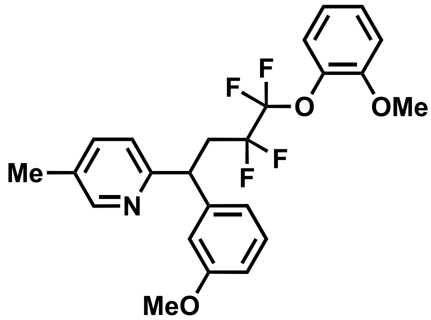

5-methyl-2-(3,3,4,4-tetrafluoro-4-(2-methoxyphenoxy)-1-(3-methoxyphenyl)butyl)pyridine (5c): Compound **5c** was synthesized following the general procedure 1 (standard-scale), using 2-(2-bromo-1,1,2,2-tetrafluoroethoxy) anisole (90.91 mg, 0.3 mmol), 5-Methyl-2-vinylpyridine (23.8 mg, 0.2 mmol) and 3-methoxyphenylmagnesium

bromide (0.3 mL, 1.0 M solution in THF, 0.3 mmol). The product **5c** was obtained as a clear oil (52.7 mg, 59% yield) after purification by column chromatography on silica gel with hexane/EtOAc (8:2).

^{1}H NMR (400 MHz, CDCl$_3$) δ = 8.42 (d, *J* = 2.4 Hz, 1H), 7.38 (dd, *J* = 7.9, 1.6 Hz, 1H), 7.24 – 7.16 (m, 3H), 7.12 (d, *J* = 7.9 Hz, 1H), 7.02 – 6.94 (m, 3H), 6.91 (td, *J* = 7.6, 1.5 Hz, 1H), 6.74 (ddd, *J* = 8.3, 2.5, 1.0 Hz, 1H), 4.57 (dd, *J* = 8.0, 5.8 Hz, 1H), 3.81 (s, 3H), 3.78 (s, 3H), 3.60 – 3.45 (m, 1H), 3.01 – 2.86 (m, 1H), 2.28 (s, 3H).

^{13}C NMR (100 MHz, CDCl$_3$) *δ* = 159.8, 159.0, 152.6, 149.7, 145.1, 138.0, 137.2, 131.0, 129.7, 127.5, 124.0, 122.7, 120.7, 120.3, 119.8 – 114.8 (m), 113.9, 112.9, 112.0, 56.1, 55.3, 45.7, 35.9 (t, *J* = 20.6 Hz), 18.2.

^{19}F NMR (376 MHz, CDCl$_3$) δ = -88.45 (dt, *J* = 8.3, 4.2 Hz, 2F), -115.81 (tt, *J* = 19.4, 3.5 Hz, 2F).

IR (film): 624, 665, 705, 747, 853, 901, 942, 992, 1029, 1043, 1106, 1169, 1187, 1217, 1260, 1282, 1304, 1383, 1437, 1456, 1485, 1502, 1598, 2839, 2957 cm^{-1}

HRMS (+APCI-FTMS) calcd for $C_{24}H_{23}F_4NO_3$ $[M+H]^+$ m/z = 450.1687; found: 450.1678.

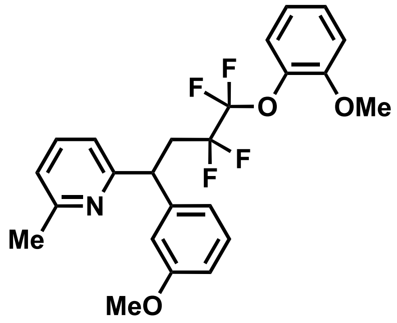

5-methyl-2-(3,3,4,4-tetrafluoro-4-(2-methoxyphenoxy)-1-(3-methoxyphenyl)butyl)pyridine (5d): Compound **5d** was synthesized following the general procedure 1 (standard-scale), using 2-(2-bromo-1,1,2,2-tetrafluoroethoxy) anisole (90.91 mg, 0.3 mmol), 2-Methyl-2-vinylpyridine (23.8 mg, 0.2 mmol) and 3-methoxyphenylmagnesium bromide (0.3 mL, 1.0 M solution in THF, 0.3 mmol). The product **5d** was obtained as a pale yellow oil (43.0 mg, 48% yield) after purification by column chromatography on silica gel with hexane/EtOAc (8:2).

^{1}H NMR (400 MHz, CDCl$_3$) δ = 7.44 (t, *J* = 7.7 Hz, 1H), 7.24 – 7.19 (m, 3H), 7.03 – 6.90 (m, 6H), 6.75 (ddd, *J* = 8.2, 2.5, 1.1 Hz, 1H), 4.56 (t, *J* = 6.8 Hz, 1H), 3.83 (s, 3H), 3.79 (s, 3H), 3.62 – 3.44 (m, 1H), 3.00 – 2.84 (m, 1H), 2.56 (s, 3H).

^{13}C NMR (100 MHz, CDCl$_3$) *δ* = 161.1, 159.8, 158.0, 152.6, 145.2, 138.1, 136.7, 129.6, 127.5, 124.0, 122.5 – 112.9 (m), 121.1, 120.7, 120.5, 119.9, 114.1, 113.0, 111.9, 56.2, 55.3, 46.1, 36.1 (t, *J* = 20.6 Hz), 24.8.

^{19}F NMR (376 MHz, CDCl$_3$) δ = -88.49 (q, *J* = 5.5 Hz, 2F), -115.73 (ddddd, *J* = 72.8, 15.9, 11.8, 7.6, 3.8 Hz, 2F).

IR (film): 609, 654, 670, 705, 746, 808, 854, 953, 989, 1026, 1043, 1104, 1169, 1186, 1218, 1260, 1282, 1302, 1439, 1455, 1502, 1575, 1590, 2839, 2958 cm^{-1}

HRMS (+ESI-FTMS) calcd for $C_{24}H_{23}F_4NO_3$ $[M+H]^+$ m/z = 450.1687; found: 450.1674.

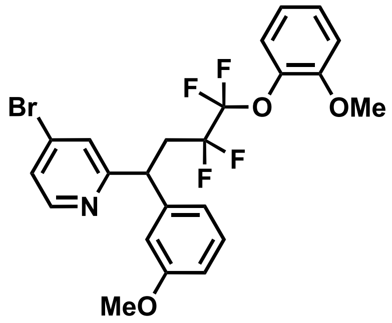

4-bromo-2-(3,3,4,4-tetrafluoro-4-(2-methoxyphenoxy)-1-(3-methoxyphenyl)butyl)pyridine (5e): Compound **5e** was synthesized following the general procedure 1 (standard-scale), using 2-(2-bromo-1,1,2,2-tetrafluoroethoxy) anisole (90.91 mg, 0.3 mmol), 4-Bromo-2-vinylpyridine (36.8 mg, 0.2 mmol) and 3-methoxyphenylmagnesium bromide (0.3 mL, 1.0 M solution in THF, 0.3 mmol). The product **5e** was obtained as a clear oil (34.2 mg, 33% yield) after purification by column chromatography on silica gel with hexane/EtOAc (8:2).

^{1}H NMR (400 MHz, CDCl$_3$) δ = 8.41 (d, *J* = 5.3 Hz, 1H), 7.41 (d, *J* = 1.9 Hz, 1H), 7.29 (dd, *J* = 5.3, 1.8 Hz, 1H), 7.25 – 7.19 (m, 3H), 7.00 – 6.95 (m, 3H), 6.92 (td, *J* = 7.8, 1.5 Hz, 1H), 6.77 (ddd, *J* = 8.3, 2.5, 1.0 Hz, 1H), 4.54 (dd, *J* = 8.1, 5.5 Hz, 1H), 3.82 (s, 3H), 3.79 (s, 3H), 3.61 – 3.46 (m, 1H), 2.98 – 2.83 (m, 1H).

^{13}C NMR (100 MHz, CDCl$_3$) *δ* = 163.4, 159.9, 152.6, 150.1, 144.1, 137.9, 133.2, 129.9, 127.6, 126.6, 125.2, 124.0, 120.7, 120.3, 119.6 – 101.7 (m), 114.0, 112.9, 112.3, 56.1, 55.4, 46.0, 35.9 (t, *J* =20.6 Hz).

^{19}F NMR (376 MHz, CDCl$_3$) δ = -88.42 (dt, *J* = 11.8, 4.2 Hz, 2F), -115.63 (ddq, *J* = 20.1, 16.6, 4.2 Hz, 2F).

IR (film): 607, 699, 747, 827, 853, 875, 945, 993, 1043, 1106, 1169, 1186, 1219, 1260, 1282, 1303, 1392, 1438, 1465, 1502, 1555, 1568, 1585, 1600, 2838, 2943 cm^{-1}

HRMS (+ESI-FTMS) calcd for $C_{23}H_{20}BrF_4NO_3$ $[M+H]^+$ m/z = 514.0635; found: 514.0626.

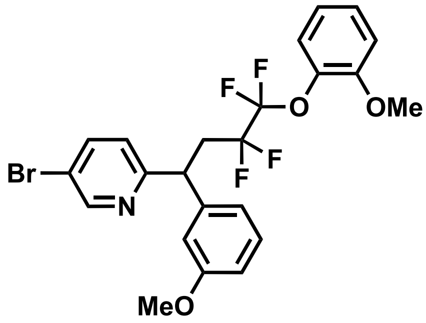

5-bromo-2-(3,3,4,4-tetrafluoro-4-(2-methoxyphenoxy)-1-(3-methoxyphenyl)butyl)pyridine (5f): Compound **5f** was synthesized following the general procedure 1 (standard-scale), using 2-(2-bromo-1,1,2,2-tetrafluoroethoxy) anisole (90.91 mg, 0.3 mmol), 5-Bromo-2-vinylpyridine (36.8 mg, 0.2 mmol) and 3-methoxyphenylmagnesium bromide (0.3 mL, 1.0 M solution in THF, 0.3 mmol). The product **5f** was obtained as a clear oil (42.7 mg, 42% yield) after purification by column chromatography on silica gel with hexane/EtOAc (8:2).

^{1}H NMR (400 MHz, $CDCl_3$) δ = 8.65 (d, *J* = 3.1 Hz, 1H), 7.69 (dd, *J* = 8.3, 2.4 Hz, 1H), 7.26 – 7.18 (m, 3H), 7.13 (d, *J* = 8.3 Hz, 1H), 6.99 – 6.89 (m, 4H), 6.76 (ddd, *J* = 8.2, 2.6, 1.0 Hz, 1H), 4.56 (dd, *J* = 8.3, 5.2 Hz, 1H), 3.82 (s, 3H), 3.78 (s, 3H), 3.61 – 3.44 (m, 1H), 2.96 – 2.80 (m, 1H).

^{13}C NMR (100 MHz, $CDCl_3$) *δ* = 160.5, 159.9, 152.6, 150.4, 144.3, 139.2, 137.9, 129.9, 127.6, 124.6, 124.0, 120.7, 120.2, 118.8, 117.8 – 115.1 (m), 113.9, 112.9, 112.2, 56.1, 55.3, 45.6, 35.90 (t, *J* = 20.6 Hz).

^{19}F NMR (376 MHz, $CDCl_3$) δ = -88.46 (d, *J* = 4.2 Hz, 2F), -115.68 (dtt, *J* = 25.0, 16.0, 4.2 Hz, 2F).

IR (film): 608, 670, 700, 748, 766, 841, 941, 1007, 1043, 1095, 1169, 1187, 1219, 1260, 1282, 1304, 1373, 1438, 1465, 1502, 1585, 1560, 2838, 2958 cm^{-1}

HRMS (+ESI-FTMS) calcd for $C_{23}H_{20}BrF_4NO_3$ $[M+H]^+$ m/z = 514.0635; found: 514.0626.

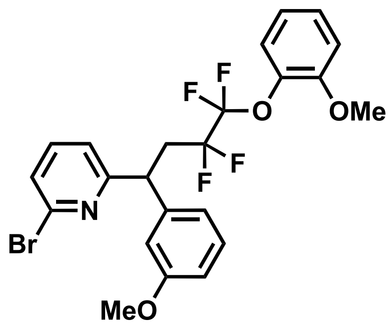

2-bromo-6-(3,3,4,4-tetrafluoro-4-(2-methoxyphenoxy)-1-(3-methoxyphenyl)butyl)pyridine (5g): Compound **5g** was synthesized following the general procedure 1 (standard-scale), using 2-(2-bromo-1,1,2,2-tetrafluoroethoxy) anisole (90.91 mg, 0.3 mmol), 2-Bromo-2-vinylpyridine (23.8 mg, 0.2 mmol) and 3-methoxyphenylmagnesium bromide (0.3 mL, 1.0 M solution in THF, 0.3 mmol). The product **5g** was obtained as a pale orange oil (26.8 mg, 26% yield) after purification by column chromatography on silica gel with hexane/EtOAc (8:2).

^{1}H NMR (400 MHz, $CDCl_3$) δ = 7.41 (t, *J* = 7.7 Hz, 1H), 7.30 (dd, *J* = 7.9, 1.0 Hz, 1H), 7.26 – 7.19 (m, 3H), 7.16 (dd, *J* = 7.5, 1.0 Hz, 1H), 7.03 – 6.95 (m, 3H), 6.92 (td, *J* = 7.6, 1.5 Hz, 1H), 6.77 (ddd, *J* = 8.3, 2.4, 1.1 Hz, 1H), 4.54 (dd, *J* = 7.8, 6.0 Hz, 1H), 3.85 (s, 3H), 3.80 (s, 3H), 3.57 – 3.39 (m, 1H), 3.00 – 2.84 (m, 1H).

^{13}C NMR (100 MHz, CDCl$_3$) δ = 163.4, 159.9, 152.6, 144.0, 141.7, 138.9, 138.0, 129.8, 127.6, 126.2, 123.9, 122.0, 120.7, 120.4, 119.7 – 114.7 (m), 113.9, 113.0, 112.4, 56.2, 55.3, 45.9, 36.1 (t, *J* = 20.6 Hz).

^{19}F NMR (376 MHz, CDCl$_3$) δ = -88.47 (s, 2F), -115.64 (tdt, *J* = 21.5, 13.9, 4.2 Hz, 2F).

IR (film): 607, 676, 700, 747, 779, 803, 854, 908, 946, 987, 1044, 1108, 1123, 1170, 1189, 1261, 1282, 1302, 1433, 1456, 1503, 1555, 1581, 1601, 2838, 2960 cm^{-1}

HRMS (+ESI-FTMS) calcd for $C_{23}H_{20}BrF_4NO_3$ $[M+H]^+$ m/z = 514.0635; found: 514.0626.

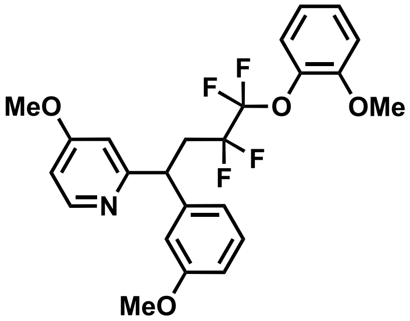

4-methoxy-2-(3,3,4,4-tetrafluoro-4-(2-methoxyphenoxy)-1-(3-methoxyphenyl)butyl)pyridine (5h): Compound **5h** was synthesized following the general procedure 1 (standard-scale), using 2-(2-bromo-1,1,2,2-tetrafluoroethoxy) anisole (90.91 mg, 0.3 mmol), 4-Methoxy-2-vinylpyridine (27.03 mg, 0.2 mmol) and 3-methoxyphenylmagnesium bromide (0.3 mL, 1.0 M solution in THF, 0.3 mmol). The product **5h** was obtained as a pale yellow oil (65.7 mg, 71% yield) after purification by column chromatography on silica gel with hexane/EtOAc (8:2).

^{1}H NMR (400 MHz, CDCl$_3$) δ = 8.40 (d, *J* = 5.7 Hz, 1H), 7.23 – 7.15 (m, 3H), 7.03 – 6.97 (m, 2H), 6.93 (dd, *J* = 8.8, 1.6 Hz, 1H), 6.88 (td, *J* = 7.6, 1.5 Hz, 1H), 6.77 – 6.69 (m, 2H), 6.62 (dd, *J* = 5.7, 2.5 Hz, 1H), 4.56 – 4.47 (m, 1H), 3.78 (s, 3H), 3.76 – 3.72 (m, 6H), 3.60 – 3.46 (m, 1H), 3.00 – 2.80 (m, 1H).

^{13}C NMR (100 MHz, CDCl$_3$) δ = 166.1, 163.5, 159.8, 152.6, 150.6, 144.8, 137.9, 129.7, 127.5, 124.0, 120.6, 120.3, 119.8 – 114.8 (m), 113.9, 112.9, 112.0, 109.3, 107.9, 56.0, 55.2, 55.1, 46.3 (t, *J* = 2.5 Hz), 35.89 (t, *J* = 20.6 Hz).

^{19}F NMR (376 MHz, CDCl$_3$) δ = -88.39 (d, *J* = 4.2 Hz, 2F), -115.69 to -115.86 (m, 2F).

IR (film): 701, 752, 820, 855, 953, 991, 1003, 1041, 1109, 1171, 1189, 1262, 1283, 1304, 1440, 1458, 1488, 1503, 1569, 1594, 2839, 2944 cm^{-1}

HRMS (+ESI-FTMS) calcd for $C_{24}H_{23}F_4NO_4$ $[M+H]^+$ m/z = 466.1636; found: 466.1620.

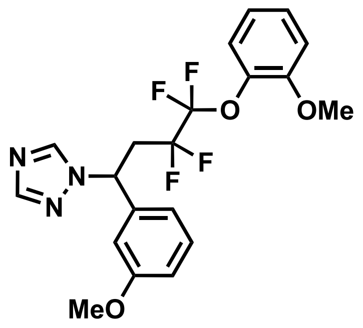

1-(3,3,4,4-tetrafluoro-4-(2-methoxyphenoxy)-1-(3-methoxyphenyl)butyl)-1*H*-1,2,4-triazole (5i): Compound **5i** was synthesized following a modified general procedure 1, using 2-(2-bromo-1,1,2,2-tetrafluoroethoxy) anisole (120.4 mg, 0.4 mmol), 1-Vinyl-1,2,4-triazole (19.02 mg, 0.2 mmol) and 3-methoxyphenylmagnesium bromide (0.4 mL, 1.0 M solution in THF, 0.4 mmol). The product **5i** was obtained as a colorless oil (44.4 mg, 52% yield) after purification by column chromatography on silica gel with hexane/EtOAc (8:2).

^{1}H NMR (400 MHz, CDCl$_3$) δ = 8.16 (s, 1H), 8.01 (s, 1H), 7.31 (t, *J* = 7.9 Hz, 1H), 7.26 – 7.20 (m, 2H), 7.04 – 6.96 (m, 3H), 6.93 (td, *J* = 7.6, 1.5 Hz, 1H), 6.88 (ddd, *J* = 8.3, 2.5, 0.9 Hz, 1H), 5.96 (dd, *J* = 9.1, 4.5 Hz, 1H), 3.82 (s, 3H), 3.80 (s, 3H), 3.69 – 3.54 (m, 1H), 3.14 – 2.98 (m, 1H).

^{13}C NMR (100 MHz, CDCl$_3$) *δ* = 160.2, 152.4, 152.3, 143.3, 139.9, 137.6, 130.3, 127.8, 123.9, 120.8, 119.1, 119.8 – 114.8 (m), 114.1, 113.1, 112.8, 57.5, 56.1, 55.4, 36.9 (t, *J* = 21.3 Hz).

^{19}F NMR (376 MHz, CDCl$_3$) δ = -88.39 (t, *J* = 4.5 Hz, 2F), -115.88 to -116.78 (m, 2F).

IR (film): 500, 561, 607, 661, 680, 710, 727, 749, 855, 931, 957, 1007, 1043, 1105, 1137, 1169, 1190, 1261, 1340, 1439, 1457, 1501, 1587, 1602, 2840, 2962 cm^{-1}

HRMS (+ESI-FTMS) calcd for $C_{20}H_{19}F_4N_3O_3$ $[M+H]^+$ m/z = 426.1435; found: 426.1426.

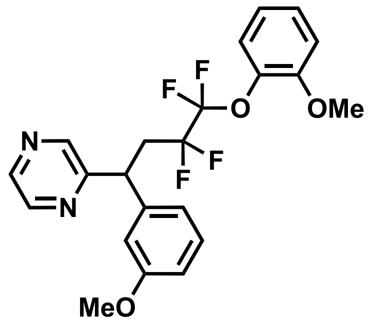

2-(3,3,4,4-tetrafluoro-4-(2-methoxyphenoxy)-1-(3-methoxyphenyl)butyl)pyrazine (5j): Compound **5j** was synthesized following a slightly modified general procedure 1 (standard-scale), using 2-(2-bromo-1,1,2,2-tetrafluoroethoxy) anisole (120.4 mg, 0.4 mmol), 2-Vinylpyrazine (21.2 mg, 0.2 mmol) and 3-methoxyphenylmagnesium bromide (0.8 mL, 1.0 M solution in THF, 0.8 mmol). The product **5j** was obtained as a yellow oil (18.3 mg, 21% yield) after purification by column chromatography on silica gel with hexane/EtOAc (8:2).

^{1}H NMR (400 MHz, CDCl$_3$) δ = 8.55 (s, 2H), 8.41 (d, *J* = 2.4 Hz, 1H), 7.26 – 7.17 (m, 3H), 7.03 – 6.95 (m, 3H), 6.92 (td, *J* = 7.6, 1.5 Hz, 1H), 6.77 (dd, *J* = 8.3, 3.6 Hz, 1H), 4.67 (dd, *J* = 8.8, 5.0 Hz, 1H), 3.82 (s, 3H), 3.79 (s, 3H), 3.62 – 3.46 (m, 1H), 3.01 – 2.85 (m, 1H).

^{13}C NMR (100 MHz, CDCl$_3$) *δ* = 160.0, 157.5, 152.5, 145.0, 144.1, 143.5, 142.9, 137.9, 130.0, 127.6, 124.0, 120.7, 120.5 – 115.0 (m), 120.2, 114.0, 112.9, 112.4, 56.1, 55.4, 43.6, 35.8 (t, *J* = 20.9 Hz).

^{19}F NMR (376 MHz, CDCl$_3$) δ = -88.39 (dd, *J* = 10.1, 5.2 Hz, 2F), -115.15 – -115.71 (m, 2F).

IR (film): 702, 750, 769, 854, 940, 993, 1019, 1044, 1108, 1140, 1171, 1189, 1262, 1282, 1303, 1406, 1439, 1457, 1503, 1526, 1586, 1601, 2839, 2946 cm^{-1}

HRMS (+ESI-FTMS) calcd for $C_{22}H_{20}F_4N_2O_3$ [M+H]$^+$ m/z = 437.1483; found: 437.1479.

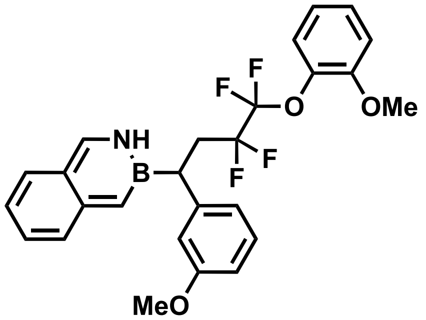

3-(3,3,4,4-tetrafluoro-4-(2-methoxyphenoxy)-1-(3-methoxyphenyl)butyl)-2,3-dihydrobenzo[*d*][1,2]azaborinine (5l): Compound **5l** was synthesized following the general procedure 1 (standard-scale), using 2-(2-bromo-1,1,2,2-tetrafluoroethoxy) anisole (90.91 mg, 0.3 mmol), 2-Vinyl-1,2-dihydrobenzo[e][1,2]azaborinine (31.0 mg, 0.2 mmol) and 3-methoxyphenylmagnesium bromide (0.3 mL, 1.0 M solution in THF, 0.3 mmol). The product **5l** was obtained as a yellow solid (71.1 mg, 73% yield) after purification by column chromatography on silica gel with hexane/EtOAc (8:2).

^{1}H NMR (400 MHz, CDCl$_3$) δ = 8.02 (d, *J* = 11.6 Hz, 1H), 7.59 (d, *J* = 8.1 Hz, 2H), 7.37 (td, *J* = 7.7, 1.5 Hz, 1H), 7.29 – 7.20 (m, 3H), 7.18 – 7.12 (m, 2H), 6.98 (dd, *J* = 8.7, 1.5 Hz, 1H), 6.97 – 6.90 (m, 2H), 6.88 (dd, *J* = 7.7, 1.6 Hz, 1H), 6.84 (d, *J* = 2.6 Hz, 1H), 6.73 (dd, *J* = 8.2, 2.6 Hz, 1H), 3.84 (s, 3H), 3.80 (s, 3H), 3.32 (t, *J* = 7.3 Hz, 1H), 3.16 – 2.98 (m, 1H), 2.93 – 2.76 (m, 1H).

^{13}C NMR (100 MHz, CDCl$_3$) *δ* = 160.0, 152.45 146.0, 145.5, 139.7, 138.0, 129.8, 129.4, 128.3, 127.4, 125.3, 123.8, 121.1, 120.6, 120.7 – 115.3 (m), 120.5, 118.2, 114.2, 112.9, 110.4, 56.0, 55.2, 34.2 (t, *J* = 21.3), 30.5.

^{19}F NMR (376 MHz, CDCl$_3$) δ = -88.25 (s, 2F), -115.06 to -115.58 (m, 2F).

^{11}B NMR (128 MHz, CDCl3) δ = 36.45

IR (film): 649, 702, 765, 779, 810, 854, 908, 987, 1042, 1070, 1108, 1170, 1187, 1219, 1260, 1282, 1388, 1438, 1502, 1561, 1582, 1615, 2837, 3010, 3370cm^{-1}

HRMS (+ESI-FTMS) calcd for $C_{26}H_{24}BF_4NO_3$ [M+H]$^+$ m/z = 486.1858; found: 486.1861.

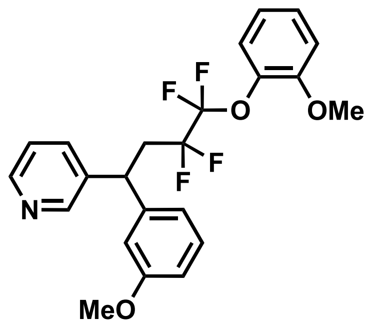

3-(3,3,4,4-tetrafluoro-4-(2-methoxyphenoxy)-1-(3-methoxyphenyl)butyl)pyridine (1a-3N): Compound **1a-3N** was synthesized following the general procedure 1 (standard-scale), using 2-(2-bromo-1,1,2,2-tetrafluoroethoxy) anisole (96.9 mg, 0.32 mmol), 3-Vinylpyridine (21.9 mg, 0.21 mmol) and 3-methoxyphenylmagnesium bromide (0.32 mL, 1.0 M solution in THF, 0.32 mmol). The product **1a-3N** was obtained as a pale yellow oil (30.5 mg, 33% yield) after purification by column chromatography on silica gel with hexane/EtOAc (8:2).

^{1}H NMR (400 MHz, CDCl$_3$) δ = 8.62 (d, *J* = 2.5 Hz, 1H), 8.47 (dd, *J* = 4.8, 1.7 Hz, 1H), 7.62 (dt, *J* = 8.0, 2.1 Hz, 1H), 7.27 – 7.20 (m, 4H), 6.99 – 6.88 (m, 3H), 6.83 (t, *J* = 2.1 Hz, 1H), 6.77 (ddd, *J* = 8.2, 2.6, 1.0 Hz, 1H), 4.56 (t, *J* = 7.3 Hz, 1H), 3.82 (s, 3H), 3.78 (s, 3H), 3.10 – 2.98 (m, 2H).

^{13}C NMR (100 MHz, CDCl$_3$) *δ* = 159.9, 152.4, 149.4, 148.1, 144.3, 139.0, 137.7, 135.0, 129.9, 127.5, 123.8, 123.5, 120.6, 119.8, 113.9, 112.7, 111.8, 56.0, 55.2, 41.7, 36.40 (t, *J* = 20.6 Hz).

^{19}F NMR (376 MHz, CDCl$_3$) δ = -88.61 (s, 2F), -114.86 to -115.68 (m, 2F).

IR (film): 699, 715, 753, 773, 856, 1025, 1044, 1109, 1171, 1190, 1219, 1262, 1302, 1439, 1458, 1503, 1602, 1717, 2852, 2922 cm^{-1}

HRMS (+ESI-FTMS) calcd for $C_{23}H_{21}F_4NO_3$ $[M+H]^+$ m/z = 436.1530; found: 436.1520.

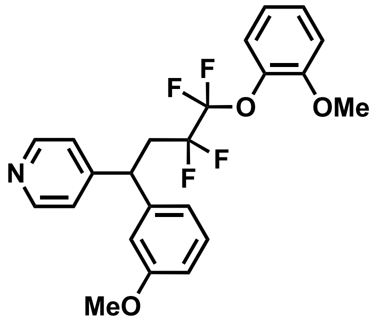

4-(3,3,4,4-tetrafluoro-4-(2-methoxyphenoxy)-1-(3-methoxyphenyl)butyl)pyridine (1a-4N): Compound **1a-4N** was synthesized following a modified general procedure 1 (standard-scale), using 2-(2-bromo-1,1,2,2-tetrafluoroethoxy) anisole (120.4 mg, 0.4 mmol), 4-Vinylpyridine (21.7 mg, 0.2 mmol) and 3-methoxyphenylmagnesium bromide (0.4 mL, 1.0 M solution in THF, 0.4 mmol). The product **1a-4N** was obtained as a pale yellow oil (13.7 mg, 16% yield) after purification by column chromatography on silica gel with hexane/EtOAc (8:2).

^{1}H NMR (400 MHz, CDCl$_3$) δ = 8.53 (s, 2H), 7.27 – 7.19 (m, 5H), 6.98 (dd, *J* = 8.2, 1.5 Hz, 1H), 6.93 (td, *J* = 7.7, 1.5 Hz, 1H), 6.86 (d, *J* = 1.5 Hz, 1H), 6.82 – 6.75 (m, 2H), 4.50 (t, *J* = 7.2 Hz, 1H), 3.82 (s, 3H), 3.79 (s, 3H), 3.15 – 2.91 (m, 2H).

^{13}C NMR (100 MHz, CDCl$_3$) *δ* = 160.0, 152.48, 152.45, 150.1, 143.8, 137.8, 130.1, 127.7, 123.9, 123.1, 120.8, 120.0, 114.2, 112.9, 112.0, 56.1, 55.4, 43.6, 36.2 (t, *J* = 21.1 Hz).

^{19}F NMR (376 MHz, CDCl$_3$) δ = -88.61 (d, *J* = 4.2 Hz), -115.25 (dddt, *J* = 119.3, 22.2, 13.2, 4.9 Hz).

IR (film): 699, 772, 1004, 1044, 1109, 1171, 1191, 1220, 1262, 1301, 1417, 1439, 1458, 1503, 1597, 1731, 2852, 2926 cm^{-1}

HRMS (+APCI-FTMS) calcd for $C_{23}H_{21}F_4NO_3$ [M+H]$^+$ m/z = 436.1530; found: 436.1528.

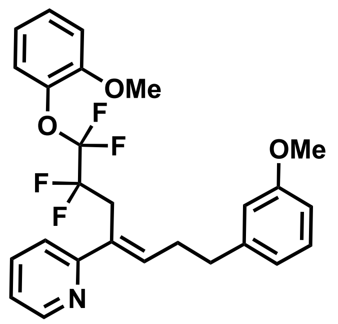

(*E*)-2-(6,6,7,7-tetrafluoro-7-(2-methoxyphenoxy)-1-(3-methoxyphenyl)hept-3-en-4-yl)pyridine (6a): Compound **6a** was synthesized following the general procedure 1 (standard-scale), using 2-(2-bromo-1,1,2,2-tetrafluoroethoxy) anisole (90.91 mg, 0.3 mmol), 2-(1-cyclopropylethenyl)pyridine (29.04 mg, 0.2 mmol) and 3-methoxyphenylmagnesium bromide (0.3 mL, 1.0 M solution in THF, 0.3 mmol). The product **6a** was obtained as a colorless oil (52.4 mg, 55% yield, *E/Z* 90:1) after purification by column chromatography on silica gel with hexane/EtOAc (8:2).

^{1}H NMR (400 MHz, CDCl$_3$) δ = 8.57 (d, *J* = 4.8, 1H), 7.64 (td, *J* = 7.7, 1.9 Hz, 1H), 7.46 (dd, *J* = 8.0, 1.0 Hz, 1H), 7.26 – 7.19 (m, 3H), 7.14 (ddd, *J* = 7.5, 4.8, 1.1 Hz, 1H), 6.98 (dd, *J* = 8.2, 1.5 Hz, 1H), 6.93 (td, *J* = 7.8, 1.5 Hz, 1H), 6.86 (dt, *J* = 7.5, 1.0 Hz, 1H), 6.83 – 6.74 (m, 2H), 6.53 (t, *J* = 7.3 Hz, 1H), 3.83 (s, 3H), 3.82 – 3.69 (m, 5H), 2.86 – 2.79 (m, 2H), 2.74 – 2.65 (m, 2H).

^{13}C NMR (100 MHz, CDCl$_3$) *δ* = 159.8, 159.5, 152.6, 148.7, 143.2, 138.0, 137.4, 136.6, 130.8, 129.5, 127.5, 123.9, 121.8, 121.0, 120.7, 120.7 – 114.4 (m), 120.6, 118.0 (t, *J* = 32.9 Hz), 116.9 (t, *J* = 35.0 Hz), 114.2, 113.0, 111.5, 56.1, 55.2, 35.5, 31.4, 29.5 (t, *J* =21.7 Hz).

^{19}F NMR (376 MHz, CDCl$_3$) δ = -88.49 (t, *J* = 4.2 Hz, 2F), -114.90 (tt, *J* = 18.7, 4.2 Hz, 2F).

IR (film): 697, 715, 749, 773, 815, 853, 995, 1029, 1044, 1105, 1170, 1189, 1261, 1283, 1302, 1435, 1466, 1503, 1566, 1585, 1602, 2839, 2936 cm^{-1}

HRMS (+ESI-FTMS) calcd for $C_{26}H_{25}F_4NO_3$ [M+H]$^+$ m/z = 476.1843; found: 476.1844.

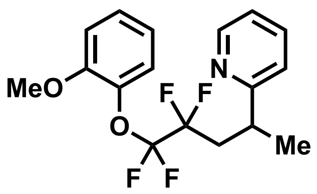

2-(4,4,5,5-tetrafluoro-5-(2-methoxyphenoxy)pentan-2-yl)pyridine (7a):

Compound **7a** was synthesized following the general procedure 1 (standard-scale), using 2-(2-bromo-1,1,2,2-tetrafluoroethoxy) anisole (90.91 mg, 0.3 mmol), 2-(prop-1-en-2-yl)pyridine (23.81 mg, 0.2 mmol) and 3-methoxyphenylmagnesium bromide (0.3 mL, 1.0 M solution in THF, 0.3 mmol). The product **7a** was obtained as a mixture after purification by column chromatography on silica gel with hexane/EtOAc (8:2).

HRMS (+ESI-FTMS) calcd for $C_{17}H_{17}F_4NO_2$ $[M+H]^+$ m/z = 344.1268; found: 344.1262.

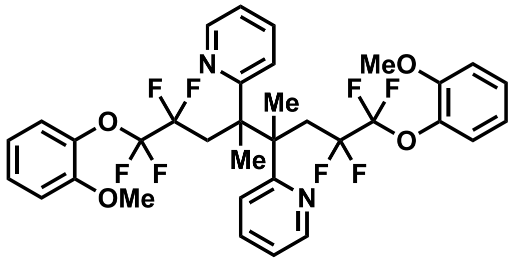

2,2'-(1,1,2,2,7,7,8,8-octafluoro-1,8-bis(2-methoxyphenoxy)-4,5-dimethyloctane-4,5-diyl)dipyridine (7b):

Compound **7b** was synthesized following the general procedure 1 (standard-scale), using 2-(2-bromo-1,1,2,2-tetrafluoroethoxy) anisole (90.91 mg, 0.3 mmol), 2-(prop-1-en-2-yl)pyridine (23.81 mg, 0.2 mmol) and 3-methoxyphenylmagnesium bromide (0.3 mL, 1.0 M solution in THF, 0.3 mmol). The product **7b** was obtained as a mixture after purification by column chromatography on silica gel with hexane/EtOAc (8:2).

HRMS (+ESI-FTMS) calcd for $C_{34}H_{32}F_8N_2O_4$ $[M+H]^+$ m/z = 685.2307; found: 685.2295.

11. General Procedure for Synthesis of Pyridine N-Oxide.

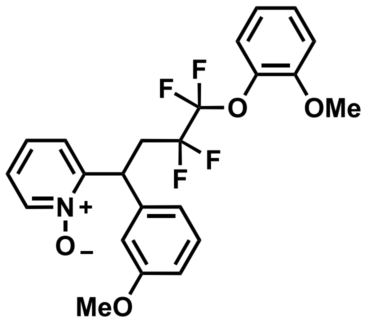

2-(3,3,4,4-tetrafluoro-4-(2-methoxyphenoxy)-1-(3-methoxyphenyl)butyl)pyridine 1-oxide (5k): To a solution of **4a** (0.25 mmol,1.0 equiv.) in DCM (4.5 mL) was added 3-chloroperoxy-benzoic acid (0.3 mmol, 1.2 equiv.) in portions. The mixture was stirred for 12 h at room

temperature. Sodium thiosulfate was then added (5.0 mL) followed by saturated sodium bicarbonate (30 mL). The mixture was extracted with DCM (3 x 5.0 mL). The combined organic layer was dried with sodium sulfate. The resulting mixture was purified via flash column chromatography using EtOAc to obtain the product **5k** as a white powder (91.6 mg, 82% yield).

^{1}H NMR (400 MHz, $CDCl_3$) δ = 8.26 (dd, *J* = 6.4, 1.6 Hz, 1H), 7.40 (dd, *J* = 7.9, 2.0 Hz, 1H), 7.32 – 7.23 (m, 4H), 7.18 (td, *J* = 7.0, 2.1 Hz, 1H), 7.07 – 6.99 (m, 3H), 6.96 (td, *J* = 7.6, 1.5 Hz, 1H), 6.83 (dd, *J* = 7.8, 2.1 Hz, 1H), 5.43 – 5.35 (m, 1H), 3.86 (s, 3H), 3.83 (s, 3H), 3.42 – 3.25 (m, 1H), 3.08 – 2.91 (m, 1H).

^{13}C NMR (100 MHz, $CDCl_3$) *δ* = 159.8, 152.4, 141.0, 140.1, 137.7, 129.6, 127.5, 125.3, 125.2, 124.0, 123.8, 120.6, 120.5, 119.1 – 116.6 (m), 114.5, 112.8, 112.4, 56.0, 55.2, 38.3, 33.3 (t, *J* = 20.9 Hz)

^{19}F NMR (376 MHz, $CDCl_3$) δ = -88.60 (s, 2F), -115.74 (ddd, *J* = 20.1, 14.6, 10.8 Hz, 2F).

IR (film): 664, 697, 751, 769, 849, 937, 1002, 1043, 1107, 1170, 1189, 1219, 1259, 1336, 1430, 1457, 1488, 1502, 1585, 1600, 2840, 2960 cm^{-1}

HRMS (+ESI-FTMS) calcd for $C_{23}H_{21}F_4NO_4$ $[M+H]^+$ m/z = 452.1479; found: 452.1482.

12. Radical Trapping Experiment and Radical Proof

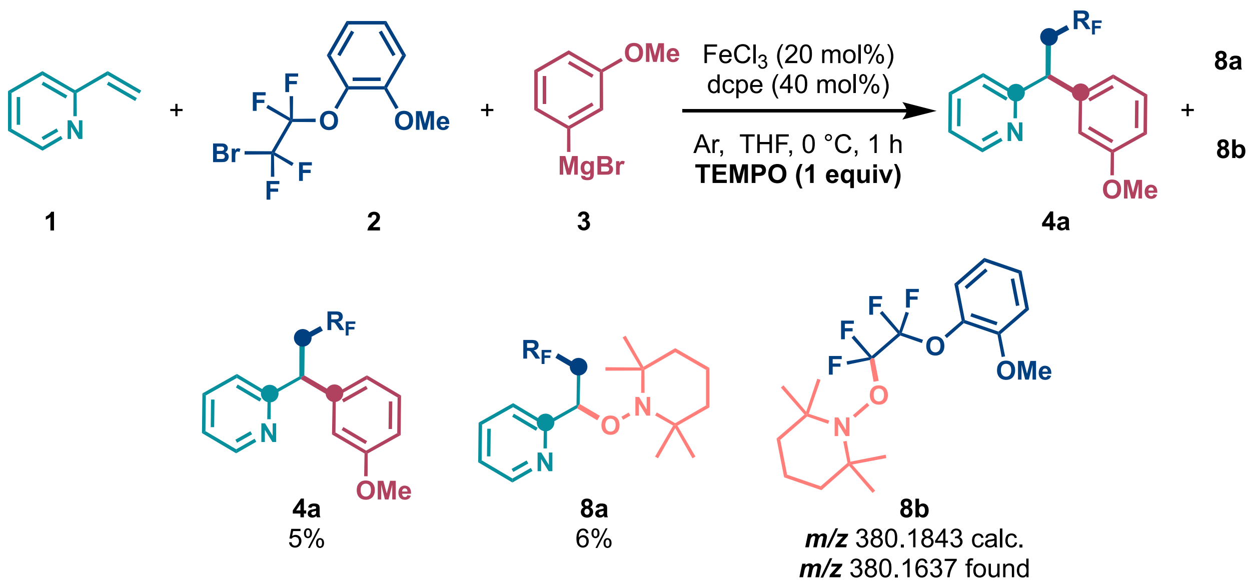

Figure S5. Radical Scavenger Reaction using TEMPO

The procedure is based off general procedure B. A flame-dried 5 mL microwave vial with a stir bar was transferred into an argon-filled glovebox and the vial was charged with 2-(2-bromo-1,1,2,2-tetrafluoroethoxy) anisole (0.3 mmol, 1.5 equiv), 2-vinylpyridine (0.2 mmol, 1.0 equiv), and TEMPO (0.2 mmol, 1.0 equiv). The vial was sealed with a Teflon cap and brought out of the glovebox without solvent. THF (0.2 mL) was then added into the reaction mixture. The resulting solution was stirred at room temperature for 5 min. The reaction mixture was then cooled to 0 °C using an ice bath and 3-methoxyphenylmagnesium bromide (0.9 M solution in THF, 0.3 mmol, 1.5 equiv) was added slowly for 1 h using a syringe pump. The resulting reaction mixture was then stirred at 0 °C for an additional 10 min. After completion, the reaction mixture was quenched with a 1.0 M aqueous solution of hydrochloric acid (0.1 mL) and water (0.5 mL) and then extracted with ethyl acetate (3 x 2 mL). The combined organic solution was filtered through a short pad of silica and evaporation of solvent gave a residue that was used for crude ^{1}H NMR and HRMS. Product **8b** was detected using +ESI-FTMS. The product **4a** was obtained as a colorless liquid (5 mg, 5% yield) and product **8a** was obtained as a colorless liquid (5.8 mg, 6%) after purification by column chromatography on silica gel with hexane/EtOAc (8:2).

8a HRMS (+ESI-FTMS) calcd for $C_{25}H_{32}F_4N_2O_3$ $[M+H]^+$ m/z = 485.2422; found: 485.2417.

8b HRMS (+ESI-FTMS) calcd for $C_{18}H_{25}F_4NO_3$ $[M+H]^+$ m/z = 380.1843; found: 380.1837.

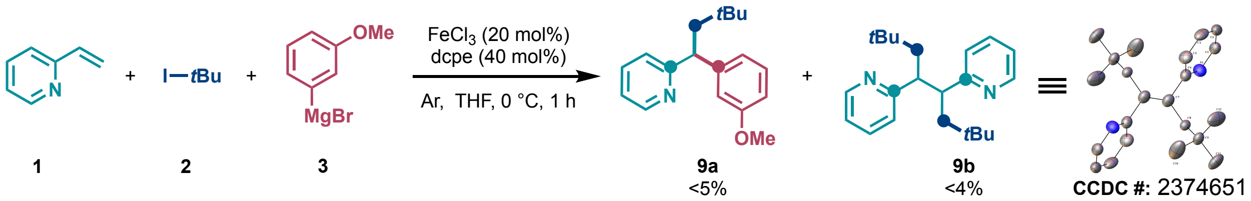

Figure S6. Reaction using *t*Bu-I yielding a dimer

The procedure is based off general procedure B. A flame-dried 5 mL microwave vial with a stir bar was transferred into an argon-filled glovebox and the vial was charged with *tert*-butyl iodide (0.3 mmol, 1.5 equiv), 2-vinylpyridine (0.2 mmol, 1.0 equiv). The vial was sealed with a Teflon cap and brought out of the glovebox without solvent. THF (0.2 mL) was then added into the reaction mixture. The resulting solution was stirred at room temperature for 5 min. The reaction mixture was then cooled to 0 °C using an ice bath and 3-methoxyphenylmagnesium bromide (0.9 M solution in THF, 0.3 mmol, 1.5 equiv) was added slowly for 1 h using a syringe pump. The resulting reaction mixture was then stirred at 0 °C for an additional 10 min. After completion, the reaction mixture was quenched with a 1.0 M aqueous solution of hydrochloric acid (0.1 mL) and water (0.5 mL) and then extracted with ethyl acetate (3 x 2 mL). The combined organic solution was filtered through a short pad of silica and evaporation of solvent gave the crude mixture. The product **9a** was obtained as a light yellow oil (5 mg, 5% yield) and product **9b** was obtained as a light yellow solid (4.7 mg, 4%) after purification by column chromatography on silica gel with hexane/EtOAc (7:3). The product **9b** was crystallized using chloroform, and single crystal x-ray diffraction gave the crystal structure of the dimer.

13. Crystal Data and Experimental for Product 9b

The single crystal of **9b** (CCDC 2374651) was prepared by diffusing of chloroform in a solution of **9b**. ORTEP drawing of **9b** showing the ellipsoids at the 50% probability level (crystal size 0.30 × 0.08 × 0.02 mm^3).

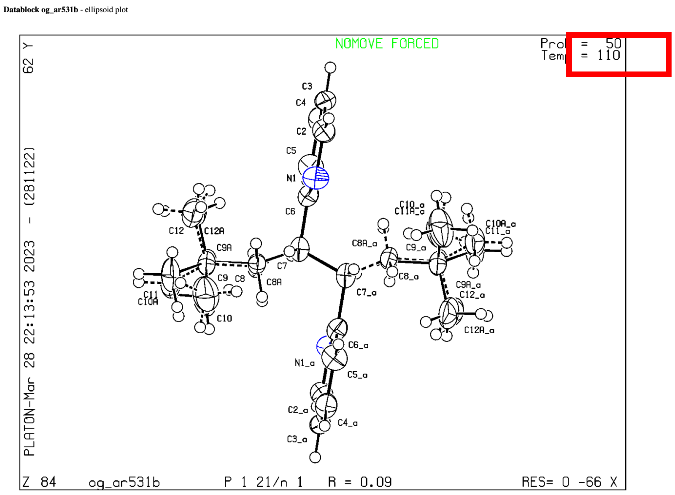

Figure S7. ORTEP drawing of **9b**

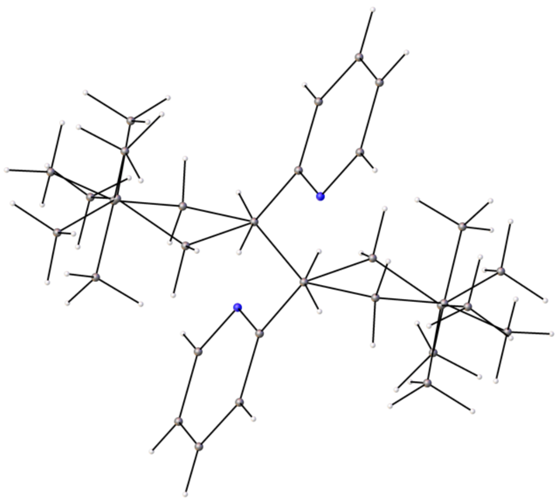

Experimental. Single colourless plate-shaped crystals of **OG_AR531B** were used as supplied. A suitable crystal with dimensions $0.30 \times 0.08 \times 0.02$ mm^3 was selected and mounted on a MITIGEN holder on a Bruker Venture (PHOTON III) diffractometer. The crystal was kept at a steady T = 110.00 K during data collection. The structure was solved with the ShelXT 2018/2 (Sheldrick, 2018) solution program using dual methods and by using Olex2 1.5 (Dolomanov et al., 2009) as the graphical interface. The model was refined with XL (Sheldrick, 2008) using full matrix least squares minimisation on F^2.

Crystal Data. $C_{22}H_{32}N_2$, M_r = 324.49, monoclinic, $P2_1/n$ (No. 14), a = 6.1860(3) Å, b = 9.6516(5) Å, c = 17.2249(9) Å, β = 97.047(3)°, α = γ = 90°, V = 1020.64(9) Å^3, T = 110.00 K, Z = 2, Z' = 0.5, μ(CuK_α) = 0.459, 6281 reflections measured, 1743 unique (R_{int} = 0.0345) which were used in all calculations. The final wR_2 was 0.2396 (all data) and R_1 was 0.0882 (I≥2 σ(I)).

**Compound**	**OG_AR531B**
Formula	$C_{22}H_{32}N_2$
$D_{calc.}$/ g cm^{-3}	1.056
μ/mm^{-1}	0.459
Formula Weight	324.49
Colour	colourless
Shape	plate-shaped
Size/mm^3	0.30×0.08×0.02
T/K	110.00
Crystal System	monoclinic
Space Group	$P2_1/n$
a/Å	6.1860(3)
b/Å	9.6516(5)
c/Å	17.2249(9)
α/°	90
β/°	97.047(3)
γ/°	90
V/Å^3	1020.64(9)
Z	2
Z'	0.5
Wavelength/Å	1.54178
Radiation type	CuK_α
Θ_{min}/°	5.175
Θ_{max}/°	65.071
Measured Refl's.	6281
Indep't Refl's	1743
Refl's I≥2 σ(I)	1573
R_{int}	0.0345
Parameters	155
Restraints	265
Largest Peak	0.927
Deepest Hole	-0.282
GooF	1.046
wR_2 (all data)	0.2396
wR_2	0.2348
R_1 (all data)	0.0934
R_1	0.0882

Structure Quality Indicators

**Reflections:**	d min (Cu\a) 2Θ=130.1° 0.85	I/σ(I) 31.3	Rint m=3.74 3.45%	Full 130.1° 99.8
**Refinement:**	Shift 0.000	Max Peak 0.9	Min Peak -0.3	GooF 1.046

Experimental (Long Form)

Data Collection

A Leica M80 microscope was used to identify a suitable single **colourless plate-shaped** crystal of **OG_AR531B** showing well defined faces with dimensions 0.30 × 0.08 × 0.02 mm^3 from a representative sample of crystals of the same habit. The crystal mounted on a nylon loop was then placed in a cold nitrogen stream (Oxford) maintained at T = 110.00 K.

Crystal screening, unit cell determination, and data collection were carried out using a Bruker Venture (PHOTON III) diffractometer. The diffraction pattern was indexed and the total number of runs and images was based on the strategy calculation from the program APEX 3 (Bruker, 2018). Data were measured using ϕ and ω scans with CuK_α radiation. Data was collected to a maximum resolution of Θ = 65.071° (0.85 Å). The unit cell was refined using SAINT V8.38A (Bruker, 2018) on 4577 reflections, 73 % of the observed reflections.

Data Reduction, Structure Solution, and Refinement

Integrated Intensity information for each reflection was obtained by reduction of data frames using SAINT V8.38A (Bruker, 2018). The final completeness is 99.80 % out to 65.071° in Θ. SADABS-2016/2 (Bruker, 2016/2) was used for absorption correction. wR_2(int) was 0.0930 before and 0.0441 after correction. The Ratio of minimum to maximum transmission is 0.8325. The λ/2 correction factor is Not present. The absorption coefficient μ of this material is 0.459 mm^{-1} at this wavelength (λ = 1.54178Å) and the minimum and maximum transmissions are 0.628 and 0.754.

Systematic reflection conditions and statistical tests of the data suggested the space group $P2_1/n$ (# 14) and was confirmed by ShelXT 2018/2 (Sheldrick, 2018) structure solution program using dual methods. The structure was refined by full matrix least squares minimisation on $\boldsymbol{F^2}$ using version 2018/3 of XL (Sheldrick, 2008). All non-hydrogen atoms were refined anisotropically. Hydrogen atom positions were calculated geometrically and refined using the riding model.

_refine_special_details: Diffraction from the crystal was weak. Data collection was extended to 60s per degree at higher angles. The compound crystallized in the space group P21/n with Z=2, Z'=0.5. Elongated ellipsoids and nearby residual electron density peaks indicated atoms C8-C12 were disordered and was modeled between two positions with an occupancy ratio of 0.82. Appropriate restraints and constraints were added to keep the bond distances, angles, and thermal ellipsoids meaningful.

_exptl_absorpt_process_details: SADABS-2016/2 (Bruker,2016/2) was used for absorption correction. wR_2(int) was 0.0930 before and 0.0441 after correction. The Ratio of minimum to maximum transmission is 0.8325. The λ/2 correction factor is Not present.

The value of Z' is 0.5. This means that only half of the formula unit is present in the asymmetric unit, with the other half consisting of symmetry equivalent atoms.

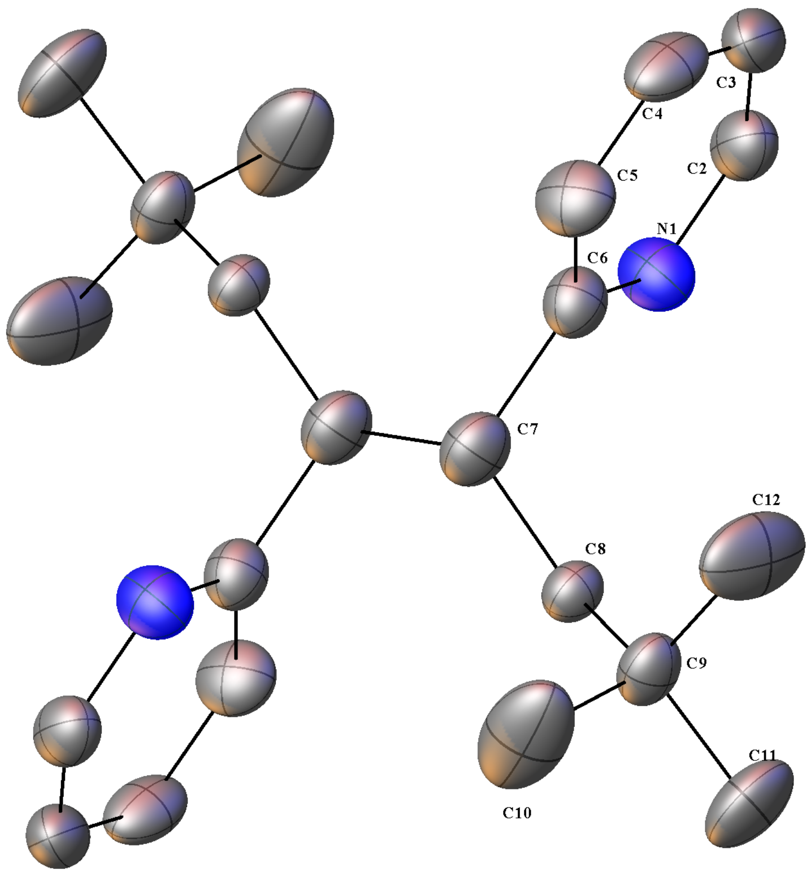

Figure S8: Thermal ellipsoids plot (50 % probability) of OG_AR531B. Hydrogen atoms are omitted for clarity. Symmetry generated atoms are not labeled.

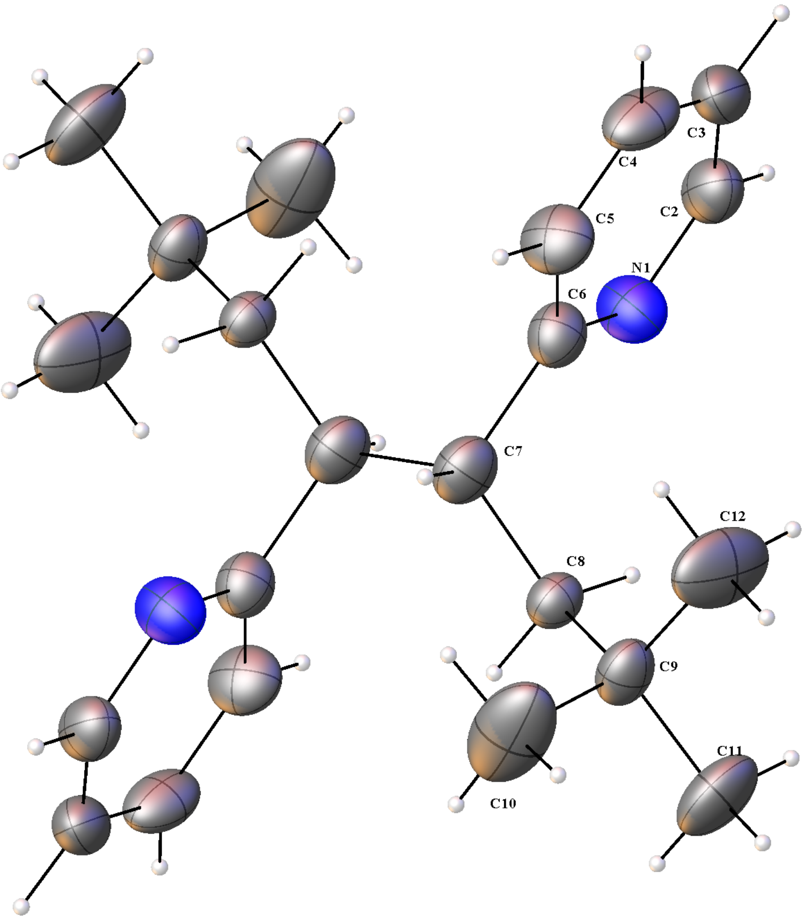

Figure S9: Thermal ellipsoids plot (50 % probability) of OG_AR531B. Hydrogen atoms are shown at an arbitrarily chosen small radius, and not labeled, for clarity. Symmetry generated atoms are not labeled.

Reflection Statistics

Total reflections (after filtering)	6517	Unique reflections	1743
Completeness	0.998	Mean I/σ	24.77
hkl_{max} collected	(7, 12, 21)	hkl_{min} collected	(-6, -12, -21)
hkl_{max} used	(7, 11, 20)	hkl_{min} used	(-7, 0, 0)
Lim d_{max} collected	999.0	Lim d_{min} collected	0.85
d_{max} used	17.09	d_{min} used	0.85
Friedel pairs	1757	Friedel pairs merged	1
Inconsistent equivalents	0	R_{int}	0.0345
R_{sigma}	0.0319	Intensity transformed	0
Omitted reflections	0	Omitted by user (OMIT hkl)	0
Multiplicity	(4188, 1181, 165, 59)	Maximum multiplicity	10
Removed systematic absences	236	Filtered off (Shel/OMIT)	764

Table S7: Fractional Atomic Coordinates (×10^4) and Equivalent Isotropic Displacement Parameters (Å^2×10^3) for **OG_AR531B**. U_{eq} is defined as 1/3 of the trace of the orthogonalised U_{ij}.

Atom	x	y	z	U_{eq}
N1	5110(4)	7541(2)	5616.1(15)	42.8(7)
C2	4330(5)	8820(3)	5712.1(17)	41.9(7)
C3	2153(5)	9105(3)	5704.4(16)	43.9(8)
C4	705(4)	8037(4)	5575.9(17)	47.4(8)
C5	1468(4)	6714(3)	5476.6(18)	44.8(8)

Atom	x	y	z	U_{eq}
C6	3681(4)	6488(3)	5516.7(15)	36.5(7)
C7	4603(5)	5039(3)	5402.1(15)	41.1(7)
C8	6200(5)	4632(3)	6091.5(17)	30.8(8)
C9	5361(7)	3928(4)	6794(2)	37.6(7)
C10	4405(9)	2523(5)	6561(3)	84.2(17)
C11	7289(7)	3718(5)	7428(2)	62.8(12)
C12	3663(8)	4819(6)	7128(3)	75.2(16)
C8A	4440(20)	4007(15)	5944(7)	34(2)
C9A	5420(20)	3870(15)	6802(7)	37.6(7)
C10A	4470(30)	2603(15)	7166(11)	59(4)
C11A	7870(20)	3680(20)	6820(13)	77(4)
C12A	5010(40)	5146(15)	7285(11)	61(4)

Table S8: Anisotropic Displacement Parameters (×10[4]) for **OG_AR531B**. The anisotropic displacement factor exponent takes the form: $-2\pi^2[h^2a^{*2} \times U_{11} + ... + 2hka^* \times b^* \times U_{12}]$

Atom	U_{11}	U_{22}	U_{33}	U_{23}	U_{13}	U_{12}
N1	30.7(12)	42.5(14)	54.6(15)	-4.0(11)	2.3(10)	-2.3(10)
C2	46.1(16)	36.2(14)	41.0(15)	-2.2(11)	-3.9(12)	-4.3(12)
C3	54.8(17)	43.0(16)	32.9(15)	-3.8(11)	1.5(12)	15.4(13)
C4	28.7(13)	74(2)	39.7(15)	7.9(14)	5.5(11)	16.6(14)
C5	28.8(14)	50.2(17)	53.7(18)	-0.6(13)	-1.8(12)	-6.3(12)
C6	33.2(14)	43.8(15)	32.7(14)	-2.6(11)	4.8(10)	-0.8(11)
C7	44.2(15)	46.9(16)	32.3(15)	4.9(11)	5.4(12)	-3.2(12)
C8	30.3(14)	36.1(16)	25.9(15)	2.2(11)	3.2(11)	1.3(12)
C9	45.6(15)	40.7(15)	26.2(13)	3.8(10)	3.7(11)	-6.1(11)
C10	127(4)	59(3)	61(3)	19(2)	-10(3)	-43(3)
C11	66(3)	87(3)	33(2)	17.5(19)	-3.6(17)	-3(2)
C12	76(3)	112(4)	44(2)	27(2)	32(2)	30(3)
C8A	40(4)	39(4)	24(4)	1(4)	6(4)	-1(4)
C9A	45.6(15)	40.7(15)	26.2(13)	3.8(10)	3.7(11)	-6.1(11)
C10A	82(9)	57(8)	38(7)	12(7)	8(7)	-10(7)
C11A	67(7)	97(9)	63(8)	17(8)	-7(7)	2(8)
C12A	80(9)	66(8)	36(7)	1(6)	8(8)	-2(8)

Table S9: Bond Lengths in Å for **OG_AR531B**.

Atom	Atom	Length/Å
N1	C2	1.344(4)
N1	C6	1.344(4)
C2	C3	1.373(4)
C3	C4	1.366(4)
C4	C5	1.379(4)
C5	C6	1.380(4)
C6	C7	1.532(4)
C7	C7[1]	1.527(5)
C7	C8	1.501(4)
C7	C8A	1.378(14)

Atom	Atom	Length/Å
C8	C9	1.533(5)
C9	C10	1.513(5)
C9	C11	1.528(5)
C9	C12	1.524(5)
C8A	C9A	1.532(10)
C9A	C10A	1.522(7)
C9A	C11A	1.525(7)
C9A	C12A	1.525(7)

----
[1]1-x,1-y,1-z

Table S10: Bond Angles in ° for **OG_AR531B**.

Atom	Atom	Atom	Angle/°
C2	N1	C6	118.0(2)
N1	C2	C3	123.3(3)

Atom	Atom	Atom	Angle/°
C4	C3	C2	118.3(3)
C3	C4	C5	119.4(2)

Atom	Atom	Atom	Angle/˚
C4	C5	C6	119.5(3)
N1	C6	C5	121.3(3)
N1	C6	C7	117.2(2)
C5	C6	C7	121.4(3)
C7[1]	C7	C6	109.2(3)
C8	C7	C6	110.9(2)
C8	C7	C7[1]	116.8(3)
C8A	C7	C6	120.8(6)
C8A	C7	C7[1]	129.7(7)
C7	C8	C9	119.1(3)
C10	C9	C8	110.3(3)
C10	C9	C11	108.4(3)
C10	C9	C12	109.9(4)

Atom	Atom	Atom	Angle/˚
C11	C9	C8	108.2(3)
C12	C9	C8	111.3(3)
C12	C9	C11	108.6(3)
C7	C8A	C9A	131.5(12)
C10A	C9A	C8A	110.0(12)
C10A	C9A	C11A	108.9(7)
C10A	C9A	C12A	109.1(7)
C11A	C9A	C8A	107.8(13)
C12A	C9A	C8A	112.6(12)
C12A	C9A	C11A	108.4(7)

––––
[1]1-x,1-y,1-z

Table S11: Torsion Angles in ˚ for **OG_AR531B**.

Atom	Atom	Atom	Atom	Angle/˚
N1	C2	C3	C4	-1.5(4)
N1	C6	C7	C7[1]	-70.8(4)
N1	C6	C7	C8	59.3(3)
N1	C6	C7	C8A	115.5(7)
C2	N1	C6	C5	3.0(4)
C2	N1	C6	C7	179.6(2)
C2	C3	C4	C5	1.5(4)
C3	C4	C5	C6	0.7(4)
C4	C5	C6	N1	-3.0(4)
C4	C5	C6	C7	-179.4(3)
C5	C6	C7	C7[1]	105.7(3)
C5	C6	C7	C8	-124.2(3)
C5	C6	C7	C8A	-68.0(8)
C6	N1	C2	C3	-0.8(4)
C6	C7	C8	C9	87.4(3)
C6	C7	C8A	C9A	-64.7(17)
C7[1]	C7	C8	C9	-146.8(3)
C7[1]	C7	C8A	C9A	123.0(14)
C7	C8	C9	C10	65.3(4)
C7	C8	C9	C11	-176.3(3)
C7	C8	C9	C12	-57.0(4)
C7	C8A	C9A	C10A	172.6(13)
C7	C8A	C9A	C11A	-68.9(17)
C7	C8A	C9A	C12A	50.7(19)

––––
[1]1-x,1-y,1-z

Table S12: Hydrogen Fractional Atomic Coordinates (×10^4) and Equivalent Isotropic Displacement Parameters (Å^2×10^3) for **OG_AR531B**. U_{eq} is defined as 1/3 of the trace of the orthogonalised U_{ij}.

Atom	x	y	z	U_{eq}
H2	5335.27	9565.95	5789.17	50
H3	1665.99	10020.61	5786.39	53
H4	-814.72	8203.26	5555.15	57
H5	477.19	5963.39	5381.2	54
H7B	6079.64	5315.08	5657.61	49
H7	3351.66	4380.46	5389.76	49
H8A	6991.24	5479.9	6285.47	37
H8B	7281.73	4004.34	5898.78	37
H10D	3215.65	2635.2	6134.36	126
H10E	3842.27	2094.99	7011.49	126

Atom	x	y	z	U_{eq}
H10F	5537.67	1930.74	6386.48	126
H11D	8389.06	3137.96	7222.02	94
H11E	6787.84	3261.75	7881.32	94
H11F	7928.04	4619.06	7585.7	94
H12D	4292.37	5726.06	7278.06	113
H12E	3205.65	4362.12	7589.47	113
H12F	2398.01	4940.95	6732.22	113
H8AA	4915.33	3158.78	5691.28	41
H8AB	2849.64	3895.61	5955	41
H10A	2895.9	2714.88	7153.28	88
H10B	5141.32	2506.19	7709.4	88
H10C	4780.14	1774.05	6869.88	88
H11A	8189.19	2697.7	6735.38	115
H11B	8603.32	3972.49	7329.11	115
H11C	8390.1	4236.47	6405.64	115
H12A	6240.08	5788.97	7284.95	91
H12B	4874.41	4865.8	7823.69	91
H12C	3669.38	5603.76	7058.04	91

Table S13: Atomic Occupancies for all atoms that are not fully occupied in **OG_AR531B**.

Atom	Occupancy
H7B	0.178(4)
H7	0.822(4)
C8	0.822(4)
H8A	0.822(4)
H8B	0.822(4)
C9	0.822(4)
C10	0.822(4)
H10D	0.822(4)
H10E	0.822(4)
H10F	0.822(4)
C11	0.822(4)
H11D	0.822(4)
H11E	0.822(4)
H11F	0.822(4)
C12	0.822(4)
H12D	0.822(4)
H12E	0.822(4)
H12F	0.822(4)
C8A	0.178(4)
H8AA	0.178(4)
H8AB	0.178(4)
C9A	0.178(4)
C10A	0.178(4)
H10A	0.178(4)
H10B	0.178(4)
H10C	0.178(4)
C11A	0.178(4)
H11A	0.178(4)
H11B	0.178(4)
H11C	0.178(4)
C12A	0.178(4)
H12A	0.178(4)
H12B	0.178(4)
H12C	0.178(4)

Citations

APEX 3 (Bruker, 2018)

O.V. Dolomanov and L.J. Bourhis and R.J. Gildea and J.A.K. Howard and H. Puschmann, Olex2: A complete structure solution, refinement and analysis program, *J. Appl. Cryst.*, (2009), **42**, 339-341.

SADABS, Bruker axs, Madison, WI (?).

Sheldrick, G.M., Crystal structure refinement with ShelXL, *Acta Cryst.*, (2015), **C71**, 3-8.

Sheldrick, G.M., ShelXT-Integrated space-group and crystal-structure determination, *Acta Cryst.*, (2015), **A71**, 3-8.

Software for the Integration of CCD Detector System Bruker Analytical X-ray Systems, Bruker axs, Madison, WI (after 2013).

14. Spectral Data.

Compound 4a. ^{1}H NMR ($CDCl_3$, 400 MHz). Bottom: ^{13}C NMR ($CDCl_3$, 100 MHz).

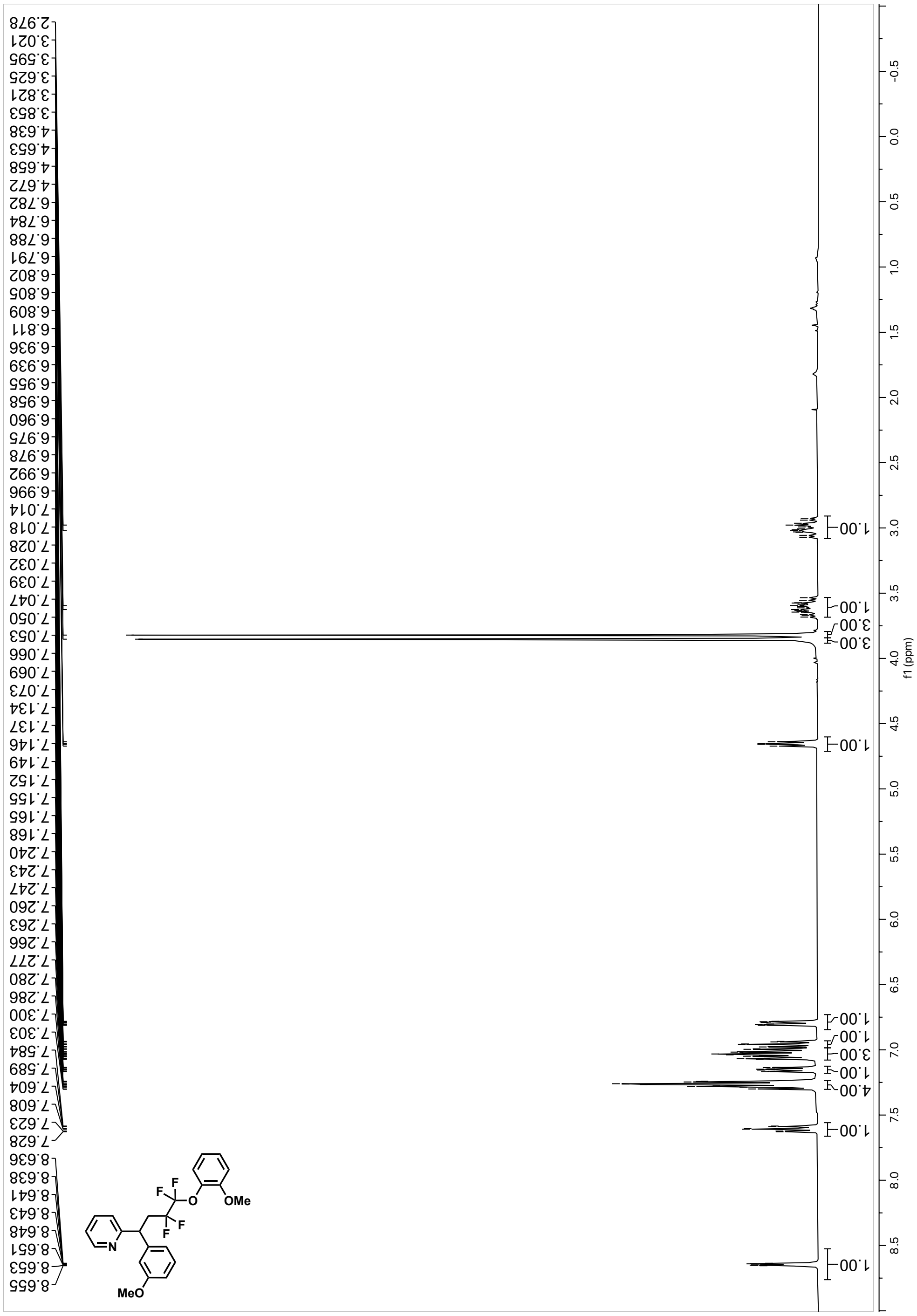

Compound 4a. ^{13}C NMR ($CDCl_3$, 100 MHz).

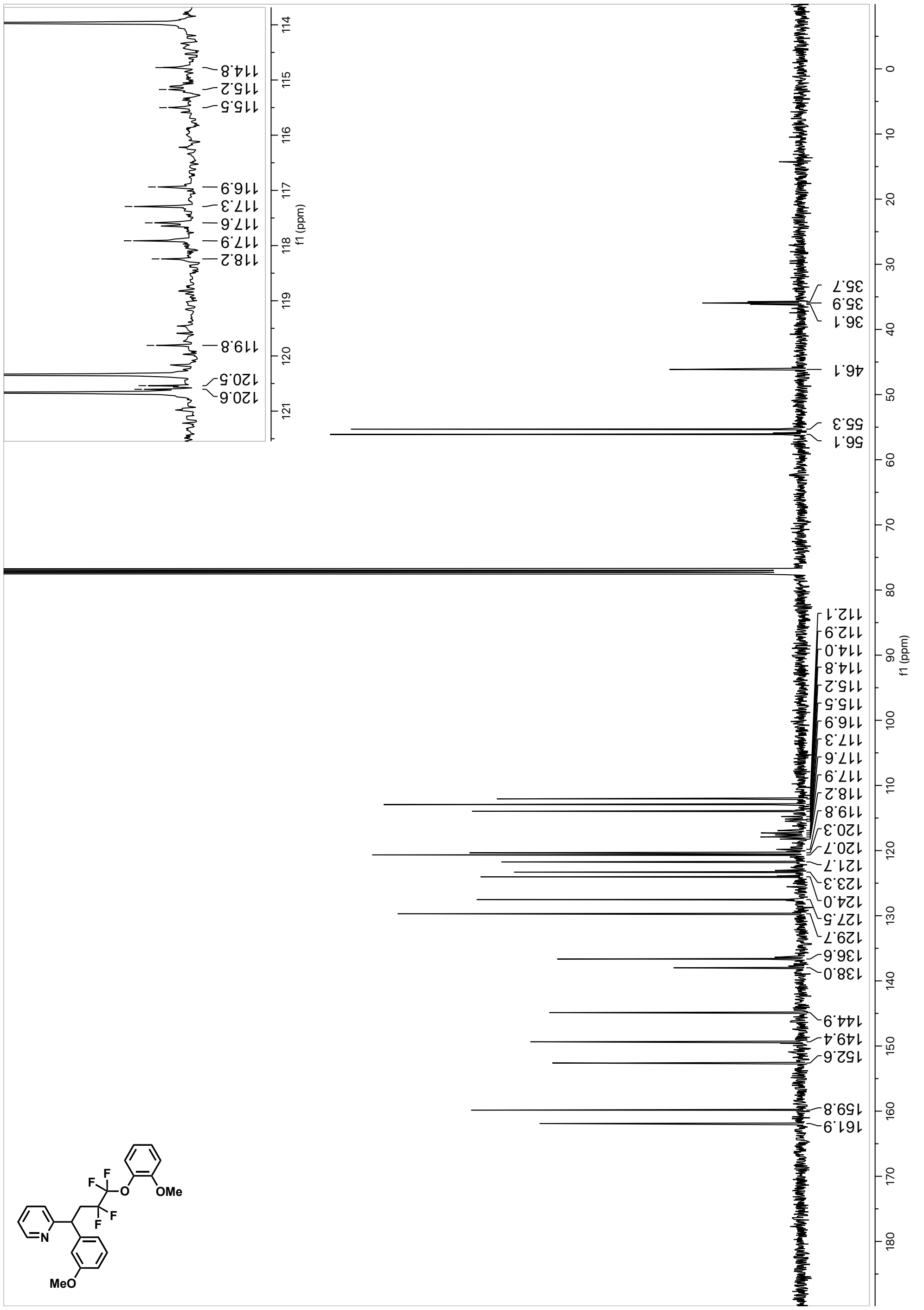

Compound 4a. ^{19}F NMR ($CDCl_3$, 376 MHz).

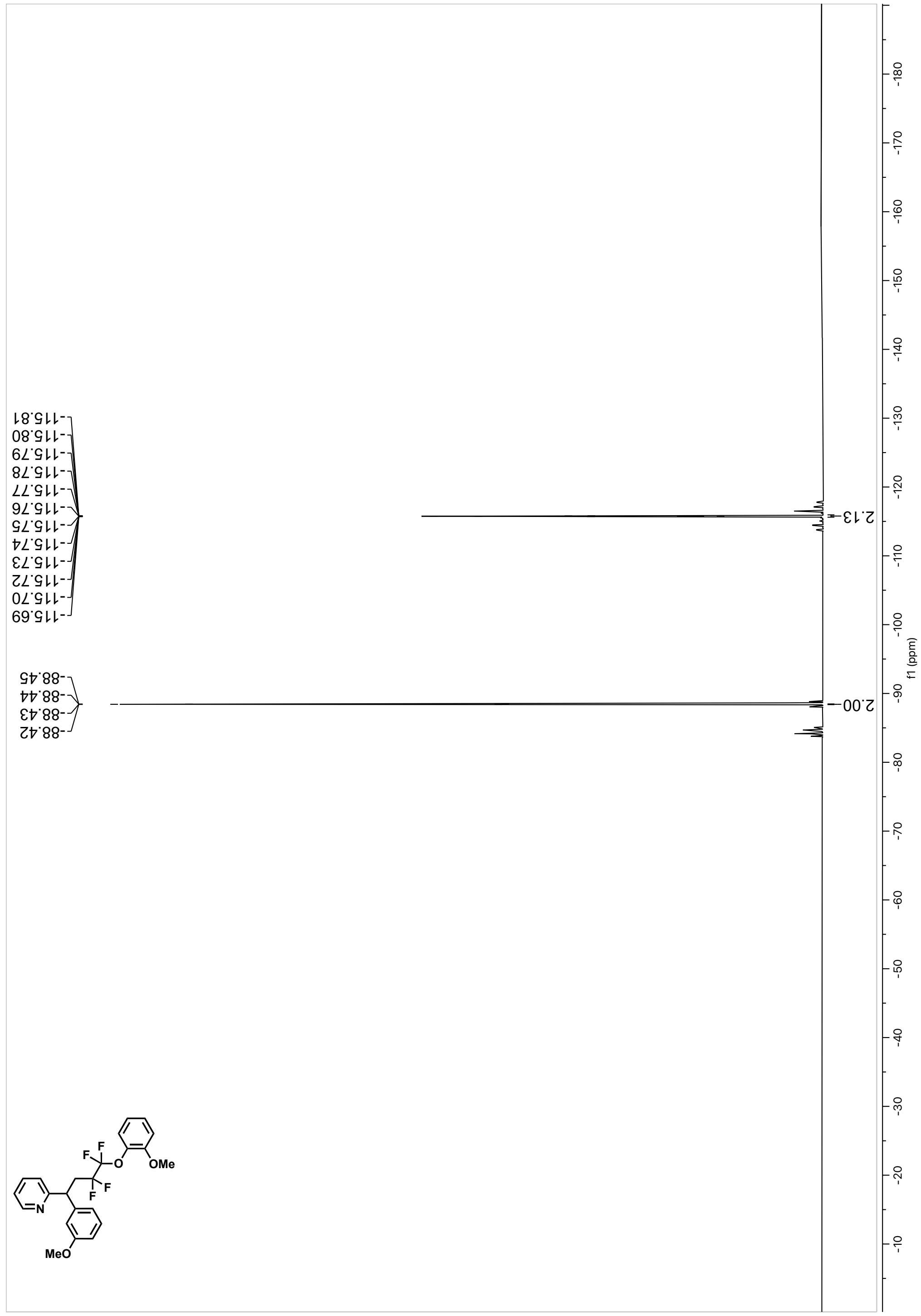

Compound 4b. ^{1}H NMR ($CDCl_3$, 400 MHz).

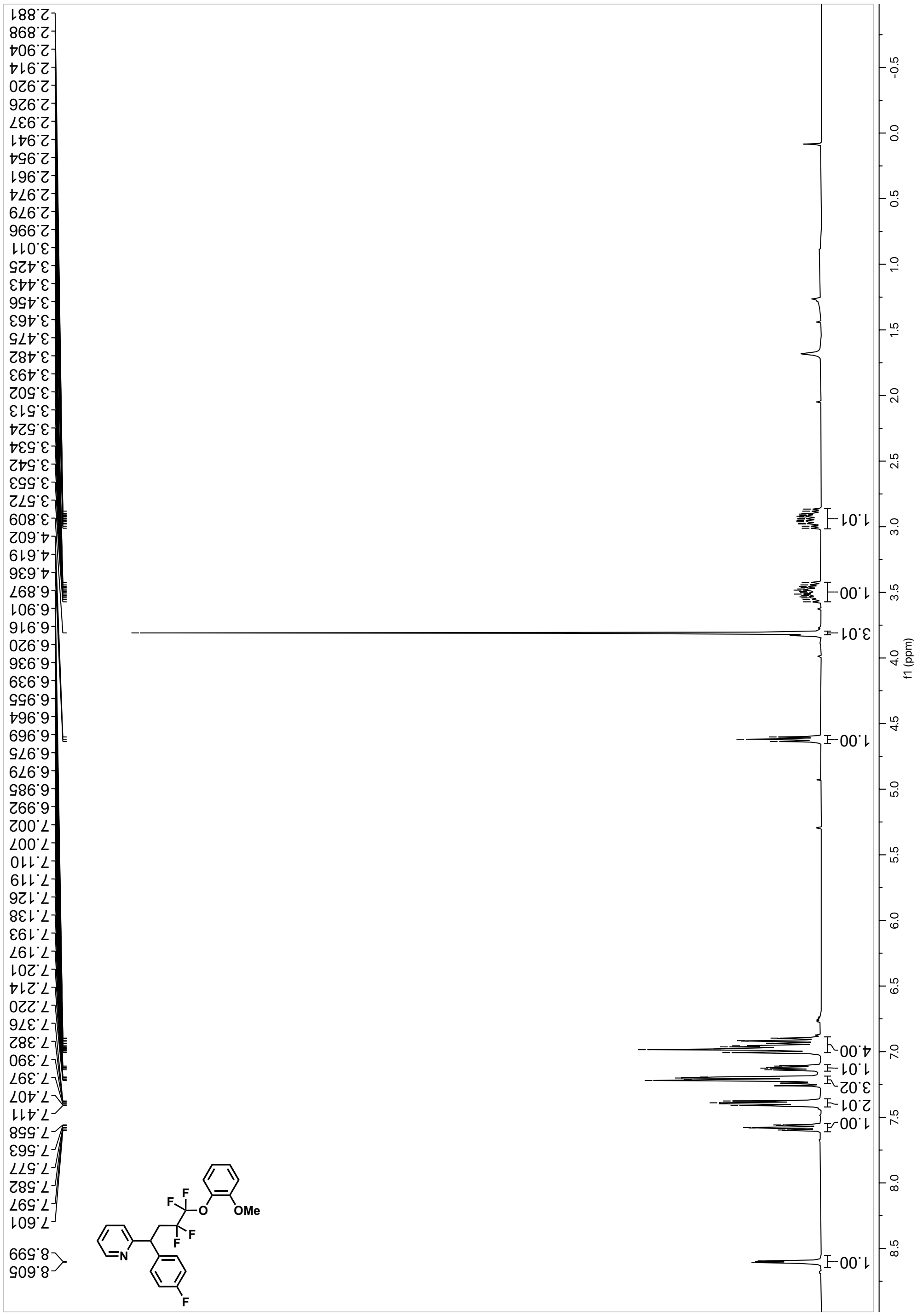

Compound 4b. ^{13}C NMR ($CDCl_3$, 100 MHz).

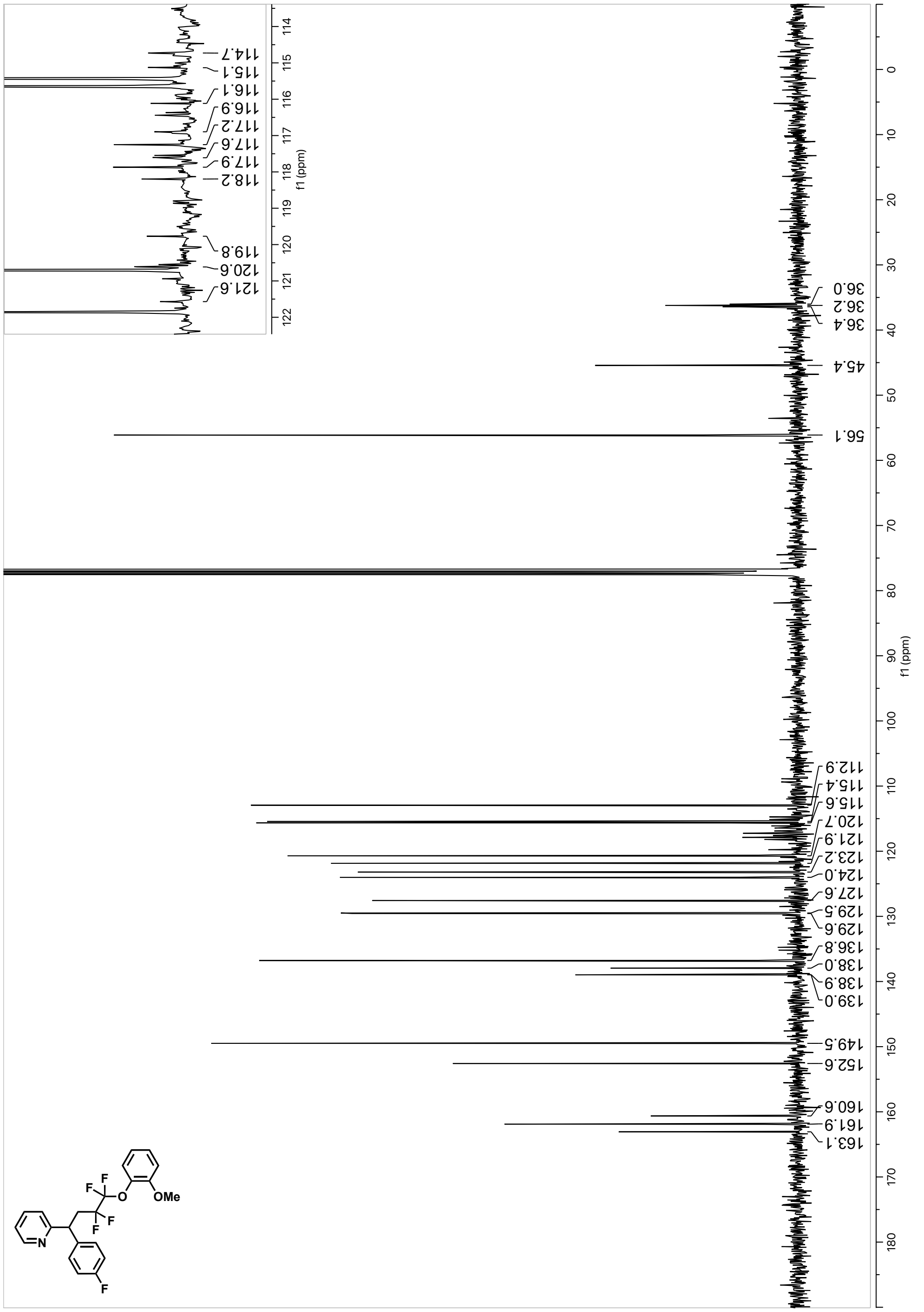

Compound 4b. ^{19}F NMR ($CDCl_3$, 376 MHz).

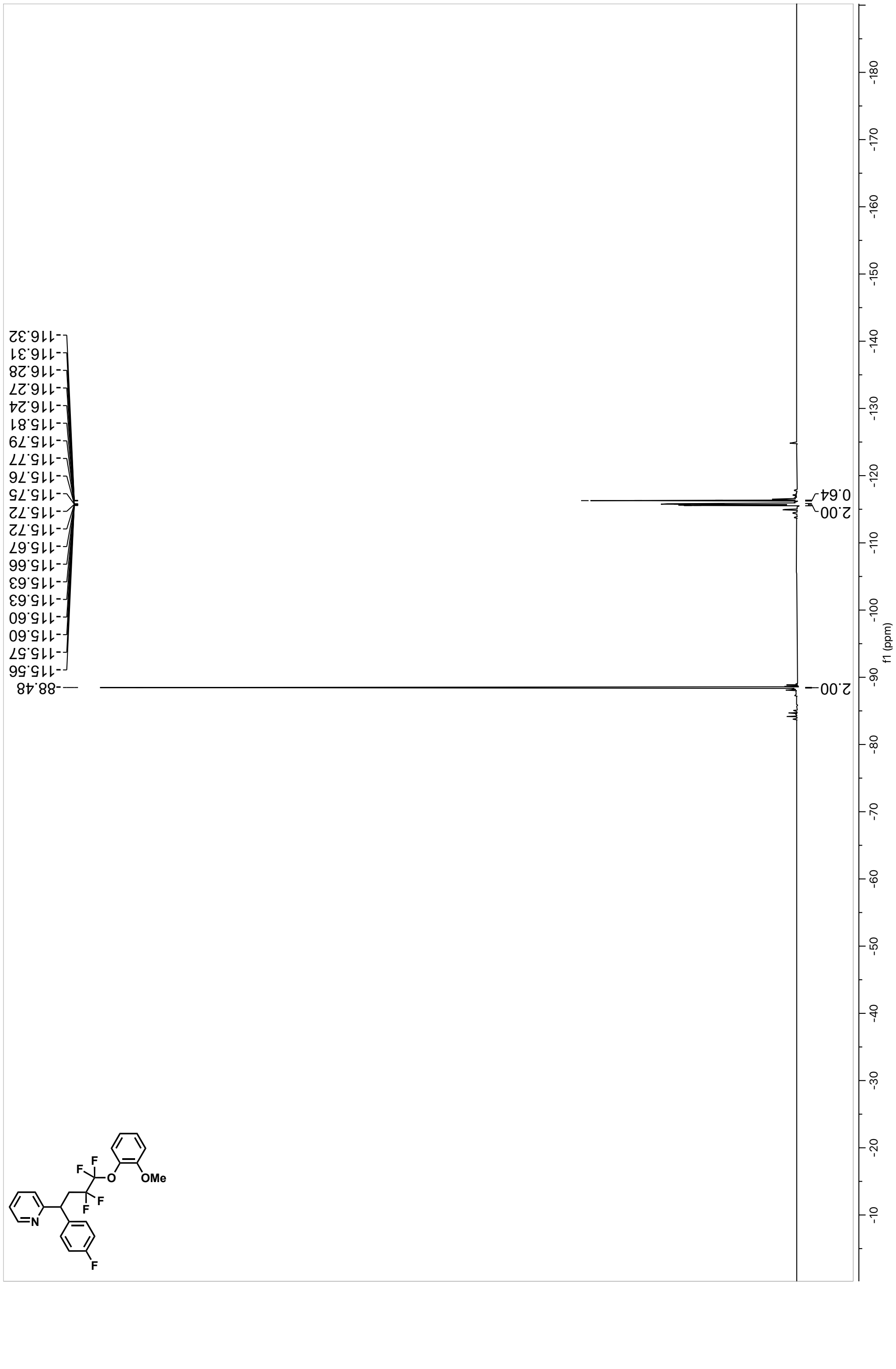

Compound 4c. ^{1}H NMR ($CDCl_3$, 400 MHz).

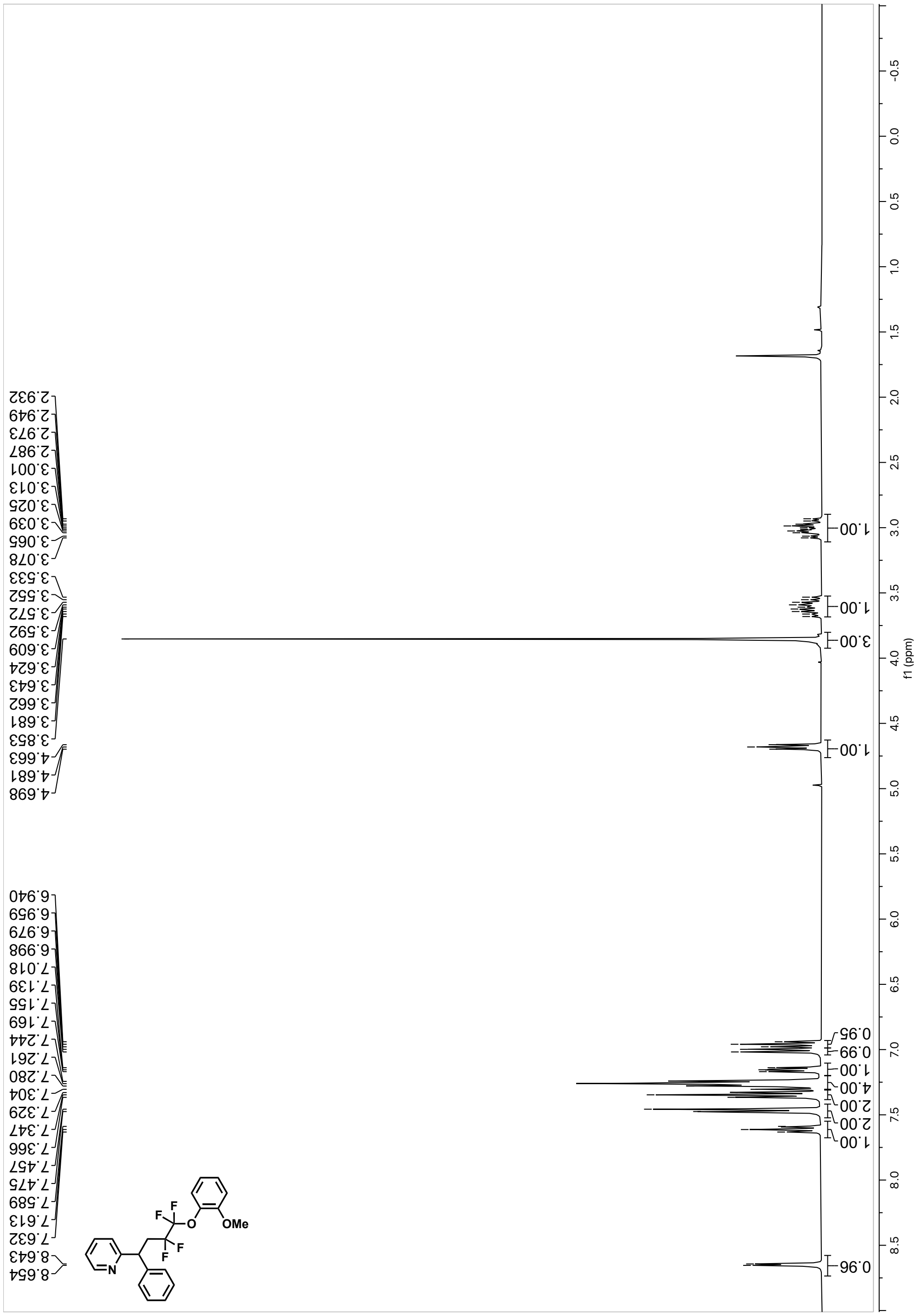

Compound 4c. ^{13}C NMR ($CDCl_3$, 100 MHz).

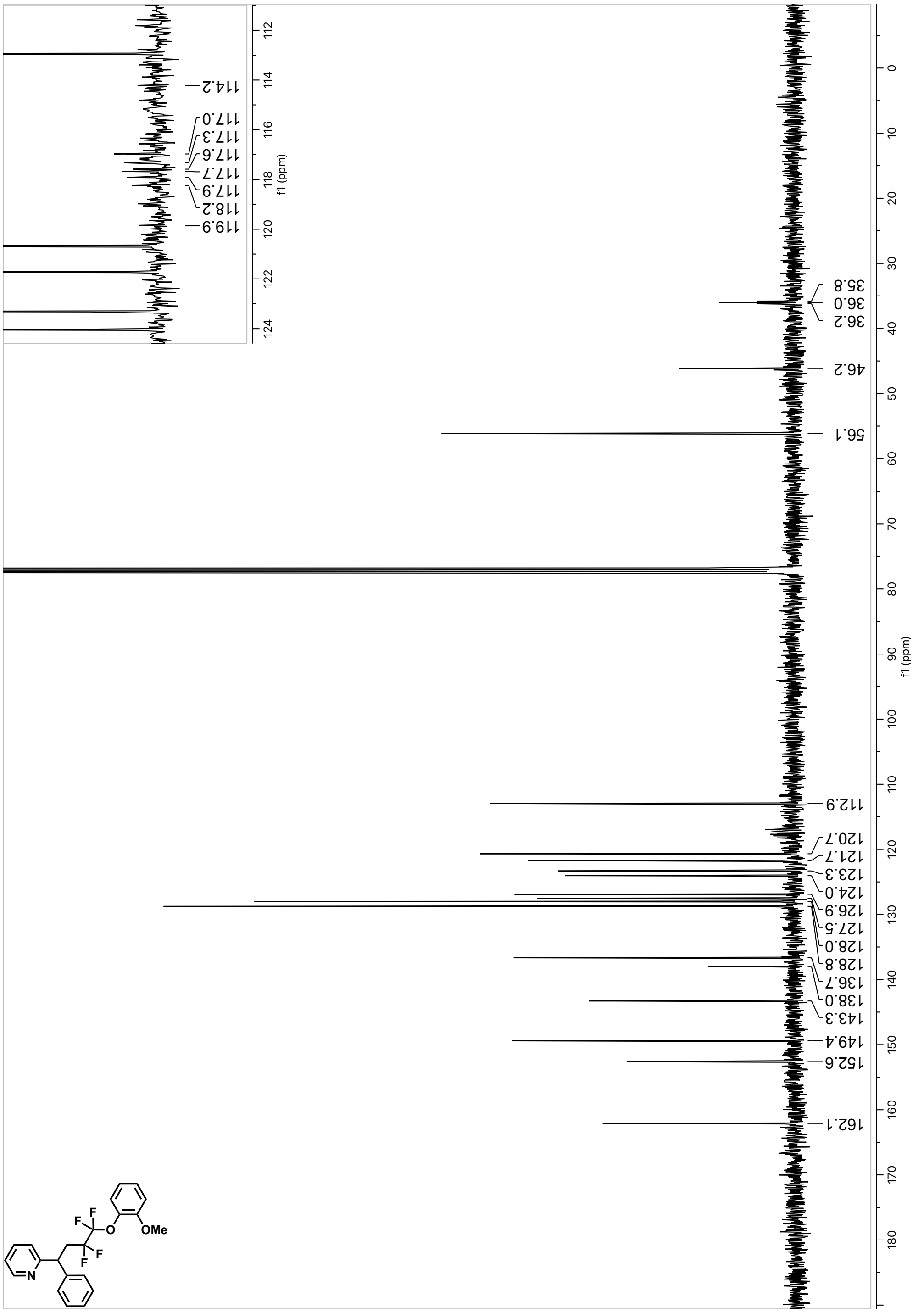

Compound 4c. ^{19}F NMR ($CDCl_3$, 376 MHz).

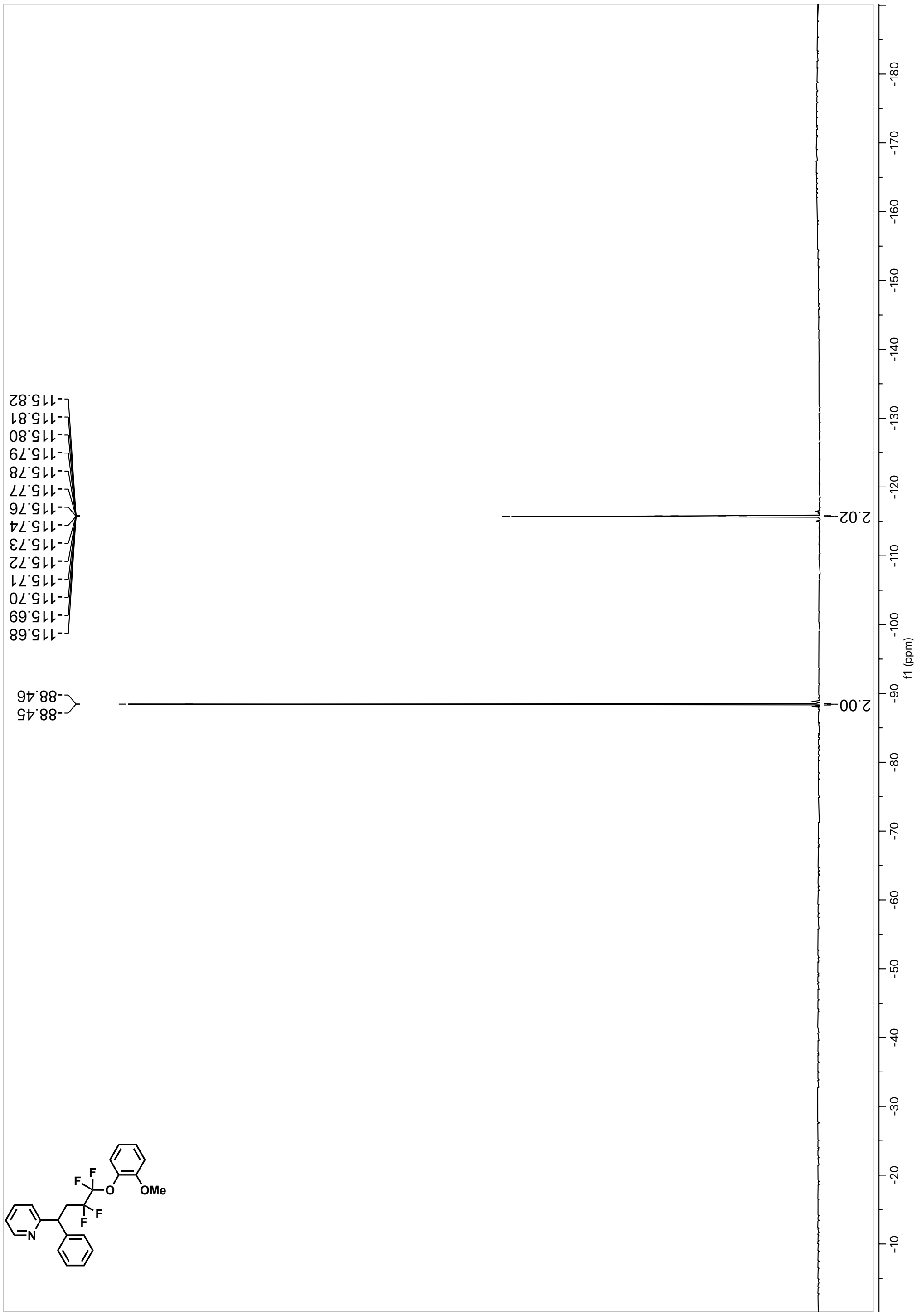

Compound 4d. ^{1}H NMR ($CDCl_3$, 400 MHz).

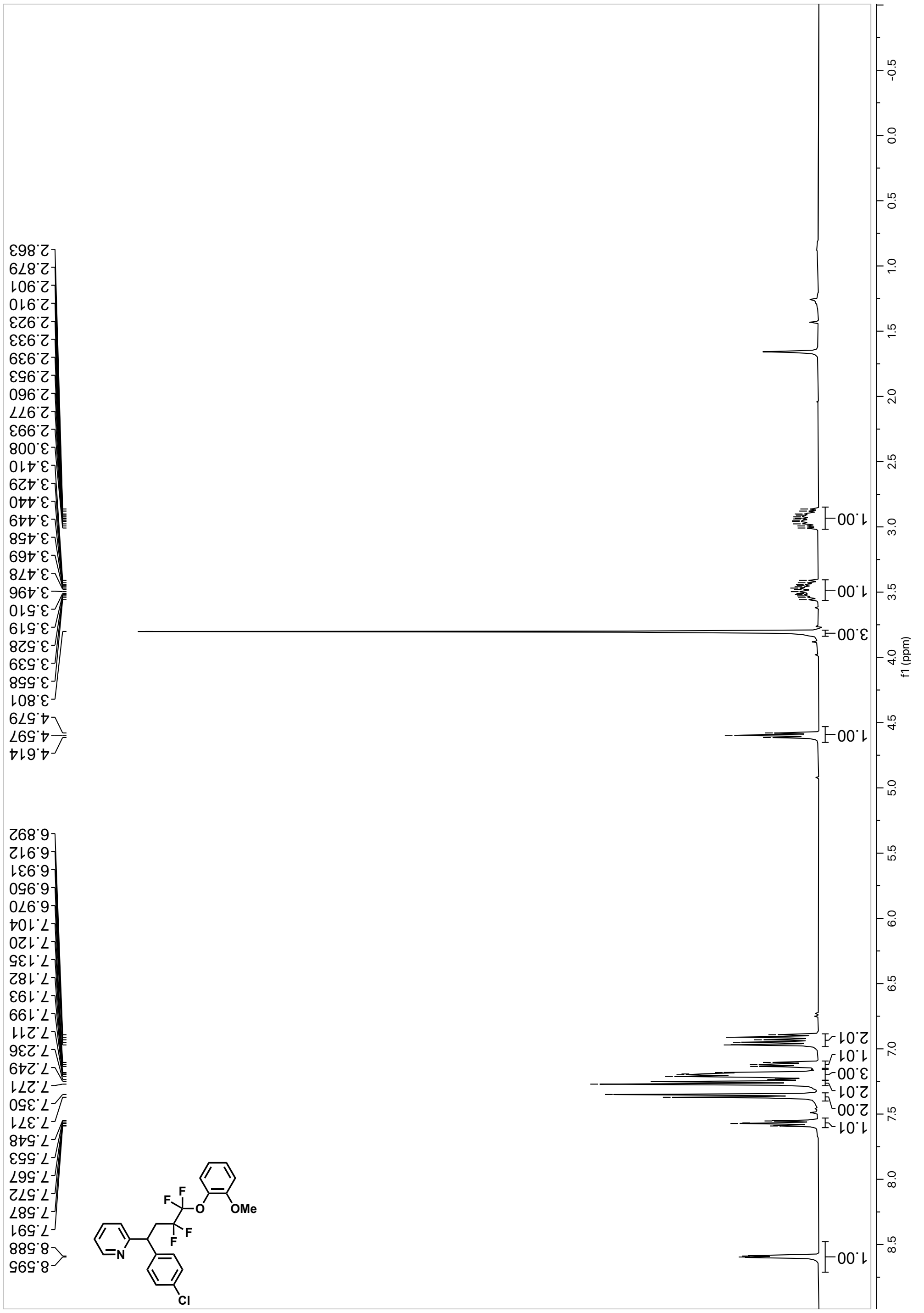

Compound 4d.^{13}C NMR ($CDCl_3$, 100 MHz).

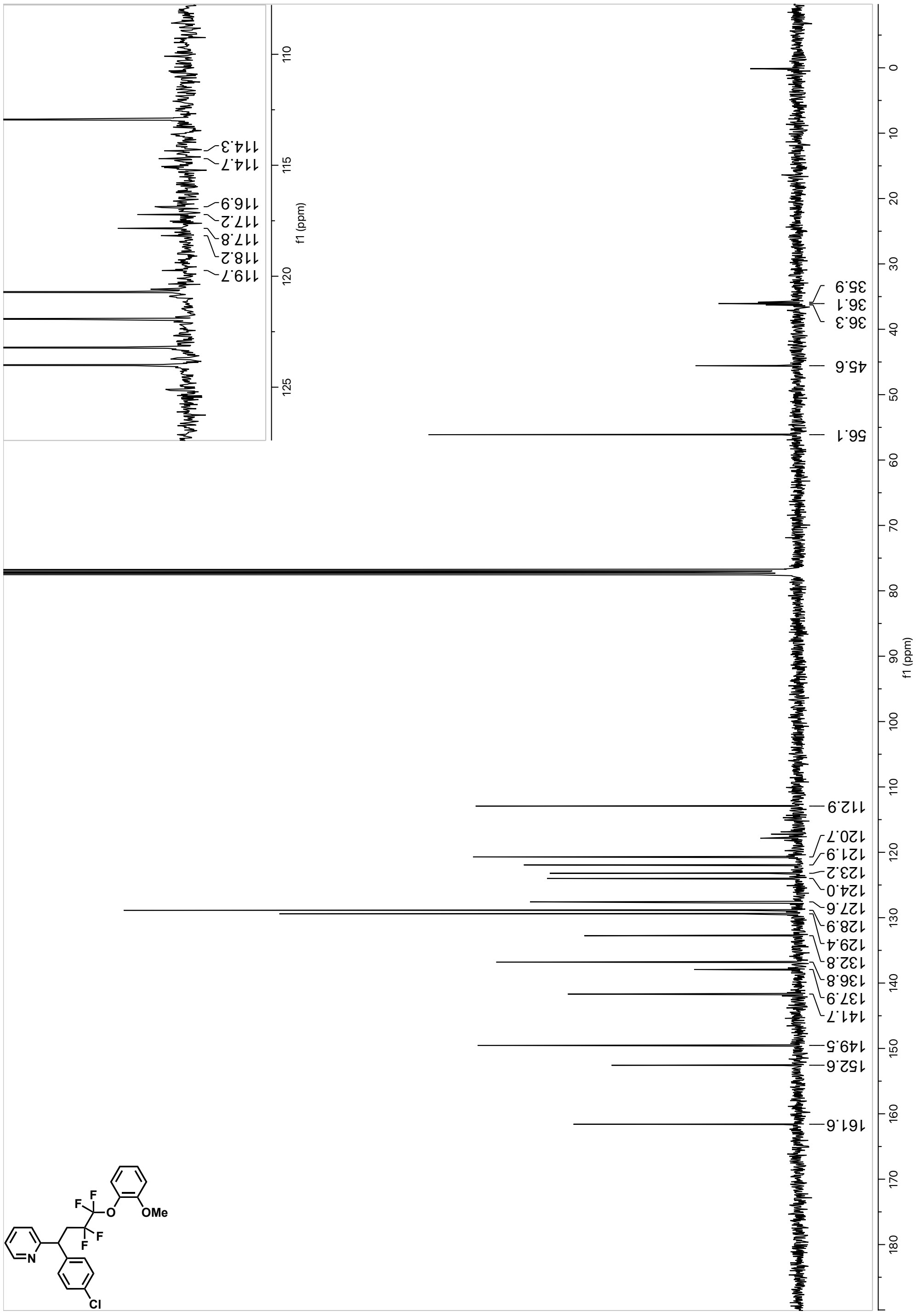

Compound 4d. ^{19}F NMR ($CDCl_3$, 376 MHz).

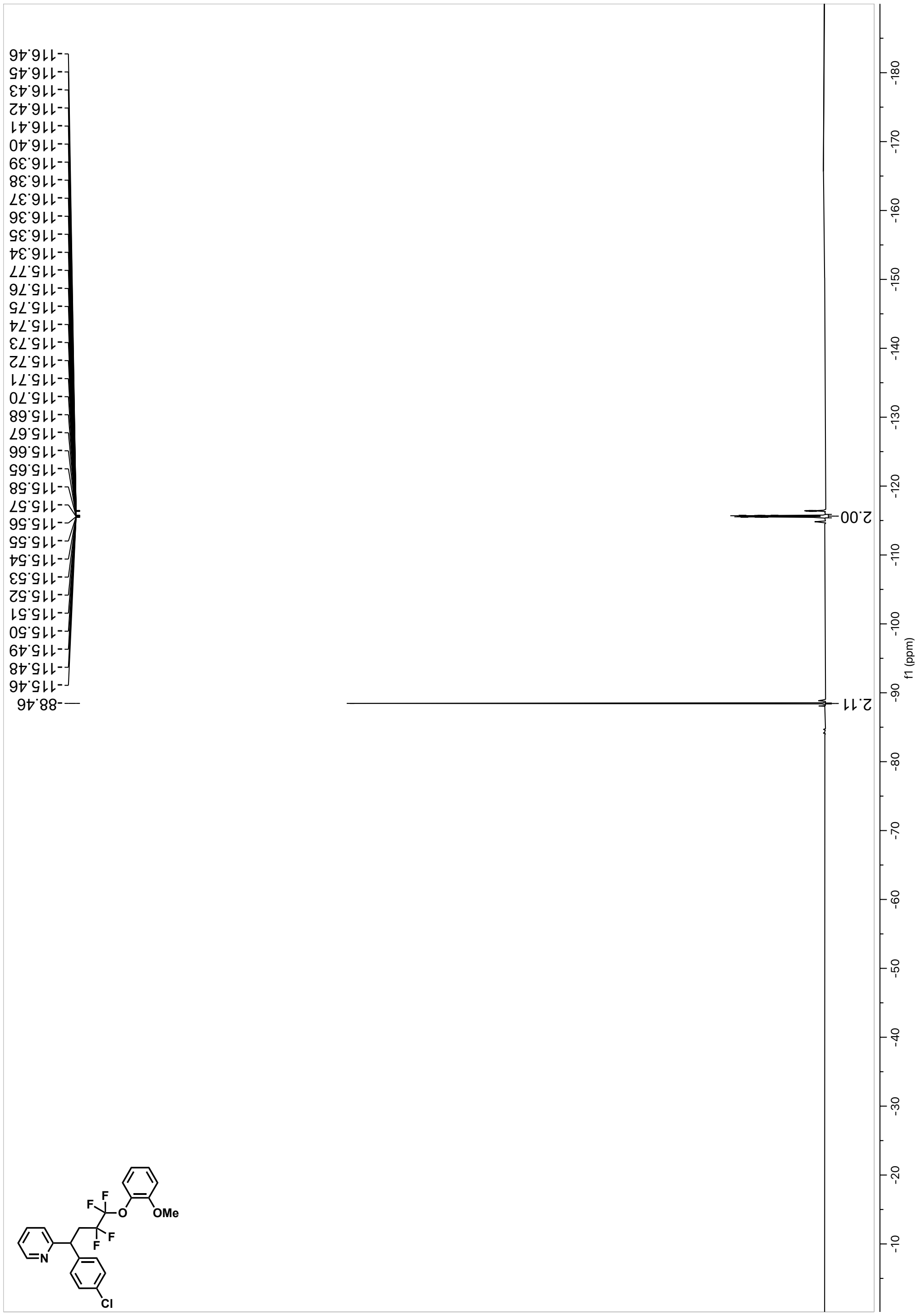

Compound 4e. ^{1}H NMR ($CDCl_3$, 400 MHz).

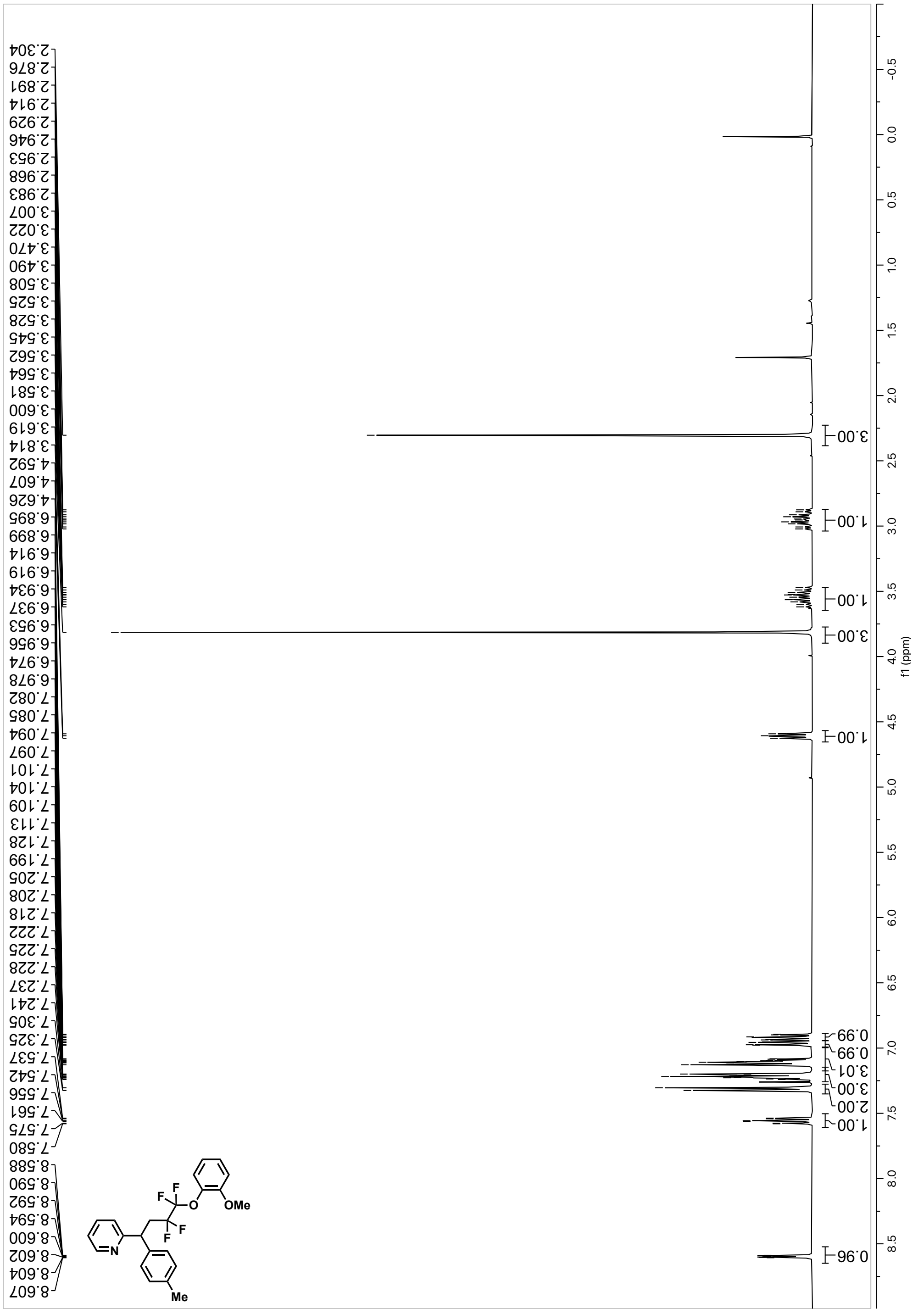

Compound 4e. ^{13}C NMR ($CDCl_3$, 100 MHz).

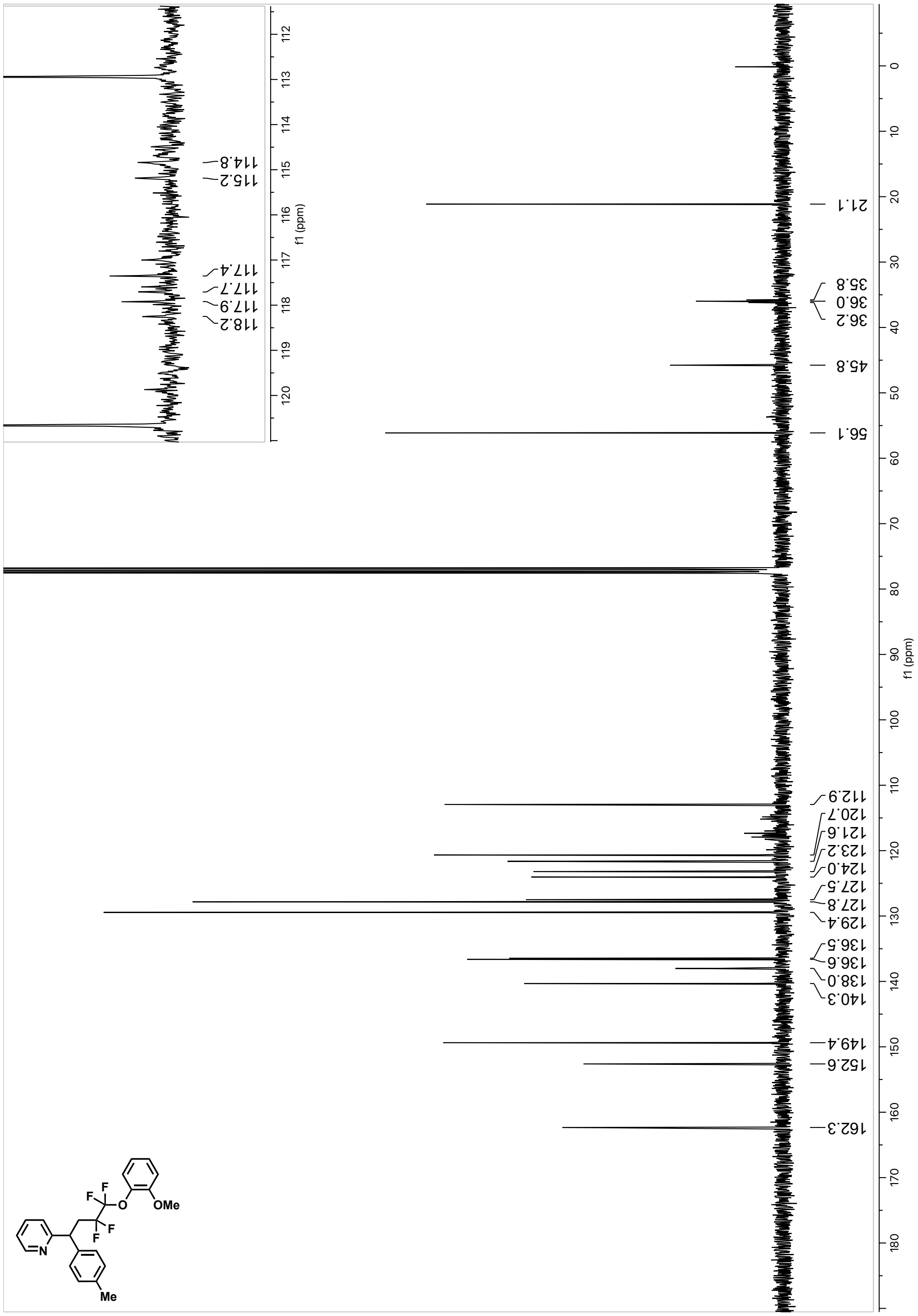

Compound 4e. ^{19}F NMR ($CDCl_3$, 376 MHz).

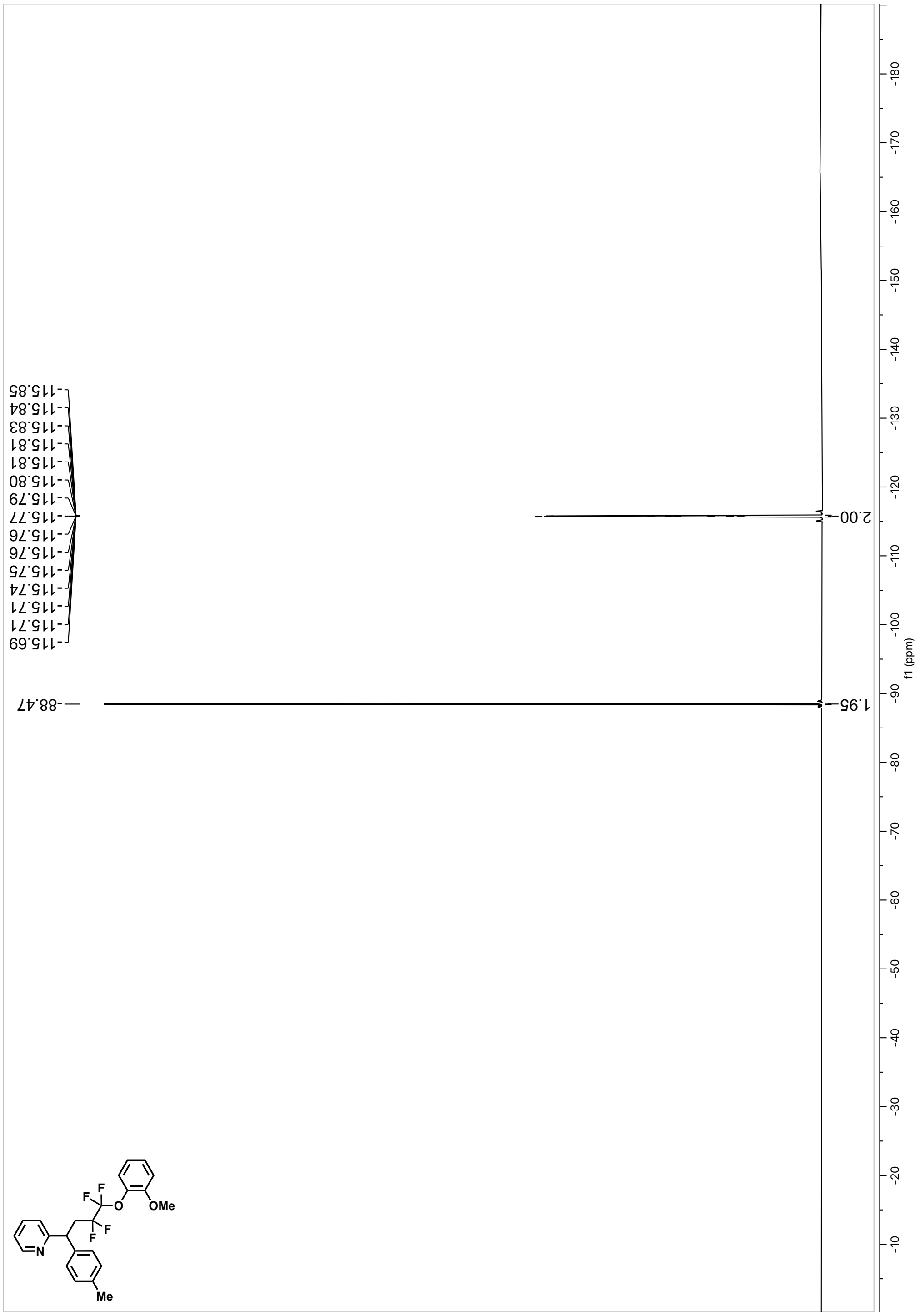

Compound 4f. ^{1}H NMR ($CDCl_3$, 400 MHz).

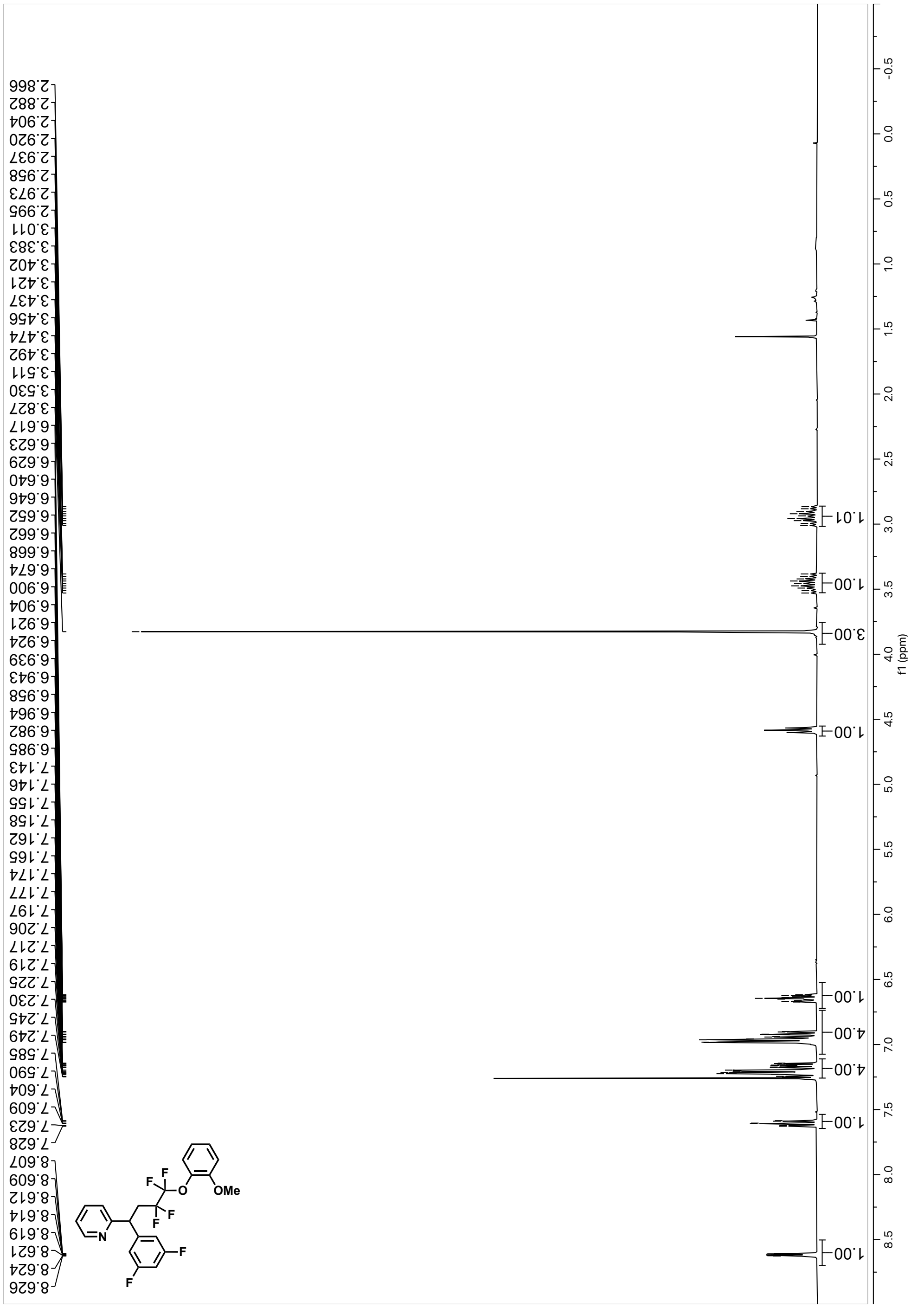

Compound 4f. ^{13}C NMR ($CDCl_3$, 100 MHz).

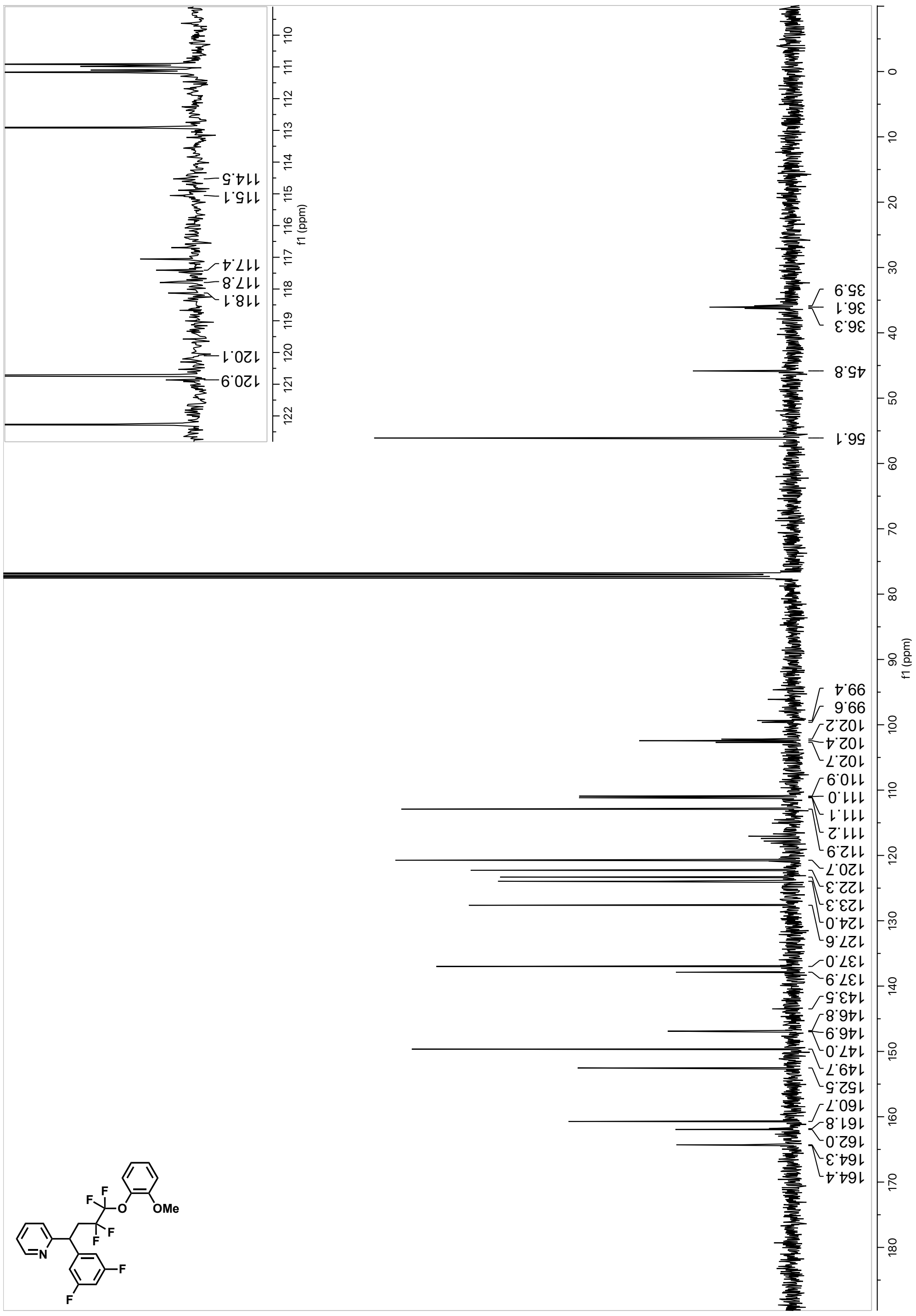

Compound 4f. ^{19}F NMR ($CDCl_3$, 376 MHz).

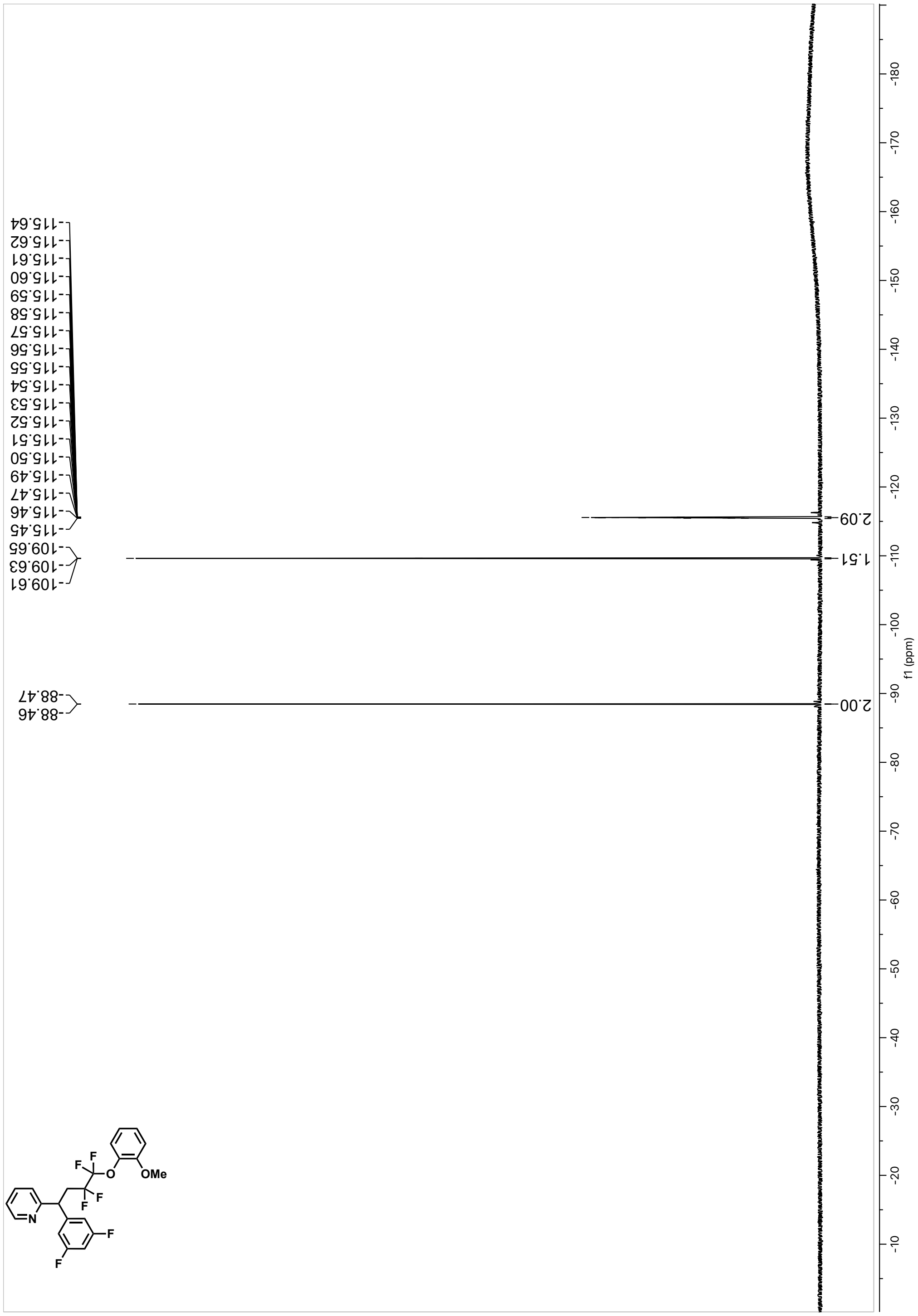

Compound 4g.: ^{1}H NMR ($CDCl_3$, 400 MHz).

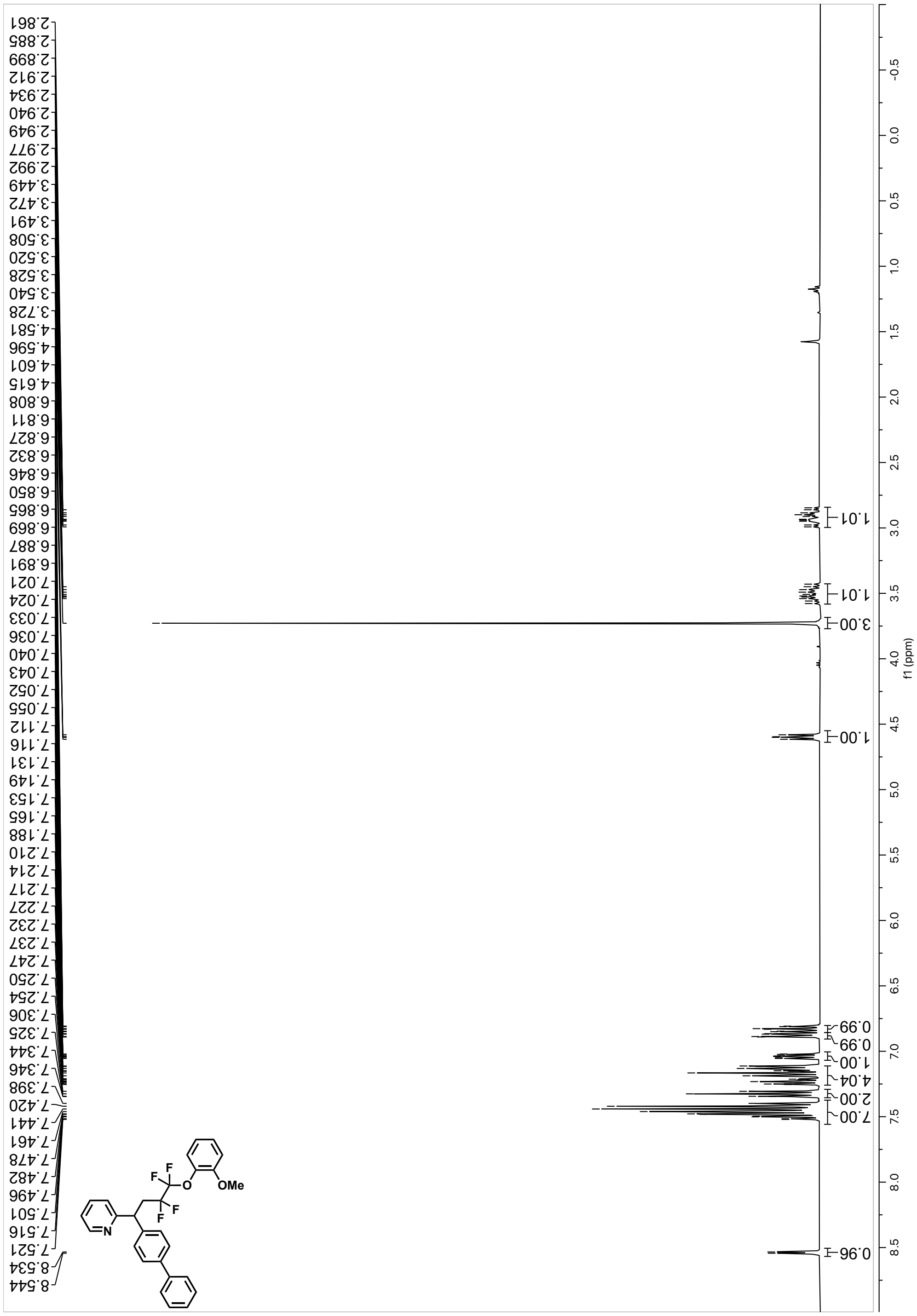

Compound 4g. ^{13}C NMR ($CDCl_3$, 100 MHz).

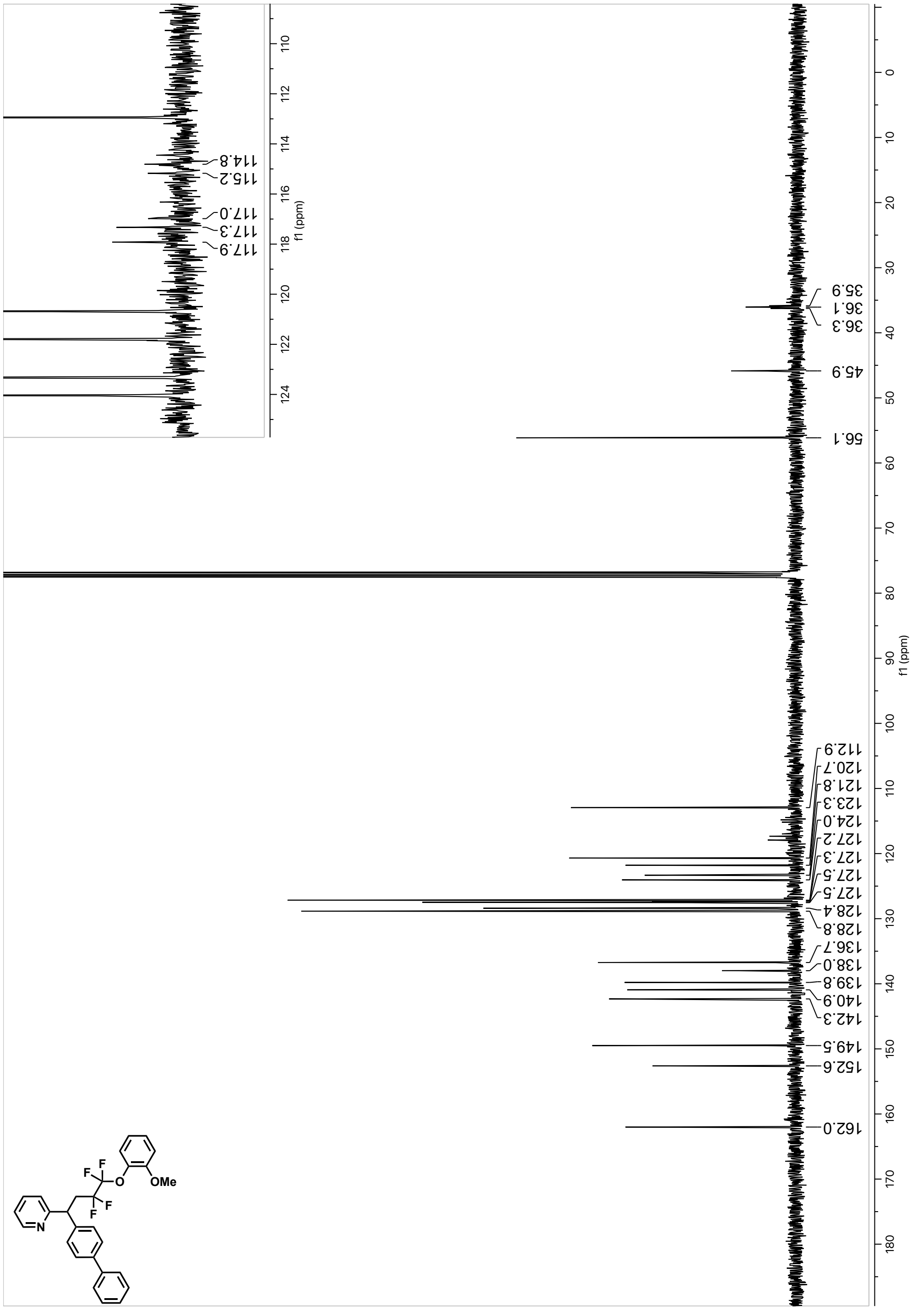

Compound 4g. ^{19}F NMR ($CDCl_3$, 376 MHz).

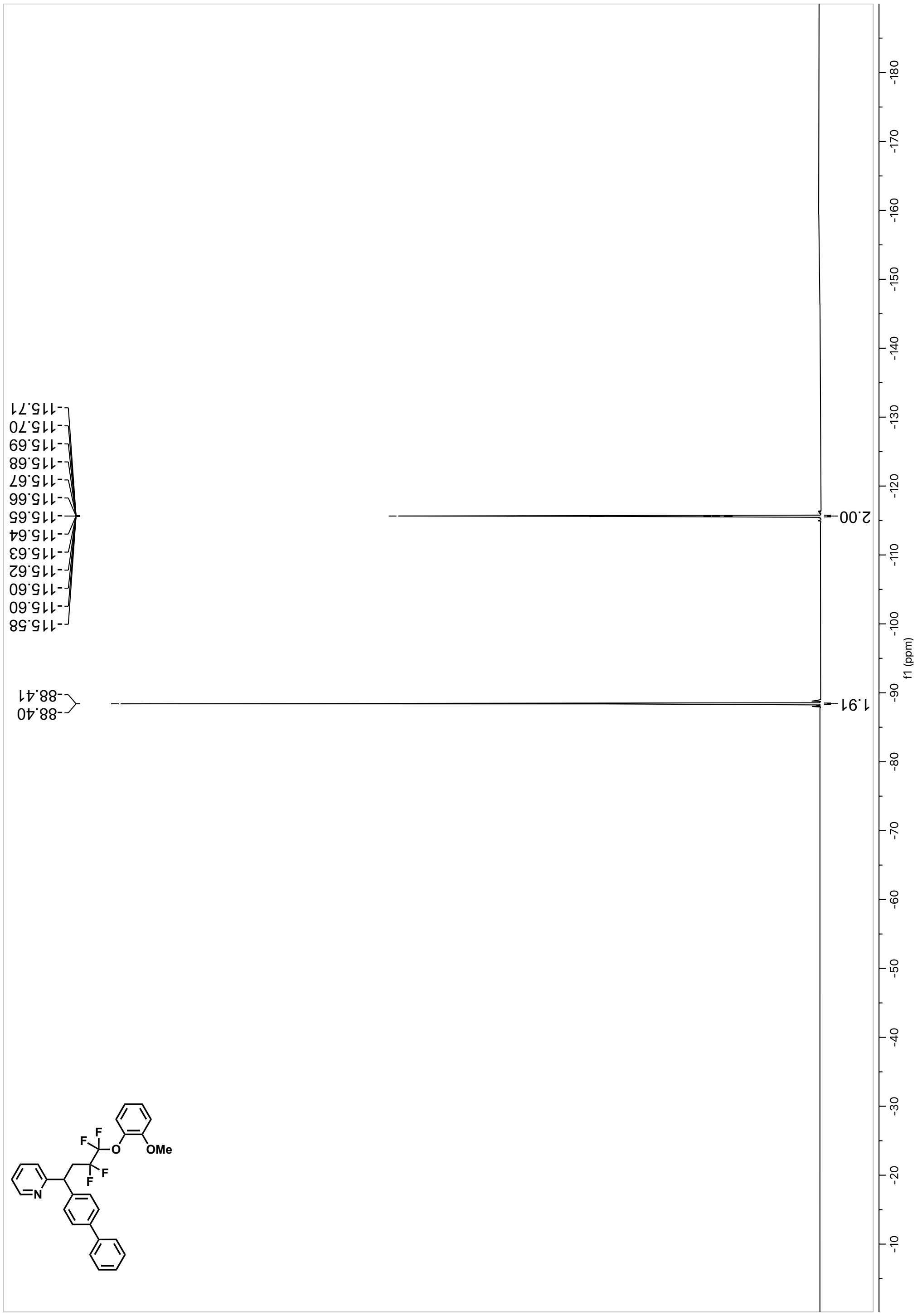

Compound 4h. ^{1}H NMR ($CDCl_3$, 400 MHz).

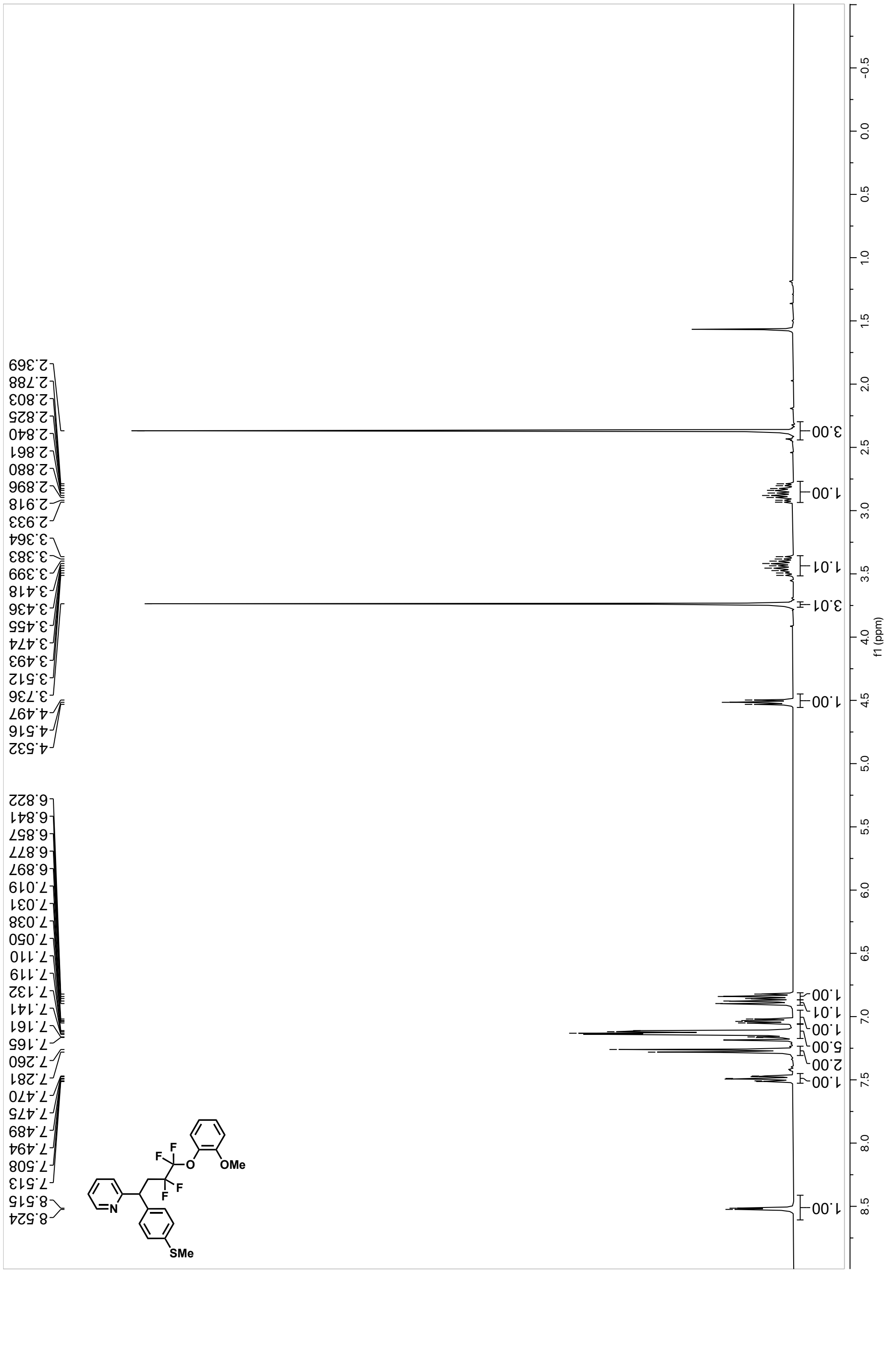

Compound 4h. ^{13}C NMR ($CDCl_3$, 100 MHz).

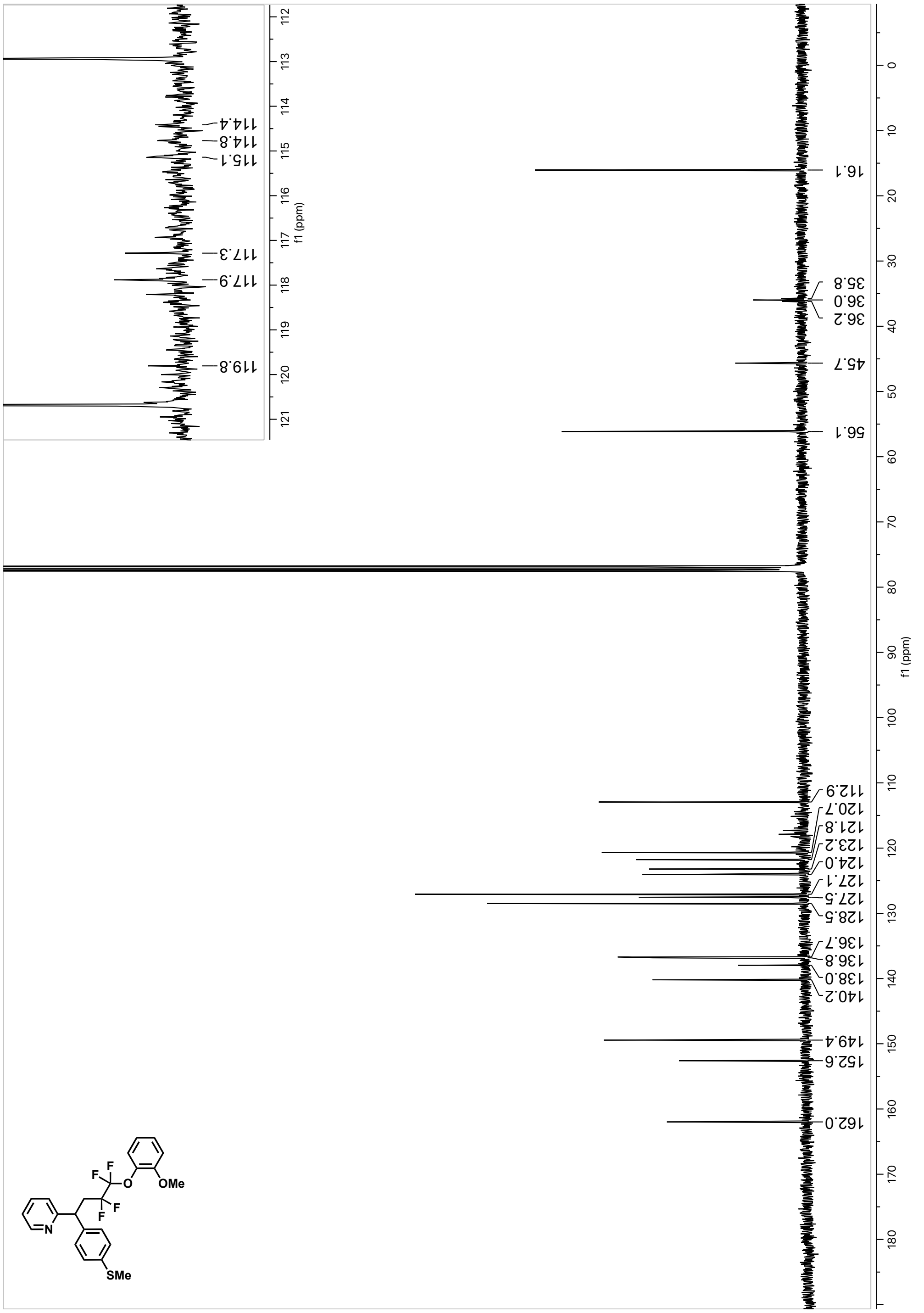

Compound 4h. ^{19}F NMR ($CDCl_3$, 376 MHz).

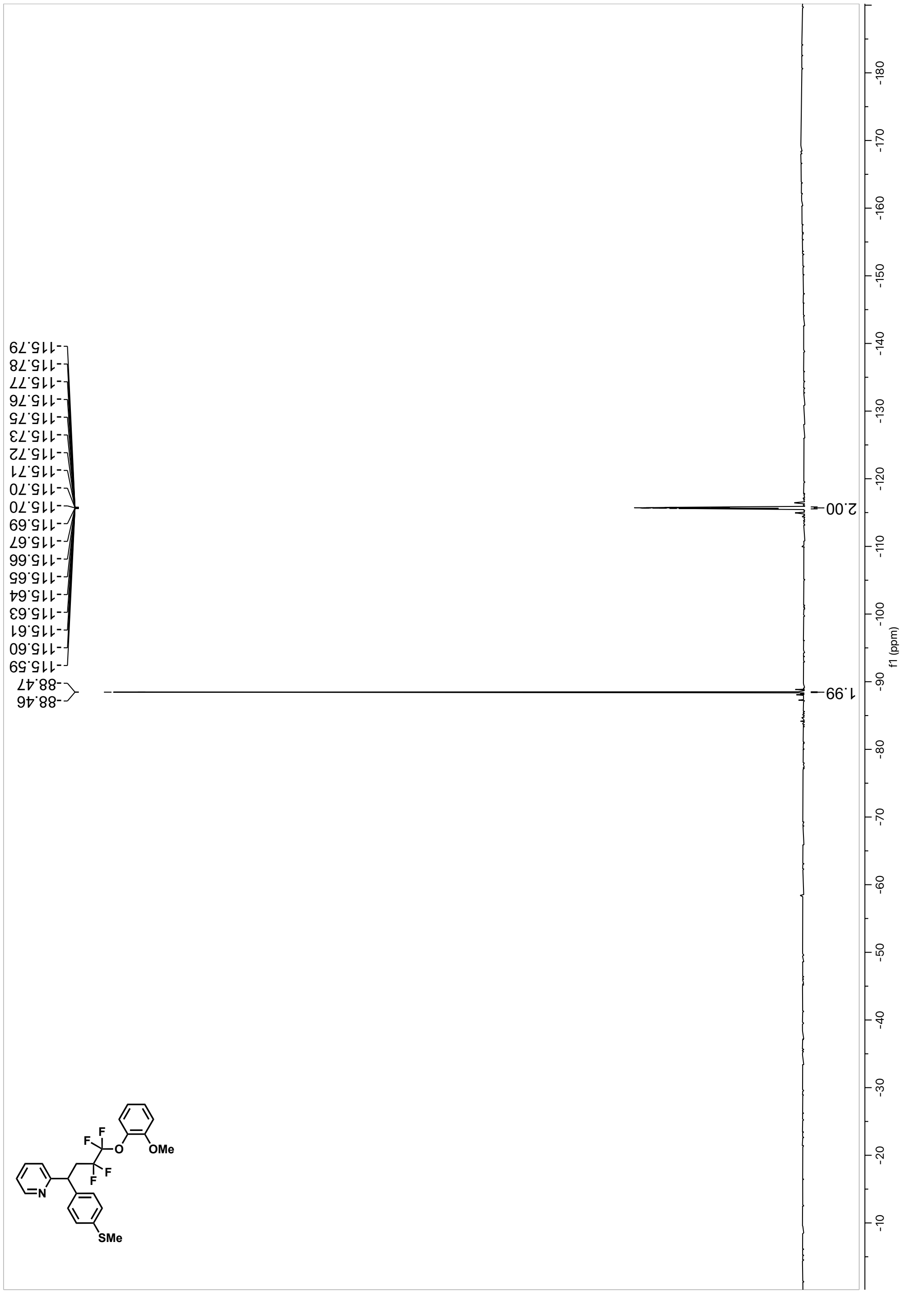

Compound 4i. ^{1}H NMR ($CDCl_3$, 400 MHz).

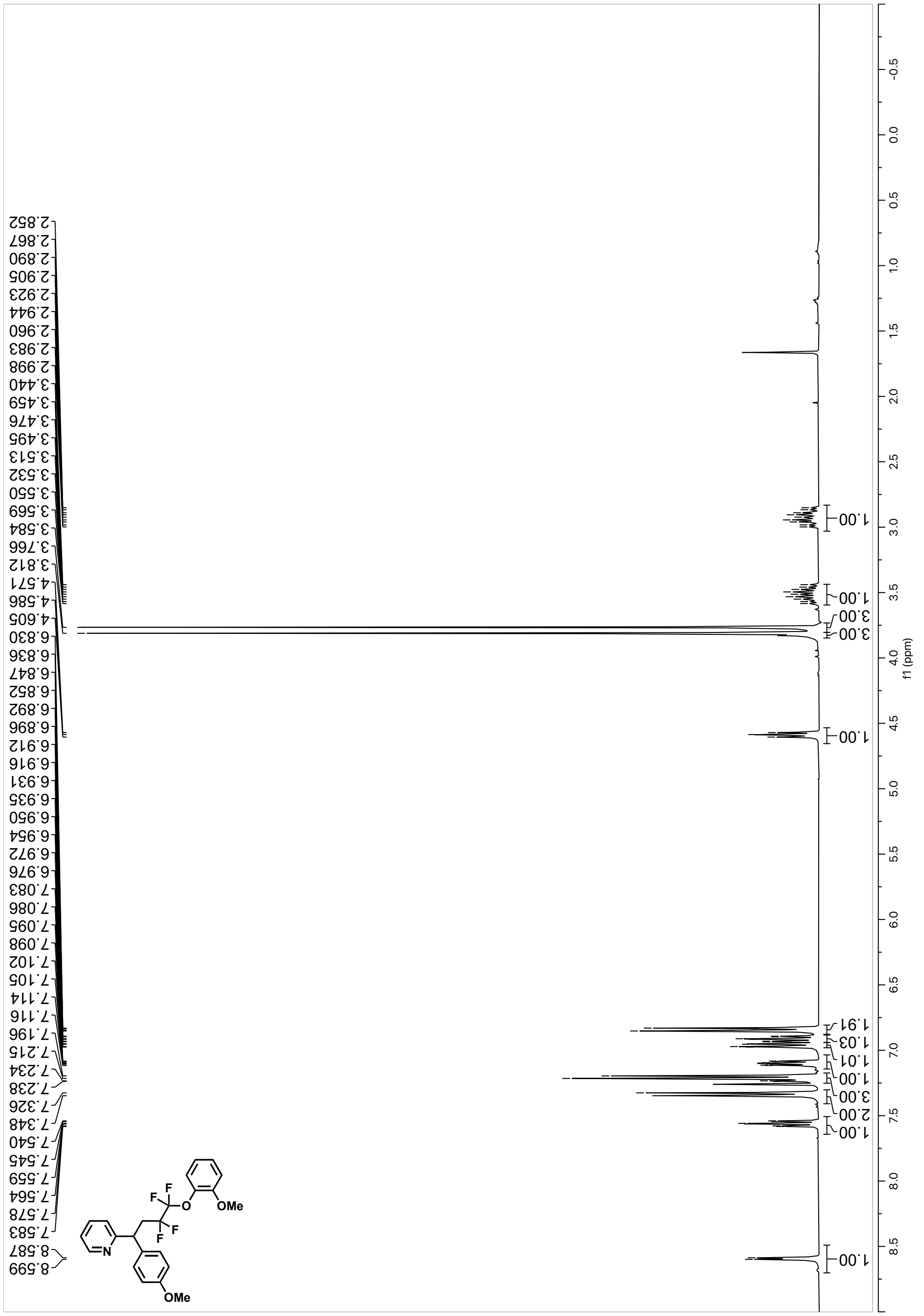

Compound 4i. ^{13}C NMR ($CDCl_3$, 100 MHz).

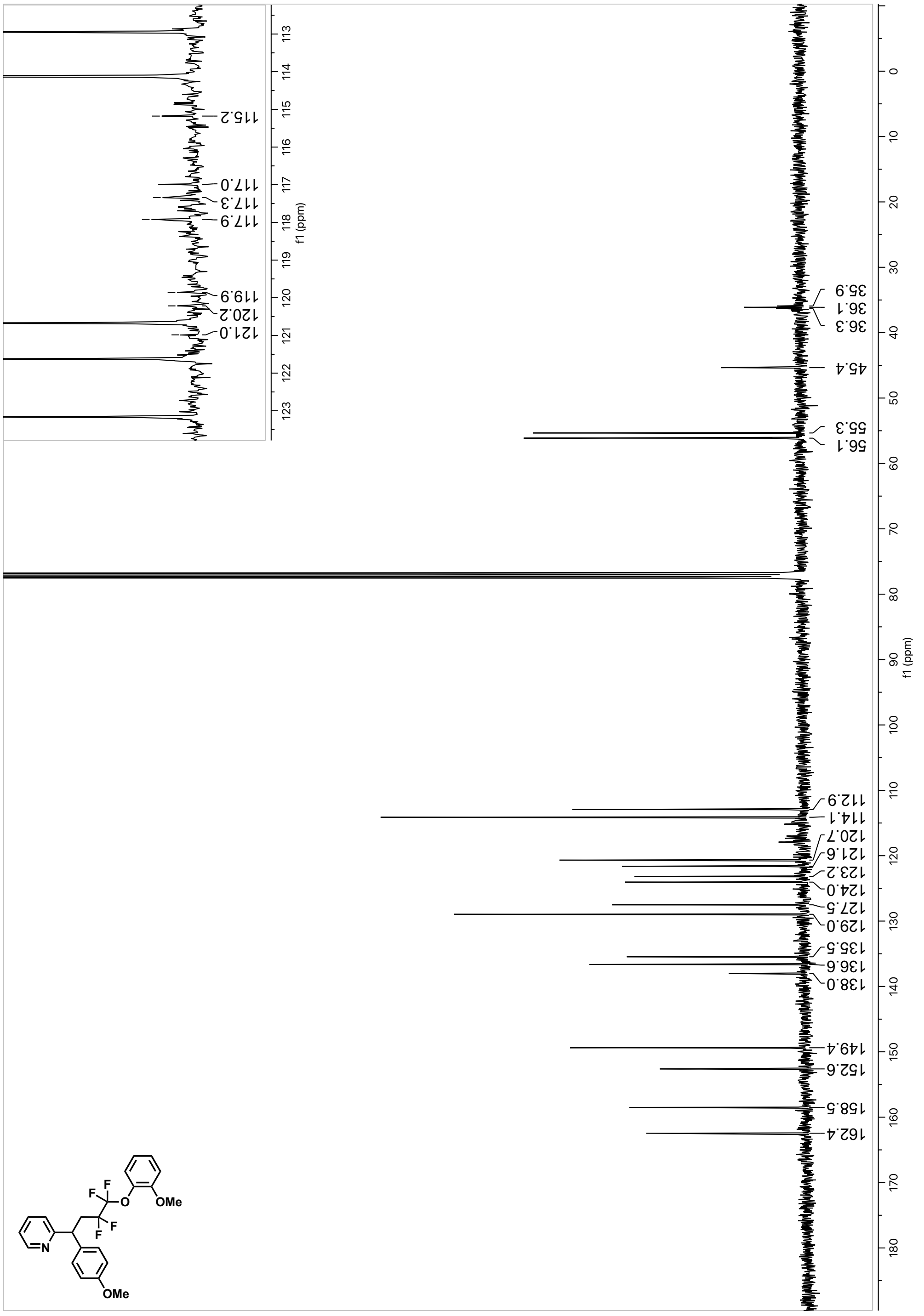

Compound 4i. ^{19}F NMR ($CDCl_3$, 376 MHz).

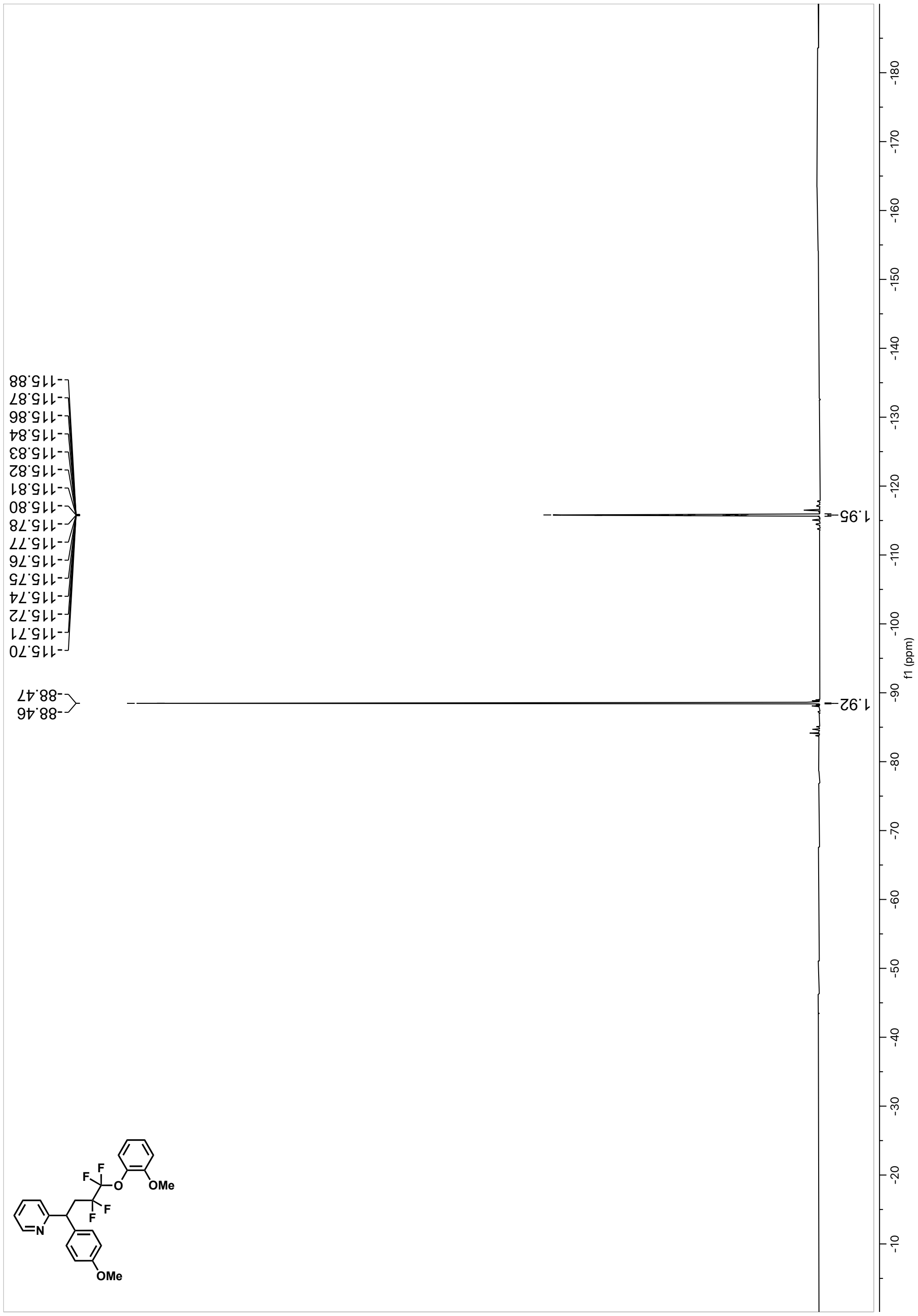

Compound 4j. ^{1}H NMR ($CDCl_3$, 400 MHz).

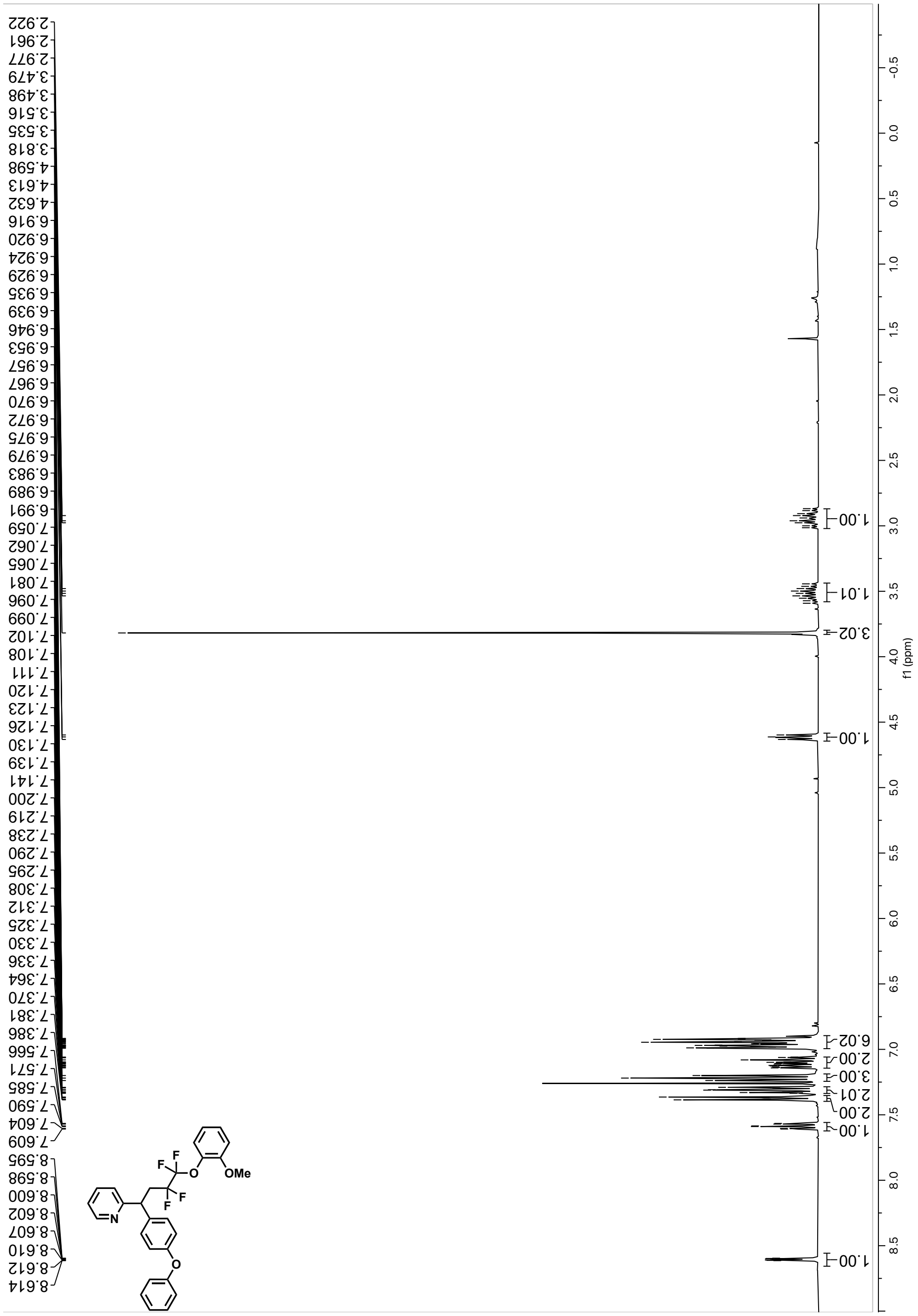

Compound 4j. ^{13}C NMR ($CDCl_3$, 100 MHz).

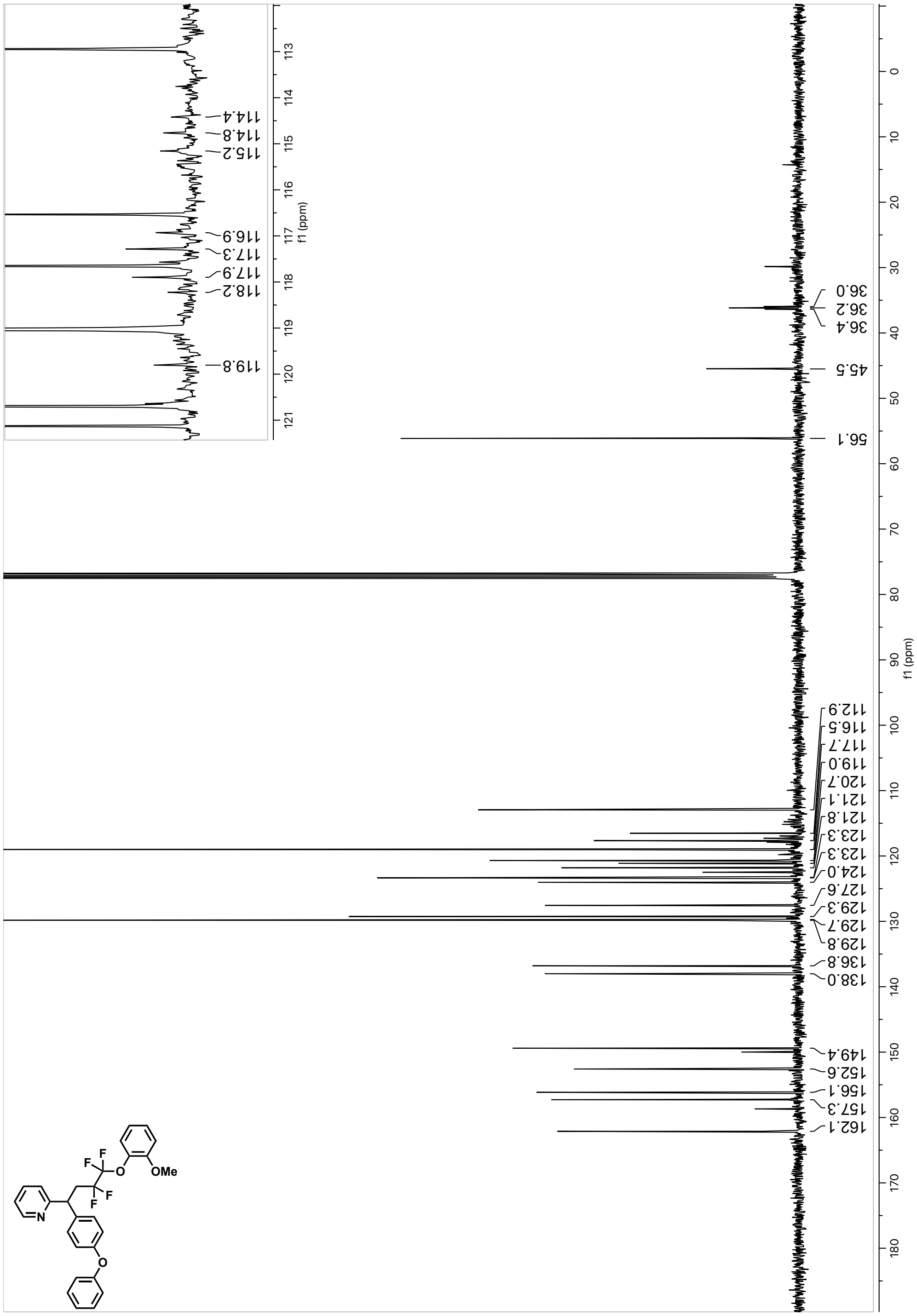

Compound 4j. ^{19}F NMR ($CDCl_3$, 376 MHz).

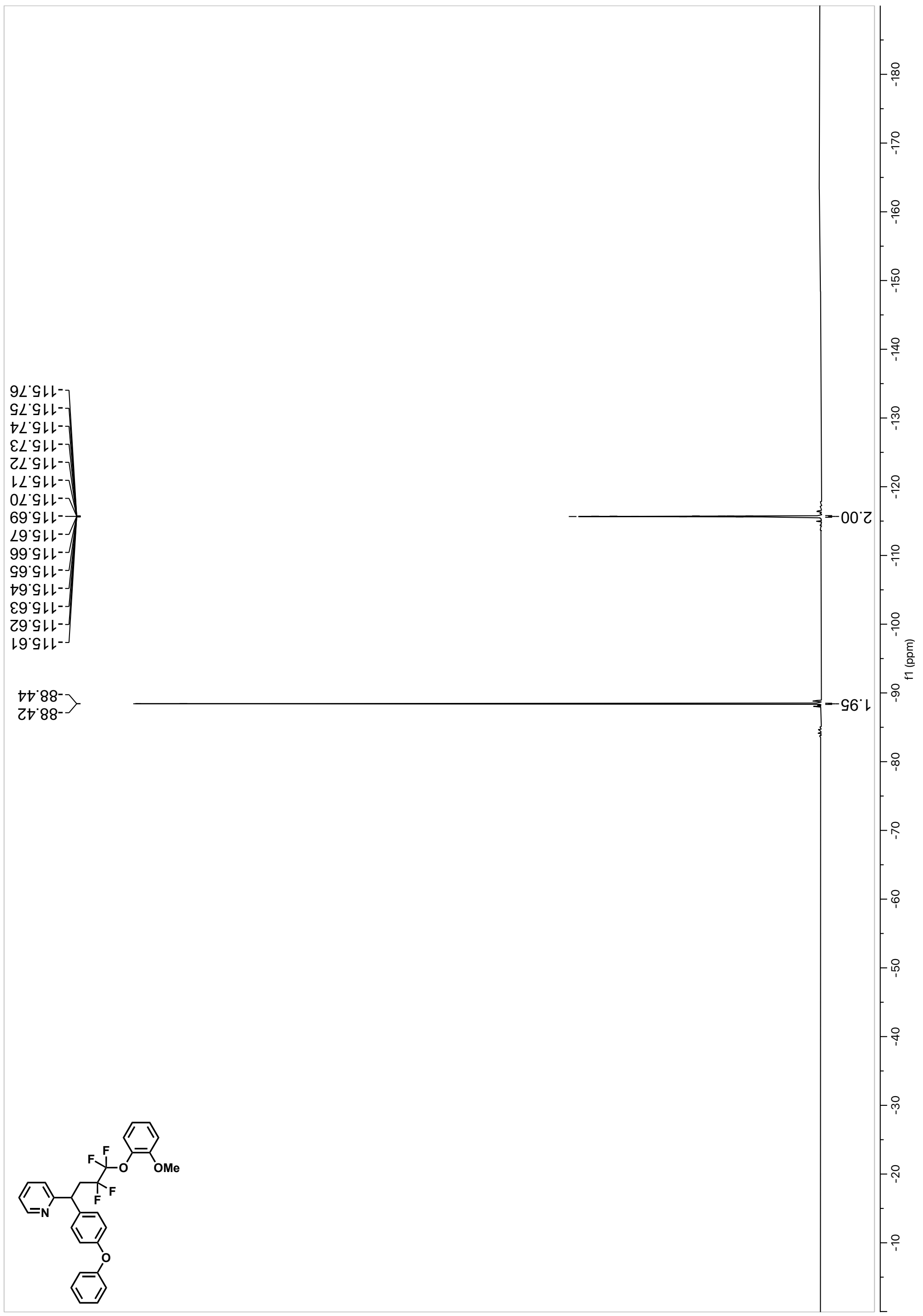

Compound 4k. ^{1}H NMR ($CDCl_3$, 400 MHz).

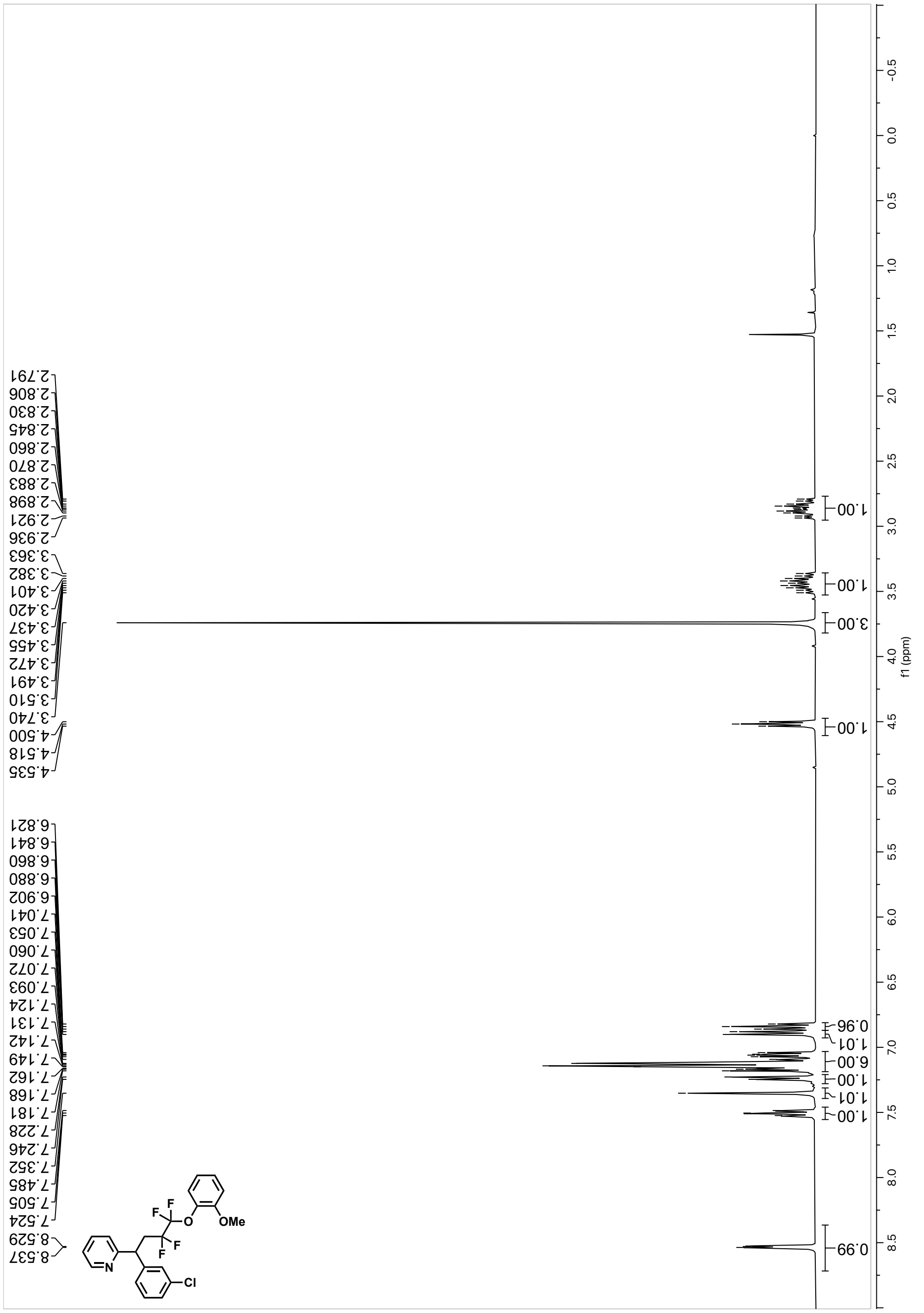

Compound 4k. ^{13}C NMR ($CDCl_3$, 100 MHz).

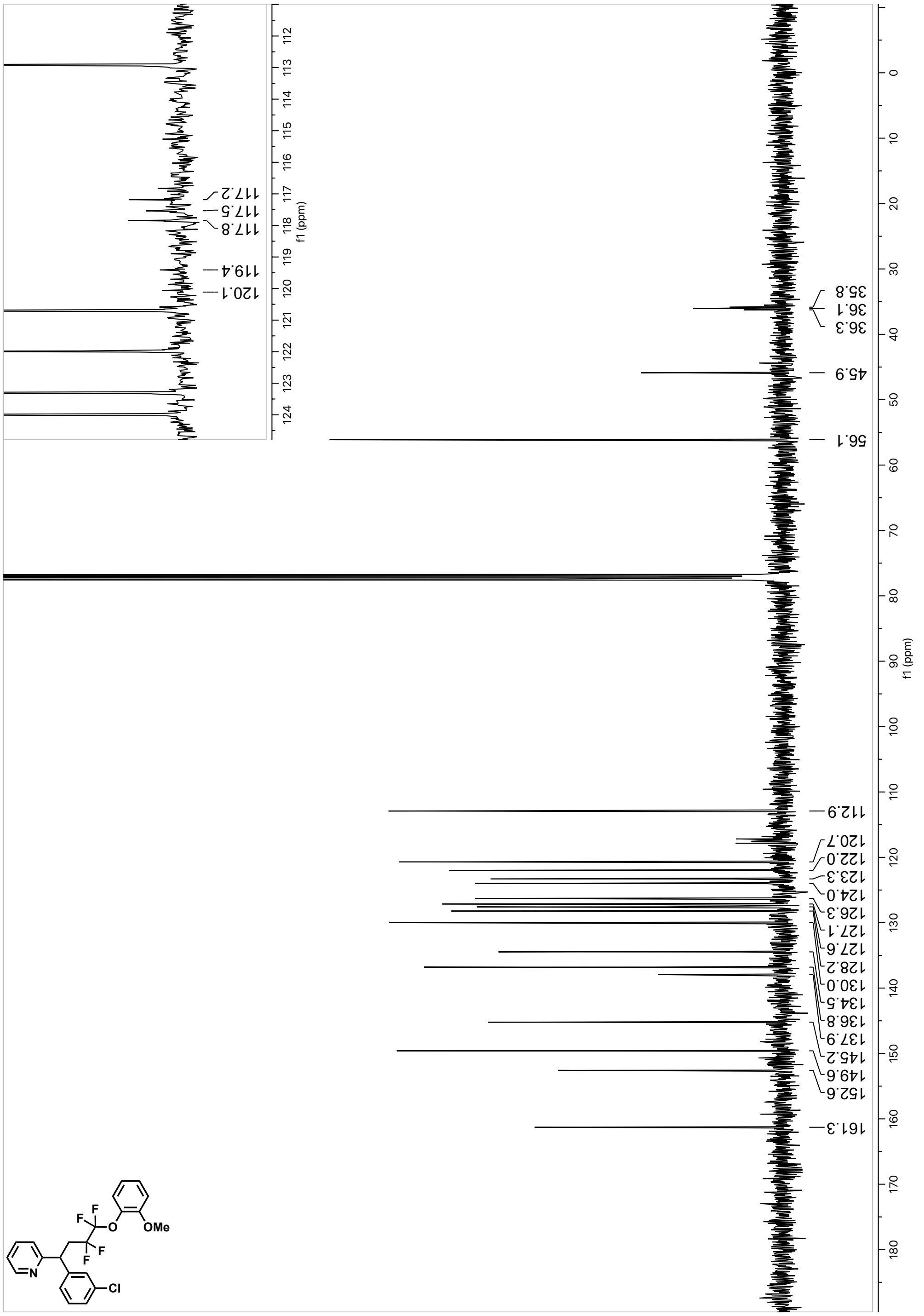

Compound 4k. ^{19}F NMR ($CDCl_3$, 376 MHz).

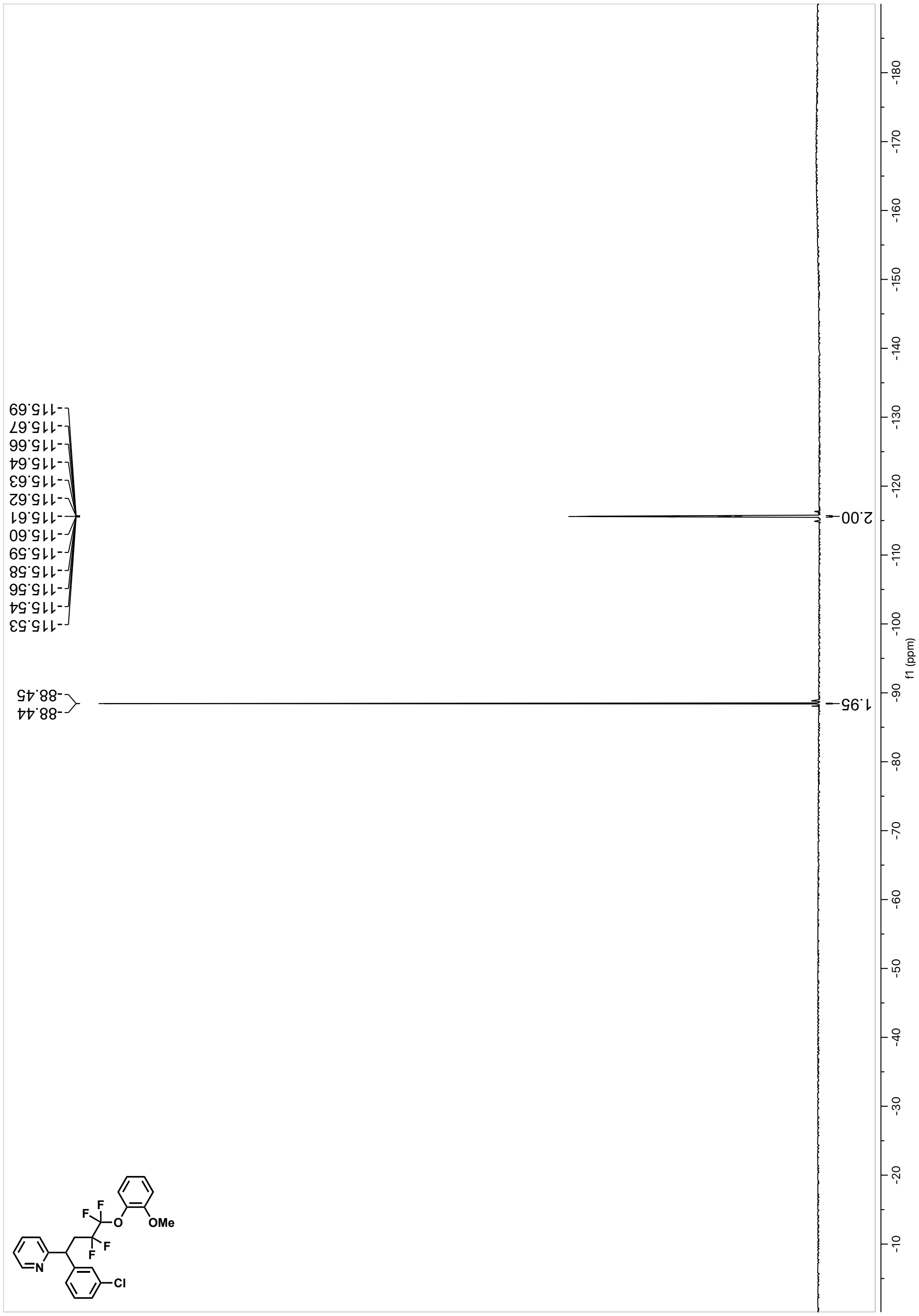

Compound 4l. ^{1}H NMR ($CDCl_3$, 400 MHz).

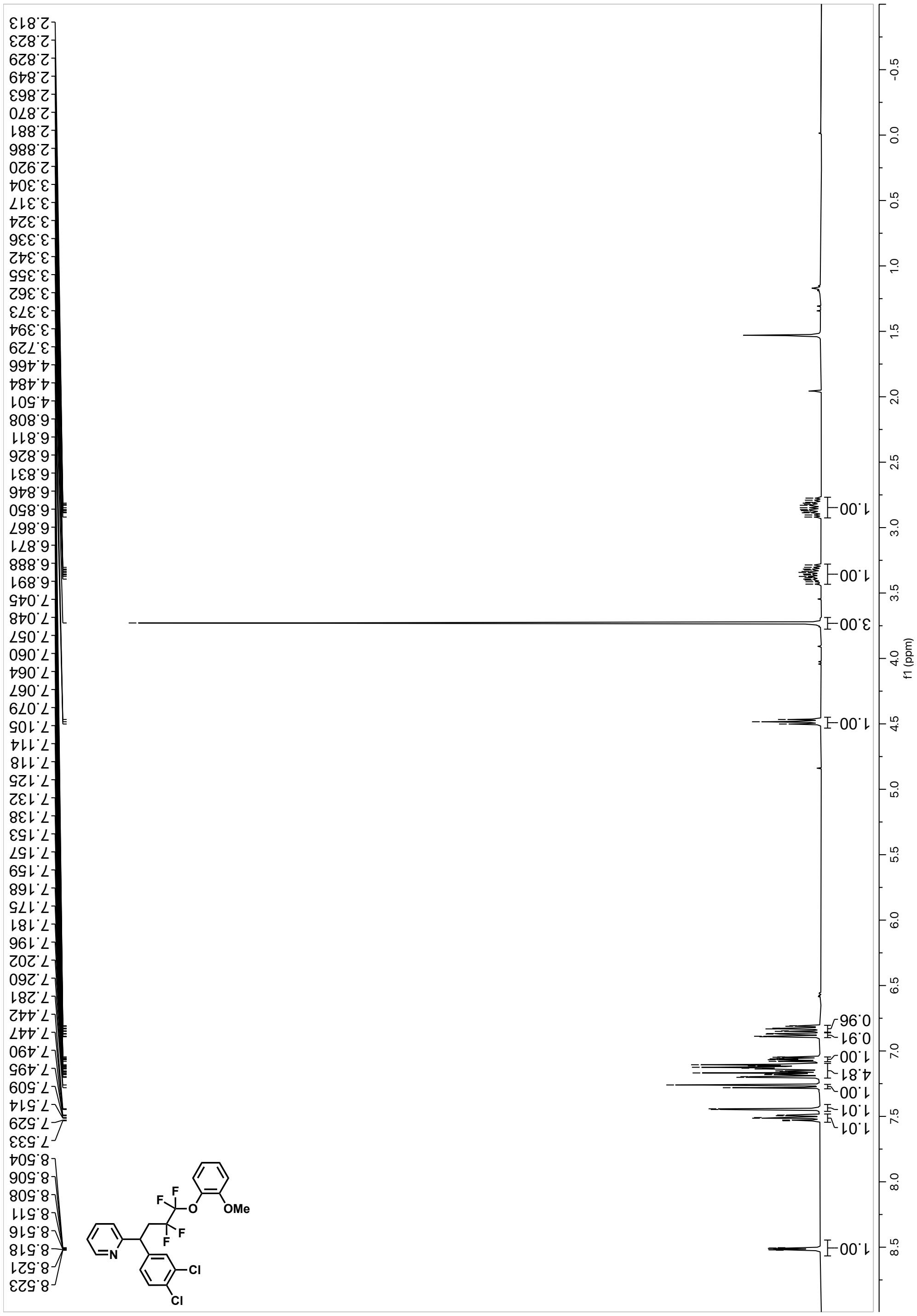

Compound 4l. ^{13}C NMR ($CDCl_3$, 100 MHz).

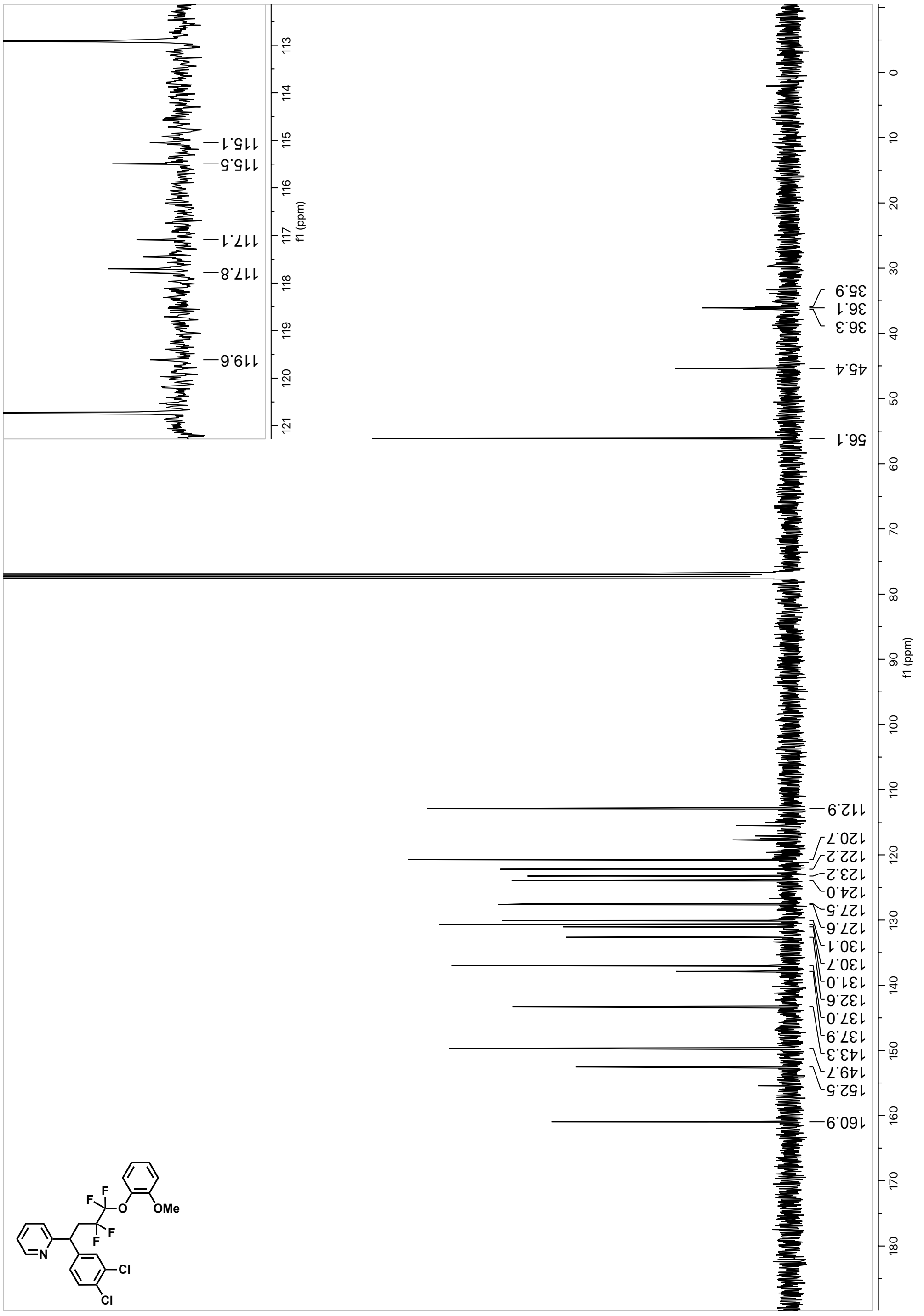

Compound 4l. ^{19}F NMR (CDCl$_3$, 376 MHz).

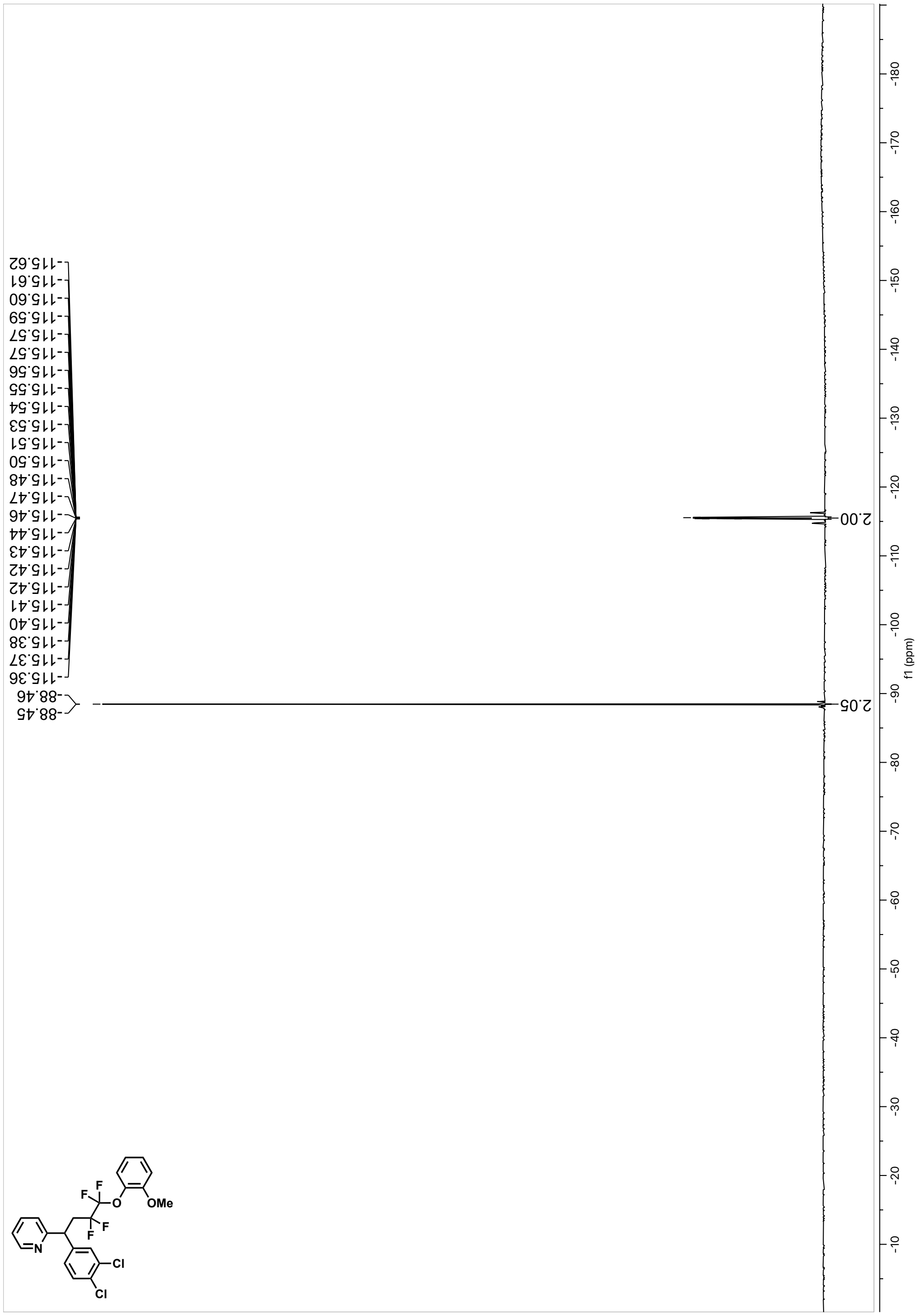

Compound 4m. ^{1}H NMR ($CDCl_3$, 400 MHz).

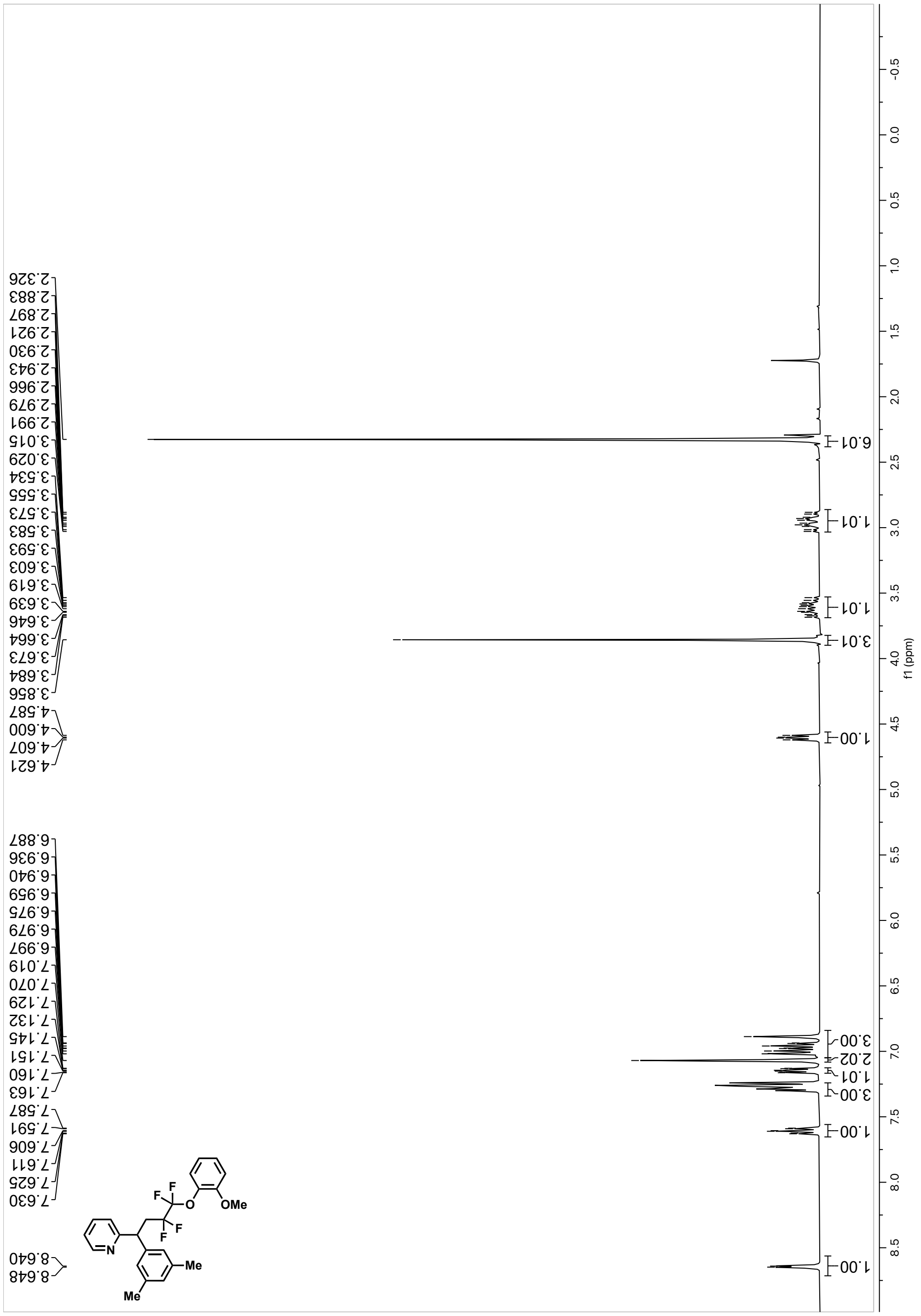

Compound 4m. ^{13}C NMR ($CDCl_3$, 100 MHz).

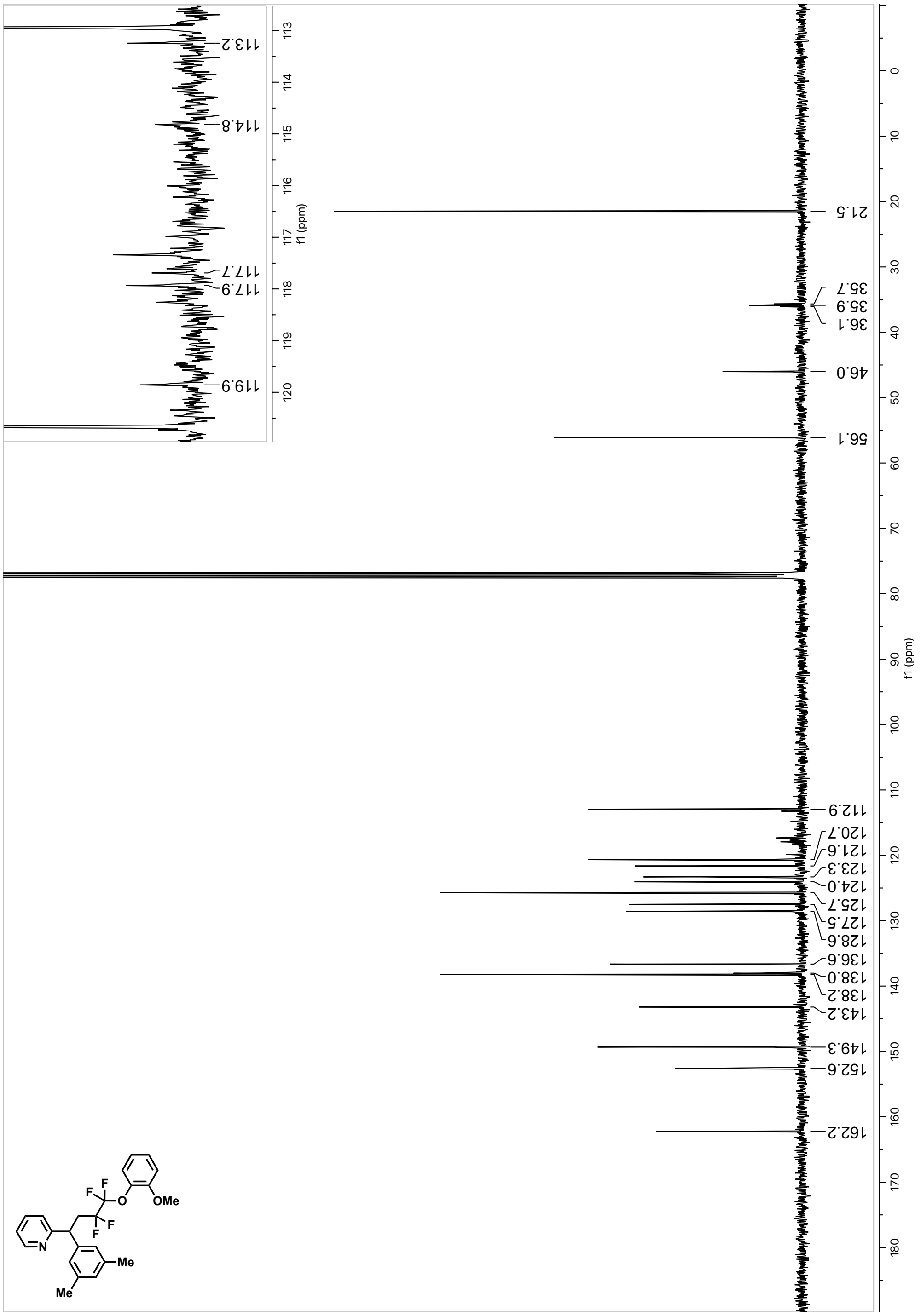

Compound 4m. ^{19}F NMR ($CDCl_3$, 376 MHz).

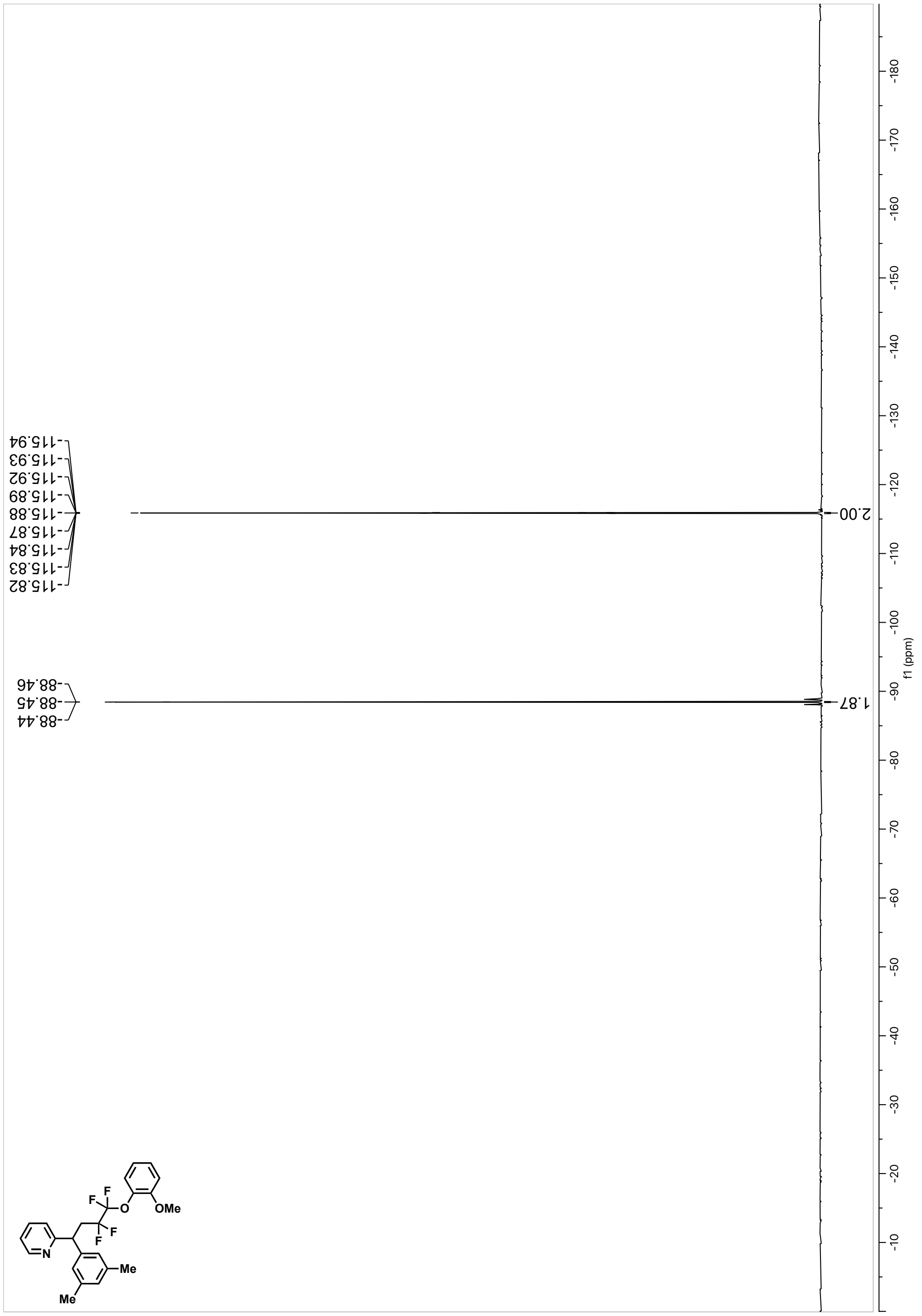

Compound 4n. ^{1}H NMR ($CDCl_3$, 400 MHz).

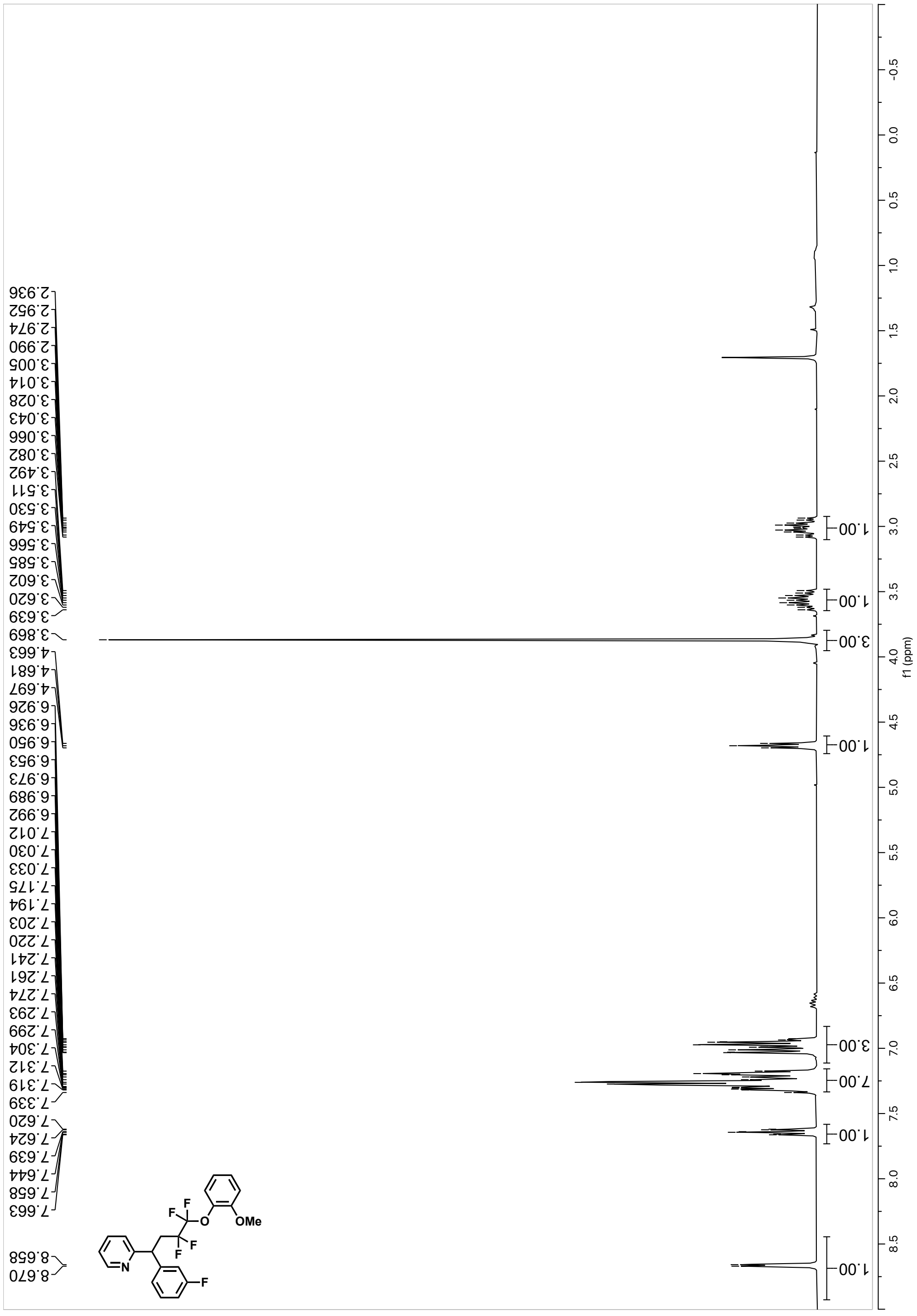

Compound 4n. ^{13}C NMR ($CDCl_3$, 100 MHz).

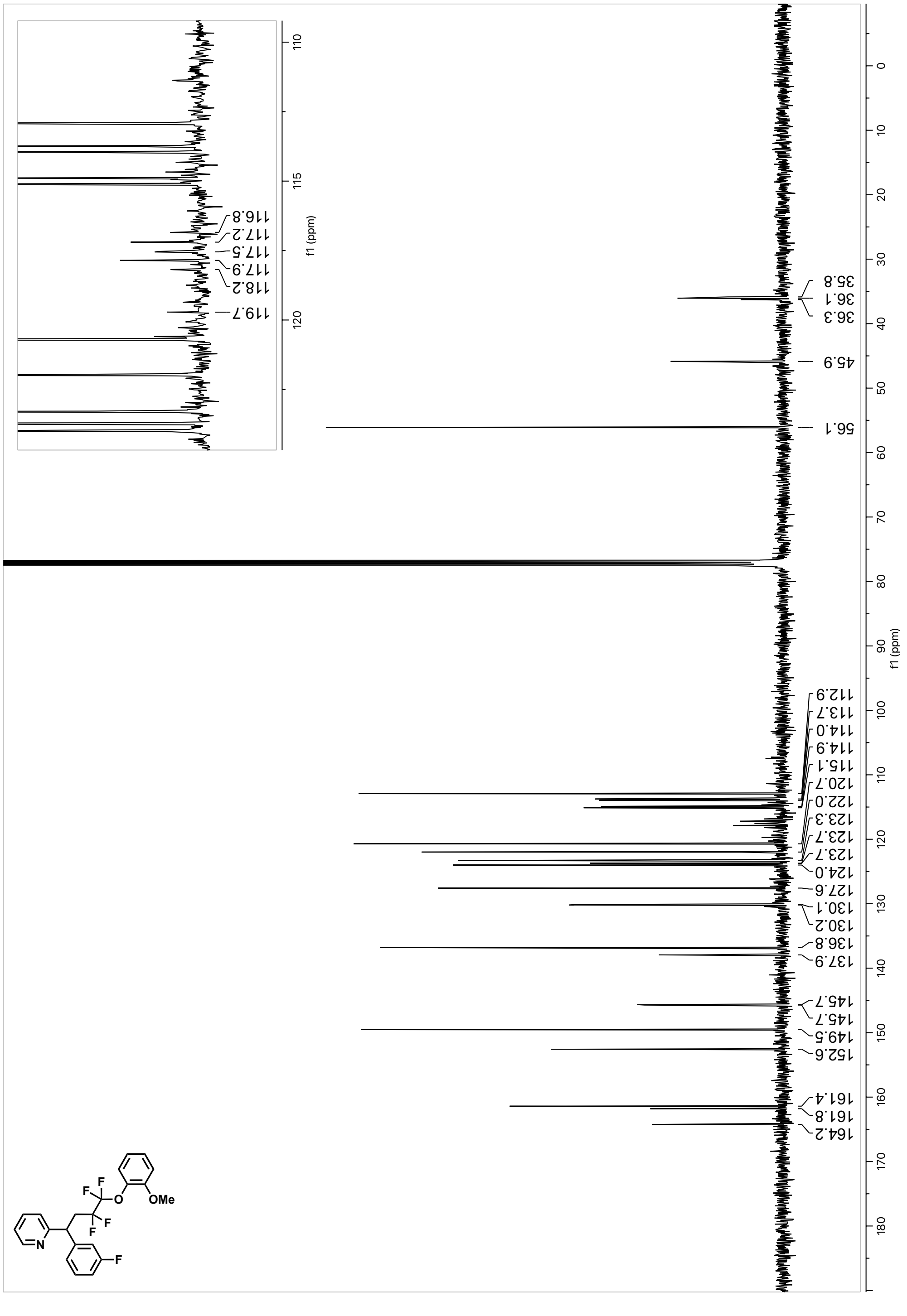

Compound 4n. ^{19}F NMR ($CDCl_3$, 376 MHz).

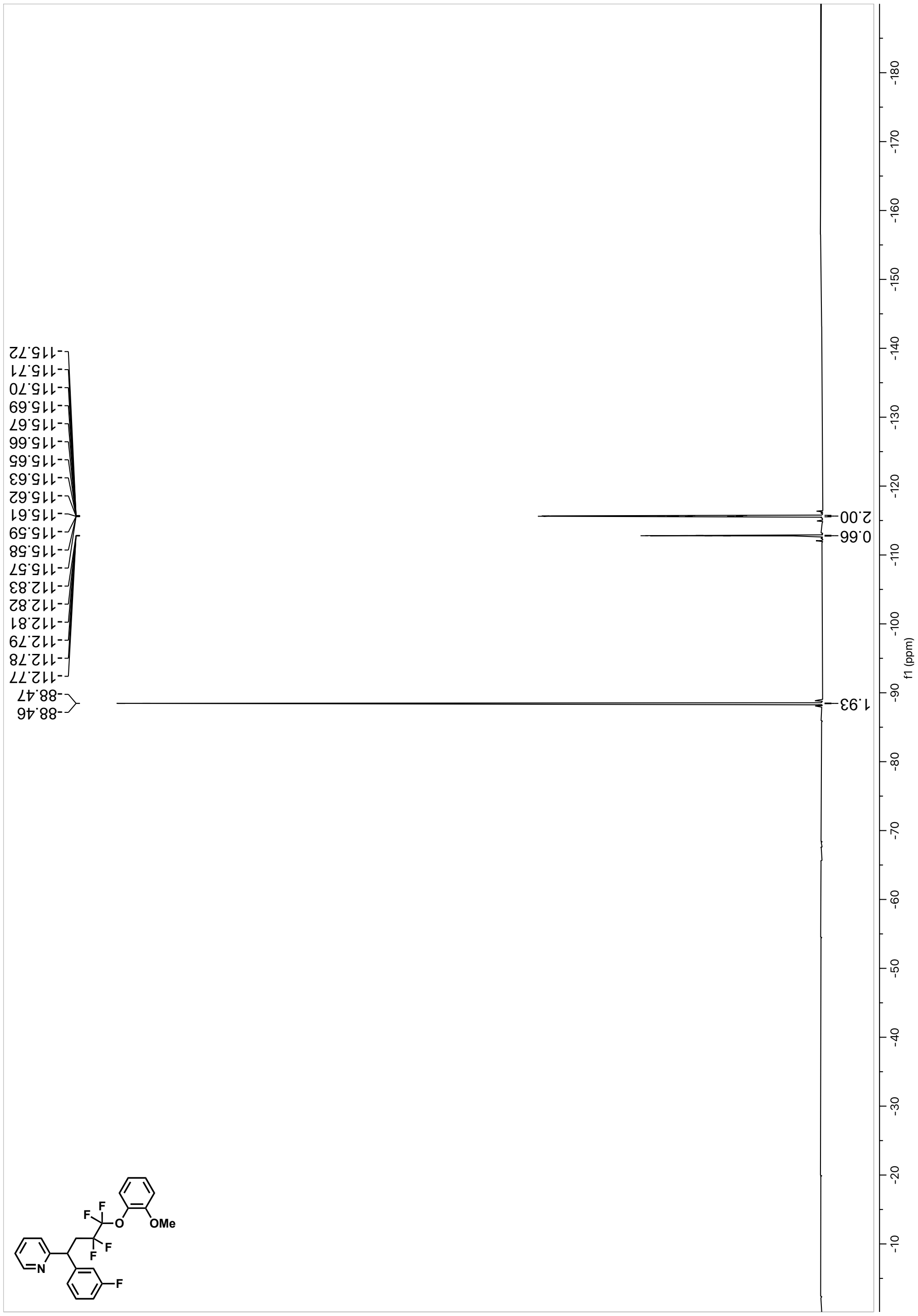

Compound 4o. ^{1}H NMR ($CDCl_3$, 400 MHz).

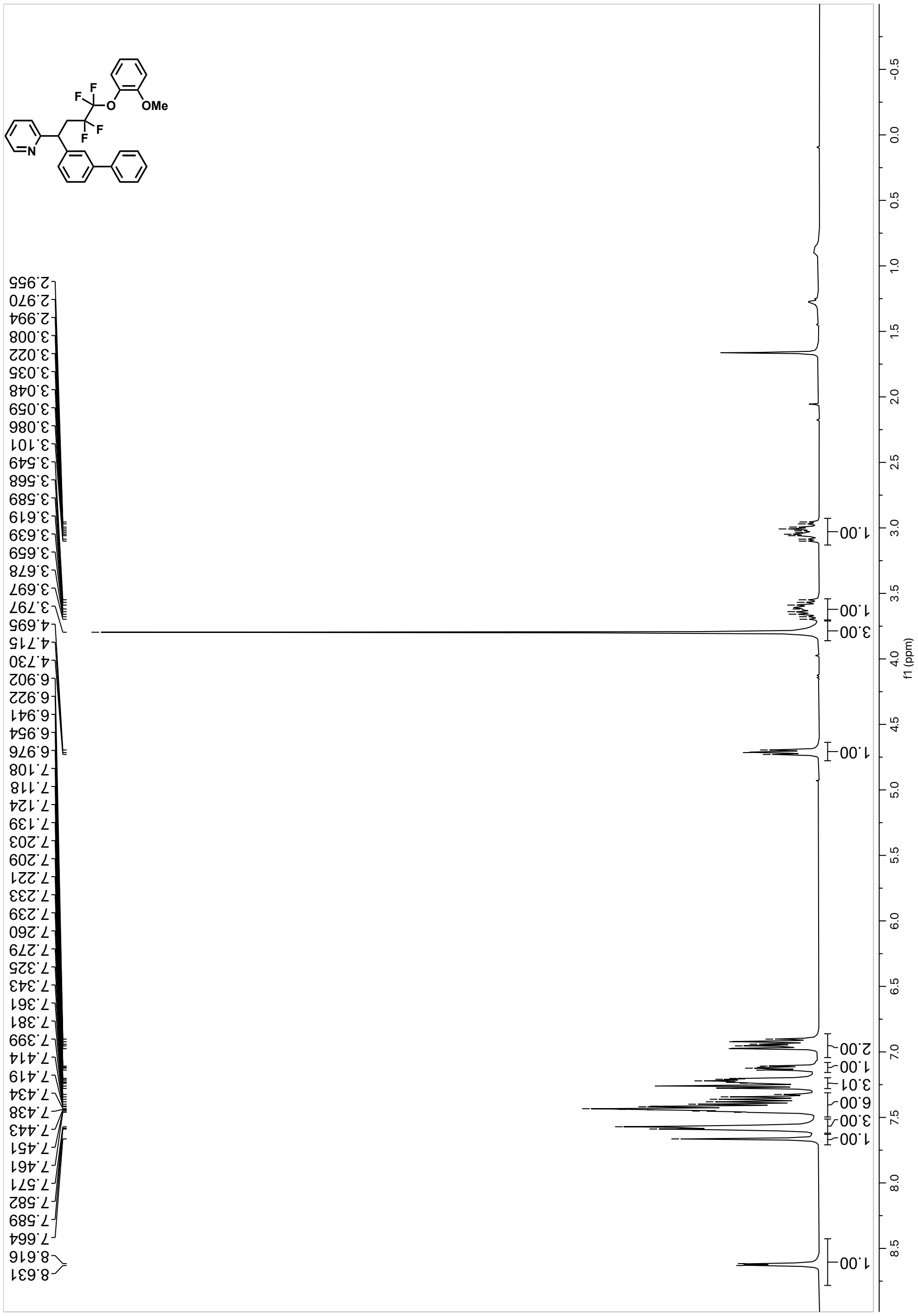

Compound 4o. ^{13}C NMR ($CDCl_3$, 100 MHz).

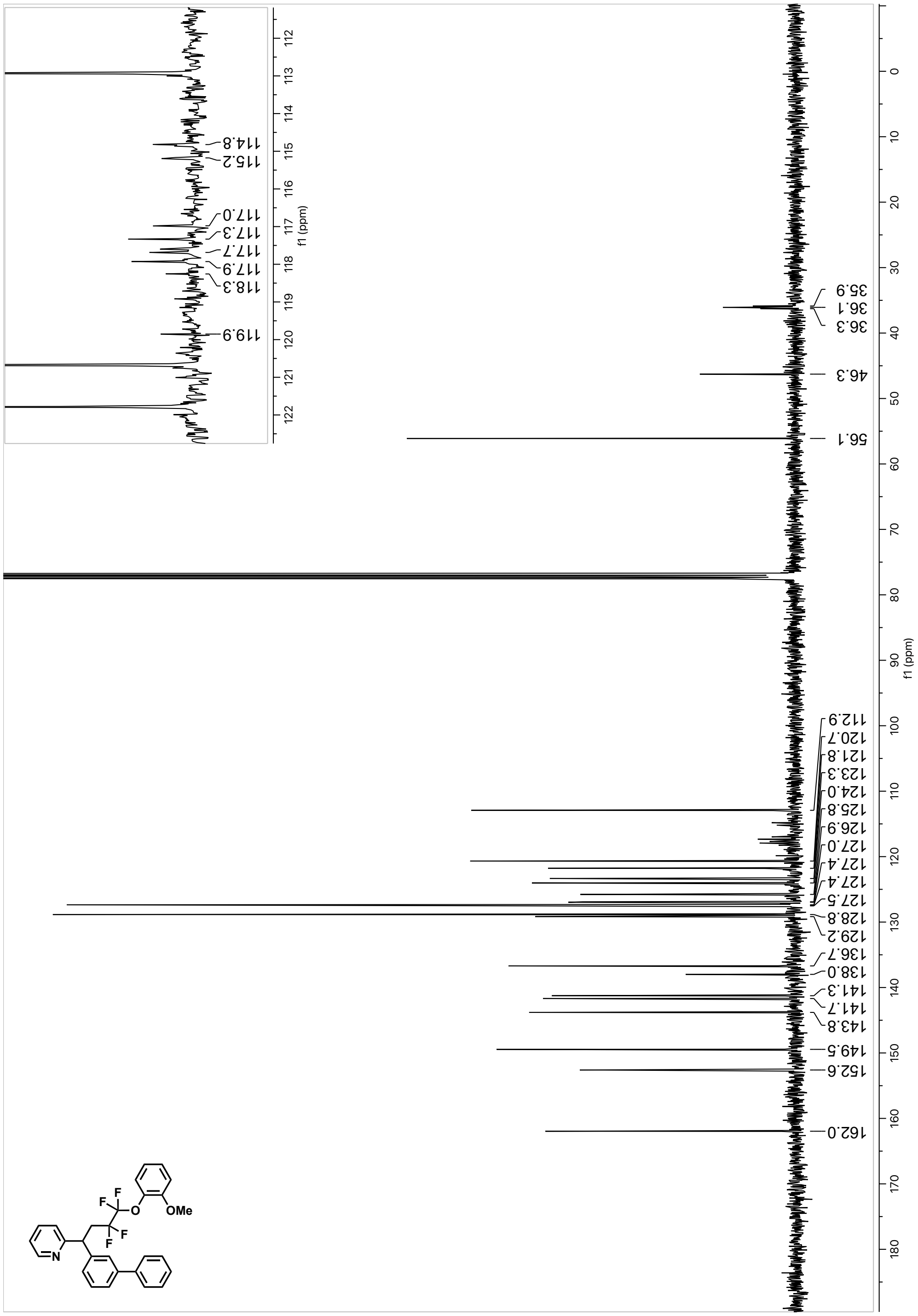

Compound 4o. ^{19}F NMR ($CDCl_3$, 376 MHz).

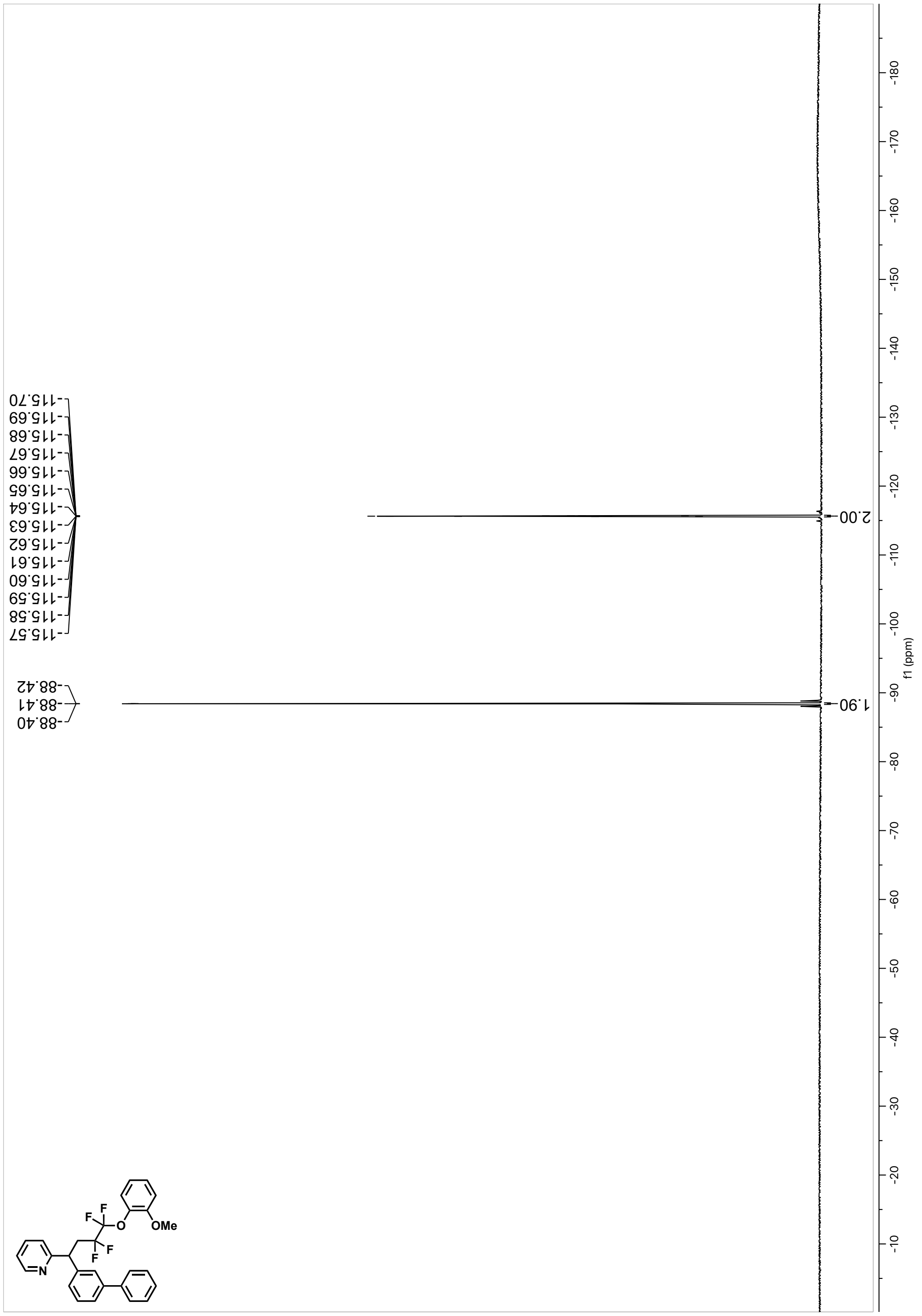

Compound 4p. ^{1}H NMR ($CDCl_3$, 400 MHz).

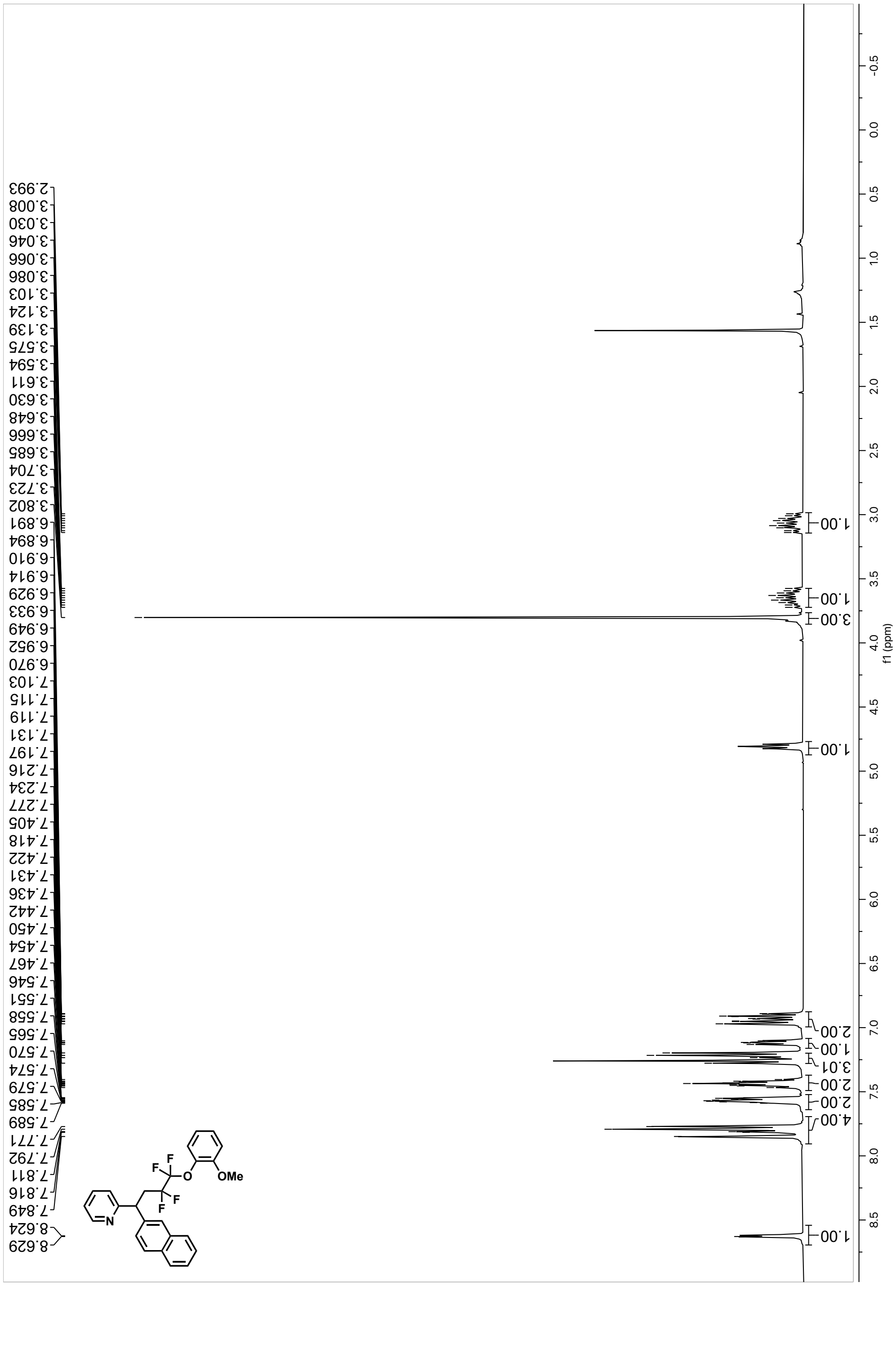

Compound 4p. ^{13}C NMR ($CDCl_3$, 100 MHz).

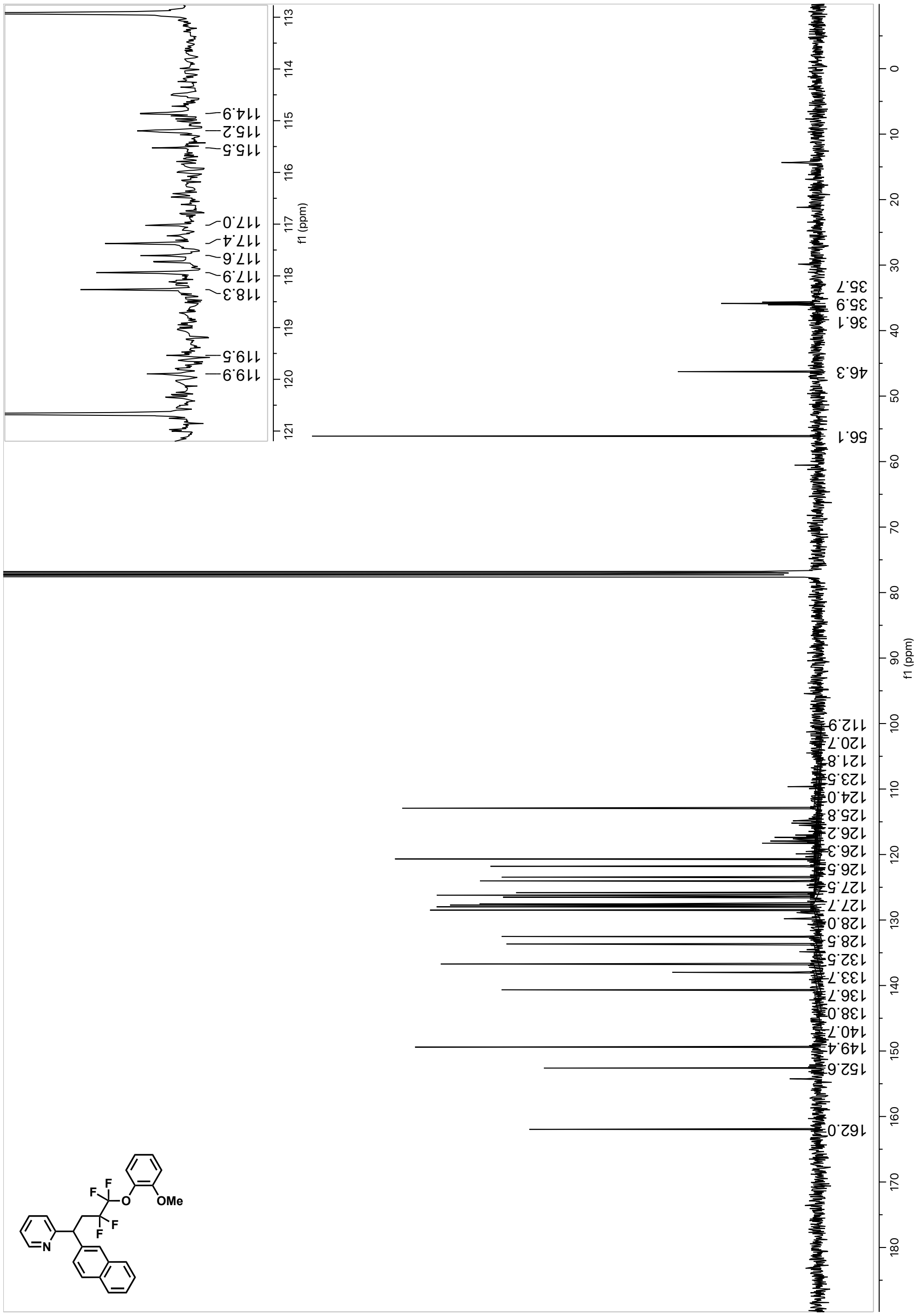

Compound 4p. ^{19}F NMR ($CDCl_3$, 376 MHz).

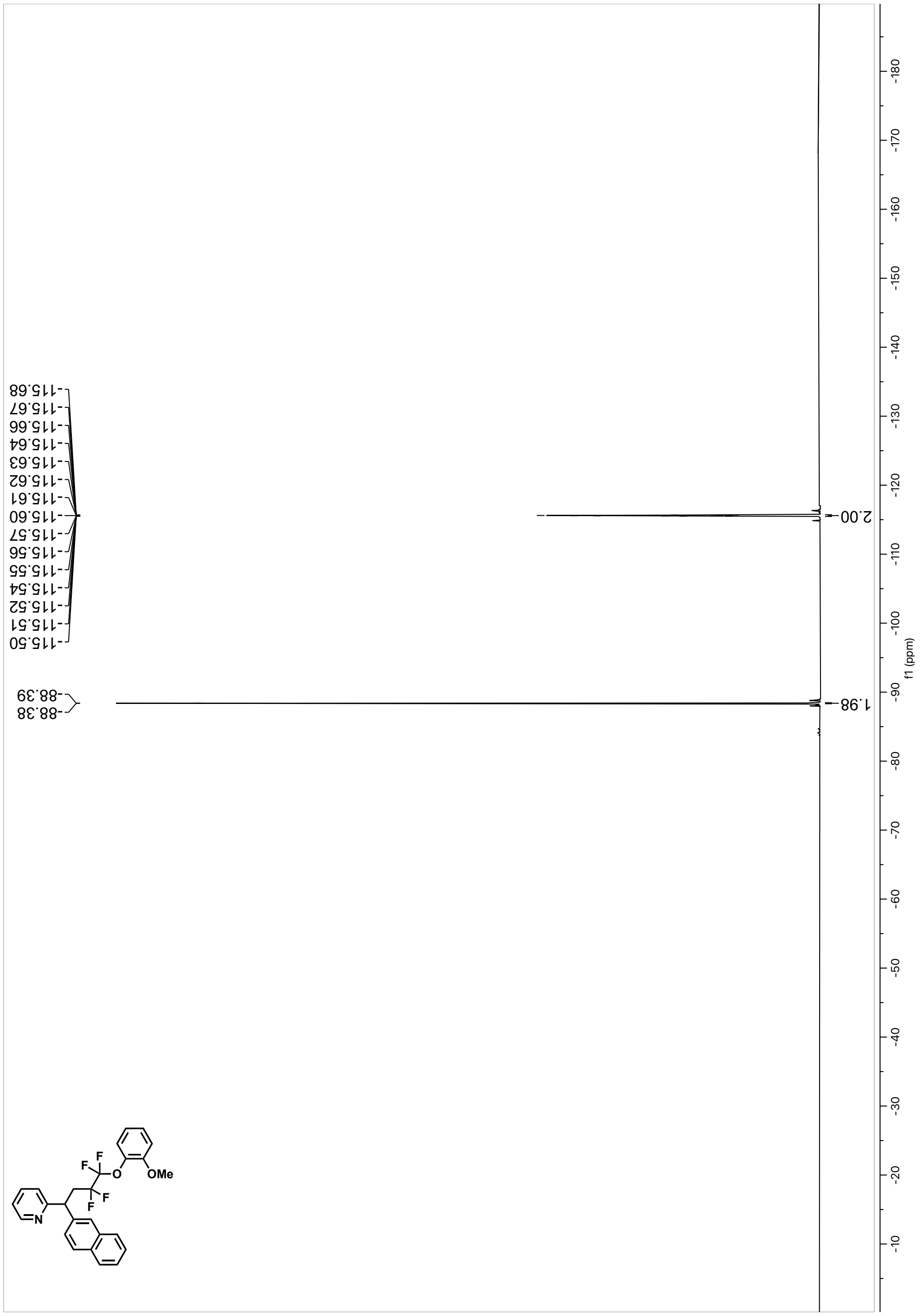

Compound 4q. ^{1}H NMR ($CDCl_3$, 400 MHz).

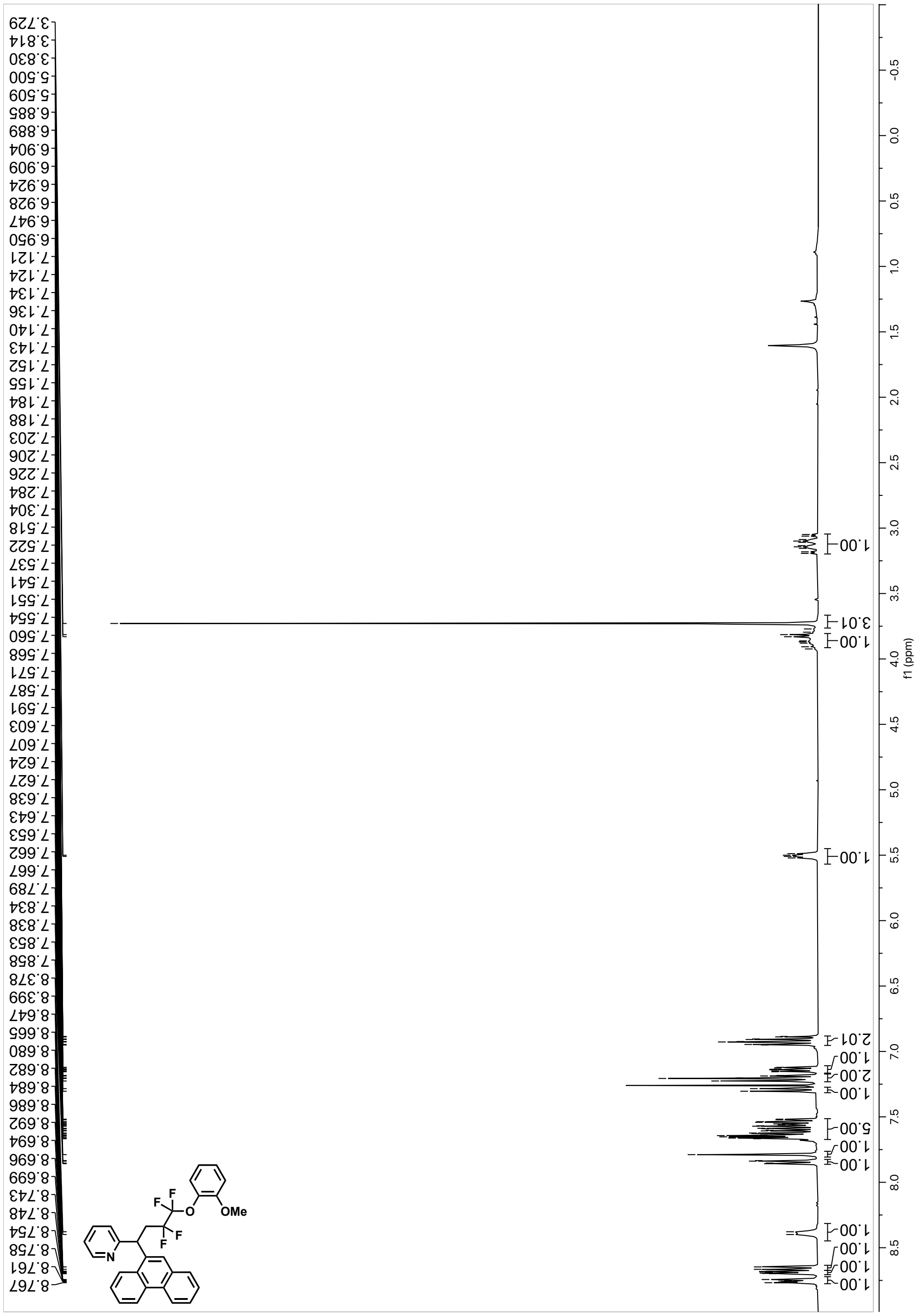

Compound 4q. ^{13}C NMR ($CDCl_3$, 100 MHz).

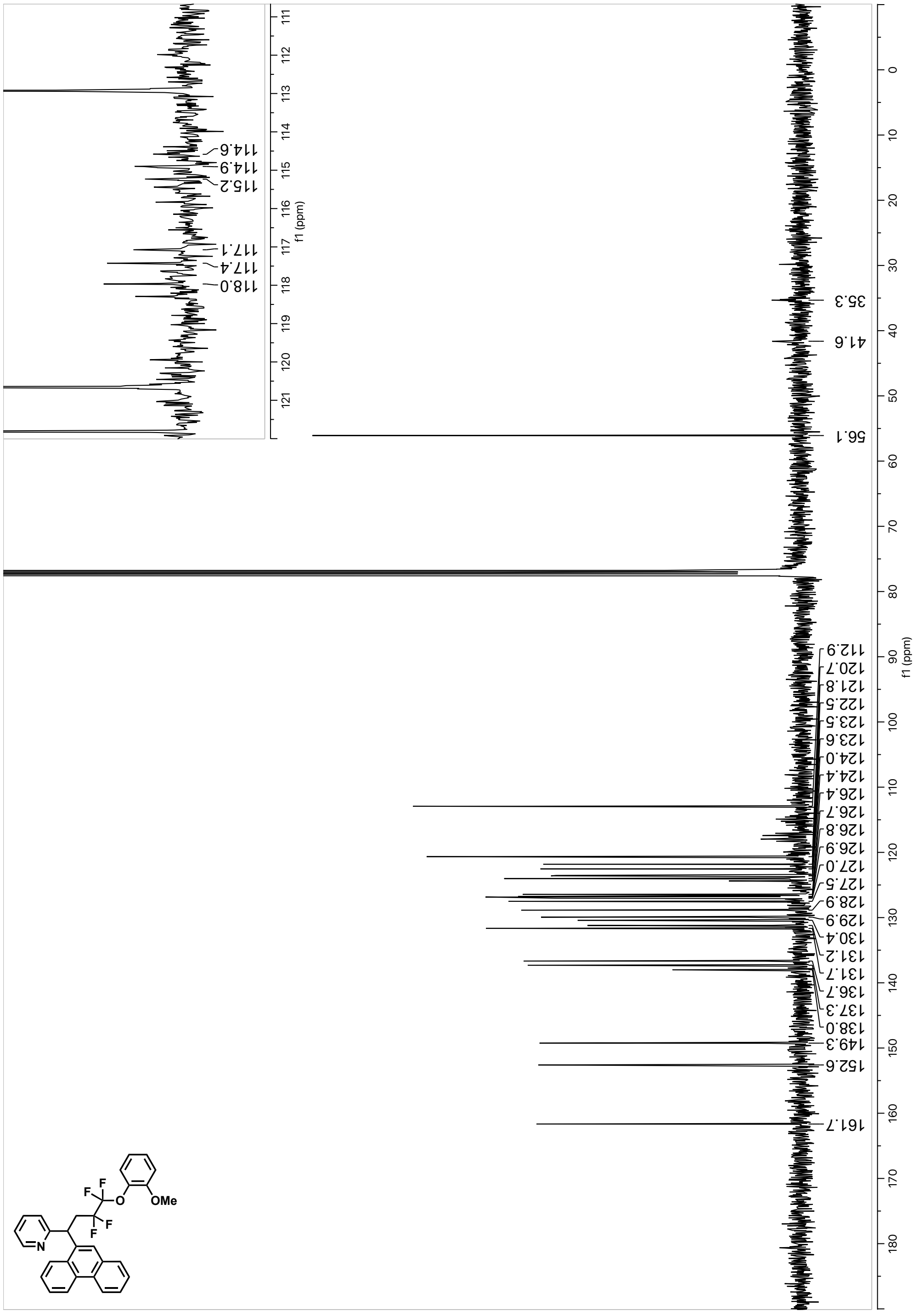

Compound 4q. ^{19}F NMR ($CDCl_3$, 376 MHz).

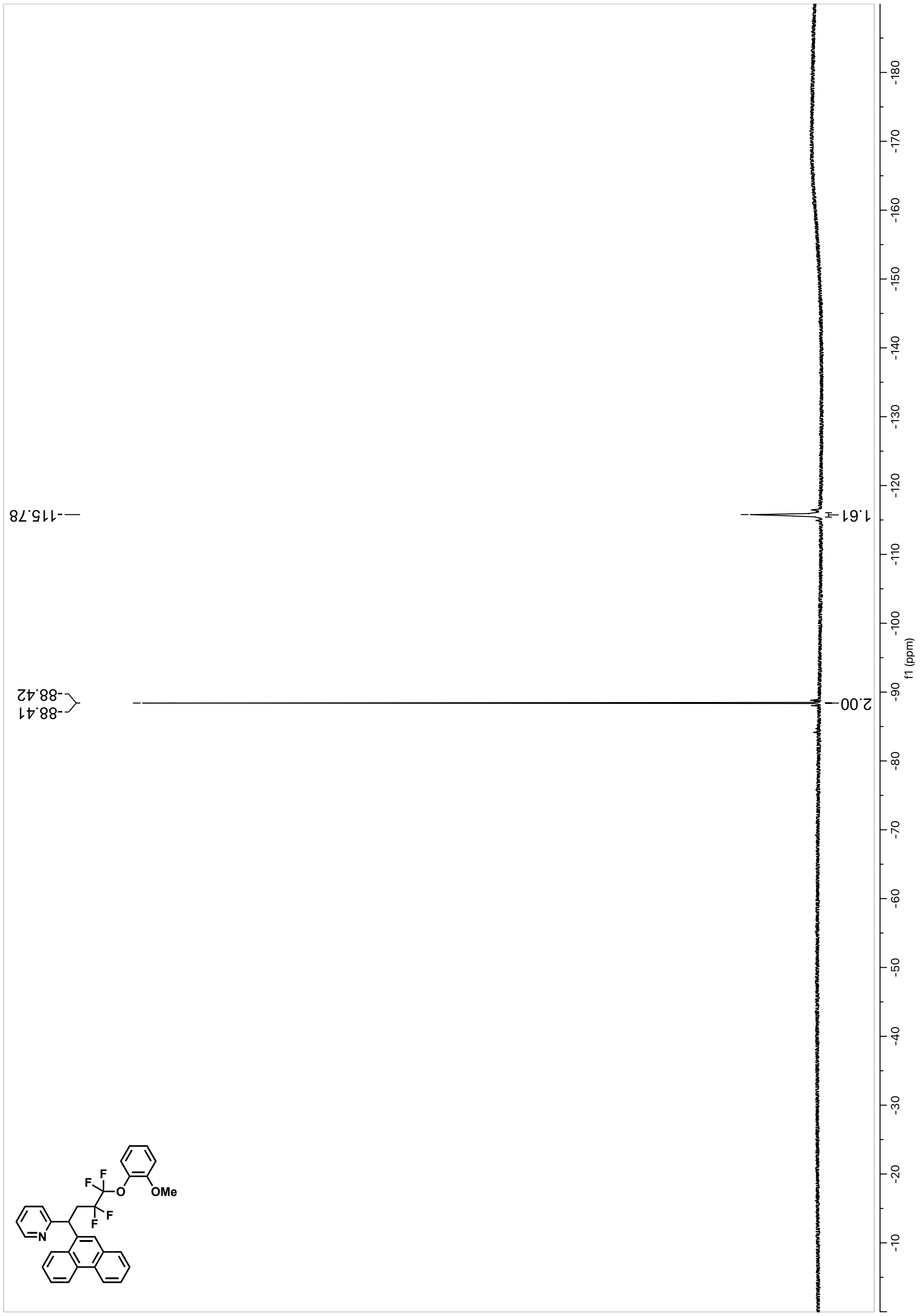

Compound 4r. ^{1}H NMR ($CDCl_3$, 400 MHz).

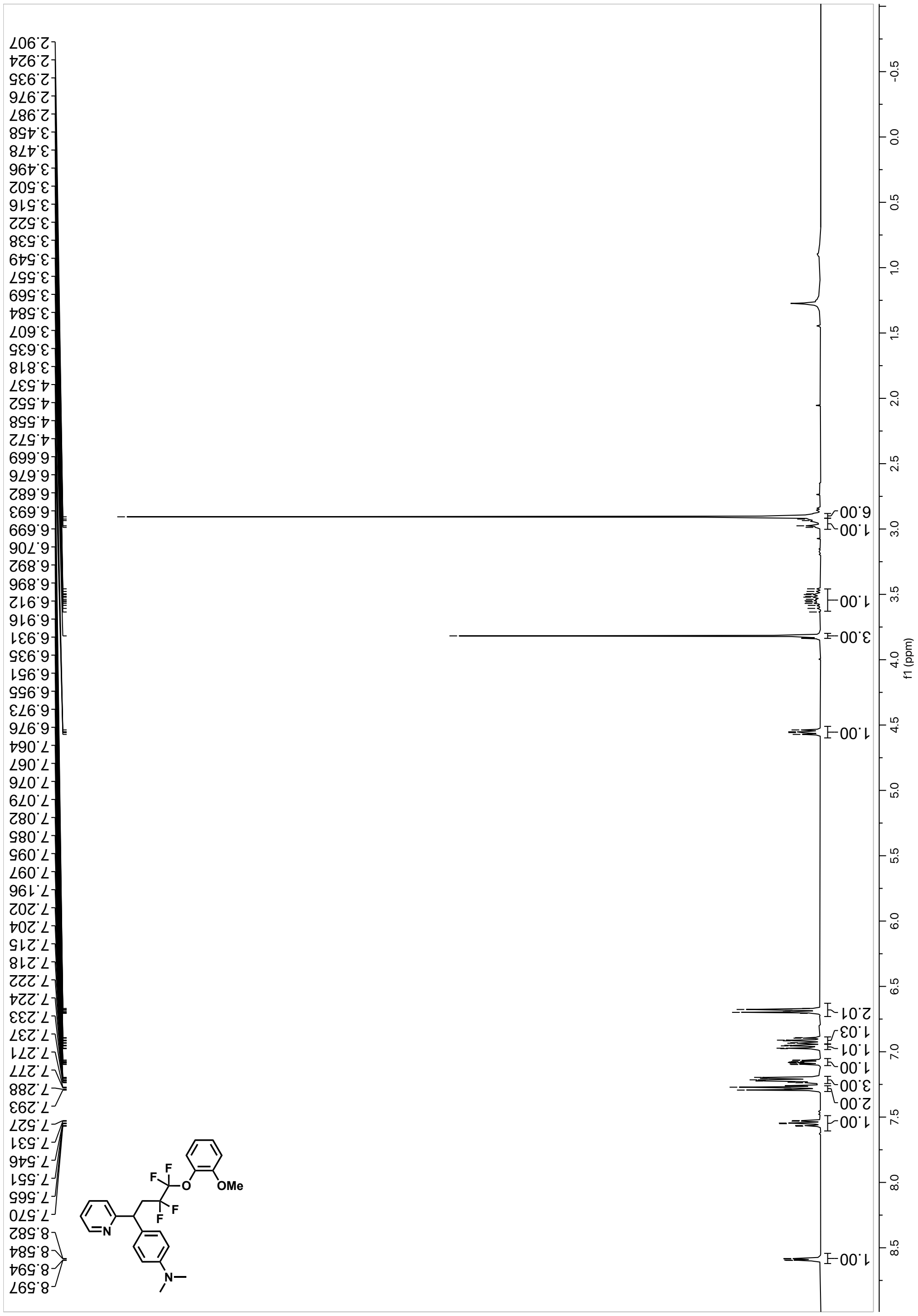

Compound 4r. ^{13}C NMR

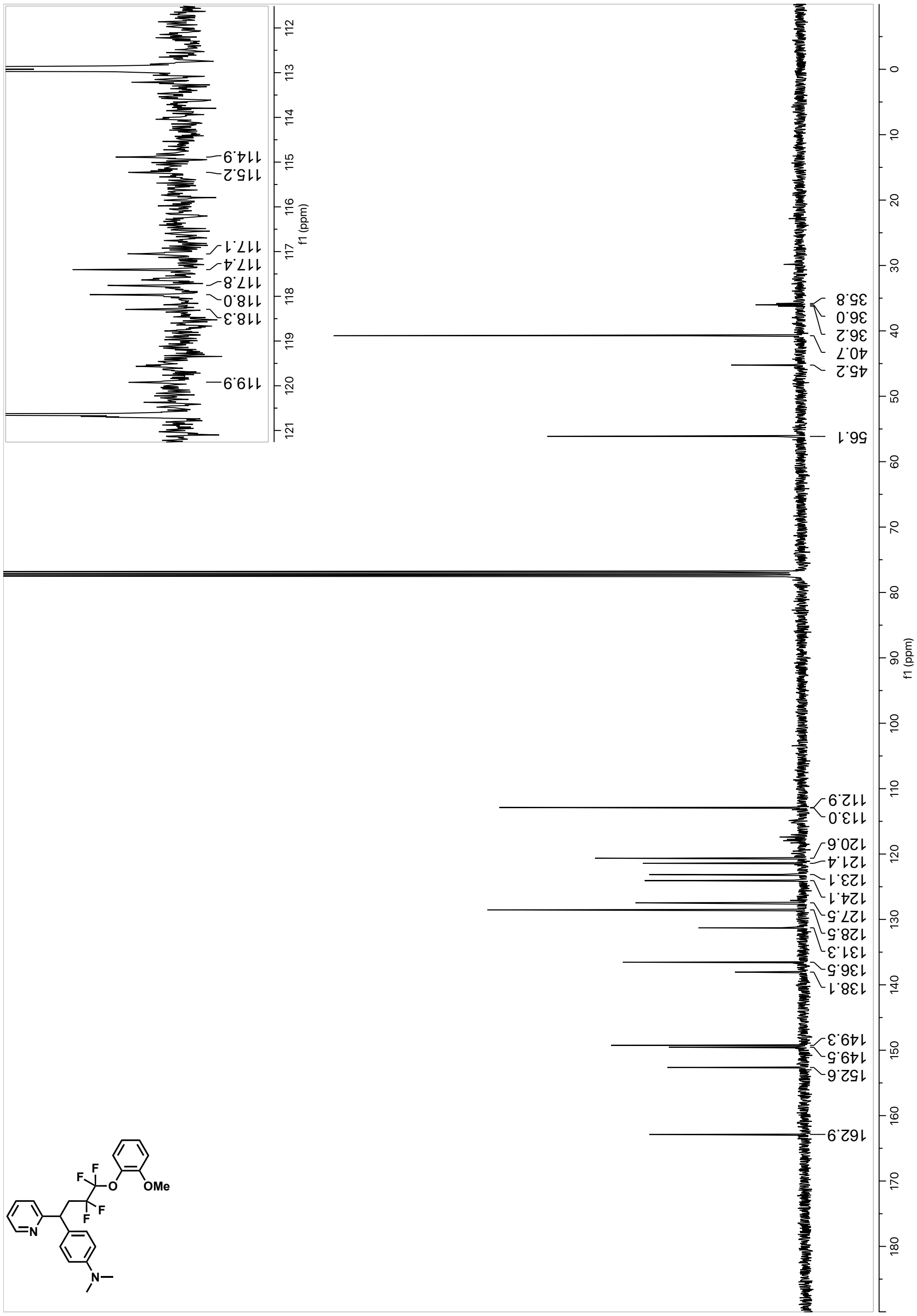

Compound 4r. ^{19}F NMR ($CDCl_3$, 376 MHz).

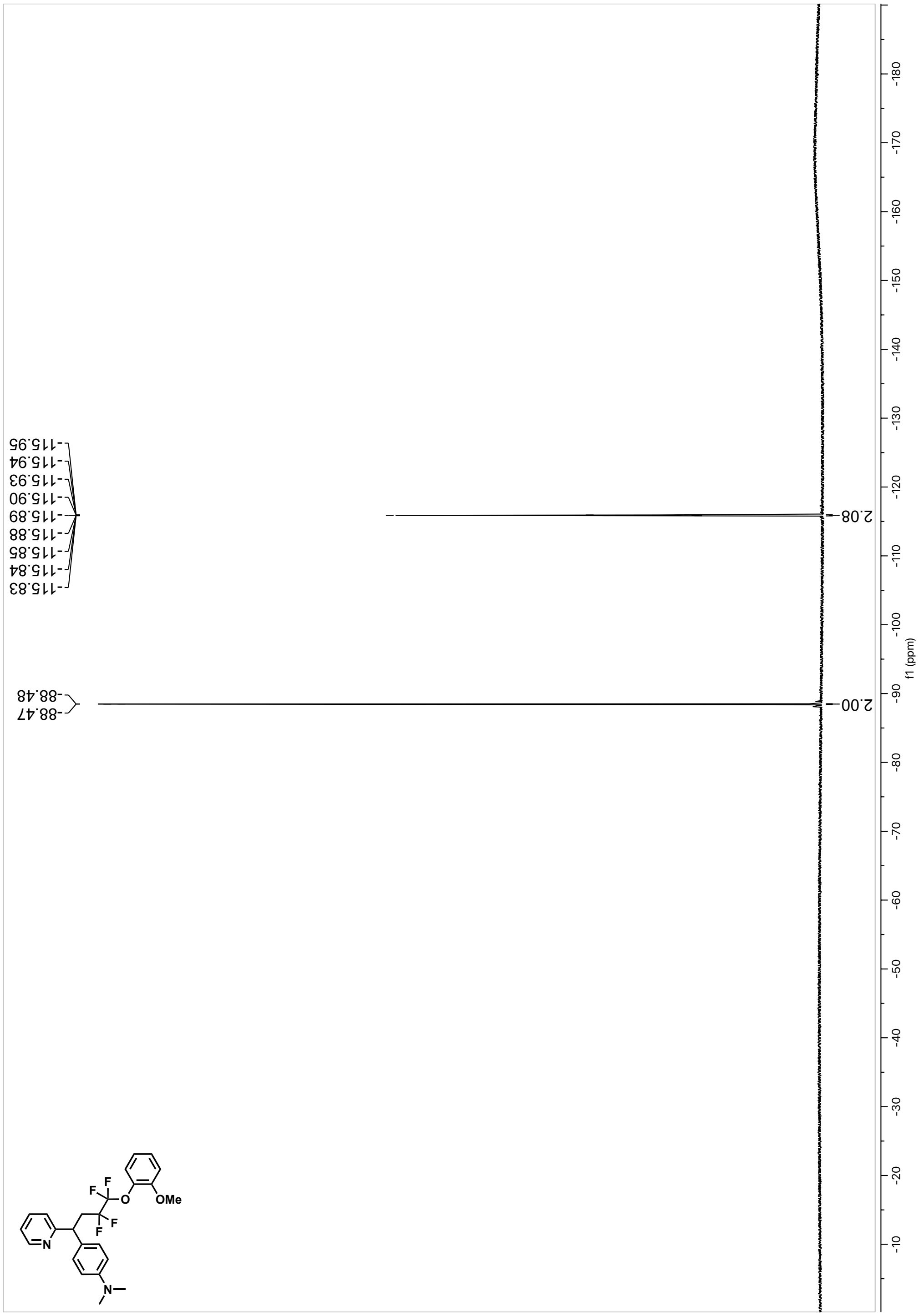

Compound 4s. ^{1}H NMR ($CDCl_3$, 400 MHz).

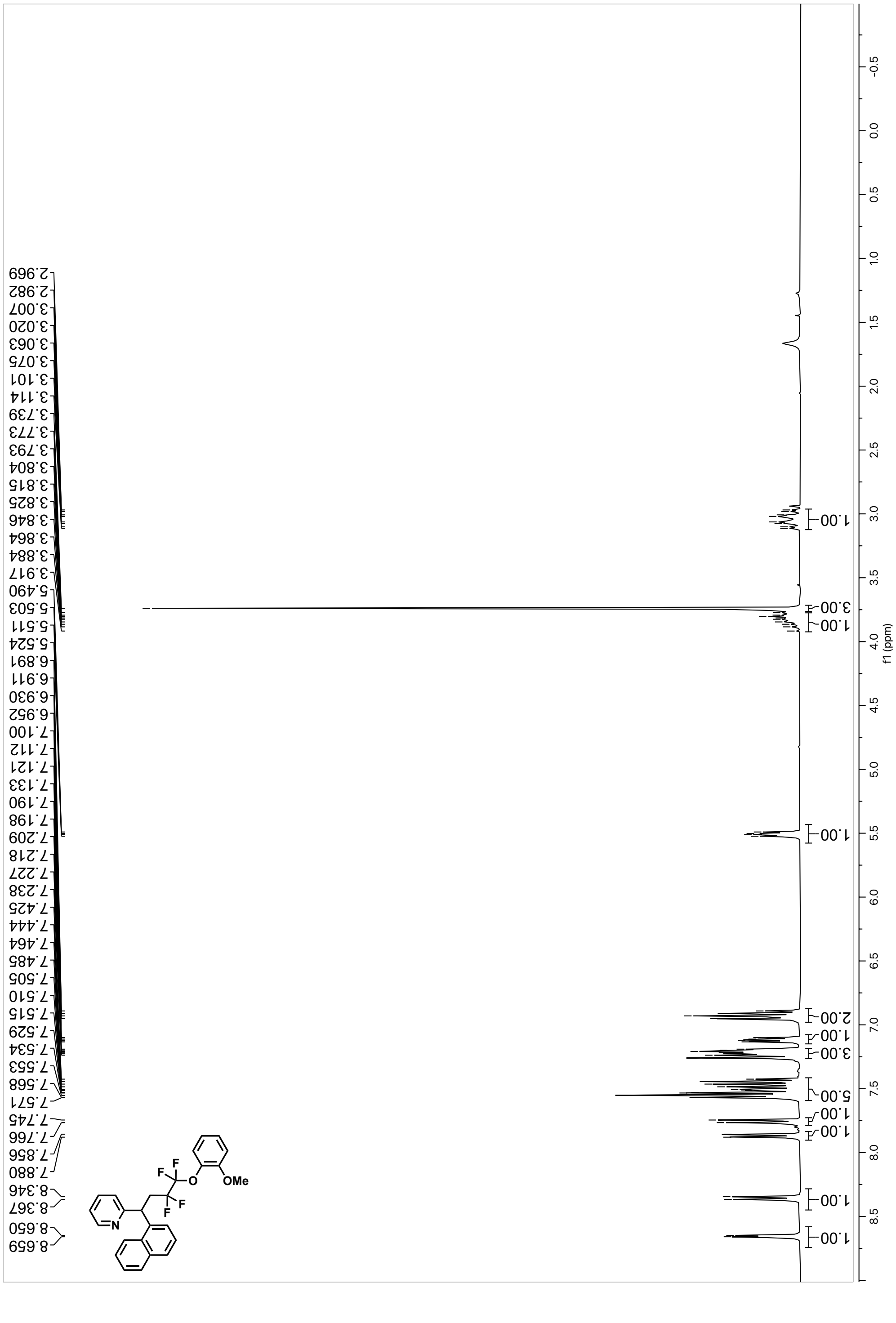

Compound 4s. ^{13}C NMR ($CDCl_3$, 100 MHz).

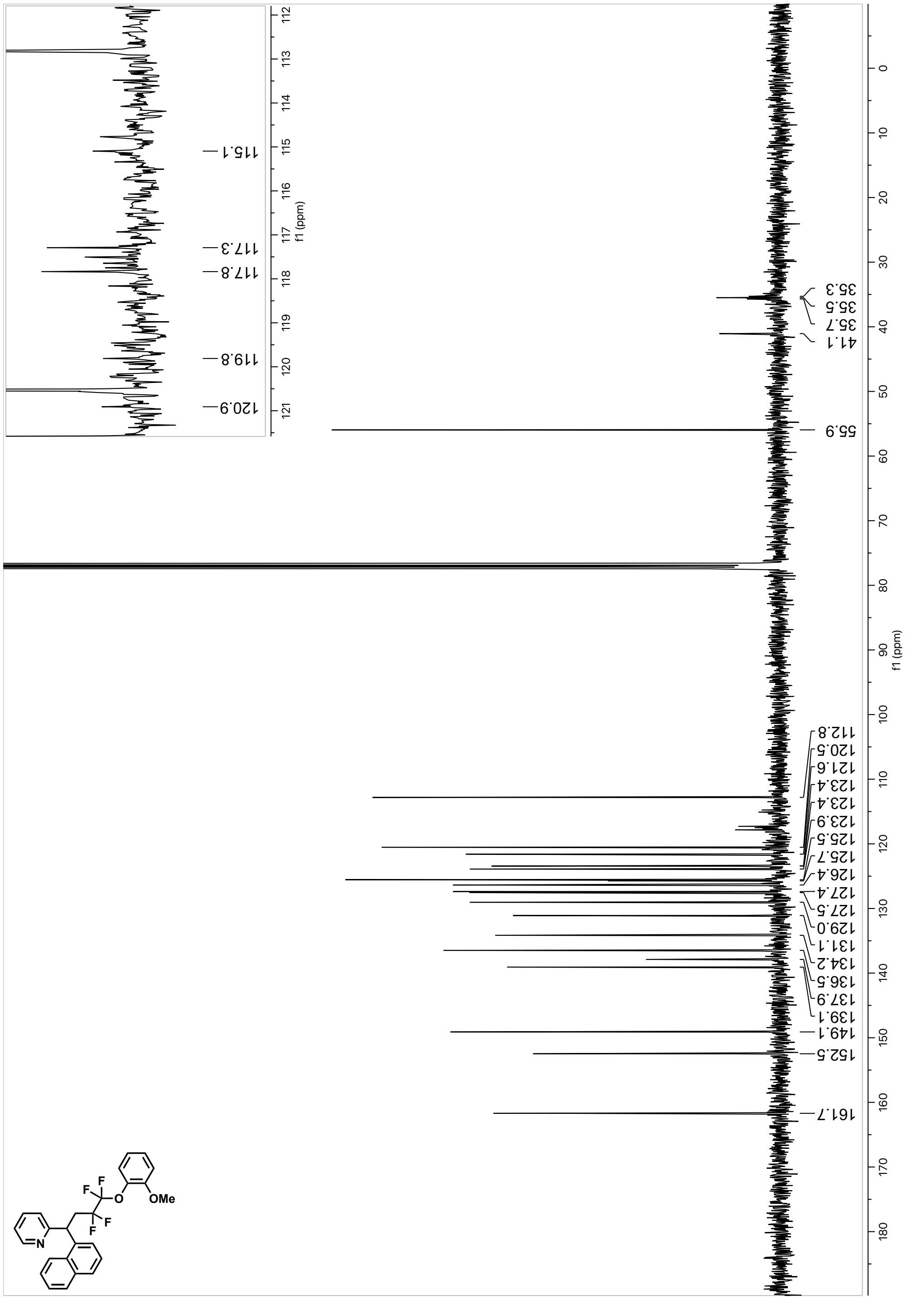

Compound 4s. ^{19}F NMR (CDCl$_3$, 376 MHz).

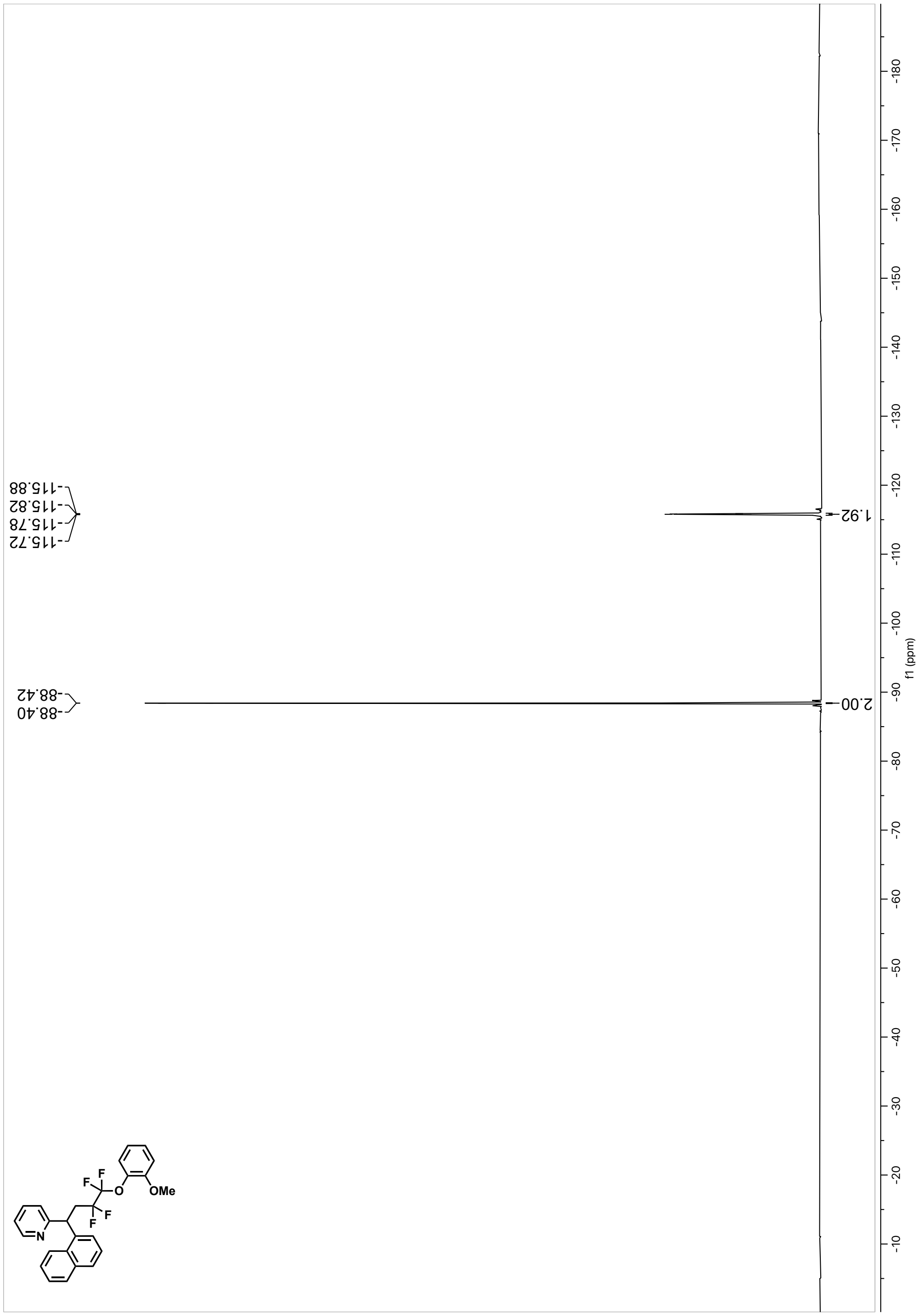

Compound 4t. ^{1}H NMR ($CDCl_3$, 400 MHz).

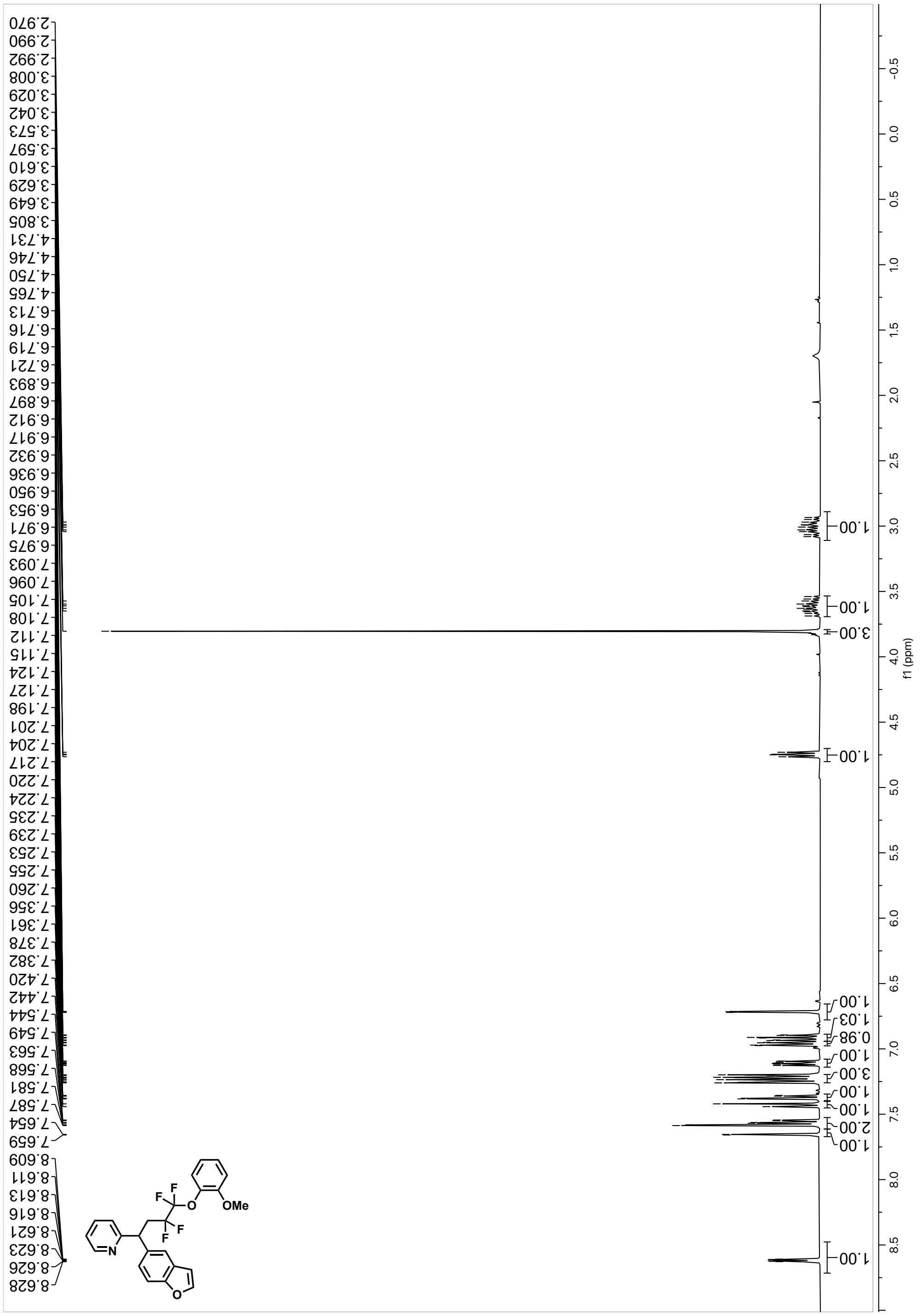

Compound 4t. ^{13}C NMR ($CDCl_3$, 100 MHz).

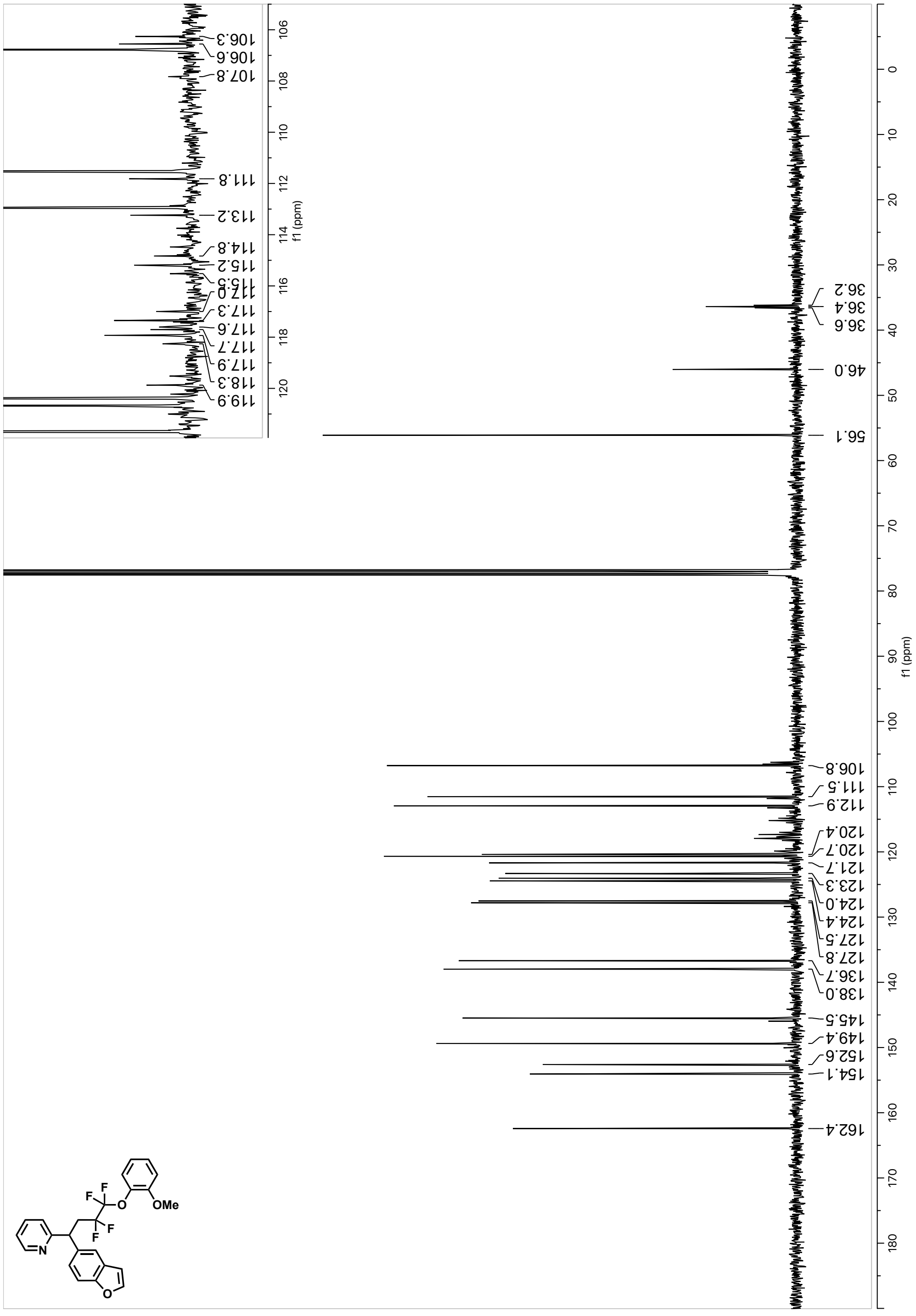

Compound 4t. ^{19}F NMR ($CDCl_3$, 376 MHz).

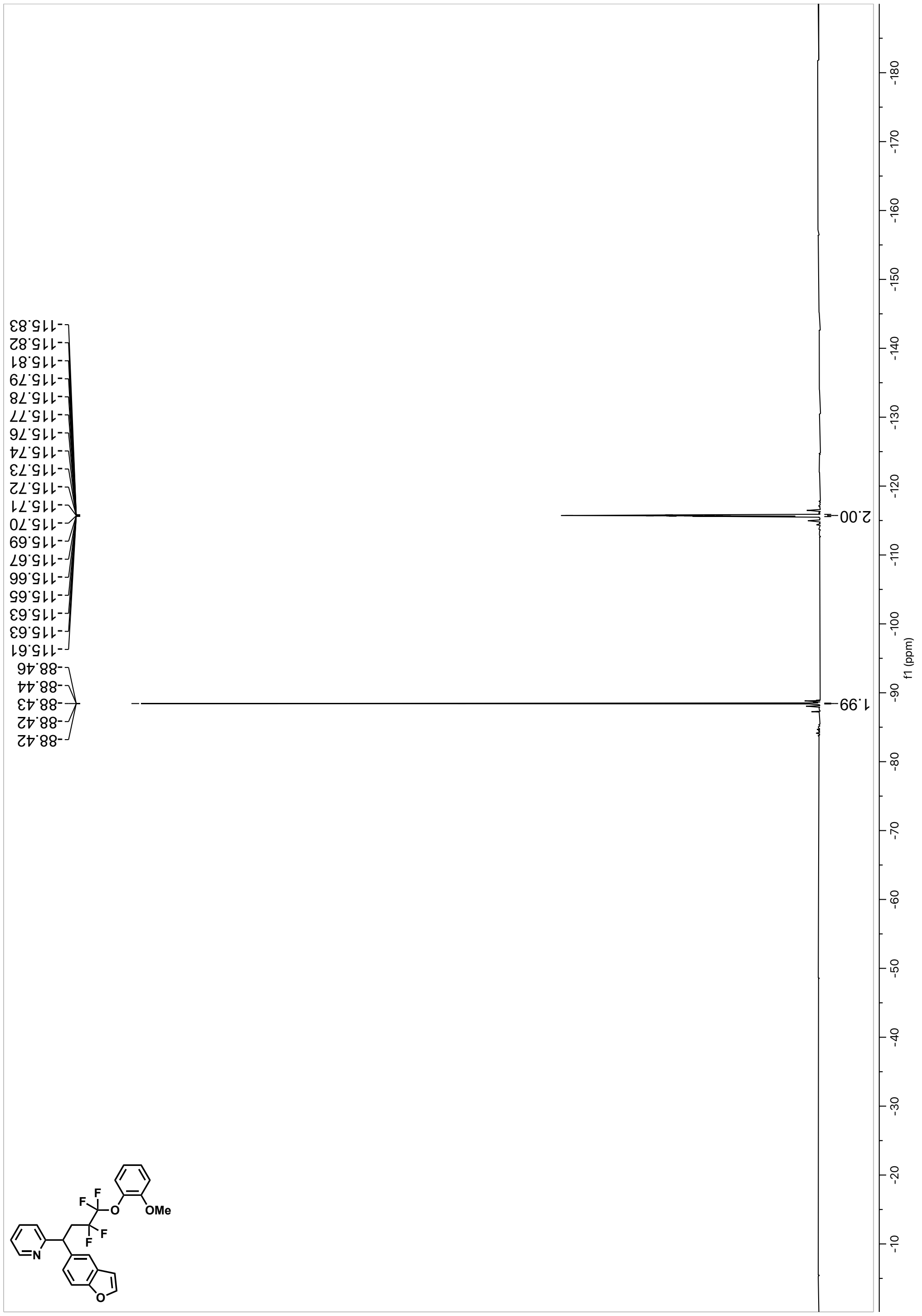

Compound 4u. ^{1}H NMR ($CDCl_3$, 400 MHz).

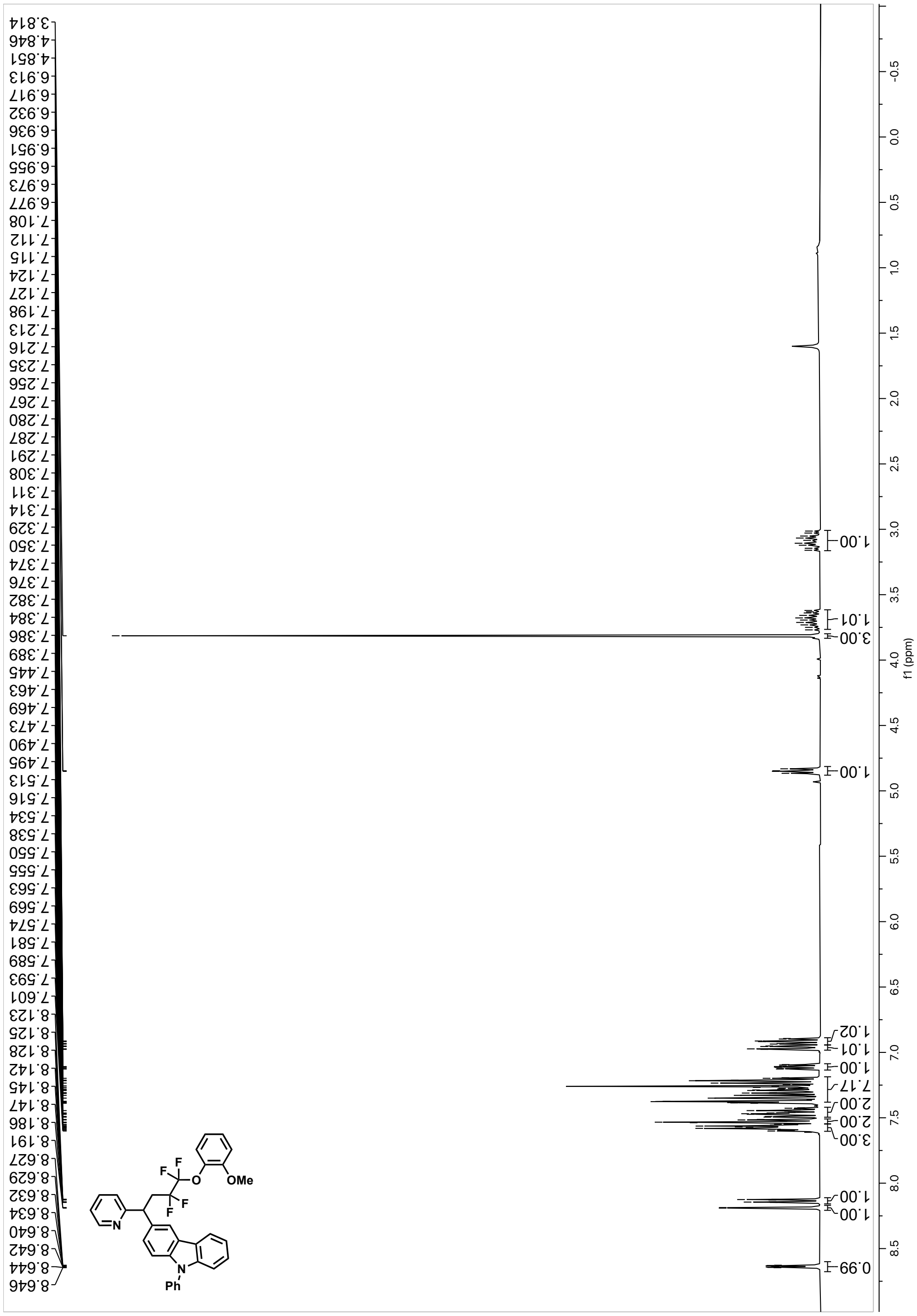

Compound 4u. ^{13}C NMR ($CDCl_3$, 100 MHz).

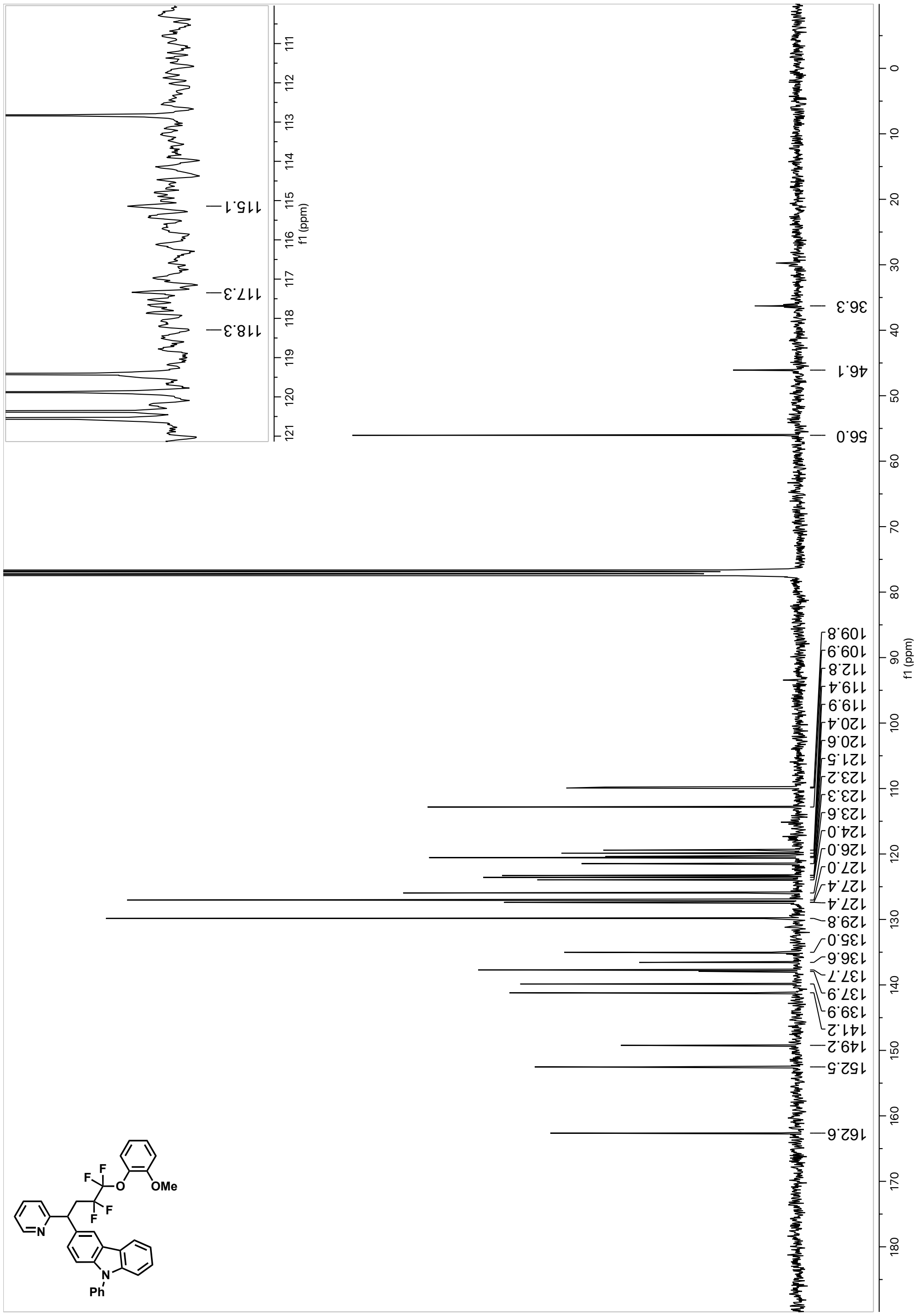

Compound 4u. ^{19}F NMR ($CDCl_3$, 376 MHz).

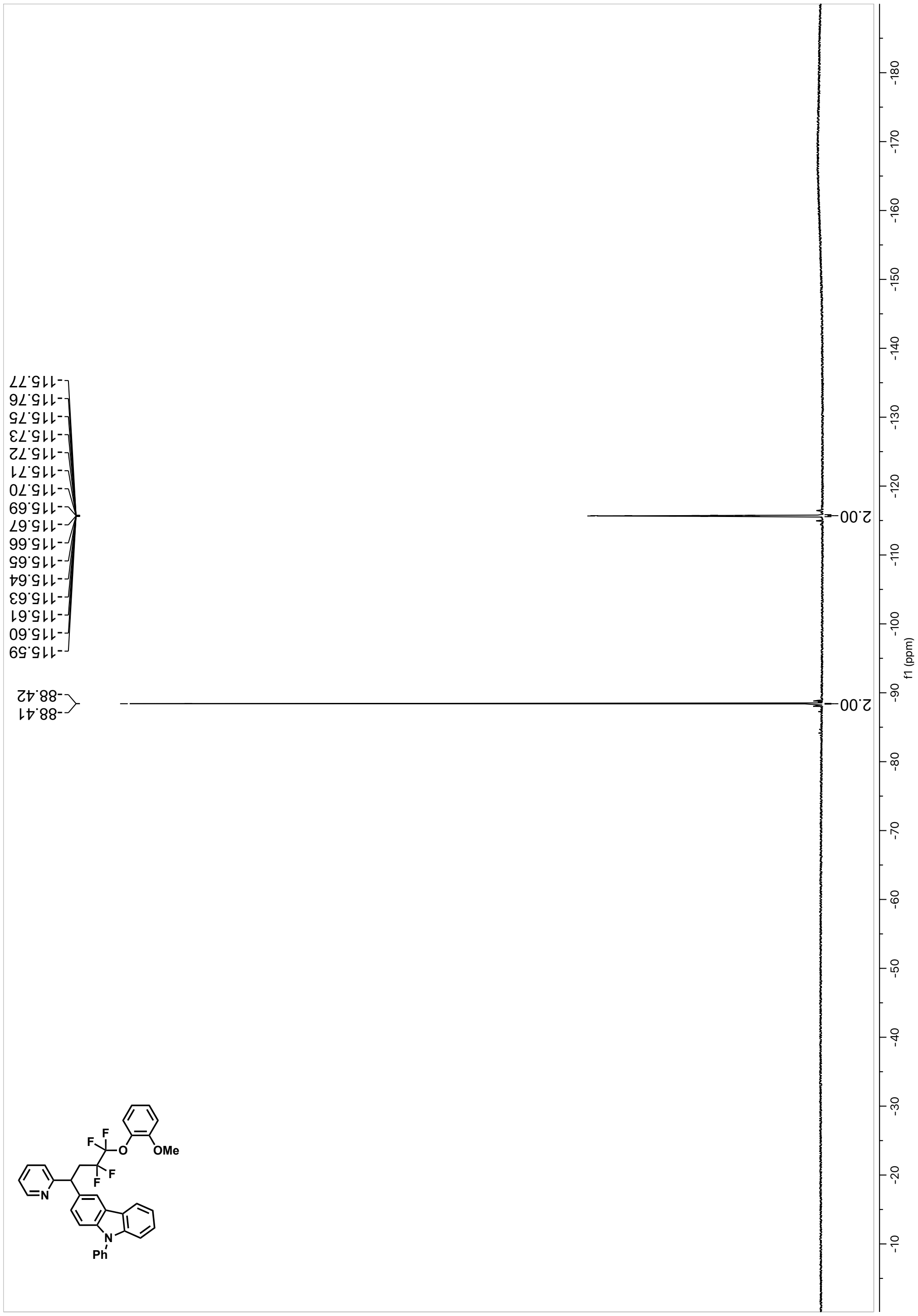

Compound 4v. ^{1}H NMR ($CDCl_3$, 400 MHz).

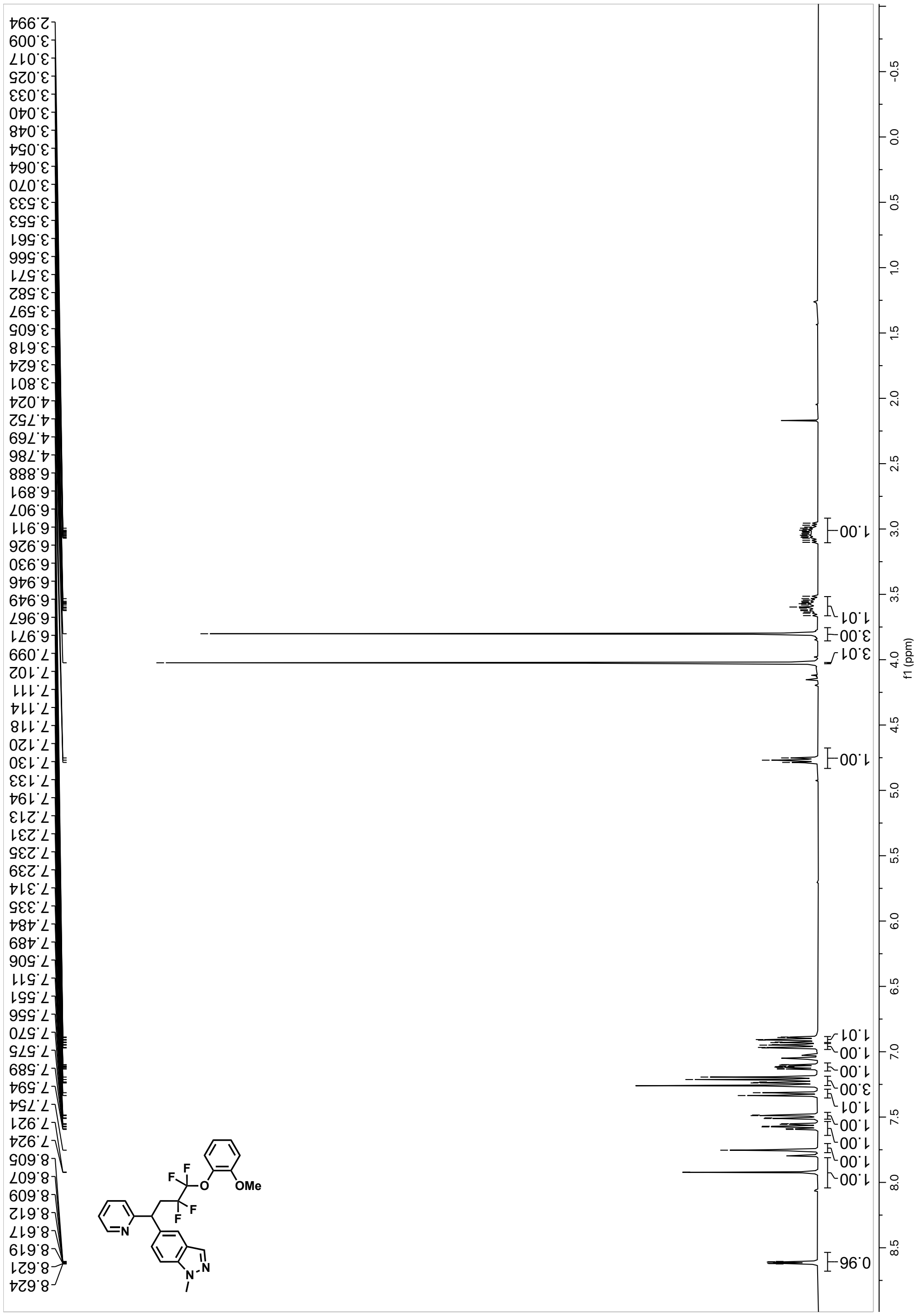

Compound 4v. ^{13}C NMR ($CDCl_3$, 100 MHz).

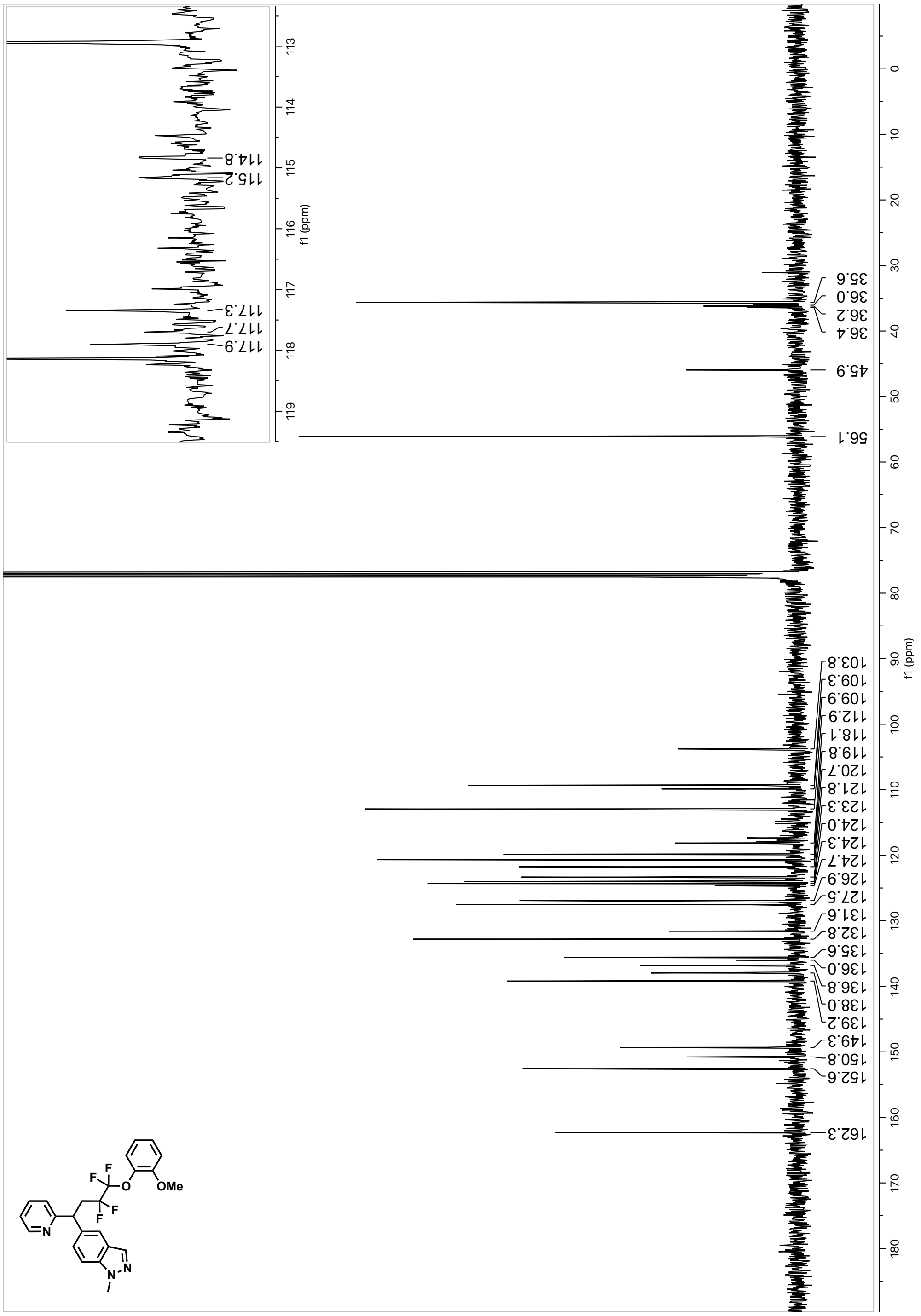

Compound 4v. ^{19}F NMR ($CDCl_3$, 376 MHz).

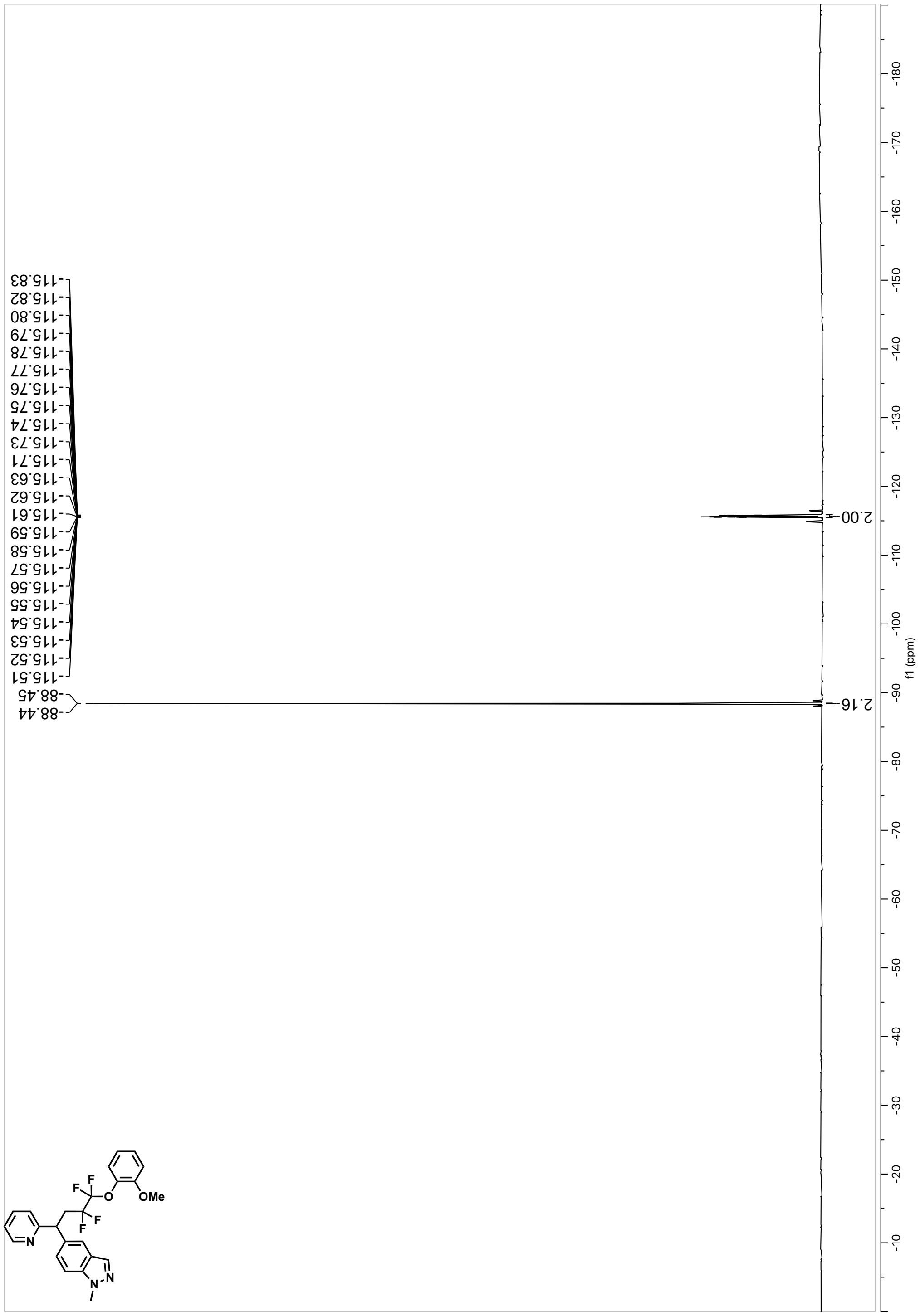

Compound 4w. Top: ^{1}H NMR ($CDCl_3$, 400 MHz). Bottom: ^{13}C NMR ($CDCl_3$, 100 MHz).

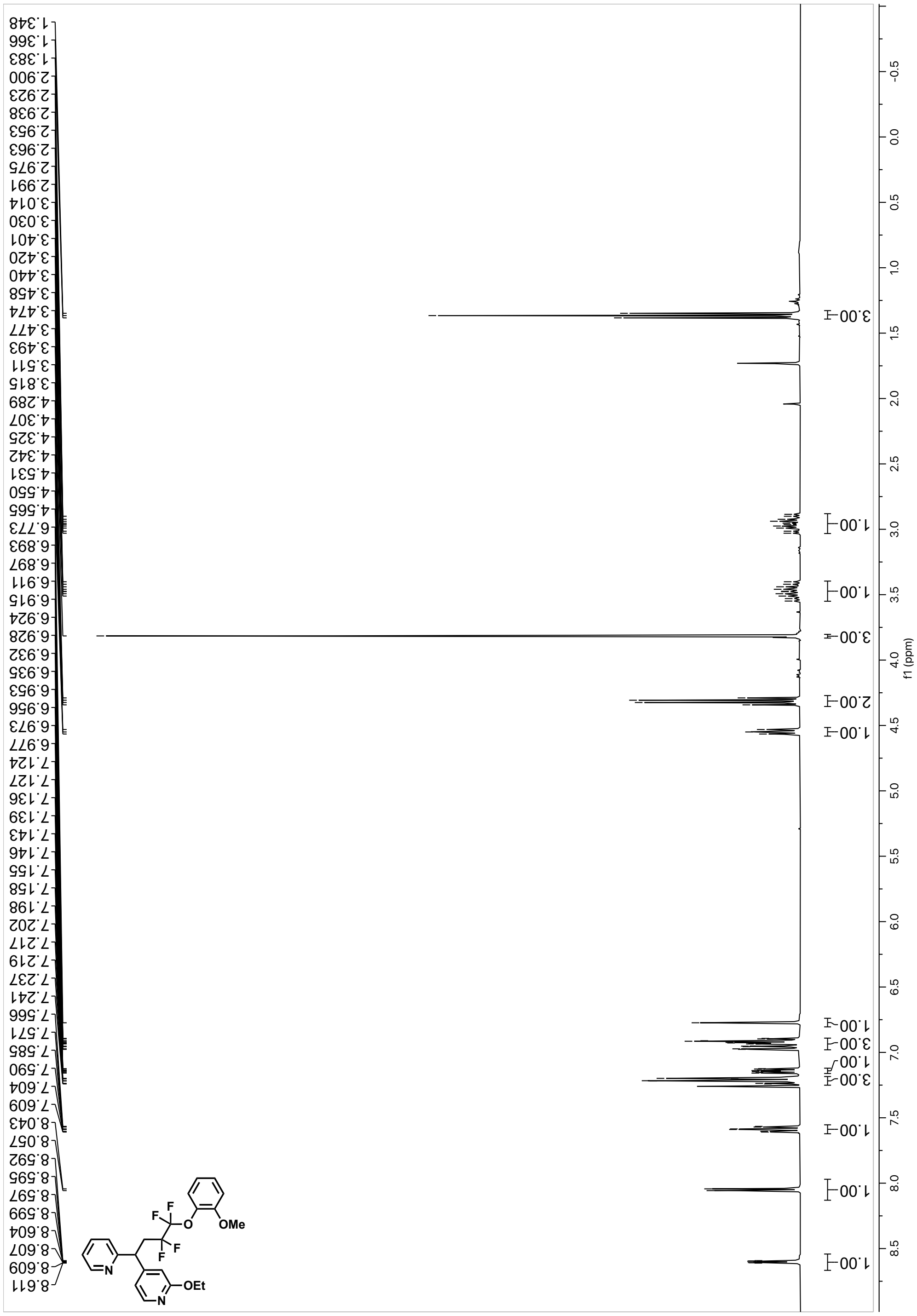

Compound 4w. ^{13}C NMR ($CDCl_3$, 100 MHz).

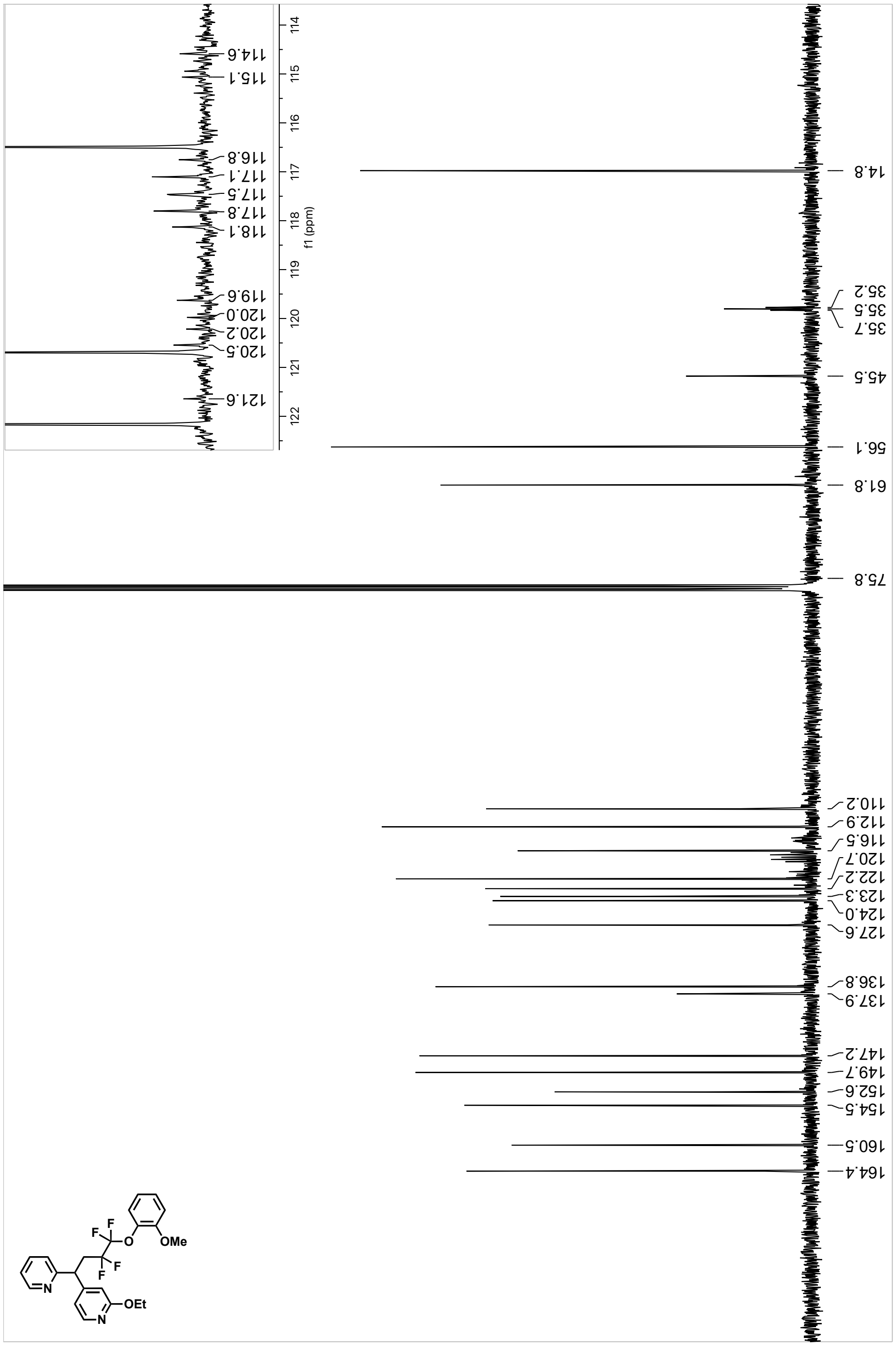

Compound 4w. ^{19}F NMR (CDCl$_3$, 376 MHz).

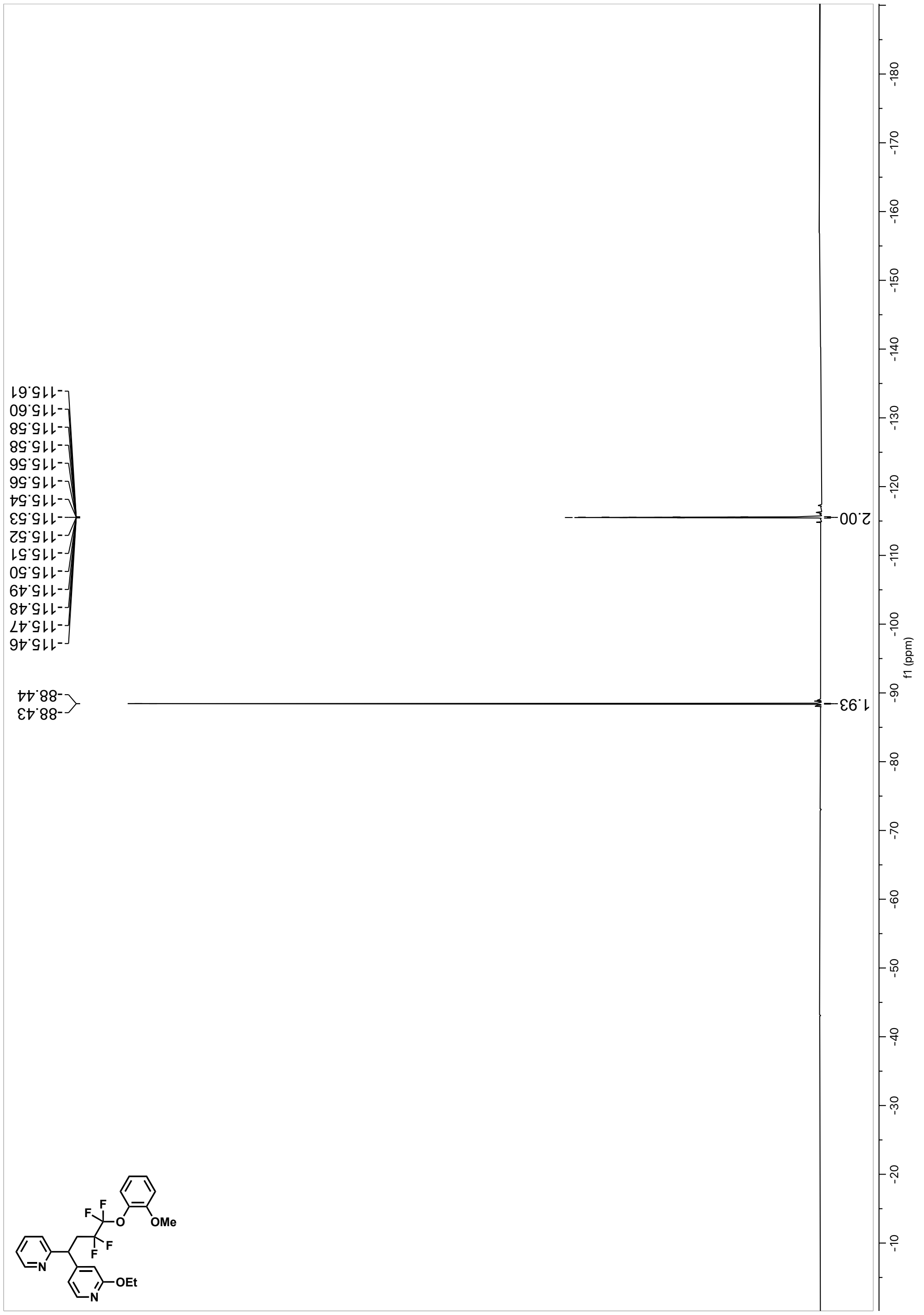

Compound 4x. ^{1}H NMR ($CDCl_3$, 400 MHz).

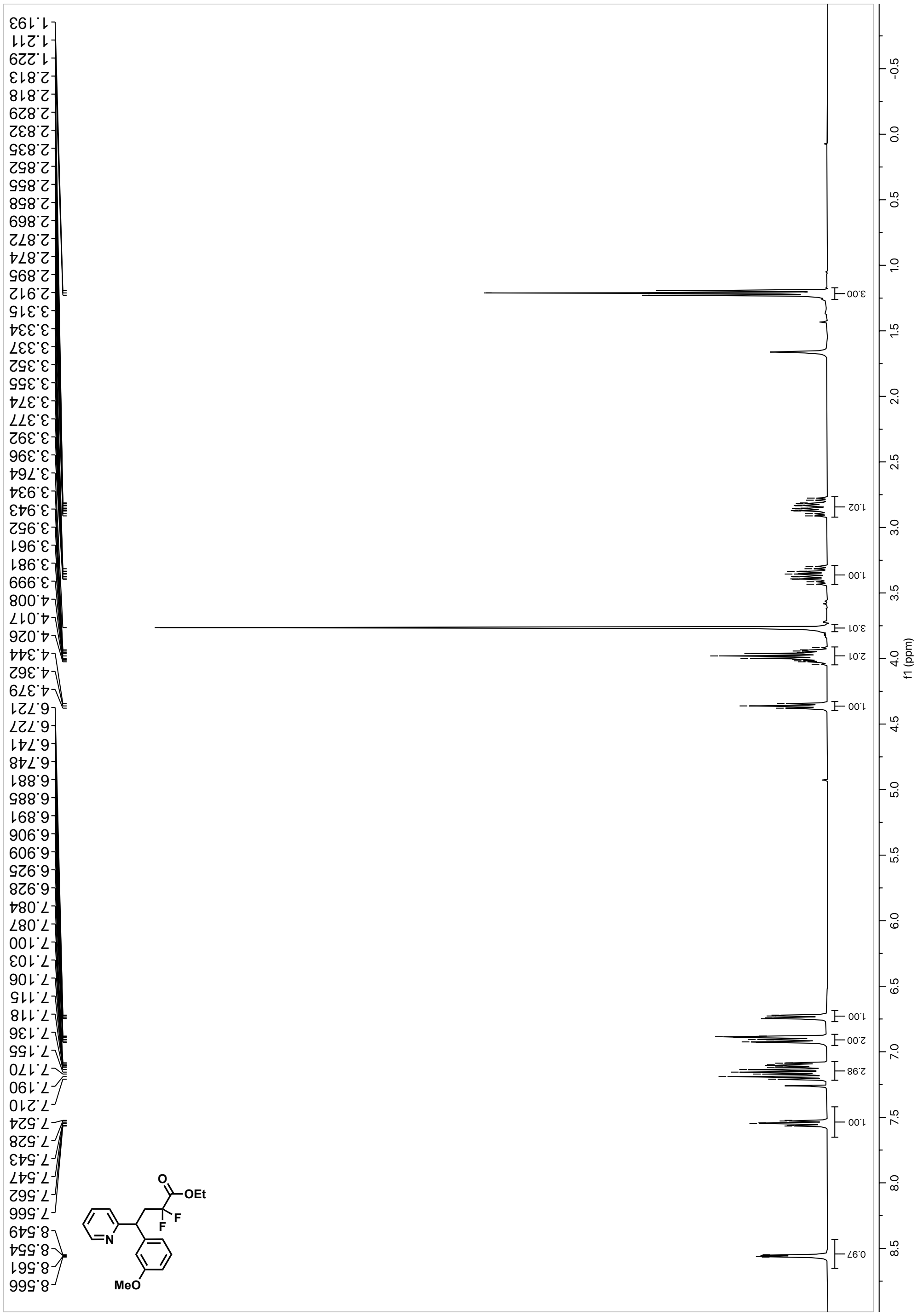

Compound 4x. ^{13}C NMR ($CDCl_3$, 100 MHz).

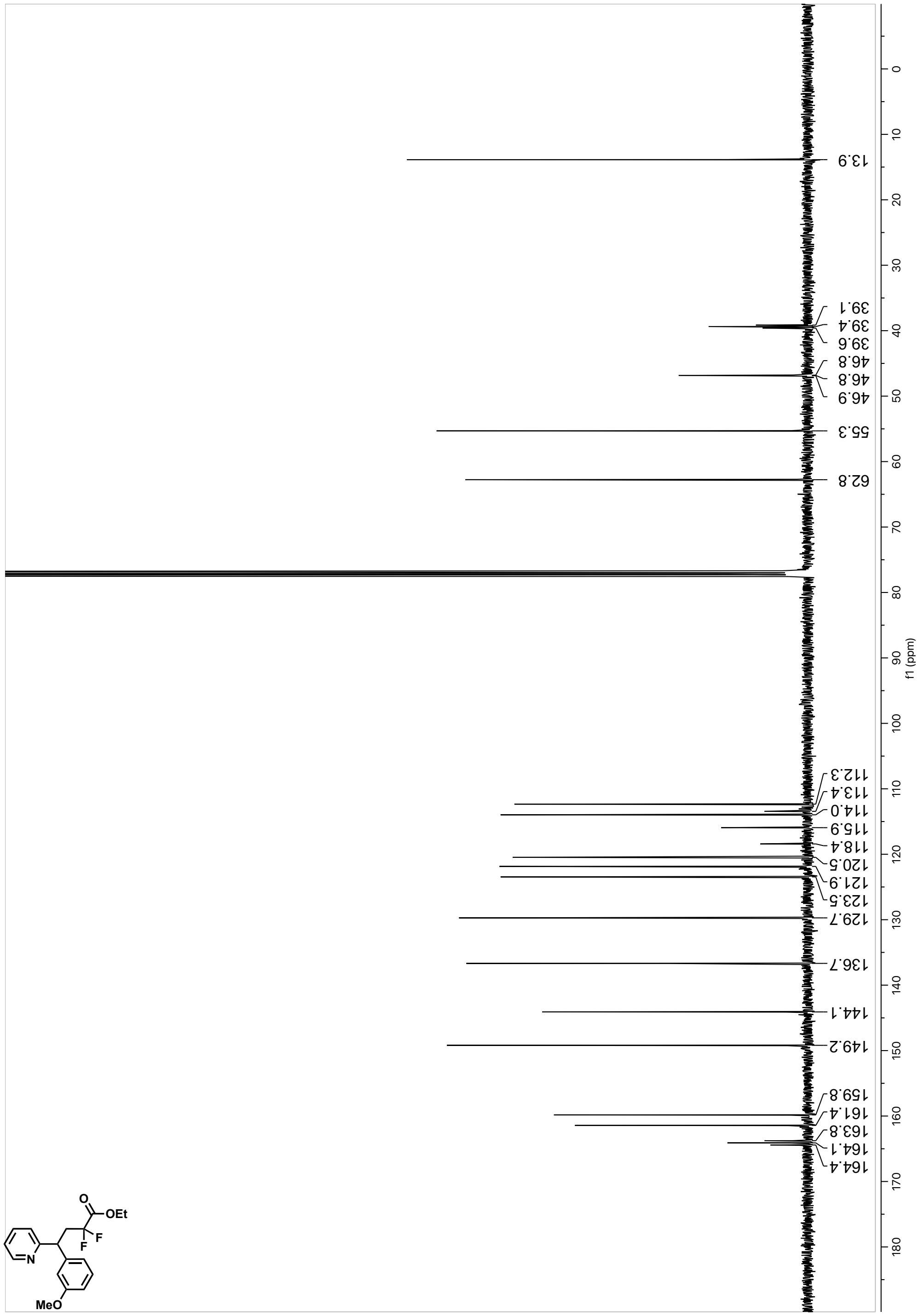

Compound 4x. ^{19}F NMR ($CDCl_3$, 376 MHz).

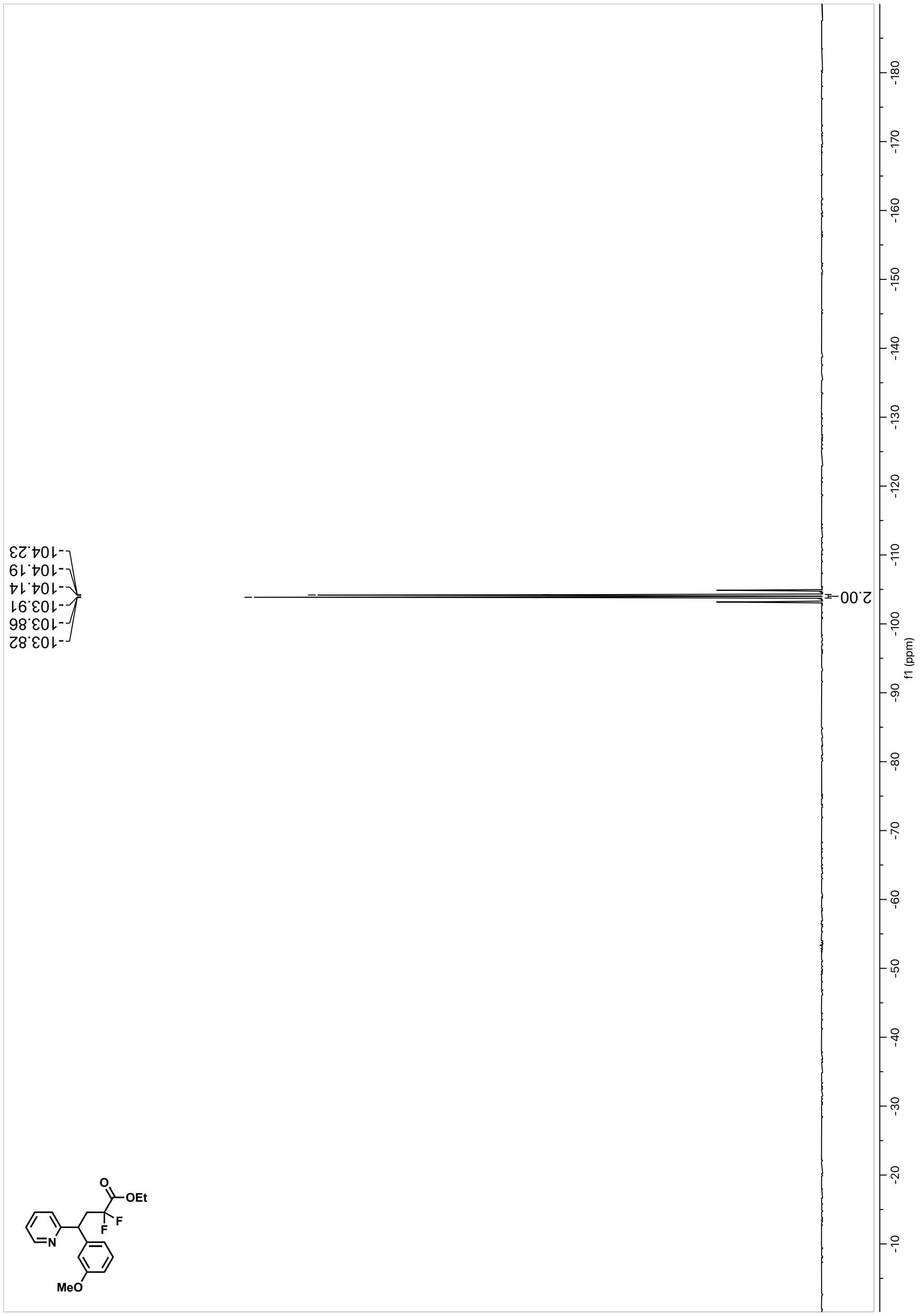

Compound 4y. ^{1}H NMR ($CDCl_3$, 400 MHz).

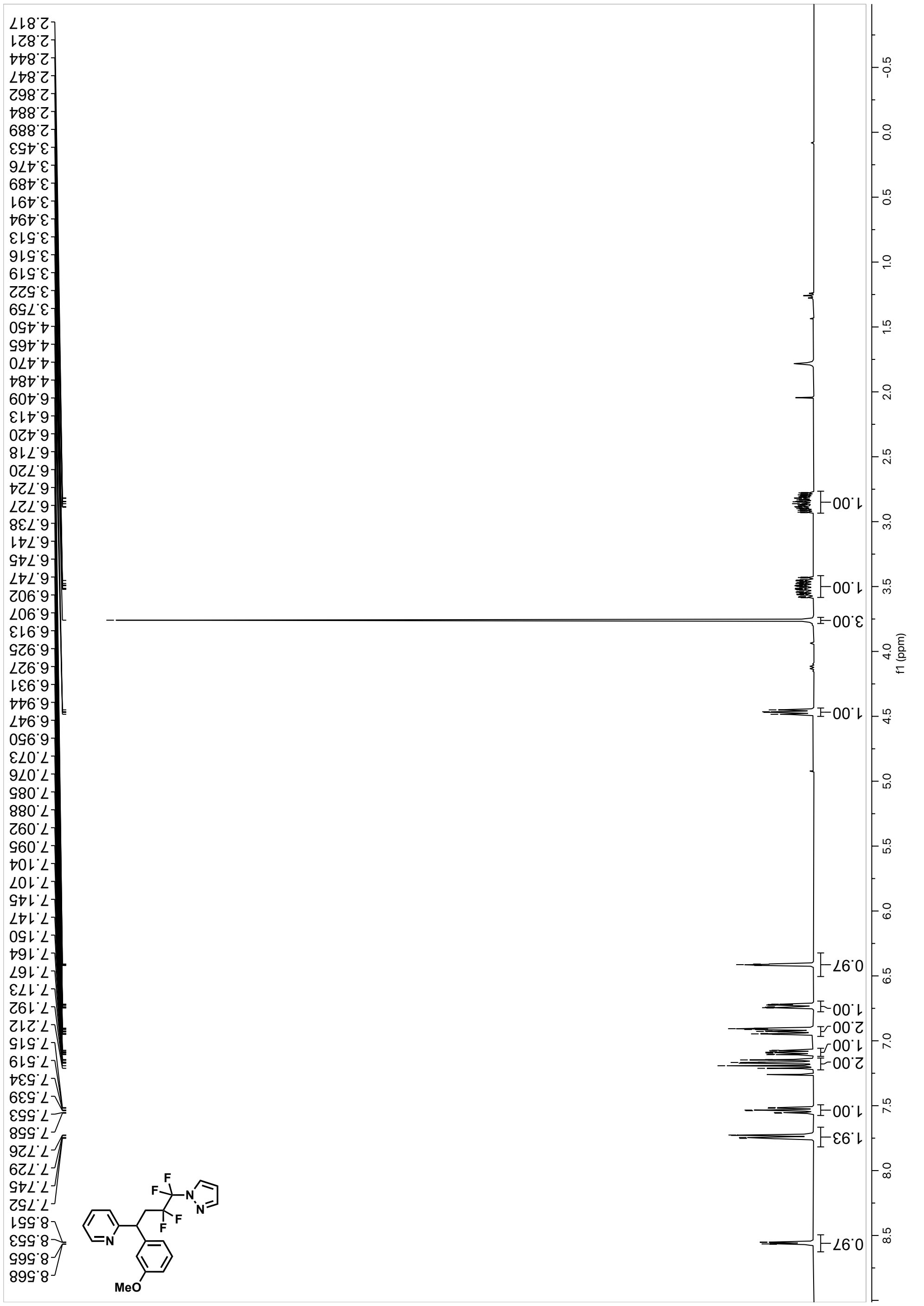

Compound 4y. ^{13}C NMR ($CDCl_3$, 100 MHz).

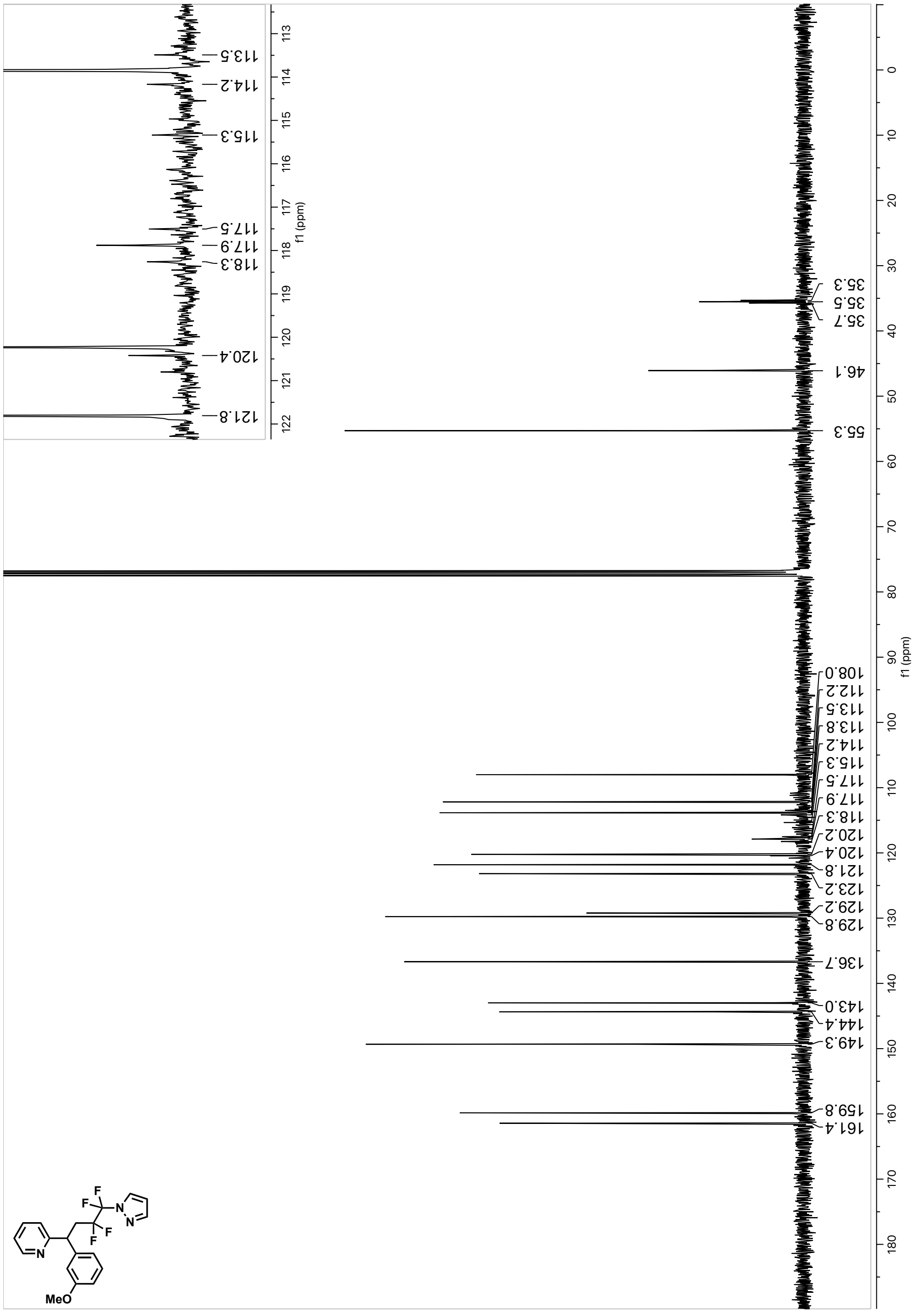

Compound 4y. ^{19}F NMR ($CDCl_3$, 376 MHz).

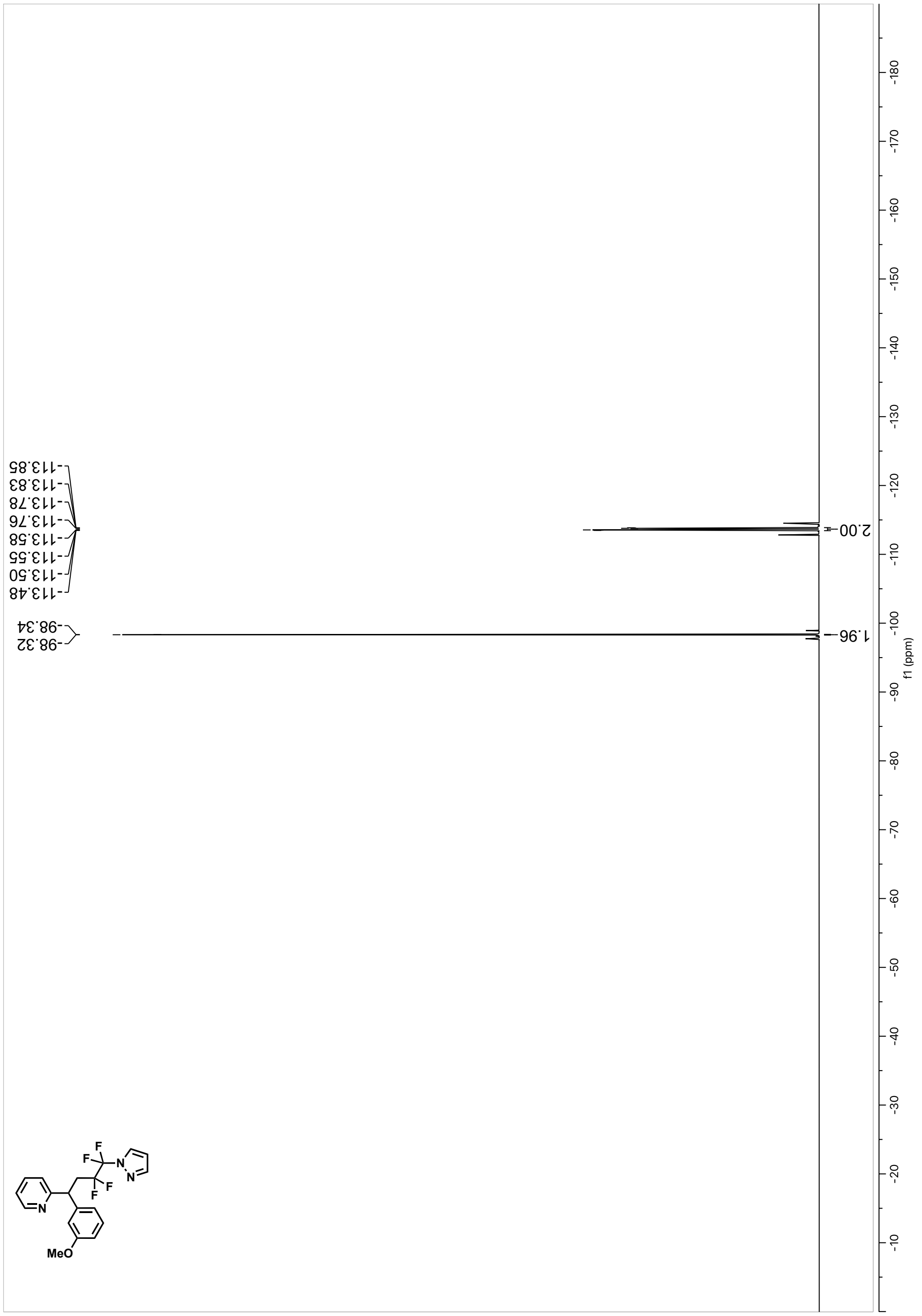

Compound 4z. ^{1}H NMR ($CDCl_3$, 400 MHz).

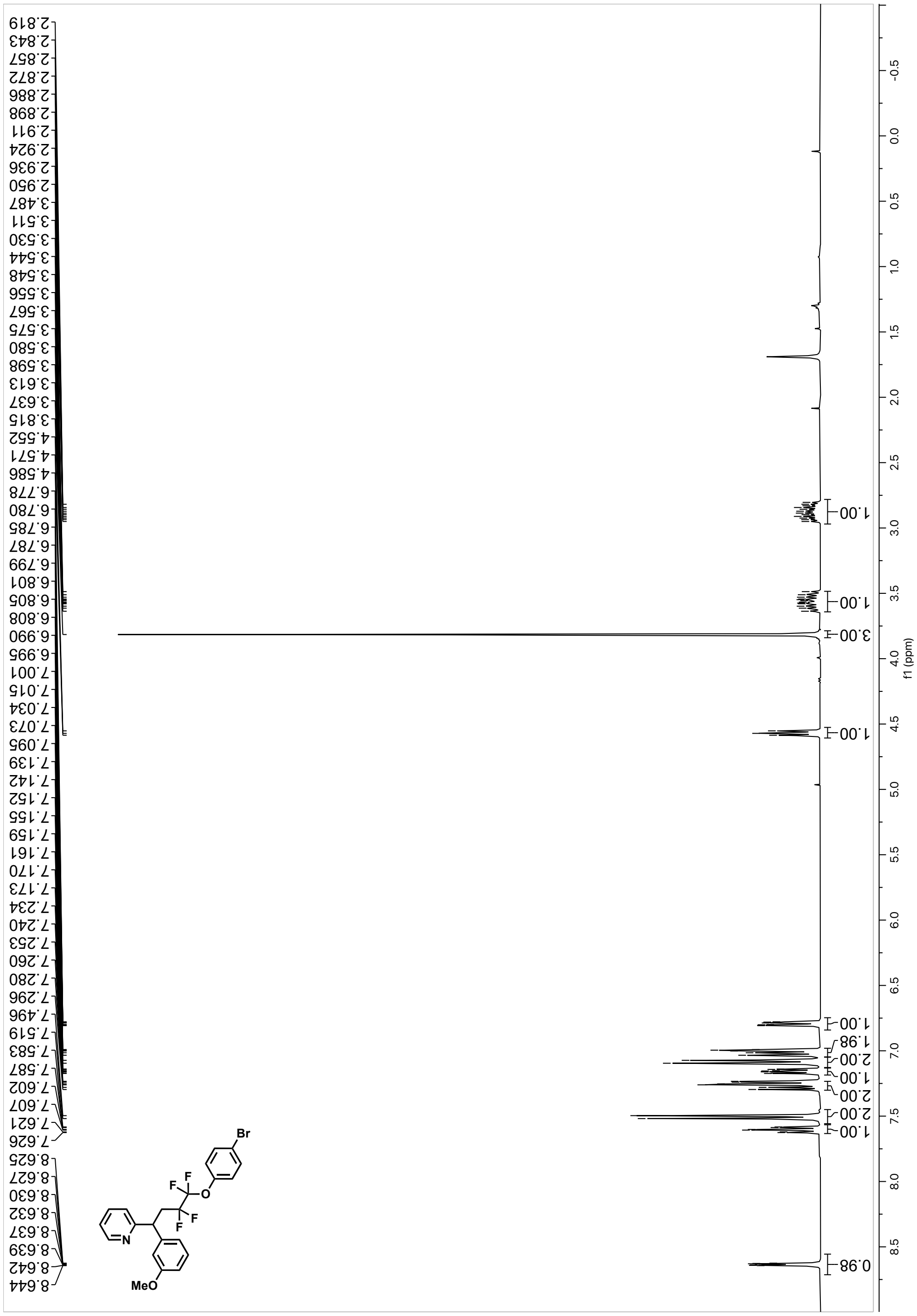

Compound 4z. ^{13}C NMR ($CDCl_3$, 100 MHz).

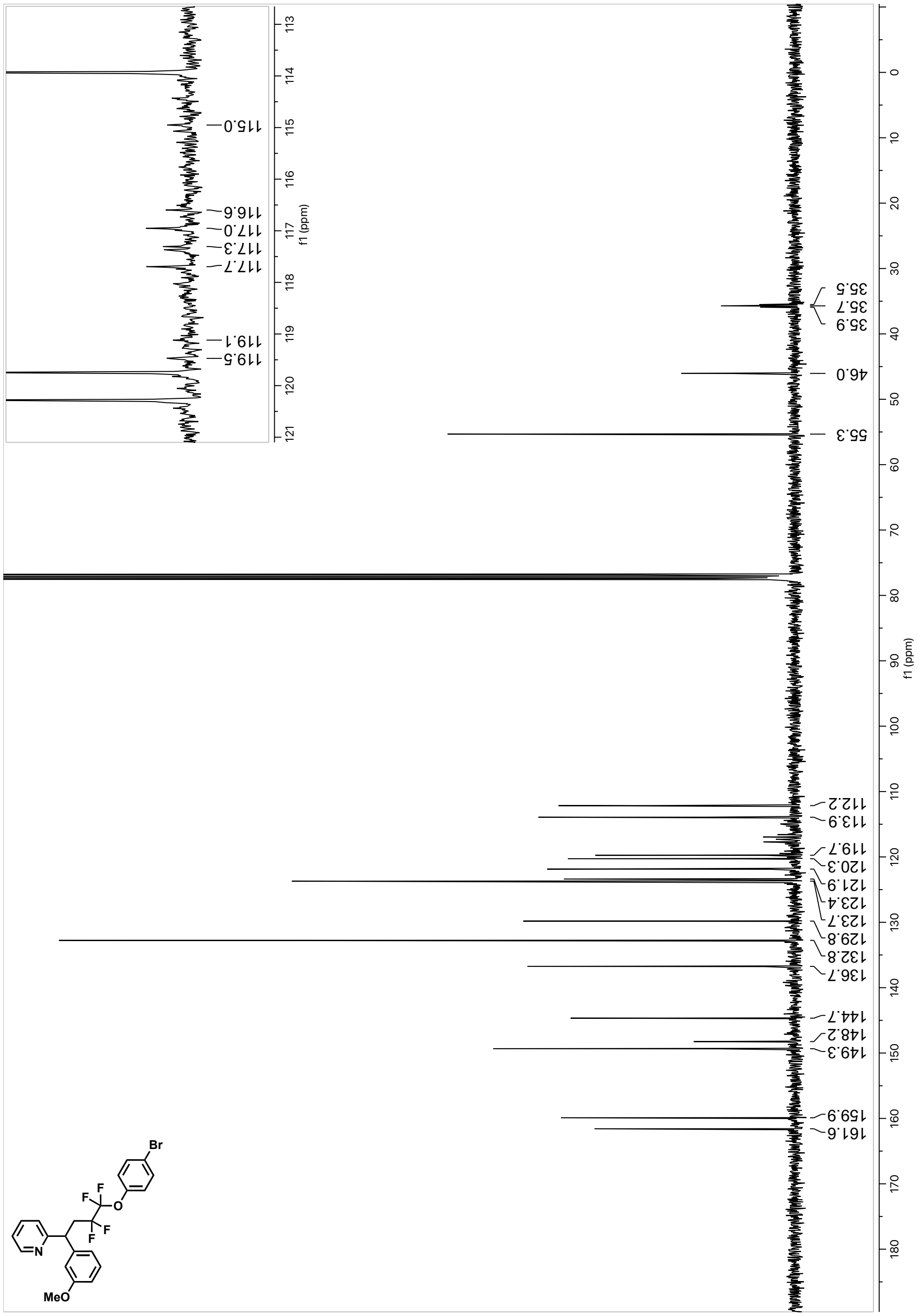

Compound 4z. ^{19}F NMR (CDCl$_3$, 376 MHz).

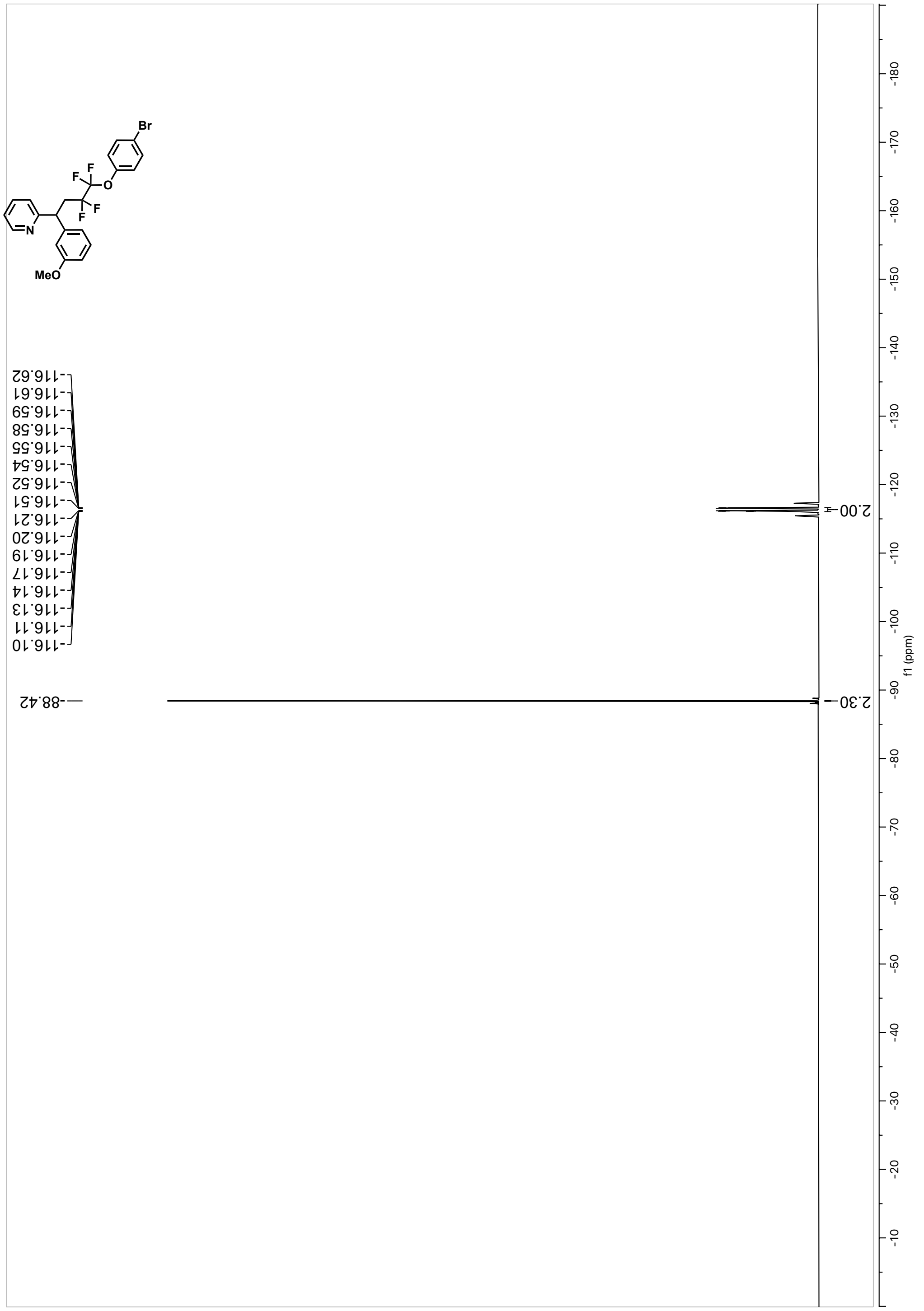

Compound 4z-a. ^{1}H NMR ($CDCl_3$, 400 MHz).

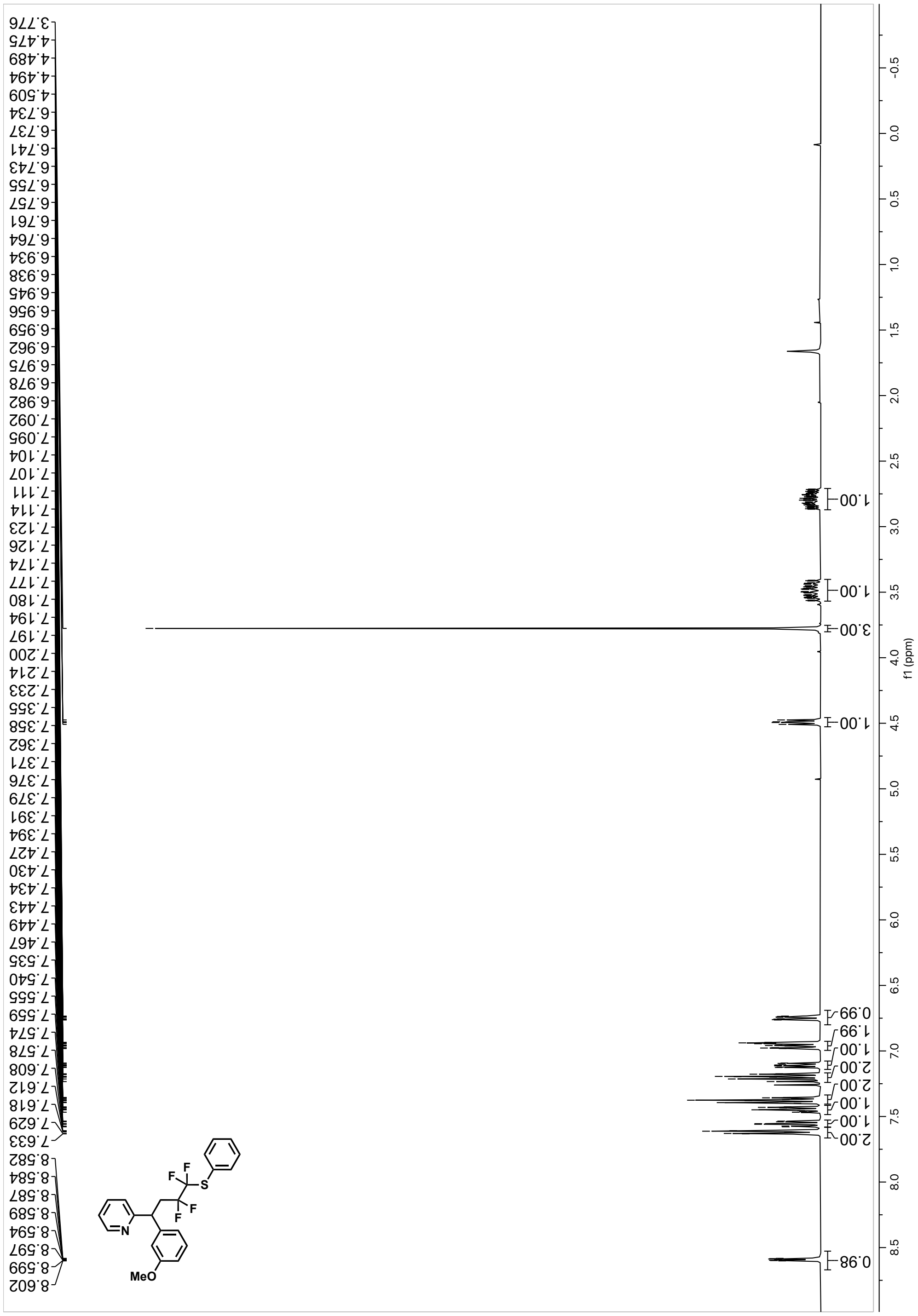

Compound 4z-a. ^{13}C NMR ($CDCl_3$, 100 MHz).

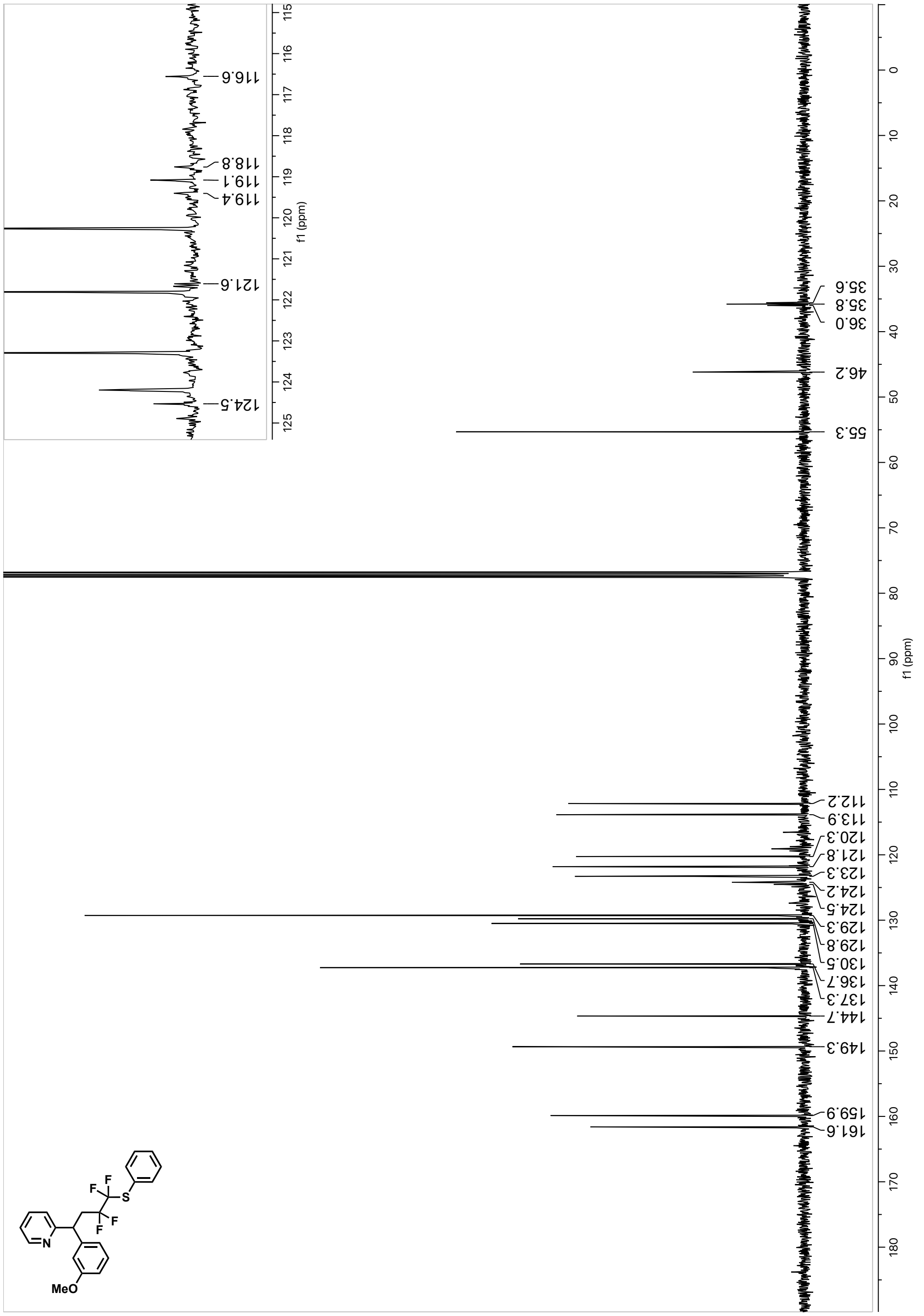

Compound 4z-a. ^{19}F NMR ($CDCl_3$, 376 MHz).

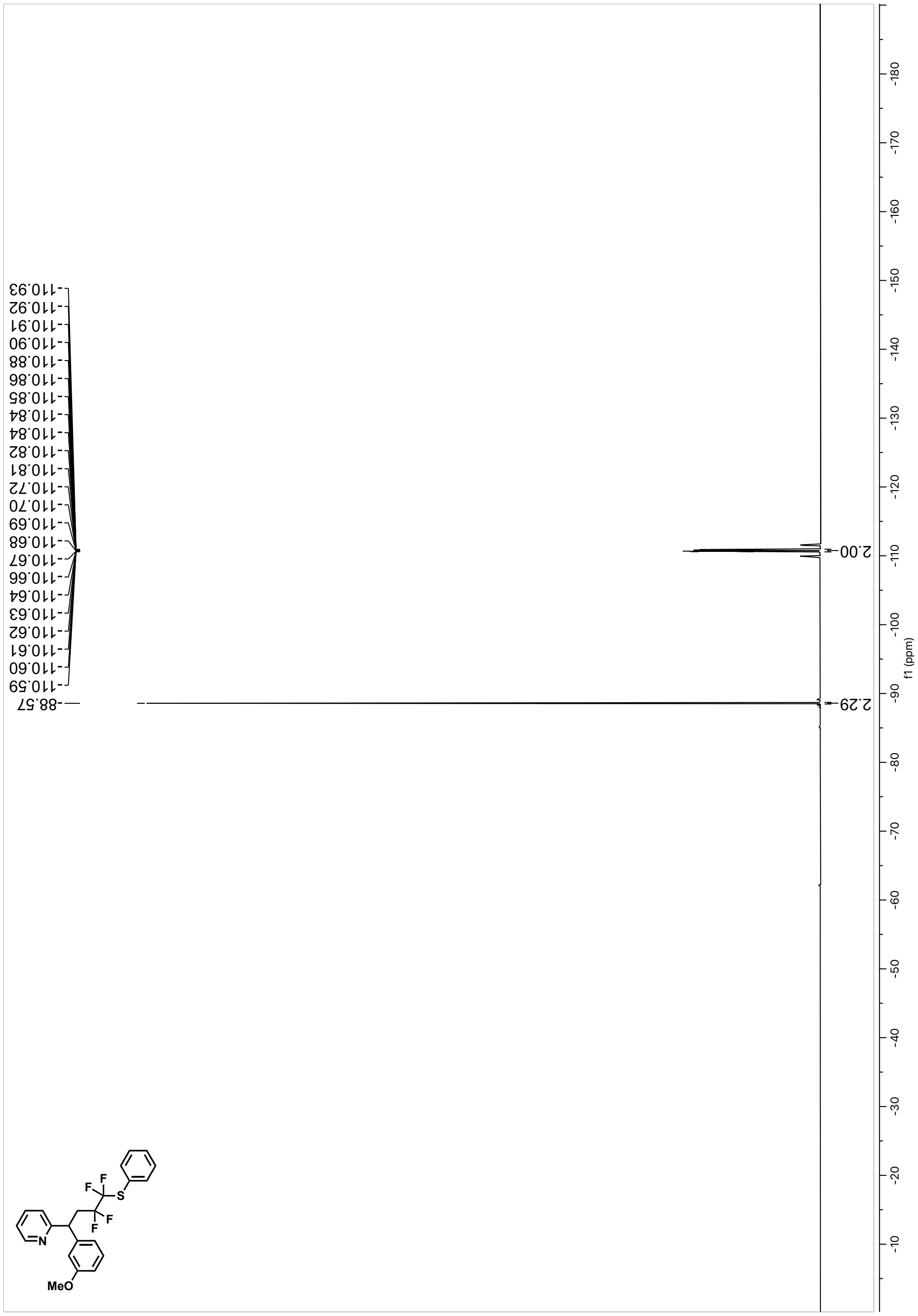

Compound 4z-b. ^{1}H NMR ($CDCl_3$, 400 MHz).

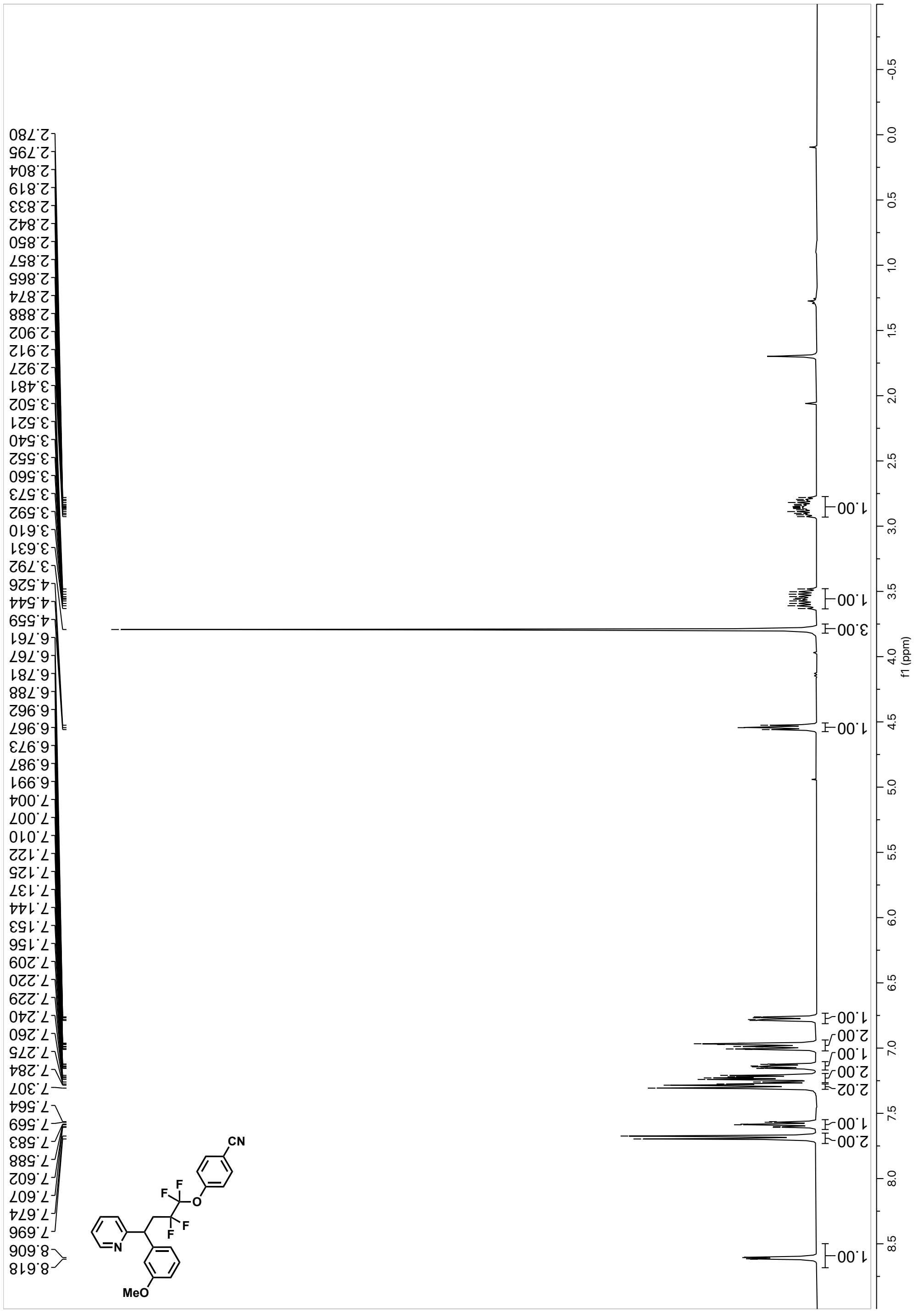

Compound 4z-b. ^{13}C NMR ($CDCl_3$, 100 MHz).

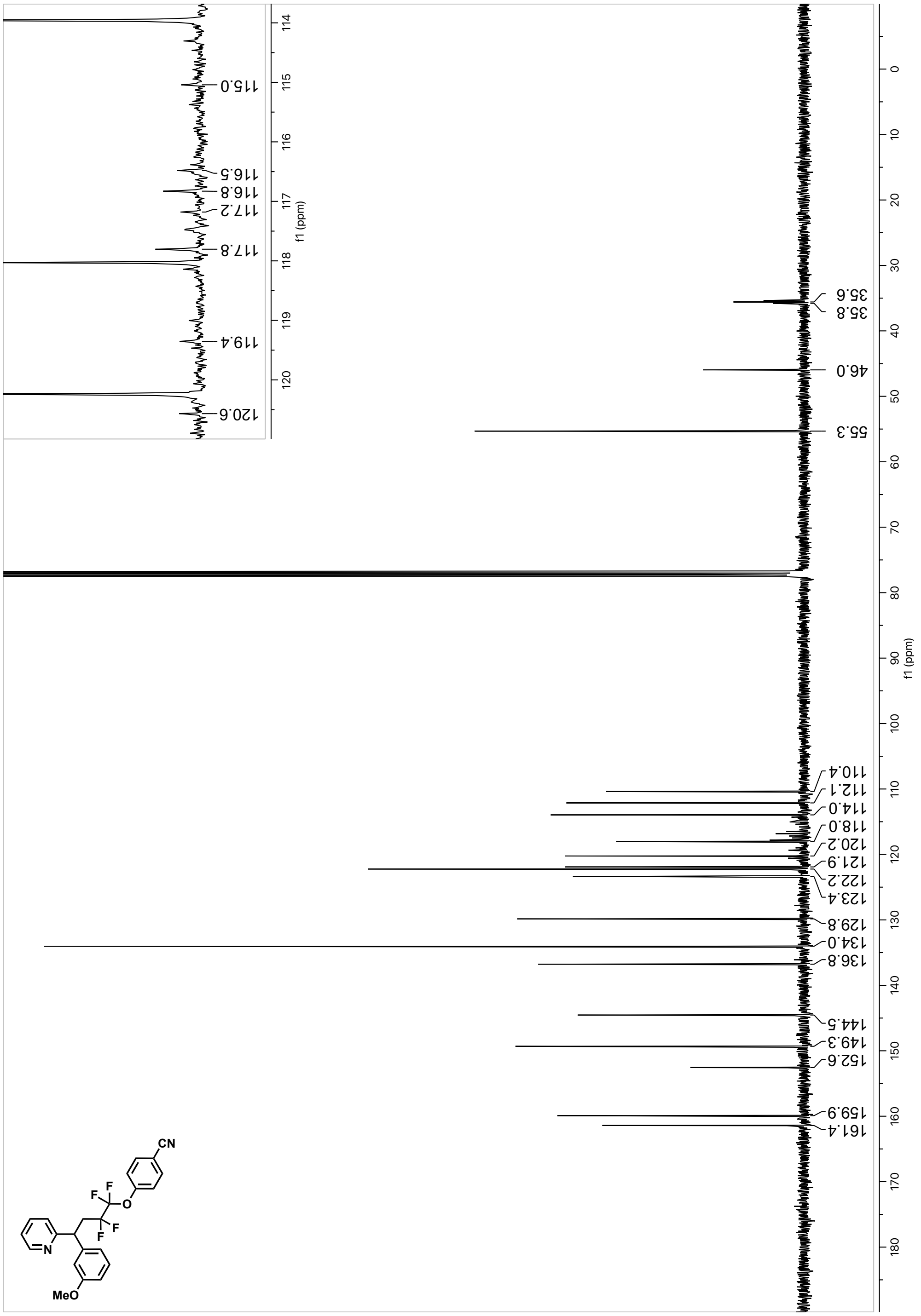

Compound 4z-b. ^{19}F NMR ($CDCl_3$, 376 MHz).

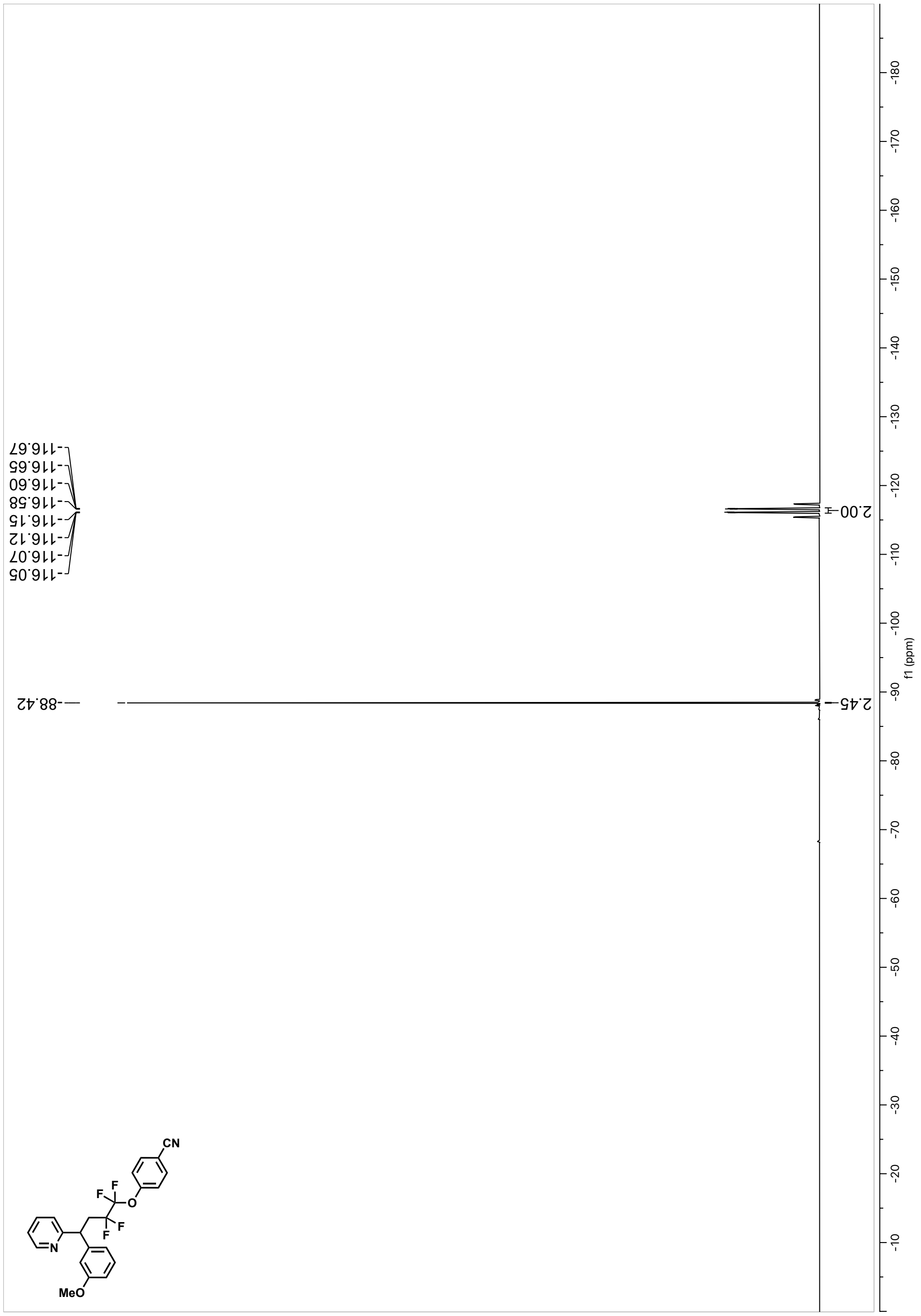

Compound 4z-c. ^{1}H NMR ($CDCl_3$, 400 MHz).

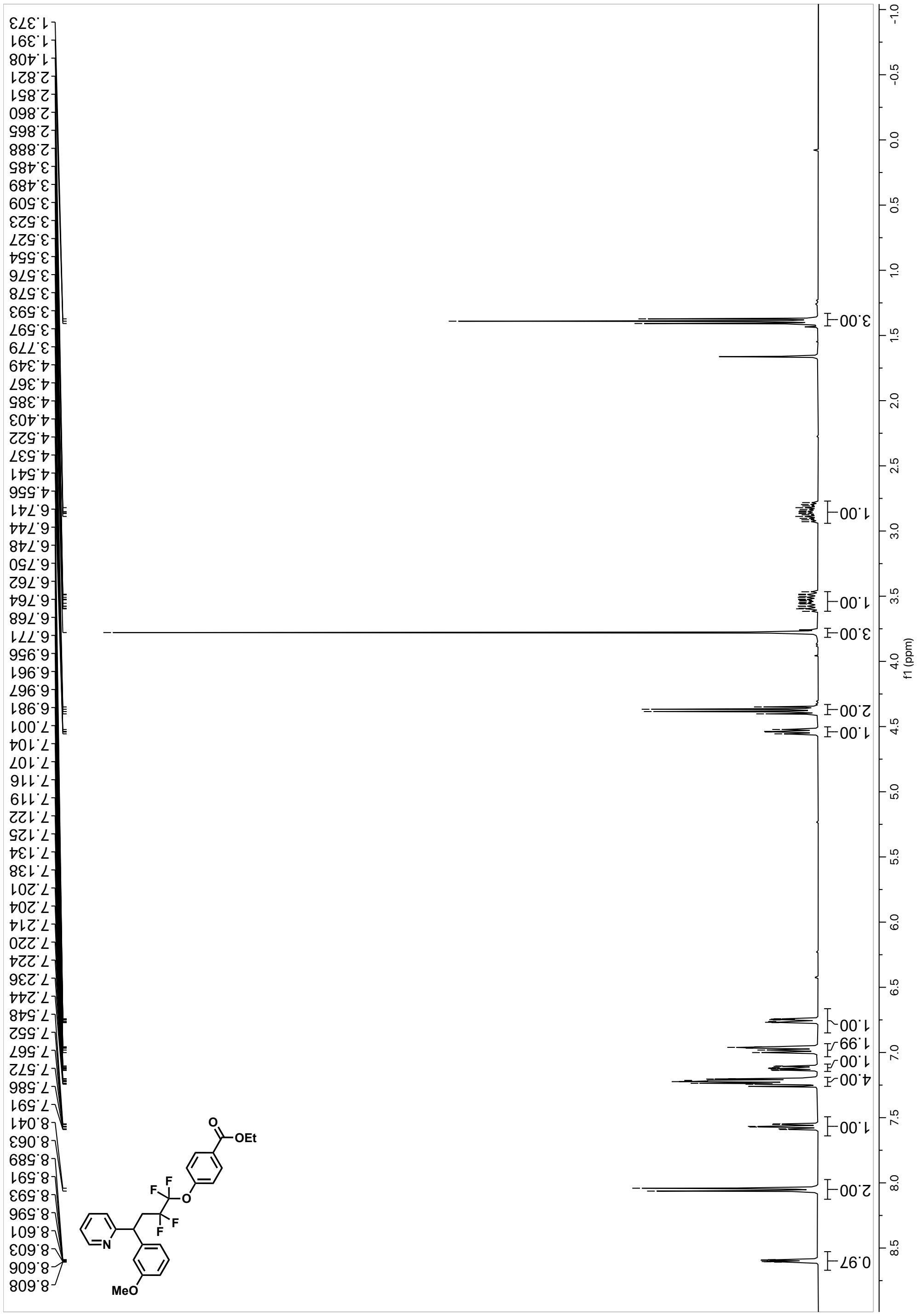

Compound 4z-c. ^{13}C NMR ($CDCl_3$, 100 MHz).

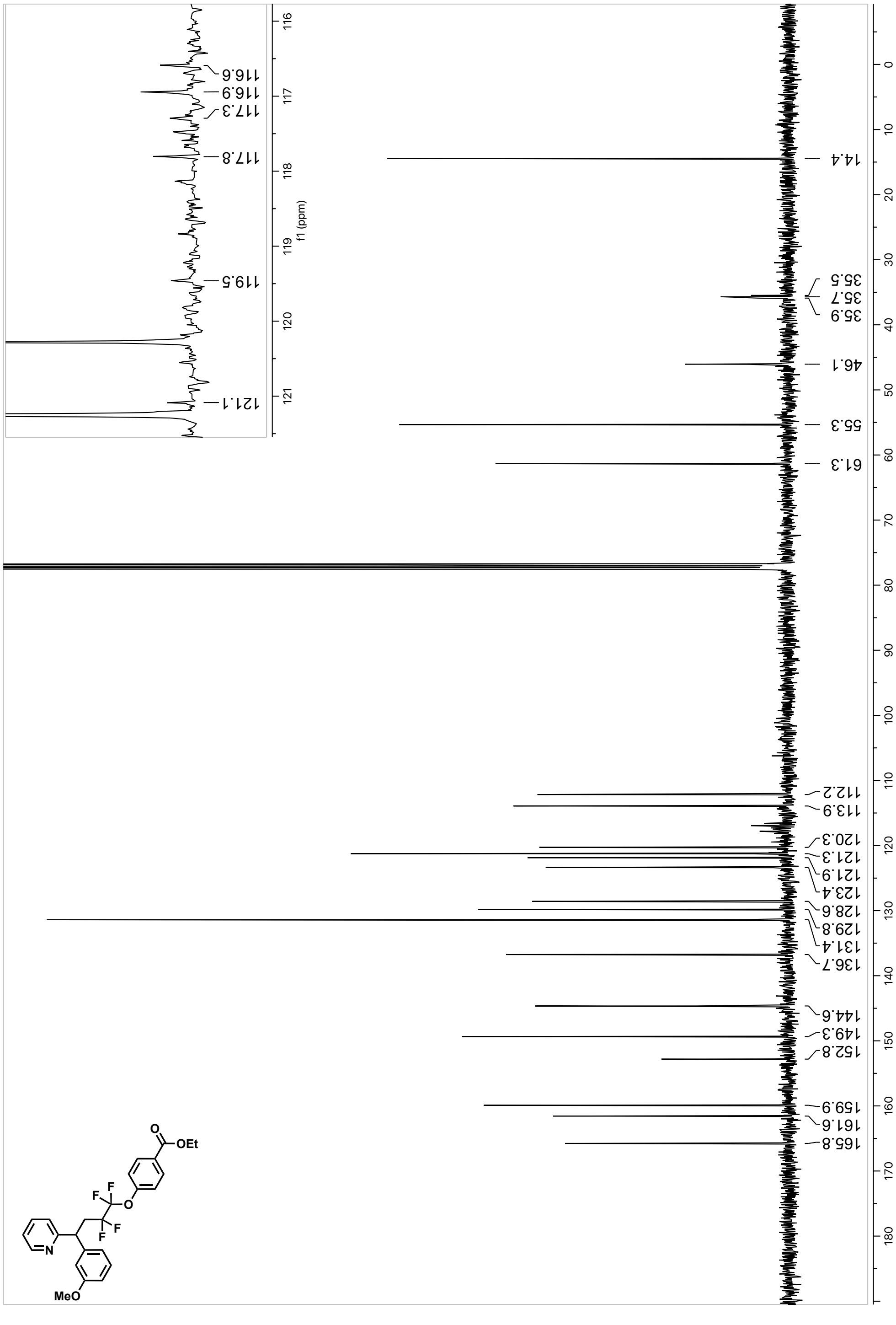

Compound 4z-c. ^{19}F NMR ($CDCl_3$, 376 MHz).

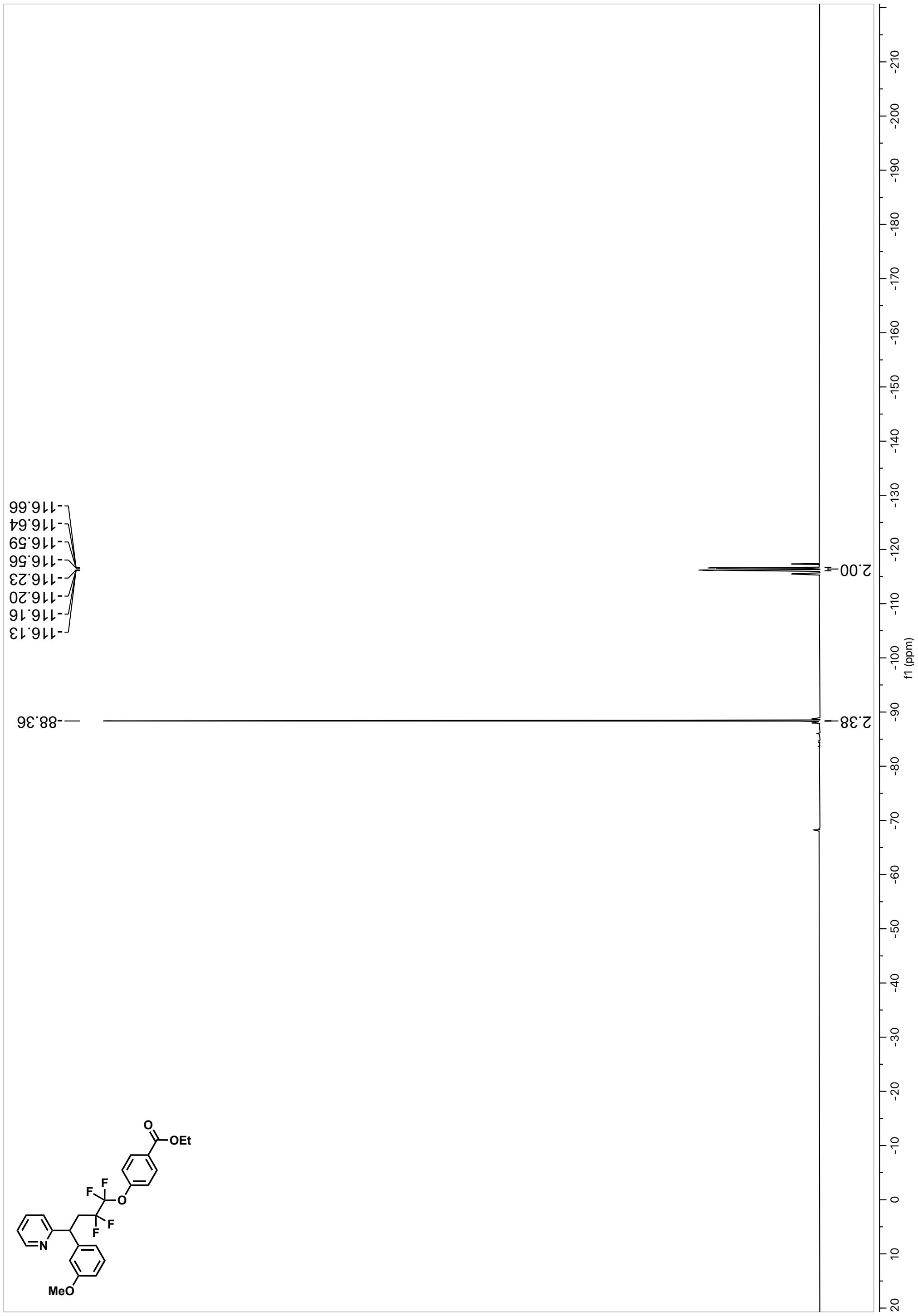

Compound 4z-d. ^{1}H NMR ($CDCl_3$, 400 MHz).

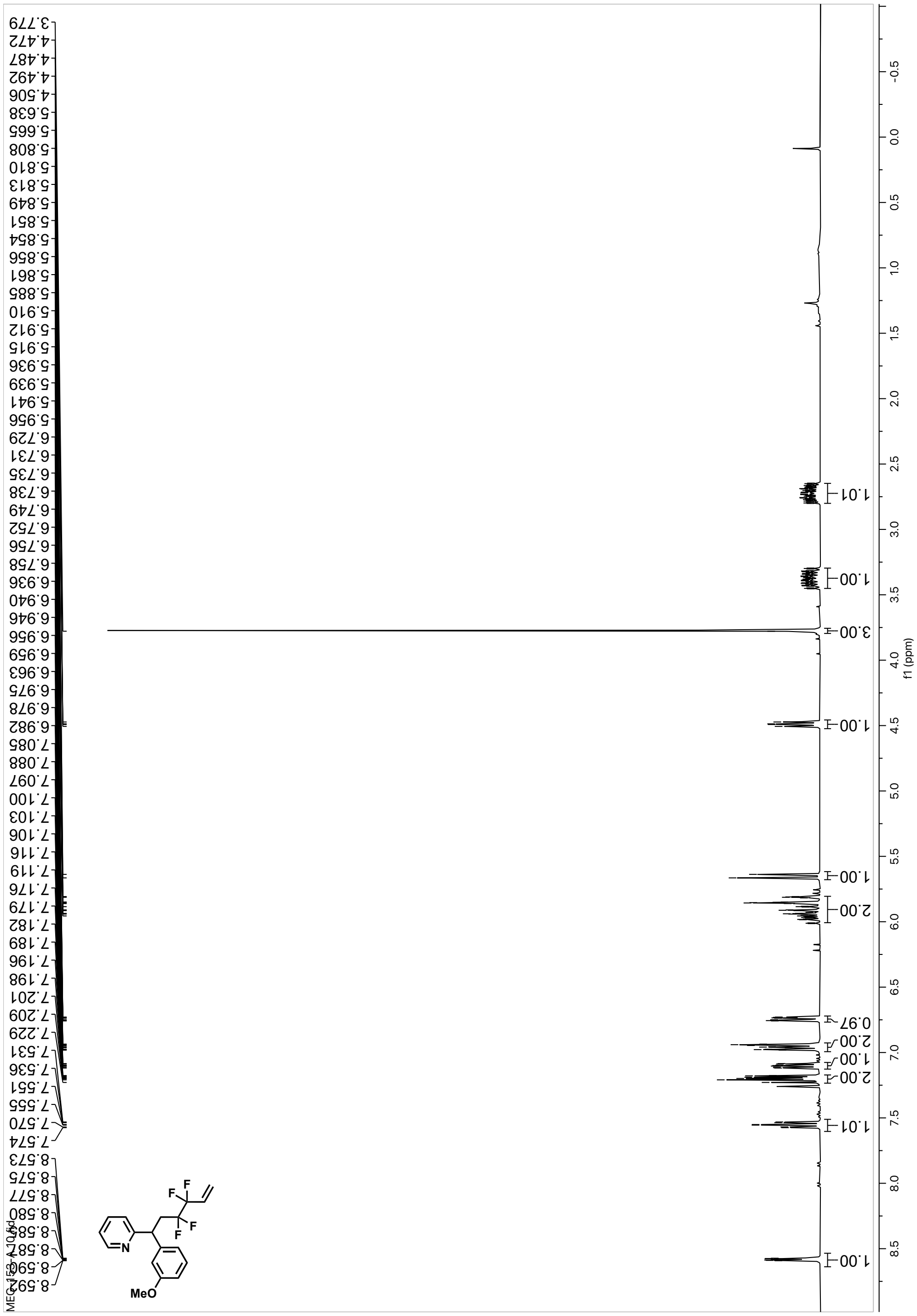

Compound 4z-d. ^{13}C NMR ($CDCl_3$, 100 MHz).

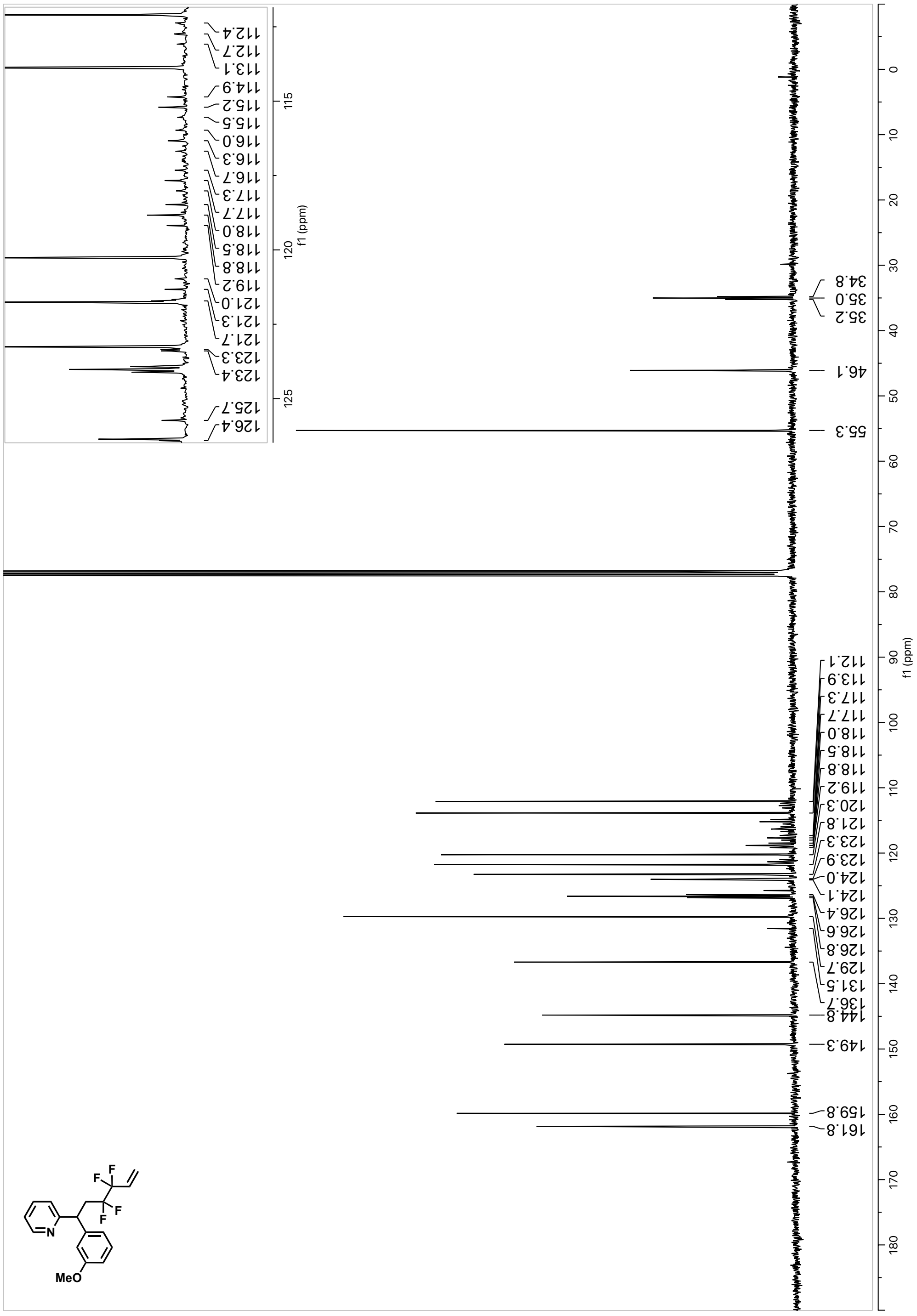

Compound 4z-d. ^{19}F NMR ($CDCl_3$, 376 MHz).

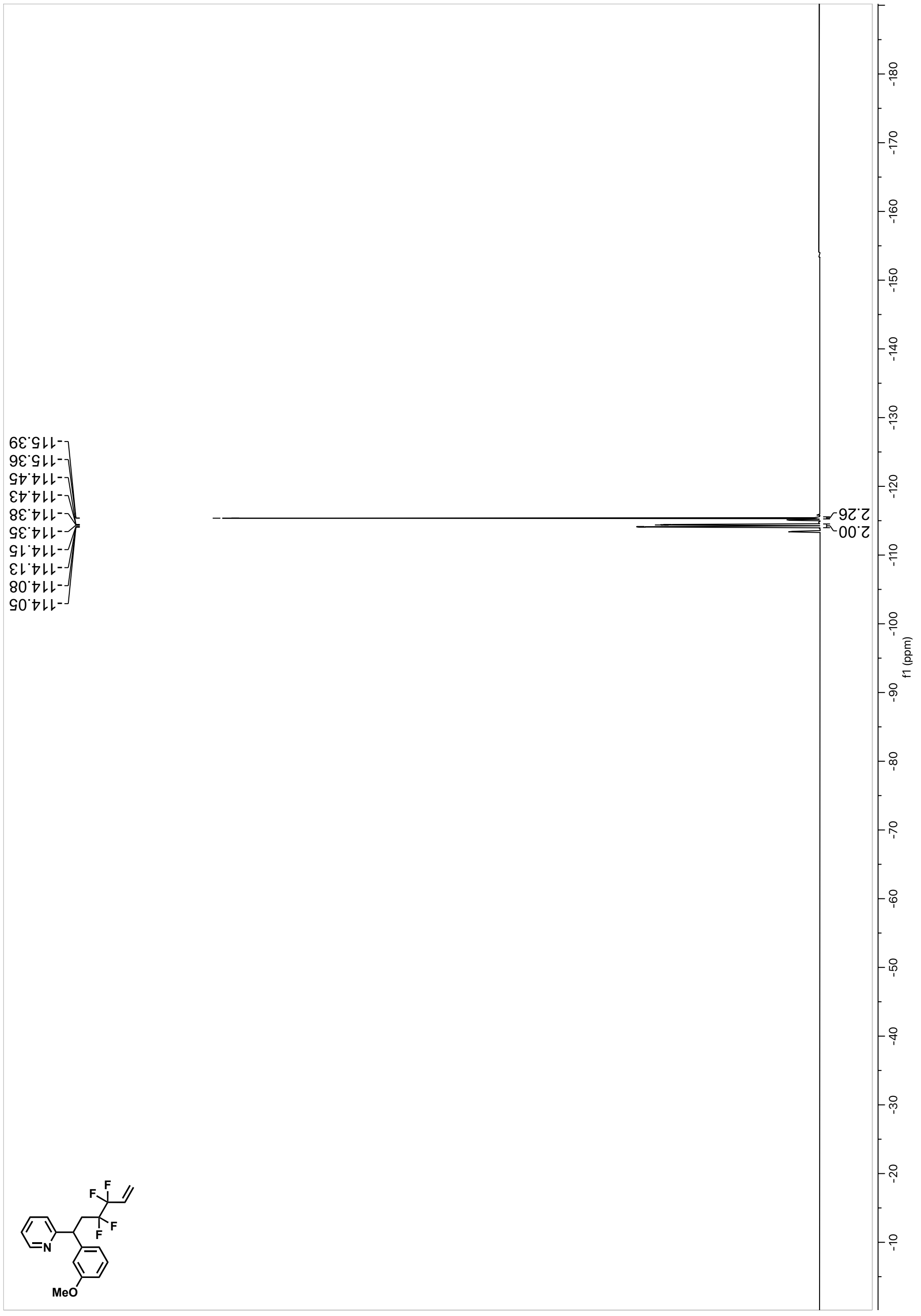

Compound 4z-e. ^{1}H NMR ($CDCl_3$, 400 MHz).

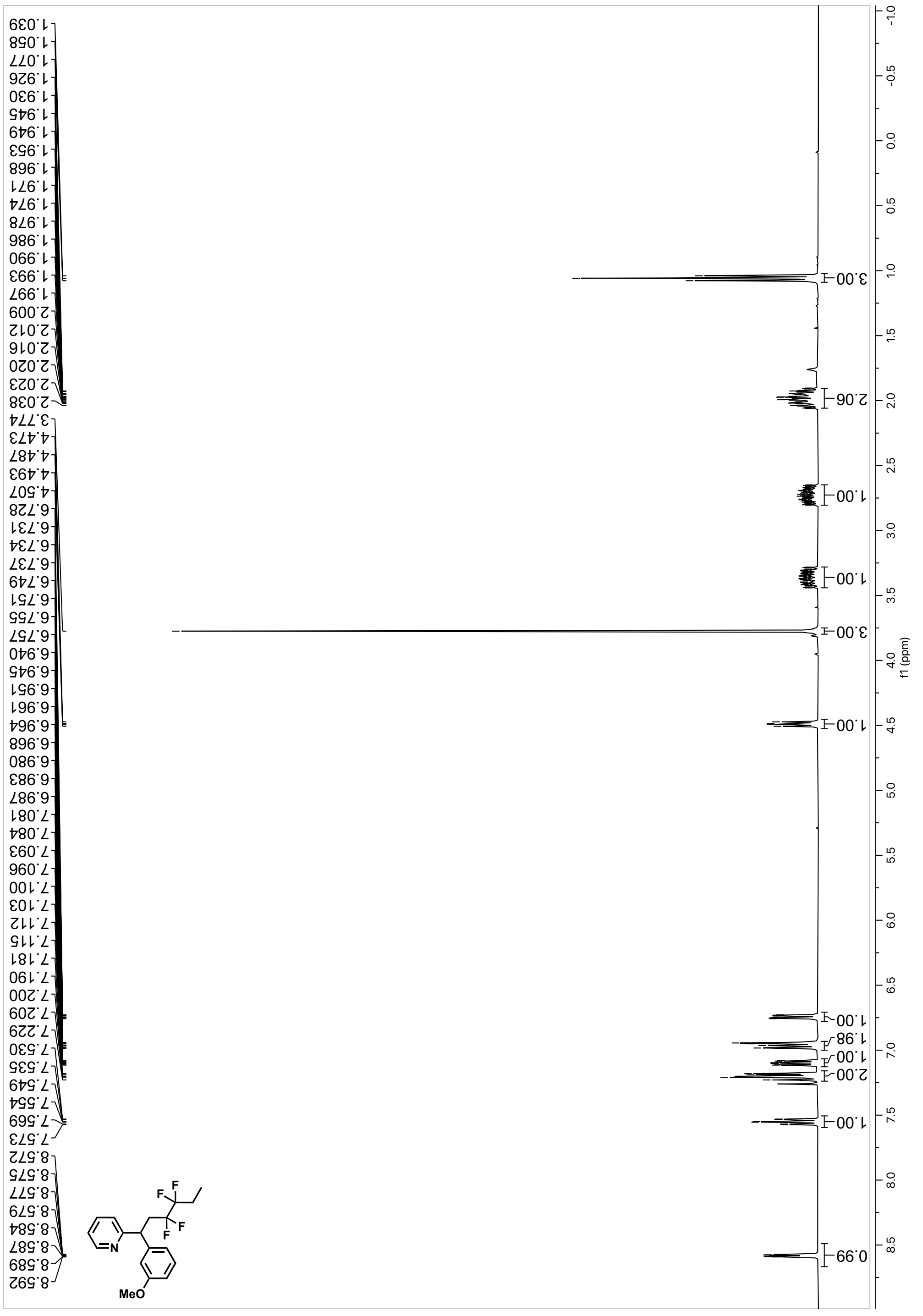

Compound 4z-e. ^{13}C NMR ($CDCl_3$, 100 MHz).

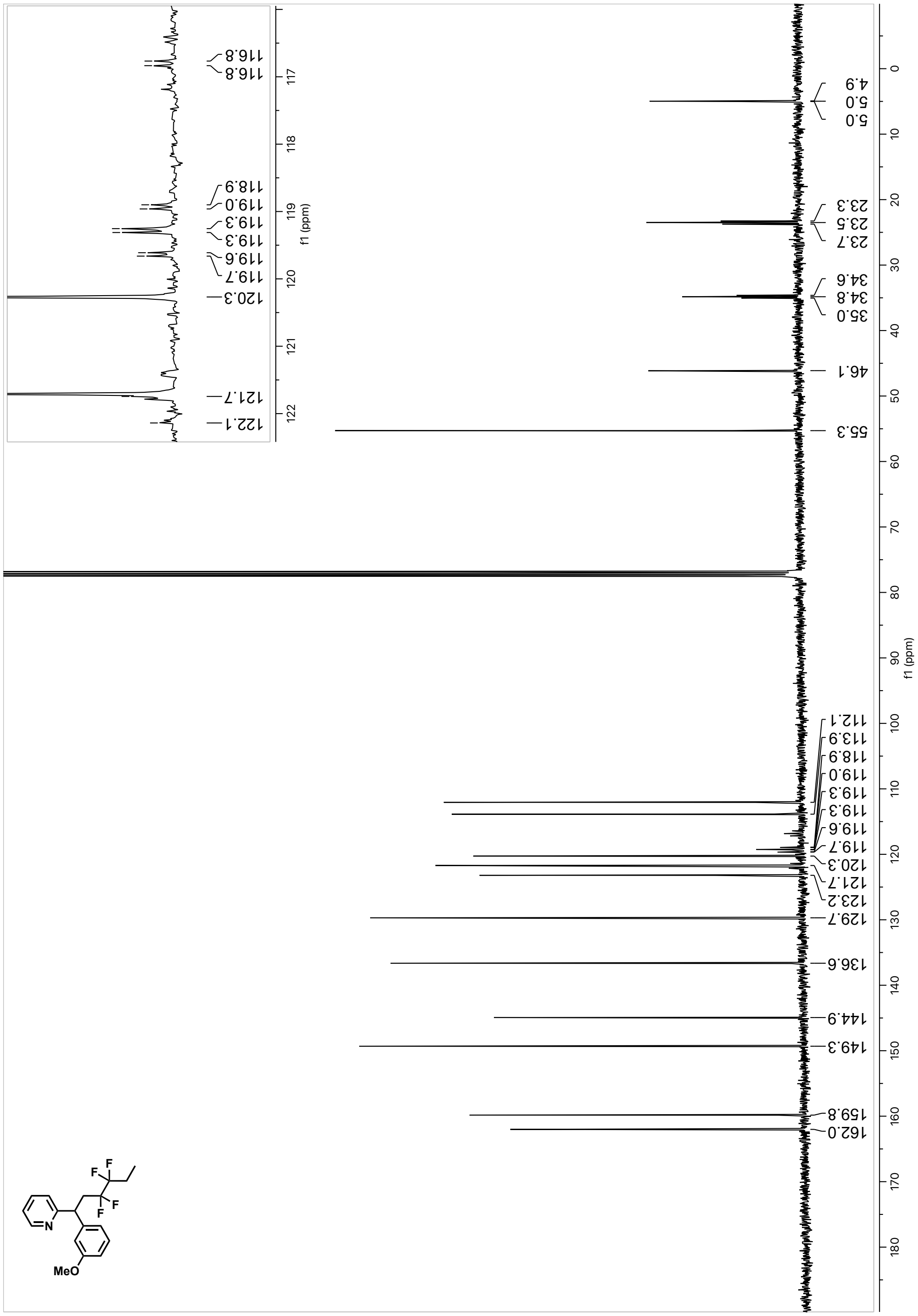

Compound 4z-e. ^{19}F NMR ($CDCl_3$, 376 MHz).

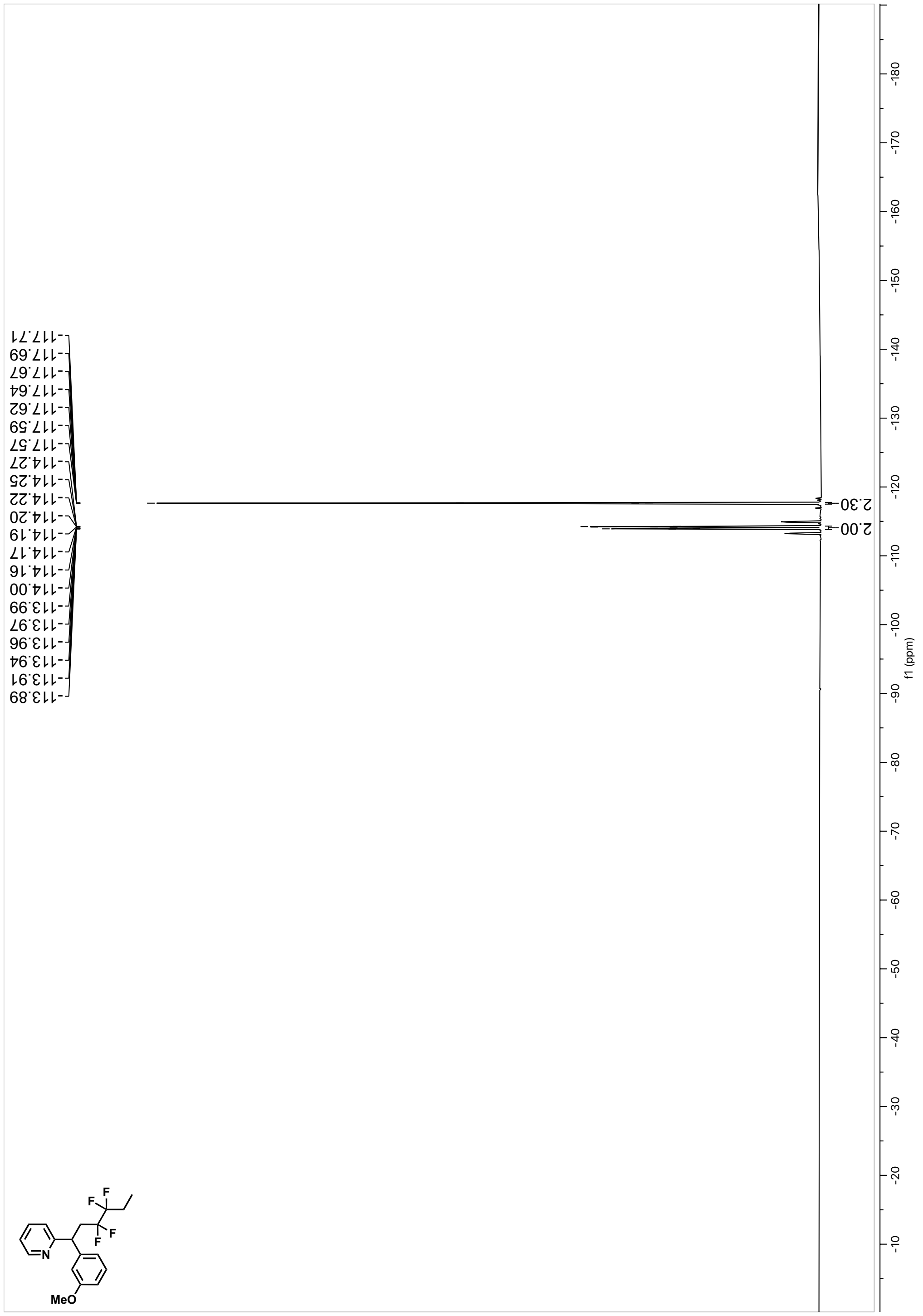

Compound 4z-f. ^{1}H NMR ($CDCl_3$, 400 MHz).

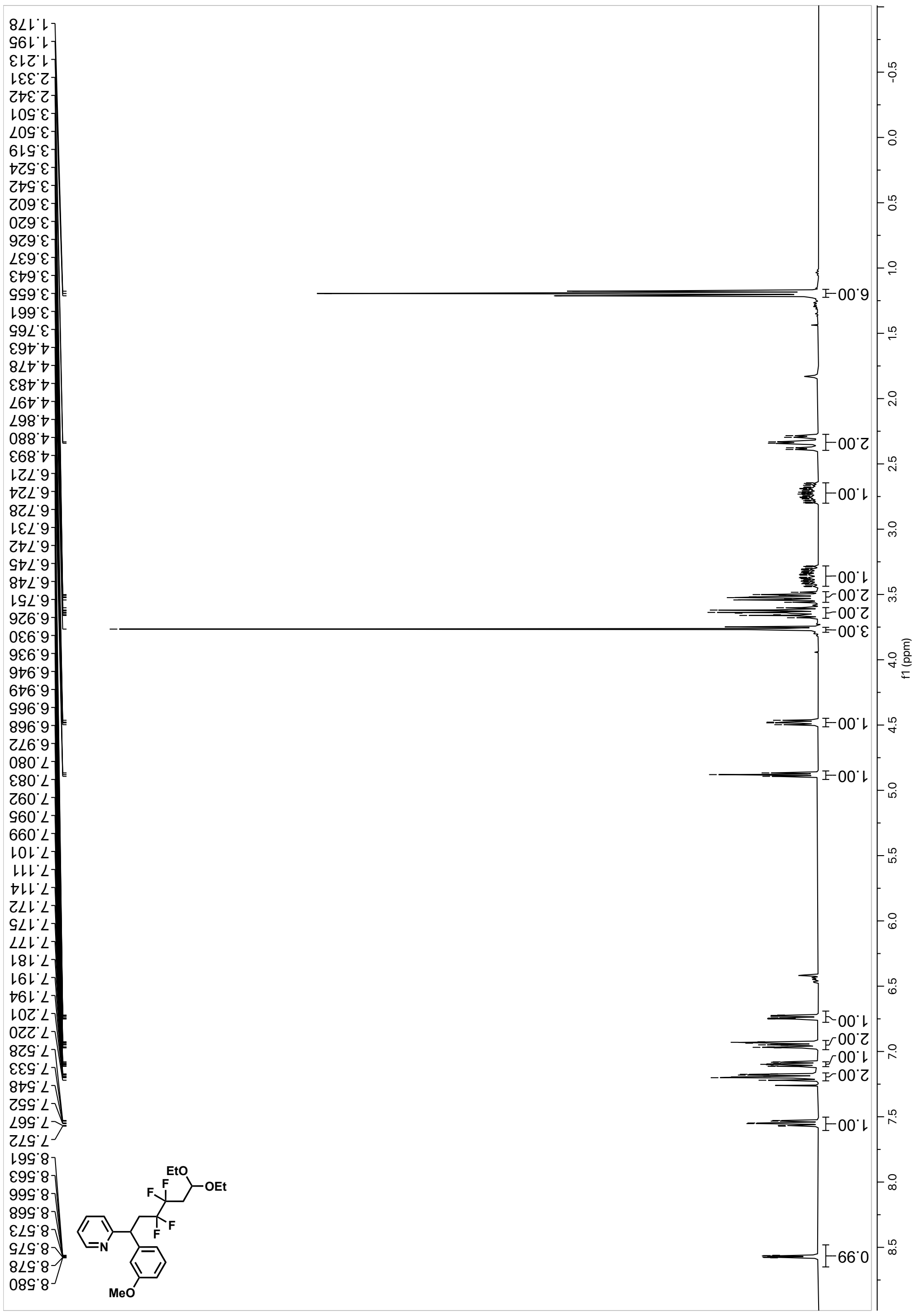

Compound 4z-f. ^{13}C NMR ($CDCl_3$, 100 MHz).

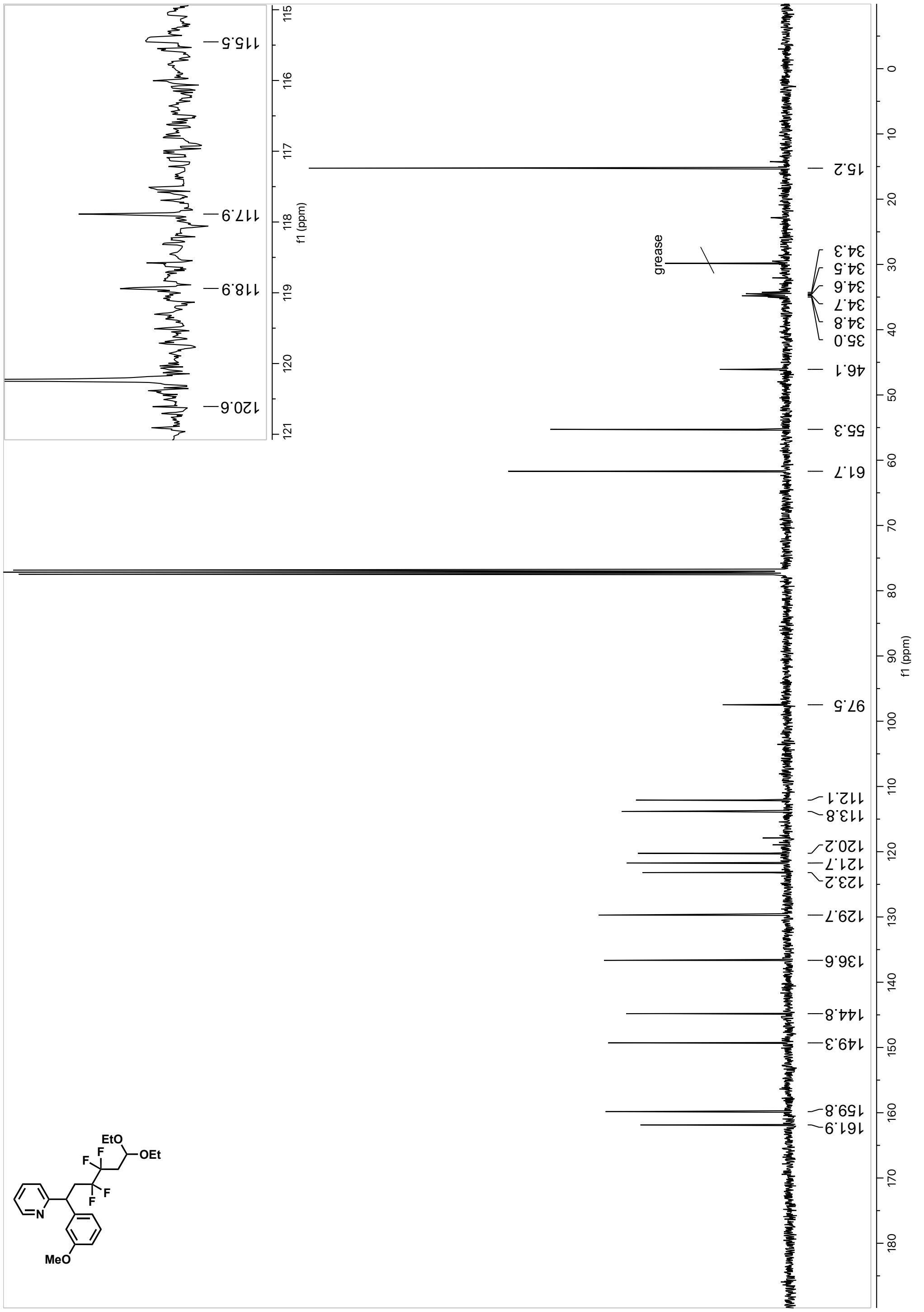

Compound 4z-f. ^{19}F NMR ($CDCl_3$, 376 MHz).

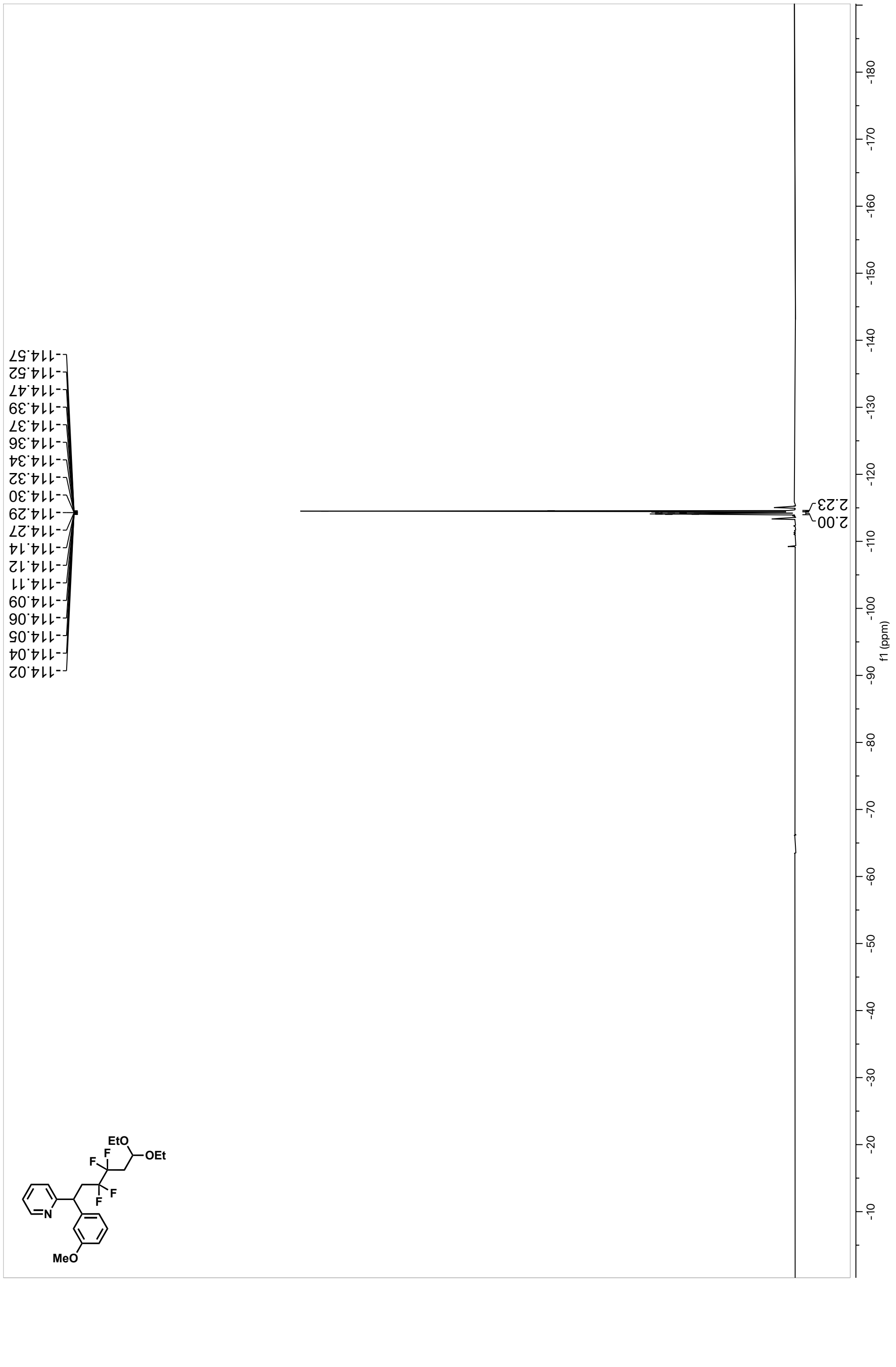

Compound 4z-g. ^{1}H NMR ($CDCl_3$, 400 MHz).

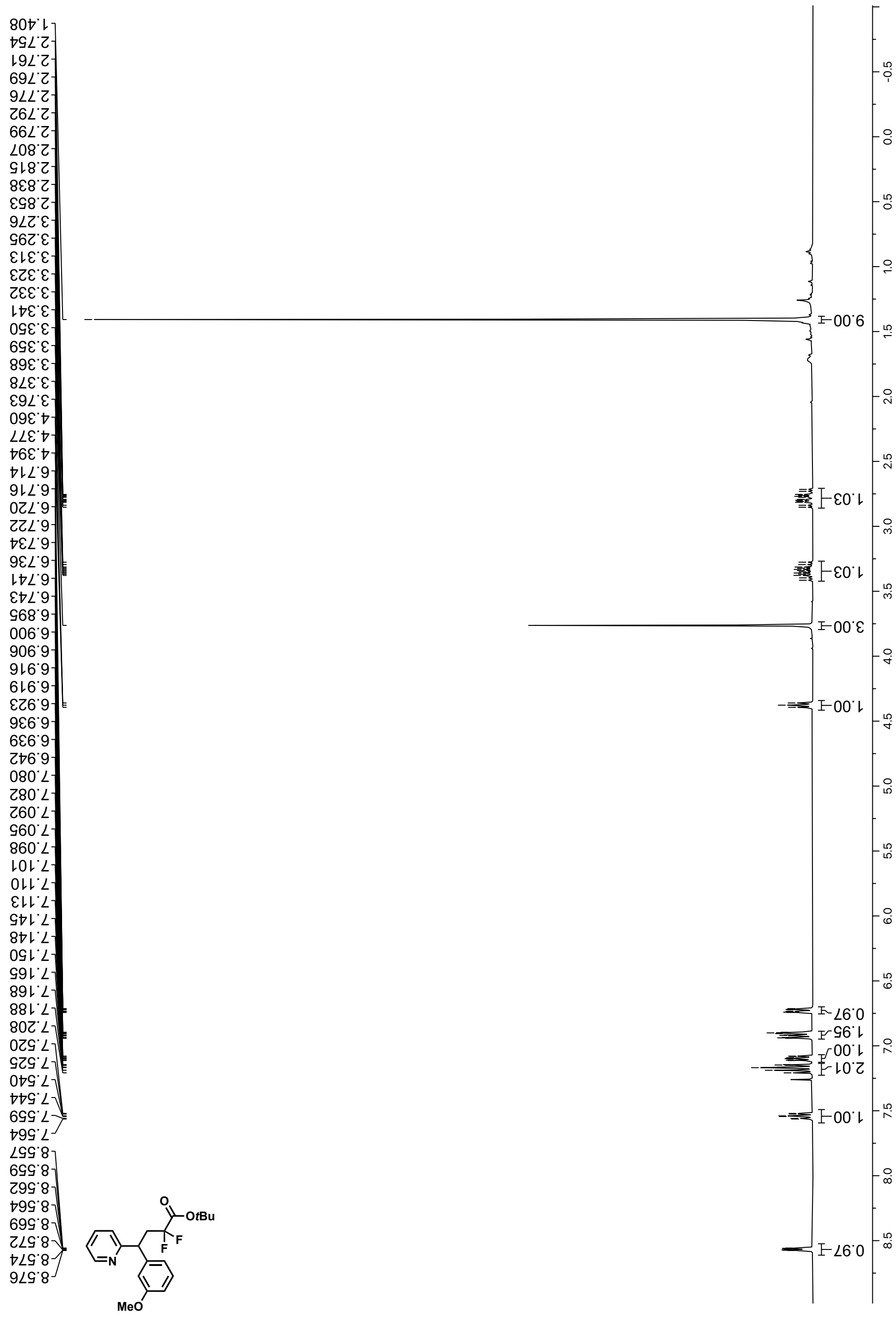

Compound 4z-g. ^{13}C NMR ($CDCl_3$, 100 MHz).

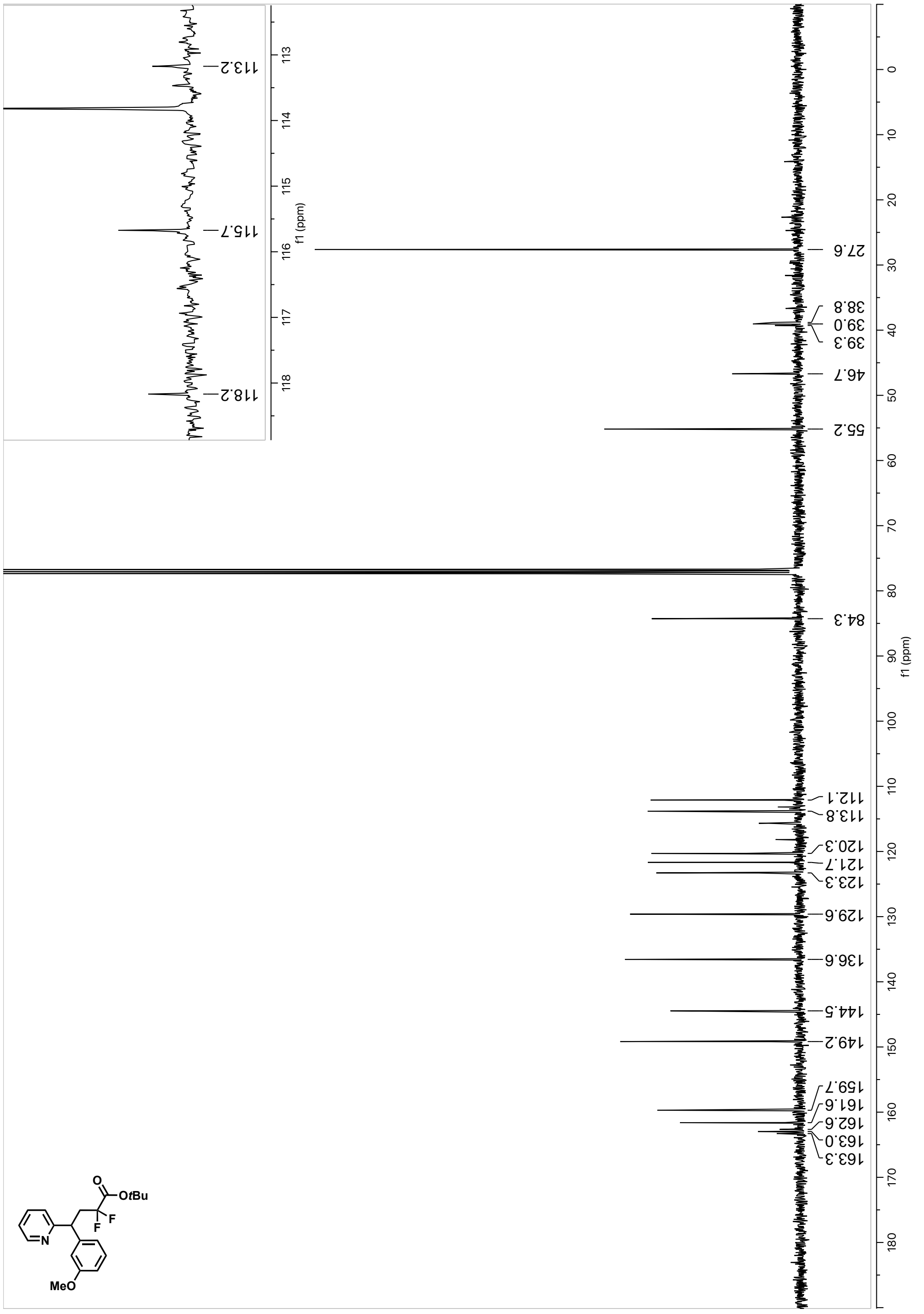

Compound 4z-g. ^{19}F NMR ($CDCl_3$, 376 MHz).

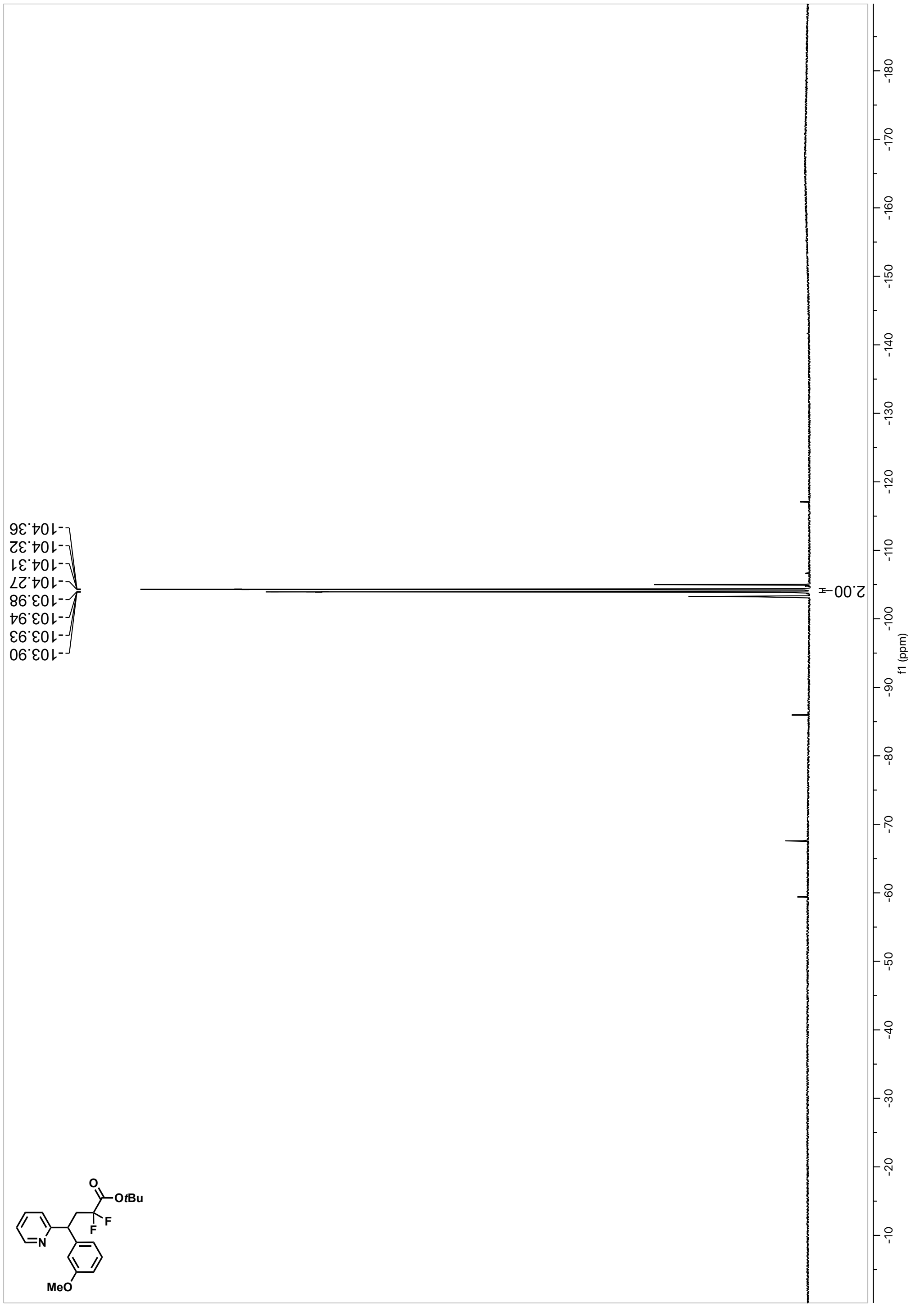

Compound 5a. ^{1}H NMR ($CDCl_3$, 400 MHz).

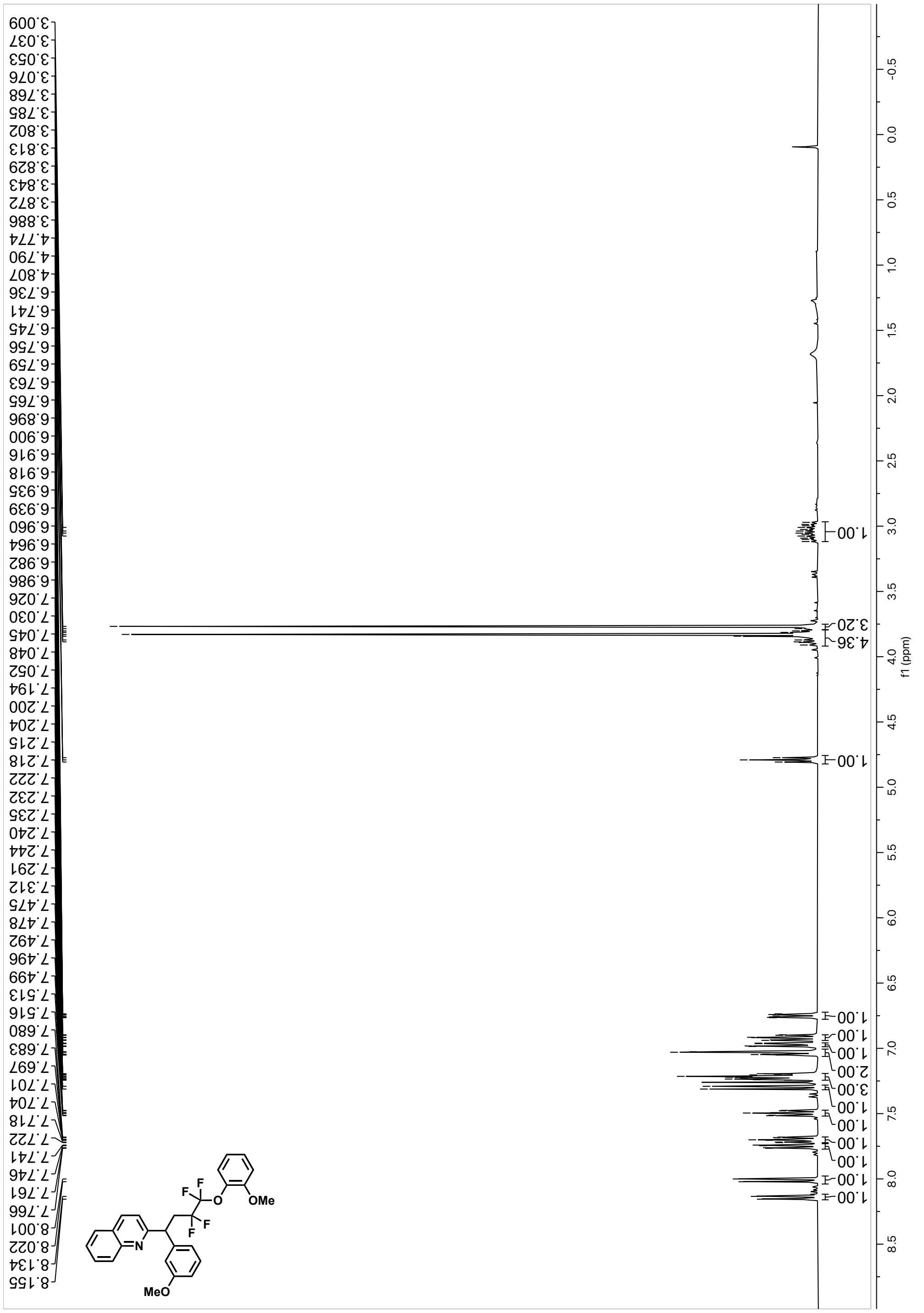

Compound 5a. ^{13}C NMR ($CDCl_3$, 100 MHz).

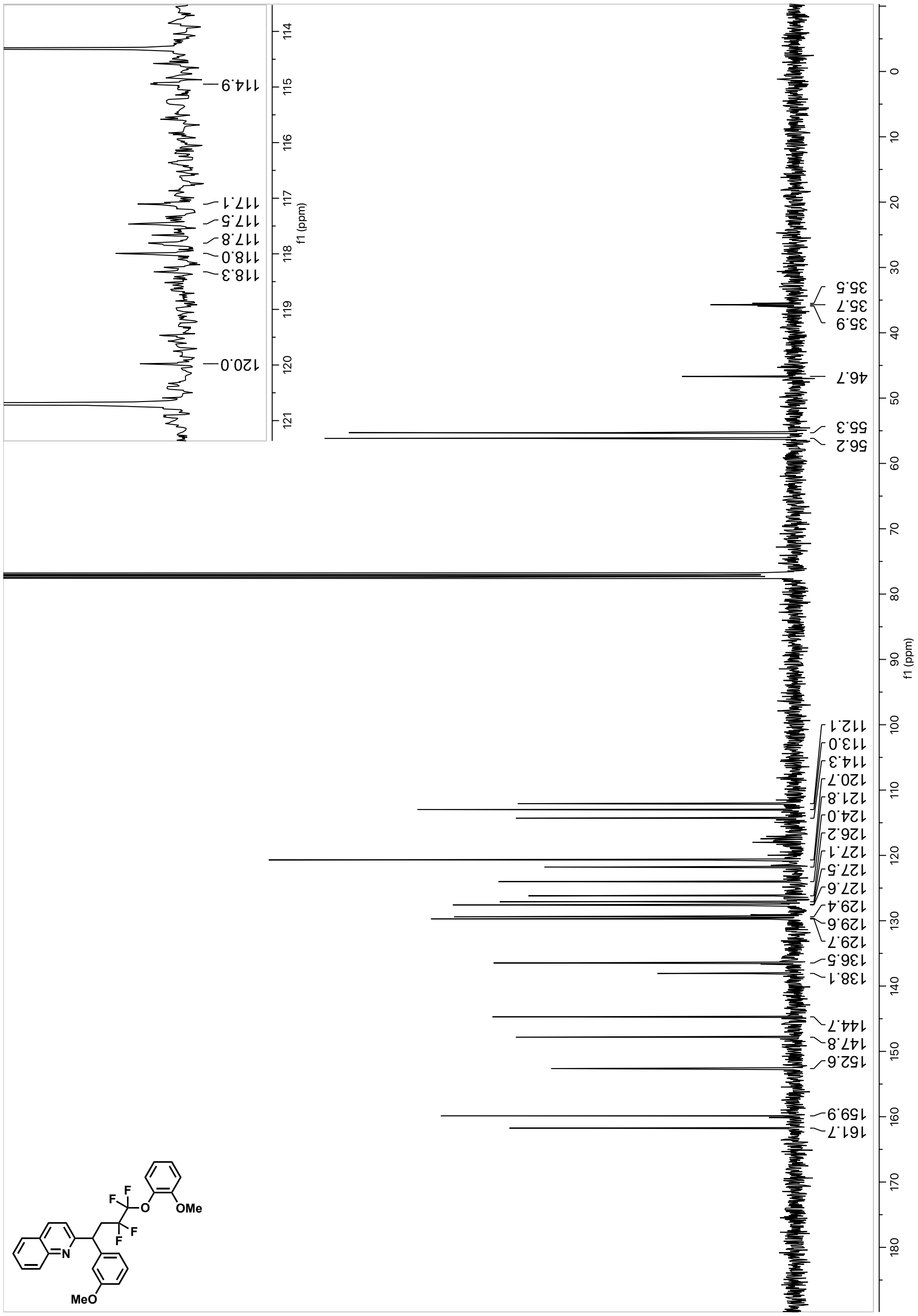

Compound 5a. ^{19}F NMR ($CDCl_3$, 376 MHz).

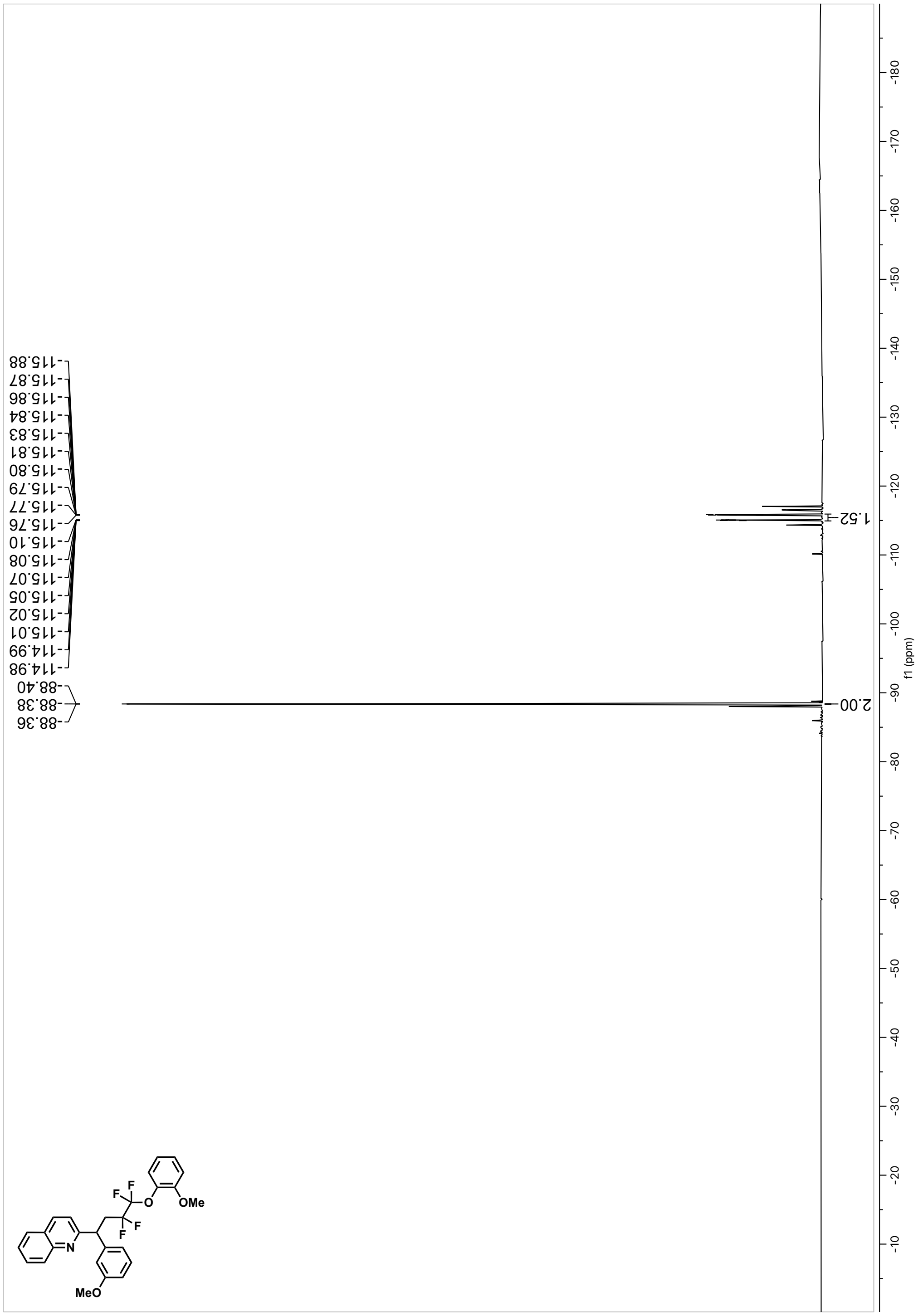

Compound 5b. ^{1}H NMR ($CDCl_3$, 400 MHz).

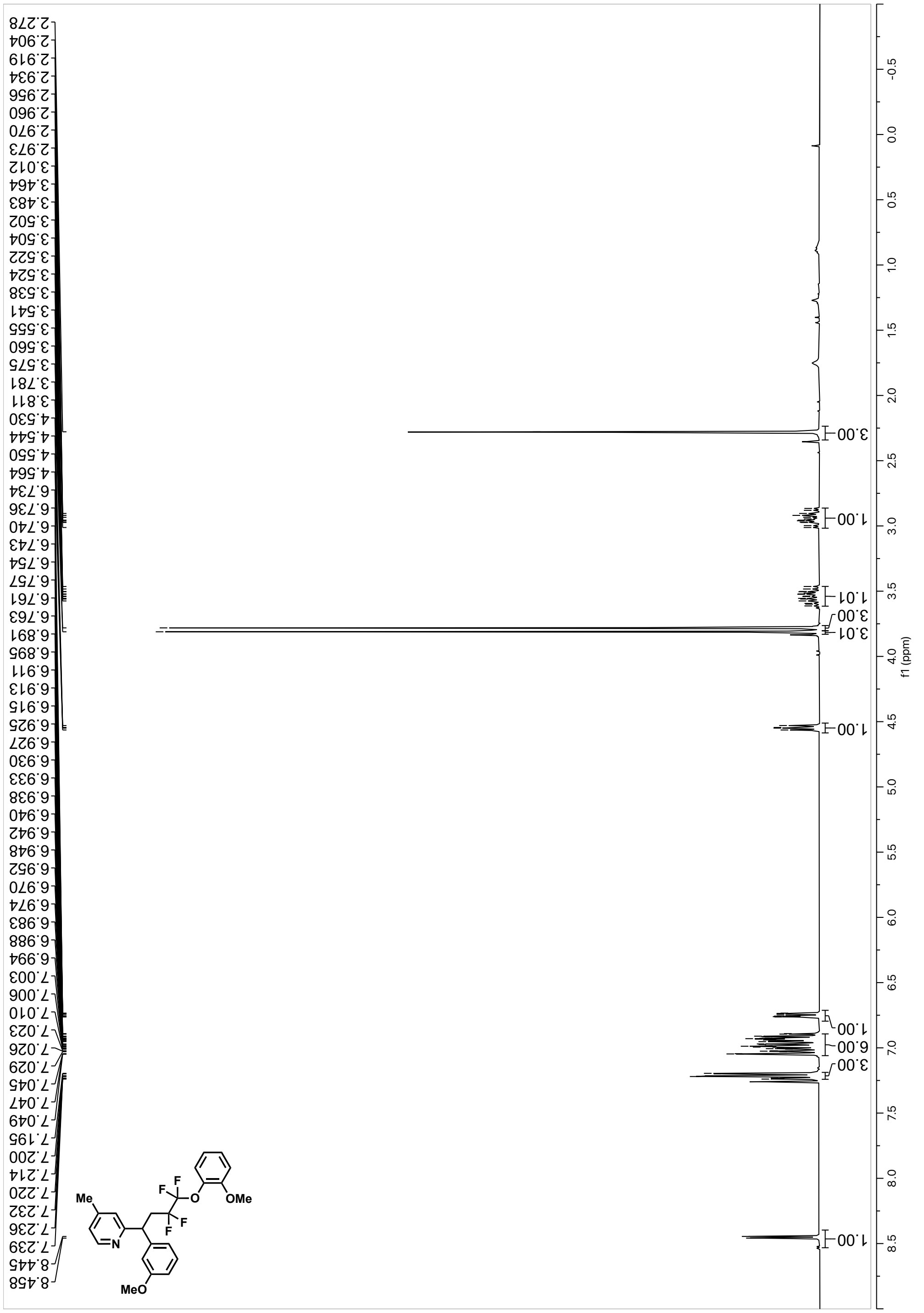

Compound 5b. ^{13}C NMR ($CDCl_3$, 100 MHz).

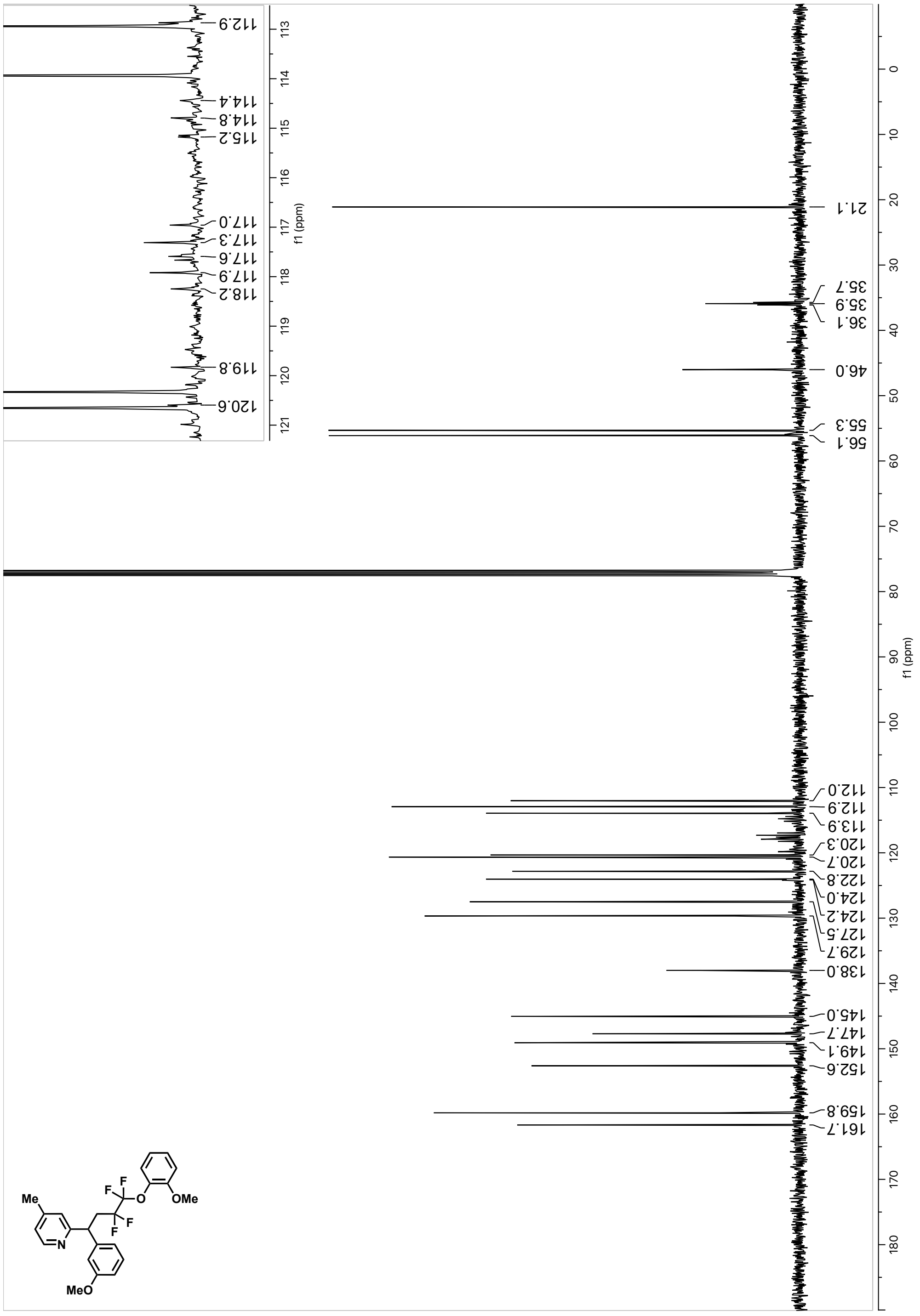

Compound 5b. ^{19}F NMR ($CDCl_3$, 376 MHz).

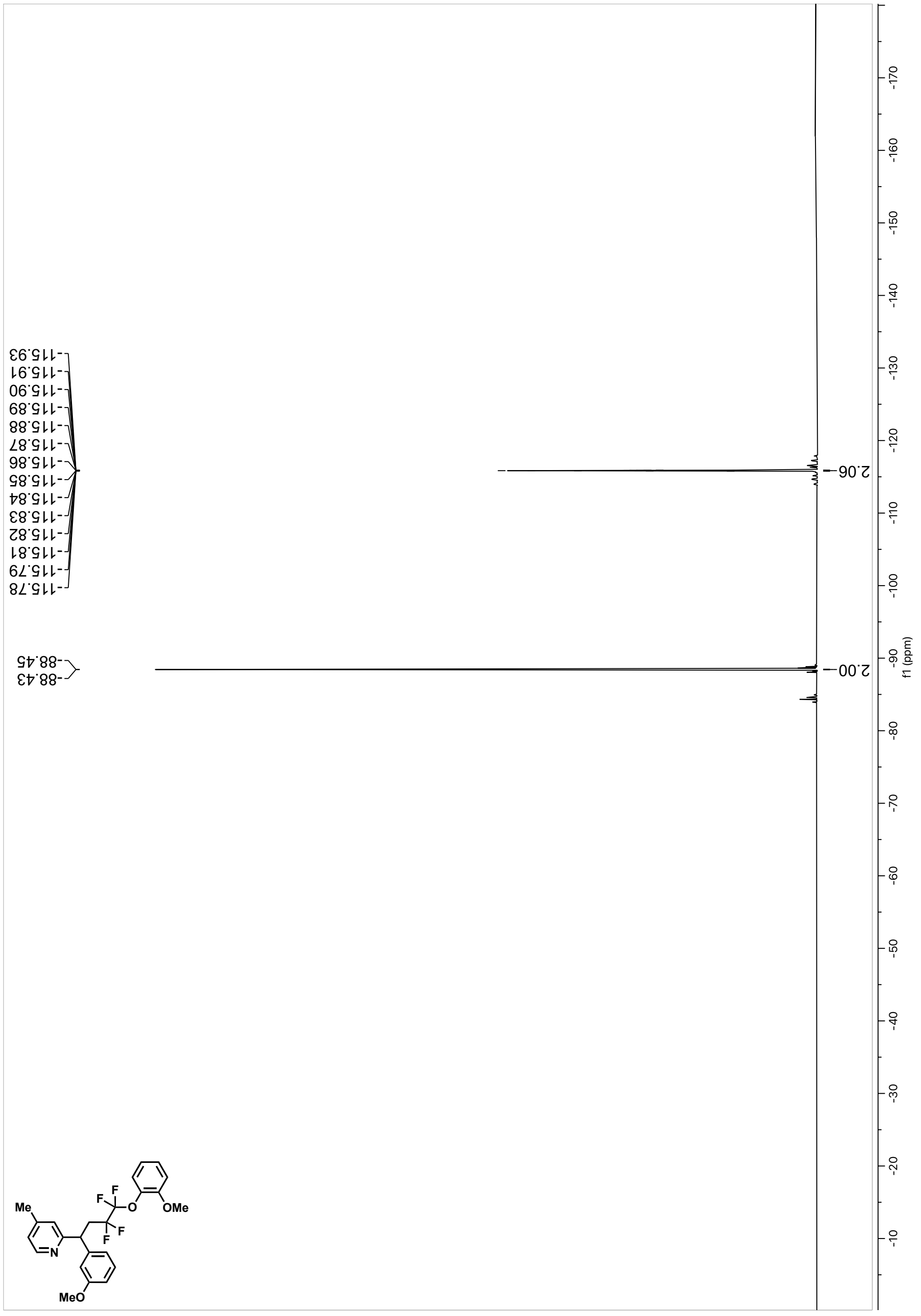

Compound 5c. ^{1}H NMR ($CDCl_3$, 400 MHz).

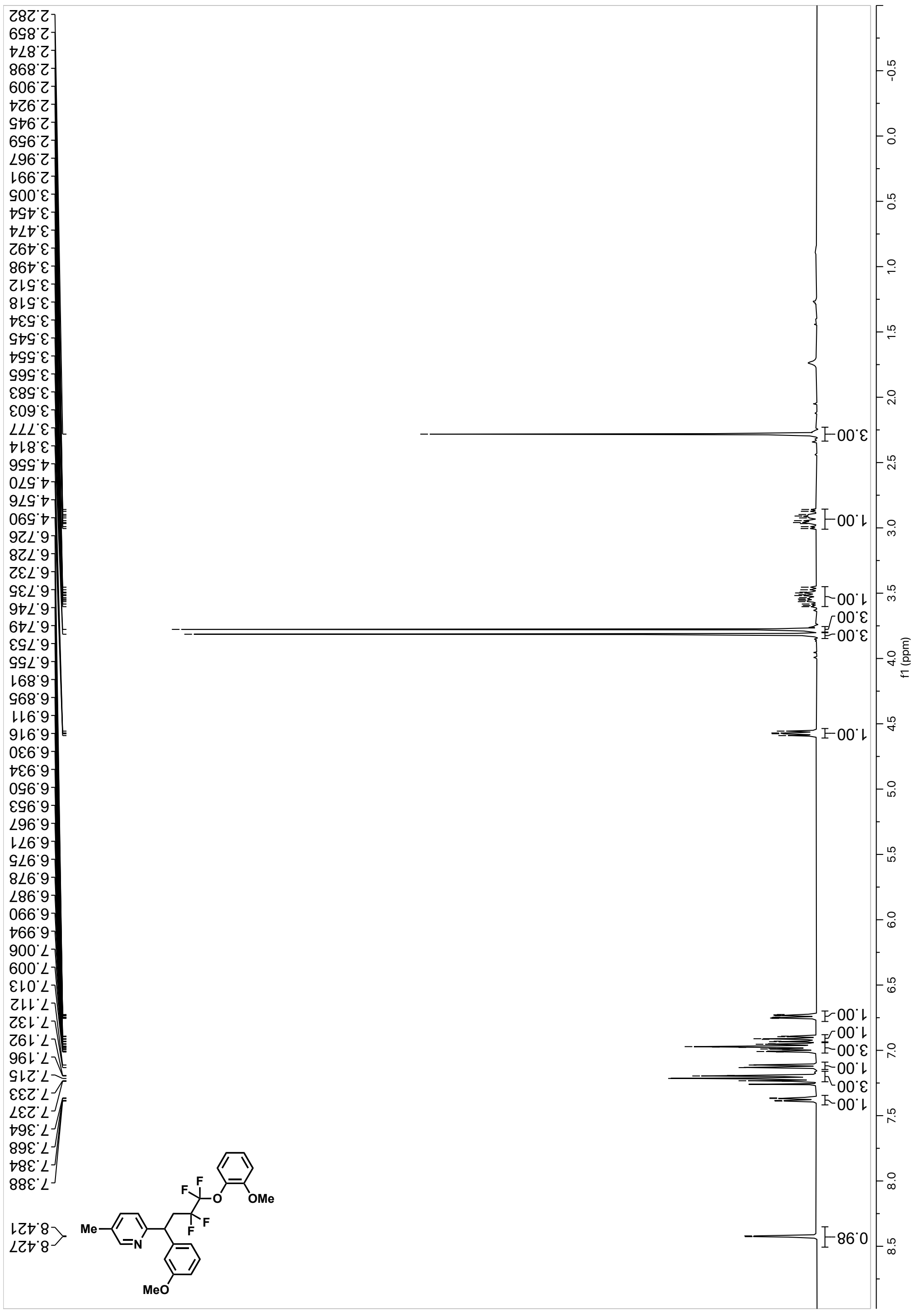

Compound 5c. ^{13}C NMR ($CDCl_3$, 100 MHz).

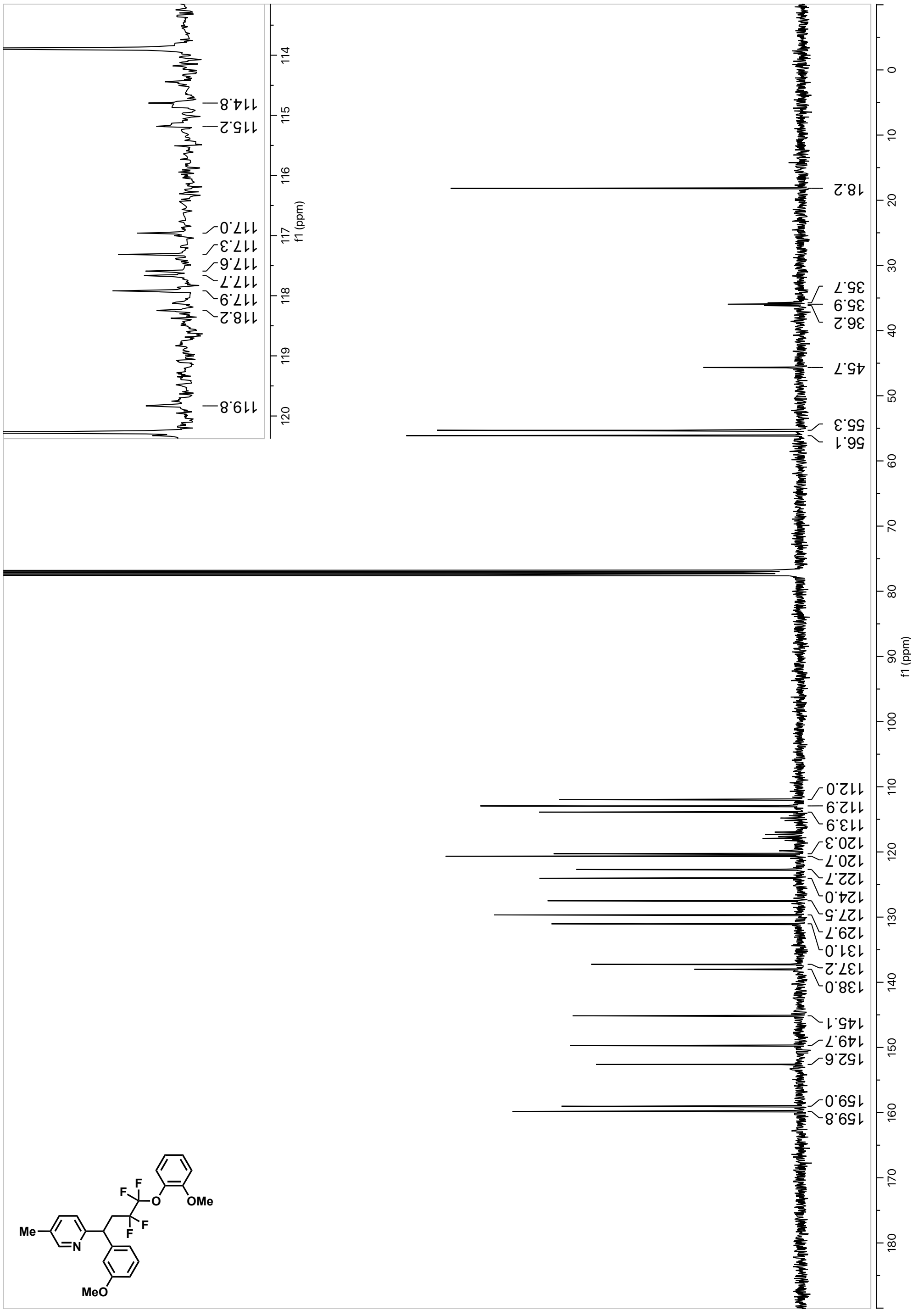

Compound 5c. ^{19}F NMR ($CDCl_3$, 376 MHz).

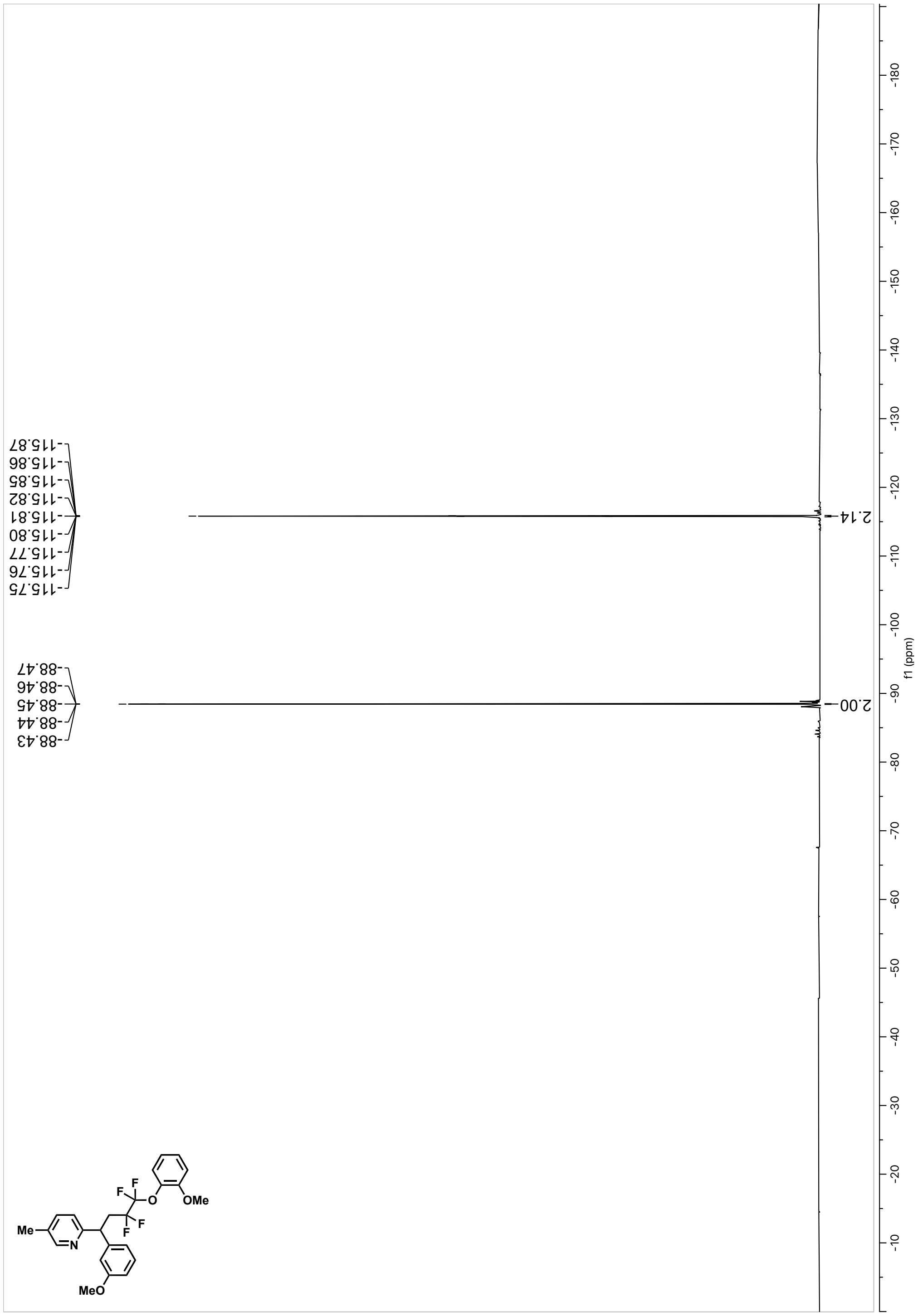

Compound 5d. ^{1}H NMR ($CDCl_3$, 400 MHz).

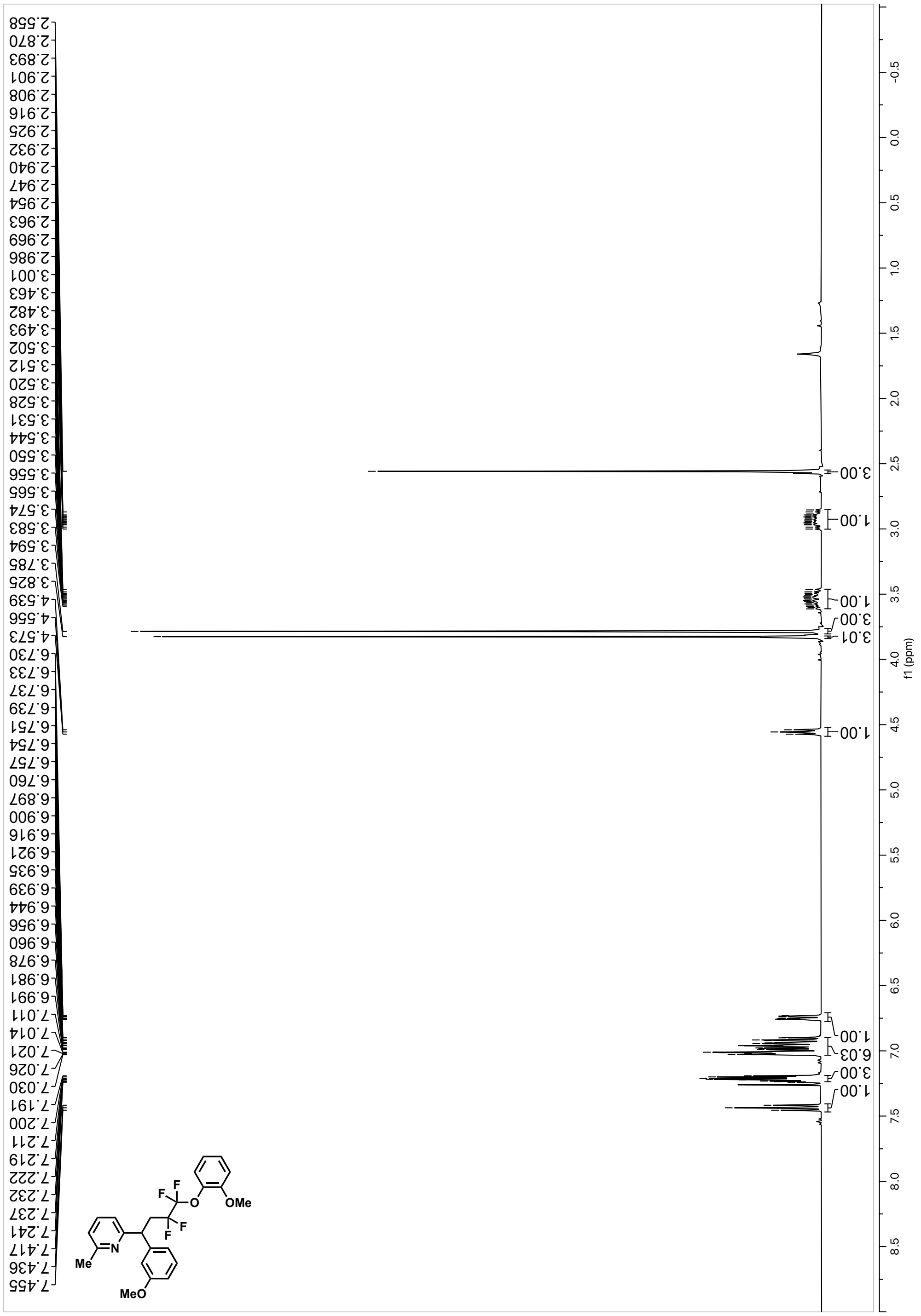

Compound 5d. ^{13}C NMR ($CDCl_3$, 100 MHz).

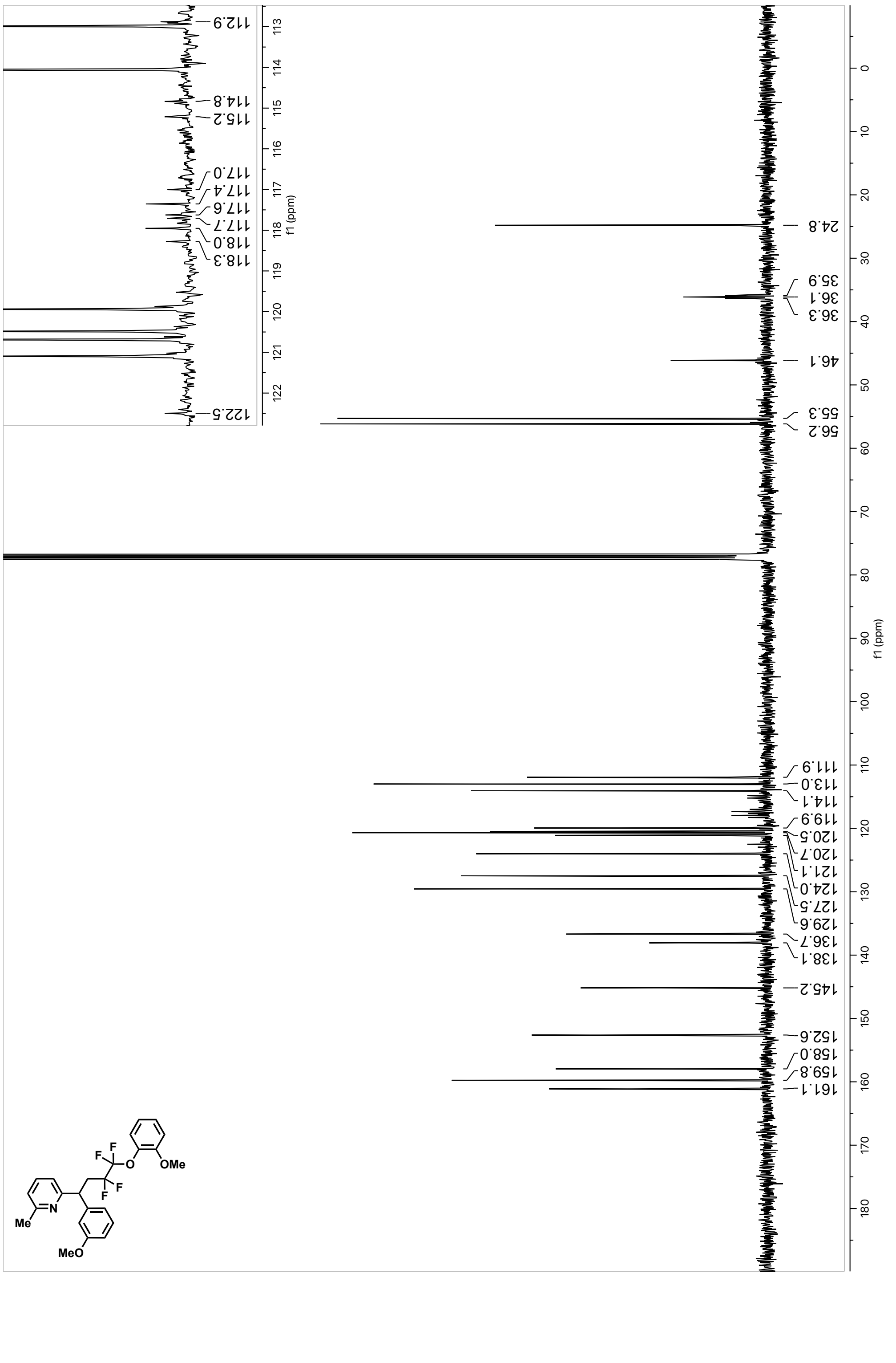

Compound 5d. ^{19}F NMR ($CDCl_3$, 376 MHz).

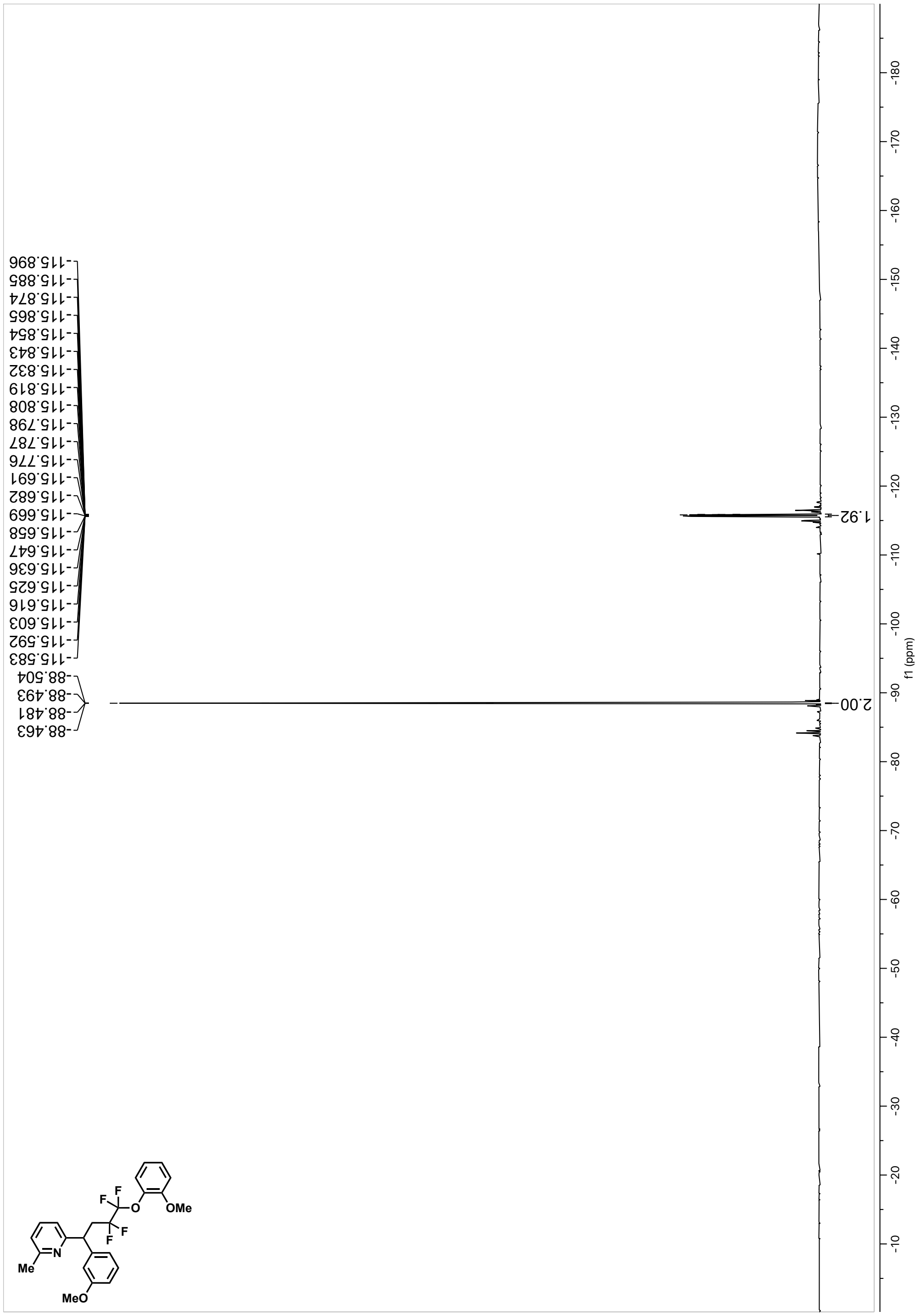

Compound 5e. ^{1}H NMR ($CDCl_3$, 400 MHz). Bottom: ^{13}C NMR ($CDCl_3$, 100 MHz).

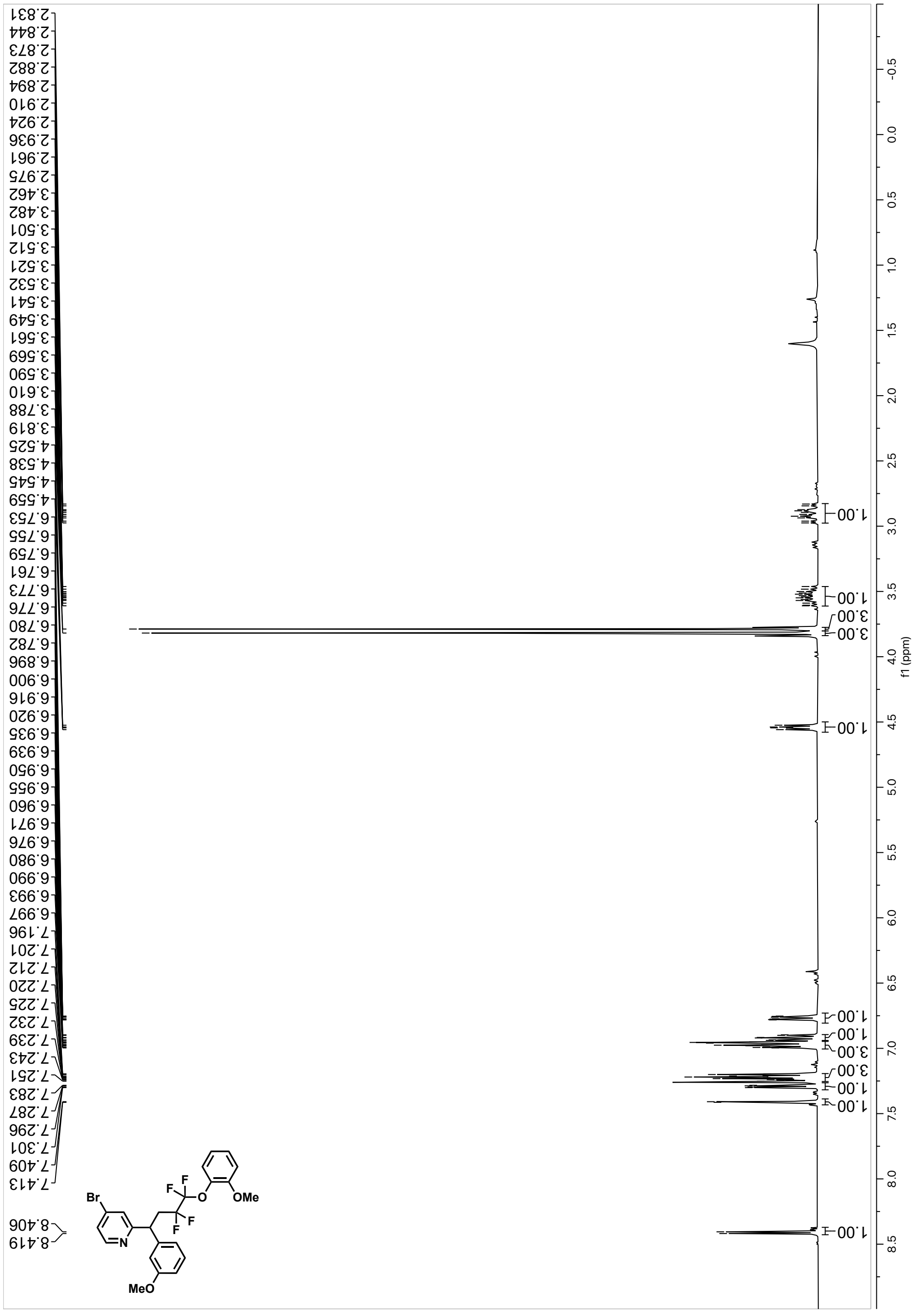

Compound 5e. ^{13}C NMR ($CDCl_3$, 100 MHz).

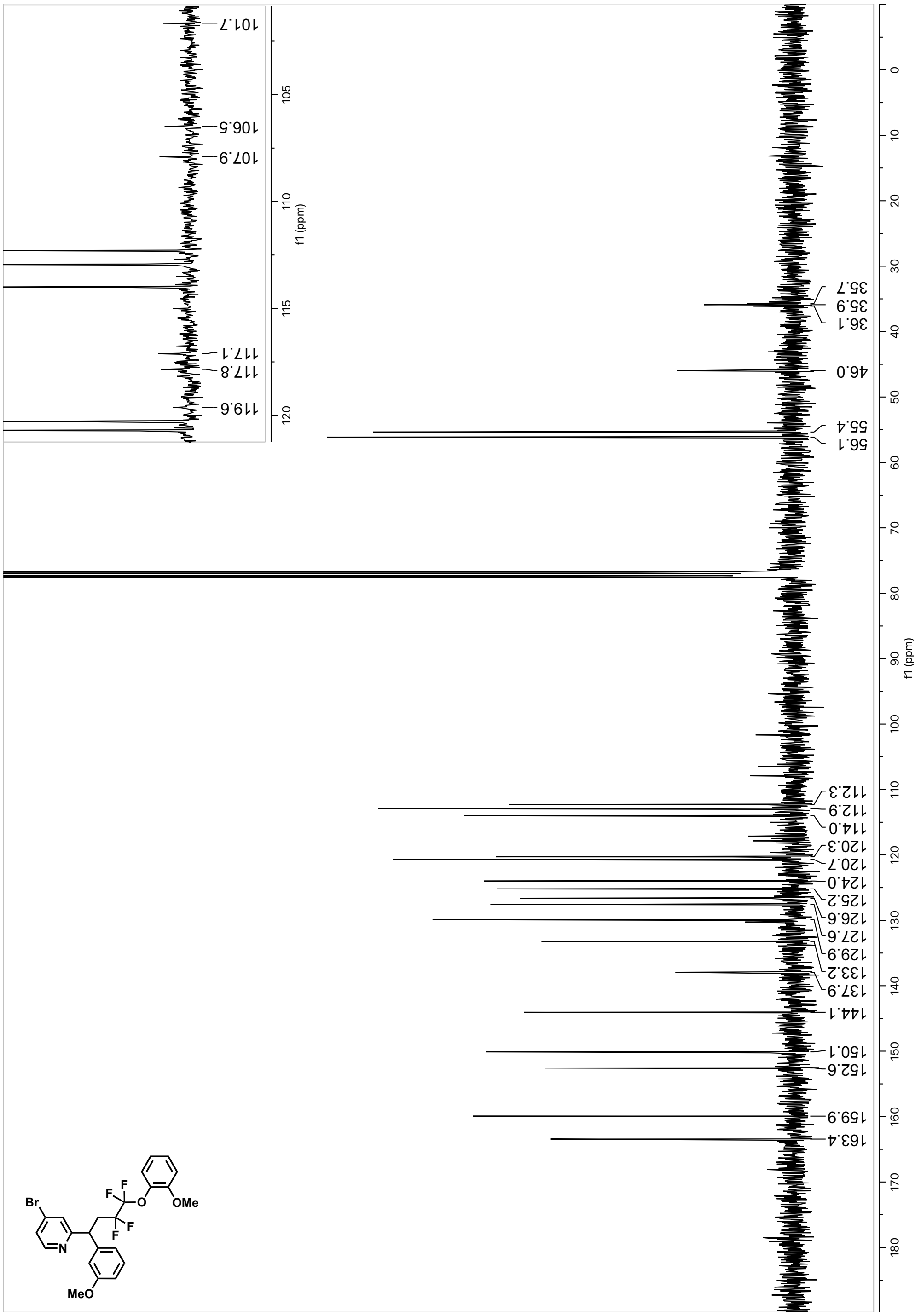

Compound 5e. ^{19}F NMR ($CDCl_3$, 376 MHz).

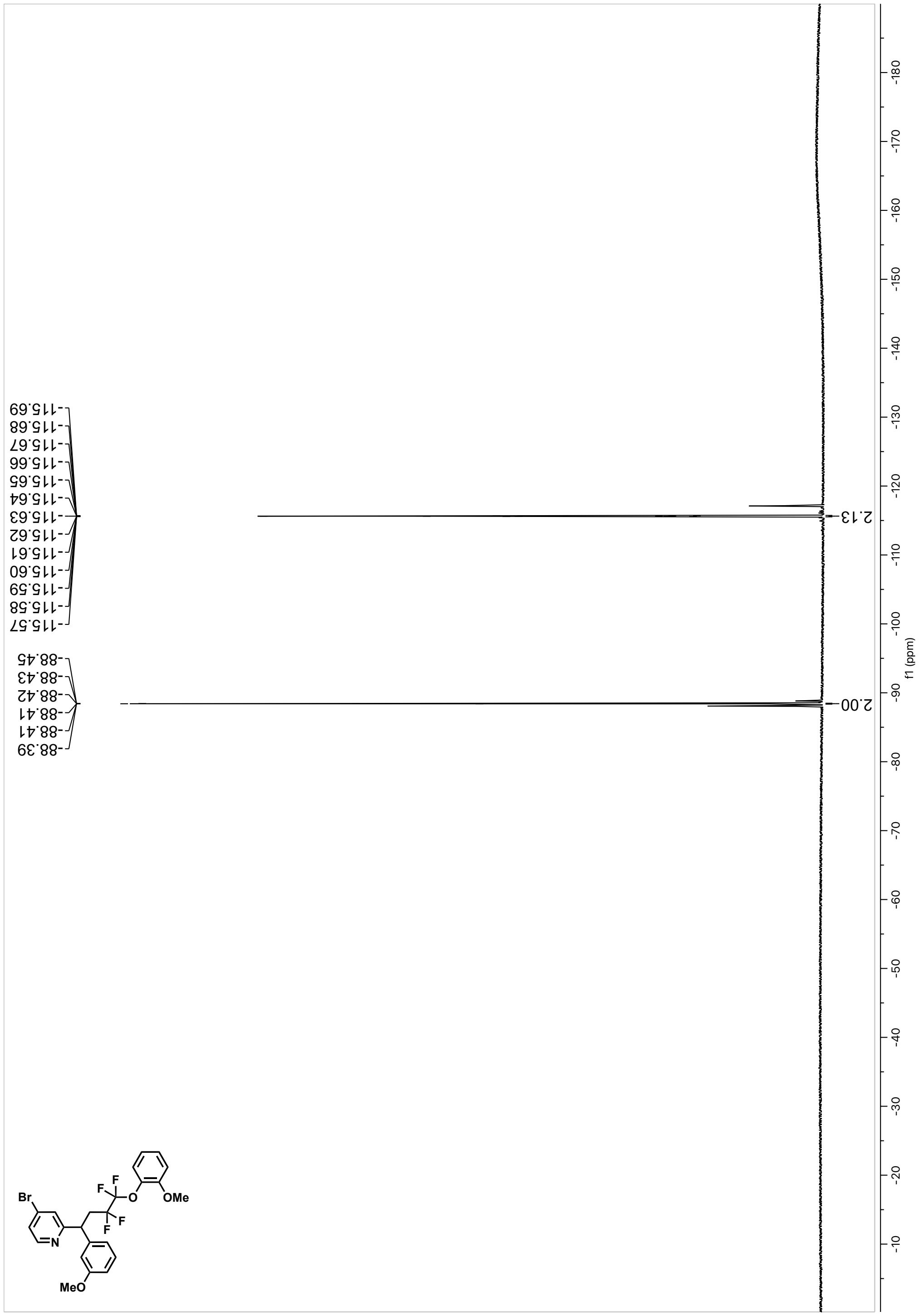

Compound 5f. ^{1}H NMR ($CDCl_3$, 400 MHz).

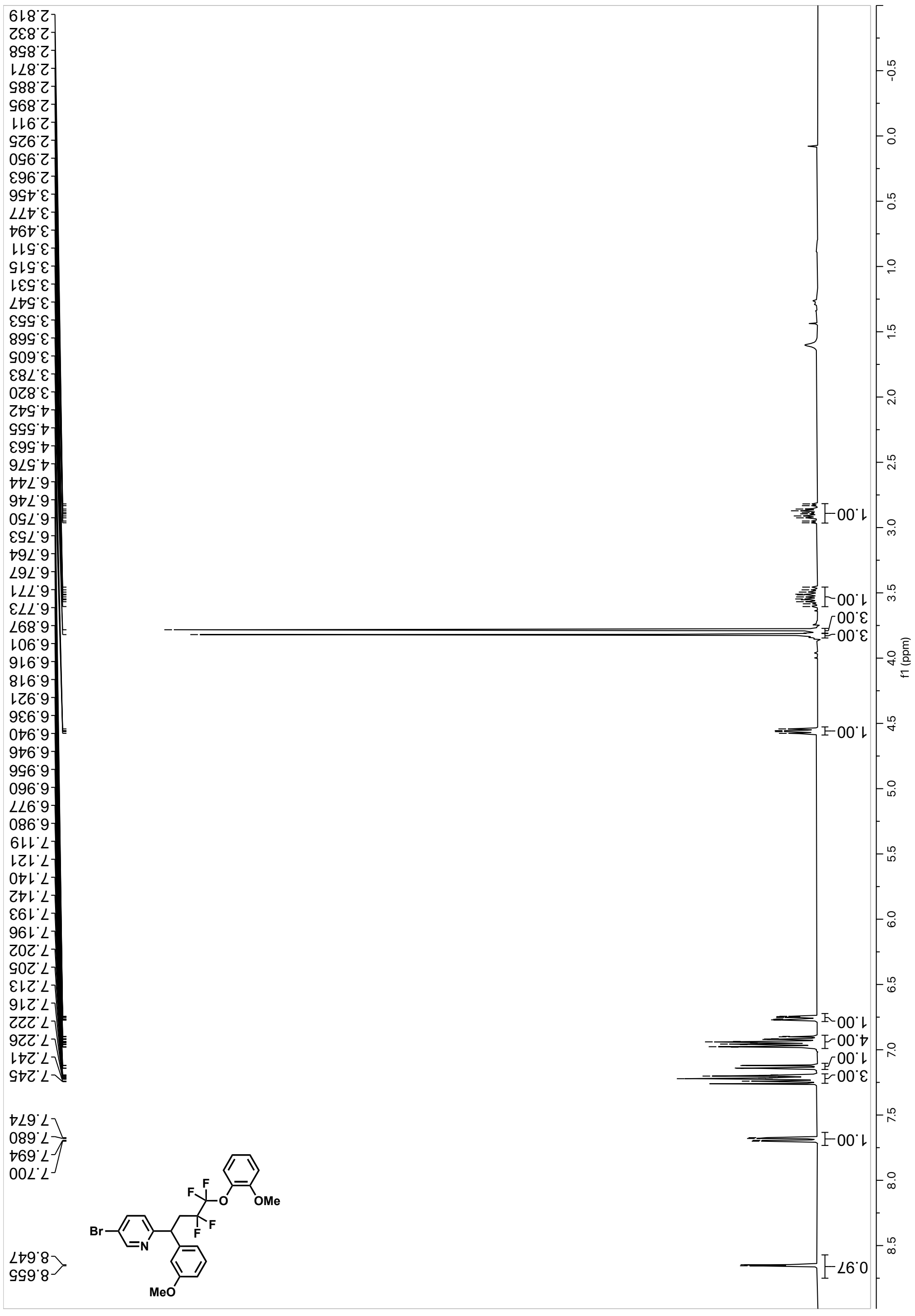

Compound 5f. ^{13}C NMR ($CDCl_3$, 100 MHz).

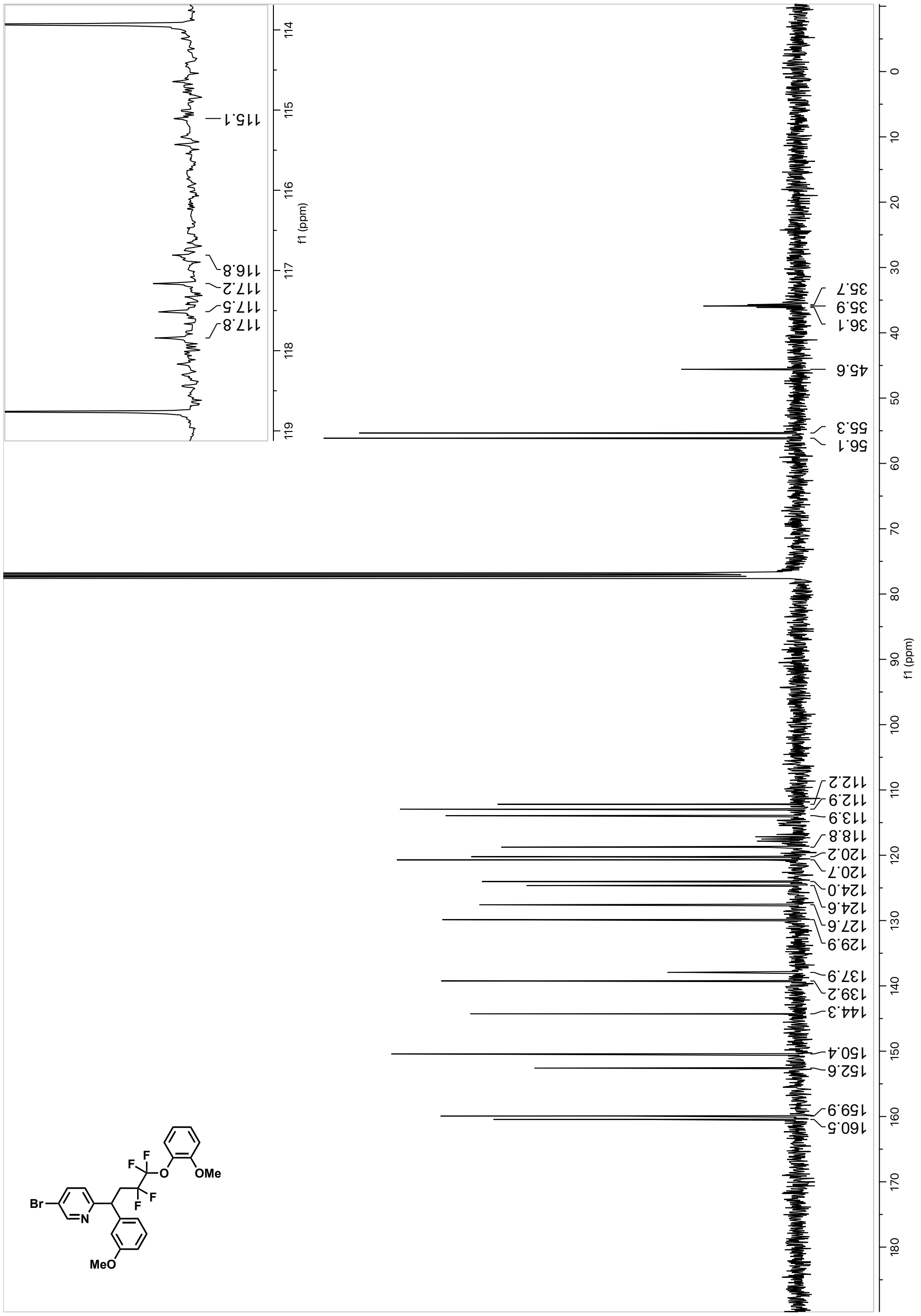

Compound 5f. ^{19}F NMR ($CDCl_3$, 376 MHz).

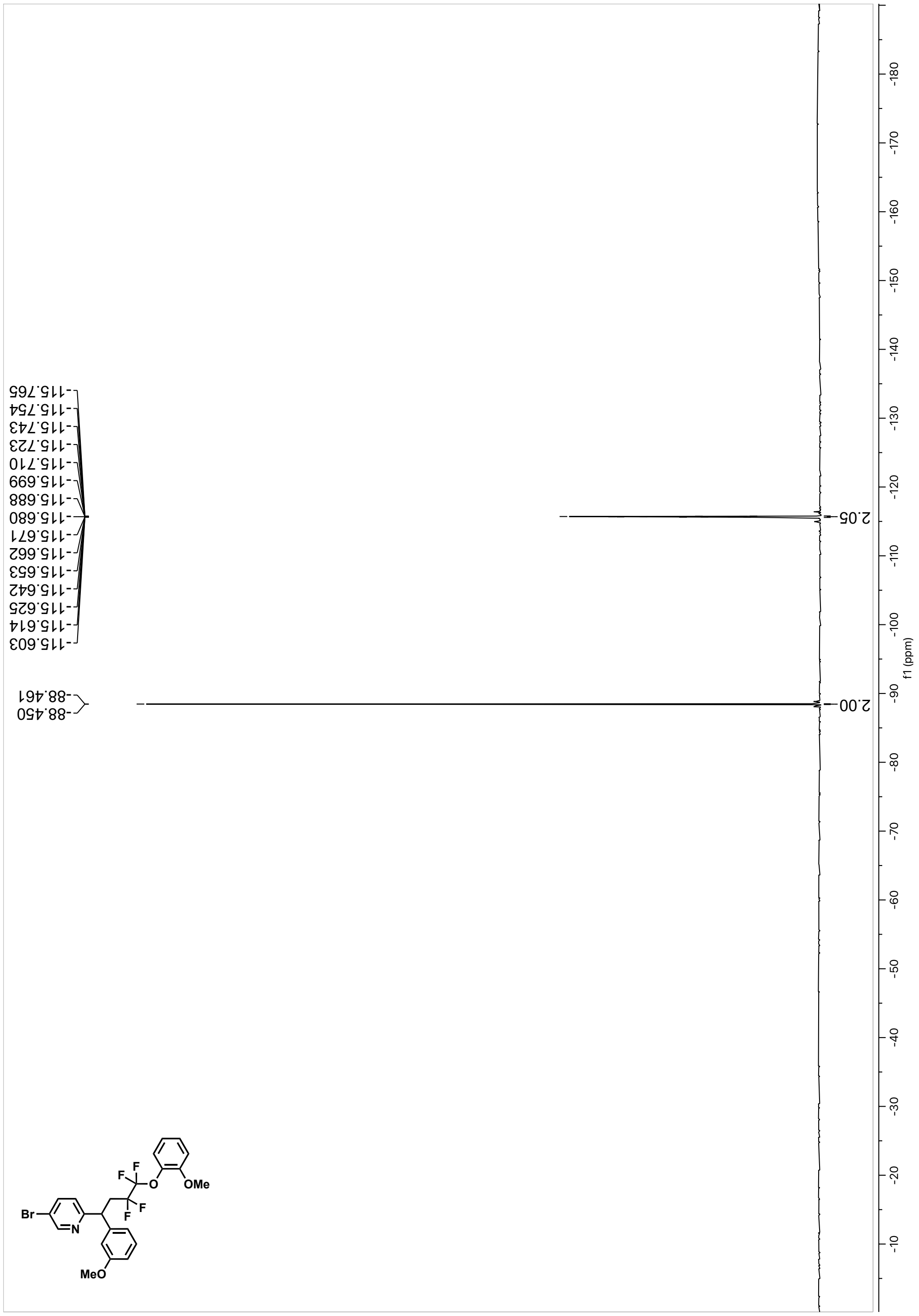

Compound 5g. ^{1}H NMR ($CDCl_3$, 400 MHz).

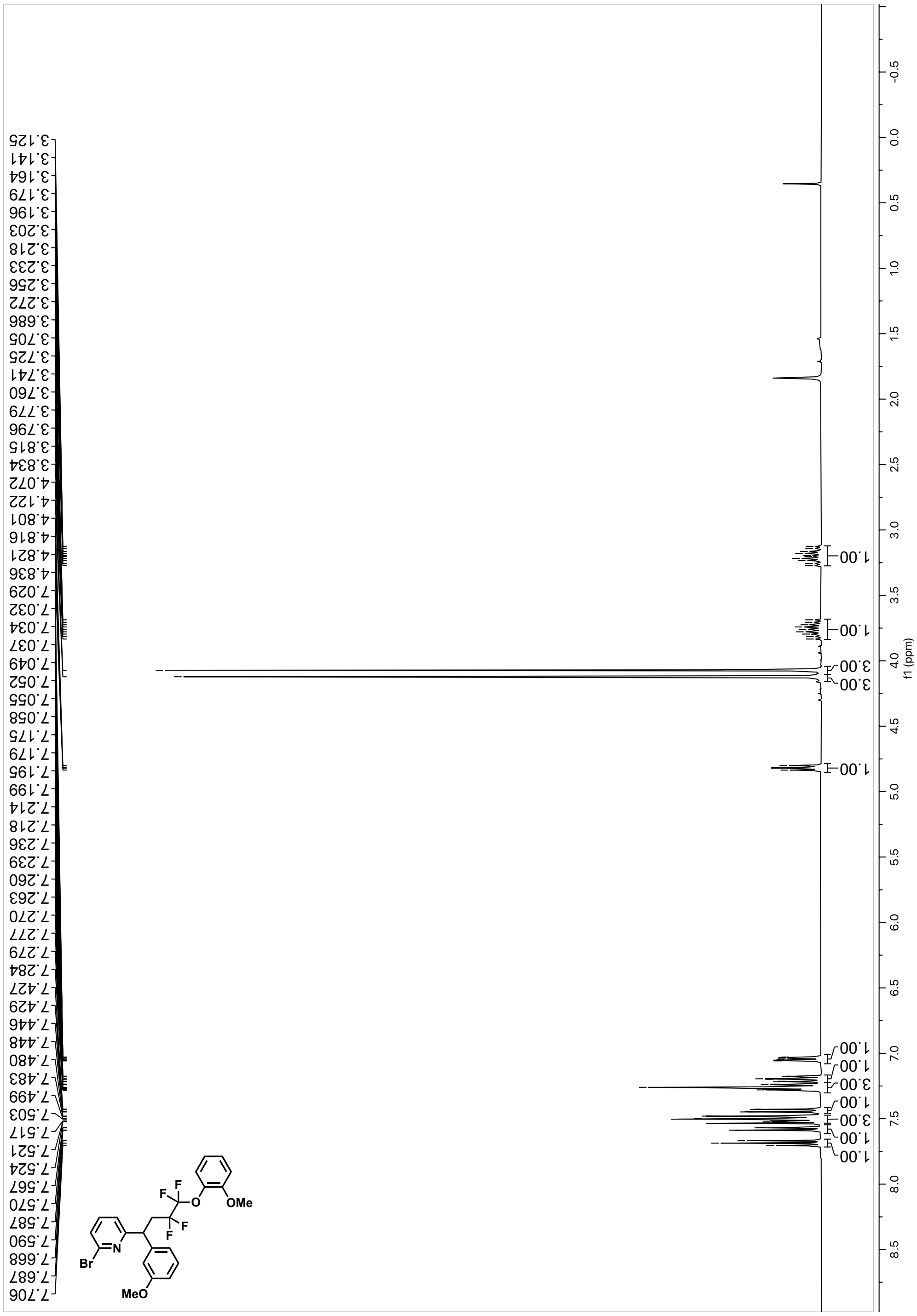

Compound 5g. ^{13}C NMR ($CDCl_3$, 100 MHz).

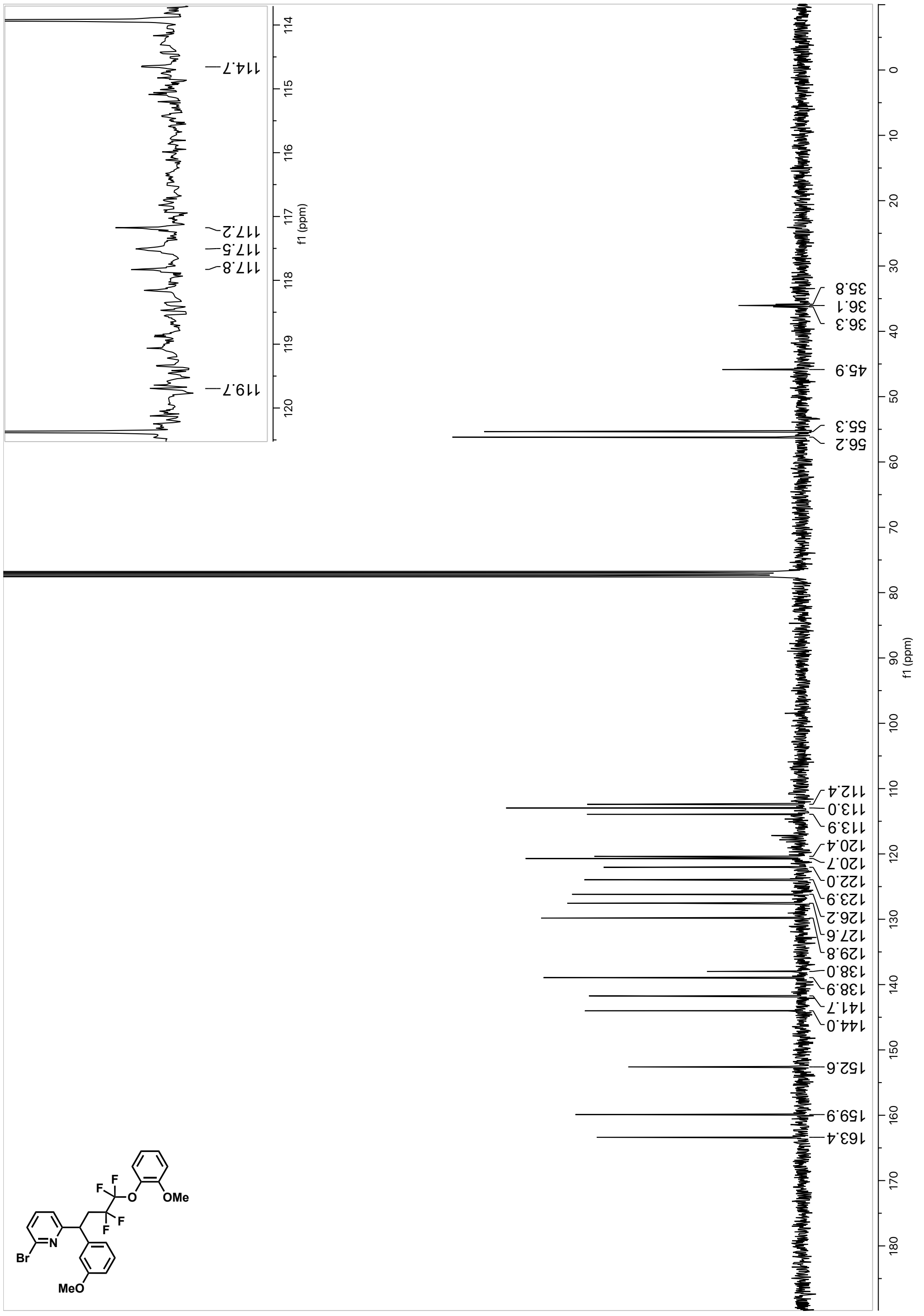

Compound 5g. ^{19}F NMR (CDCl$_3$, 376 MHz).

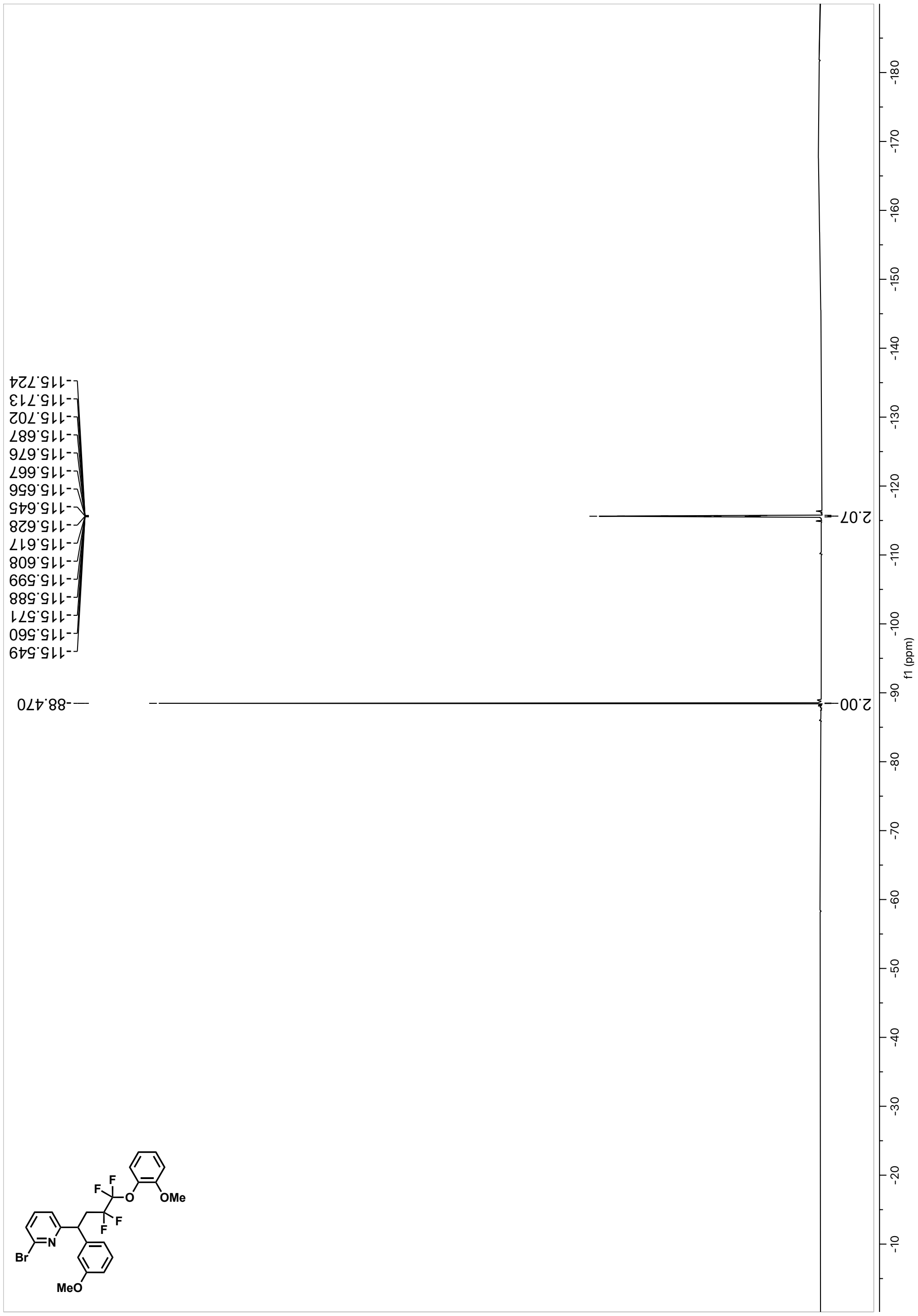

Compound 5h. ^{1}H NMR ($CDCl_3$, 400 MHz).

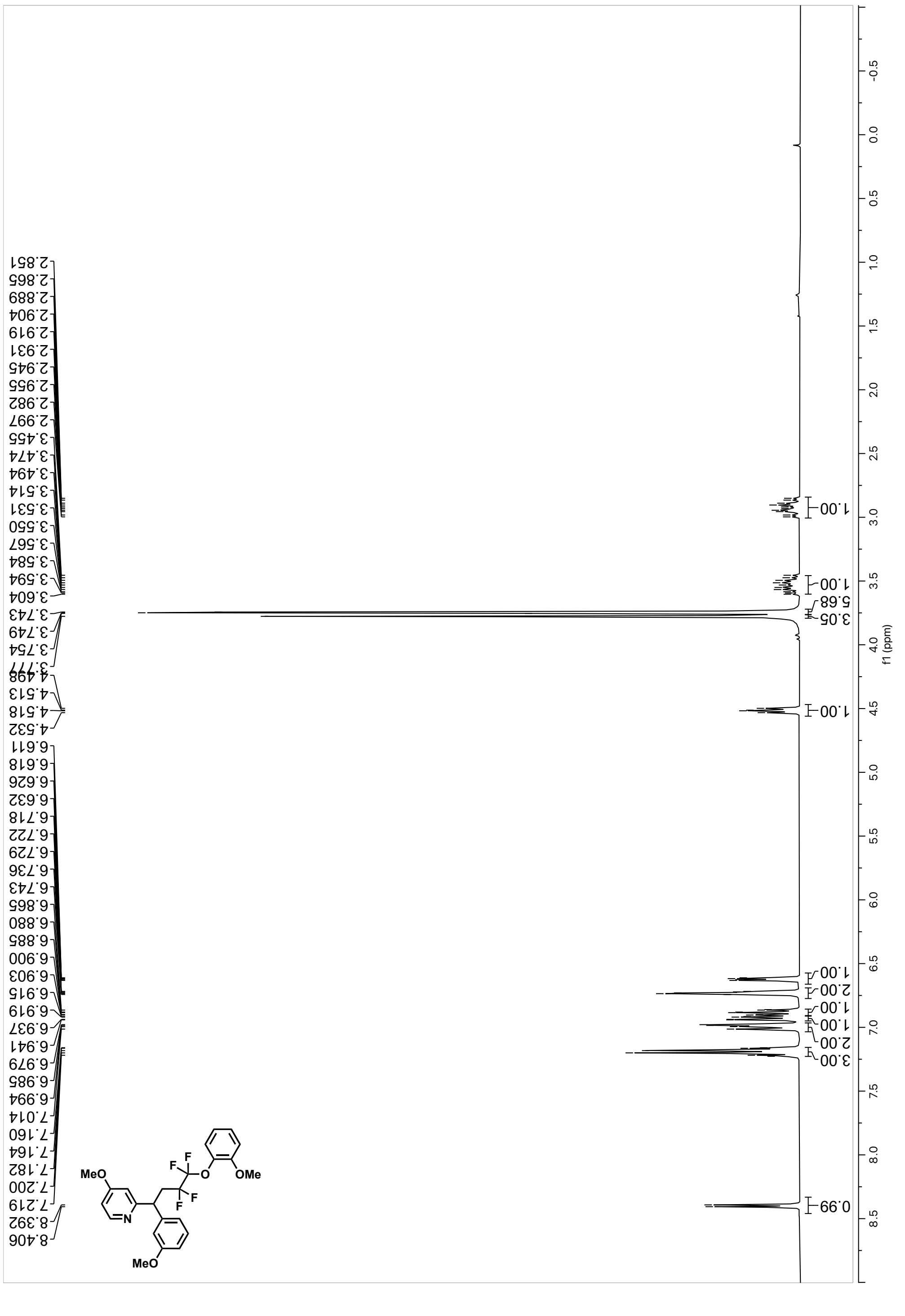

Compound 5h. ^{13}C NMR ($CDCl_3$, 100 MHz).

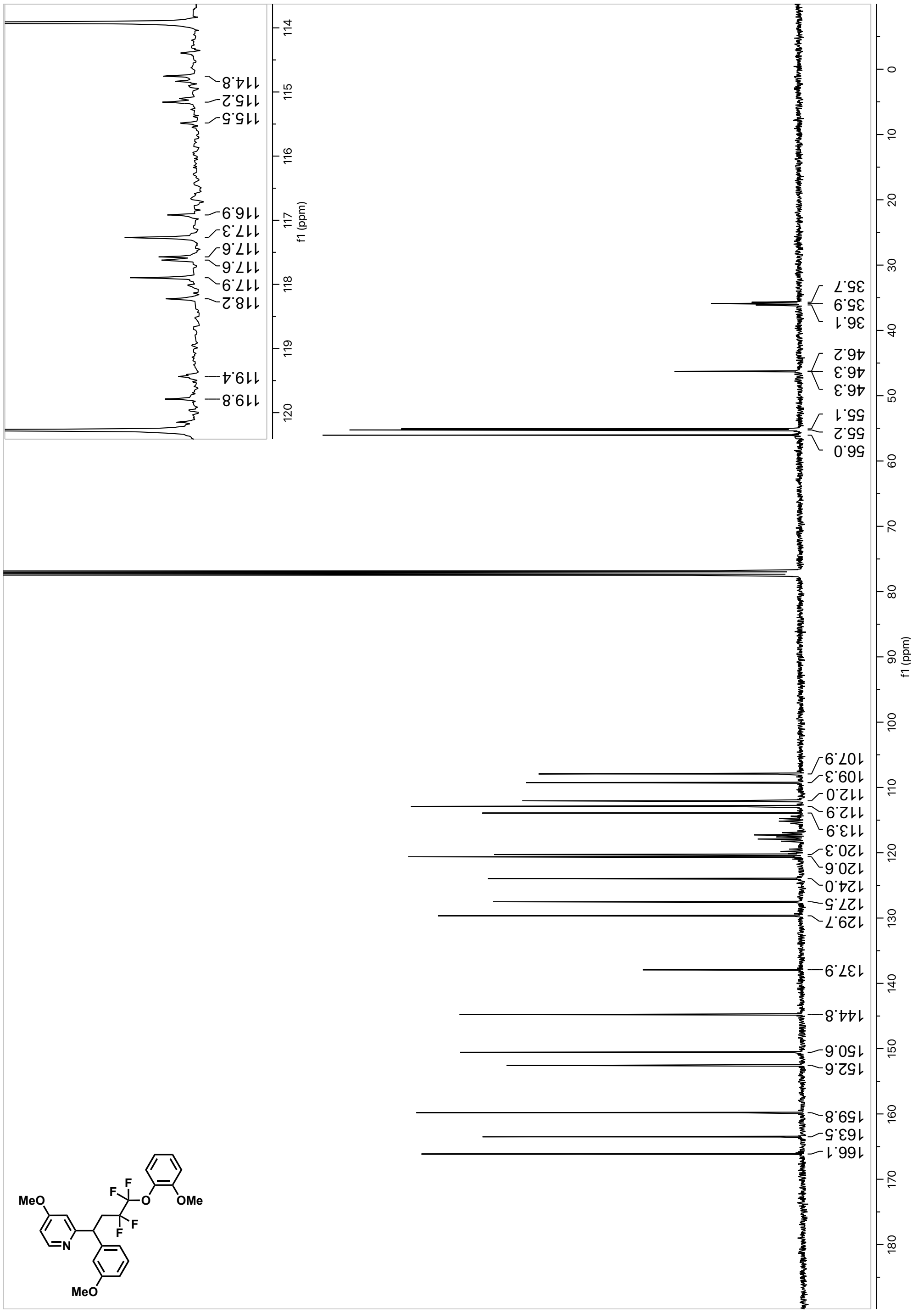

Compound 5h. ^{19}F NMR ($CDCl_3$, 376 MHz).

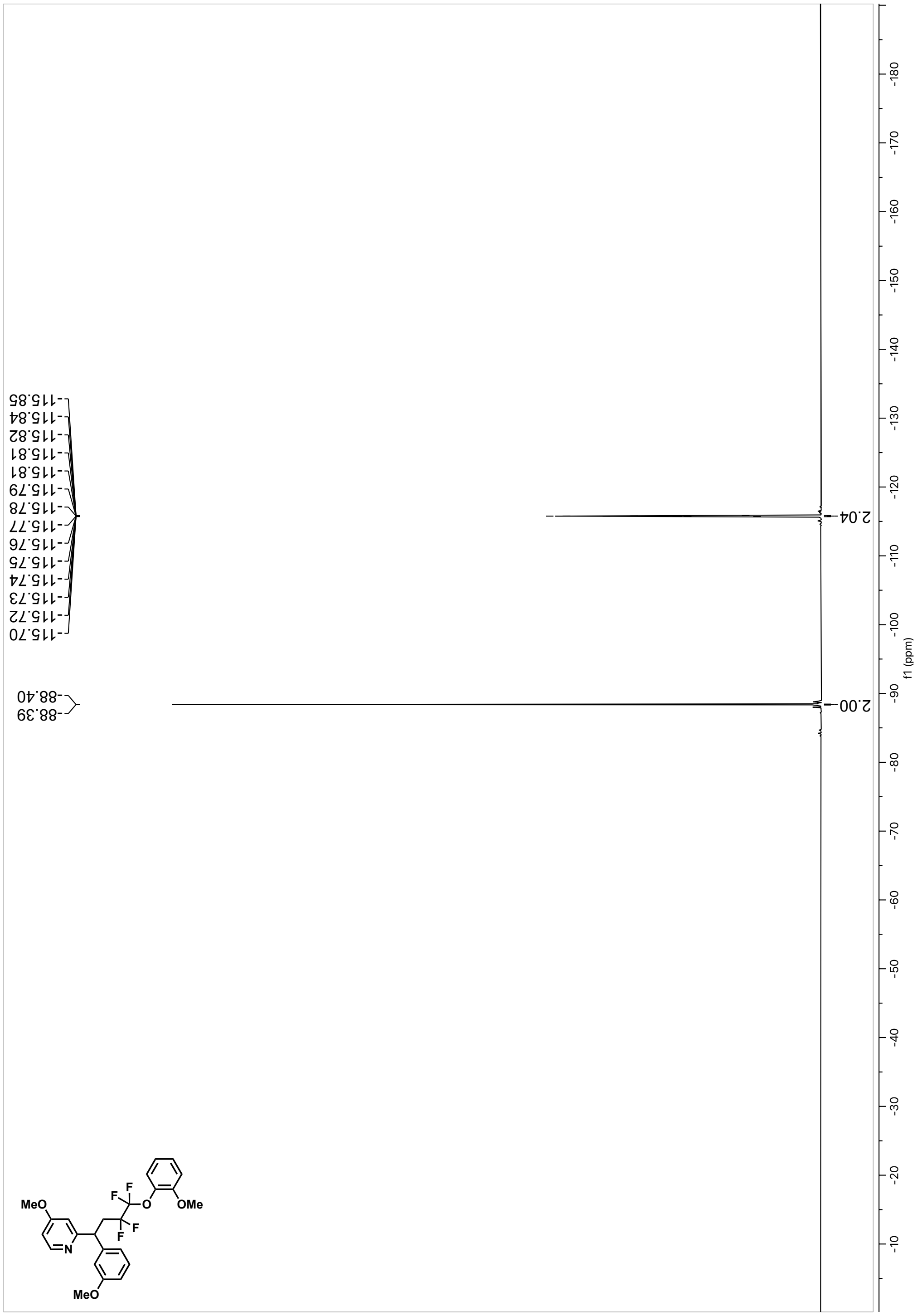

Compound 5i. ^{1}H NMR ($CDCl_3$, 400 MHz).

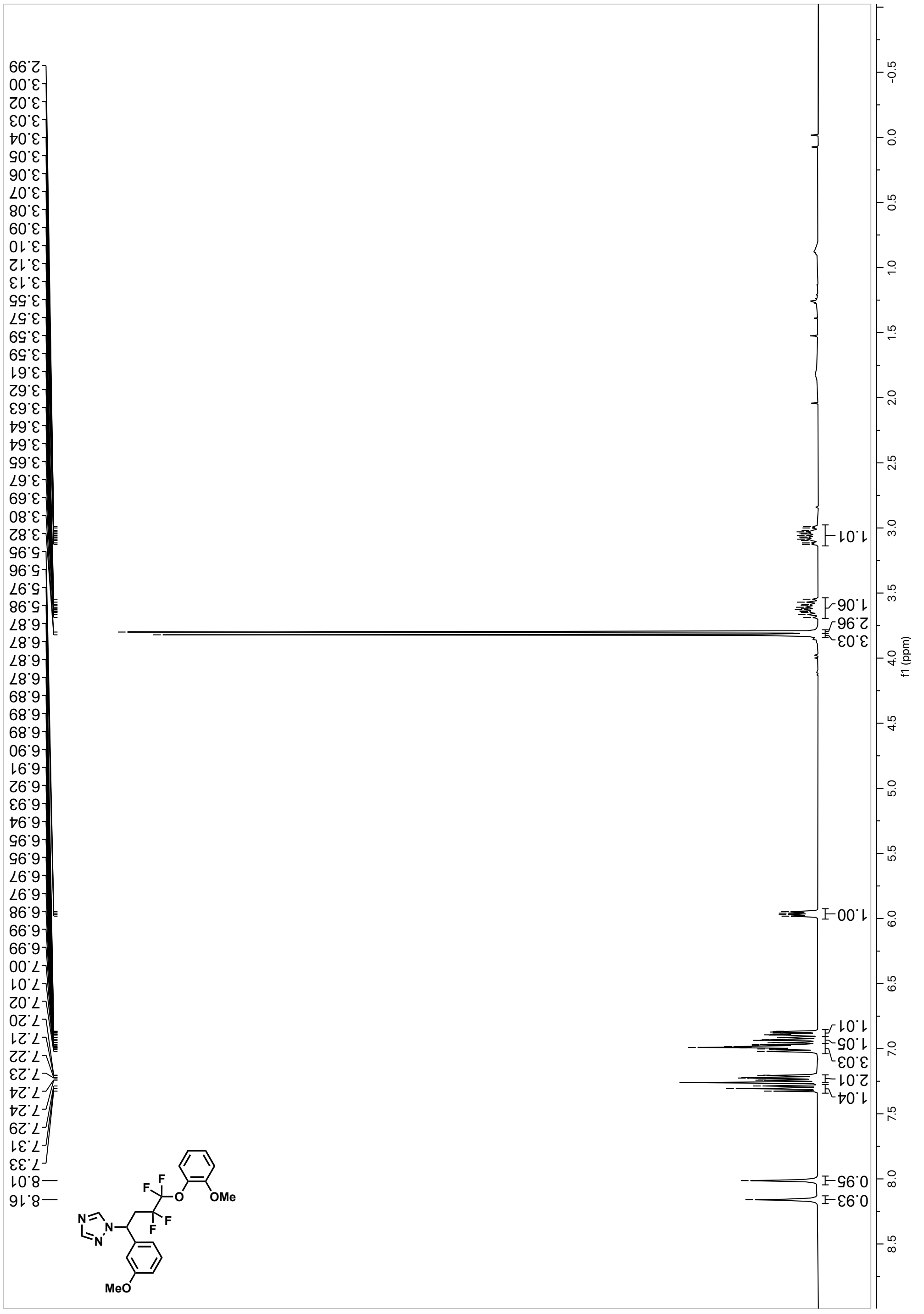

Compound 5i. ^{13}C NMR ($CDCl_3$, 100 MHz).

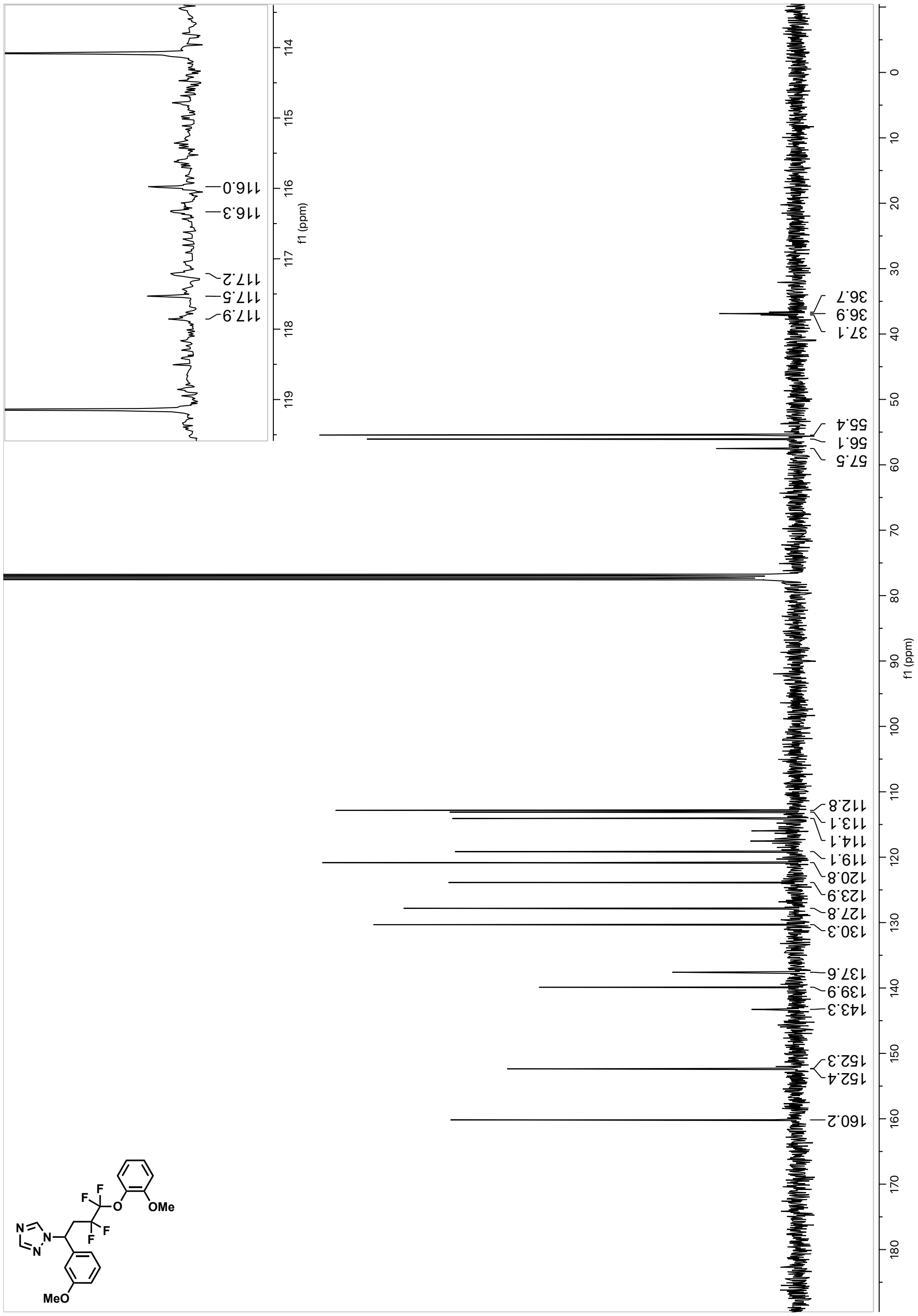

Compound 5i. ^{19}F NMR ($CDCl_3$, 376 MHz).

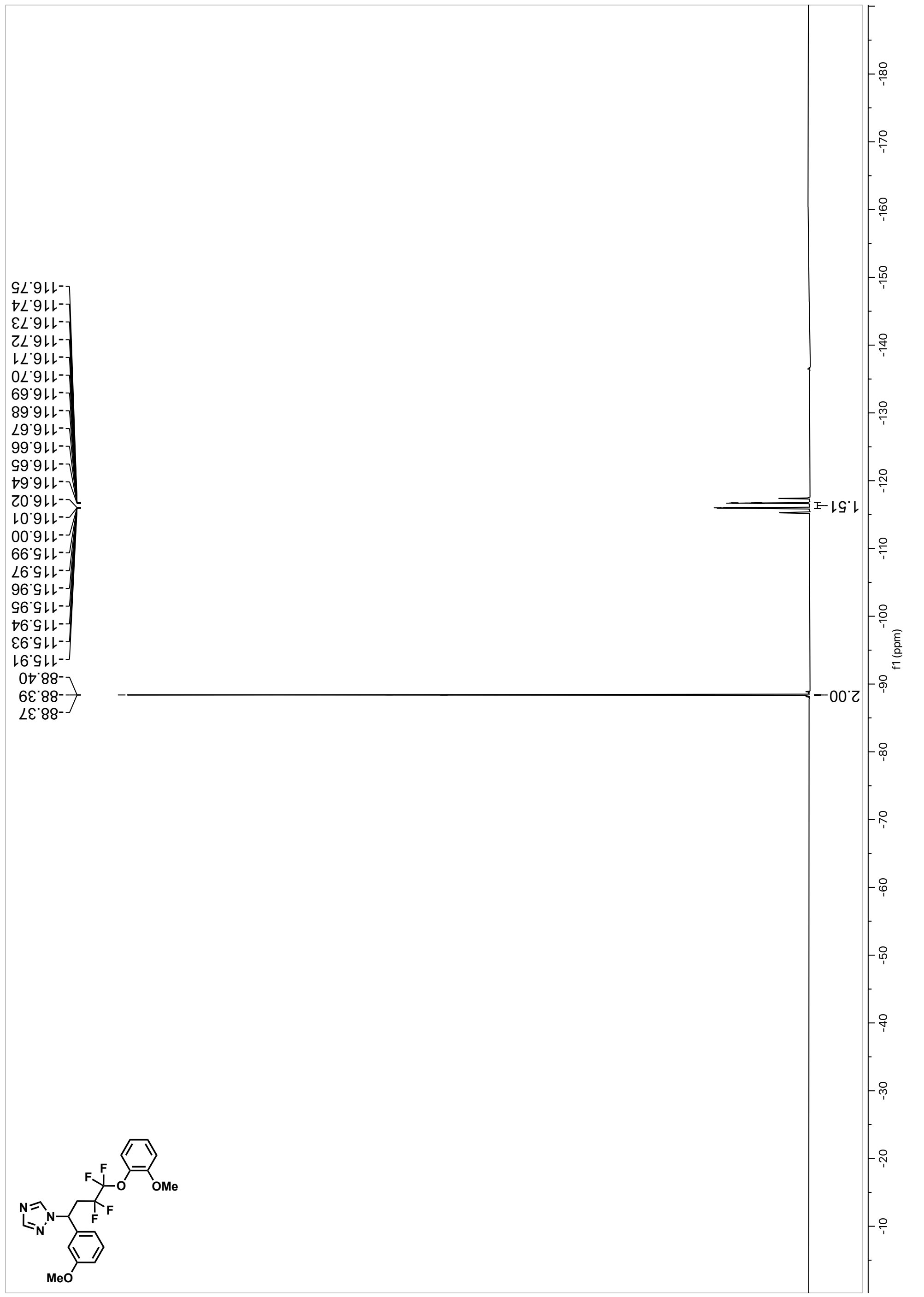

Compound 5j. ^{1}H NMR ($CDCl_3$, 400 MHz).

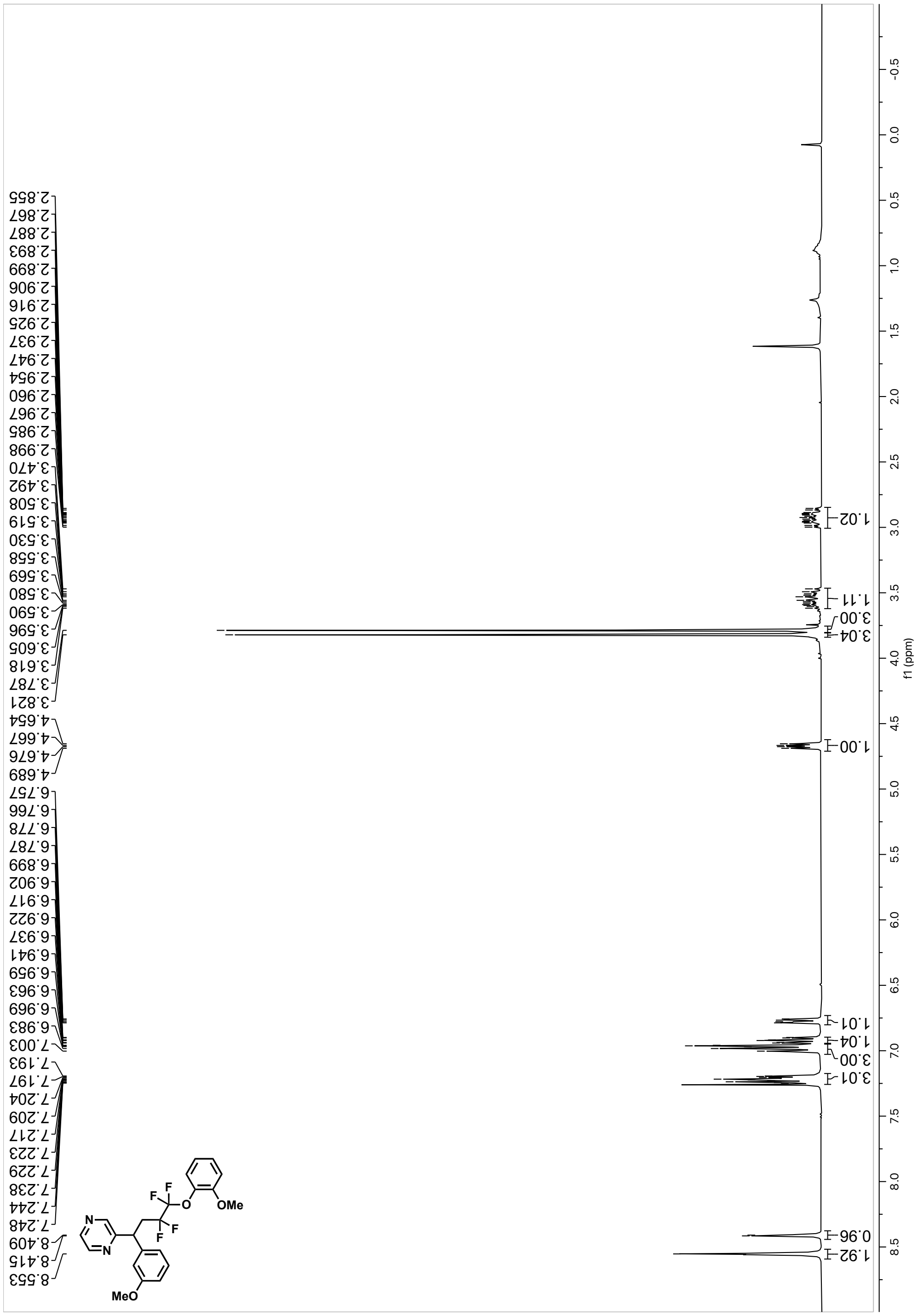

Compound 5j. ^{13}C NMR ($CDCl_3$, 100 MHz).

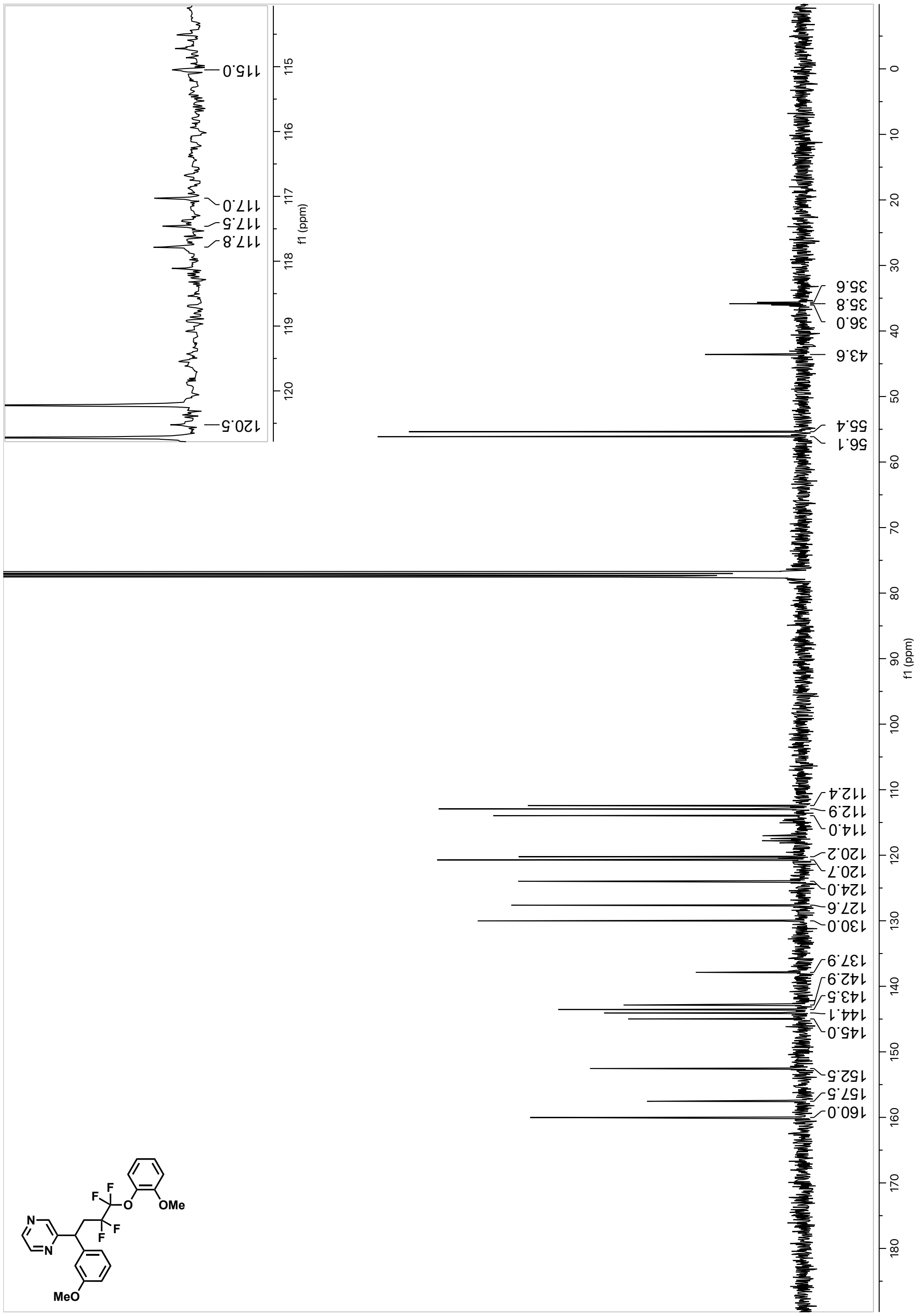

Compound 5j. ^{19}F NMR ($CDCl_3$, 376 MHz).

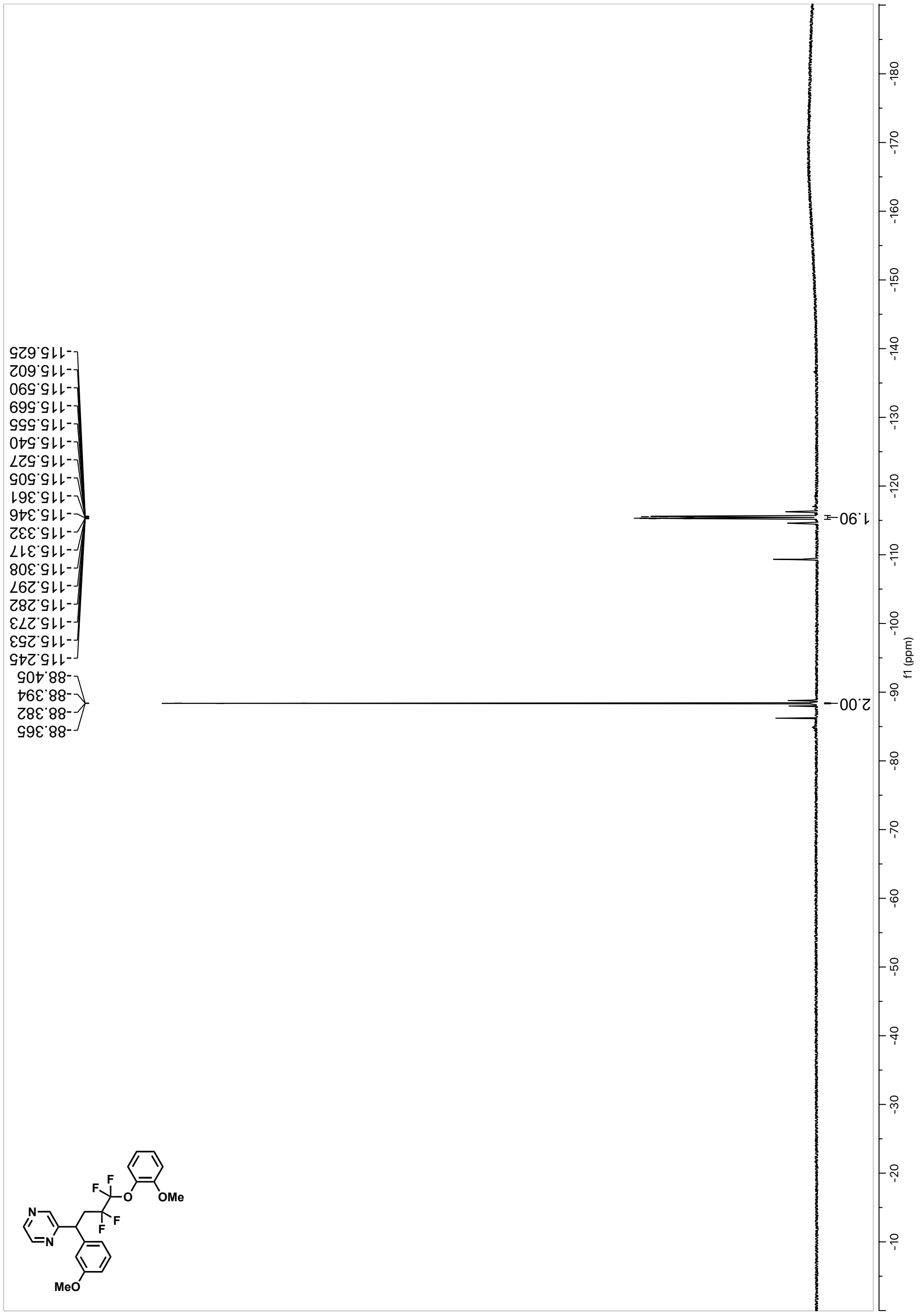

Compound 5l. ^{1}H NMR ($CDCl_3$, 400 MHz).

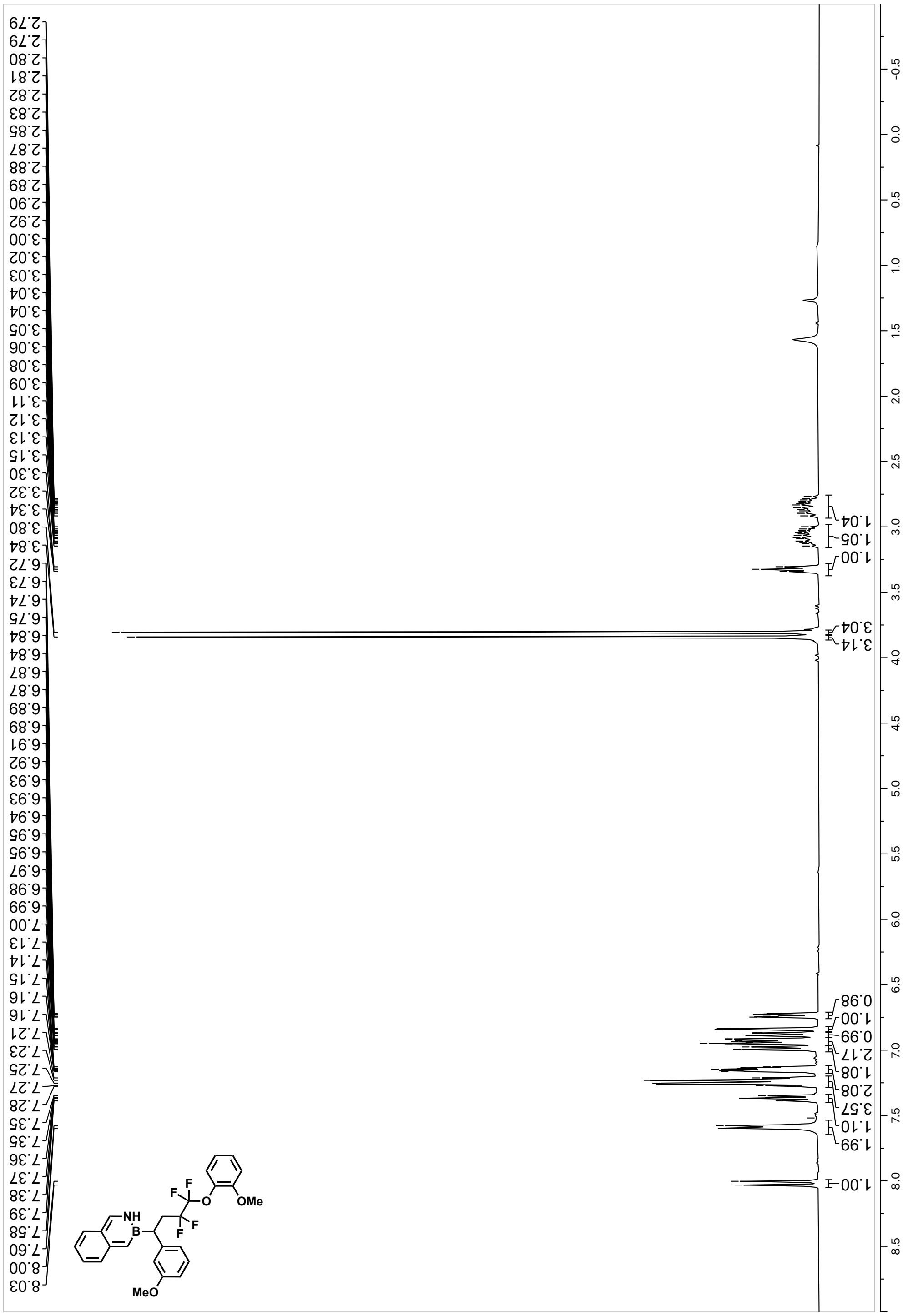

Compound 5l. ^{13}C NMR ($CDCl_3$, 100 MHz).

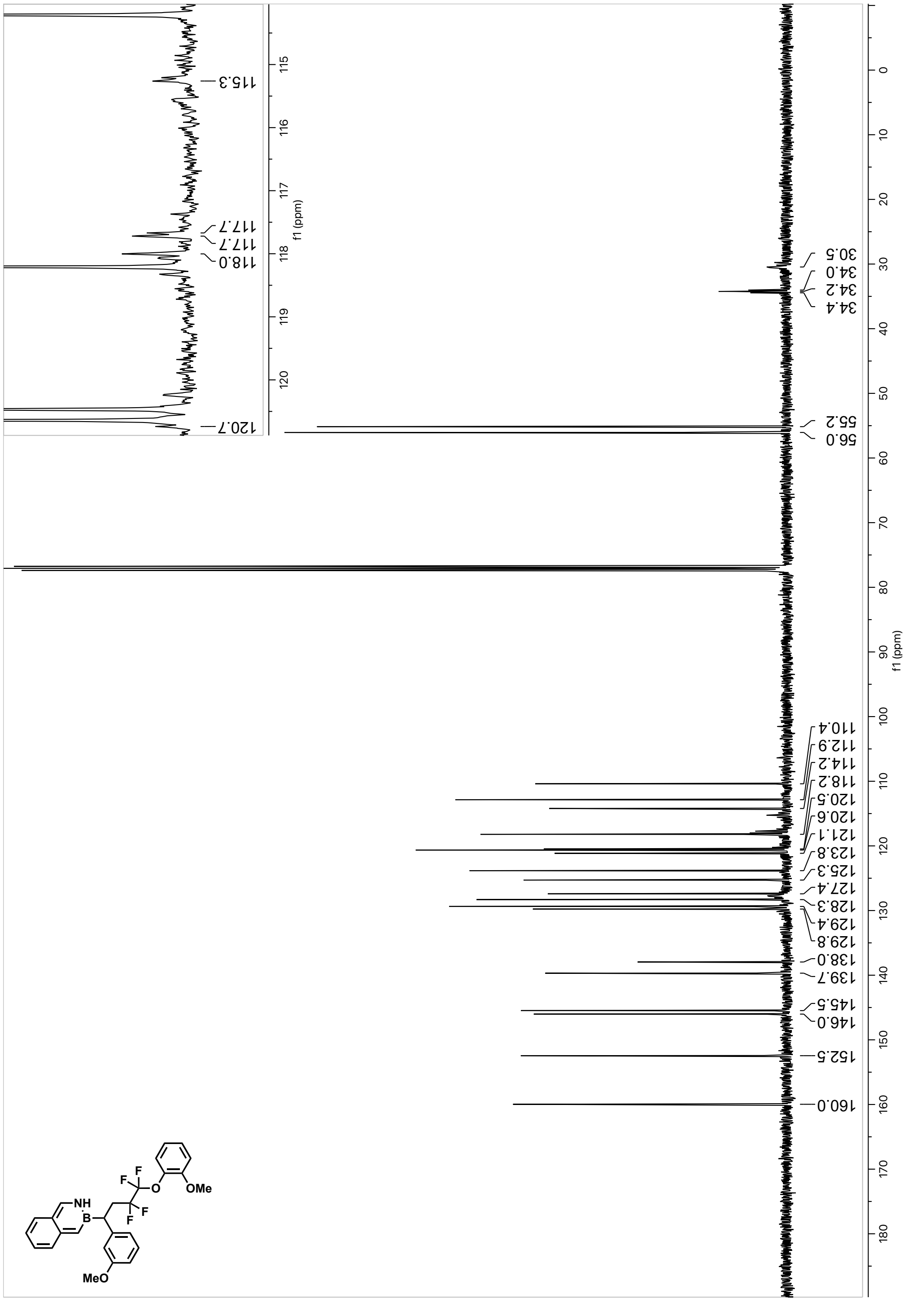

Compound 5l. ^{19}F NMR ($CDCl_3$, 376 MHz).

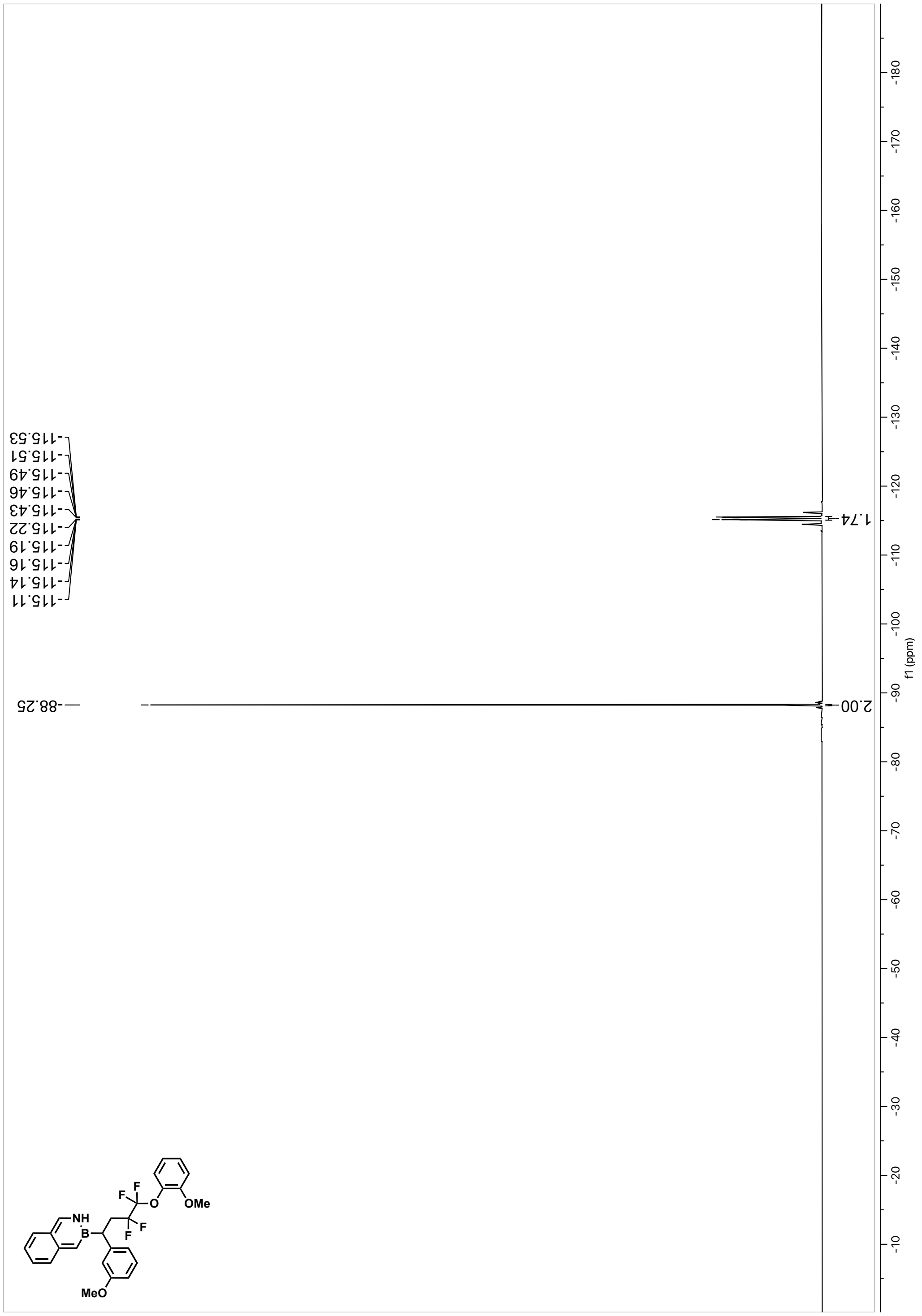

Compound 5l. ^{11}B NMR ($CDCl_3$, 128 MHz)

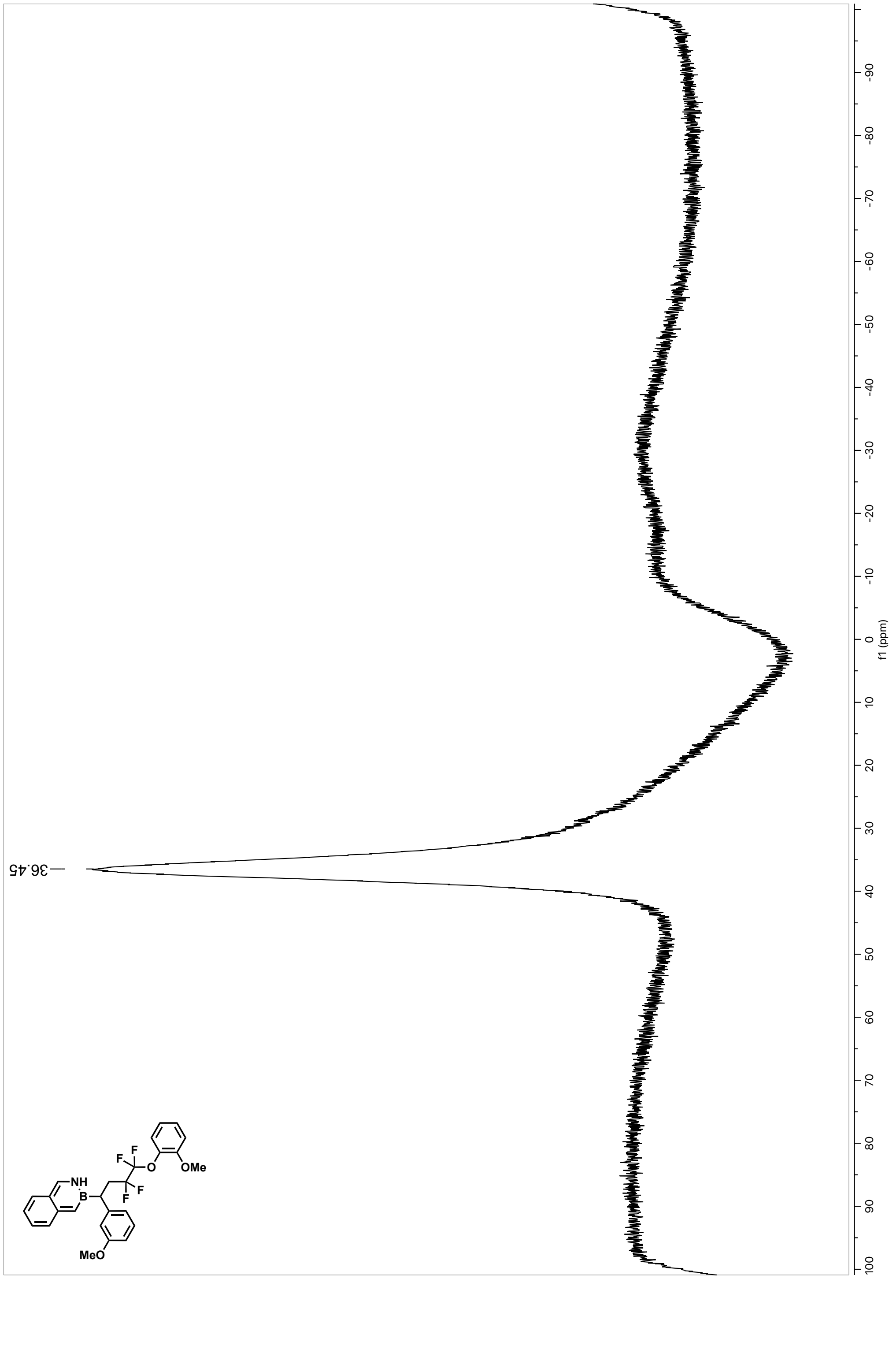

Compound 1a-3N. ^{1}H NMR ($CDCl_3$, 400 MHz).

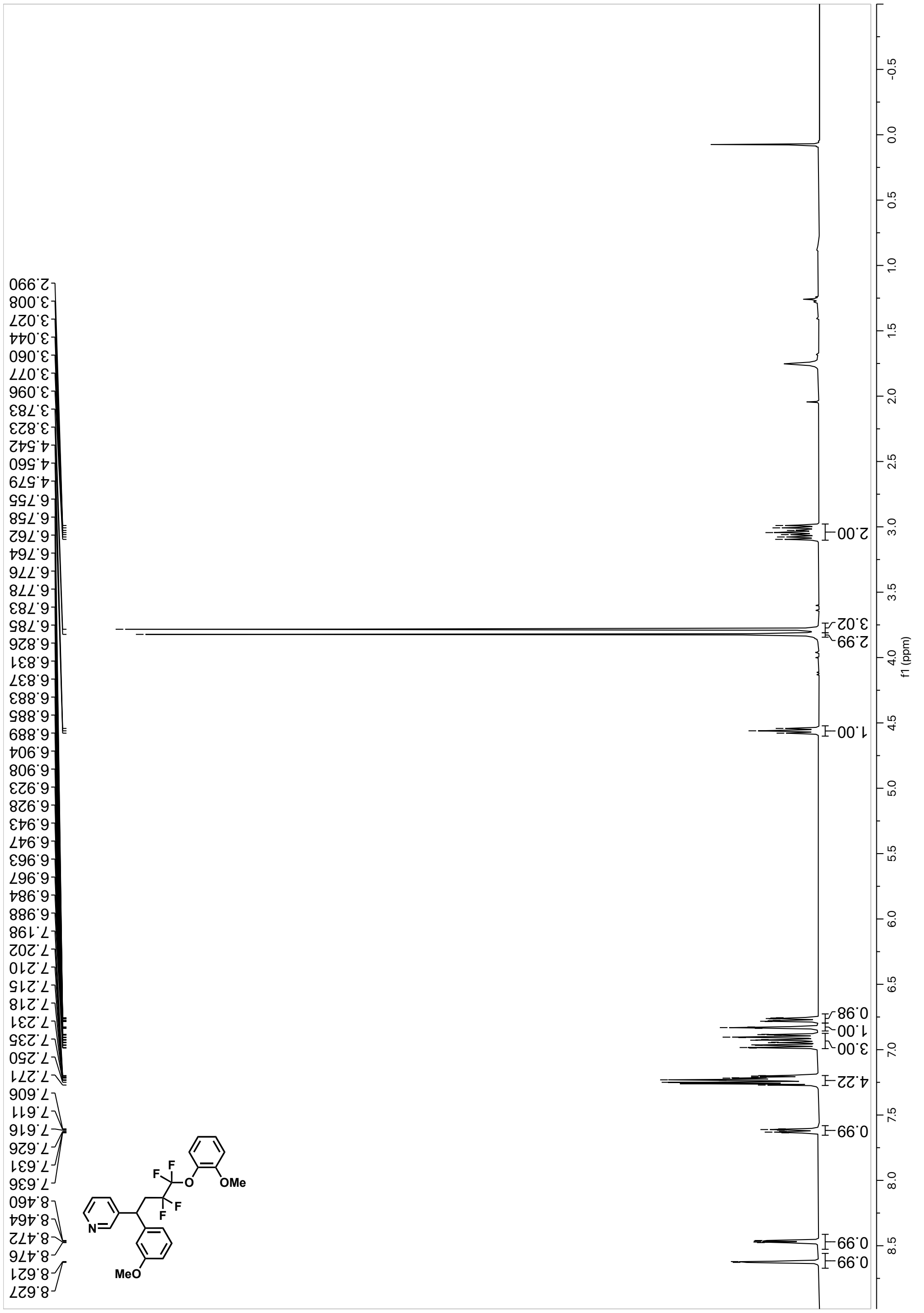

Compound 1a-3N. ^{13}C NMR (CDCl$_3$, 100 MHz).

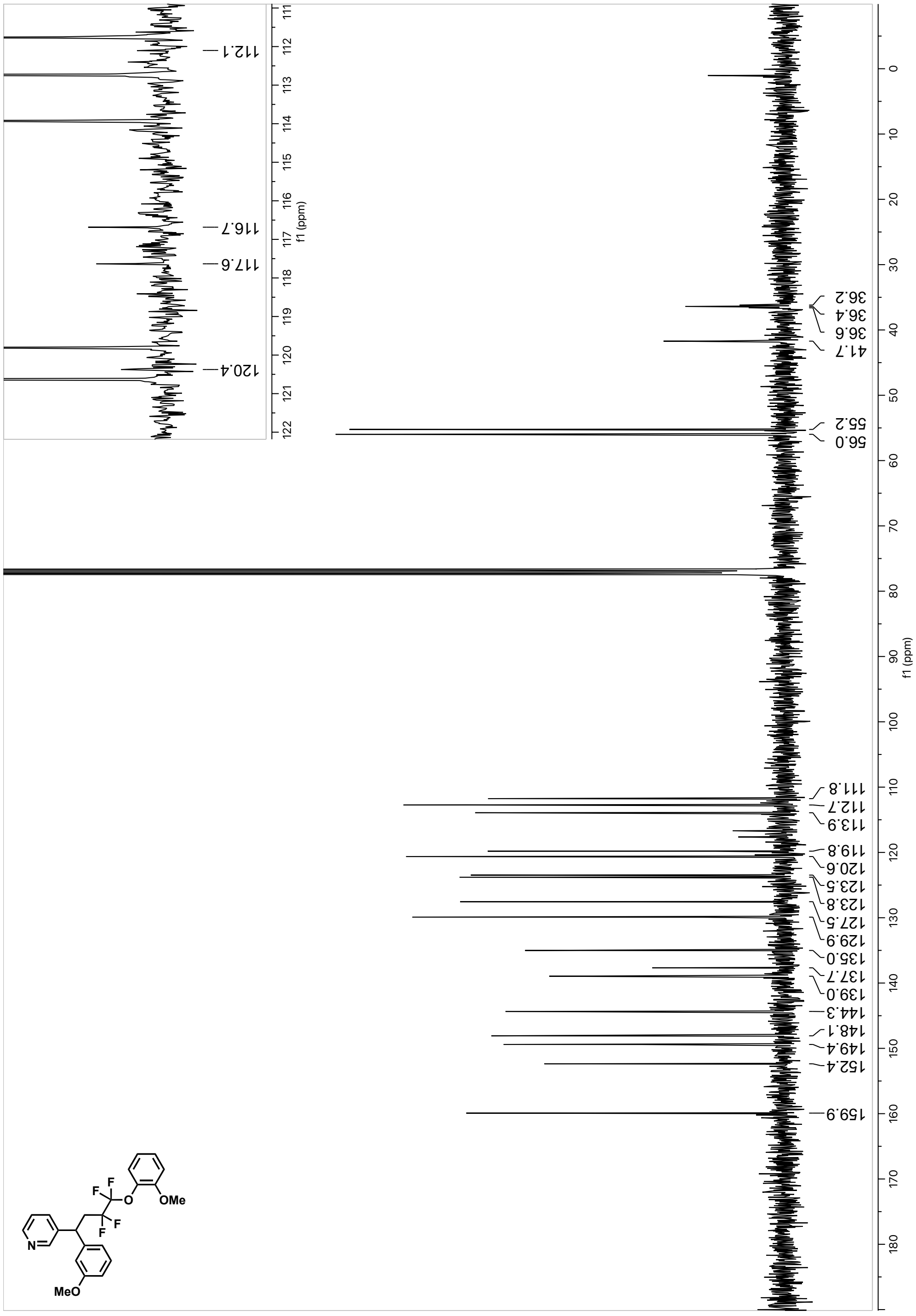

Compound 1a-3N. ^{19}F NMR ($CDCl_3$, 376 MHz).

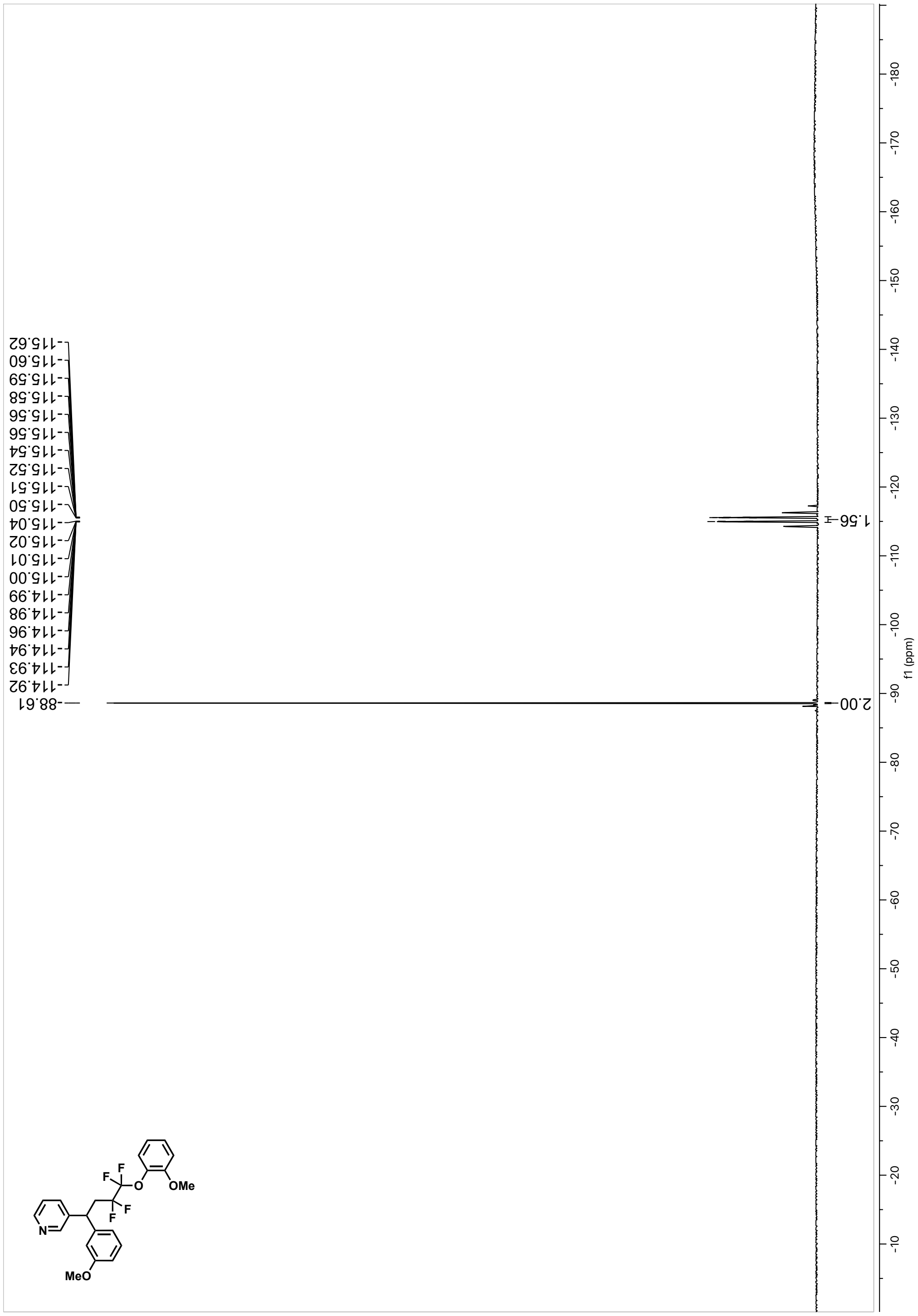

Compound 1a-4N. ^{1}H NMR ($CDCl_3$, 400 MHz).

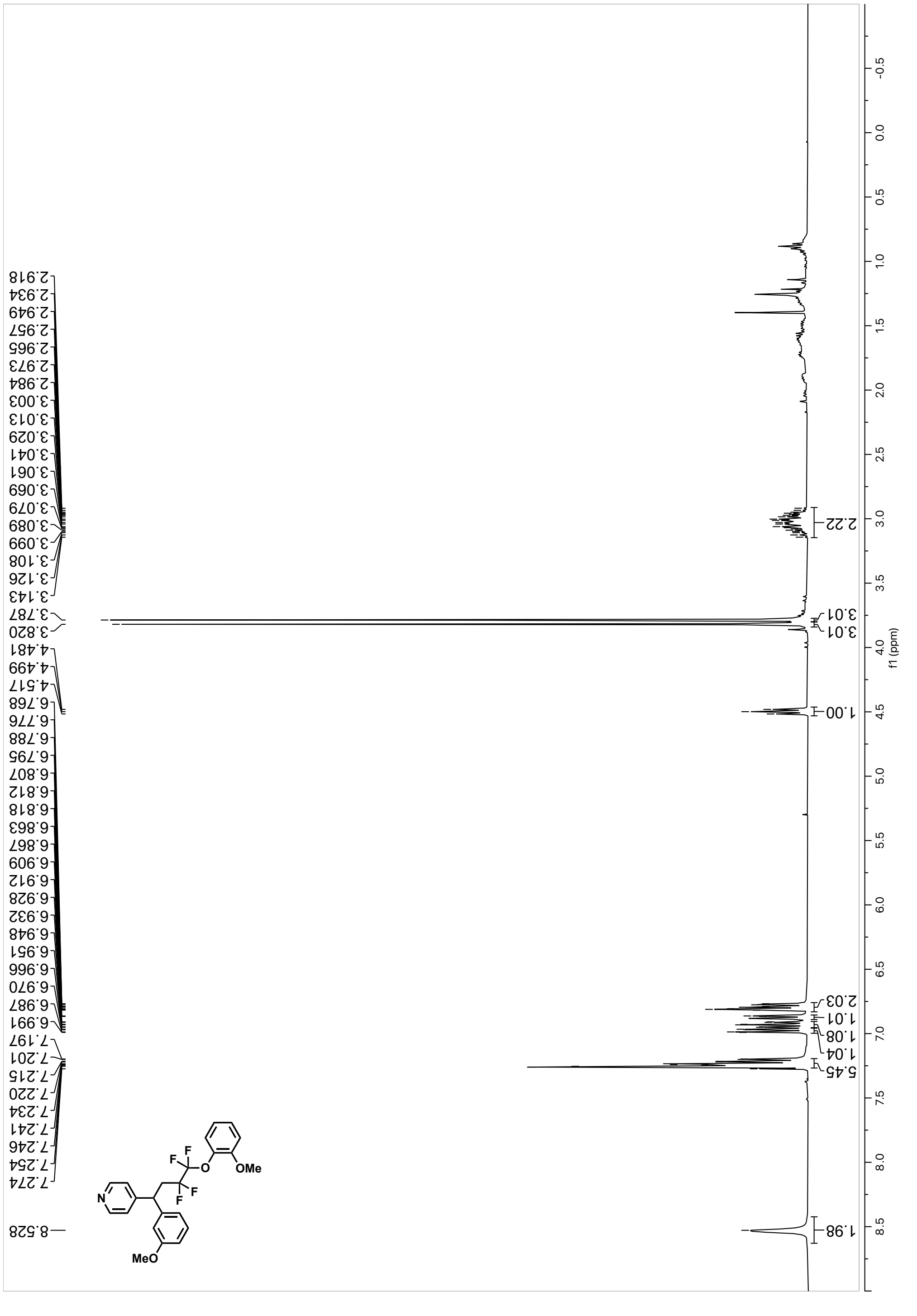

Compound 1a-4N. ^{13}C NMR ($CDCl_3$, 100 MHz).

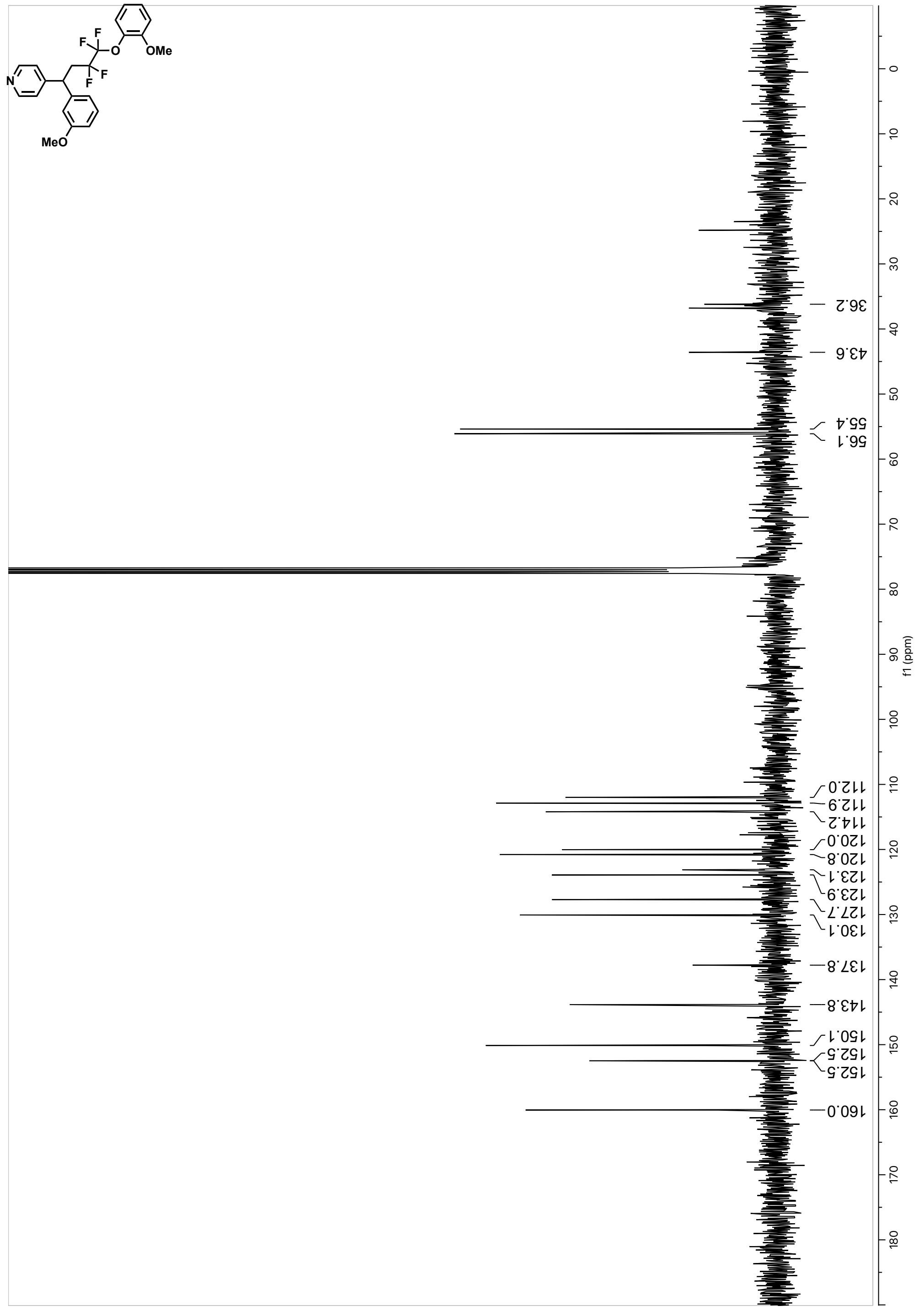

Compound 1a-4N. ^{19}F NMR ($CDCl_3$, 376 MHz).

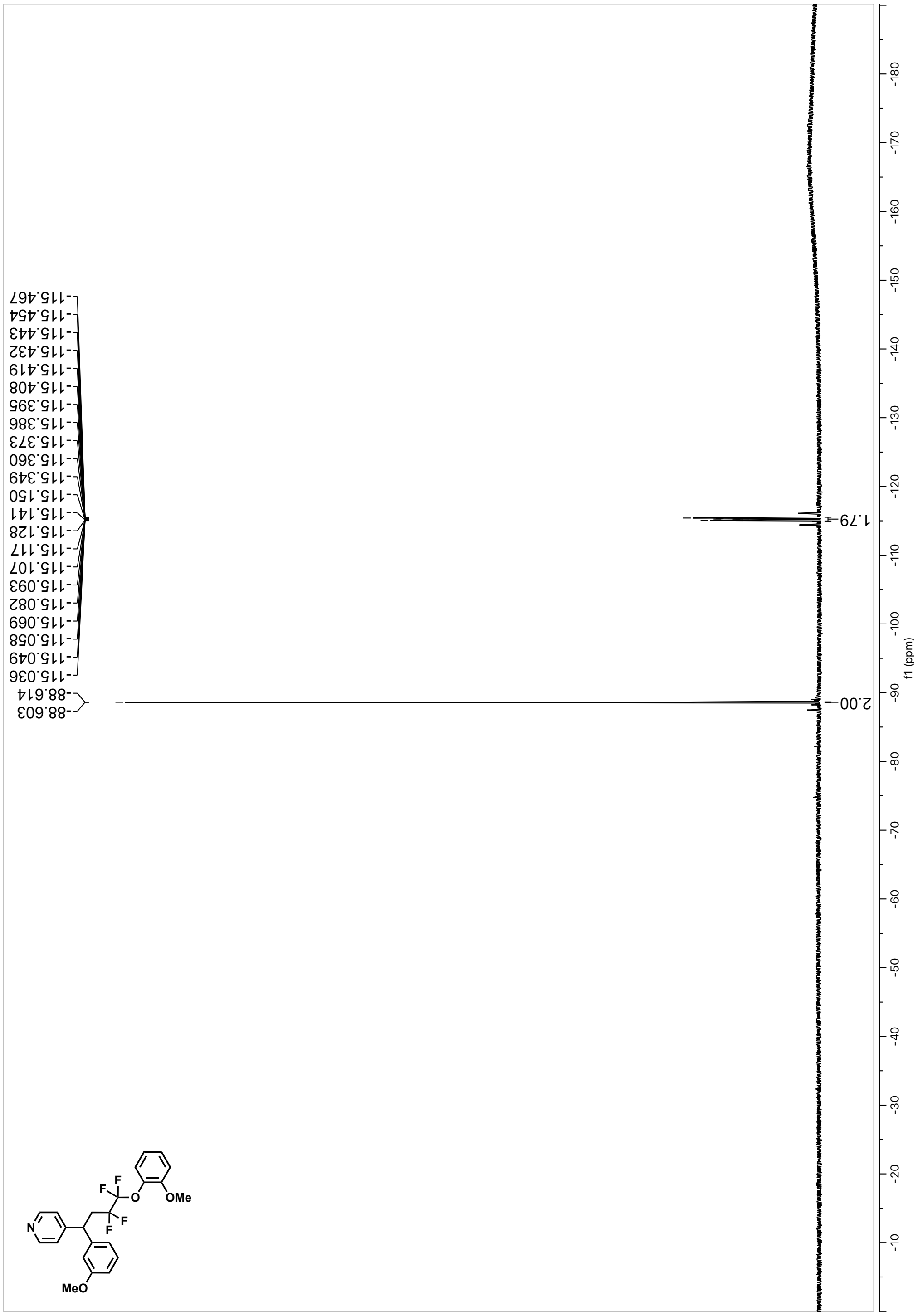

Compound 6a. ^{1}H NMR ($CDCl_3$, 400 MHz).

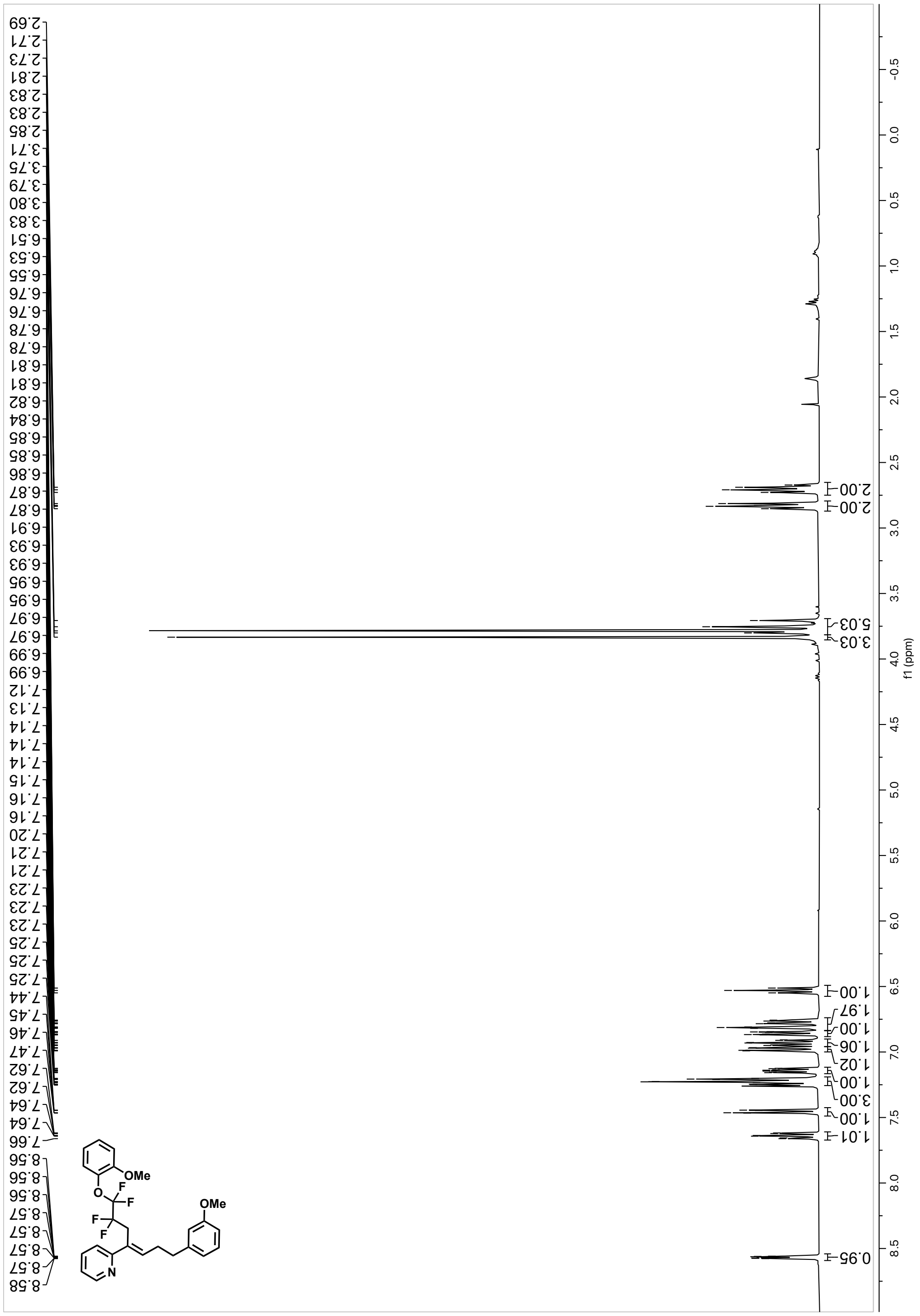

Compound 6a. ^{13}C NMR ($CDCl_3$, 100 MHz).

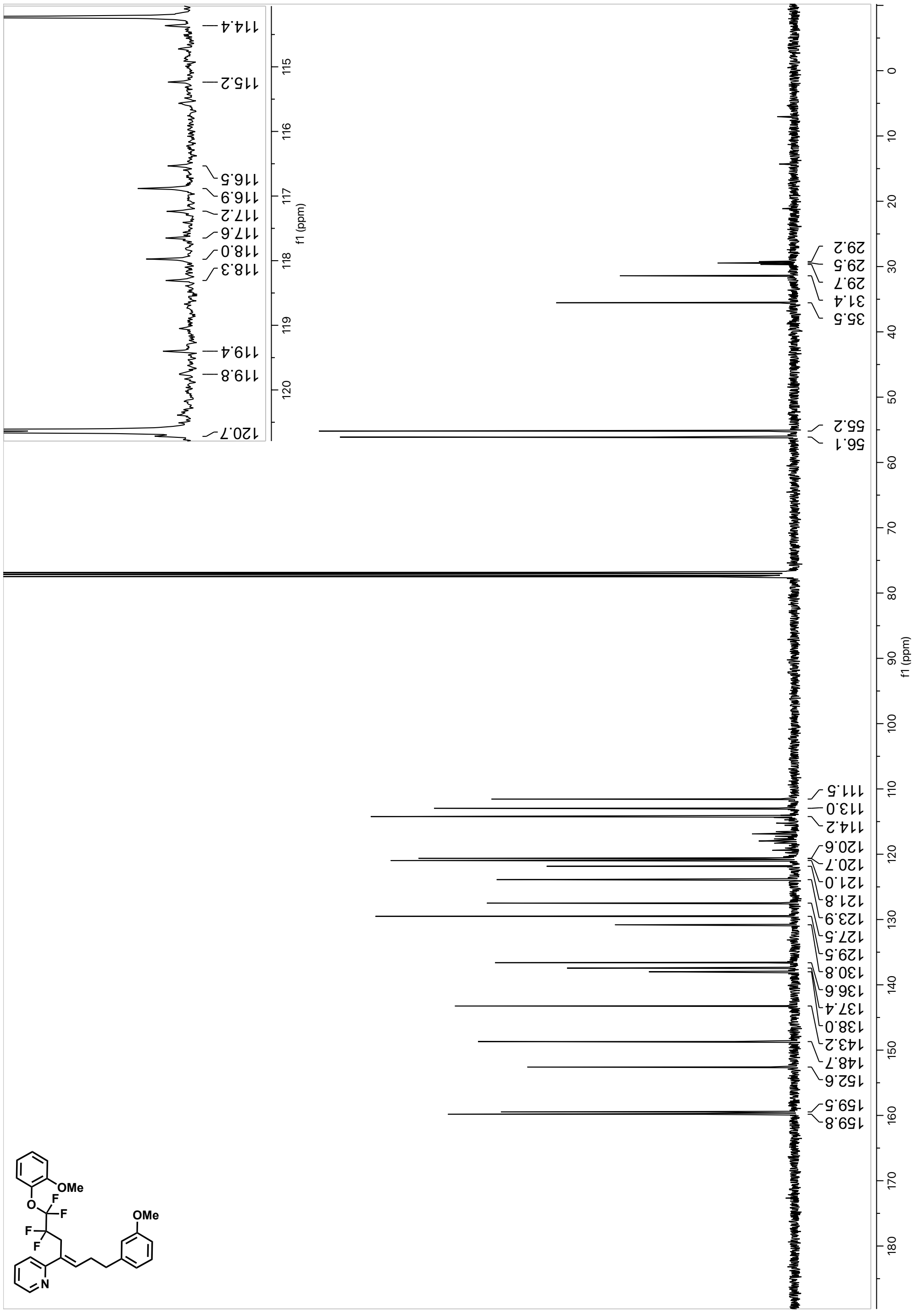

Compound 6a. ^{19}F NMR (CDCl$_3$, 376 MHz).

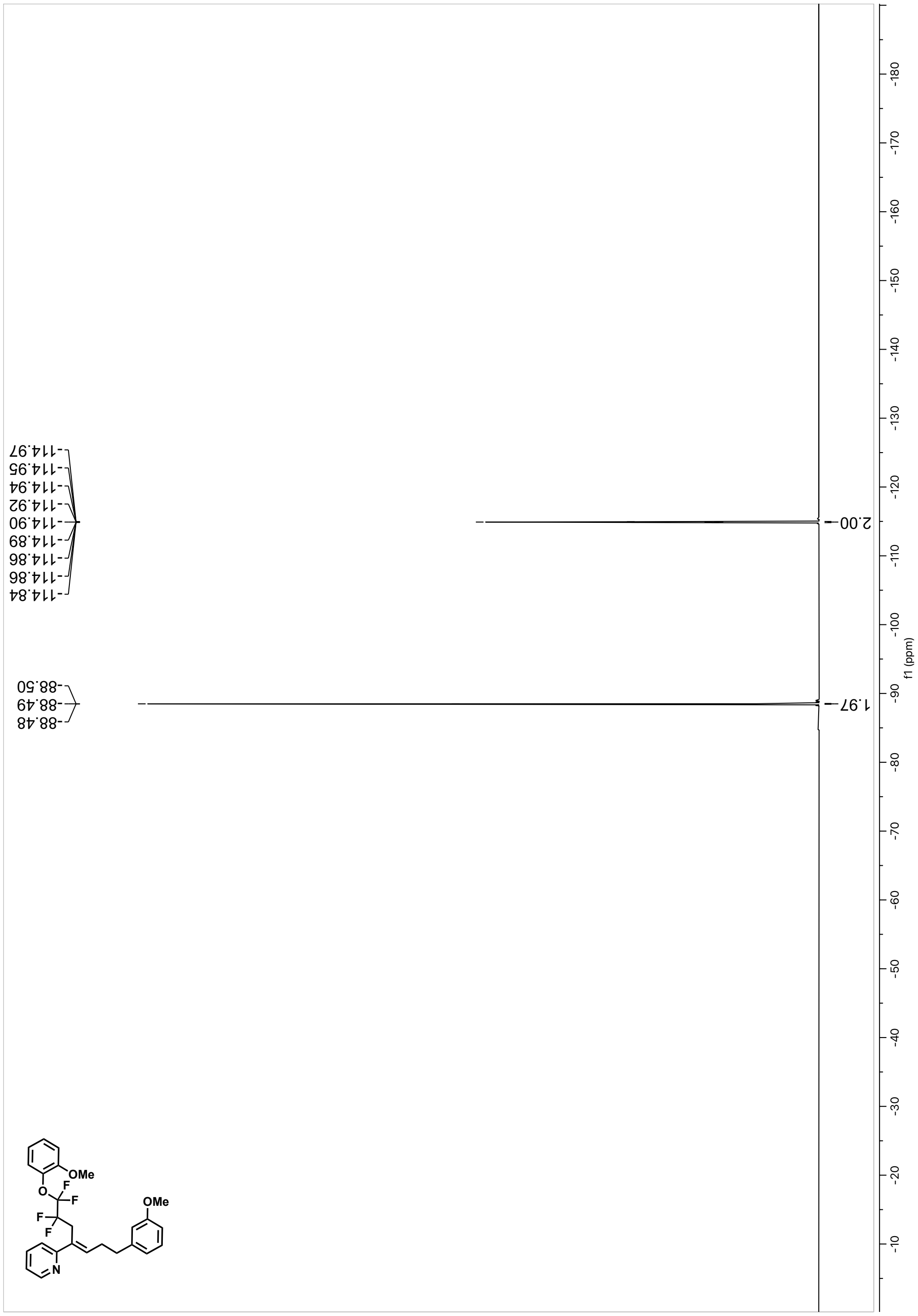

Compound 5k. ^{1}H NMR ($CDCl_3$, 400 MHz).

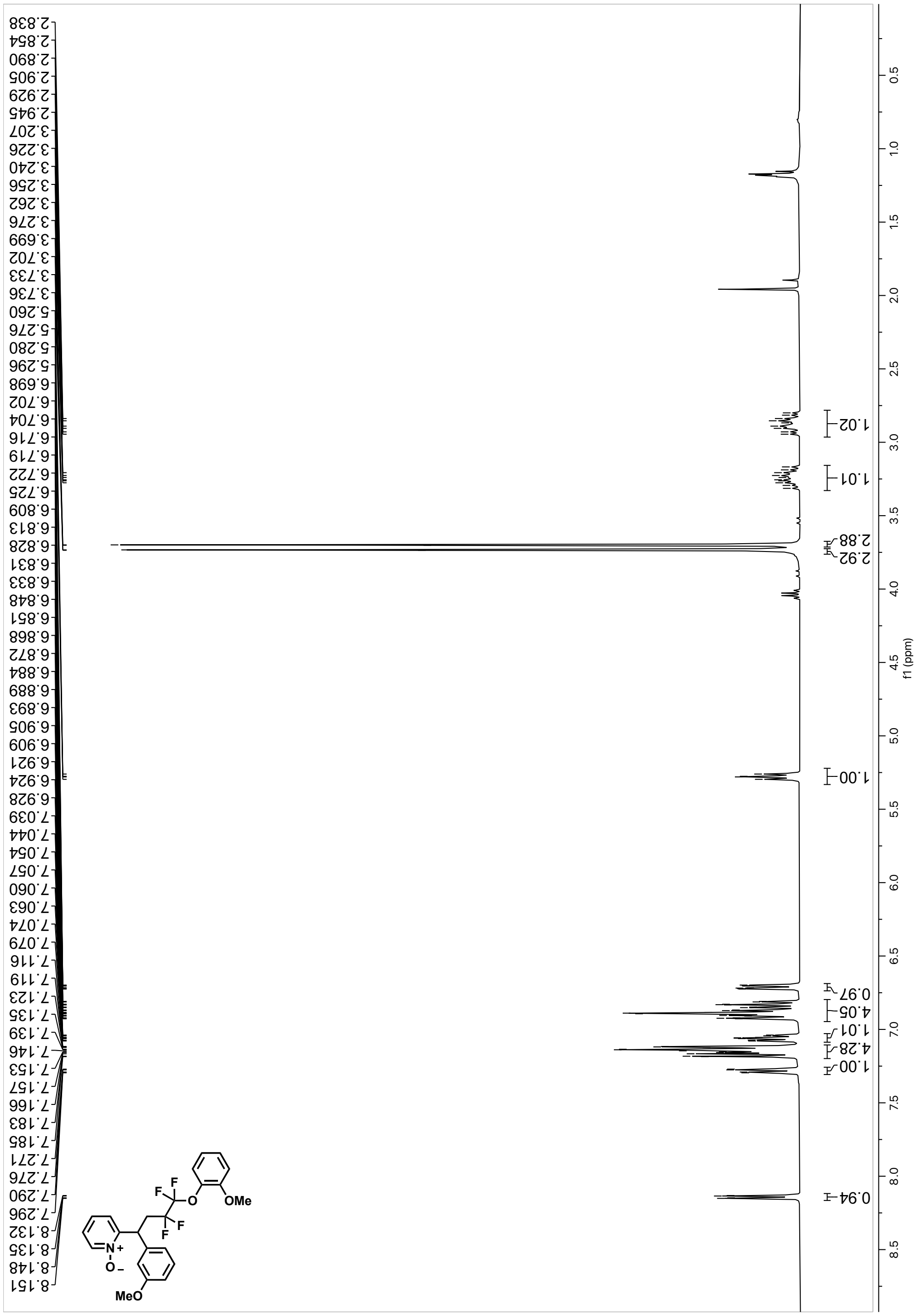

Compound 5k. ^{13}C NMR ($CDCl_3$, 100 MHz).

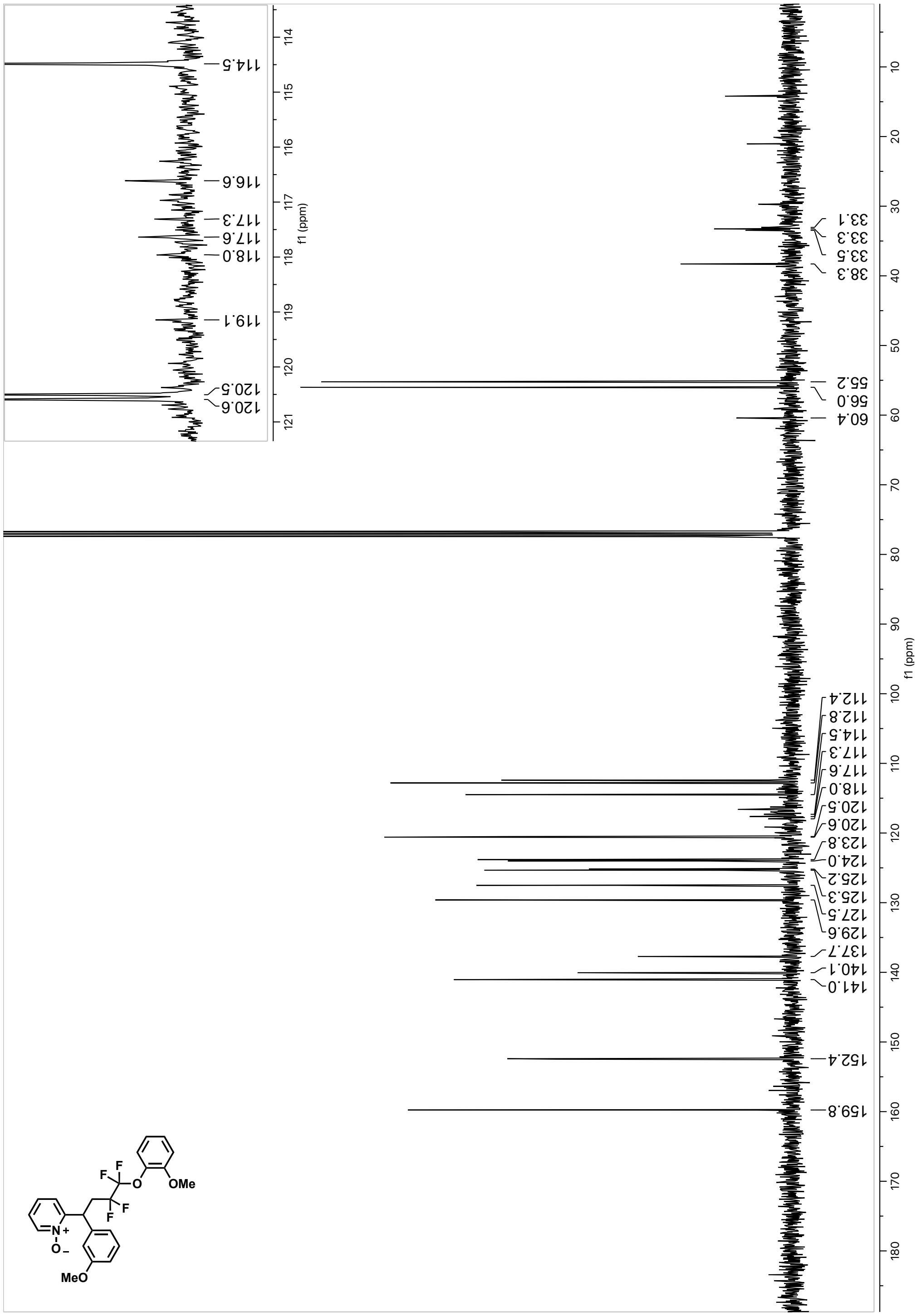

Compound 5k. ^{19}F NMR ($CDCl_3$, 376 MHz).

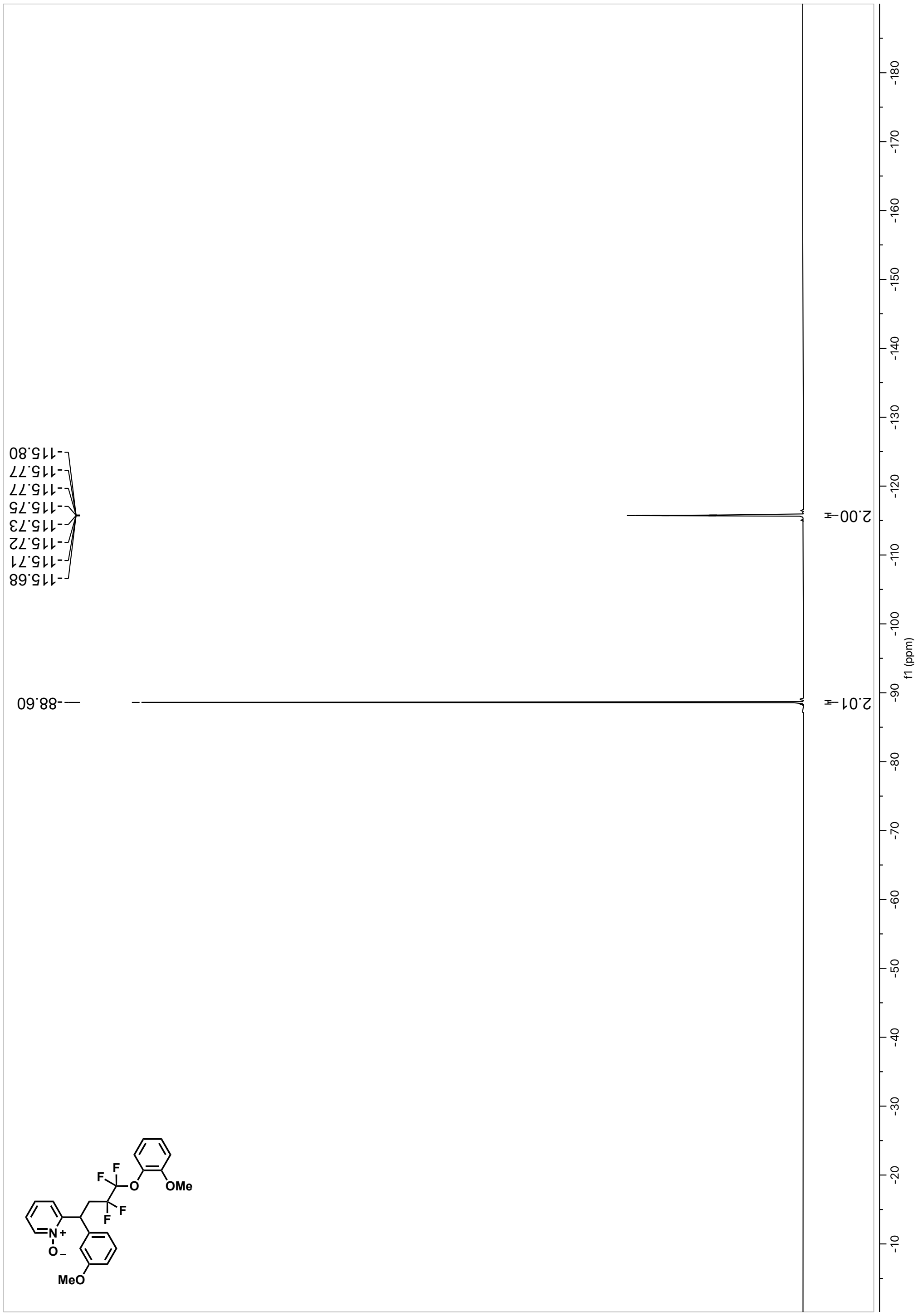

Compound B. Top: Full Mass Spectrum of Side Products. Bottom: Measured and Expected Mass for **Compound B**

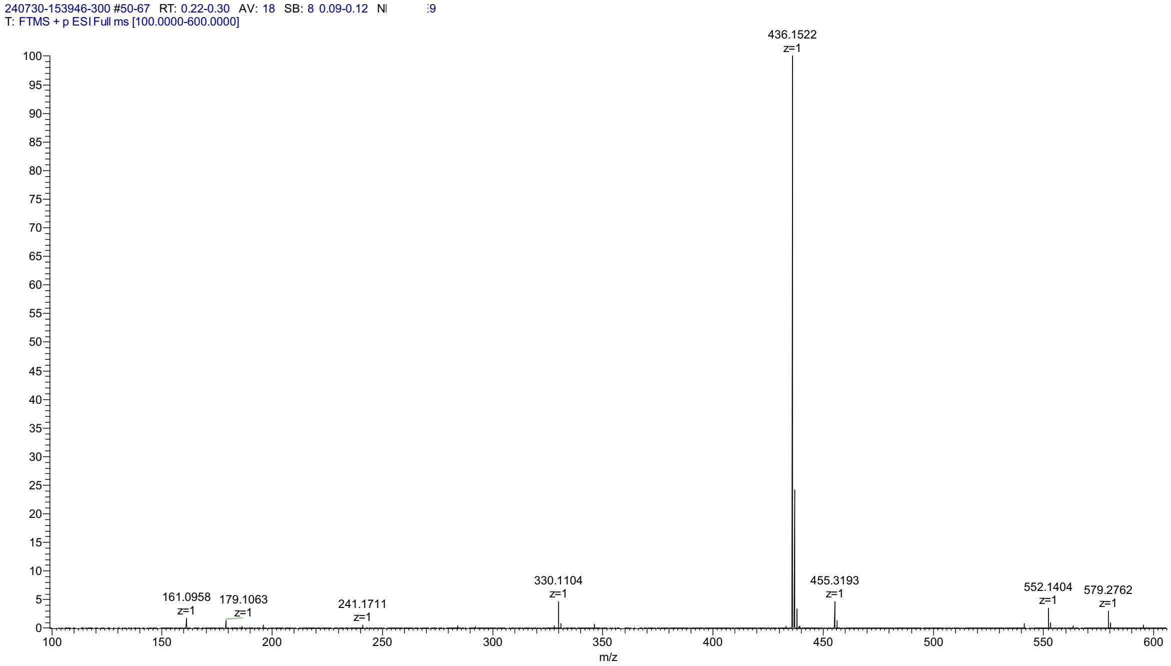

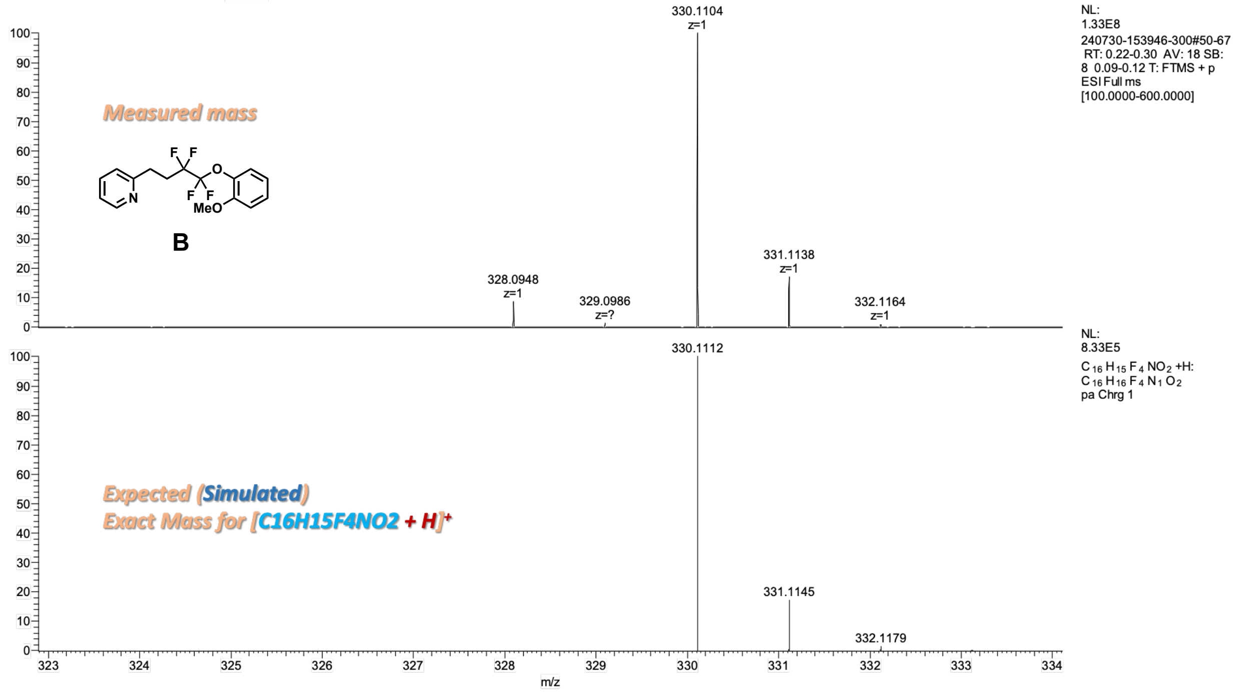

Compound C. Top: Full Mass Spectrum of Side Products. Bottom: Measured and Expected Mass for **Compound C**

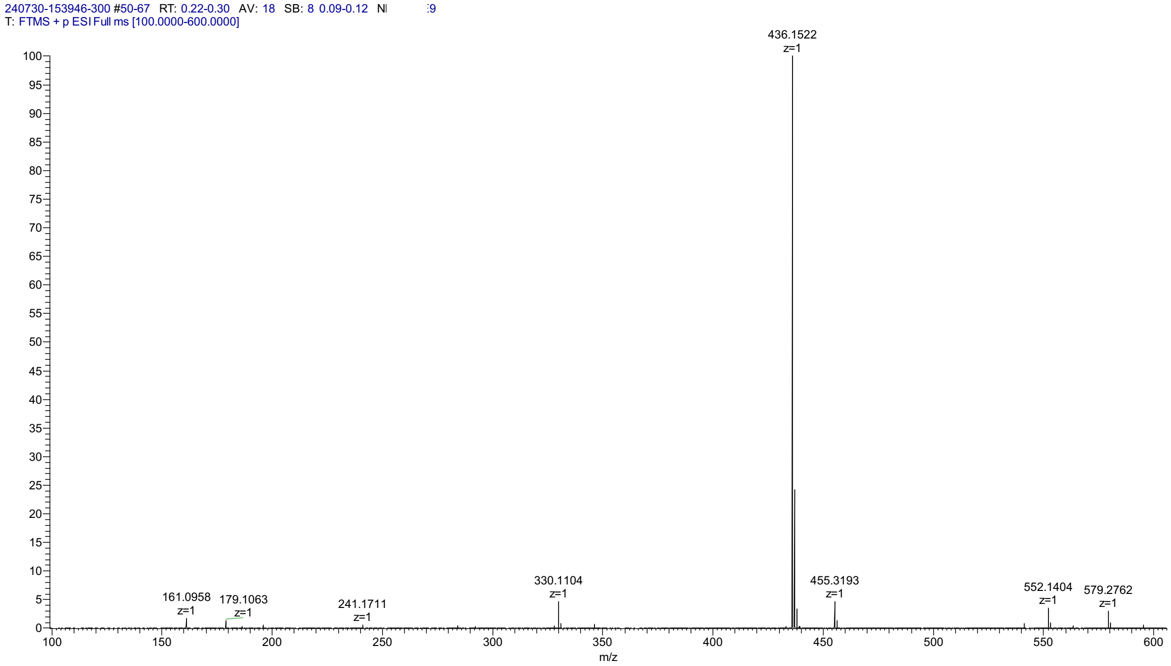

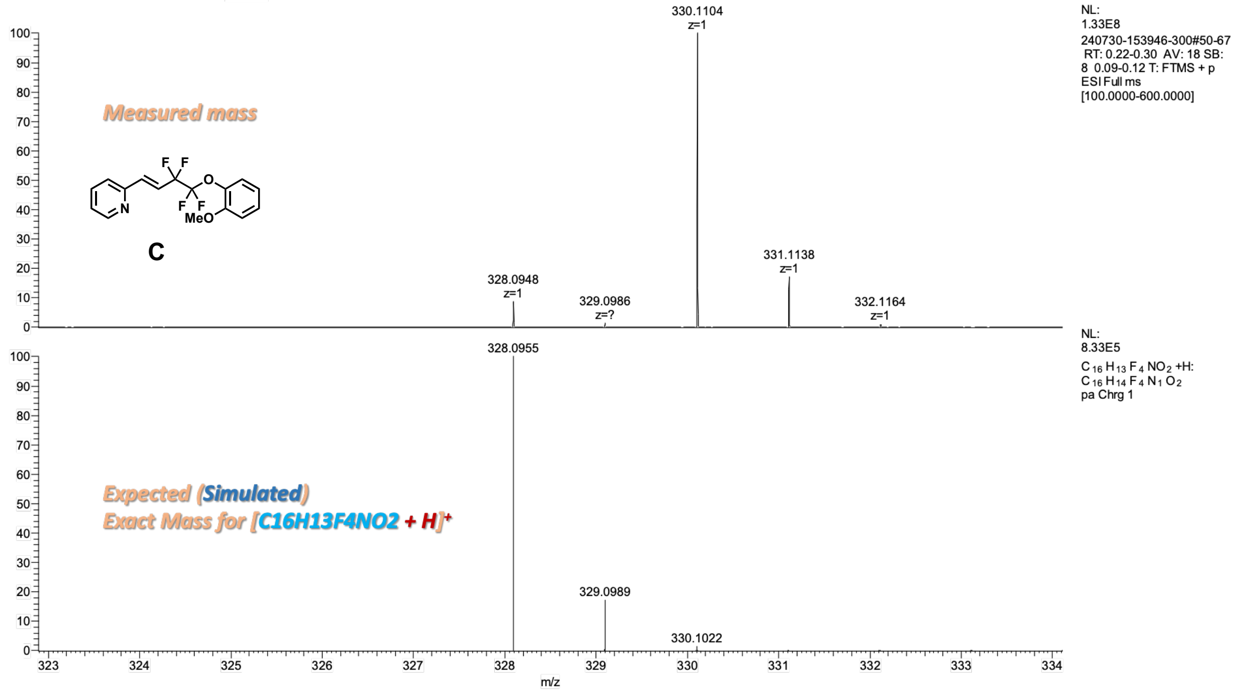

Compound D. Top: Full Mass Spectrum of Side Products. Bottom: Measured and Expected Mass for **Compound D**

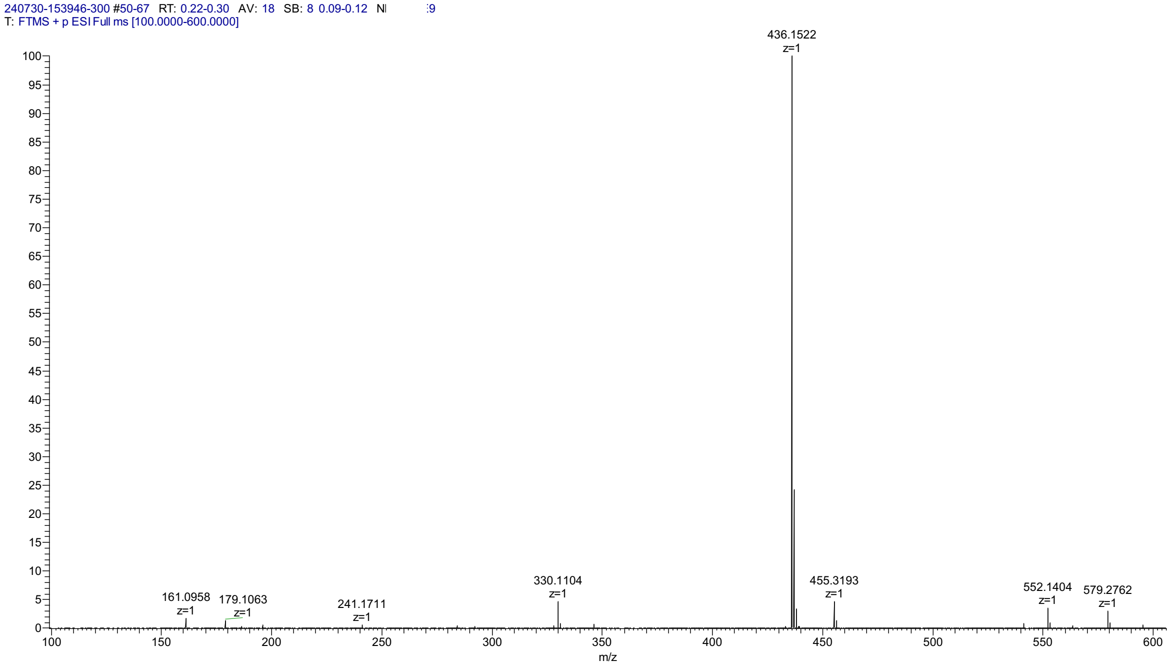

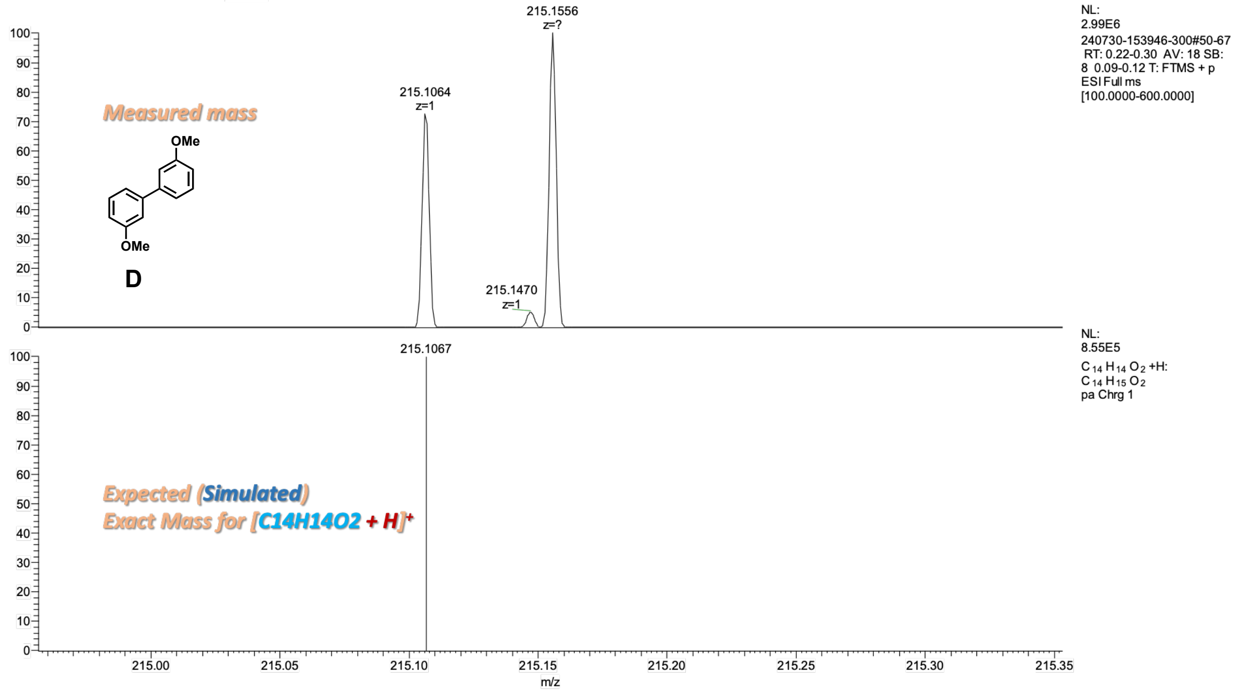

Compound E. Top: Full Mass Spectrum of Side Products. Bottom: Measured and Expected Mass for **Compound E**

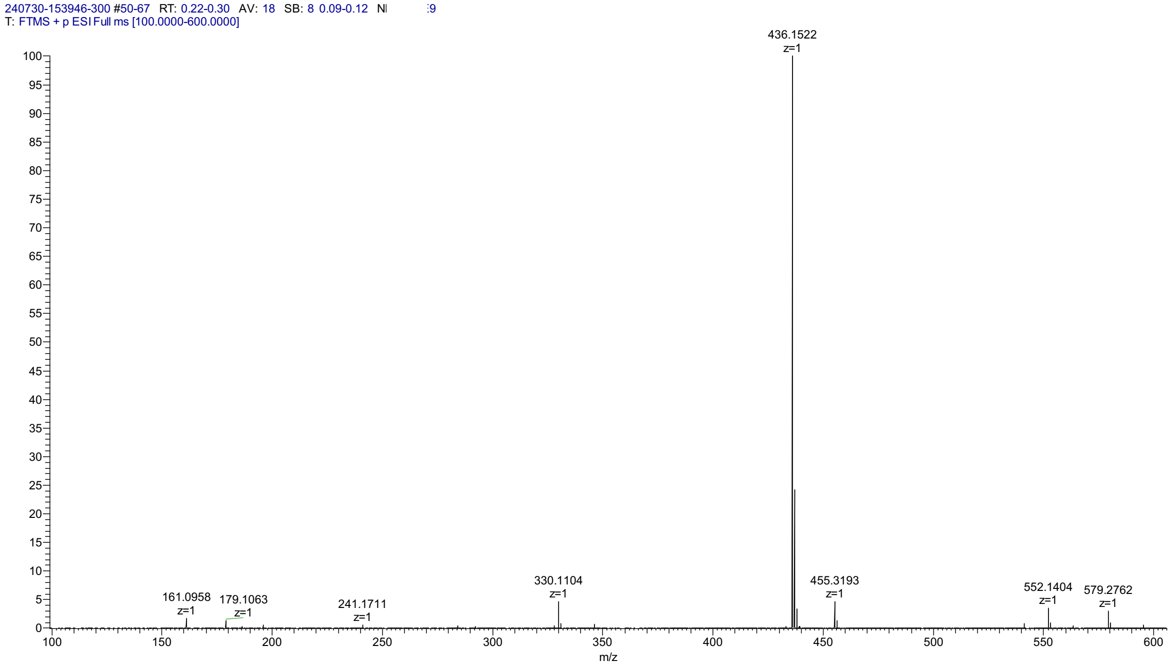

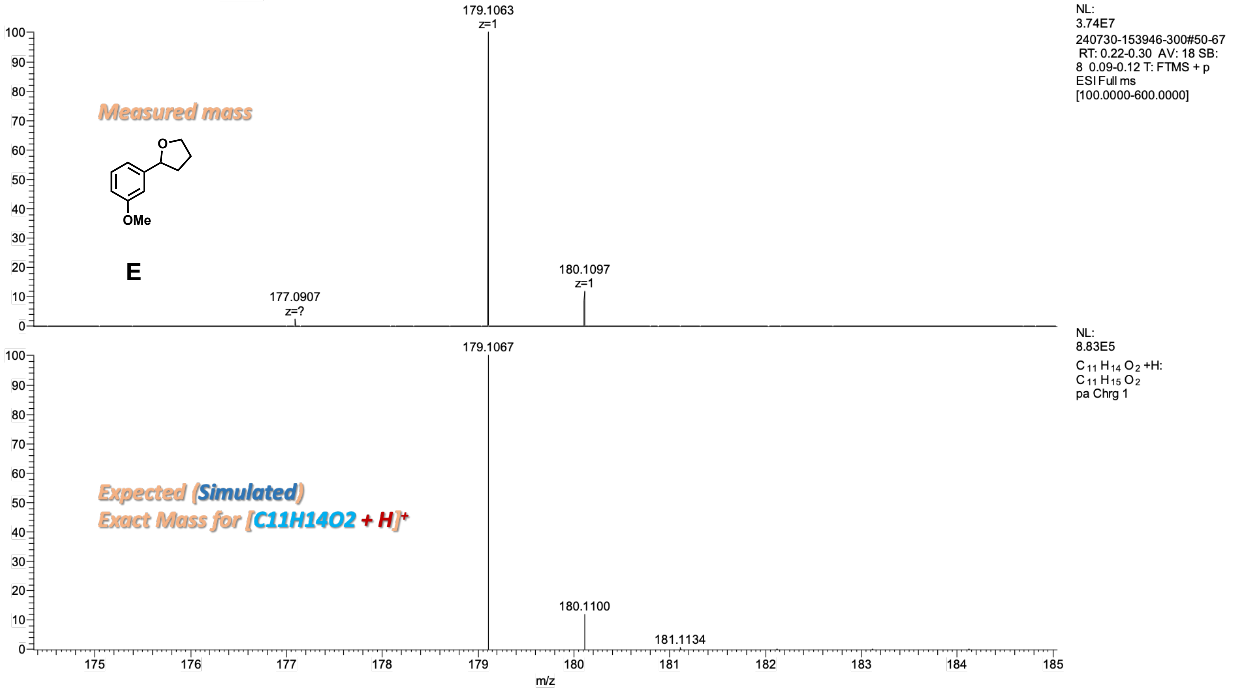

Compound 7a. Top: Full Mass Spectrum. Bottom: Measured and Expected Mass

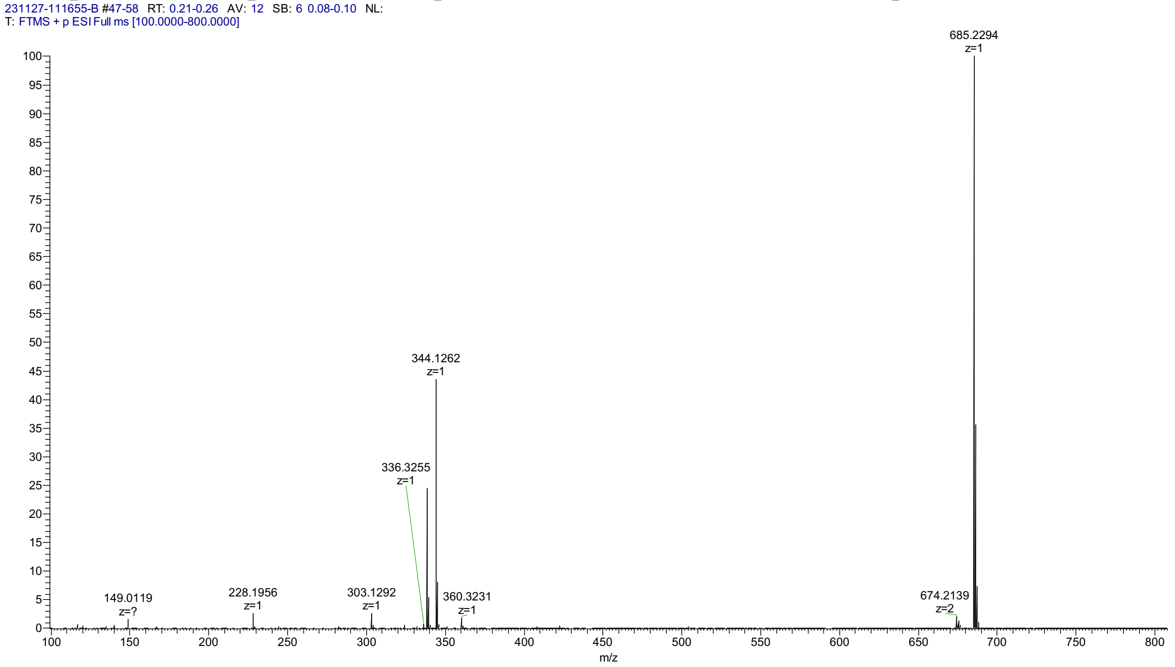

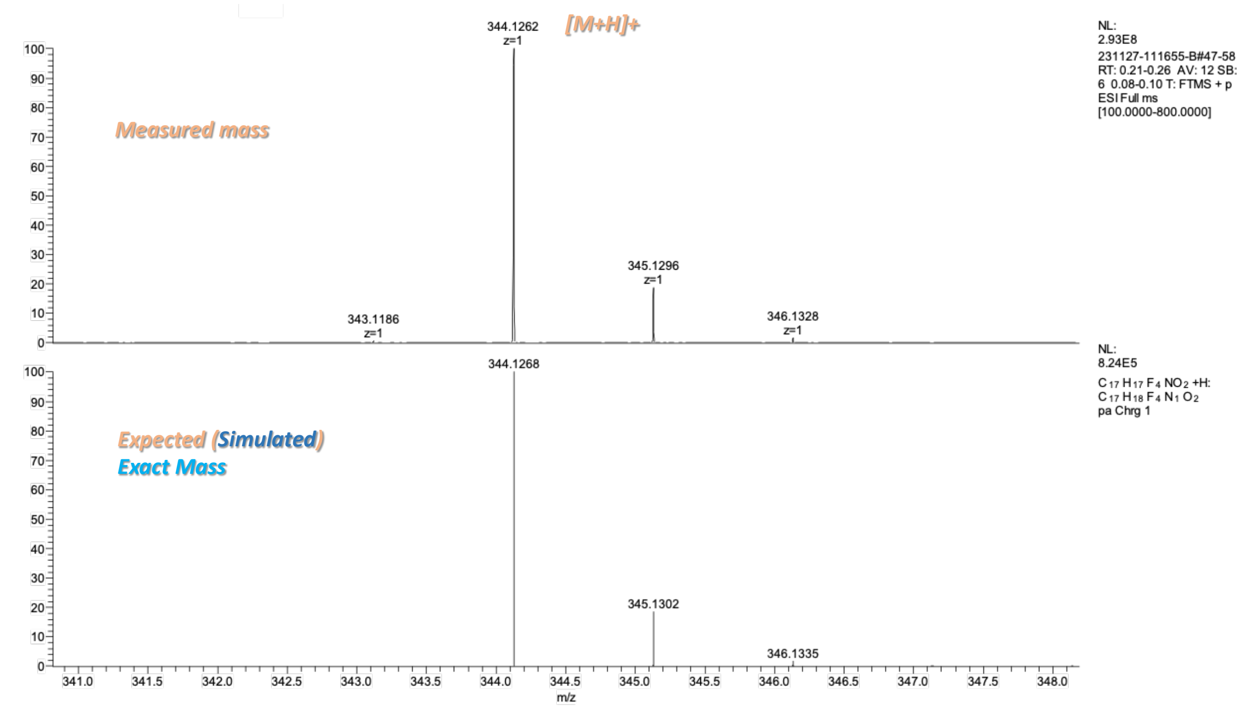

Compound 7b. Top: Full Mass Spectrum. Bottom: Measured and Expected Mass

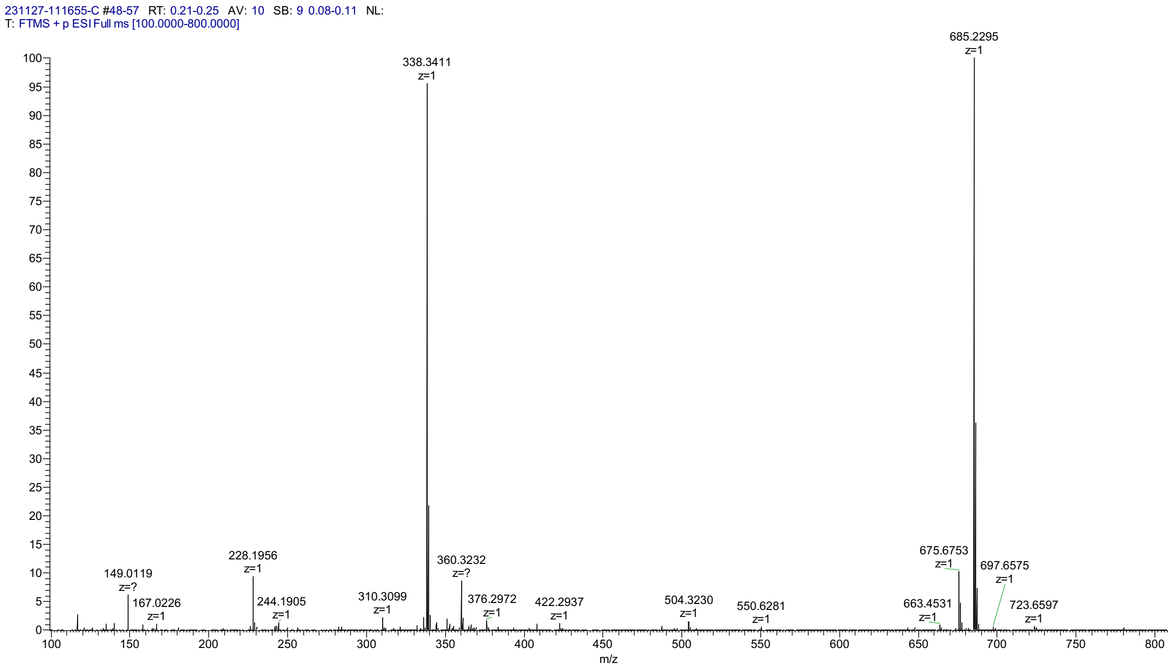

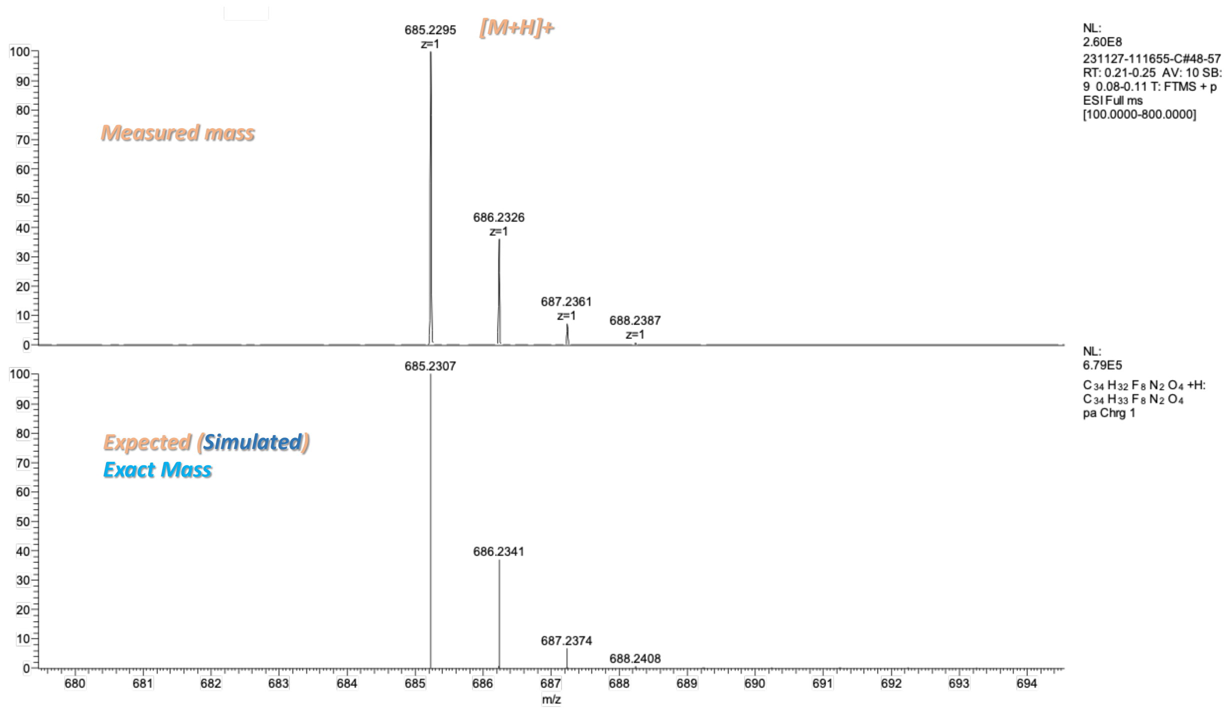

Compound 8a. Top: Full Mass Spectrum. Bottom: Measured and Expected Mass

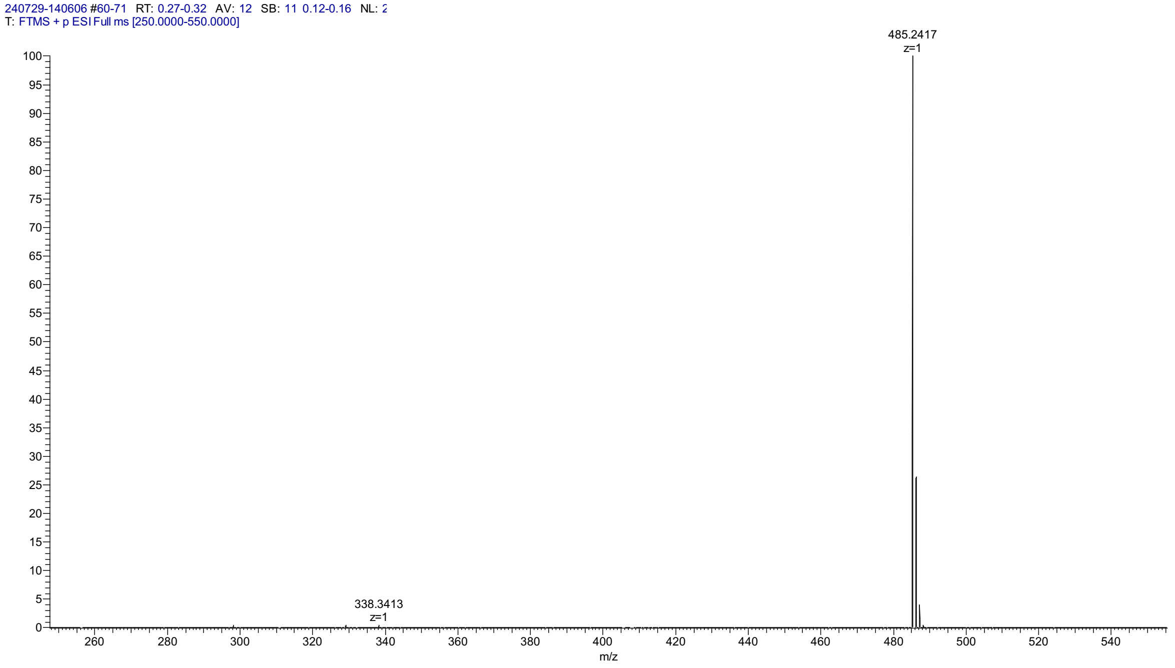

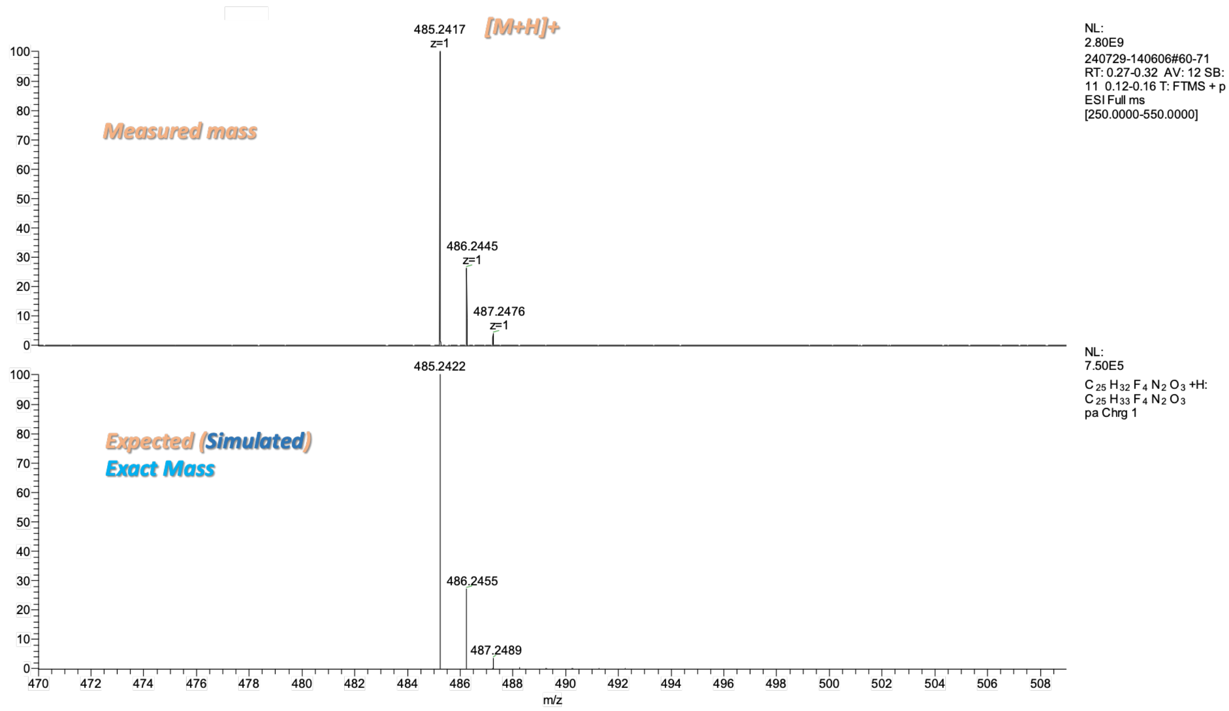

Compound 8b. Top: Full Mass Spectrum. Bottom: Measured and Expected Mass

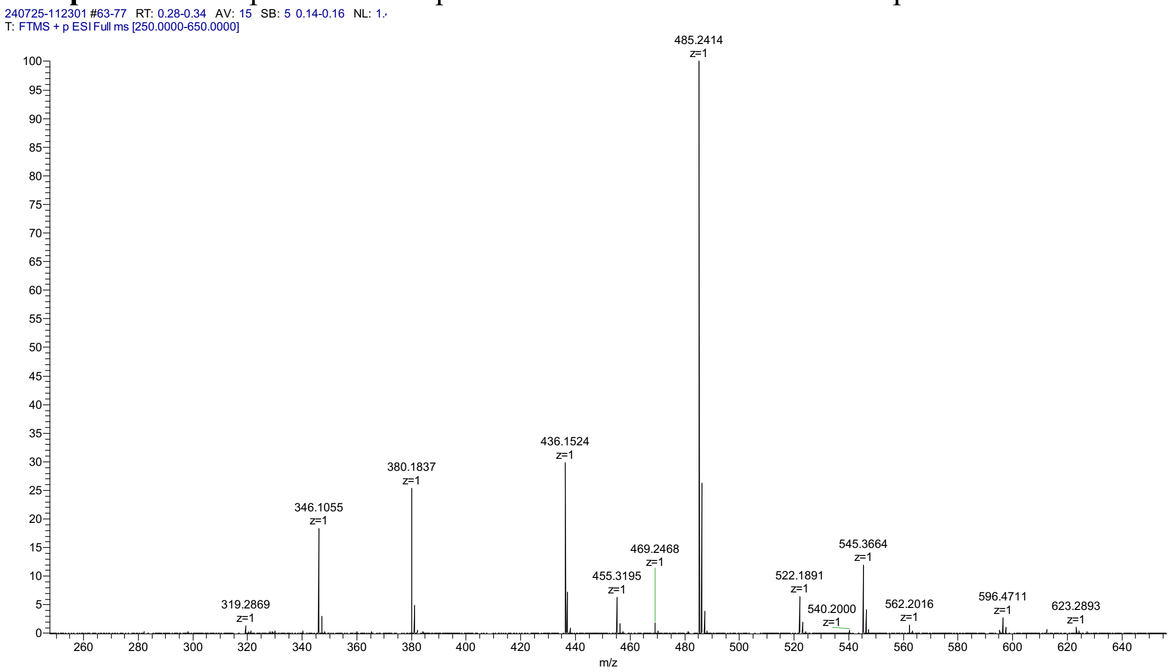

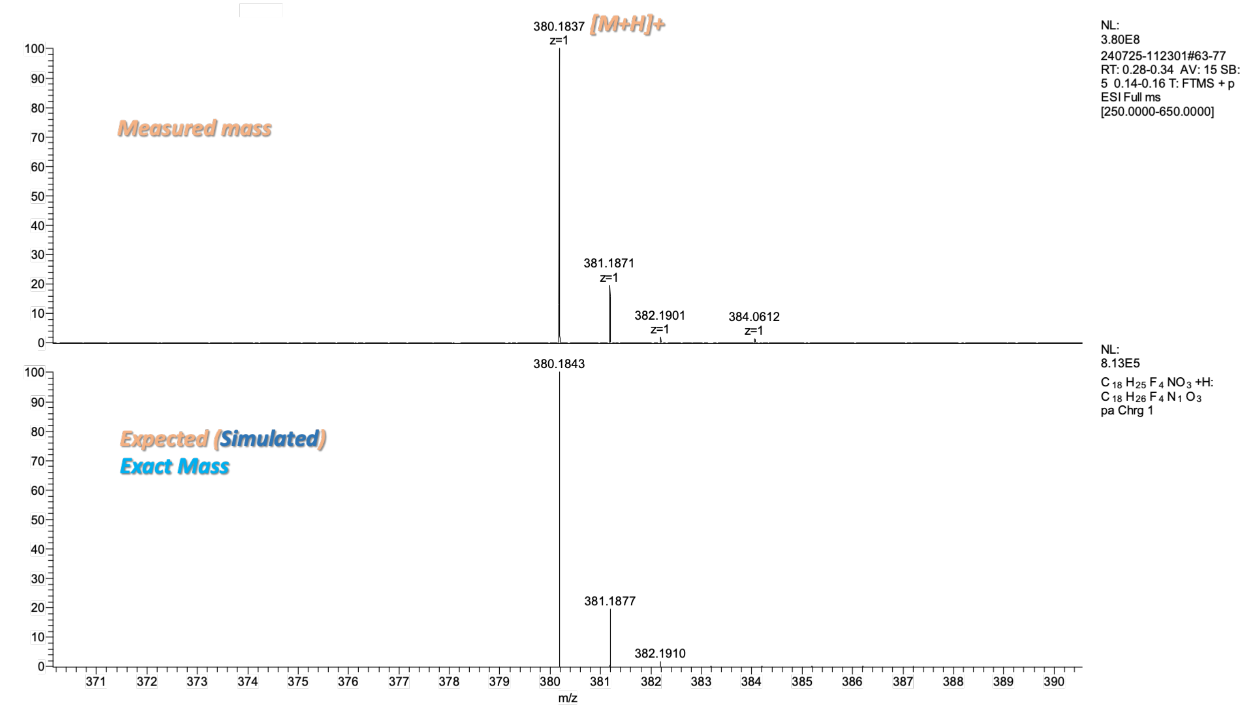

15. Computational Methods, Results, and Coordinates

All geometry optimizations of intermediates and transition states were achieved using spin unrestricted UB3LYP[6]-D3[7]/6-31G(d)[8] method with "opt=noeigen" and "guess=mix" keywords as implemented in Gaussian16[9]. Frequency calculations were also conducted at the same level of theory to obtain vibrational frequencies to determine the identity of stationary points as intermediates (no imaginary frequencies) or transition states (only one imaginary frequency), as well as obtain thermal correction to enthalpy and free energy at 298 K. Intrinsic Reaction Coordinate (IRC) calculations were done on the transition states to verify the correct transition state associated with the reaction. The endpoint geometries obtained from the IRC calculations were further optimized to verify the authenticity of the transition state. Also, an extensive conformational search was performed for all the organoiron intermediates and transition states. Additionally, the spin of each intermediate and transition state for the organoiron species was selected based on our previous mechanistic reports of related catalytic cycles.[10] Only the lowest-energy species were shown and discussed. The solvent effects were taken into consideration using the SMD solvent model [11] with tetrahydrofuran as the solvent used experimentally. Finally, we performed single-point calculations on the optimized geometries using the more robust uM06L[12]-D3/def2-TZVPP[13] method to improve our numerical precision. Therefore, the final reported energy values are calculated at the uM06L-D3/def2-TZVPP-SMD(THF)// uB3LYP-D3/6-31G(d)-SMD(THF) level of theory.

All structural figures were generated with CYLview.[14] Distances in structural figures are shown in Å and energies are in kcal/mol.

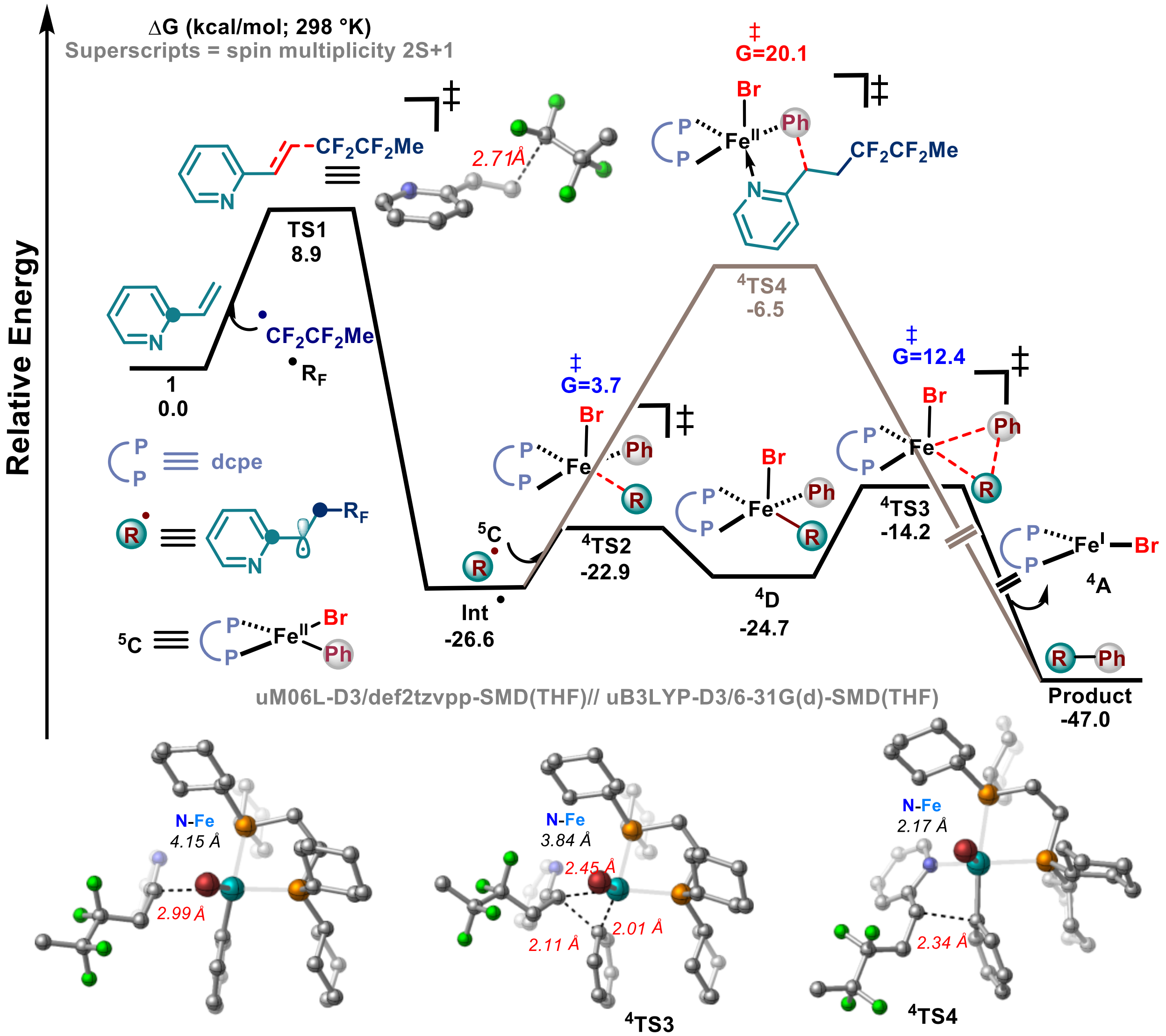

Figure S10. DFT calculation for dicarbofunctionalization of vinyl pyridine. Calculated Gibbs free energies [uM06L-D3/def2tzvpp-SMD(THF)// uB3LYP-D3/6-31G(d)-SMD(THF)] are given in kcal mol^{-1}.

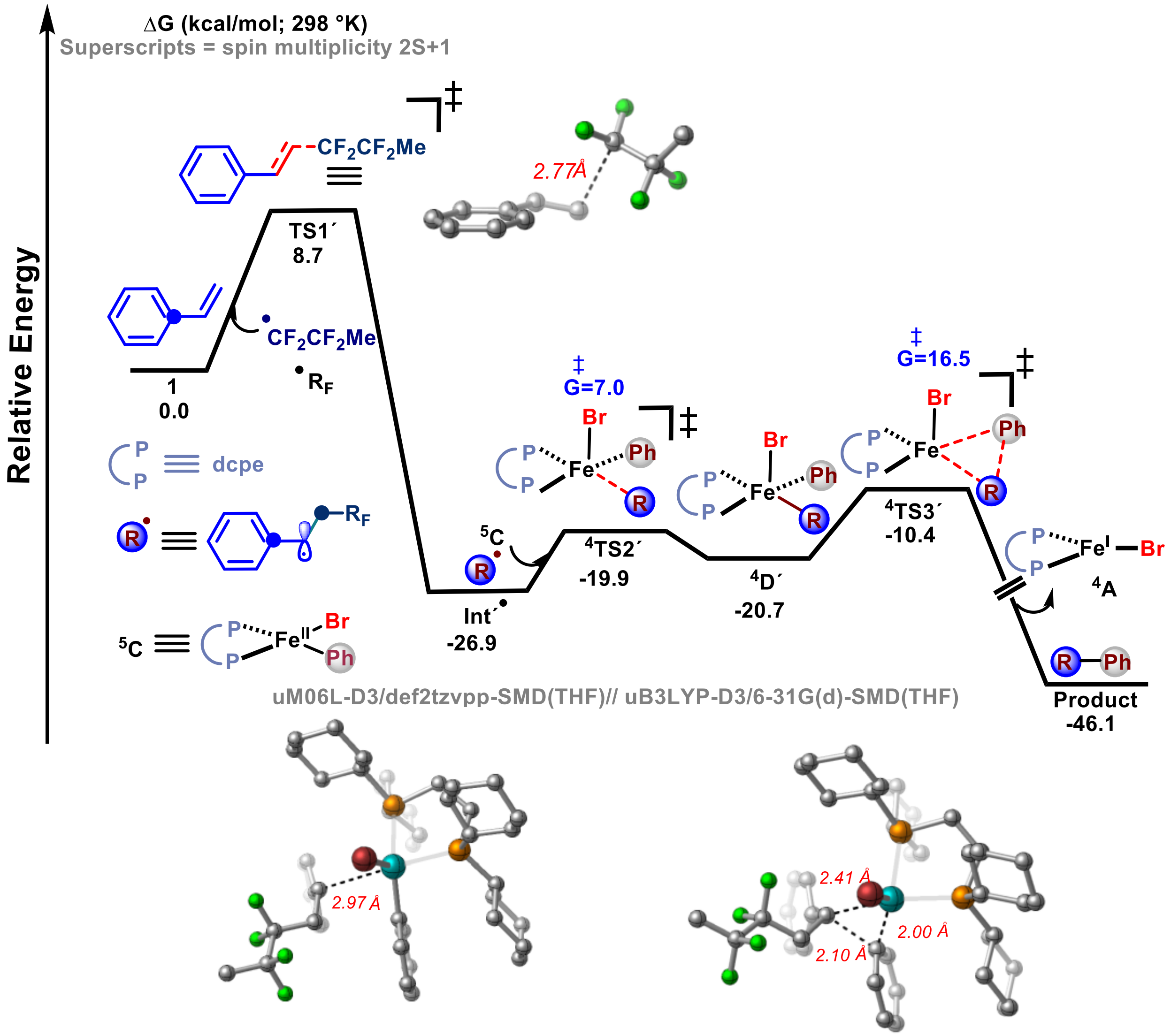

Figure S11. DFT calculation for dicarbofunctionalization of styrene. Calculated Gibbs free energies [uM06L-D3/def2tzvpp-SMD(THF)// uB3LYP-D3/6-31G(d)-SMD(THF)] are given in kcal mol^{-1}.

Noncovalent interaction (NCI) analysis, also known as reduced density gradient (RDG) method, was performed on Multiwfn to study the possible effect of noncovalent interaction in ^{4}**TS3.**[15] Extension distance of 0 Bohr, medium quality grid (totally about 512000 points) were set by default. Further visualization of the color-filled RDG isosurface was realized by VMD, where RDG isosurface and color range were set as 0.5, and -0.035 to 0.2, respectively.[16] Also, to investigate the bonding in ^{4}**TS3**, Natural Bonding Orbitals (NBO, version 3.1)[17] approaches were employed. Distances in structural figures are shown in Å and energies are in kcal/mol.

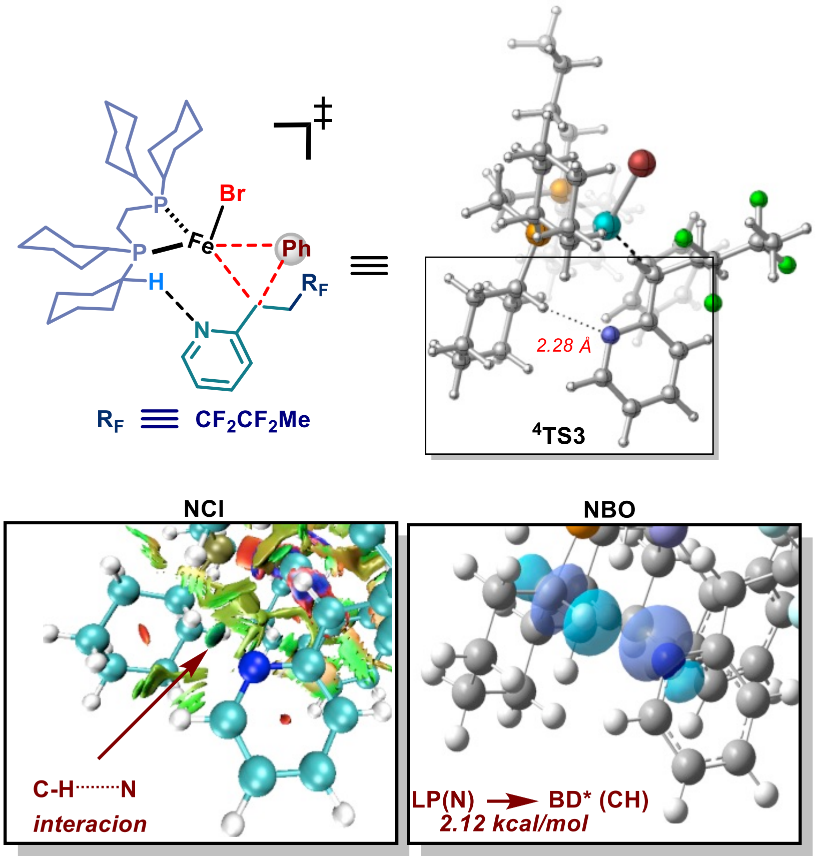

Figure S12. NBO and NCI analysis for **^{4}TS3** performed at uB3LYP-D3/6-31G(d)-SMD(THF) level of theory.

The proposed catalytic cycle following the computational methods is shown in **Figure S12**. Formation of the mono-halide Fe(I) **A**, will then undergo single electron halogen-atom abstraction to form 2• and dihalide Fe(II) **B** concurrently. The radical 2• can then escape the solvent cage to undergo radical addition to the vinyl azaarene **1**, which forms Int•. The slow addition of the Grignard reagent can selectively promote the mono-arylation of Fe(II) **B** to form monoaryl Fe(II) **C**. Next, Int• will undergo reversible radical addition to monoaryl Fe(II) **C** to form Fe(III) **D**. Then reductive elimination from Fe(III) **D** will yield the desired three-component product **4** and Fe(I) species **A** to restart the catalytic cycle.

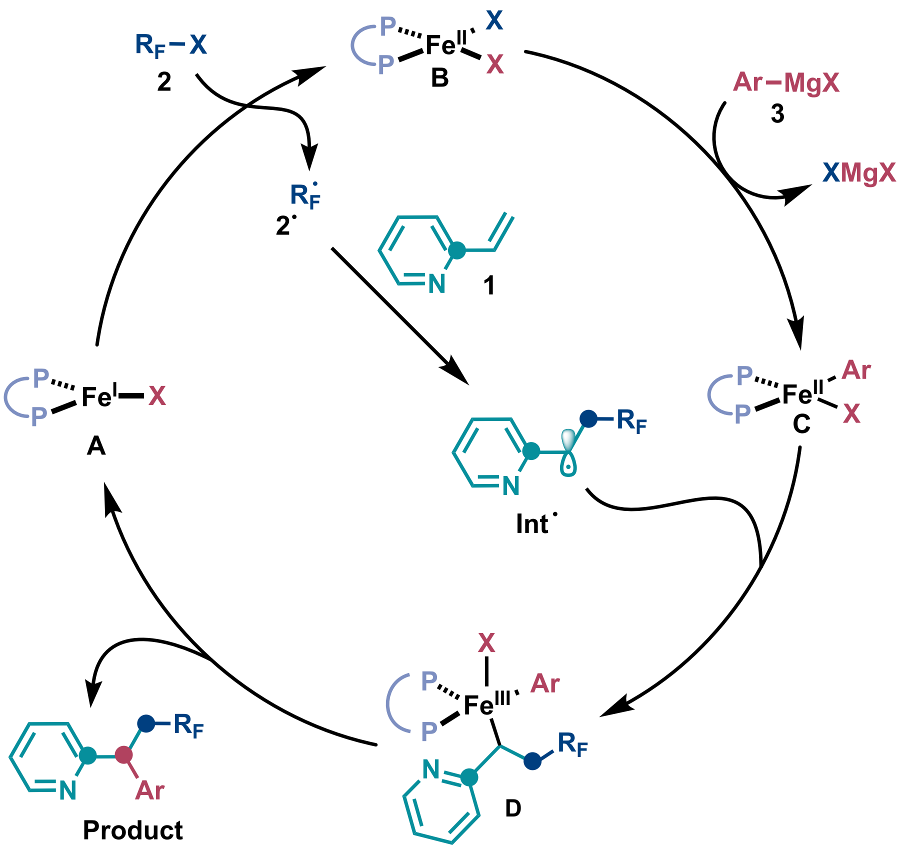

Figure S13. Proposed catalytic cycle.

Finally, to gain further insight on the origins of differences in energies for ^{4}**TS3** (vinyl pyridine **A** vs styrene **B**) were analyzed with regard to their distortion and interaction (**Table S7**).[18] Single-point energy calculations at the uM06L-D3/def2tzvpp-SMD(THF) level of theory were performed for: (i) the whole structure, (ii) just the **Int•**. (frag1) and (iii) just the ^{5}**C** fragment (i.e. the remaining fragment after removing the **Int•**; frag2). The contributions of distortion and interaction were then calculated as follows:

Potential energy surface (ΔE) = *E (**TS**) – [E (**Int•**) + E(^{5}**C**)]*
ΔE (distortion) = *[E (fragment 1) + E (fragment 2)] – [E (**Int•**) + E(^{5}**C**)]*
ΔE (interaction) = *ΔE – ΔE (distortion)*

Table S14. Single point energies used for the distortion/interaction analysis and resulting ΔE values. All energies in the table are in kcal/mol.

	E(^{4}TS3) a.u.	*E (Int•) a.u.*	*E (^{5}C) a.u.*	*E(frag1) a.u.*	*E(frag2) a.u.*	*ΔE*	*$ΔE_{dist}$*	*$ΔE_{int}$*
**A**	-6613.69181947	-841.407605102	-5772.27400301	-841.389627106	-5772.26546853	**-6.4**	**16.6**	**-23.0**
**B**	-6597.64256837	-825.363844733	-5772.27400301	-825.342086613	-5772.26484021	**-3.0**	**19.4**	**-22.4**

Table S15. Cartesian coordinates (xyz format) of all the structures involved in each reaction mechanism studied calculated at the uB3LYP-D3/6-31G(d)-SMD(THF) and single-point energies.

1 (*vinyl pyridine*)
E(scf) = -325.703694428 a.u.
ν_{min} = 72.95 cm^{-1}

C	10.040031	6.307673	10.839050
H	10.438939	7.319478	10.787366
C	9.675855	5.682930	9.712137
H	9.271361	4.673661	9.697851
H	9.774048	6.175015	8.748290
C	9.969080	5.777376	12.212990
C	9.486347	4.493349	12.524109
C	9.458162	4.081699	13.852013
H	9.139720	3.830193	11.738279
C	10.373488	6.210647	14.445007
C	9.911128	4.956119	14.842329
H	9.088480	3.093328	14.111525
H	10.735812	6.921773	15.185939
H	9.907377	4.676674	15.891386
N	10.407181	6.622838	13.171960

Zero-point correction= 0.122055 (Hartree/Particle)
Thermal correction to Energy= 0.128606
Thermal correction to Enthalpy= 0.129550
Thermal correction to Gibbs Free Energy= 0.091099
Sum of electronic and zero-point Energies= -325.581640
Sum of electronic and thermal Energies= -325.575088
Sum of electronic and thermal Enthalpies= -325.574144
Sum of electronic and thermal Free Energies= -325.612596

uM06L-D3/def2-TZVPP-SMD(THF).
E(scf) = -325.765195012 a.u.

1 (*styrene*)
E(scf) = -309.665782374 a.u.
ν_{min} = 55.01 cm^{-1}

C	10.039188	6.305254	10.794534
H	10.419576	7.322412	10.696388
C	9.690525	5.650827	9.678464
H	9.306074	4.633609	9.683724
H	9.782994	6.124704	8.704884
C	9.975929	5.808751	12.180258
C	9.479519	4.535252	12.520743
C	9.443880	4.114693	13.847822
H	9.116753	3.869248	11.743104
C	10.395679	6.219717	14.549725
C	9.901679	4.953941	14.870044
H	9.056095	3.127946	14.088031
H	10.753959	6.881181	15.334352
H	9.871688	4.622202	15.904478
C	10.430708	6.640311	13.219653
H	10.817325	7.627840	12.977279

Zero-point correction= 0.133848 (Hartree/Particle)
Thermal correction to Energy= 0.140559

Thermal correction to Enthalpy= 0.141503
Thermal correction to Gibbs Free Energy= 0.102610
Sum of electronic and zero-point Energies= -309.531934
Sum of electronic and thermal Energies= -309.525224
Sum of electronic and thermal Enthalpies= -309.524279
Sum of electronic and thermal Free Energies= -309.563173

uM06L-D3/def2-TZVPP-SMD(THF).
E(scf) = -309.720997403 a.u.

•CF_2CF_2Me
E(scf) = -515.425713893 a.u.
ν_{min} = 59.27 cm^{-1}

C	11.529973	5.043600	9.331642	H	12.979223	2.881174	8.286666
F	11.734315	4.222722	10.365264	H	12.516758	3.160038	6.582763
F	12.404151	6.053357	9.339931	H	13.503058	4.370353	7.453344
C	11.431569	4.331974	7.993394	F	11.060837	5.273752	7.067045
C	12.697093	3.638165	7.550179	F	10.407175	3.425544	8.097756

Zero-point correction= 0.060063 (Hartree/Particle)
Thermal correction to Energy= 0.067052
Thermal correction to Enthalpy= 0.067996
Thermal correction to Gibbs Free Energy= 0.027843
Sum of electronic and zero-point Energies= -515.365651
Sum of electronic and thermal Energies= -515.358662
Sum of electronic and thermal Enthalpies= -515.357718
Sum of electronic and thermal Free Energies= -515.397871

uM06L-D3/def2-TZVPP-SMD(THF).
E(scf) = -515.575402772 a.u.

TS1
E(scf) = -841.140229181 a.u.
ν_{min} = -37.96 cm^{-1}

C	9.860215	6.976229	11.121321	C	9.834951	4.740255	12.299636
H	10.095516	8.022336	11.309082	C	9.759277	4.033572	13.495088
C	9.661366	6.553348	9.858884	H	9.952548	4.216593	11.357973
H	9.337937	5.544136	9.624922	C	9.646296	6.133837	14.636437
H	9.731348	7.242985	9.022649	C	9.655753	4.739444	14.695450
C	12.038123	5.373480	9.296593	H	9.790980	2.947262	13.491649
F	12.509587	5.001348	10.492276	H	9.583069	6.720613	15.551778
F	12.899114	6.193947	8.682215	H	9.597462	4.228264	15.651477
C	9.795110	6.147229	12.333128	N	9.717810	6.831413	13.497184

C	11.607617	4.204766	8.429670	H	13.444300	3.848374	7.392041
C	12.717830	3.285630	7.983714	F	10.960971	4.734826	7.341396
H	13.214310	2.860284	8.859879	F	10.672196	3.490766	9.147798
H	12.291426	2.482406	7.375851				

Zero-point correction= 0.183068 (Hartree/Particle)
Thermal correction to Energy= 0.197594
Thermal correction to Enthalpy= 0.198538
Thermal correction to Gibbs Free Energy= 0.138687
Sum of electronic and zero-point Energies= -840.957161
Sum of electronic and thermal Energies= -840.942635
Sum of electronic and thermal Enthalpies= -840.941691
Sum of electronic and thermal Free Energies= -841.001542

uM06L-D3/def2-TZVPP-SMD(THF).
E(scf) = -841.346131422 a.u.

TS1´
E(scf) = -825.102381310 a.u.
ν_{min} = -21.49 cm^{-1}

C	9.925903	6.998447	11.052592	C	9.734541	4.755758	14.704577
H	10.191877	8.046781	11.189522	H	9.832712	2.995672	13.459028
C	9.695773	6.554277	9.803189	H	9.681313	6.686380	15.668313
H	9.354466	5.545244	9.595263	H	9.685164	4.195572	15.634587
H	9.774442	7.222100	8.950034	C	9.803433	6.866759	13.527026
C	12.081219	5.250193	9.243215	H	9.803858	7.954420	13.547085
F	12.550919	4.837886	10.426752	C	11.562711	4.118155	8.376241
F	12.983843	6.017012	8.617466	C	12.609643	3.148156	7.886421
C	9.864249	6.203928	12.286907	H	13.109012	2.686133	8.742064
C	9.881258	4.794373	12.285242	H	12.124735	2.376003	7.282173
C	9.813386	4.082457	13.480476	H	13.346879	3.680612	7.280161
H	9.978655	4.256113	11.348234	F	10.915072	4.695639	7.312357
C	9.733444	6.152444	14.722906	F	10.611583	3.445750	9.112544

Zero-point correction= 0.194836 (Hartree/Particle)
Thermal correction to Energy= 0.209562
Thermal correction to Enthalpy= 0.210506
Thermal correction to Gibbs Free Energy= 0.149872
Sum of electronic and zero-point Energies= -824.907545
Sum of electronic and thermal Energies= -824.892819
Sum of electronic and thermal Enthalpies= -824.891875
Sum of electronic and thermal Free Energies= -824.952510

uM06L-D3/def2-TZVPP-SMD(THF).
E(scf) = -825.301956636 a.u.

Int•
E(scf) = -841.202995575 a.u.
ν_{min} = 31.31 cm^{-1}

C	10.273859	6.528109	11.107758	C	9.714552	4.984071	14.953988
H	10.526610	7.583496	11.085306	H	9.418198	3.040192	14.049956
C	10.181474	5.797035	9.805738	H	10.077113	7.047467	15.488399
H	9.429728	5.001989	9.826402	H	9.578120	4.654691	15.979184
H	9.909610	6.486486	9.001140	N	10.175222	6.817164	13.449497
C	11.502067	5.150517	9.399220	C	11.468215	4.403659	8.051093
F	11.903124	4.241591	10.349929	C	12.765901	3.764436	7.635270
F	12.490528	6.099173	9.316939	H	13.073918	3.032310	8.384918
C	10.082273	5.951672	12.387048	H	12.617774	3.265416	6.673780
C	9.809920	4.568940	12.590023	H	13.539146	4.529464	7.536156
C	9.624968	4.093403	13.879815	F	11.056133	5.307783	7.099598
H	9.766120	3.891800	11.744929	F	10.477659	3.452411	8.146889
C	9.994968	6.330314	14.672016				

Zero-point correction= 0.185933 (Hartree/Particle)
Thermal correction to Energy= 0.199708
Thermal correction to Enthalpy= 0.200652
Thermal correction to Gibbs Free Energy= 0.143586
Sum of electronic and zero-point Energies= -841.017063
Sum of electronic and thermal Energies= -841.003288
Sum of electronic and thermal Enthalpies= -841.002343
Sum of electronic and thermal Free Energies= -841.059409

uM06L-D3/def2-TZVPP-SMD(THF).
E(scf) = -841.407605102 a.u.

Int´•
E(scf) = -825.165558282 a.u.
ν_{min} = 24.56 cm^{-1}

C	10.317087	6.520244	11.042066	H	9.777514	3.912034	11.735236
H	10.593813	7.568549	10.969038	C	10.053729	6.335318	14.761223
C	10.186869	5.762808	9.756610	C	9.755999	4.979572	14.966536
H	9.425165	4.978677	9.810286	H	9.431262	3.068565	14.014762
H	9.905423	6.437590	8.942432	H	10.139237	7.006841	15.611912
C	11.487777	5.089895	9.332574	H	9.610593	4.596640	15.972801
F	11.899455	4.193353	10.290356	C	10.240087	6.825602	13.477710
F	12.488168	6.023313	9.209520	H	10.468930	7.877895	13.325498
C	10.133783	5.975920	12.335229	C	11.415994	4.319450	7.999410
C	9.838419	4.599846	12.571792	C	12.693806	3.649422	7.570545
C	9.653213	4.121844	13.861758	H	13.001044	2.922717	8.325773

H	12.519594	3.138979	6.619435	F	11.002315	5.213718	7.038584
H	13.479651	4.397852	7.446620	F	10.410924	3.387644	8.129528

Zero-point correction= 0.197629 (Hartree/Particle)
Thermal correction to Energy= 0.211564
Thermal correction to Enthalpy= 0.212508
Thermal correction to Gibbs Free Energy= 0.155033
Sum of electronic and zero-point Energies= -824.967930
Sum of electronic and thermal Energies= -824.953995
Sum of electronic and thermal Enthalpies= -824.953051
Sum of electronic and thermal Free Energies= -825.010525

uM06L-D3/def2-TZVPP-SMD(THF).
E(scf) = -825.363844733 a.u.

^{5}C
E(scf) = -5769.66437627 a.u.
ν_{min} = 20.58 cm^{-1}

Br	9.994415	5.235261	10.360099	H	6.059993	6.977340	11.165912
Fe	9.162707	6.347279	12.319773	C	8.732867	9.038844	9.883573
P	8.401291	8.578540	11.660062	H	8.290941	8.208985	9.314078
P	10.907632	7.605783	13.494380	C	8.099631	10.358079	9.403284
C	9.313116	9.846515	12.686631	H	7.015777	10.353133	9.564563
H	9.336841	10.811592	12.167270	H	8.506347	11.193644	9.991072
H	8.719961	9.992593	13.593361	C	8.394151	10.594458	7.911418
C	10.748397	9.418505	13.069701	H	7.891216	9.812854	7.323304
H	11.113395	10.037247	13.897574	H	7.963250	11.553665	7.595891
H	11.423183	9.593907	12.227604	C	9.899531	10.559608	7.612690
C	6.594124	8.924378	11.949833	H	10.386097	11.409902	8.114076
H	6.386274	9.977974	11.717570	H	10.075647	10.684557	6.536473
C	6.218840	8.661004	13.424019	C	10.534166	9.254205	8.112508
H	6.519197	7.641155	13.696244	H	11.617972	9.263392	7.937902
H	6.765698	9.338232	14.089868	H	10.128279	8.405786	7.542763
C	4.708909	8.816889	13.660972	C	10.250424	9.036099	9.605650
H	4.478371	8.593737	14.710628	H	10.723244	9.849301	10.173152
H	4.420011	9.864161	13.487364	H	10.698010	8.096437	9.944062
C	3.898995	7.906857	12.728618	C	12.700014	7.201039	13.188224
H	2.823421	8.051889	12.892485	H	13.323674	7.917077	13.742110
H	4.118829	6.856553	12.970080	C	13.040463	7.307725	11.685687
C	4.255456	8.173746	11.260680	H	12.362445	6.656301	11.120227
H	3.945825	9.194950	10.993111	H	12.878079	8.329700	11.323565
H	3.703058	7.492554	10.600827	C	14.492350	6.888543	11.408396
C	5.763492	8.025138	11.008023	H	14.693145	6.953675	10.331139
H	5.986492	8.257093	9.960145	H	15.177871	7.593156	11.902854

C	14.768693	5.468402	11.919176	H	9.917975	9.441838	18.001490
H	14.147658	4.756928	11.355448	C	8.990461	7.650439	17.226967
H	15.815394	5.192276	11.737009	H	9.027048	6.565703	17.403590
C	14.438504	5.351852	13.412651	H	7.978243	7.980453	17.494341
H	15.123847	5.993215	13.986729	C	9.248323	7.917712	15.737104
H	14.599337	4.324031	13.762671	H	9.117369	8.990395	15.538912
C	12.991055	5.775636	13.706336	H	8.513690	7.381041	15.127273
H	12.802621	5.711016	14.784346	C	8.192626	5.099161	13.613936
H	12.300182	5.072439	13.217970	C	8.960100	4.357327	14.540687
C	10.676965	7.491035	15.342464	H	10.045672	4.457050	14.531684
H	10.754924	6.413854	15.541592	C	8.384097	3.493573	15.478591
C	11.723566	8.214595	16.207845	C	6.995603	3.333579	15.517838
H	11.680561	9.295378	16.012241	H	6.539249	2.667442	16.246784
H	12.736356	7.883287	15.950816	C	6.201525	4.033225	14.605309
C	11.461483	7.958250	17.702611	H	5.119794	3.909400	14.619894
H	11.613537	6.888890	17.910613	C	6.797222	4.893843	13.675598
H	12.195778	8.505364	18.308177	H	6.146732	5.423184	12.979577
C	10.033878	8.352632	18.107391	H	9.013809	2.945359	16.177551
H	9.861545	8.118159	19.165796				

Zero-point correction= 0.799775 (Hartree/Particle)
Thermal correction to Energy= 0.838827
Thermal correction to Enthalpy= 0.839771
Thermal correction to Gibbs Free Energy= 0.725198
Sum of electronic and zero-point Energies= -5768.864601
Sum of electronic and thermal Energies= -5768.825549
Sum of electronic and thermal Enthalpies= -5768.824605
Sum of electronic and thermal Free Energies= -5768.939178

uM06L-D3/def2-TZVPP-SMD(THF).
E(scf) = -5772.27400301 a.u.

^{4}TS2
E(scf) = -6610.89290228 a.u.
ν_{min} = -65.07 cm^{-1}

Br	10.635670	8.605879	7.745516	H	11.311249	12.772575	9.868879
Fe	9.554183	9.192387	9.842714	C	6.631778	11.527545	9.481191
P	8.474513	11.362127	9.242533	H	6.354713	12.571871	9.278284
P	11.111036	10.668891	11.049759	C	6.220216	11.188712	10.929535
C	9.193977	12.691360	10.335192	H	6.574142	10.181374	11.175070
H	9.075895	13.674314	9.863525	H	6.693034	11.878010	11.639154
H	8.587371	12.703953	11.244186	C	4.694808	11.236753	11.107942
C	10.675058	12.453834	10.696953	H	4.438583	10.959526	12.138851
H	10.953298	13.071123	11.559040	H	4.342262	12.268559	10.959225

C	3.985238	10.310242	10.111959
H	2.896649	10.375077	10.239056
H	4.272221	9.269521	10.320866
C	4.377522	10.659461	8.670531
H	4.013283	11.669284	8.428126
H	3.895767	9.970567	7.964593
C	5.900472	10.608136	8.479187
H	6.154334	10.879052	7.447847
H	6.243513	9.577880	8.629981
C	8.795776	11.944252	7.492899
H	8.539411	11.068415	6.880404
C	7.959309	13.147396	7.014058
H	6.887110	12.960751	7.132154
H	8.199664	14.025403	7.631378
C	8.255693	13.463219	5.536819
H	7.917191	12.616643	4.921540
H	7.670728	14.336887	5.220571
C	9.751084	13.701867	5.286437
H	10.061303	14.620056	5.807740
H	9.935106	13.868237	4.217049
C	10.592967	12.526704	5.801690
H	11.663077	12.735751	5.672288
H	10.372530	11.625673	5.211384
C	10.295222	12.240165	7.280074
H	10.572856	13.125459	7.868495
H	10.905902	11.405764	7.631697
C	12.951769	10.605781	10.743540
H	13.414984	11.371270	11.382484
C	13.316812	10.915402	9.275650
H	12.809482	10.195651	8.622231
H	12.966162	11.912089	8.985503
C	14.835513	10.828028	9.053184
H	15.062613	11.027594	7.997844
H	15.334557	11.614317	9.639421
C	15.388544	9.458370	9.468997
H	14.959294	8.685542	8.814256
H	16.477061	9.426535	9.330541
C	15.021266	9.139353	10.924022
H	15.523268	9.853267	11.594153
H	15.379003	8.139113	11.200190
C	13.503296	9.221123	11.139191
H	13.251737	8.972677	12.175572
H	13.020964	8.464640	10.507004
C	10.903440	10.487699	12.892556
H	11.082944	9.418436	13.056413
C	11.868654	11.285150	13.786459
H	11.733785	12.362827	13.614496
H	12.910114	11.049126	13.540308
C	11.612870	10.971283	15.272075
H	11.866174	9.917587	15.458989
H	12.283390	11.572764	15.899672
C	10.149767	11.215319	15.669226
H	9.990829	10.937475	16.719221
H	9.929883	12.290618	15.589622
C	9.186421	10.440142	14.758556
H	9.324822	9.360293	14.910063
H	8.144846	10.667758	15.020254
C	9.439974	10.779361	13.282957
H	9.224815	11.845267	13.127410
H	8.751405	10.221648	12.637111
C	7.987048	7.975559	10.325426
C	7.427447	7.796502	11.605727
H	7.915863	8.238308	12.473405
C	6.261005	7.053326	11.820627
C	5.612335	6.444563	10.742664
H	4.707491	5.862232	10.901420
C	6.147575	6.585911	9.457736
H	5.657073	6.111364	8.609577
C	7.310658	7.337846	9.262536
H	7.704877	7.429394	8.250779
H	5.864392	6.942779	12.828446
C	10.629083	6.570397	10.814731
H	11.457966	7.222605	10.570661
C	10.128737	5.671389	9.729432
H	9.125907	5.288842	9.925979
H	10.089954	6.220907	8.787125
C	11.048360	4.483011	9.507716
F	11.165288	3.728793	10.654389
F	12.316538	4.913176	9.191466
C	10.386420	6.346045	12.203496
C	9.449222	5.389939	12.678823
C	9.256446	5.236986	14.043513
H	8.887322	4.783697	11.978804
C	10.903413	6.941399	14.386128
C	9.996031	6.025601	14.930859
H	8.537455	4.511541	14.414838
H	11.497338	7.575670	15.043118
H	9.879018	5.938338	16.006448
N	11.101942	7.112505	13.080401
C	10.599648	3.523392	8.388767

C	11.515047	2.355739	8.132916	H	12.501999	2.718819	7.837937
H	11.606388	1.753164	9.039350	F	10.454870	4.272563	7.244545
H	11.092180	1.747625	7.328661	F	9.342322	3.074820	8.728156

Zero-point correction= 0.987407 (Hartree/Particle)
Thermal correction to Energy= 1.041263
Thermal correction to Enthalpy= 1.042207
Thermal correction to Gibbs Free Energy= 0.894082
Sum of electronic and zero-point Energies= -6609.905495
Sum of electronic and thermal Energies= -6609.851640
Sum of electronic and thermal Enthalpies= -6609.850695
Sum of electronic and thermal Free Energies= -6609.998821

uM06L-D3/def2-TZVPP-SMD(THF).
E(scf) = -6613.70096127 a.u.

^{4}TS2´
E(scf) = -6594.85152060 a.u.
ν_{min} = -73.19 cm^{-1}

Br	10.733540	8.573696	7.762920	H	6.196804	10.855814	7.348592
Fe	9.608509	9.143523	9.843281	H	6.239698	9.533307	8.508947
P	8.468344	11.309393	9.218853	C	8.818606	11.930970	7.486987
P	11.110797	10.679808	11.061217	H	8.566104	11.070347	6.851236
C	9.120910	12.637357	10.356042	C	7.995761	13.149073	7.022277
H	8.972679	13.627176	9.907714	H	6.920839	12.963656	7.114034
H	8.497311	12.602160	11.253226	H	8.226640	14.010154	7.666359
C	10.600593	12.451468	10.745375	C	8.321330	13.503621	5.560098
H	10.834097	13.054984	11.630298	H	7.992858	12.675213	4.915334
H	11.243044	12.819081	9.942525	H	7.744599	14.387300	5.256737
C	6.615835	11.464717	9.406268	C	9.821792	13.744345	5.345690
H	6.350118	12.513539	9.210862	H	10.125044	14.644960	5.900718
C	6.153493	11.112664	10.835701	H	10.027167	13.942361	4.285614
H	6.491941	10.101691	11.085184	C	10.647992	12.549927	5.841014
H	6.605597	11.792749	11.567338	H	11.721541	12.755988	5.737403
C	4.623240	11.172056	10.963314	H	10.433253	11.668730	5.219370
H	4.330202	10.884793	11.981622	C	10.322353	12.220109	7.304457
H	4.284470	12.208671	10.815557	H	10.600227	13.082324	7.925843
C	3.939281	10.264420	9.932913	H	10.916678	11.367087	7.637105
H	2.847616	10.336700	10.025115	C	12.953515	10.736287	10.753182
H	4.211056	9.218822	10.137544	H	13.358083	11.557038	11.363192
C	4.381406	10.629597	8.510039	C	13.279983	11.020270	9.270868
H	4.033185	11.645745	8.270301	H	12.808207	10.250579	8.648833
H	3.917169	9.954821	7.779099	H	12.862669	11.983064	8.956903
C	5.909468	10.568544	8.366638	C	14.795957	11.017700	9.019491

H	14.988813	11.199578	7.954294
H	15.260740	11.847444	9.573025
C	15.432942	9.693995	9.460867
H	15.027613	8.878091	8.844250
H	16.517559	9.713640	9.293620
C	15.122607	9.410357	10.935498
H	15.600620	10.177480	11.562779
H	15.544181	8.443718	11.239961
C	13.609441	9.415788	11.197512
H	13.419438	9.233033	12.260373
H	13.148212	8.590740	10.638405
C	10.899856	10.450395	12.898454
H	11.115593	9.385907	13.051555
C	11.819959	11.261772	13.826715
H	11.625702	12.335849	13.695172
H	12.874047	11.095173	13.577749
C	11.571354	10.871825	15.295591
H	11.869214	9.822695	15.436724
H	12.210681	11.474244	15.953957
C	10.096218	11.032533	15.692271
H	9.944477	10.697525	16.726435
H	9.830060	12.099850	15.662826
C	9.171053	10.260875	14.739366
H	9.351418	9.181427	14.843431
H	8.119549	10.435933	15.001369
C	9.422562	10.674514	13.282649
H	9.174994	11.738765	13.168755
H	8.759327	10.123066	12.606281
C	7.992731	7.981730	10.298406
C	7.414088	7.783865	11.566967
H	7.891923	8.206177	12.449320
C	6.239329	7.044588	11.753608
C	5.601398	6.458530	10.657473
H	4.691354	5.878695	10.794153
C	6.153346	6.621054	9.381837
H	5.671010	6.165204	8.518896
C	7.321810	7.370700	9.215763
H	7.726911	7.479068	8.209731
H	5.828595	6.919839	12.754148
C	10.744807	6.569554	10.788455
H	11.631903	7.087627	10.437986
C	10.082371	5.679355	9.781416
H	9.068116	5.397634	10.065778
H	10.022778	6.187108	8.817466
C	10.865314	4.396720	9.559027
F	10.961951	3.669571	10.724166
F	12.154979	4.680205	9.167785
C	10.612578	6.385178	12.202903
C	9.525430	5.684613	12.792419
C	9.416550	5.557207	14.172225
H	8.755725	5.258690	12.159889
C	11.486290	6.770796	14.463109
C	10.388964	6.102008	15.021510
H	8.565799	5.026986	14.593185
H	12.260789	7.179606	15.107504
H	10.298933	5.996869	16.099199
C	10.266472	3.457259	8.493953
C	11.032912	2.184508	8.249439
H	11.086644	1.603490	9.172747
H	10.515638	1.604529	7.480341
H	12.043948	2.421960	7.911228
F	10.162681	4.179793	7.327794
F	8.980725	3.165975	8.892174
C	11.595155	6.910268	13.084749
H	12.457901	7.412221	12.661460

Zero-point correction= 0.999955 (Hartree/Particle)
Thermal correction to Energy= 1.053787
Thermal correction to Enthalpy= 1.054731
Thermal correction to Gibbs Free Energy= 0.907439
Sum of electronic and zero-point Energies= -6593.851566
Sum of electronic and thermal Energies= -6593.797734
Sum of electronic and thermal Enthalpies= -6593.796790
Sum of electronic and thermal Free Energies= -6593.944081

uM06L-D3/def2-TZVPP-SMD(THF).

E(scf) = -6597.65389010 a.u.

^{4}D
E(scf) = -6610.90903048 a.u.
ν_{min} = 17.03 cm^{-1}

Br	10.797392	8.545838	7.636425	H	11.359469	12.500895	5.474562
Fe	9.766770	8.679114	9.890644	H	10.106290	11.295418	5.182743
P	8.701798	10.933437	9.428961	C	10.233619	11.941716	7.238237
P	11.406815	10.078796	11.128237	H	10.505397	12.857465	7.781021
C	9.691833	12.209014	10.368236	H	10.950007	11.166365	7.510131
H	9.592114	13.186466	9.881971	C	13.273432	9.928085	10.972684
H	9.253452	12.306334	11.364777	H	13.656475	10.946399	11.139939
C	11.169919	11.811761	10.489988	C	13.650418	9.496952	9.540672
H	11.707009	12.520351	11.129751	H	13.241294	8.497703	9.351336
H	11.660070	11.834503	9.512799	H	13.190358	10.158684	8.798692
C	6.932503	11.317525	9.922212	C	15.172606	9.457991	9.347506
H	6.787776	12.377394	9.670325	H	15.403693	9.131515	8.325225
C	6.655870	11.168306	11.430959	H	15.584562	10.472499	9.458923
H	6.840245	10.132921	11.737524	C	15.831649	8.526096	10.372139
H	7.329417	11.803647	12.015474	H	15.484718	7.497273	10.195291
C	5.202831	11.548598	11.764742	H	16.922178	8.521973	10.245479
H	5.024526	11.401258	12.837960	C	15.461258	8.943219	11.801193
H	5.059274	12.620983	11.564647	H	15.889584	9.935528	12.009145
C	4.193248	10.742436	10.937280	H	15.902364	8.250485	12.529804
H	3.169364	11.063298	11.169201	C	13.938072	8.997406	12.005569
H	4.266657	9.680231	11.211480	H	13.725848	9.340365	13.024809
C	4.477293	10.886727	9.436467	H	13.511300	7.992099	11.918260
H	4.310248	11.930185	9.129178	C	11.059637	10.168515	12.962633
H	3.781839	10.267895	8.854983	H	11.627166	9.323904	13.362917
C	5.921167	10.476323	9.113134	C	11.544922	11.451861	13.658086
H	6.105830	10.559959	8.036705	H	10.997389	12.318576	13.262682
H	6.050312	9.423460	9.367954	H	12.608533	11.623551	13.449354
C	8.798862	11.526126	7.636072	C	11.306357	11.369563	15.174977
H	8.531301	10.633201	7.055770	H	11.918388	10.556454	15.592334
C	7.839013	12.676241	7.255879	H	11.640815	12.297799	15.656215
H	6.800748	12.441235	7.499876	C	9.827126	11.105030	15.491440
H	8.105391	13.575981	7.829751	H	9.677785	11.025667	16.575930
C	7.924552	12.985863	5.750097	H	9.228532	11.963656	15.151297
H	7.570309	12.107261	5.191206	C	9.329317	9.833253	14.790706
H	7.241281	13.810405	5.507498	H	9.853252	8.960406	15.203826
C	9.352559	13.324340	5.306897	H	8.259507	9.680295	14.984477
H	9.658769	14.273746	5.771453	C	9.577329	9.892133	13.276024
H	9.388656	13.477380	4.220366	H	8.954592	10.680492	12.842311
C	10.329150	12.221543	5.731535	H	9.270014	8.948403	12.811405

C	7.979985	7.779794	9.839412	F	12.480263	5.168221	9.096015
C	7.163636	7.594258	10.965648	C	10.360499	6.480882	11.895232
H	7.515083	7.898216	11.948815	C	9.275338	5.717082	12.375304
C	5.893145	7.013585	10.864769	C	9.166163	5.447215	13.734273
C	5.413680	6.592318	9.622322	H	8.526254	5.345600	11.687541
H	4.427962	6.141253	9.537619	C	11.185790	6.672843	14.054574
C	6.218478	6.751883	8.489258	C	10.143177	5.931636	14.607787
H	5.858592	6.423177	7.516200	H	8.330275	4.860852	14.107784
C	7.482240	7.338529	8.598992	H	11.974162	7.073148	14.691616
H	8.091334	7.460703	7.707029	H	10.102873	5.740100	15.675723
H	5.282873	6.888662	11.757196	N	11.297718	6.947206	12.750191
C	10.546321	6.822510	10.460860	C	10.981816	3.504460	8.402562
H	11.595280	7.103766	10.324214	C	12.034980	2.431070	8.323282
C	10.193571	5.708045	9.478125	H	12.140294	1.945193	9.295801
H	9.240356	5.228784	9.705399	H	11.731098	1.693578	7.575439
H	10.114272	6.110701	8.468354	H	12.990795	2.873645	8.034485
C	11.252991	4.628130	9.422040	F	10.821829	4.100588	7.172138
F	11.416321	4.016909	10.648204	F	9.764696	2.947622	8.730021

Zero-point correction= 0.991592 (Hartree/Particle)
Thermal correction to Energy= 1.044721
Thermal correction to Enthalpy= 1.045665
Thermal correction to Gibbs Free Energy= 0.902399
Sum of electronic and zero-point Energies= -6609.917439
Sum of electronic and thermal Energies= -6609.864310
Sum of electronic and thermal Enthalpies= -6609.863366
Sum of electronic and thermal Free Energies= -6610.006631

uM06L-D3/def2-TZVPP-SMD(THF).
E(scf) = -6613.71217384 a.u.

^{4}D′
E(scf) = -6594.86533098 a.u.
ν_{min} = 17.73 cm^{-1}

Br	11.300516	8.503108	8.002353	C	6.967910	11.215267	10.004935
Fe	9.818472	8.623938	10.012879	H	6.816573	12.289675	9.828307
P	8.718699	10.856027	9.432322	C	6.773833	10.968054	11.513494
P	11.479371	10.120114	11.168633	H	7.008212	9.923847	11.748986
C	9.722104	12.169337	10.301503	H	7.462101	11.590109	12.093696
H	9.606180	13.127015	9.780444	C	5.334441	11.282694	11.954397
H	9.313362	12.302867	11.306731	H	5.222587	11.055895	13.022681
C	11.206362	11.781084	10.374178	H	5.153655	12.361954	11.839425
H	11.785146	12.555035	10.889385	C	4.299252	10.509780	11.127964
H	11.615501	11.691576	9.365481	H	3.282757	10.779735	11.442515

H	4.417459	9.432662	11.313597
C	4.489910	10.780396	9.630266
H	4.284576	11.841185	9.420842
H	3.772413	10.194768	9.041115
C	5.918061	10.432150	9.185631
H	6.030850	10.624783	8.113923
H	6.076265	9.362618	9.326328
C	8.707374	11.425330	7.630855
H	8.320869	10.551972	7.086180
C	7.803407	12.643632	7.331350
H	6.777883	12.490226	7.673870
H	8.191244	13.518678	7.873046
C	7.772981	12.950424	5.823197
H	7.306198	12.102000	5.301386
H	7.132507	13.823385	5.640725
C	9.176244	13.185389	5.252297
H	9.592366	14.107039	5.686281
H	9.125962	13.341210	4.166710
C	10.102267	12.011297	5.589363
H	11.122771	12.213355	5.238300
H	9.760415	11.108270	5.062593
C	10.125601	11.729913	7.099293
H	10.518836	12.618018	7.613630
H	10.797900	10.897468	7.306148
C	13.344696	9.869628	11.040102
H	13.763442	10.382237	11.918661
C	13.984267	10.497165	9.783273
H	13.526594	10.055075	8.890435
H	13.791628	11.574884	9.750991
C	15.504391	10.263650	9.755003
H	15.920897	10.693950	8.834873
H	15.968456	10.803349	10.594465
C	15.857112	8.774939	9.859026
H	15.472145	8.248742	8.973331
H	16.946445	8.639152	9.863391
C	15.232884	8.159931	11.117162
H	15.681533	8.620344	12.010366
H	15.449725	7.085235	11.169756
C	13.712492	8.374602	11.138256
H	13.294976	7.933477	12.050154
H	13.269405	7.842372	10.288326
C	11.246679	10.392417	13.007623
H	11.874886	9.612572	13.461232
C	11.736106	11.753806	13.536984
H	11.125915	12.555236	13.097698

H	12.773729	11.942245	13.235138
C	11.611859	11.818313	15.068724
H	12.282662	11.069723	15.515267
H	11.948275	12.799579	15.427861
C	10.171219	11.546799	15.526128
H	10.111689	11.557963	16.622026
H	9.521695	12.358521	15.165460
C	9.655386	10.209349	14.976596
H	10.227279	9.384070	15.423448
H	8.606467	10.055575	15.261265
C	9.793010	10.142282	13.449108
H	9.147805	10.905354	13.002271
H	9.441419	9.171731	13.082470
C	8.058343	7.738835	9.696057
C	7.115774	7.447332	10.692371
H	7.340314	7.666768	11.732726
C	5.881686	6.862605	10.383966
C	5.565895	6.553099	9.058751
H	4.608362	6.100073	8.813459
C	6.496436	6.827894	8.051002
H	6.263082	6.588648	7.015315
C	7.726018	7.412912	8.368134
H	8.437346	7.619899	7.571340
H	5.170664	6.648761	11.179554
C	10.418282	6.747531	10.736631
H	11.472307	7.027298	10.763825
C	10.247743	5.649581	9.687587
H	9.280575	5.148859	9.751291
H	10.320704	6.070488	8.684423
C	11.327377	4.591209	9.779169
F	11.303641	3.946183	10.997261
F	12.579237	5.165738	9.675307
C	9.994859	6.384743	12.120101
C	8.888924	5.557149	12.402627
C	8.521473	5.252532	13.713982
H	8.304046	5.145305	11.588351
C	10.366459	6.558791	14.538105
C	9.252999	5.753336	14.793812
H	7.659613	4.613012	13.890721
H	10.964038	6.943658	15.361050
H	8.968448	5.511049	15.814428
C	10.722846	6.865636	13.226301
H	11.591571	7.487682	13.043313
C	11.258906	3.493856	8.698998
C	12.335393	2.443111	8.771993

H	12.285851	1.930554	9.735176	F	11.295819	4.122110	7.474827
H	12.180468	1.722513	7.964390	F	10.017426	2.904843	8.800724
H	13.315591	2.911996	8.661215				

Zero-point correction= 1.003366 (Hartree/Particle)
Thermal correction to Energy= 1.056679
Thermal correction to Enthalpy= 1.057623
Thermal correction to Gibbs Free Energy= 0.913731
Sum of electronic and zero-point Energies= -6593.861965
Sum of electronic and thermal Energies= -6593.808652
Sum of electronic and thermal Enthalpies= -6593.807708
Sum of electronic and thermal Free Energies= -6593.951600

uM06L-D3/def2-TZVPP-SMD(THF).
E(scf) = -6597.66140116 a.u.

^{4}TS3
E(scf) = -6610.88723741 a.u.
ν_{min} = -274.12 cm^{-1}

Br	10.757040	8.402061	7.752620	H	6.218320	10.441942	7.712834
Fe	9.648443	8.873100	9.885853	H	6.235118	9.300163	9.051152
P	8.581442	11.055211	9.351305	C	8.870048	11.610316	7.581574
P	11.220537	10.303208	11.143644	H	8.663060	10.705576	6.993779
C	9.392298	12.375005	10.395044	C	7.972656	12.752779	7.066716
H	9.296450	13.352662	9.907266	H	6.911666	12.511887	7.186775
H	8.820884	12.431667	11.323736	H	8.162474	13.660709	7.658096
C	10.875621	12.087617	10.713356	C	8.253426	13.039337	5.580285
H	11.215927	12.737460	11.528243	H	7.959294	12.157856	4.991785
H	11.489849	12.326529	9.843418	H	7.623366	13.871184	5.238663
C	6.757263	11.307990	9.650028	C	9.733975	13.347233	5.318966
H	6.505881	12.337343	9.358367	H	9.996693	14.296413	5.810079
C	6.371762	11.123436	11.131613	H	9.908395	13.488771	4.244347
H	6.667373	10.118709	11.456284	C	10.634945	12.233997	5.870163
H	6.904672	11.837895	11.768865	H	11.692921	12.493712	5.732452
C	4.857101	11.299163	11.332090	H	10.461497	11.303793	5.310572
H	4.604879	11.134805	12.388014	C	10.351982	11.982115	7.357423
H	4.583070	12.339219	11.099658	H	10.577724	12.902402	7.913851
C	4.048999	10.349385	10.437238	H	11.010739	11.198220	7.737840
H	2.973517	10.528673	10.566424	C	13.053734	10.083745	10.853506
H	4.242179	9.313400	10.746866	H	13.592098	10.741255	11.549897
C	4.444690	10.505699	8.962873	C	13.479611	10.453392	9.416799
H	4.151213	11.505033	8.607645	H	12.887124	9.867862	8.704657
H	3.903899	9.777407	8.344610	H	13.278366	11.511374	9.212749
C	5.957600	10.323227	8.771363	C	14.973394	10.170196	9.187506

H	15.236268	10.415506	8.150137	C	5.506333	6.712391	10.496172
H	15.573615	10.830669	9.831599	H	4.466242	6.403075	10.554041
C	15.324515	8.708777	9.496896	C	6.230685	6.556473	9.307806
H	14.785906	8.053683	8.796246	H	5.748495	6.132904	8.428988
H	16.396905	8.531193	9.342887	C	7.567407	6.946014	9.232446
C	14.917664	8.344021	10.930327	H	8.101407	6.824867	8.294660
H	15.514192	8.933346	11.642851	H	5.603808	7.389418	12.546300
H	15.131396	7.286468	11.132760	C	10.109135	6.620586	10.721041
C	13.424535	8.615956	11.158938	H	10.998151	7.244540	10.655407
H	13.138718	8.341884	12.180973	C	10.167381	5.536694	9.662095
H	12.854078	7.963759	10.486846	H	9.354124	4.813256	9.746577
C	11.009036	10.205417	12.994460	H	10.109139	5.987843	8.671043
H	11.129857	9.133762	13.190734	C	11.480267	4.776591	9.696095
C	12.015417	10.981317	13.861081	F	11.664600	4.139267	10.903746
H	11.930869	12.059493	13.661039	F	12.545178	5.639809	9.550255
H	13.043655	10.690440	13.618011	C	9.952609	6.189356	12.124829
C	11.754648	10.716816	15.355542	C	9.135274	5.102659	12.498088
H	11.955455	9.655663	15.565429	C	9.011296	4.765695	13.839617
H	12.459065	11.297472	15.965483	H	8.599359	4.539894	11.741991
C	10.308548	11.043568	15.755044	C	10.471126	6.585452	14.346626
H	10.143159	10.801102	16.812848	C	9.696296	5.518505	14.797976
H	10.141740	12.126081	15.648761	H	8.384434	3.928897	14.136824
C	9.302370	10.294100	14.869933	H	11.017342	7.208587	15.053724
H	9.386319	9.213257	15.050295	H	9.629868	5.290937	15.857407
H	8.275088	10.581385	15.130081	N	10.599840	6.920756	13.056898
C	9.564222	10.578631	13.384166	C	11.628120	3.699004	8.603349
H	9.399198	11.648967	13.201360	C	12.938666	2.957810	8.600812
H	8.844452	10.037415	12.758347	H	13.079367	2.453075	9.559151
C	8.237062	7.516437	10.347509	H	12.922842	2.218822	7.795091
C	7.487445	7.641082	11.544620	H	13.758472	3.660692	8.437403
H	7.971897	8.020766	12.440278	F	11.429810	4.314445	7.390208
C	6.148075	7.262569	11.612642	F	10.584201	2.815066	8.770917

Zero-point correction= 0.989168 (Hartree/Particle)
Thermal correction to Energy= 1.042340
Thermal correction to Enthalpy= 1.043284
Thermal correction to Gibbs Free Energy= 0.898697
Sum of electronic and zero-point Energies= -6609.898069
Sum of electronic and thermal Energies= -6609.844897
Sum of electronic and thermal Enthalpies= -6609.843953
Sum of electronic and thermal Free Energies= -6609.988540

uM06L-D3/def2-TZVPP-SMD(THF).
E(scf) = -6613.69181947 a.u.

^{4}TS3′
E(scf) = -6594.84274290 a.u.
ν_{min} = -292.13 cm^{-1}

Br	10.756018	8.365119	7.751448
Fe	9.646235	8.853105	9.881698
P	8.572564	11.040293	9.350591
P	11.218595	10.290240	11.143849
C	9.368309	12.354075	10.412523
H	9.266314	13.337913	9.938448
H	8.790417	12.390527	11.338436
C	10.849591	12.077112	10.740639
H	11.173707	12.714092	11.571922
H	11.472850	12.339916	9.884193
C	6.746003	11.316535	9.620427
H	6.513877	12.343759	9.306389
C	6.329982	11.169432	11.097821
H	6.608388	10.170440	11.452925
H	6.856818	11.894817	11.727760
C	4.813775	11.367983	11.263821
H	4.538341	11.228561	12.317546
H	4.558611	12.406692	11.005212
C	4.009996	10.411851	10.371975
H	2.934819	10.607786	10.477483
H	4.183362	9.379602	10.704541
C	4.435316	10.535203	8.902742
H	4.162180	11.531591	8.523726
H	3.896274	9.802767	8.287833
C	5.948700	10.328656	8.743973
H	6.231656	10.425873	7.688818
H	6.206349	9.307229	9.045700
C	8.885109	11.595029	7.584162
H	8.680259	10.690539	6.995107
C	8.001399	12.741401	7.055002
H	6.937609	12.505308	7.158385
H	8.186122	13.648985	7.648584
C	8.305650	13.025518	5.572695
H	8.015464	12.145214	4.980486
H	7.684967	13.860406	5.221457
C	9.791416	13.325223	5.332615
H	10.052581	14.272703	5.827854
H	9.981914	13.466096	4.260638
C	10.677460	12.206328	5.896126
H	11.739029	12.458989	5.773439
H	10.505824	11.277267	5.334133
C	10.371924	11.955722	7.379050
H	10.598040	12.873254	7.939999
H	11.017809	11.164953	7.766283
C	13.057222	10.141301	10.830325
H	13.570759	10.870030	11.472978
C	13.423348	10.448441	9.362218
H	12.842422	9.794091	8.703426
H	13.157755	11.479995	9.105153
C	14.921025	10.229011	9.096363
H	15.135663	10.430778	8.038829
H	15.509583	10.950078	9.683667
C	15.350511	8.803996	9.467708
H	14.814548	8.088650	8.826407
H	16.422966	8.663319	9.280650
C	15.017148	8.507119	10.934540
H	15.609219	9.168320	11.584814
H	15.293074	7.476503	11.192553
C	13.522590	8.721321	11.214472
H	13.316085	8.524365	12.273047
H	12.951291	7.989340	10.627885
C	11.024136	10.186053	12.998829
H	11.161039	9.121103	13.211815
C	12.032131	10.967323	13.859594
H	11.928082	12.045012	13.669153
H	13.060728	10.693860	13.598434
C	11.794104	10.684595	15.354520
H	12.016047	9.625583	15.552727
H	12.495565	11.272544	15.960634
C	10.347306	10.981547	15.774703
H	10.198991	10.721730	16.830756
H	10.161042	12.062350	15.685249
C	9.342395	10.227583	14.891656
H	9.448254	9.144905	15.052799
H	8.313987	10.492228	15.169831
C	9.579607	10.539524	13.407124
H	9.404160	11.610612	13.239981
H	8.858536	9.999285	12.782218
C	8.210599	7.536158	10.329871
C	7.464284	7.704042	11.522668
H	7.958692	8.087276	12.410897
C	6.115689	7.359630	11.599290

C	5.461806	6.796457	10.497371	C	8.839132	4.876078	13.861891
H	4.415895	6.509105	10.563139	H	8.192553	4.946032	11.816174
C	6.181304	6.601377	9.311165	C	10.797897	6.172591	14.405911
H	5.689268	6.169668	8.441828	C	9.766568	5.320984	14.809846
C	7.524546	6.963840	9.225420	H	8.037315	4.204584	14.159526
H	8.052190	6.816845	8.287956	H	11.542265	6.511057	15.122785
H	5.575987	7.519359	12.530546	H	9.691728	4.999121	15.845275
C	10.066178	6.620076	10.702208	C	10.887186	6.579746	13.073673
H	10.984004	7.197121	10.586385	H	11.712494	7.209272	12.760485
C	10.073351	5.514286	9.658121	C	11.498873	3.671574	8.558557
H	9.251472	4.807644	9.790384	C	12.789272	2.895489	8.548759
H	9.996187	5.942316	8.658333	H	12.908937	2.365388	9.496134
C	11.371497	4.727009	9.675818	H	12.760420	2.175816	7.726109
F	11.541565	4.056900	10.866980	H	13.628711	3.579831	8.407507
F	12.456287	5.570281	9.551419	F	10.430548	2.812429	8.698407
C	9.951156	6.164783	12.112820	F	11.327264	4.317748	7.357585
C	8.929329	5.291563	12.534540				

Zero-point correction= 1.001406 (Hartree/Particle)
Thermal correction to Energy= 1.054549
Thermal correction to Enthalpy= 1.055494
Thermal correction to Gibbs Free Energy= 0.911177
Sum of electronic and zero-point Energies= -6593.841337
Sum of electronic and thermal Energies= -6593.788194
Sum of electronic and thermal Enthalpies= -6593.787249
Sum of electronic and thermal Free Energies= -6593.931566

uM06L-D3/def2-TZVPP-SMD(THF).
E(scf) = -6597.64256837 a.u.

^{4}TS4
E(scf) = -6610.87211047 a.u.
ν_{min} = -142.49 cm^{-1}

Br	11.499752	8.589954	8.095933	H	6.703685	12.214305	9.935335
Fe	9.783737	8.639255	9.863202	C	6.751446	10.567262	11.328898
P	8.687383	10.921590	9.329291	H	7.033702	9.504271	11.322997
P	11.301779	10.258959	11.347152	H	7.424658	11.072457	12.026044
C	9.620128	12.249424	10.262726	C	5.304528	10.700444	11.828527
H	9.540103	13.204524	9.730937	H	5.220089	10.266072	12.833396
H	9.130599	12.391237	11.231410	H	5.054334	11.767790	11.922793
C	11.104942	11.881534	10.457554	C	4.315655	10.029645	10.867104
H	11.636774	12.690815	10.969476	H	3.285136	10.167842	11.219621
H	11.583755	11.748286	9.482399	H	4.507183	8.947368	10.851443
C	6.918616	11.135985	9.905193	C	4.476295	10.583265	9.445424

H	4.181593	11.643642	9.431342
H	3.804889	10.057310	8.754186
C	5.923574	10.447578	8.948619
H	6.000391	10.853051	7.935283
H	6.176977	9.388397	8.880304
C	8.677261	11.556014	7.560866
H	8.226395	10.728581	6.993976
C	7.848587	12.837607	7.324877
H	6.824527	12.734556	7.694008
H	8.302930	13.668223	7.883653
C	7.812302	13.199185	5.829560
H	7.285678	12.401512	5.285109
H	7.228783	14.118145	5.685465
C	9.222733	13.364060	5.248624
H	9.702288	14.238748	5.713432
H	9.170405	13.567524	4.171003
C	10.078979	12.121140	5.522422
H	11.104005	12.274605	5.159966
H	9.672420	11.264161	4.965163
C	10.105771	11.773448	7.018611
H	10.577229	12.602629	7.565775
H	10.716690	10.884619	7.185455
C	13.149767	9.891017	11.357315
H	13.554691	10.362908	12.263571
C	13.936159	10.454285	10.158259
H	13.510347	10.058534	9.230455
H	13.842358	11.546476	10.123026
C	15.422708	10.069716	10.242000
H	15.951896	10.454488	9.360191
H	15.876176	10.555289	11.119693
C	15.608049	8.550557	10.356354
H	15.233010	8.071476	9.440347
H	16.674460	8.300946	10.433565
C	14.839857	7.996004	11.563416
H	15.273772	8.406258	12.488117
H	14.948277	6.904965	11.619690
C	13.351126	8.365190	11.490657
H	12.830594	7.990599	12.381262
H	12.891727	7.870669	10.626360
C	11.065134	10.652958	13.174557
H	11.588289	9.813681	13.660164
C	11.726641	11.957025	13.663680
H	11.200956	12.814554	13.220889
H	12.770115	12.020740	13.333497
C	11.656176	12.064403	15.196783

H	12.250853	11.251823	15.639400
H	12.113537	13.006484	15.526253
C	10.209441	11.969564	15.702746
H	10.187310	11.991673	16.799901
H	9.650597	12.852036	15.357094
C	9.514995	10.704116	15.179140
H	9.994346	9.815238	15.615738
H	8.464432	10.685987	15.496865
C	9.599991	10.617823	13.647162
H	9.065435	11.474517	13.219120
H	9.088090	9.717199	13.289049
C	8.171828	7.496723	8.964074
C	7.070361	7.042172	9.730461
H	7.211508	6.851772	10.795400
C	5.816901	6.784063	9.170912
C	5.607009	6.972401	7.798978
H	4.635334	6.768733	7.355549
C	6.672197	7.420456	7.006973
H	6.523720	7.576530	5.939238
C	7.921350	7.671093	7.579487
H	8.735093	8.009937	6.939525
H	5.001715	6.431192	9.801046
C	9.689933	5.733647	9.214203
H	10.424832	6.307526	8.667203
C	9.048124	4.617574	8.447295
H	8.261331	4.116491	9.018326
H	8.579326	5.025778	7.544692
C	10.036619	3.564487	7.979386
F	10.682087	2.985242	9.051998
F	11.013772	4.142896	7.203456
C	9.772232	5.742080	10.640446
C	9.539047	4.611216	11.461537
C	9.614287	4.723796	12.839665
H	9.353338	3.648770	11.000476
C	10.142341	7.036310	12.555876
C	9.935303	5.963517	13.412743
H	9.444719	3.852771	13.467378
H	10.381378	8.015949	12.949504
H	10.009981	6.098515	14.486569
N	10.028909	6.962902	11.220114
C	9.429062	2.411192	7.157514
C	10.408093	1.376441	6.669950
H	10.910418	0.913090	7.521960
H	9.862885	0.614406	6.106352
H	11.151585	1.848290	6.023760

F	8.767985	2.976381	6.091218	F	8.468430	1.819446	7.947961

Zero-point correction= 0.988003 (Hartree/Particle)
Thermal correction to Energy= 1.041438
Thermal correction to Enthalpy= 1.042382
Thermal correction to Gibbs Free Energy= 0.896542
Sum of electronic and zero-point Energies= -6609.884107
Sum of electronic and thermal Energies= -6609.830673
Sum of electronic and thermal Enthalpies= -6609.829728
Sum of electronic and thermal Free Energies= -6609.975569

uM06L-D3/def2-TZVPP-SMD(THF).
E(scf) = -6613.67730207 a.u.

Product (Pyridine)
E(scf) = -1072.91700418 a.u.
ν_{min} = 23.43 cm^{-1}

C	8.121261	6.800143	10.555198	F	12.256446	6.717562	9.768904
C	7.611945	8.004178	11.064602	C	9.717595	5.866528	12.235563
H	8.274526	8.668702	11.614491	C	9.142977	4.632656	12.569014
C	6.273792	8.351660	10.880954	C	9.288121	4.147401	13.864741
C	5.416803	7.495981	10.182575	H	8.587482	4.072870	11.822467
H	4.373995	7.764899	10.036003	C	10.520359	6.133653	14.374526
C	5.911745	6.294441	9.673290	C	9.996405	4.912837	14.794175
H	5.254915	5.621671	9.127586	H	8.854999	3.191525	14.147049
C	7.253452	5.948428	9.859037	H	11.072847	6.763685	15.069822
H	7.616161	5.008536	9.453438	H	10.135521	4.576242	15.817031
H	5.900746	9.291214	11.280681	N	10.391251	6.610214	13.126147
C	9.581972	6.449987	10.830432	C	12.358828	4.636252	8.689640
H	10.152217	7.383198	10.839316	C	13.859013	4.533765	8.755780
C	10.191994	5.539937	9.745018	H	14.155354	4.054433	9.691299
H	9.808913	4.518288	9.807214	H	14.206605	3.934593	7.909916
H	9.938852	5.926733	8.753778	H	14.299864	5.531723	8.703825
C	11.702984	5.458898	9.818359	F	11.973020	5.204549	7.497378
F	12.111021	4.896898	11.005944	F	11.794114	3.381184	8.728286

Zero-point correction= 0.280765 (Hartree/Particle)
Thermal correction to Energy= 0.299279
Thermal correction to Enthalpy= 0.300223
Thermal correction to Gibbs Free Energy= 0.231697
Sum of electronic and zero-point Energies= -1072.636239
Sum of electronic and thermal Energies= -1072.617725
Sum of electronic and thermal Enthalpies= -1072.616781
Sum of electronic and thermal Free Energies= -1072.685307

uM06L-D3/def2-TZVPP-SMD(THF).
E(scf) = -1073.15728030 a.u.

Product (Phenyl)
E(scf) = -1056.87818619 a.u.
ν_{min} = 20.22 cm^{-1}

C	8.096081	6.813422	10.560019
C	7.640675	8.038024	11.072280
H	8.337408	8.681829	11.604868
C	6.313756	8.435985	10.913438
C	5.410963	7.611061	10.235709
H	4.376343	7.918708	10.108565
C	5.850810	6.390705	9.721780
H	5.159109	5.740956	9.191458
C	7.182468	5.995183	9.882531
H	7.500266	5.041717	9.471593
H	5.984770	9.390784	11.315842
C	9.550057	6.416000	10.804579
H	10.142225	7.335494	10.757710
C	10.097639	5.479632	9.705608
H	9.644928	4.486296	9.759664
H	9.865473	5.897332	8.721364
C	11.599983	5.279736	9.772120
F	11.948605	4.496499	10.846618
F	12.249458	6.483735	9.937998
C	9.732231	5.833048	12.205767
C	8.965589	4.742956	12.639581
C	9.149607	4.204947	13.913506
H	8.214129	4.319214	11.977894
C	10.869438	5.840897	14.354856
C	10.102356	4.753544	14.777502
H	8.546103	3.359375	14.234075
H	11.613441	6.275028	15.018141
H	10.243437	4.336948	15.771468
C	10.684684	6.374202	13.076788
H	11.294383	7.211706	12.745955
C	12.212119	4.615886	8.520628
C	13.692427	4.352062	8.589605
H	13.909508	3.677777	9.420651
H	14.010081	3.891423	7.650337
H	14.228466	5.292480	8.736192
F	11.925179	5.430216	7.448928
F	11.527395	3.439497	8.315355

Zero-point correction= 0.292469 (Hartree/Particle)
Thermal correction to Energy= 0.311120
Thermal correction to Enthalpy= 0.312064
Thermal correction to Gibbs Free Energy= 0.243331
Sum of electronic and zero-point Energies= -1056.585717
Sum of electronic and thermal Energies= -1056.567066
Sum of electronic and thermal Enthalpies= -1056.566122
Sum of electronic and thermal Free Energies= -1056.634856

uM06L-D3/def2-TZVPP-SMD(THF).
E(scf) = -1057.11188209 a.u.

16. References

1. Smith, J. J.; Best, D.; Lam, H. W. Copper-catalyzed borylative coupling of vinylazaarenes and N-Boc imines. *Chem. Commun.* **2016**, *52*, 3770-3772.

2. Li, J.; Chen, J.; Jiao, W.; Wang, G.; Li, Y.; Cheng, X.; Li, G. Difluoroalkylation/C–H Annulation Cascade Reaction Induced by Visible-Light Photoredox Catalysis. *J. Org. Chem.* **2016**, *81*, 9992-10001.

3. Andersen, T. L.; Frederiksen, M. W.; Domino, K.; Skrydstrup, T. Direct Access to α,α-Difluoroacylated Arenes by Palladium-Catalyzed Carbonylation of (Hetero)Aryl Boronic Acid Derivatives. *Angew. Chem. Int. Ed.* **2016**, *55*, 10396-10400.

4. Nugent, J.; Shire, B. R.; Caputo, D. F. J.; Pickford, H. D.; Nightingale, F.; Houlsby, I. T. T.; Mousseau, J. J.; Anderson, E. A. Synthesis of All-Carbon Disubstituted Bicyclo[1.1.1]pentanes by Iron-Catalyzed Kumada Cross-Coupling. *Angew. Chem. Int. Ed.* **2020**, *59*, 11866-11870.

5. van de Wouw, H. L.; Lee, J. Y.; Klausen, R. S. Gram-scale free radical polymerization of an azaborine vinyl monomer. *Chem. Commun.* **2017**, *53*, 7262-7265.

6. (a) Lee, C.; Yang, W.; Parr, R. G. Development of the Colle-Salvetti Correlation-Energy Formula into a Functional of the Electron Density. *Phys. Rev. B* **1988**, *37*, 785–789. (b) Becke, A. D. Density Functional Thermochemistry. III. The Role of Exact Exchange. *J. Chem. Phys.* **1993**, *98*, 5648–5652.

7. (a) Grimme, S. Accurate description of van der Waals complexes by density functional theory including empirical corrections. *J. Comput. Chem.* **2004**, *25*, 1463–1473. (b) Grimme, S.; Antony, J.; Ehrlich, S.; Krieg, H. A consistent and accurate ab initio parametrization of density functional dispersion correction (DFT-D) for the 94 elements H-Pu. *J. Chem. Phys.* **2010**, *132*, 154104. (c) Grimme, S. Density functional theory with London dispersion corrections. *WIRES Comput. Mol. Sci.* **2011**, *1*, 211-228. (d) Ehrlich, S.; Moellmann, J.; Grimme, S. Dispersion-Corrected Density Functional Theory for Aromatic Interactions in Complex Systems. *Acc. Chem. Res.* **2012**, *46*, 916–926.

8. (a) Petersson, G. A.; Tensfeldt, T. G.; Montgomery, J. A., Jr. A Complete Basis Set Model Chemistry. III. The Complete Basis Set-quadratic Configuration Interaction Family of Methods. *J. Chem. Phys.* **1991**, *94*, 6091–6101. (b) Petersson, G. A.; Bennett, A.; Tensfeldt, T. G.; Al-Laham, M. A.; Shirley, W. A.; Mantzaris, J. A Complete Basis Set Model Chemistry. I. The Total Energies of Closed-shell Atoms and Hydrides of the First-row Elements. *J. Chem. Phys.* **1988**, *89*, 2193–2218.

9. Gaussian 16, Revision C.01, Frisch, M. J.; Trucks, G. W.; Schlegel, H. B.; Scuseria, G. E.; Robb, M. A.; Cheeseman, J. R.; Scalmani, G.; Barone, V.; Petersson, G. A.; Nakatsuji, H.; Li, X.; Caricato, M.; Marenich, A. V.; Bloino, J.; Janesko, B. G.; Gomperts, R.; Mennucci, B.; Hratchian, H. P.; Ortiz, J. V.; Izmaylov, A. F.; Sonnenberg, J. L.; Williams-Young, D.; Ding, F.; Lipparini, F.; Egidi, F.; Goings, J.; Peng, B.; Petrone, A.; Henderson, T.; Ranasinghe, D.; Zakrzewski, V. G.; Gao, J.; Rega, N.; Zheng, G.; Liang, W.; Hada, M.; Ehara, M.; Toyota, K.; Fukuda, R.; Hasegawa, J.; Ishida, M.; Nakajima, T.; Honda, Y.; Kitao, O.; Nakai, H.; Vreven, T.; Throssell, K.; Montgomery, J. A., Jr.; Peralta, J. E.; Ogliaro, F.; Bearpark, M. J.; Heyd, J. J.; Brothers, E. N.; Kudin, K. N.; Staroverov, V. N.; Keith, T. A.; Kobayashi, R.; Normand, J.; Raghavachari, K.; Rendell, A. P.; Burant, J. C.; Iyengar, S. S.; Tomasi, J.; Cossi, M.; Millam, J. M.; Klene, M.; Adamo, C.; Cammi, R.; Ochterski, J. W.; Martin, R. L.; Morokuma, K.; Farkas, O.; Foresman, J. B.; Fox, D. J. Gaussian, Inc., Wallingford CT, 2016.

10. (a) Liu, L.; Aguilera, M. C.; Lee, W.; Youshaw, C. R.; Neidig, M. L.; Gutierrez, O. General Method for Iron-catalyzed Multicomponent Radical Cascades Cross-couplings. *Science.* **2021**, *374*, 432−439. (b) Youshaw, C. R.; Yang, M. H.; Gogoi, A. R.; Rentería-Gómez, A.; Liu, L.; Morehead, L. M.; Gutierrez, O. Iron-Catalyzed Enantioselective Multicomponent Cross-Couplings of α-Boryl Radicals. *Org. Lett*. **2023**, *25*, 8320-8325. (c) Lee, W.; Zhou, J.; Gutierrez, O. Mechanism of Nakamura′s Iron-Catalyzed Asymmetric Cross-coupling Reaction: The Role of Spin in Controlling Selectivity. *J. Am. Chem. Soc.* **2017**, *139*, 16126−16133. (d) Aguilera, M. C.; Gogoi, A. R.; Lee, W.; Liu, L.; Brennessel, W.; Gutierrez, O.; Neidig, M. L. Insight into Radical Initiation, Solvent Effects and Biphenyl Production in Iron-Bisphosphine Cross-Couplings. *ACS Catal.* **2023**, *13*, 8987−8996. (e) Renteria-Gomez, A.; Lee, W.; Yin, S.; Davis, M.; Gogoi, A. R; Gutierrez, O. General and Practical Route to Diverse 1-(Difluoro)alkyl-3-aryl Bicyclo[1.1.1]pentanes Enabled by an Fe-Catalyzed Multicomponent Radical Cross-Coupling Reaction. *ACS Catal.* **2022***, 12*, 11547-11556.
11. Marenich, A. V.; Cramer, C. J.; Truhlar, D. G. Universal Solvation Model Based on Solute Electron Density and on a Continuum Model of the Solvent Defined by the Bulk Dielectric Constant and Atomic Surface Tensions. *J. Phys. Chem. B* **2009**, *113*, 6378–6396.
12. Y. Zhao and D. G. Truhlar, "A new local density functional for main-group thermochemistry, transition metal bonding, thermochemical kinetics, and noncovalent interactions," *J. Chem. Phys.*, **125** (2006), 194101: 1-18.
13. (a) Weigend, F.; Ahlrichs, R. Balanced basis sets of split valence, triple zeta valence and quadruple zeta valence quality for H to Rn: Design and assessment of accuracy. *Phys. Chem. Chem. Phys.* **2005**, *7*, 3297-3305. (b) Weigend, F. Accurate Coulomb-fitting basis sets for H to Rn. *Phys. Chem. Chem. Phys.* **2006**, *8*, 1057-1065.
14. Legault, C. Y. (2009) CYLview, 1.0b, Universite de Sherbrooke: Sherbrooke, Canada, http://www.cylview.org.
15. Lu, T.; Chen, F., Multiwfn: A multifunctional wavefunction analyzer. *J. Comp. Chem.* **2012**, *33*, 580-592.
16. Humphrey, W.; Dalke, A.; Schulten, K., VMD – Visual Molecular Dynamics. *J. Mol. Graphics* **1996**, *14*, 33-38.
17. NBO Version 3.1, Glendening, E.; Reed, A.; Carpenter, J.; Weinhold. F.
18. Bickelhaupt, F. M.; Houk, K. N. *Angew. Chem. Int. Ed.* **2017**, *56*, 10070-10086.
